# Chemical Targeting and Manipulation of Type III Secretion in the Phytopathogen Xanthomonas campestris for Control of Disease

**DOI:** 10.1128/AEM.02349-19

**Published:** 2020-01-21

**Authors:** Le Zhou, Cheng Wang, Guo-Hua Wang, Zai-Wa Wei, Qiu-Xia Fu, Xiao-Hong Hang, Mei Yang, Bo-Le Jiang, Ji-Liang Tang

**Affiliations:** a State Key Laboratory for Conservation and Utilization of Subtropical Agro-bioresources, College of Life Science and Technology, Guangxi University, Nanning, Guangxi, China; University of Queensland

**Keywords:** *Xanthomonas*, type III secretion, small molecule, inhibitor, virulence

## Abstract

Xanthomonas campestris pv. campestris is the causative agent of black rot disease in crucifer plants. This Gram-negative bacterium utilizes the type III secretion system (T3SS), encoded by the *hrp* gene cluster, to aid in its resistance to host defenses and the ability to cause disease. The T3SS injects a set of proteins known as effectors into host cells that come into contact with the bacterium. The T3SS is essential for the virulence and hypersensitive response (HR) of X. campestris pv. campestris, making it a potential target for disease control strategies. Using a unique and straightforward high-throughput screening method, we examined a large collection of diverse small molecules for their potential to modulate the T3SS without affecting the growth of X. campestris pv. campestris. Screening of 13,129 different compounds identified 10 small molecules that had a significant inhibitory influence on T3SS. Moreover, reverse transcription-quantitative PCR (qRT-PCR) assays demonstrated that all 10 compounds repress the expression of the *hrp* genes. Interestingly, the effect of these small molecules on *hrp* genes may be through the HpaS and ColS sensor kinase proteins that are key to the regulation of the T3SS *in planta*. Five of the compounds were also capable of inhibiting X. campestris pv. campestris virulence in a Chinese radish leaf-clipping assay. Furthermore, seven of the small molecules significantly weakened the HR in nonhost pepper plants challenged with X. campestris pv. campestris. Taken together, these small molecules may provide potential tool compounds for the further development of antivirulence agents that could be used in disease control of the plant pathogen X. campestris pv. campestris.

**IMPORTANCE** The bacterium Xanthomonas campestris pv. campestris is known to cause black rot disease in many socioeconomically important vegetable crops worldwide. The management and control of black rot disease have been tackled with chemical and host resistance methods with variable success. This has motivated the development of alternative methods for preventing this disease. Here, we identify a set of novel small molecules capable of inhibiting X. campestris pv. campestris virulence, which may represent leading compounds for the further development of antivirulence agents that could be used in the control of black rot disease.

## Abstract (precis)

The bacterium Xanthomonas campestris pv. campestris is known to cause black rot disease in many socioeconomically important vegetable crops worldwide. The management and control of black rot disease have been tackled with chemical and host resistance methods with variable success. This has motivated the development of alternative methods for preventing this disease. Here, we identify a set of novel small molecules capable of inhibiting X. campestris pv. campestris virulence, which may represent leading compounds for the further development of antivirulence agents that could be used in the control of black rot disease.

## INTRODUCTION

Plant diseases caused by bacterial pathogens place major restrictions on crop production and cause significant losses on a global scale annually (1, 2). The planting of resistant varieties of crops represents the most attractive option for bacterial disease control (3, 4). However, this is not always an option, as resistance varieties are not always available or sustainable. In these situations, chemicals have been used for the effective management of bacterial plant disease. Plant disease management using chemicals can be very problematic because of the limited number of available bactericides and the continued failure of traditional bactericides due to resistance development. This current situation has driven the search for new chemical compounds that can interfere with bacterial processes and disease progression in unique ways. One approach that has gained notoriety is the development of antivirulence agents that inhibit bacterial virulence factors rather than bacterial growth and survival, allowing the host to clear the infecting bacteria (5–7).

Numerous Gram-negative bacterial plant pathogens, including *Pseudomonas*, *Erwinia*, and *Xanthomonas* species, utilize type III secretion systems (T3SSs) to inject effector proteins directly into host cells to suppress defense responses. The T3SS is a complex apparatus composed of 20 to 25 different proteins, which consists of an extracellular pilus-like (plant pathogens) or needle-like (animal pathogens) appendage, a membrane-spanning basal body, and the peripheral inner membrane cytoplasmic component (1, 8–10). The disruption of the T3SS has been reported to lead to a complete loss of virulence without influencing bacterial growth (11–13). The T3SS apparatus and the effector proteins that it secretes have been considered potentially valuable targets for developing antivirulence agents. Several studies have been conducted to identify T3SS inhibitors in animal and human bacterial pathogens with variable success (14–16). More recently, a few studies have focused on identifying agents that inhibit the T3SS in plant bacterial pathogens (3, 4, 17–20). Khokhani et al. identified several plant phenolic compounds that act as T3SS inhibitors of Erwinia amylovora (the causal agent of fire blight) (17). Yang et al. identified four small-molecule compounds that belong to salicylidene acylhydrazide class, which could either strongly or moderately suppress the T3SS gene expression of E. amylovora (18). Two plant phenolic compounds, i.e., *p*-coumaric acid and *trans*-4-hydroxycinnamohydroxamic acid, were reported to show significant inhibition of the Dickeya dadantii (the causal agent of soft-rot disease) T3SS (19, 20). Additionally, a number of phenolic compounds were shown to significantly inhibit the Xanthomonas oryzae pv. oryzae (the rice leaf blight pathogen) T3SS (3, 4). Despite identifying molecules that interfere with the T3SSs in these bacterial plant pathogens, those studies were limited because of the small number of compounds screened and the follow-up tests carried out on active compounds mainly being *in vitro* in nature. Additionally, the spectrum of activity of these T3SS inhibitors against other plant pathogens has not been demonstrated. This argues for the need for additional direct screening against other specific bacterial plant pathogens.

Xanthomonas campestris pv. campestris is the causal agent of black rot disease of cruciferous crops worldwide (2). The Cruciferae family of plants is composed of approximately 338 genera worldwide (1, 2). These plants are of significant socioeconomic importance, as many are popular vegetable crops for human and animal consumption, including mustard, collards, rutabaga, turnip, cabbage, broccoli, cauliflower, sprout, radish, and kale. The main yield-limiting and destructive pathogen of these cruciferous crops worldwide is X. campestris pv. campestris. X. campestris pv. campestris disease (black rot) contributes to quantitative and qualitative reductions in vegetable crop production and decreases the plant yield and nutritional value. The management and control of black rot disease have been tackled with chemical, biological control, and host resistance methods with variable success, which has motivated the development of alternatives to control this disease in cruciferous crops. Like many other bacterial pathogens, the pathogenicity of X. campestris pv. campestris relies upon a T3SS. The proteins composing the X. campestris pv. campestris T3SS apparatus are encoded by a cluster of *hrp* (hypersensitive response and pathogenicity) genes (21, 22). These genes are directly activated by a key regulator named HrpX, an AraC-type transcriptional regulator (21, 22). The expression of *hrpX* is regulated by a two-component signal transduction system (TCS) consisting of the sensor histidine kinase HpaS and the response regulator HrpG (23). Approximately 30 X. campestris pv. campestris effector proteins have been identified by experimental assays or bioinformatic prediction (24–27). The *hrp* genes and the genes encoding effector proteins are expressed weakly in nutrient-rich media but are induced in certain minimal media and *in planta* (28, 29). Additionally, the deletion of the genes encoding HrpX (regulator) or XopN (effector protein) significantly reduces the virulence of X. campestris pv. campestris (28, 29).

In this work, we developed and applied a high-throughput cell-based luciferase reporter assay for the identification of small molecules that modulate the X. campestris pv. campestris T3SS. A total of 13,129 different small molecules were screened for their ability to modulate the transcription of the X. campestris pv. campestris *xopN* gene without affecting growth. Active compounds were tested for their ability to modulate T3SS functionality *in vitro* and *in vivo*. Additionally, these active compounds were tested for their influence on the X. campestris pv. campestris hypersensitive response (HR) in nonhost pepper plants and virulence in the host plant Chinese radish. This study identifies potential antivirulence compounds that could be used in the control of black rot disease and as potential tool molecules that can be used to study virulence regulation in X. campestris pv. campestris.

## RESULTS

### Development of a luciferase-based reporter system to monitor *xopN* transcription in X. campestris pv. campestris.

In order to identify compounds that potentially modulate the T3SS in the model strain X. campestris pv. campestris 8004, we established a high-throughput luciferase bioreporter assay. We constructed a bioreporter strain that allowed the library of small molecules to be screened for their effects on the promoter activity of the *xopN* gene, which encodes an effector protein important for the virulence of X. campestris pv. campestris (25, 27). This was achieved by generating the reporter construct pXopNlux (Table 1), by fusing a 647-bp DNA segment containing the promoter and the effector signal sequence of the gene encoding XopN to the 5′ end of the promoterless *luxAB* gene that had been cloned into pLAFR6 (Table 1). The reporter construct was then introduced into X. campestris pv. campestris wild-type strain 8004, generating a bioreporter strain named 8004/pXopNlux (Table 1). The luciferase activity produced by the bioreporter strain gives a measure of *xopN* promoter activity and/or protein expression efficiency in X. campestris pv. campestris. In addition, the expression of *xopN* in strain 8004 is positively controlled by the key *hrp* regulator HrpX (25); therefore, the luciferase activity may also reflect the expression of *hrpX*. Simultaneously, a promoterless *luxAB* construct (pLux) (Table 1) was constructed as a control. Strain 8004 carrying this construct (8004/pLux) (Table 1) did not produce significant luciferase activity (see Fig. S1 in the supplemental material).

**TABLE 1:** Bacterial strains and plasmids used in this work^*a*^

Strain or plasmid	Relevant characteristics	Reference or source
Strains		
* *X. campestris pv. campestris		
* *8004	Wild-type strain; Rif^r^	28
8004/pLux	8004 harboring plasmid pLux; Rif^r^ Tc^r^	This work
8004/pXopNlux	8004 harboring plasmid pXopNlux; Rif^r^ Tc^r^	This work
Δ*hrcV*	Same as 8004 but with an *hrcV* deletion; Rif^r^	29
* *E. coli		
DH5α	*hsdR17*(r^−^ m^+^) *supE44 thi-1 recA1 gyrA96* (Nal^r^) *relA1 Δ*(*lacZYA-argF*)*U169* (*lacZΔ*M15)	30
ED8767	*recA met*	31
FJAT-333	DH5α harboring plasmid pUT*gfpluxAB*; Amp^r^ Kan^r^	32

Plasmids		
pUT*gfpluxAB*	Plasmid containing the *luxAB* gene; Amp^r^ Kan^r^	32
pET-30a(+)	Prokaryotic expression vector; Kan^r^	Novagen
pK18mob	Suicide plasmid in X. campestris pv. campestris; Kan^r^	33
pLAFR6	Broad-host-range cloning vector; Tc^r^	34
pRK2073	Helper plasmid; Tra^+^ Mob^+^ ColE1 Spc^r^	35
pLux	pLAFR6 containing the *luxAB* gene; Tc^r^	This work
pKxopN	pK18mob harboring the promoter and the signal sequence of the X. campestris pv. campestris T3SE gene *xopN* (*XC_0241*); Kan^r^	This work
pXopNlux	pLAFR6 with *luxAB* driven by the promoter and the signal sequence of the X. campestris pv. campestris T3SE gene *xopN* (*XC_0241*)	This work

a Rif^r^, Tc^r^, Kan^r^, Spc^r^, and Amp^r^ indicate resistance to rifampin, tetracycline, kanamycin, spectinomycin, and ampicillin, respectively. T3SE, type III secreted effector.

**FIG 1:**
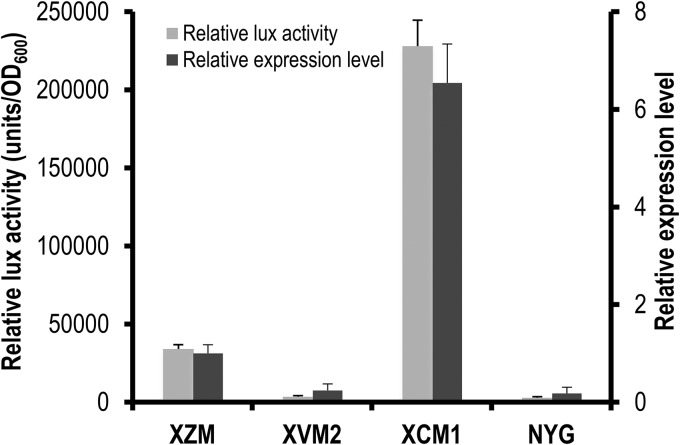
Luciferase (lux) activity produced by strain 8004/pXopNlux and its *xopN* gene expression levels under various conditions. The strain was grown in NYG medium overnight. Bacterial cells were collected; suspended to an optical density at 600 nm of 0.05 in the minimal medium XVM2, XCM1, or XZM or the rich medium NYG; and distributed into 96-well plates. The luciferase activity was assayed after incubation at 28°С with shaking (600 rpm) for 16 h. Simultaneously, the relative expression level of the *xopN* gene of strain 8004/pXopNlux in the different media was determined by qRT-PCR. Values given are the means and standard deviations from triplicate measurements. Data presented were obtained from a representative experiment, and similar results were obtained in two other independent experiments.

As X. campestris pv. campestris T3SS genes are expressed weakly in nutrient-rich media but are induced in specific minimal media and *in planta* (36, 37), it was important to select an optimal medium for the high-throughput screening assay. We compared the luciferase activities produced by the bioreporter strain in the minimal media XVM2, XCM1, and XZM (media are described in Materials and Methods). Bacterial cells of the bioreporter strain were suspended to an optical density at 600 nm (OD_600_) of 0.05 in the media and incubated in 96-well plates at 28°С with shaking (600 rpm) for 16 h. As shown in Fig. 1, the luciferase activity produced in XVM2 medium was relatively low, suggesting that this medium might be suitable only for screening for compounds that have an induction effect on T3SS. On the contrary, XCM1 medium gave very high luciferase activity, suggesting that it might be suitable for screening for compounds that inhibit the T3SS but not compounds that potentially have an induction effect. Although the bacterial cells grew similarly in the three media, XZM medium produced a mean luciferase activity value between those of XVM2 and XCM1 media (Fig. 1), which seemed most appropriate to potentially capture compounds that both inhibit and induce expression. qRT-PCR (reverse transcription-quantitative PCR) confirmed that the expression levels of the reporter gene *xopN* in the media were similar to the luciferase activities (Fig. 1).

### High-throughput screen to identify small molecules that modulate the *Xanthomonas* T3SS.

The inventory of compounds that we could screen consisted of 17,045 small molecules, which was acquired from the National Compound Resource Center of China (http://www.chemicallibrary.org.cn/). This collection of small molecules was derived from 26 sublibraries and composed of 13,126 unique molecules, with the remaining 3,919 being duplicates or derivatives. The set was assembled from the core collection with the aim of maximizing chemical diversity. Using the luciferase-based reporter system developed, we screened the 13,126 different small molecules. Additionally, we included imidocarb, 4,4′-thiobis(2-methylphenol), and benzoic acid, which have been identified as potent inhibitors of the T3SS in the human pathogen Yersinia pestis and the plant pathogen Erwinia amylovora (17, 38, 39). Taken together, a total of 13,129 different compounds were examined. Briefly, bacterial cells of the bioreporter strain 8004/pXopNlux were suspended to an OD_600_ of 0.05 in XZM medium and incubated in 96-well plates supplemented with each of the compounds at a final concentration of 30 μM for 16 h, followed by a luciferase activity assay. The reporter strain cultured in medium supplemented with 0.3% (vol/vol) dimethyl sulfoxide (DMSO) was used as a control.

Molecules were excluded if they significantly suppressed bacterial cell growth (*P < *0.05 by a *t* test), and those without a significant effect on growth were subjected to a luciferase activity assay. Molecules were judged to inhibit or induce luciferase activity significantly in the reporter strain when the average activity was <50% or ≥2-fold relative to the control (supplemented with 0.3% [vol/vol] DMSO). Molecules identified as inhibitor or inducer candidates were carried forward for further tests.

In the first round of screening, an experiment with three replicates was carried out for each of the compounds. As shown in Table S1, 630 out of the 13,126 compounds exhibited significant suppression of bacterial growth (*P < *0.05 by a *t* test) after incubation in XZM medium for 16 h (see Materials and Methods for details). Of the remaining 12,496 compounds, the average luciferase activities of 116 and 64 molecules were <50% and >2-fold relative to the control, respectively (Table S1). These inhibitor or inducer candidates were subjected to a second round of screening to confirm their action. In this round, all of the compounds were tested three times (each with three replicates). As shown in Table S1, of the 180 candidates, only 24 had an average luciferase activity of <50% and 11 had an average luciferase activity of >2-fold relative to the control (Table S1).

As shown in Table S2, the previously identified inhibitors 4,4′-thiobis(2-methylphenol) and benzoic acid did not display any significant effect on the luciferase activity or the growth of the bioreporter strain at concentrations ranging from 5 to 100 μM. This suggested that the compounds did not affect the T3SS of X. campestris pv. campestris at the concentrations tested. However, imidocarb did not affect the growth of the bioreporter strain at concentrations ranging from 5 to 20 μM but produced only 14 to 17% luciferase activity compared to the control (DMSO) (Table S2). It should be noted that at a concentration of >30 μM, imidocarb suppressed the growth of the bioreporter strain (Table S2). These data suggest that under the conditions tested, imidocarb can inhibit reporter activity, but 4,4′-thiobis(2-methylphenol) and benzoic acid cannot. Imidocarb and the other 35 potent small molecules were taken for further studies.

### The potencies of molecules that modulate the *Xanthomonas* T3SS are different under various growth conditions and concentrations.

**FIG 2:**
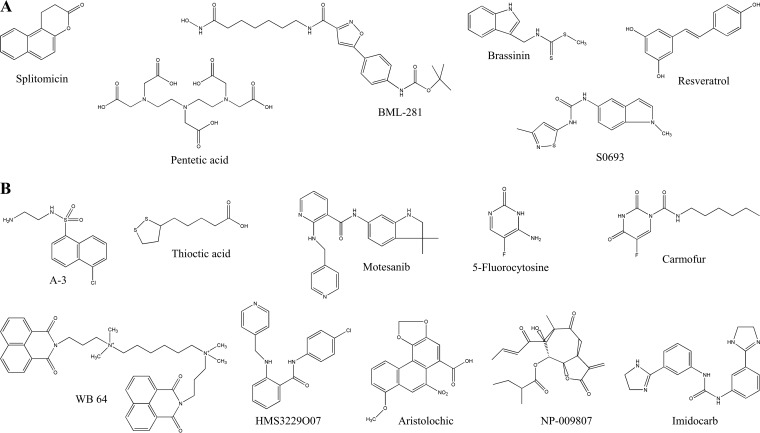
The structures of the compounds identified to modulate *xopN* expression under various growth conditions. These 16 compounds demonstrated potency in all of the media tested. (A) Compounds with an inductive effect on *xopN* expression. (B) Compounds with an inhibitory effect on *xopN* expression.

The luciferase activity produced by the bioreporter strain 8004/pXopNlux varied in the minimal media XVM2, XCM1, and XZM. This is consistent with the contention that *hrp* genes are activated depending on the plant environment (36, 37). We examined the impacts of various conditions on the potencies of imidocarb and the other 35 small molecules that modulated the luciferase activity of the bioreporter strain. For this, the 24 potential inhibitors as well as imidocarb were examined in XCM1 medium for an influence on the bioreporter strain, while the 11 potential inducer candidates were examined in XVM2 medium. The final concentration of the compounds used was 30 μM (apart from imidocarb, whose concentration was 5 μM). Interestingly, under the different conditions, 10 of the potential inhibitors (10/25) and 1 inducer (1/11) significantly suppressed bacterial growth (*P < *0.05 by a *t* test) (Table S3). Additionally, 10 inhibitor candidates and 6 inducer candidates had average luciferase activities of <50% and >2-fold relative to the control (Table S3), respectively. As expected, the reporter strain 8004/pXopNlux produced very low luciferase activity (2,165 U/OD_600_) in nutrient-rich NYG medium; however, in NYG medium supplemented with any of the 6 inducer candidate compounds, it produced significantly higher luciferase activity (7,925 to 13,407 U/OD_600_) (Fig. S2). These results reveal that the effects of the compounds on X. campestris pv. campestris growth and T3SS expression vary under different conditions. The 16 compounds that demonstrated potency in all of the media tested were taken for further study. The structures of these compounds are shown in Fig. 2.

To determine the optimal dose of the small molecules described above, a detailed dose-response assay was carried out in XZM medium. For this, various concentrations of target small molecules ranging from 5 to 120 μM were tested for their effect on the luciferase activity of the bioreporter strain (8004/pXopNlux). The optimal dosage was selected, where the lowest concentration of the target molecule had a maximum effect on the luciferase activity of the bioreporter strain without influencing bacterial growth, or its influence was significantly better (*P ≤ *0.05 by a *t* test) than at lower concentrations (Fig. S3).

As shown in Table S4, all of the compounds did not affect bacterial growth at concentrations of ≤40 μM, although some of them significantly repressed bacterial growth at concentrations of ≥80 μM (*P ≤ *0.05 by a *t* test). As a consequence, the defined optimal dosages were 5 μM for carmofur, NP-009807, and imidocarb; 10 μM for pentetic acid, A-3, and HMS3229O07; 20 μM for splitomicin, S0693, BML-281, resveratrol, motesanib, 5-fluorocytosine, and WB 64; 30 μM for brassinin and aristolochic acid; and 40 μM for thioctic acid (Fig. S3).

### Small molecules influence the expression of regulatory genes of the *hrp* system and associated effectors.

**FIG 3:**
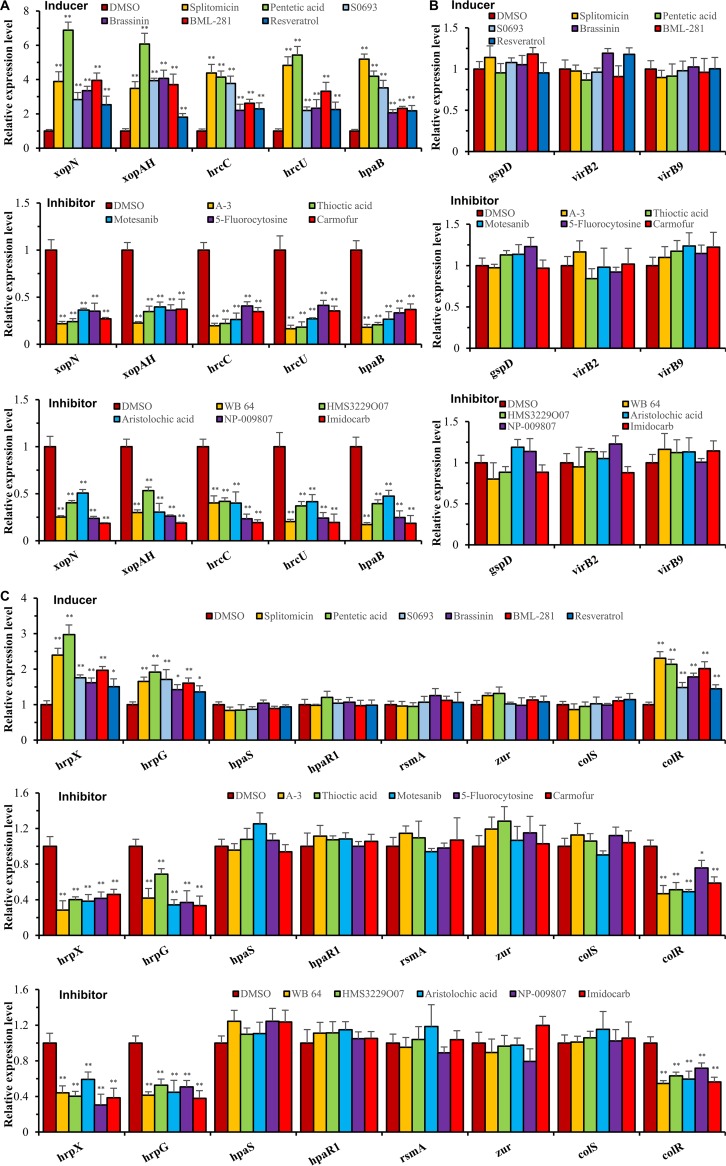
Effects of the selected compounds on the expression of target genes in X. campestris pv. campestris. X. campestris pv. campestris strain 8004 was grown for 20 h in XZM medium in the presence of inducers and inhibitors using the optimal dosages of the compounds. Bacterial cells were collected, and relative mRNA levels of a number of genes were measured by qRT-PCR. The relative mRNA levels of each gene were calculated with respect to the mRNA level of the gene in the strain grown in XZM medium supplemented with an equivalent volume of DMSO (equaling 1). The expression level of the 16S rRNA gene was used as an internal control for data analysis. Three replicates were used in each experiment. The experiment was repeated three times, and similar results were obtained. Asterisks indicate statistically significant differences (by Student’s *t* test) (*, *P < *0.05; **, *P < *0.01). (A) Two T3SE genes (*xopN* and *xopAH*), two *hrp* genes (*hrcC* and *hrcU*), and the T3SE chaperone gene *hpaB*. (B) The T2SS gene *gspD* and two T4SS genes (*virB2* and *virB9*). (C) Eight *hrp* regulatory genes (*hrpX*, *hrpG*, *hpaS*, *hpaR1*, *rsmA*, *zur*, *colS*, and *colR*).

Several molecules identified using the luciferase-based reporter system may show promise as agents that can influence the efficiency of the T3SS of X. campestris pv. campestris. To clarify and gain a better understanding of the influence of these small molecules on X. campestris pv. campestris physiology, we performed qRT-PCR to assess the impact of the small molecules on the expression of genes that encode elements of the X. campestris pv. campestris T3SS apparatus and associated effector proteins. Target genes, which included *hrcC* (*XC_3003*), *hrcU* (*XC_3012*), and *hpaB* (*XC_3022*) in the *hrp* cluster as well as *xopN* (*XC_2041*) and *xopAH* (*XC_2004*), were taken as representatives in the study. The genes *xopN* and *xopAH* encode effectors that contribute to the full virulence of X. campestris pv. campestris (24, 25). The genes *hrcC*, *hrcU*, and *hpaB* are parts of various operons in the *hrp* gene cluster (40). qRT-PCR demonstrated that the expression of all tested genes was significantly altered when X. campestris pv. campestris was incubated for 20 h in XZM medium supplemented with the optimal dosage of each of the small molecules (Fig. 3). The expression levels of the genes in X. campestris pv. campestris exposed to the inducer molecules were at least 2-fold higher than those of the control (Fig. 3A). In contrast, inhibitor molecules suppressed the expression levels of the genes to <50% relative to the control (Fig. 3A).

A second set of qRT-PCR experiments was carried out to examine the influence of the small molecules on a number of *hrp* regulatory genes (*colS*, *colR*, *hpaS*, *hpaR1*, *hrpG*, *hrpX*, *rsmA*, and *zur*). *colS* and *colR* encode a TCS that positively regulates the expression of the *hrpC* and *hrpE* operons (41). *hpaR1* encodes a GntR family transcriptional regulator that positively controls the expression of *hrpG* (42). *rsmA* encodes the RNA-binding posttranscriptional regulator RsmA, which affects the expression of the *hrp* gene cluster (43, 44). The protein encoded by *zur* is a key regulator of zinc homeostasis, which positively regulates the expression of *hrpX* (22). As shown in Fig. 3C, the expression levels of *hrpX*, *hrpG*, and *colR* were significantly increased or reduced in relation to the inducer or inhibitor small molecules to which X. campestris pv. campestris was exposed. However, the expression levels of other tested regulatory genes, i.e., *hpaS*, *hpaR1*, *rsmA*, *zur*, and *colS*, were not significantly (*P > *0.05 by a *t* test) influenced by any of the inducer and inhibitor small molecules (Fig. 3C). In addition, one T2SS (type II secretion system)-encoding gene (*gspD*) and two T4SS (type IV secretion system)-encoding genes (*virB2* and *virB9*) were also included in the experiment. None of the inducer and inhibitor molecules appeared to significantly (*P ≤ *0.05 by a *t* test) influence the expression of these genes (Fig. 3B). Taken together, the data suggest that under the conditions tested, the small molecules appear to influence the expression of X. campestris pv. campestris T3SS apparatus-encoding genes and some T3SS regulatory genes and T3SS effector genes tested but without effects on T2SS and T4SS.

### Small molecules influence X. campestris pv. campestris HR induction in nonhost pepper plants.

The ability of X. campestris pv. campestris to induce HR in a nonhost plant is a direct indication that the T3SS is functional and active (27). It is known that the X. campestris pv. campestris strain 8004 triggers an AvrBs1-dependent HR in nonhost pepper (Capsicum annuum cv. ECW-10R) plants (27). AvrBs1 is encoded by *XC_2081* and secreted by the T3SS into plant cells (27). We tested the influence of the small molecules on the HR-inducing ability of X. campestris pv. campestris on nonhost pepper plants. In brief, bacterial cells were collected and suspended in 0.3% DMSO with or without the target small molecule at its optimal concentration. The bacterial cells in the suspension were adjusted to an optical density at 600 nm of 0.01. The pepper leaves were inoculated by infiltrating a 5-μl volume of the bacterial suspension into the abaxial leaf surface using a blunt-end plastic syringe.

**FIG 4:**
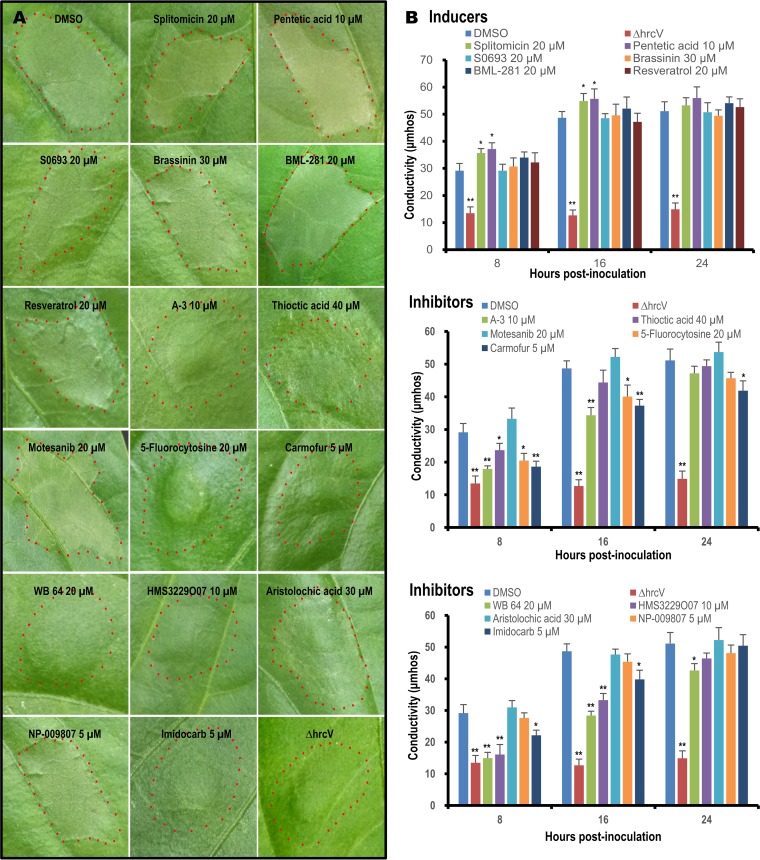
Effects of selected compounds on X. campestris pv. campestris HR induction in nonhost pepper plants. Bacterial cells from a culture of X. campestris pv. campestris wild-type strain 8004 grown overnight were resuspended to an OD_600_ of 0.01 in 0.3% DMSO or each of the inducers (splitomicin, pentetic acid, S0693, brassinin, BML-281, and resveratrol) and inhibitors (A-3, thioctic acid, motesanib, 5-fluorocytosine, carmofur, WB 64, HMS3229O07, aristolochic acid, NP-009807, and imidocarb) dissolved in 0.3% DMSO and infiltrated into the leaf mesophyll tissue of pepper leaves (*Capsicum annuum* cv. ECW-10R) with a blunt-end plastic syringe. The concentrations of the compounds used are indicated after their names. The Δ*hrcV* mutant was used as a T3SS deficiency control. (A) HR symptoms in the inoculated leaves. Photographs were taken 8 h after infiltration. (B) Electrolyte leakage from the inoculated leaves. The experiment was repeated three times. The results presented are from a representative experiment, and similar results were obtained in all other independent experiments.

Infiltration of X. campestris pv. campestris strain 8004 (suspended in 0.3% DMSO) into nonhost pepper leaves triggered visible HR symptoms at 8 h postinoculation, as shown in Fig. 4A. However, an Δ*hrpV* mutant strain deficient in the T3SS could not trigger HR symptoms (Fig. 4A). Under the tested conditions, the 8004 strain treated with each of the inducer molecules (splitomicin, pentetic acid, S0693, brassinin, BML-281, and resveratrol) triggered HR symptoms (Fig. 4A). Notably, the HR symptoms in the presence of splitomicin or pentetic acid appeared more serious than those caused by the wild type and the wild type treated with other inducer molecules (S0693, brassinin, BML-281, and resveratrol) (Fig. 4A). In contrast, for the wild type exposed to the inhibitor molecules A-3, thioctic acid, 5-fluorocytosine, carmofur, WB 64, HMS3229O07, and imidocarb, there was a definite decrease in HR symptoms compared to the control (Fig. 4A) at 8 h postinoculation. However, the wild type exposed to the inhibitor molecules motesanib, aristolochic acid, and NP-009807 showed no difference compared to the wild type (Fig. 4A). This suggested that the inhibitors A-3, thioctic acid, 5-fluorocytosine, carmofur, WB 64, HMS3229O07, and imidocarb influenced the T3SS function of X. campestris pv. campestris *in planta* but that the inhibitors motesanib, aristolochic acid, and NP-009807 did not.

To gain a more quantitative measure of the influence of the inducer and inhibitor molecules on X. campestris pv. campestris T3SS *in planta* function, we used an electrolyte leakage assay. For this, four 0.4-cm^2^ disks were collected from the area infiltrated with bacteria and incubated in 5 ml of distilled water, and conductivity was measured. Consistent with the observations described above, the pepper leaf tissues infiltrated with strain 8004 exposed to the inducer molecule splitomicin or pentetic acid had a significantly (*P < *0.05 by a *t* test) higher electrolyte leakage score than the control (DMSO) at 8 h and 16 h postinoculation, although no significant difference was detected at 24 h postinoculation (Fig. 4B). All other inducer small molecules (S0693, brassinin, BML-281, and resveratrol) caused electrolyte leakage similar to that of the control at the tested time points (Fig. 4B). In contrast, treatments with the inhibitor molecule carmofur or WB 64 showed significantly (*P < *0.01 or 0.05 by a *t* test) lower electrolyte leakage values at all tested time points than that of the control (Fig. 4B). Additionally, exposure of the wild type to the inhibitors A-3, 5-fluorocytosine, HMS3229O07, and imidocarb led to significantly (*P < *0.01 or 0.05 by a *t* test) lower electrolyte leakage values at 8 h and 16 h postinoculation than for the control (Fig. 4B), while thioctic acid exposure led to a significantly (*P < *0.05 by *t* test) lower electrolyte leakage value at only 8 h postinoculation than for the control (Fig. 4B). However, exposure to motesanib, aristolochic acid, and NP-009807 led to electrolyte leakage values at all tested time points that were similar to those of the control (Fig. 4B). Taken together, two inducer molecules (splitomicin and pentetic acid) and seven inhibitor molecules (carmofur, WB 64, A-3, 5-fluorocytosine, HMS3229O07, imidocarb, and thioctic acid) demonstrate a significant influence on X. campestris pv. campestris T3SS function in nonhost plants.

### Small molecules influence the virulence of X. campestris pv. campestris on the host plant Chinese radish.

**FIG 5:**
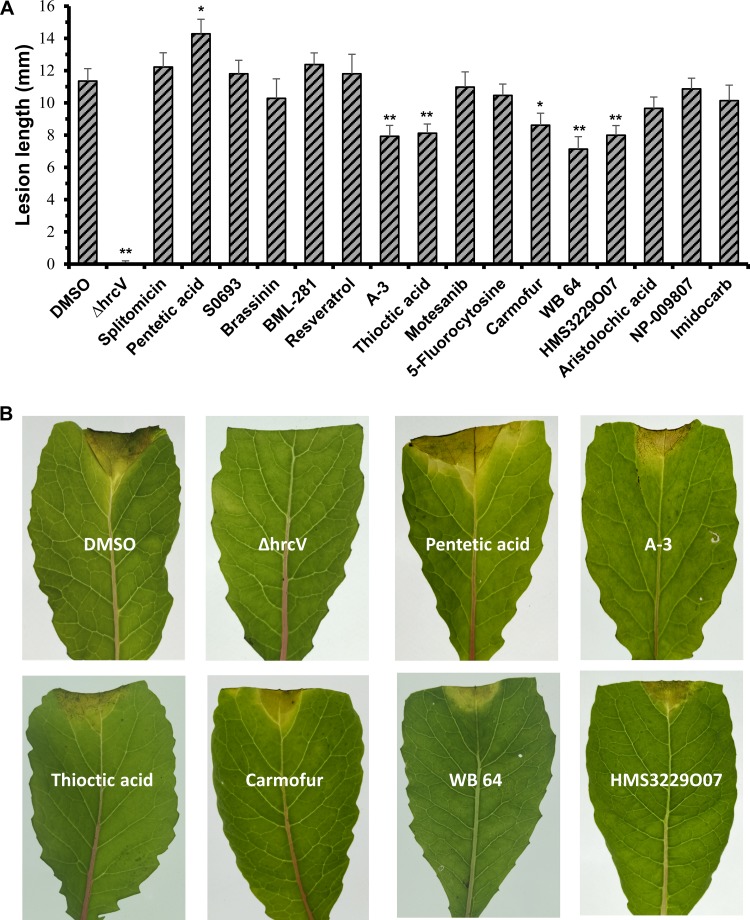
Effects of selected compounds on the virulence of X. campestris pv. campestris strain 8004 in the host plant Chinese radish. Bacterial cells of strain 8004 were suspended in 0.3% DMSO containing an inducer or inhibitor compound at the concentration of its optimal dosage and adjusted to an optical density at 600 nm of 0.001. Leaves on 5-week-old seedlings were cut with scissors dipped in the bacterial suspension. At least 36 leaves were inoculated for each treatment. (A) Lesion length was measured 10 days after inoculation, and data were analyzed by Student’s *t* test, compared with the control (DMSO treatment) (*, *P < *0.05; **, *P < *0.01). (B) Disease symptom pictures taken at 10 days postinoculation. The experiment was repeated three times independently, and similar results were obtained. ΔhrcV, X. campestris pv. campestris T3SS-deficient mutant strain.

A functional T3SS is essential for the full virulence of X. campestris pv. campestris in host plants (27). We further examined whether the identified T3SS inducer and inhibitor small molecules influence the virulence of X. campestris pv. campestris in host plants. To do this, bacterial cells of the X. campestris pv. campestris wild-type strain 8004 were suspended in 0.3% DMSO containing each of the target molecules using the optimal concentrations defined above. The bacterial cell suspension was adjusted to an optical density at 600 nm of 0.001 and inoculated into the leaves of the host plant Chinese radish (Raphanus sativus) by a leaf-clipping method. At 10 days postinoculation, the lengths of lesions caused by X. campestris pv. campestris treated with or without the target molecule were assessed. Of the molecules shown to induce the T3SS, X. campestris pv. campestris treated with pentetic acid produced significantly (*P < *0.05 by a *t* test) longer lesions than the control (Fig. 5). X. campestris pv. campestris treated with the other inducer molecules showed lesion lengths that were very similar to those of the control (Fig. 5). In contrast, treatment of X. campestris pv. campestris with the inhibitor molecules A-3, carmofur, HMS3229O07, thioctic acid, and WB 64 significantly (*P < *0.01 or 0.05 by a *t* test) reduced the lesion length produced compared to the control. However, the other inhibitor molecules did not significantly influence lesion development by X. campestris pv. campestris (Fig. 5). These data indicate that an inducer molecule (pentetic acid) and five inhibitor molecules (A-3, carmofur, HMS3229O07, thioctic acid, and WB 64) demonstrate a significant influence on T3SS function during X. campestris pv. campestris infection of a host plant. Compounds dissolved in DMSO at concentrations three times higher than their optimal doses were infiltrated into the leaves of Chinese radish. Ten days after infiltration, no damage was seen on the infiltrated leaves, suggesting that the compounds do not harm the plant under the tested conditions.

## DISCUSSION

Here, we report a set of small molecules that are able to suppress the disease symptoms of X. campestris pv. campestris by influencing the function of the T3SS when infecting the host plant Chinese radish (Table 2). Additionally, we identified a small molecule that could potentiate disease symptoms of X. campestris pv. campestris in the same fashion (Table 2).

**TABLE 2:** Effects of identified inducer and inhibitor compounds^*a*^

Compound	Effect on:															
	Operons in *hrp* cluster			*hrp* regulatory genes								T2SS gene *gspD*	T4SS genes		Plant test	
	*hrpA*	*hrpC*	*hrpE*	*hrpX*	*hrpG*	*hpaS*	*hpaR1*	*rsmA*	*zur*	*colS*	*colR*		*virB2*	*virB9*	HR	Virulence
Splitomicin	+**	+**	+**	+**	+*	\	\	\	\	\	+**	\	\	\	+	\
Pentetic acid	+**	+**	+**	+**	+**	\	\	\	\	\	+**	\	\	\	+	+*
S0693	+**	+**	+**	+**	+*	\	\	\	\	\	+*	\	\	\	\	\
Brassinin	+*	+*	+**	+*	+*	\	\	\	\	\	+**	\	\	\	\	\
BML-281	+**	+*	+**	+**	+*	\	\	\	\	\	+*	\	\	\	\	\
Resveratrol	+*	+*	+*	+*	+*	\	\	\	\	\	+*	\	\	\	\	\
A-3	−**	−**	−**	−**	−*	\	\	\	\	\	−**	\	\	\	−	−**
Thioctic acid	−**	−**	−**	−**	−*	\	\	\	\	\	−**	\	\	\	−	−**
Motesanib	−**	−**	−**	−**	−**	\	\	\	\	\	−**	\	\	\	\	\
5-Fluorocytosine	−**	−**	−**	−**	−*	\	\	\	\	\	−*	\	\	\	−	\
Carmofur	−**	−**	−**	−**	−*	\	\	\	\	\	−**	\	\	\	−	−*
WB 64	−**	−**	−**	−**	−**	\	\	\	\	\	−**	\	\	\	−	−**
HMS3229O07	−**	−**	−**	−**	−**	\	\	\	\	\	−**	\	\	\	−	−**
Aristolochic acid	−**	−**	−**	−*	−*	\	\	\	\	\	−*	\	\	\	\	\
NP-009807	−**	−**	−**	−**	−**	\	\	\	\	\	−*	\	\	\	\	\
Imidocarb	−**	−**	−**	−**	−**	\	\	\	\	\	−**	\	\	\	−	\

a Asterisks indicate statistically significant differences, compared with the control (DMSO) (by Student’s *t* test) (*, *P *< 0.05; **, *P *< 0.01). +, positive effect; −, negative effect; \, no significant effect.

Several high‐throughput screening approaches have been used to identify T3SS inhibitors from chemical libraries (3, 4, 17–20, 45). Screens of a large number of compounds have been deployed mainly in animal and human bacterial pathogen tests, but screens looking at plant-pathogenic bacteria have focused mainly on smaller chemical sets (3, 4, 17–20, 45). In the present study, the X. campestris pv. campestris strain 8004/pXopNlux, containing a bioreporter plasmid with *luxAB* genes transcriptionally fused to the *xopN* promoter, was constructed for screening. The *xopN* gene was selected because it encodes an effector protein known for its importance in X. campestris pv. campestris virulence (25, 27). A total of 13,126 different small-molecule compounds in a library were tested to identify agents that influence T3SS expression in X. campestris pv. campestris. After two rounds of screening, 35 compounds showed significant effects on *xpoN* promoter activity. This efficiency was equivalent to those of other large libraries containing thousands of small molecules (5–7). Finally, after a set of assays, six of the compounds demonstrated suppression of the HR of X. campestris pv. campestris in pepper, five of which showed an inhibitory influence on virulence symptom development by X. campestris pv. campestris on the host plant Chinese radish.

If such agents were to be used in agriculture, it would be important to gain a greater understanding of the functional mechanism behind how they influence the T3SS of X. campestris pv. campestris. Like most *Xanthomonas* species, the T3SS apparatus of X. campestris pv. campestris is composed of more than 20 proteins that are encoded by the *hrp* gene cluster mainly consisting of six operons (*hrpA* to *hrpF*) (21, 40). Focusing on the action of the 5 compounds that showed an inhibitory influence on X. campestris pv. campestris virulence, our qRT-PCR data revealed that all compounds had an influence on the three selected representative *hrp* genes, i.e., *hrcC*, *hrcU*, and *hpaB*. Given that these three genes are within the *hrpA*, *hrpC*, and *hrpE* operons, the results indicate that the expression of these operons is influenced. As the HrcC and HrcU proteins are the main components of the T3SS and HpaB is a chaperone controlling the translocation of effectors (46), the results suggest that these agents may influence the expression of structural elements of the T3SS and the translocation of effector proteins. In addition, these agents also affect the transcription of the effector genes *xopN* and *xopAH* (Fig. 3). The promoters of *xopN* and *xopAH* as well as the *hrpA*, *hrpC*, and *hrpE* operons contain so-called PIP boxes. It has been demonstrated that in *Xanthomonas* species, the AraC-type transcriptional regulator HrpX directly activates its regulon genes by binding to their PIP boxes (26, 47, 48). It is likely that because of the scope of the influence of these agents, the expression of the *hrp* operons and the effector genes is probably affected via an influence on *hrpX*. This is supported by further qRT-PCR data that showed that these agents did not influence the expression of the other main *hrp* gene regulators *hpaR1*, *zur*, *rsmA*, *hpaS*, and *colS* but not *hrpG* and *colR* (Fig. 3). Given that HpaS and ColS are membrane-bound sensor histidine kinases that influence the expression of *hrp* genes (23, 40, 41) and that HpaS not only activates HrpG via phosphorylation but also positively regulates the transcription of *hrpG* (23), it is possible that these agents influence the expression of *hrp* genes via an interaction with HpaS or ColS. However, it is also possible that the compounds affect the expression of *hrpG* and *colR* via an unknown regulator(s) rather than HpaS and ColS. Verification of these possibilities will be a worthy subject for further studies.

In addition to the screening of the large small-molecule library, the compounds imidocarb, 4,4′-thiobis(2-methylphenol), and benzoic acid were examined to determine their potential influence on the X. campestris pv. campestris T3SS using the bioreporter system. These compounds were selected as they have previously been shown to have strong inhibitory effects on the T3SS in Y. pestis or E. amylovora (17, 38, 39). Interestingly, only imidocarb but not 4,4′-thiobis(2-methylphenol) or benzoic acid showed a significant influence on the X. campestris pv. campestris T3SS, indicating that a T3SS inhibitor agent in one bacterium may not be effective on a T3SS from another bacterium. Although the T3SSs are structurally conserved in most bacteria, the reasons why these compounds are not effective against X. campestris pv. campestris are manyfold, including differences in the mechanisms of regulation and the permeation of the agent.

Notably, among the identified compounds, only 7 agents significantly suppressed X. campestris pv. campestris HR induction. These data indicate that conditions play a major role in the function and potency of the compounds. Given that plant tissue is an environment that is very different from the conditions used in the screening assay, it is not surprising that some compounds do not confer a significant effect inside plant tissues. This observation is reinforced by tests in various media that influenced not only the potency of some compounds on the T3SS but also the growth of bacterial cells (Fig. 1; see also Table S1 in the supplemental material). The fact that some compounds in various minimal media exhibited different effects on bacterial growth may suggest that the compounds probably influence the availability of some nutrients. However, the mechanisms by which the compounds accomplish their functions need to be further investigated, although some of them have been applied as antitumor agents (splitomicin, BML-281, resveratrol, A-3, motesanib, and carmofur), antifungal (5-fluorocytosine) or antimycobacterial (WB 64) drugs, antioxidants (thioctic acid and imidocarb), a chelating titrant (pentetic acid), and kinase inhibitors (HMS3229O07 and motesanib) (Table S5). In addition, leaf-clipping assays revealed that treatments with just five of the compounds identified (A-3, carmofur, HMS3229O07, thioctic acid, and WB 64) had a significant effect on virulence (Fig. 5). Although 5-fluorocytosine and imidocarb showed a significant influence on HR induction (Fig. 4), they did not show a significant effect on virulence (Fig. 5). One possibility may be that the effect of these compounds on the T3SS did not last long enough after the bacterial cells were inoculated into plant leaves with the leaf-clipping method in the virulence assay. As detailed below, unlike the infiltration method used in the HR assay, which infiltrated not only bacterial cells but also the compound suspension into plant tissues, the leaf-clipping method with scissors introduced only bacterial cells into plant leaves. Of course, more work needs to be performed in the future to fully elucidate the exact cause.

Although the main objective of this study was to identify small molecules that inhibit the T3SS in order to suppress X. campestris pv. campestris disease, it must be noted that several agents were recognized to do the opposite. Of the 16 compounds that showed consistent modulation of *xpoN* promoter activity in various growth media, a total of 6 had the ability to induce promoter activity. Even though these compounds were active during the *in vitro* assays, only two of the molecules (splitomicin and pentetic acid) demonstrated HR induction of X. campestris pv. campestris in pepper, and just one (pentetic acid) contributed to a significant increase in disease symptoms in Chinese radish.

In summary, we have shown the inhibitory effects of the compounds A-3, carmofur, HMS3229O07, thioctic acid, and WB 64 on the T3SS of X. campestris pv. campestris both *in vitro* and *in planta*. In addition, we identified two molecules (splitomicin and pentetic acid) that could potentiate the X. campestris pv. campestris T3SS both *in vitro* and *in planta*. All of the compounds identified in the screen that were active against X. campestris pv. campestris have never been identified previously and do not resemble the phenolic compounds and their derivatives described in other studies (3, 4, 17–20, 45). All of the agents identified in this work except resveratrol are nonphenolic and belong to the ester (splitomicin), carboxylic acid (pentetic acid and thioctic acid), amide (BML-281, S0693, motesanib, and WB 64), indole (brassinin), sulfonamide (A-3), pyrimidine (5-fluorocytosine and carmofur), pyridine (HMS3229O07), phenanthrene (aristolochic acid), heterocycle (NP-009807), and imidazole (imidocarb) groups (Fig. 2). These findings are important because they may provide not only potential tool compounds for the further understanding of virulence regulation in X. campestris pv. campestris but also chemical starting points for the generation of antivirulence drugs, which might be employed in the prevention or treatment of X. campestris pv. campestris infection in the future.

## MATERIALS AND METHODS

### Bacterial strains, plasmids, and growth conditions.

The bacterial strains and plasmids used in this study are listed in Table 1. X. campestris pv. campestris strains were grown at 28°С in NYG medium (5 g of peptone, 3 g of yeast extract, and 20 g of glycerol per liter [pH 7.0]) (28), XVM2 minimal medium [3.42 g of sucrose, 1.8 g of fructose, 0.3 g of Casamino Acids, 1.32 g of (NH_4_)_2_SO_4_, 0.6 g of MgSO_4_, 0.06 g of K_2_HPO_4_, 0.022 g of KH_2_PO_4_, 1.17 g of NaCl, 0.11 g of CaCl_2_, 0.00278 g of FeSO_4_ (pH 6.7)] (49), XCM1 minimal medium [1.0 g of (NH_4_)_2_SO_4_, 10.5 g of K_2_HPO_4_, 4.5 g of KH_2_PO_4_, 0.246 g of MgSO_4_, 2.362 g of succinic acid, and 0.15 g of Casamino Acids per liter (pH 6.6)], or XZM minimal medium [1.0 g of (NH_4_)_2_SO_4_, 1 g of K_2_HPO_4_, 0.5 g of KH_2_PO_4_, 0.01 g of MgSO_4_, 1.2 g of NaCl, 5 g of fructose, and 0.3 g of Casamino Acids per liter (pH 6.0)]. Escherichia coli strains were grown in L medium (50). The following antibiotics were added at the indicated final concentrations: ampicillin at 100 μg/ml, kanamycin at 25 μg/ml, rifampin at 50 μg/ml, spectinomycin at 50 μg/ml, and tetracycline at 5 μg/ml for X. campestris pv. campestris and 15 μg/ml for E. coli.

### Sources of the screened compounds.

The 17,045-small-molecule library used in this work was purchased from the National Compound Resource Center (Shanghai, China) (http://www.chemicallibrary.org.cn/). The follow-up compounds used after the primary screen were purchased from several companies (listed in Table S5 in the supplemental material). All of the compounds were maintained in DMSO (dimethyl sulfoxide).

### Construction of the bioreporter strain 8004/pXopNlux.

To screen for compounds that modulate the T3SS of X. campestris pv. campestris, a reporter strain named 8004/pXopNlux (Table 1) was constructed. A 2,139-bp DNA fragment containing the *luxAB* luciferase genes was amplified by PCR using plasmid pUTgfpluxAB (Table 1) DNA isolated from the E. coli strain FJAT-333 (Table 1) as the template and the primer set luxAB-F/luxAB-R (Table 3). Using the BamHI/HindIII sites, the fragment was cloned into vector pLAFR6 (Table 1), generating the recombinant plasmid pLux. Simultaneously, A 647-bp DNA fragment containing the promoter and the signal sequence of the gene *xopN* (*XC_0241*) encoding the effector protein XopN (from 488 bp upstream to 159 bp downstream of the start codon of the open reading frame [ORF] *XC_0241*) was amplified by PCR using the total DNA of X. campestris pv. campestris strain 8004 as the template and the primer set 0241-F/0241-R (Table 3). The resulting fragment was cloned into pLux using the EcoRI/BamHI sites, generating the recombinant plasmid pXopNlux (Table 1). All fragments were confirmed by sequencing (Tables S6 and S7). Finally, the plasmid pXopNlux was introduced into the X. campestris pv. campestris strain 8004 by conjugation as previously described (28), generating the reporter strain 8004/pXopNlux (Table 1).

**TABLE 3:** Primers used in this study^*a*^

Primer	Nucleotide sequence (5′→3′)	Amplified segment and description
luxAB-F	AAAGGATCCACGCCAGAAATGGCTTAGGTCTTA	2,139-bp fragment of the *luxAB* gene; used for construction of plasmid pUT*gfpluxAB*
luxAB-R	GGGAAGCTTTTACGAGTGGTATTTGACGATG	

0241-F	GGGGAATTCCGCTGGTCACGCCGTGCATGGG	647-bp fragment downstream of the *XC_0241* (*xopN*) start codon; used for construction of plasmid pXopNlux
0241-R	GGGGGATCCGGCATCGAATGGCTGGGCGAG	

0241-F2	AGCCGCATCCACGAAACGGA	126-bp DNA fragment of the *XC_0241* (*xopN*) gene; used for qRT-PCR
0241-R2	AACAGCGCGGTGCGTCGTAA	

16S-F	GAGGAAGGTGGGGATGACGTCA	108-bp DNA fragment of the 16S rRNA gene; used for qRT-PCR
16S-R	GATTGGCTTACCCTCGCGGG	

2004-F	TTGAGGCGGCCATATCACTC	119-bp DNA fragment of the *XC_2004* (*xopAH*) gene; used for qRT-PCR
2004-R	CCACACTGCCGATACACCTT	

hrcC-F	CGAAGTGCAGGTGTTTCAGC	122-bp DNA fragment of the *hrcC* gene; used for qRT-PCR
hrcC-R	CACGCACGCCGTATATGTTG	

hrcU-F	ACTGGAACTGCGTGCCTATG	120-bp DNA fragment of the *hrcU* gene; used for qRT-PCR
hrcU-R	GTTTCGAACAGCGATTCGGG	

hpaB-F	GCTGGAAGCGAATTTGTCGG	94-bp DNA fragment of the *hpaB* gene; used for qRT-PCR
hpaB-R	CAGTGGAACACGTACGACCA	

hrpG-F	TGCGTGGCATCGGACGACAG	91-bp DNA fragment of the *hrpG* gene; used for qRT-PCR
hrpG-R	CACTCGAAACGGCCCAGCAC	

hrpX-F	CGAAGTCGCATTGCTGGGCG	92-bp DNA fragment of the *hrpX* gene; used for qRT-PCR
hrpX-R	GCCTTGGACGCCTGCCGATA	

hpaR1-F	CGCACAGAAATTGCGTGGAA	101-bp DNA fragment of the *hpaR1* gene; used for qRT-PCR
hpaR1-R	GATCGTCGATGGTCAGTCCC	

zur-F	CGAATCGGTCAATGCCTTCG	129-bp DNA fragment of the *zur* gene; used for qRT-PCR
zur-R	TCCAGTTGCGAGACCACATC	

colR-F	GTTGCAGGAAGTGGAAGTGC	107-bp DNA fragment of the *colR* gene; used for qRT-PCR
colR-R	CCAGCGTGTCCAGGTTGTAT	

hpaS-F	ACTGATGCTGGACACCATGT	96-bp DNA fragment of the *hpaS* gene; used for qRT-PCR
hpaS-R	TTGTTGGAAAACCCGATGCG	

rsmA-F	TTGACGCGCCTAAGGATGTT	104-bp DNA fragment of the *rsmA* gene; used for qRT-PCR
rsmA-R	ACAATCGTCGTTGTGATGCG	

colS-F	CTACGCGATGGCATTCACAC	129-bp DNA fragment of the *colS* gene; used for qRT-PCR
colS-R	CTTGAGCGTCTGGGTCATGT	

1633-F	ACCAAGTGCGTACTGGTCTG	97-bp DNA fragment of the *XC_1633* (*virB9*) gene; used for qRT-PCR
1633-R	TCCCAGCCACCAGTAAAACC	

1637-F	GCGGTAGTGATTGCCGGATA	114-bp DNA fragment of the *XC_1637* (*virB2*) gene; used for qRT-PCR
1637-R	CATGTTGGCAATCTGAGCGG	

3563-F	CATGCTTCAGCTACCGCCTA	112-bp DNA fragment of the *XC_3563* (*gspD*) gene; used for qRT-PCR
3563-R	GCGCACAAGTAGTGTGTTGG	

a The underlined sequences indicate the restriction sites for BamHI, HindIII, EcoRI, and BamHI, respectively (top to bottom).

### Luciferase activity assay and screening of compounds.

The reporter strain 8004/pXopNlux was grown overnight in NYG medium. Bacterial cells were collected and suspended in the selected medium to an optical density at 600 nm of 0.05. Next, 100-μl bacterial suspensions supplemented with certain amounts of a compound dissolved in DMSO were cultivated in 96-well plates at 28°С with shaking (600 rpm) for 16 h. To determine the relative luciferase (Lux) activity, the optical density of bacterial cells at 600 nm was determined by using the Synergy H1 hybrid multimode reader (BioTek, USA), and the bacterial cells were then transferred to white immunoplates, where the Lux value was immediately determined by using the Synergy H1 hybrid multimode reader (BioTek, USA), with 0.5 μl 2% decanal added as the substrate. The relative Lux activity was obtained by dividing the Lux value with the bacterial density (OD_600_).

### RNA manipulation and gene expression analysis.

X. campestris pv. campestris was grown in NYG medium overnight at 28°C. Bacterial cells were collected, suspended to an optical density at 600 nm of 0.05 in the tested medium supplemented if necessary with small molecules of interest at their optimal dosages, and cultured for 16 or 20 h. Next, the total RNA was extracted from the culture with a total RNA extraction kit (Promega, Beijing, China), and reverse transcription was performed using a cDNA synthesis kit (TaKaRa, Dalian, China). Each kit was used according to the manufacturer’s instructions. To determine the transcription level of the genes tested, reverse transcription-quantitative PCR (qRT-PCR) was performed. SYBR green-labeled PCR fragments for the genes tested were amplified by using the corresponding primer sets listed in Table 3. The expression level of the 16S rRNA gene was used as an internal control for data analysis. All of the qRT-PCRs were performed in triplicate.

### Nonhost pepper plant hypersensitive response assay.

The HR of X. campestris pv. campestris was tested on nonhost pepper (*Capsicum annuum* cv. ECW-10R) plants. X. campestris pv. campestris strains were grown in NYG medium overnight at 28°C with shaking at 200 rpm. Bacterial cells were collected and suspended in 0.3% DMSO containing an inducer or inhibitor compound at the concentration of its optimal dosage. The bacterial cells in the suspension were adjusted to an optical density at 600 nm of 0.01. The pepper leaves were inoculated by infiltrating an ∼5-μl bacterial suspension into the abaxial leaf surface by using a blunt-end plastic syringe. The inoculated plants were maintained in a greenhouse with a 12-h day/night cycle with illumination by a fluorescent lamp and a constant temperature of 28°C, and HR symptoms were observed and photographed 8 h after inoculation. At least three plants were inoculated in each experiment, and the experiment was repeated three times. Electrolyte leakage was assayed by using a method described previously (23). Briefly, for each sample, four 0.4-cm^2^ disks were collected from the area infiltrated by bacteria and incubated in 5 ml of distilled water. Conductivity was measured with a DDS-307A conductometer (Shanghai Jingke Scientific Instrument Co., Ltd., China). Three samples were taken for each measurement in each experiment, and the experiment was repeated three times.

### Chinese radish leaf-clipping assay.

The virulence of X. campestris pv. campestris to Chinese radish (Raphanus sativus) was tested by the leaf-clipping method (51). X. campestris pv. campestris strains were grown in NYG medium overnight at 28°C with shaking at 200 rpm. Bacterial cells were collected and suspended in 0.3% DMSO containing an inducer or inhibitor compound at the concentration of its optimal dosage. The bacterial cells in the suspension were adjusted to an optical density at 600 nm of 0.001. Leaves on 5-week-old seedlings were cut with scissors dipped in the bacterial suspension. At least 36 leaves were inoculated for each treatment. Lesion length was measured 10 days after inoculation, and data were analyzed by a *t* test. The experiment was repeated three times independently.

## Supplementary Material

Supplemental file 1
