# Supplementary material for: Chemical Targeting and Manipulation of Type III Secretion in the Phytopathogen Xanthomonas campestris for Control of Disease
Source: Appl Environ Microbiol. 2020 Jan 21;86(3):e02349-19. doi: 10.1128/AEM.02349-19 (PMC6974632; doi:10.1128/AEM.02349-19)
Supplement: Supplemental file 1 [file zam003209570s1.pdf]

Zhou et al. Chemically targeting and manipulating type III secretion in phytopathogen *Xanthomonas campestris* for control of disease

Supplemental materials

1. **FIG S1** Luciferase activity produced by *Xcc* strains in the minimal medium XZM.
2. **FIG S2** Luciferase activity produced by strain 8004/pXopNlux in the nutrient rich medium NYG supplemented with inducer compounds.
3. **FIG S3** The defined optimal dosage of the identified inhibitor and inducer compounds.
4. **Table S1** The results from the first and second screen.
5. **Table S2** The effect of three known T3SS inhibitors on the lux activity of the reporter strain.
6. **Table S3** The result of the third screen.
7. **Table S4** The optimal dosage of the inhibitors and inducers.
8. **Table S5** The source of the compounds used in this work.
9. **Table S6** Sequences of the 2139-bp DNA fragment containing the luciferase genes *luxAB*.
10. **Table S7** Sequences of the 647-bp DNA fragment containing the promoter and signal sequence of xopN gene.

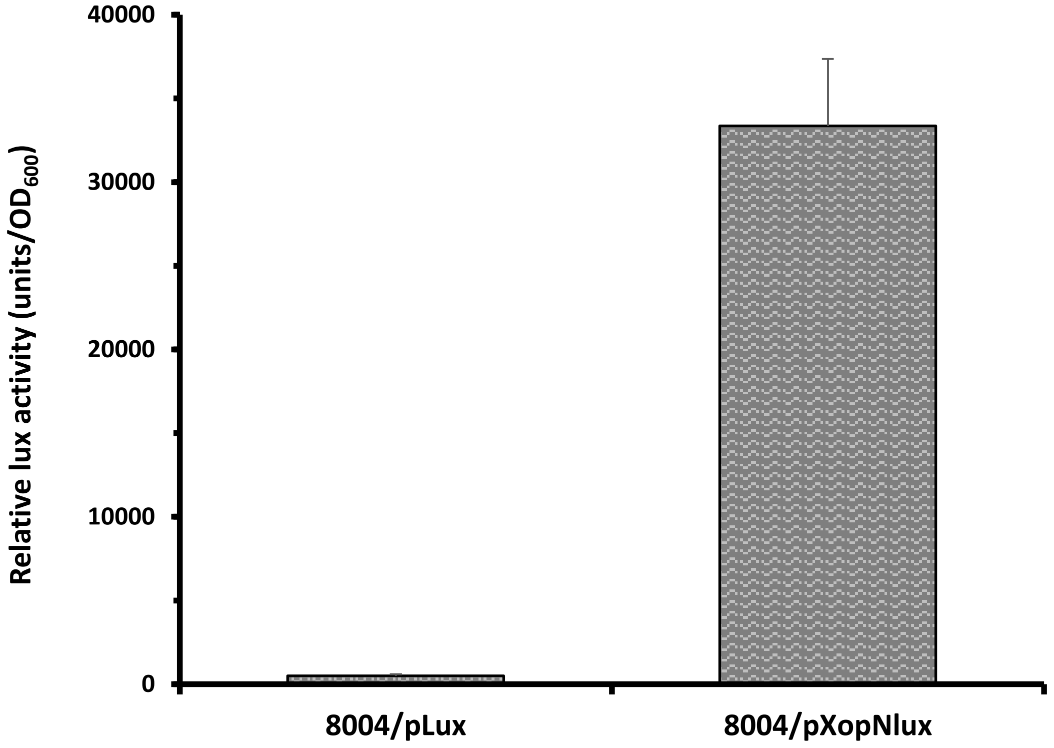

FIG S1 Luciferase activity produced by *Xcc* strains in the minimal medium XZM. The strains 8004/pLux and 8004/pXopNlux were grown in NYG overnight. Bacterial cells were collected and suspended to an optical density of 0.05 (600 nm) in the minimal medium XZM. The luciferase activity was assayed after incubation at 28 ^{o}C with shaking (600 rpm) for 16 h. Values given are the means and standard deviations of triplicate measurements. Data presented were from a representative experiment and similar results were obtained in two other independent experiments.

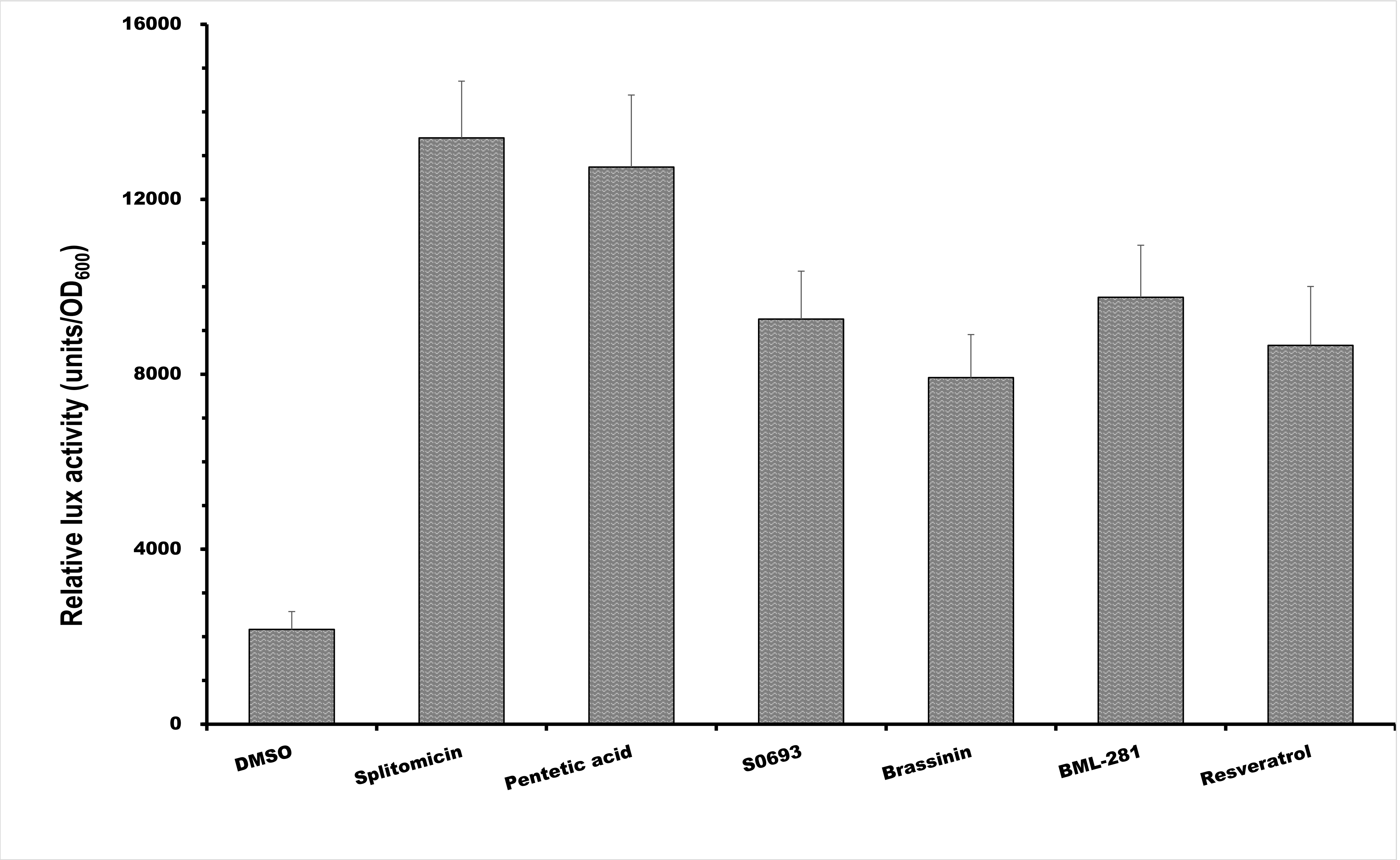

FIG S2 Luciferase activity produced by strain 8004/pXopNlux in the nutrient rich medium NYG supplemented with inducer compounds. The strain 8004/pXopNlux was grown in NYG medium overnight. Bacterial cells were collected and suspended to an optical density of 0.05 (600 nm) in NYG supplemented with each of the inducer compounds (splitomicin, pentetic acid, S0693, brassinin, BML-281 and resveratrol) at the concentration of its optimal dosage presented in Fig. S3. The luciferase activity was assayed after incubation at 28 ^{o}C with shaking (600 rpm) for 16 h. Values given are the means and standard deviations of triplicate measurements. Data presented were from a representative experiment and similar results were obtained in two other independent experiments.

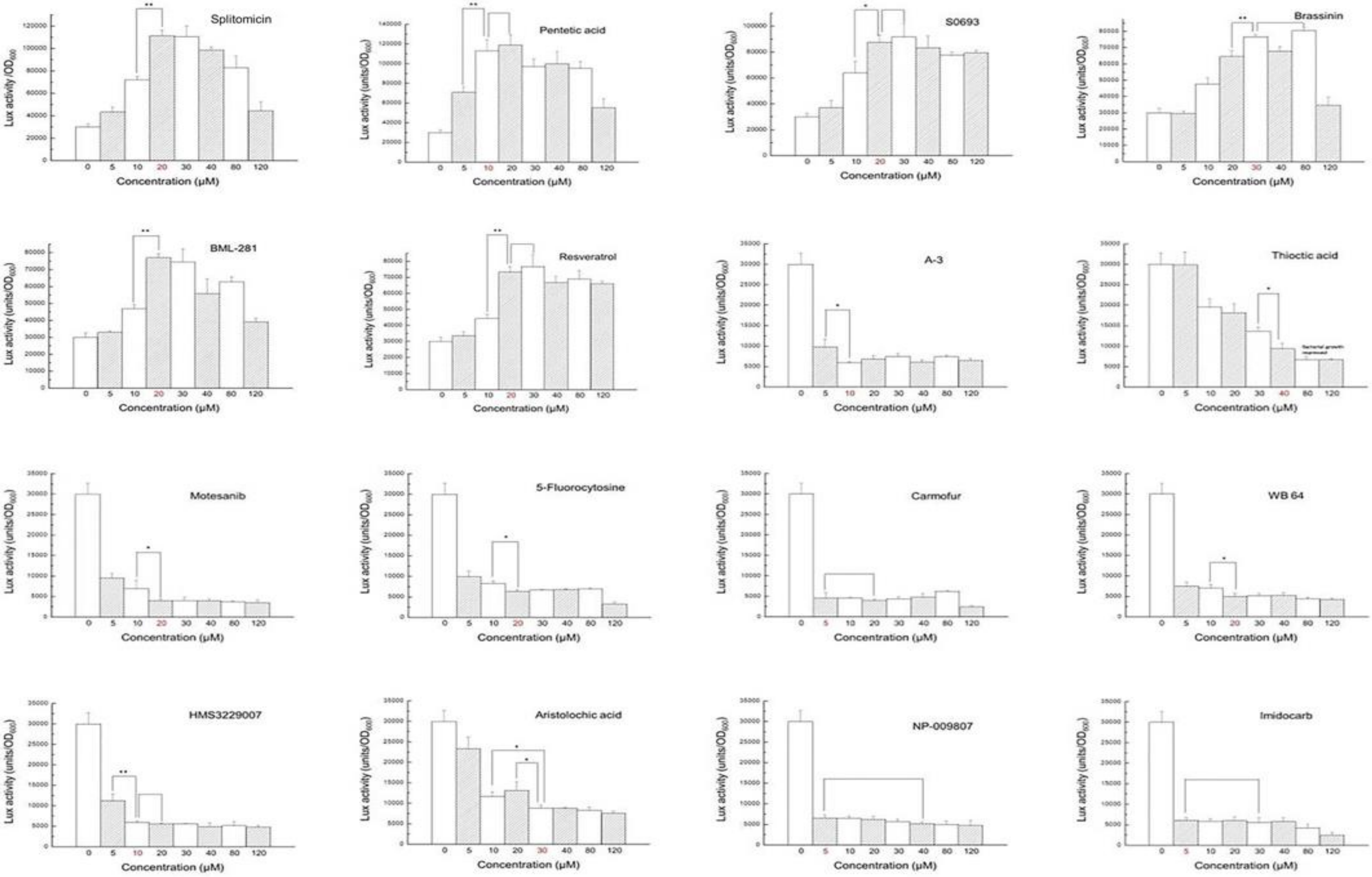

FIG S3 The defined optimal dosage of the identified inhibitor and inducer compounds. The strain 8004/pXopNlux was grown in the nutrient rich medium NYG overnight. Bacterial cells were collected and suspended to an OD_{600} of 0.05 in the minimal medium XZM supplemented with each of the inducer (splitomicin, pentetic acid, S0693, brassinin, BML-281 and resveratrol) or inhibitor (A-3, thioctic acid, motesanib, 5-fluorocytosine, carmofur, WB 64, HMS3229O07, aristolochic acid, NP-009807 and imidocarb) compounds with a concentration ranging from 5 to 120 μM and distributed into 96-well plates. The luciferase activity was assayed after incubation at 28 ^{o}C with shaking (600 rpm) for 16 h. Values given are the means and standard deviations of triplicate measurements. Data presented were from a representative experiment and similar results were obtained in two other independent experiments. *, *t*-test, *P*≤0.05; **, *t*-test, *P*≤0.01. The concentration in red indicates the optimal dosage defined.

Table S1 The results from the first and second screen#

Name	First screening (13126 compounds)			Second screening (180 compounds)		
	OD_{600}	Lux activity	Lux activity ratio (compound/DMSO)	OD_{600}	Lux activity	Lux activity ratio (compound/DMSO)
DMSO	0.112 ±0.006	35715 ±6923	1.000	0.116 ±0.008	32679 ±5842	1.000
(-)-[3R,4S]-Chromanol 293B	0.099 ±0.002	33787 ±1002	0.946			
(-)-3,4-Dihydroxynorephedrine	0.117 ±0.011	27928 ±813	0.782			
(-)-Bicuculline methiodide	0.108 ±0.001	32952 ±1269	0.923			
(-)-Bicuculline methobromide	0.106 ±0.004	31170 ±1752	0.873			
(-)-Bicuculline methochloride	0.109 ±0.004	35342 ±2661	0.990			
(-)-Bicuculline methydrobromideomide, 1(S), 9(R)	0.098 ±0.003	39150 ±2190	1.096			
(-)-cis-(1S,2R)-U-50488 Tartrate	0.112 ±0.009	30638 ±4233	0.858			
(-)-Cotinine	0.107 ±0.004	49194 ±4923	1.377			
(-)-Cyanopindolol hemifumarate	0.112 ±0.009	32879 ±817	0.921			
(-)-Epiafzelechin	0.114 ±0.010	31809 ±2156	0.891			
(–)-Epigallocatechin Gallate	0.104 ±0.006	53032 ±6661	1.485			
(-)-Epinephrine bitartrate	0.100 ±0.002	37522 ±3513	1.051			
(-)-Eseroline fumarate	0.102 ±0.003	35529 ±3376	0.995			
(-)-Eseroline fumarate salt	0.126 ±0.003	57076 ±4021	1.558			
(-)-HA-966	0.105 ±0.008	38833 ±6156	1.087			
(-)-Huperzine A	0.103 ±0.009	36915 ±3943	1.034			
(-)-Isoproterenol hydrochloride	0.103 ±0.002	28631 ±2623	0.802			
(-)-Levobunolol hydrochloride	0.123 ±0.009	61468 ±2727	1.678			
(-)-Maackiain	0.120 ±0.006	33008 ±1371	0.924			
(-)-MK 801 Maleate	0.110 ±0.006	33650 ±2771	0.942			
(-)-N6-(2-Phenylisopropyl)-Adenosine	0.101 ±0.007	54195 ±9042	1.517			
(-)-Naproxen Sodium	0.094 ±0.008	36665 ±892	1.027			
(-)-Nicotine	0.102 ±0.007	25136 ±754	0.704			
(-)-N-Methylsedridine	0.125 ±0.010	42663 ±4076	1.195			
(-)-Norcodeine	0.106 ±0.006	36898 ±212	1.033			

(-)-N-Phenylcarbamoyleseroline	0.102 ± 0.006	19850 ± 1182	0.556			
(-)-Perillic acid	0.111 ± 0.007	46893 ± 5806	1.313			
(-)-Physostigmine	0.113 ± 0.010	37158 ± 3815	1.040			
(-)-Quinpirole hydrochloride	0.111 ± 0.010	32363 ± 2311	0.906			
(-)-Scopolamine hydrobromide	0.112 ± 0.004	35249 ± 6052	0.987			
(-)-Scopolamine methyl bromide	0.103 ± 0.005	29951 ± 7430	0.839			
(-)-Scopolamine methyl nitrate	0.094 ± 0.001 *					
(-)-Scopolamine,n-Butyl-bromide	0.101 ± 0.005	26495 ± 6909	0.742			
(-)-Sulpiride	0.101 ± 0.008	26292 ± 5304	0.736			
(-)-Terreic acid	0.105 ± 0.005	28544 ± 2996	0.799			
(-)-Tetramisole Hydrochloride	0.101 ± 0.009	39758 ± 934	1.113			
(-)-trans-(1S,2S)-U-50488 Hydrochloride	0.111 ± 0.005	29648 ± 2305	0.830			
(-)-α-Methylnorepinephrine	0.099 ± 0.007	36545 ± 3425	1.023			
(+)-3-Hydroxyh-N-Methylmorphinan D-Tartrate	0.123 ± 0.011	40798 ± 3316	1.142			
(+)-Afzelechin	0.110 ± 0.003	29322 ± 1062	0.821			
(+)-AJ 76 Hydrochloride	0.100 ± 0.002	29402 ± 1673	0.823			
(+)-Bicuculline	0.104 ± 0.003	23929 ± 277	0.670			
(+)-Bromocriptine methanesulfonate	0.072 ± 0.004 **					
(+)-Brompheniramine maleate	0.102 ± 0.001	33498 ± 2939	0.938			
(+)-Butaclamol	0.115 ± 0.008	37611 ± 2882	1.053			
(+)-Butaclamol hydrochloride	0.103 ± 0.002	32657 ± 2957	0.914			
(+)-Catechin Hydrate	0.099 ± 0.002	37603 ± 1246	1.053			
(+)-Chlorpheniramine maleate	0.105 ± 0.004	37713 ± 1871	1.056			
(+)-Conocarpan	0.133 ± 0.007	45300 ± 5923	1.268			
(+)-Conocarpan acetate	0.136 ± 0.001	28487 ± 1080	0.798			
(+)-Cyclazocine	0.106 ± 0.008	47638 ± 938	1.334			
(+)-HA-966	0.103 ± 0.003	23775 ± 4763	0.666			
(+)-Hydrastine	0.104 ± 0.006	40020 ± 1863	1.121			
(+)-Isoproterenol (+)-bitartrate salt	0.108 ± 0.002	48711 ± 6869	1.364			
(+)-Lariciresinol	0.108 ± 0.010	38206 ± 926	1.070			
(+)-Levobunolol hydrochloride	0.107 ± 0.004	54929 ± 2934	1.499			
(+)-MK 801 Maleate	0.108 ± 0.005	31154 ± 1550	0.872			

(+)-Muscarine chloride	0.100 ±0.007	27966 ±1545	0.783			
(+)-N6-(2-Phenylisopropyl)-adenosine	0.107 ±0.009	38720 ±3770	1.084			
(+)-Nicotine (+)-di-p-toluoyl tartrate	0.103 ±0.014	37318 ±4269	1.045			
(+)-Nicotine di-p-toluoyl-D-tartrate	0.116 ±0.007	27653 ±744	0.774			
(+)-N-Methylallosedridine	0.125 ±0.010	37247 ±1968	1.043			
(+)-PD 128907 hydrochloride	0.111 ±0.002	34955 ±1386	0.979			
(+)-Pilocarpine hydrochloride	0.106 ±0.004	46689 ±6517	1.307			
(+)-Pinoresinol	0.128 ±0.005	42315 ±2364	1.185			
(+)-Pinoresinol diacetate	0.125 ±0.010	30753 ±1147	0.861			
(+)-Pinoresinol-4-O-β-D-glucopyranoside	0.119 ±0.006	34211 ±1000	0.958			
(+)-Quisqualic acid	0.101 ±0.009	34378 ±2969	0.963			
(+)-SK&F 10047 hydrochloride	0.110 ±0.009	30581 ±2134	0.856			
(+)-S-Myricanol glucoside	0.116 ±0.004	33052 ±2716	0.925			
(+)-Tubocurarine chloride	0.109 ±0.003	39459 ±3144	1.105			
(+)-U-50488 Hydrochloride	0.107 ±0.000	30522 ±967	0.855			
(+)-UH 232 Maleate	0.106 ±0.009	32326 ±1068	0.905			
(+,-)-Octopamine Hydrochloride	0.104 ±0.002					
(+,-)-Synephrine	0.112 ±0.008	52916 ±5098	1.482			
(±)-1-(1,2-Diphenylethyl)-piperidine maleate	0.108 ±0.004	30746 ±1262	0.861			
(±)-13-Azaprostanoic acid	0.114 ±0.005	37441 ±4171	1.048			
(±)-13-HODE	0.111 ±0.002	34922 ±514	0.978			
(±)-1-Aminocyclopentane-cis-1,3-dicarboxylic acid	0.112 ±0.012	28560 ±3551	0.800			
(±)-1-Aminocyclopentane-trans-1,3-dicarboxylic acid	0.106 ±0.001	33610 ±1949	0.941			
(±)-2-(N-Phenylethyl-N-propyl)-amino-5-hydroxytetralin)	0.112 ±0.004	23281 ±2792	0.652			
(±)-2,3-Dichloro-α-methylbenzylamine hydrochloride	0.104 ±0.003	31860 ±2743	0.892			
(±)-2-Amino-3-phosphonopropionic acid	0.095 ±0.001	41388 ±695	1.159			
(±)-2-Amino-4-phosphonobutyric acid	0.100 ±0.003	39175 ±745	1.097			
(±)-2-Amino-5-phosphonopentanoic acid	0.097 ±0.004	39961 ±954	1.119			
(±)-2-Amino-7-phosphonoheptanoic acid	0.096 ±0.002	33955 ±4063	0.951			
(±)-3-(3,4-dihydroxyphenyl)-2-methyl-DL-alanine	0.099 ±0.001	42461 ±1878	1.189			
(±)-4-Amino-3-(5-chloro-2-thienyl)-butanoic acid	0.102 ±0.003	31247 ±2381	0.875			
(±)-4-Hydroxynon-2-enal	0.115 ±0.003	29438 ±792	0.824			

(±)-5'-Chloro-5'-deoxy-ENBA	0.116 ±0.002	37847 ±2403	1.060			
(±)-5-HETE	0.103 ±0.005	34021 ±1499	0.953			
(±)-5-HETE Lactone	0.103 ±0.003	28628 ±2114	0.802			
(±)-5-HETrE	0.115 ±0.002	29658 ±405	0.830			
(±)-6-Chloro-PB hydrobromide	0.116 ±0.008					
(±)-7-Hydroxy-DPAT hydrobromide	0.100 ±0.006	44213 ±2838	1.238			
(±)-8-Hydroxy-DPAT hydrobromide	0.098 ±0.004	51592 ±2548	1.426			
(±)-9-HODE	0.116 ±0.004	34057 ±2306	0.954			
(±)-AC 7954 Hydrochloride	0.113 ±0.004	33556 ±2838	0.940			
(±)-AMPA hydrobromide	0.102 ±0.004	38746 ±1158	1.085			
(±)-AMT hydrochloride	0.099 ±0.007	38794 ±754	1.086			
(±)-Anatoxin A fumarate	0.110 ±0.006	38122 ±2486	1.067			
(±)-Atenolol	0.101 ±0.014	49601 ±6431	1.389			
(±)-Baclofen	0.097 ±0.006	44542 ±4612	1.247			
(±)-Bay K 8644	0.108 ±0.004	60170 ±5829	1.663			
(±)-Bisoprolol hemifumarate	0.115 ±0.004	36955 ±4456	1.035			
(±)-Blebbistatin	0.107 ±0.009	27553 ±829	0.771			
(±)-Brompheniramine maleate	0.106 ±0.008	38935 ±2935	1.090			
(±)-Butaclamol hydrochloride	0.106 ±0.005	47578 ±5076	1.332			
(±)-CGP-12177A Hydrochloride	0.102 ±0.011	35684 ±3755	0.999			
(±)-Chloro-APB Hydrobromide	0.097 ±0.007	38432 ±866	1.076			
(±)-Chlorpheniramine maleate	0.104 ±0.004	42509 ±3769	1.190			
(±)-cis-Piperidine-2,3-Dicarboxylic acid	0.099 ±0.002	31501 ±638	0.882			
(±)-Cloprostenol sodium salt	0.111 ±0.007	31617 ±1723	0.885			
(±)-CPP	0.101 ±0.013	35875 ±951	1.004			
(±)-DOI hydrochloride	0.100 ±0.002	34516 ±1556	0.966			
(±)-Epibatidine	0.100 ±0.009	35352 ±4508	0.990			
(±)-Epinephrine hydrochloride	0.109 ±0.004	40980 ±1850	1.147			
(±)-HA-966	0.101 ±0.006	32108 ±3413	0.899			
(±)-Ibotenic acid	0.104 ±0.001	32471 ±1137	0.909			
(±)-Ibuprofen	0.099 ±0.002	40401 ±2654	1.131			
(±)-Isoproterenol hydrochloride	0.123 ±0.006	21795 ±3495	0.610			

(±)-McN 5652	0.118 ±0.003	26371 ±2702	0.738			
(±)-Methoxyverapamil hydrochloride	0.114 ±0.011	39616 ±2668	1.109			
(±)-Metoprolol (+)-tartrate	0.099 ±0.007	32975 ±1797	0.923			
(±)-Muscarine chloride	0.105 ±0.013	37167 ±2746	1.041			
(±)-N-Allylnormetazocine hydrochloride	0.107 ±0.005	44593 ±2411	1.249			
(±)-Nipecotic acid	0.104 ±0.002	46064 ±3047	1.290			
(±)-Norepinephrine (+)-bitartrate	0.095 ±0.002	43615 ±3437	1.221			
(±)-Normetanephrine hydrochloride	0.108 ±0.019	38339 ±2463	1.073			
(±)-Octoclothepin maleate	0.101 ±0.000	20126 ±249	0.556			
(±)-Octopamine hydrochloride	0.094 ±0.003	47423 ±5016	1.328			
(±)-Palmitoylcarnitine chloride	0.103 ±0.003	28809 ±1247	0.807			
(±)-p-Aminoglutethimide	0.108 ±0.015	41657 ±6435	1.166			
(±)-p-Chlorophenylalanine	0.105 ±0.006	39370 ±3255	1.102			
(±)-PD 128,907 Hydrochloride	0.104 ±0.015	35802 ±4546	1.002			
(±)-PPHT Hydrochloride	0.104 ±0.011	35041 ±3493	0.981			
(±)-Propranolol hydrochloride	0.102 ±0.008	21626 ±2267	0.598			
(±)-Quinpirole dihydrochloride	0.104 ±0.008	26934 ±1820	0.754			
(±)-SKF 38393, N-allyl-hydrobromide	0.105 ±0.004	46130 ±2266	1.292			
(±)-SKF-38393 Hydrochloride	0.098 ±0.005	31809 ±5221	0.891			
(±)-SKF-82958 Hydrobromide	0.116 ±0.002	33524 ±628	0.939			
(±)-Sotalol Hydrochloride	0.098 ±0.003	44458 ±1234	1.245			
(±)-Sulpiride	0.104 ±0.010	25243 ±5585	0.707			
(±)-Synephrine	0.103 ±0.012	54780 ±1289	1.514			
(±)-Taxifolin	0.111 ±0.007	39053 ±1674	1.093			
(±)-Thalidomide	0.105 ±0.004	33356 ±1687	0.934			
(±)-Threo-1-Phenyl-2-decanoylamino-3-morpholino-1-propanol hydrochloride	0.109 ±0.018	28549 ±4988	0.799			
(±)-Threo-3-methylglutamic acid	0.102 ±0.007	35123 ±3572	0.983			
(±)-trans-U-50488 methanesulfonate	0.103 ±0.005	29567 ±1061	0.828			
(±)-Tropanyl-2-(4-chlorophenoxy)-butanoate	0.083 ±0.006 **					
(±)-U-50488 hydrochloride	0.105 ±0.003	26216 ±1012	0.734			
(±)-Vanillylmandelic acid	0.102 ±0.003	39755 ±1274	1.113			
(±)-Verapamil hydrochloride	0.114 ±0.007	54210 ±1419	1.498			

(±)-Vesamicol hydrochloride	0.105 ±0.012	35887 ±1370	1.005			
(±)-α-Cyclopropyl-4-phosphonophenylglycine	0.102 ±0.004	69263 ±5312	1.852			
(±)-α-Lipoic Acid	0.104 ±0.010	24221 ±859	0.669			
(±)-α-Methyl-4-carboxyphenylglycine	0.100 ±0.006	33643 ±4321	0.942			
(±)-α-Methylserotonin maleate	0.105 ±0.007	34646 ±6874	0.970			
(±)-γ-Vinyl GABA	0.112 ±0.001	37461 ±860	1.049			
(1,2,5,6-Tetrahydropyridin-4-yl)-methylphosphinic acid	0.108 ±0.010	30961 ±1974	0.867			
(16R)-Dihydrositsirikine	0.120 ±0.003	34965 ±3699	0.979			
(1R,3R,4S)-1-Aminocyclopentane-1,3,4-tricarboxylic acid	0.103 ±0.008	26763 ±4097	0.749			
(1'S,2'S)-Nicotine-1'-oxide	0.109 ±0.008	22474 ±1931	0.629			
(1S,3R,4S)-1-Aminocyclopentane-1,2,4-tricarboxylic acid	0.112 ±0.007	33610 ±2047	0.941			
(1S,9R)-β-Hydrastine	0.104 ±0.009	32159 ±1254	0.900			
(2,4-Dihydroxyphenyl)-acetonitrile	0.117 ±0.004	43898 ±3569	1.229			
(24S)-Cycloartane-3,24,25-triol 24,25-acetonide	0.122 ±0.005	37505 ±3720	1.050			
(2R,4R)-4-Aminopyrrolidine-2,4-dicarboxylate	0.101 ±0.006	26798 ±551	0.750			
(2S)-α-Ethylglutamic acid	0.109 ±0.004	22326 ±1257	0.625			
(2S,1'S,2'R)-2-(Carboxycyclopropyl)-glycine	0.105 ±0.013	41190 ±3350	1.153			
(2S,1'S,2'S)-2-(Carboxycyclopropyl)-glycine	0.096 ±0.009	63611 ±3658	1.758			
(2S,2'R,3'R)-2-(2',3'-Dicarboxycyclopropyl)-glycine	0.104 ±0.003	28459 ±3214	0.797			
(2S,3R)-Chlorpheg	0.102 ±0.007	39189 ±5693	1.097			
(2S,3S)-(-)-Glucodistylin	0.111 ±0.005	38815 ±2726	1.087			
(2S,3S,4S)-2-Methyl-2-(carboxycyclopropyl)-glycine	0.099 ±0.013	34287 ±2288	0.960			
(2S,4S)-4-Methylglutamic acid	0.106 ±0.007	36187 ±2083	1.013			
(3R)-Hydrangenol 8-O-glucoside pentaacetate	0.117 ±0.001	46788 ±1755	1.310			
(3RS,4RS)-1-Aminocyclopentane-1,3,4-tricarboxylic acid	0.100 ±0.005	26336 ±2383	0.737			
(6R)-5,6,7,8-Tetrahydro-L-biopterin hydrochloride	0.108 ±0.014	28082 ±1242	0.786			
(9-Chloro-2-(2-furyl)(1,2,4)-triazolo(1,5-C)-quinazolin-5-amine)	0.104 ±0.011	51664 ±6877	1.447			
(Â±)-Sotalol hydrochloride	0.123 ±0.003	23750 ±1412	0.665			
(Â±)-Vesamicol hydrochloride	0.101 ±0.008	47161 ±2828	1.320			
(Arg-8)-Vasopressin (AVP)	0.128 ±0.001 *					
(D-Ala2,D-Leu5)-Enkephalin	0.104 ±0.004	52683 ±727	1.475			
(D-Ala2,N-Me-Phe4, Methionin (O)-ol5)-enkephalin	0.106 ±0.011	33761 ±5334	0.945			

(D-Ala2,N-Me-Phe4,glycinol5)-Enkephalin	0.104 ± 0.006	44473 ± 1849	1.245			
(D-Arg2,Lys4)-Dermorphin (1-4)-amide	0.102 ± 0.008	52206 ± 5240	1.462			
(D-Cys(tBu)2,Thr(tBu)6)-Leu-Enkephalin-Thr	0.105 ± 0.001	32016 ± 2819	0.896			
(D-Ser2)-Leu-Enkephalin-Thr	0.102 ± 0.006	51767 ± 2716	1.449			
(D-Thr2)-Leu-Enkephalin-Thr	0.110 ± 0.007	42201 ± 2126	1.182			
(E)-3-Acetoxy-5-methoxystilbene	0.117 ± 0.001	38753 ± 4564	1.085			
(E)-3-Hydroxy-5-methoxystilbene	0.118 ± 0.010	42106 ± 5577	1.179			
(E)-4-Amino-2-butenoic acid	0.111 ± 0.013	32285 ± 3287	0.904			
(E)-5-(2-Bromovinyl)-2'-deoxyuridine	0.101 ± 0.003	38734 ± 911	1.085			
(E)-8-(6-Hydroperoxy-3,7-dimethylocta-2,7-dienyloxy)-psoralen	0.123 ± 0.001	25977 ± 1749	0.727			
(Leu5)-Enkephalin	0.114 ± 0.008	47679 ± 4206	1.335			
(Met5)-Enkephalin	0.106 ± 0.005	45109 ± 1860	1.263			
(N)-α-Methylhistamine dihydrochloride	0.102 ± 0.010	25862 ± 1664	0.724			
(Phe4)-Dermorphin (1-4)-amide	0.105 ± 0.004	44175 ± 3658	1.237			
(R S)-3,5-DHPG	0.110 ± 0.002	36585 ± 4010	1.024			
(R S)-AMPA	0.108 ± 0.001	26953 ± 1696	0.755			
(R S)-MCPG	0.104 ± 0.006	39003 ± 4438	1.092			
(R S)-Rolipram	0.112 ± 0.001	49614 ± 3559	1.389			
(R)-(-)-Niguldipine hydrochloride	0.056 ± 0.005 **					
(R)-(-)-Rolipram	0.111 ± 0.006	54998 ± 1549	1.540			
(R)-(-)-α-Methylhistamine dihydrobromide	0.105 ± 0.003	36803 ± 2307	1.030			
(R)-(+)-Atenolol	0.111 ± 0.002	37540 ± 8256	1.051			
(R)-(+)-HA-966	0.116 ± 0.004	24836 ± 659	0.695			
(R)-(+)-Propranolol hydrochloride	0.108 ± 0.012	36816 ± 6374	1.031			
(R)-(+)-WIN 55,212-2 Mesylate	0.107 ± 0.013	65058 ± 1172	1.798			
(R)-3,4-DCPG	0.114 ± 0.009	37974 ± 2940	1.063			
(R)-3-Carboxy-4-hydroxyphenylglycine	0.110 ± 0.004	36737 ± 1178	1.029			
(R)-CR8	0.110 ± 0.004	31730 ± 2642	0.888			
(R)-DRF053 Dihydrochloride	0.115 ± 0.002	30099 ± 3254	0.843			
(R)-Methanandamide	0.116 ± 0.003	31514 ± 2038	0.882			
(R)-Naproxen sodium salt	0.108 ± 0.007	20858 ± 1020	0.569			
(R)-Propranolol hydrochloride	0.118 ± 0.006	37051 ± 2179	1.037			

(R)-α-Amino-3-hydroxy-5-methyl-4-isoxazolepropionic acid	0.109 ± 0.003	31282 ± 5748	0.876			
(R,E)-Deca-2-ene-4,6-diyn-1,8-diol	0.129 ± 0.007	27033 ± 5835	0.757			
(R,R)-cis-Diethyl tetrahydro-2,8-chrysenediol	0.099 ± 0.007	106233 ± 10107	2.936	0.118 ± 0.005	41862 ± 1472	1.281
(R,S)-3-(2-Piperidinyl)-pyridine	0.108 ± 0.009	22664 ± 4382	0.635			
(R,S)-3-Hydroxyphenylglycine	0.105 ± 0.004	30572 ± 2729	0.856			
(RS)-(±)-Sulpiride	0.111 ± 0.005	28887 ± 906	0.809			
(RS)-(Tetrazol-5-yl)-glycine	0.106 ± 0.007	40154 ± 7463	1.124			
(RS)-1-Amino-5-phosphonoindan-1-carboxylic acid	0.102 ± 0.006	27728 ± 1874	0.776			
(RS)-3,4-DCPG	0.116 ± 0.005	44600 ± 1328	1.249			
(RS)-3,5-DHPG	0.118 ± 0.004	30108 ± 1139	0.843			
(RS)-3,5-Dihydroxyphenylglycine	0.105 ± 0.010	38160 ± 1090	1.068			
(RS)-4-Carboxy-3-hydroxyphenylglycine	0.110 ± 0.003	33727 ± 3481	0.944			
(RS)-4-Phosphonophenylglycine	0.104 ± 0.007	25360 ± 1469	0.710			
(RS)-AMPA	0.104 ± 0.004	32290 ± 1732	0.904			
(RS)-AMPA Hydrobromide	0.122 ± 0.004	41073 ± 4375	1.150			
(RS)-Atenolol	0.109 ± 0.009	24613 ± 2242	0.689			
(RS)-CHPG	0.105 ± 0.002	33719 ± 1044	0.944			
(RS)-CHPG Sodium salt	0.118 ± 0.003	62788 ± 3679	1.758			
(RS)-α-Ethyl-4-carboxyphenylglycine	0.101 ± 0.013	24273 ± 2360	0.680			
(RS)-α-Methyl-4-carboxyphenylglycine	0.102 ± 0.009	30832 ± 4330	0.863			
(RS)-α-Methyl-4-phosphonophenylglycine	0.104 ± 0.008	27618 ± 2480	0.773			
(RS)-α-Methyl-4-sulfonophenylglycine	0.102 ± 0.010	33148 ± 2160	0.928			
(RS)-α-Methyl-4-tetrazolylphenylglycine	0.104 ± 0.018	34059 ± 3180	0.954			
(RS)-α-Methylserine-O-phosphate	0.113 ± 0.005	30358 ± 3715	0.850			
(S)-(-)-Atenolol	0.108 ± 0.002	37624 ± 1887	1.053			
(S)-(-)-Bay K 8644	0.120 ± 0.003	45194 ± 938	1.265			
(S)-(-)-Carbidopa	0.116 ± 0.004	19546 ± 921	0.541			
(S)-(-)-Cycloserine	0.098 ± 0.011	30241 ± 154	0.847			
(S)-(-)-HA-966	0.107 ± 0.008	21570 ± 921	0.597			
(S)-(-)-Pindolol	0.116 ± 0.011	45008 ± 5312	1.260			
(S)-(-)-Propranolol hydrochloride	0.112 ± 0.010	35996 ± 5511	1.008			
(S)-(-)-Sulpiride	0.135 ± 0.007 *					

(S)-(+)-Camptothecin	0.100 ± 0.005	41647 ± 2972	1.166			
(S)-(+)-Dimethindene maleate	0.122 ± 0.006	28734 ± 3628	0.805			
(S)-(+)-Niguldipine hydrochloride	0.058 ± 0.006 **					
(S)-(+)-Rolipram	0.114 ± 0.005	53454 ± 1399	1.497			
(S)-(+)-α-Methylhistamine dihydrobromide	0.104 ± 0.012	31590 ± 3876	0.885			
(S)-2-Amino-2-methyl-4-phosphonobutanoic acid	0.105 ± 0.006	24303 ± 2164	0.680			
(S)-3,4-DCPG	0.112 ± 0.002	40911 ± 2514	1.145			
(S)-3,4-Dicarboxyphenylglycine	0.101 ± 0.009	28968 ± 1971	0.811			
(S)-3,5-DHPG	0.109 ± 0.006	41912 ± 2116	1.174			
(S)-3,5-Dihydroxyphenylglycine	0.100 ± 0.010	37156 ± 2761	1.040			
(S)-3-Carboxy-4-hydroxyphenylglycine	0.114 ± 0.008	19206 ± 1541	0.514			
(S)-3-Hydroxyphenylglycine	0.101 ± 0.011	24760 ± 765	0.693			
(S)-4-Carboxy-3-hydroxyphenylglycine	0.106 ± 0.007	21691 ± 3081	0.607			
(S)-4-Carboxyphenylglycine	0.101 ± 0.006	21441 ± 2911	0.600			
(S)-5-Fluorowillardiine	0.109 ± 0.003	37425 ± 3321	1.048			
(S)-5-Lodowillardiine	0.116 ± 0.001	65750 ± 8046	1.841			
(S)-5-Nitrowillardiine	0.107 ± 0.002	30284 ± 3203	0.848			
(S)-AMPA	0.109 ± 0.002	41931 ± 1258	1.174			
(S)-MAP4 Hydrochloride	0.102 ± 0.009	32649 ± 1422	0.914			
(S)-MCPG	0.112 ± 0.005	28997 ± 1857	0.812			
(S)-Propranolol hydrochloride	0.105 ± 0.005	28310 ± 1610	0.793			
(S)-Rolipram	0.112 ± 0.003	53306 ± 1833	1.493			
(S)-SNAP 5114	0.102 ± 0.005	36230 ± 3964	1.014			
(S)-Timolol Maleate	0.103 ± 0.006	43049 ± 4135	1.205			
(S)-Willardiine	0.105 ± 0.004	32548 ± 612	0.911			
(S)-α-Methyl-4-carboxyphenylglycine	0.101 ± 0.011	22432 ± 3691	0.628			
(S,E)-Deca-2,9-dien-4,6-diyn-1,8-diol	0.136 ± 0.007 **					
(Z)-Akuammidine	0.112 ± 0.007	34327 ± 1749	0.961			
(Z-LL)2 Ketone	0.107 ± 0.009	34016 ± 1753	0.952			
1-(1,3-Benzodioxol-5-ylcarbonyl)-piperidine	0.113 ± 0.009	51584 ± 8242	1.444			
1-(1-Naphthyl)-piperazine hydrochloride	0.105 ± 0.008	25335 ± 5078	0.709			
1-(2-Chlorophenyl)-1-(4-chlorophenyl)-2,2-dichloroethane	0.102 ± 0.005	37542 ± 2294	1.051			

1-(2-Methoxyphenyl)-4-(4-succinimidobutyl)-piperazine	0.105 ± 0.012	37921 ± 1450	1.062			
1-(2-Methoxyphenyl)-pierazine hydrochloride	0.103 ± 0.002	36701 ± 2316	1.028			
1-(2-Methyl-5-nitro-imidazol-1-yl)-propan-2-ol	0.114 ± 0.003	49149 ± 4490	1.376			
1-(3,4-Dimethoxyphenyl)-propane-1,2-diol	0.127 ± 0.004	36820 ± 3377	1.031			
1-(3-Chlorophenyl)-piperazine	0.102 ± 0.005	35209 ± 2333	0.986			
1-(3-Trifluoromethyl)-phenylpiperazine monohydrochloride	0.107 ± 0.003	52114 ± 3726	1.459			
1-(4'-Aminophenyl)-3,5-dihydro-7,8-dimethoxy-4H-2,3-benzodiazepin-4-one	0.115 ± 0.007	29442 ± 4358	0.824			
1-(4-Chlorobenzyl)-5-methoxy-2-methylindole-3-acetic acid	0.103 ± 0.002	32348 ± 3520	0.906			
1-(4-Hexyphenyl)-2-propane-1-one	0.105 ± 0.008	37058 ± 5506	1.038			
1-(4-Hydroxybenzyl)-imidazole-2-thiol	0.103 ± 0.006	32909 ± 2673	0.921			
1-(4-Iodophenyl)-3-(1-adamantyl)-guanidine	0.104 ± 0.007	28976 ± 540	0.811			
1-(4-Iodophenyl)-3-(2-adamantyl)-guanidine	0.101 ± 0.011	26233 ± 2599	0.735			
1-(5-Isoquinolinylsulfonyl)-2-methylpiperazine dihydrochloride	0.104 ± 0.003	34586 ± 3474	0.968			
1-(5-Isoquinolinylsulfonyl)-3-methylpiperazine dihydrochloride	0.103 ± 0.001	31272 ± 2396	0.876			
1-(m-Chlorophenyl)-biguanide hydrochloride	0.103 ± 0.009	44220 ± 3439	1.238			
1,10:4,5-Diepoxy-7(11)-germacren-8-one	0.124 ± 0.000	32140 ± 990	0.900			
1,10-Diaminodecane	0.098 ± 0.008	50702 ± 2185	1.401			
1,10-Phenanthroline monohydrate	0.076 ± 0.005 **					
1,18-Octadecanediol	0.125 ± 0.004	39977 ± 2323	1.119			
1,1-Dimethyl-4-phenylpiperazinium iodide	0.101 ± 0.008	39339 ± 2274	1.101			
1,1-Dimethylbiguanide, Hydrochloride	0.107 ± 0.011	31536 ± 3567	0.883			
1,1'-Ethylidene-bis-L-Tryptophan	0.110 ± 0.000	34276 ± 1751	0.960			
1,2,3,19-Tetrahydroxy-12-ursen-28-oic acid	0.114 ± 0.010	34497 ± 2015	0.966			
1,2,3,4,5,6-Hexabromocyclohexane	0.106 ± 0.002	32090 ± 1955	0.899			
1,2:4,5-Di-O-isopropylidene-β-D-fructopyranose	0.128 ± 0.007	24251 ± 2054	0.679			
1,25-Dihydroxyvitamin D3	0.114 ± 0.002	30932 ± 2054	0.866			
1,2-Benzenediol	0.117 ± 0.004	32459 ± 1133	0.909			
1,2-Diacetoxy-4,7,8-trihydroxy-3-(4-hydroxyphenyl)-dibenzofuran	0.120 ± 0.002	26068 ± 903	0.730			
1,2-Didecanoyl-glycerol (10:0)	0.109 ± 0.007	32036 ± 1461	0.897			
1,2-Dimethylhydrazine hydrochloride	0.112 ± 0.004	28620 ± 998	0.801			
1,2-Dioctanoyl-SN-glycerol	0.113 ± 0.004	33330 ± 1012	0.933			
1,2-Dioleoyl-glycerol (18:1)	0.110 ± 0.002	31296 ± 1494	0.876			

1,2-Dithiole-3-thione	0.117 ± 0.005	18631 ± 2925	0.520			
1,2-O-Isopropylidene-β-D-fructopyranose	0.128 ± 0.007	30485 ± 5212	0.854			
1,2-α-Epoxydeacetoxydihydrogedunin	0.115 ± 0.002	31187 ± 1638	0.873			
1,3,5(10)-Estratrien-3-Ol-17-One Sulphate	0.131 ± 0.001 **					
1,3,5-Cadinatriene-3,8-diol	0.125 ± 0.005	35141 ± 4680	0.984			
1,3,5-Trihydroxy-4-prenylxanthone	0.131 ± 0.005	50795 ± 2465	1.422			
1,3,5-Tris(4-hydroxyphenyl)-4-propyl-1H-pyrazole	0.062 ± 0.005 **					
1,3,7-Trihydroxy-2-prenylxanthone	0.125 ± 0.006	39261 ± 1722	1.099			
1,3-Di(2-tolyl)-guanidine	0.100 ± 0.002	36135 ± 869	1.012			
1,3-Dideacetyl-7-deacetoxy-7-oxokhivorin	0.094 ± 0.003 *					
1,3-Dihydroxy-4-methoxy-10-methylacridin-9(10H)-one	0.114 ± 0.005	45446 ± 1541	1.272			
1,3-Dimethyl-8-phenylxanthine	0.103 ± 0.014	29977 ± 3627	0.839			
1,3-Dipropyl-7-methylxanthine	0.101 ± 0.007	33155 ± 545	0.928			
1,3-Dipropyl-8-phenylxanthine	0.106 ± 0.011	35620 ± 2578	0.997			
1,3-Dipropyl-8-p-sulfophenylxanthine	0.100 ± 0.002	38292 ± 5523	1.072			
1,4,5,6-Tetrahydroxy-7,8-diprenylxanthone	0.125 ± 0.001	35219 ± 2967	0.986			
1,4,5,6-Tetrahydroxy-7-prenylxanthone	0.122 ± 0.002	46891 ± 4240	1.313			
1,4,5,8-Tetrahydroxy-2,6-dimethylanthroquinone	0.098 ± 0.011	22651 ± 2261	0.634			
1,4,6-Trihydroxy-5-methoxy-7-prenylxanthone	0.117 ± 0.008	40444 ± 4689	1.132			
1,4,7-Eudesmanetriol	0.118 ± 0.003	37428 ± 2006	1.048			
1,4-Dideoxy-1,4-imino-D-arabinitol	0.099 ± 0.012	40262 ± 1356	1.127			
1,4-Dihydro-1,2-dimethyl-4-oxo-3-quinolinecarboxylic acid	0.108 ± 0.005	23770 ± 820	0.666			
1,4-Naphthoquinone	0.097 ± 0.007	32920 ± 2015	0.922			
1,4-PBIT dihydrobromide	0.107 ± 0.002	47427 ± 3888	1.328			
1,5,15-Tri-O-methylmorindol	0.114 ± 0.004	27434 ± 3018	0.768			
1,5,8-Trihydroxy-3-methoxy-2-prenylxanthone	0.135 ± 0.009	24590 ± 643	0.689			
1,5-Isoquinolinediol	0.103 ± 0.001	23076 ± 3606	0.646			
1,6,7-Trihydroxyxanthone	0.126 ± 0.005	38145 ± 2759	1.068			
1,6-Diacetoxy-4(15),11(13)-eudesmadien-12-oic acid methyl ester	0.118 ± 0.005	41311 ± 2283	1.157			
1,6-Dioxaspiro[4.5]decan-2-methanol	0.114 ± 0.008	26780 ± 619	0.750			
1,7-Bis(4-hydroxyphenyl)-hept-1-en-3-one	0.116 ± 0.003	43801 ± 3013	1.226			
1,7-Dideacetoxy-1,7-dioxo-3-deacetylkhivorin	0.107 ± 0.003	32786 ± 3274	0.918			

1,7-Diepi-8,15-cedranediol	0.131 ± 0.008	37782 ± 2275	1.058			
1,7-Dihydroxy-3-methoxy-2-prenylxanthone	0.123 ± 0.002	43980 ± 2585	1.231			
1,7-Dimethylxanthine	0.100 ± 0.007	38681 ± 2002	1.083			
1,9-Caryolanediol 9-acetate	0.123 ± 0.001	38134 ± 2095	1.068			
1,9-Pyrazoloanthrone	0.125 ± 0.011	31940 ± 3661	0.894			
1-[1-(2-Benzo[B]thienyl)-cyclohexyl)]-Piperidine maleate	0.120 ± 0.004	26009 ± 1098	0.728			
1-[1-(2-Benzo[B]thienyl)-cyclohexyl]-Pyrrolidine fumarate	0.117 ± 0.006	30972 ± 3577	0.867			
1-[2-(Trifluoromethyl)-phenyl]-Imidazole	0.116 ± 0.013	31173 ± 2277	0.873			
1-[2-Chloro-6-[[(3-iodophenyl)-methyl]amino] -9H-purin-9-yl]-1-deoxy-N-methyl-β-D-ribofuranuronamide	0.091 ± 0.003 *					
10(14)-Cadinene-4,5-diol	0.124 ± 0.001	43409 ± 919	1.215			
10(E)-Heptadecenoic acid	0.115 ± 0.002	36987 ± 1748	1.036			
10(E)-Nonadecenoic acid	0.104 ± 0.003	26771 ± 4275	0.750			
10(E)-Pentadecenoic acid	0.115 ± 0.011	27633 ± 1516	0.774			
10(Z),13(Z)-Nonadecadienoic acid	0.120 ± 0.004	24632 ± 2121	0.690			
10(Z)-Heptadecenoic acid	0.112 ± 0.013	30528 ± 771	0.855			
10(Z)-Nonadecenoic acid	0.132 ± 0.015	23020 ± 3390	0.645			
10(Z)-Pentadecenoic acid	0.119 ± 0.006	29468 ± 1202	0.825			
10058-F4	0.102 ± 0.002	58193 ± 2261	1.608			
10-Acetoxyscandine	0.122 ± 0.014	34691 ± 1246	0.971			
10-Carboxymethylacridanone	0.109 ± 0.010	42621 ± 4507	1.193			
10-Deacetylbaccatin III	0.119 ± 0.021	45037 ± 4546	1.261			
10-DEBC hydrochloride	0.100 ± 0.003	5100 ± 119	0.141	0.109 ± 0.008	24785 ± 2766	0.758
10H-Phenothiazine, 2-chloro-10-[3-(4-methyl-1-piperazinyl)-propyl	0.118 ± 0.004	18836 ± 3197	0.524			
10-Hydroxycamptothecin	0.111 ± 0.008	23876 ± 2498	0.669			
10-Hydroxydihydroperaksine	0.105 ± 0.007	40903 ± 4291	1.145			
10-Hydroxyscandine	0.128 ± 0.005	36414 ± 770	1.020			
10-O-Acetylisocalamendiol	0.127 ± 0.005	41823 ± 699	1.171			
10-O-Vanilloylaucubin	0.126 ± 0.007	36665 ± 1928	1.027			
10-Undecenoic acid	0.116 ± 0.029	37544 ± 2795	1.051			
11(13)-Dehydroivaxillin	0.102 ± 0.013	11781 ± 1932	0.329	0.099 ± 0.007	17602 ± 818	0.539
11(E)-Eicosenoic acid	0.106 ± 0.012	37262 ± 1037	1.043			

11(E)-Octadecenoic acid	0.107 ±0.026	22799 ±4648	0.638			
11(S),16(R)-Dihydroxyoctadeca-9Z,17-diene-12,14-diyn-1-yl acetate	0.131 ±0.005	27110 ±1603	0.759			
11(Z),14(Z),17(Z)-Eicosatrienoic acid	0.103 ±0.024	26106 ±1102	0.731			
11(Z),14(Z)-Eicosadienoic acid	0.117 ±0.035	26301 ±3442	0.736			
11(Z)-Eicosenoic acid	0.106 ±0.002	43295 ±1575	1.358			
11(Z)-Octadecenoic acid	0.107 ±0.004	36482 ±1784	1.021			
11,12-Demethylenedioxy-Danuphylline	0.132 ±0.006	26895 ±1259	0.753			
11,12-Di-O-acetyltenacigenin B	0.125 ±0.001	34426 ±1654	0.964			
11,12-Epoxyeicosatrienoic acid	0.111 ±0.003	31935 ±724	0.894			
11,13-Dihydroivalin	0.123 ±0.004	36947 ±2336	1.034			
11,15-Dihydroxy-16-kauren-19-oic acid	0.116 ±0.006	35043 ±1677	0.981			
1-13-Chlorophenyl 1-4-N-Hexylpiperazine dihydrochloride	0.107 ±0.012	48825 ±3779	1.367			
11-Dehydro-thromboxane B2	0.113 ±0.004	33190 ±282	0.929			
11-Dehydroxygrevilloside B	0.119 ±0.007	38478 ±3574	1.077			
11-Dodecenoic acid	0.115 ±0.003	33065 ±2629	0.926			
11-Hydroxycodaphniphylline	0.121 ±0.002	26312 ±1657	0.737			
11-Hydroxyjasmonic acid	0.123 ±0.002	39786 ±3010	1.114			
11-Hydroxytabersonine	0.129 ±0.004	35693 ±1901	0.999			
11-Methoxyuncarine C	0.125 ±0.004	43099 ±5302	1.207			
11-O-(3-nitrobenzoyl)-bergenin	0.119 ±0.004	37254 ±3548	1.043			
11-α-Acetoxyprogesterone	0.103 ±0.008	30950 ±1302	0.867			
11-β-Hydroxycedrelone	0.125 ±0.001	34470 ±1172	0.965			
12(R)-HETE	0.102 ±0.002	29065 ±1004	0.814			
12(S),20-DiHETE	0.099 ±0.004	32238 ±2578	0.903			
12(S)-HETE	0.106 ±0.008	28575 ±1397	0.800			
12(S)-HHT	0.105 ±0.004	26903 ±3677	0.753			
12(S)-HPETE	0.101 ±0.006	24827 ±2269	0.695			
12(Z),15(Z)-Heneicosadienoic acid	0.114 ±0.018	29452 ±2968	0.825			
12(Z)-Heneicosenoic acid	0.127 ±0.001 **					
12-Acetoxyabietic acid	0.106 ±0.017	33536 ±500	0.939			
12a-hydroxy-9-demethylmunduserone-8-carboxylic acid	0.108 ±0.007	31439 ±2775	0.880			
12-Ethoxyabietic acid	0.118 ±0.002	48266 ±4641	1.351			

12-hydroxy-4,4-bisnor-4,8,11,13-podocarpatetraen-3-one	0.114 ±0.005	27625 ±3105	0.773			
12-Hydroxy-8(17),13-labdadien-16,15-olide	0.122 ±0.011	32715 ±693	0.916			
12-Hydroxyabietic acid	0.111 ±0.002	30198 ±1719	0.846			
12-Hydroxyisodrimenin	0.113 ±0.004	30301 ±3476	0.848			
12-Hydroxyjasmonic acid	0.130 ±0.009	33748 ±976	0.945			
12-Methoxydodecanoic acid	0.109 ±0.003	25005 ±1844	0.700			
12-Tridecenoic acid	0.112 ±0.004	37227 ±8903	1.042			
12-Ursene-3,16,22-triol	0.127 ±0.017	40000 ±3349	1.120			
12-α-Hydroxyevodol	0.134 ±0.002	42411 ±7258	1.187			
13(18)-Oleanen-3-ol	0.126 ±0.003	32237 ±1933	0.903			
13(18)-Oleanen-3-one	0.119 ±0.006	33012 ±1395	0.924			
13(E)-Docosenoic acid	0.108 ±0.004	35516 ±2322	0.994			
13(S)-HODE	0.110 ±0.003	33052 ±1235	0.925			
13(S)-HOTE	0.114 ±0.004	34148 ±778	0.956			
13(S)-HPODE	0.115 ±0.007	31961 ±955	0.895			
13(S)-Hydroxy-9Z,11E-octadecadienoic acid	0.117 ±0.004	44899 ±2097	1.257			
13(Z),16(Z),19(Z)-Docosatrienoic acid	0.103 ±0.004	32407 ±4380	0.907			
13(Z),16(Z)-Docosadienoic acid	0.110 ±0.020	36186 ±2835	1.013			
13(Z)-Docosenoic acid	0.107 ±0.004	35572 ±4089	0.996			
13,14-Dihydro-15-keto-prostaglandin F2a	0.101 ±0.001	24326 ±1538	0.681			
13,14-Dihydro-prostaglandin E1	0.112 ±0.003	32068 ±1403	0.898			
13-cis Retinoic acid	0.113 ±0.004	30356 ±340	0.850			
13-cis-Retinol	0.108 ±0.006	20931 ±3157	0.586			
13-Hpode	0.112 ±0.002	43612 ±3457	1.221			
13-Hydroxy-8,11,13-podocarpatrien-18-oic acid	0.119 ±0.008	41933 ±1652	1.174			
13-Hydroxylabda-8(17),14-dien-18-oic acid	0.112 ±0.002	24494 ±1448	0.686			
13-Ketooctadecadienoic acid	0.103 ±0.008	36369 ±3578	1.018			
13-Methyl-4,4-bisnor-8,11,13-podocarpatrien-3-one	0.104 ±0.009	30606 ±633	0.857			
13-Methyl-8,11,13-podocarpatriene-3,12-diol	0.120 ±0.002	50261 ±3059	1.407			
13-O-Acetylcorianin	0.124 ±0.011	40166 ±6288	1.125			
13-O-Deacetyltaxumairol Z	0.128 ±0.006	34514 ±1694	0.966			
13-O-p-Coumaroylplumieride	0.118 ±0.006	35013 ±2500	0.980			

14(E)-Tricosenoic acid	0.105 ±0.010	34522 ±3762	0.967			
14(Z)-Tricosenoic acid	0.106 ±0.002	34496 ±2503	0.966			
14,15-Dehydro-leukotriene B4	0.109 ±0.001	35997 ±1312	1.008			
14,15-Didehydroisoeburnamine	0.136 ±0.006	34062 ±1910	0.954			
14,15-Didehydrovincamenine	0.123 ±0.001	42523 ±1149	1.191			
14,15-Epoxyeicosatrienoic acid	0.110 ±0.001	29911 ±1187	0.837			
14,17-Epidioxy-28-nor-15-taraxerene-2,3-diol	0.121 ±0.021	44757 ±4050	1.253			
1400W	0.115 ±0.005	38528 ±1895	1.079			
1400W dihydrochloride	0.117 ±0.004	28166 ±1520	0.789			
14-Deoxy-11,12-didehydroandrographolide	0.117 ±0.007	28353 ±5938	0.794			
14-Deoxy-11-hydroxyandrographolide	0.125 ±0.001	38439 ±532	1.076			
14-Deoxy-12-hydroxyandrographolide	0.127 ±0.001	24004 ±838	0.672			
14-Deoxy-17-hydroxyandrographolide	0.130 ±0.004	32747 ±707	0.917			
14-O-Methylthiomethylitol A	0.135 ±0.009	26804 ±2171	0.750			
14-β-Benzoyloxy-2-deacetylbaccatin VI	0.127 ±0.002	37061 ±1120	1.038			
15(S)-HEDE	0.108 ±0.003	29208 ±485	0.818			
15(S)-HEPE	0.104 ±0.004	31353 ±424	0.878			
15(S)-HETE	0.103 ±0.003	30292 ±848	0.848			
15(S)-HETrE	0.103 ±0.005	36332 ±4511	1.017			
15(S)-HPEDE	0.102 ±0.002	30690 ±841	0.859			
15(S)-HPEPE	0.107 ±0.009	29429 ±223	0.824			
15(S)-HPETE	0.108 ±0.004	26459 ±485	0.741			
15(Z)-Tetracosenoic acid	0.101 ±0.012	39866 ±2850	1.116			
15,16-Dihydro-15-methoxy-16-oxohardwickiic acid	0.126 ±0.006	43509 ±5276	1.218			
15,16-Dihydrotanshinone I	0.108 ±0.017	38247 ±1043	1.071			
15,16-Dinor-8(17),11-labdadien-13-one	0.123 ±0.003	30937 ±2847	0.866			
15,16-Di-O-acetyldarutoside	0.131 ±0.011	35713 ±3159	1.000			
15,16-Epoxy-12R-hydroxylabda-8(17),13(16),14-triene	0.126 ±0.001	40859 ±5913	1.144			
15,16-Epoxy-12S-hydroxylabda-8(17),13(16),14-triene	0.125 ±0.007	29461 ±2356	0.825			
15,18-Dihydroxy-8,11,13-abietatrien-7-one	0.108 ±0.003	39780 ±3109	1.114			
15-Demethylplumieride	0.118 ±0.002	40354 ±3275	1.130			
15-Deoxoeucosterol	0.117 ±0.001	41068 ±3118	1.150			

15-Deoxy-D12,14-prostaglandin J2	0.134 ± 0.004	8986 ± 574	0.238	0.119 ± 0.001	11556 ± 557	0.354
15d-Prostaglandin J2	0.112 ± 0.003	32590 ± 2828	0.913			
15-Ethoxychinensine A	0.123 ± 0.006	27774 ± 1428	0.778			
15-Hydroxy-7-oxo-8,11,13-abietatrien-18-oic acid	0.124 ± 0.003	43086 ± 5812	1.206			
15-Hydroxydehydroabietic acid	0.111 ± 0.013	30124 ± 947	0.843			
15-Isopimarene-8,18-diol	0.119 ± 0.002	34662 ± 2525	0.971			
15-Ketoeicosatetraenoic acid	0.117 ± 0.005	28228 ± 3733	0.790			
15-Keto-prostaglandin E2	0.112 ± 0.005	28666 ± 699	0.803			
15-Keto-prostaglandin F2a	0.105 ± 0.007	32831 ± 953	0.919			
15-Methoxychinensine A	0.129 ± 0.008	26409 ± 6694	0.739			
15-Methoxypinusolidic acid	0.120 ± 0.004	32385 ± 1779	0.907			
15-Nor-14-oxolabda-8(17),12-dien-18-oic acid	0.119 ± 0.002	35172 ± 2577	0.985			
16,16-Dimethyl-prostaglandin E2	0.098 ± 0.002 *					
16-Acetoxy-7-O-acetylhorminone	0.125 ± 0.004	32100 ± 956	0.899			
16-Hydroxy-8(17),13-labdadien-15,16-olid-19-oic acid	0.117 ± 0.003	32753 ± 3139	0.917			
16-Kaurene-2,6,15-triol	0.132 ± 0.007	36077 ± 1720	1.010			
16-O-Acetyldarutigenol	0.110 ± 0.016	30996 ± 4046	0.868			
16-O-Methyl-14,15-didehydroisovincanol	0.117 ± 0.004	31097 ± 2447	0.871			
16-Oxoprometaphanine	0.115 ± 0.004	41006 ± 1354	1.148			
17-Allylamino-geldanamycin	0.110 ± 0.002	23776 ± 2292	0.666			
17-Hydroxyprogesterone	0.120 ± 0.002	30099 ± 1636	0.843			
17-Octadecynoic acid	0.105 ± 0.004	35466 ± 1188	0.993			
17-PA	0.102 ± 0.002	45693 ± 709	1.279			
17-Phenyl-trinor-prostaglandin E2	0.108 ± 0.004	25428 ± 3436	0.712			
17-α-Hydroxyprogesterone	0.101 ± 0.004	44872 ± 1799	1.256			
17-α-Neriifolin	0.120 ± 0.003	39165 ± 1664	1.097			
17-α-Thevebioside	0.125 ± 0.002	35069 ± 2422	0.982			
17-β-Estradiol	0.113 ± 0.007	89082 ± 619	2.606	0.109 ± 0.005	36174 ± 2679	1.107
18-Aminoabieta-8,11,13-triene sulfate	0.135 ± 0.002 **					
18-Nor-8,11,13-Abietatriene-4,15-diol	0.131 ± 0.008	29143 ± 890	0.816			
18-Norabieta-8,11,13-trien-4-ol	0.120 ± 0.004	42804 ± 2250	1.198			
18-O-Demethyldiosbulbin F	0.118 ± 0.003	37051 ± 1756	1.037			

18-α-Glycyrrhetinic acid	0.114 ± 0.002	42024 ± 3937	1.177			
19(R)-Hydroxy-prostaglandin E2	0.112 ± 0.012	25968 ± 606	0.727			
19(R)-Hydroxy-prostaglandin F2a	0.101 ± 0.003	39256 ± 2069	1.099			
19(S)-Acetoxy-11-methoxytabersonine	0.128 ± 0.004	42512 ± 4662	1.190			
19,20-(E)-Isovallesamine	0.119 ± 0.009	44626 ± 1583	1.250			
19,20-(E)-Vallesamine	0.119 ± 0.003	31569 ± 361	0.884			
19-[(β-D-glucopyranosyl)-oxy]-19-oxo-ent-labda-8(17),13-dien-16,15-olide	0.124 ± 0.002	35977 ± 434	1.007			
19-Hydroxybaccatin III	0.136 ± 0.007	40047 ± 3668	1.121			
19-Nor-4-hydroxyabieta-8,11,13-trien-7-one	0.123 ± 0.003	44488 ± 5082	1.246			
19-Nortestosterone	0.112 ± 0.011	37520 ± 4062	1.051			
1a,25-Dihydroxyvitamin D3	0.129 ± 0.012	34676 ± 1889	0.971			
1-Acetyl-4-methylpiperazine hydrochloride	0.108 ± 0.001	30324 ± 773	0.849			
1-Acetyltagitinin A	0.122 ± 0.002	32162 ± 6099	0.901			
1-Acyl-PAF	0.108 ± 0.004	30776 ± 591	0.862			
1-Allyl-3,7-dimethyl-8-phenylxanthine	0.103 ± 0.008	35429 ± 3508	0.992			
1-Allyl-3,7-dimethyl-8-p-sulfophenylxanthine	0.099 ± 0.005	33253 ± 4734	0.931			
1-Amino-1-cyclohexanecarboxylic acid hydrochloride	0.105 ± 0.003	32886 ± 330	0.921			
1-Amino-5-bromouracil	0.104 ± 0.003	38579 ± 2983	1.080			
1-Aminobenzotriazole	0.100 ± 0.001	40873 ± 3902	1.144			
1-Aminocyclobutane-cis-1,3-dicarboxylic acid	0.103 ± 0.007	31846 ± 5911	0.892			
1-Aminocyclobutane-trans-1,3-dicarboxylic acid	0.102 ± 0.002	25501 ± 3889	0.714			
1-Aminocyclopropanecarboxylic acid hydrochloride	0.101 ± 0.002	37038 ± 4138	1.037			
1-Azakenpaullone	0.114 ± 0.002	29147 ± 4393	0.816			
1-BCP	0.118 ± 0.005	29776 ± 5502	0.834			
1-benzoyl-5-methoxy-2-methylindole-3-acetic acid	0.102 ± 0.009	35204 ± 1953	0.986			
1-Benzyl-1-methyl-4-cyclopentylmethoxycarbonylpiperidinium bromide	0.102 ± 0.009	24683 ± 2838	0.691			
1-Benzylimidazole	0.125 ± 0.004	95096 ± 3818	2.648	0.117 ± 0.009	45126 ± 3660	1.381
1-Deacetylnimbolinin B	0.118 ± 0.003	45986 ± 2386	1.288			
1-Decarboxy-3-oxo-ceanothic acid	0.124 ± 0.014	30009 ± 3414	0.840			
1-Dehydroxy-23-deoxojessic acid	0.124 ± 0.002	27960 ± 2715	0.783			
1-Deoxy-1-[6-[[(3-iodophenyl)-methyl]amino]<br>-9H-purin-9-yl]-N-methyl-β-D-ribofuranuronamide	0.106 ± 0.007	70596 ± 4239	1.888			

1-Deoxymannojirimycin hydrochloride	0.113 ±0.003	37777 ±2046	1.058			
1-Deoxynojirimycin hydrochloride	0.098 ±0.004	38637 ±6445	1.082			
1-EBIO	0.113 ±0.008	30219 ±1241	0.846			
1H-Cyclopenta[b]quinolin-9-amine, 2,3,5,6,7,8-hexahydro-, monohydrochloride	0.105 ±0.005	30653 ±1047	0.858			
1-Hexadecyl-2-arachidonoyl-glycerol	0.117 ±0.004	31982 ±774	0.895			
1-Hexadecyl-2-methylglycero-3 PC	0.102 ±0.002	35913 ±438	1.006			
1-Hexadecyl-2-O-acetyl-glycerol	0.105 ±0.003	29720 ±4430	0.832			
1-Hexadecyl-2-O-methyl-glycerol	0.108 ±0.004	29234 ±467	0.819			
1H-Imidazol-2-amine, N-(2,6-dichlorophenyl)-4,5-dihydro	0.119 ±0.008	52970 ±1814	1.475			
1H-Imidazole-5-carboxylic acid, 1-(1-phenylethyl)-, ethyl ester, (R)	0.114 ±0.004	45760 ±6254	1.281			
1H-Indole-2-propanoic acid, 1-[(4-chlorophenyl) -methyl]-3-[(1,1-dimethylethyl)-thio]-α-dimethyl-5-(1-methylethyl)	0.131 ±0.003 *					
1H-Indole-3-carboxylic acid	0.089 ±0.002 **					
1-Hydroxy-1-(4-hydroxy-2-methoxyphenyl)-3-(4-hydroxyphenyl)-propan-2-one	0.125 ±0.003	29295 ±1056	0.820			
1-Hydroxy-2-oxopomolic acid	0.117 ±0.010	47963 ±3069	1.343			
1-Hydroxy-2-prenylnaphthalene	0.120 ±0.002	49879 ±4596	1.397			
1-Hydroxy-3,6,7-trimethoxy-2,8-diprenylxanthone	0.108 ±0.004	39490 ±645	1.106			
1-Hydroxybaccatin I	0.123 ±0.006	40875 ±160	1.144			
1-Hydroxycanthin-6-one	0.125 ±0.003	41790 ±2846	1.170			
1-Hydroxyrutaecarpine	0.112 ±0.011	34319 ±1248	0.961			
1-Isomangostin hydrate	0.127 ±0.007	38590 ±4434	1.080			
1-Methoxycarbonyl-β-carboline	0.124 ±0.004	31142 ±3347	0.872			
1-Methyl-2-heptyl-4(1H)-quinolinone	0.136 ±0.014	54463 ±3621	1.525			
1-Methyl-2-pentyl-4(1H)-quinolinone	0.131 ±0.015	47228 ±5026	1.322			
1-Methyl-4-[2-(2-naphthyl)-ethenyl]-pyridinium iodide	0.111 ±0.001	23870 ±1363	0.668			
1-Methyl-6-methoxy-1,2,3,4-tetrahydro-β-carboline	0.111 ±0.004	33100 ±4955	0.927			
1-Methyladenosine	0.105 ±0.007	35064 ±1592	0.982			
1-Methylhistamine dihydrochloride	0.113 ±0.008	33324 ±1835	0.933			
1-Methylimidazole	0.111 ±0.002	34651 ±2072	0.970			
1-Monopalmitin	0.101 ±0.010	34053 ±834	0.953			
1-Naphthyl PP1	0.107 ±0.005	37219 ±3246	1.042			
1-Octadecyl-2-methylglycero-3 PC	0.104 ±0.001	27673 ±1848	0.775			

1-O-Deacetyl-2-α-hydroxykhayanolide E	0.130 ± 0.007	39115 ± 2356	1.095			
1-O-Deacetylkhayanolide E	0.116 ± 0.001	35158 ± 2919	0.984			
1-Oleoyl-2-acetyl-glycerol	0.112 ± 0.008	32833 ± 1593	0.919			
1-Oxo-4-hydroxy-2-en-4-ethylcyclohexa-5,8-olide	0.131 ± 0.005	37415 ± 1198	1.048			
1-Phenyl-3-(2-thiazolyl)-2-thiourea	0.091 ± 0.001 *					
1-Phenylbiguanide	0.103 ± 0.013	38687 ± 2052	1.083			
1-Phenylbiguanide hydrochloride	0.104 ± 0.008	28887 ± 3708	0.809			
1-Stearoyl-2-arachidonoyl-glycerol	0.115 ± 0.006	32583 ± 2473	0.912			
1-Stearoyl-2-linoleoyl-glycerol	0.113 ± 0.001	28520 ± 432	0.799			
2",4"-Di-O-(Z-p-coumaroyl)-afzelin	0.124 ± 0.027	26190 ± 349	0.733			
2(1H)-Pyrimidinone, 4-amino-1-Ã‚Â¿-D-arabinofuranosyl	0.114 ± 0.005	31023 ± 5242	0.869			
2-(2-Aminoethyl)-isothiourea dihydrobromide	0.096 ± 0.004	25728 ± 1934	0.720			
2-(2-Aminoethyl)-pyridine	0.120 ± 0.002	27851 ± 1727	0.780			
2-(2'-Hydroxytetracosanoylamino)-octadecane-1,3,4-triol	0.137 ± 0.009	35759 ± 1229	1.001			
2-(3,4-Dichlorophenyl)-N-methyl-N-[(1S)<br>-1-(3-isothiocyanatophenyl)-2-(1-pyrrolidinyl)-ethyl]acetamide	0.070 ± 0.002 **					
2-(3,4-Dihydroxyphenyl)-ethanol	0.124 ± 0.004	38595 ± 2050	1.081			
2-(4-Chloro-3-hydroxybuten-1-yl)-5-(pentadien-1,3-yl)-thiophene	0.133 ± 0.006	25932 ± 6548	0.726			
2-(4-Hydroxy-3-methoxyphenyl)-7-methoxy-5-benzofuranpropanol	0.117 ± 0.005	41401 ± 3818	1.159			
2-(4-Hydroxyphenyl)-ethanol	0.118 ± 0.006	43725 ± 3826	1.224			
2-(Methylthio)-adenosine 5'-diphosphate trisodium salt hydrate	0.093 ± 0.006	48893 ± 5091	1.369			
2-(α-Naphthoyl)-ethyltrimethylammonium iodide	0.080 ± 0.004 **					
2,16,19-Kauranetriol 2-O-β-D-allopyranoside	0.133 ± 0.004	42332 ± 640	1.185			
2,16-Kauranediol	0.131 ± 0.007	25923 ± 854	0.726			
2,16-Kauranediol 2-O-β-D-allopyranoside	0.121 ± 0.019	42593 ± 4582	1.193			
2,2',3'-Trihydroxy-4,6-dimethoxybenzophenone	0.122 ± 0.006	41363 ± 959	1.158			
2,2,5,5-Tetramethylcyclohexane-1,4-dione	0.125 ± 0.001	32749 ± 2754	0.917			
2,24-Dihydroxyursolic acid	0.112 ± 0.027	31850 ± 3584	0.892			
2,2'-Azo-bis-2-aminopropane	0.112 ± 0.007	33765 ± 137	0.945			
2,2'-Bipyridyl	0.100 ± 0.006	36114 ± 2541	1.011			
2',2'-Bisepigallocatechin digallate	0.091 ± 0.007 *					
2,2-Dimethyl-8-prenylchromene 6-carboxylic acid	0.125 ± 0.013	40447 ± 4865	1.132			

2',3' -Dideoxycytidine	0.111 ±0.006	33279 ±1062	0.932			
2,3,2'',3''-Tetrahydroochnaflavone	0.116 ±0.006	49168 ±3687	1.377			
2,3,23-Trihydroxy-12-oleanen-28-oic acid	0.130 ±0.007	47649 ±3035	1.334			
2,3,24-Trihydroxy-12-ursen-28-oic acid	0.114 ±0.008	31519 ±801	0.883			
2,3,24-Trihydroxyolean-12-en-28-oic acid	0.134 ±0.010	28409 ±8033	0.795			
2,3',4,6-Tetrahydroxybenzophenone	0.123 ±0.003	31335 ±3124	0.877			
2,3,4'-Trihydroxy-3',5'-dimethoxypropiophenone	0.119 ±0.007	33487 ±1625	0.938			
2,3,4-Trihydroxy-4'-ethoxybenzophenone	0.100 ±0.005	42334 ±2729	1.185			
2,3,4'-Trihydroxy-4-methoxybenzophenone	0.102 ±0.002	35327 ±2158	0.989			
2',3,5,6',7-Pentahydroxyflavanone	0.128 ±0.005	32446 ±1718	0.908			
2,3,8-Tri-O-methylellagic acid	0.128 ±0.013	40426 ±3801	1.132			
2,3-Bis(3,4-dimethoxybenzyl)-butyrolactone	0.107 ±0.006	29111 ±2419	0.815			
2,3-Butanedione	0.105 ±0.008	30910 ±1355	0.865			
2,3-Butanedione 2-Monoxime	0.092 ±0.004	27725 ±1388	0.776			
2,3-Butanedione monoxime	0.105 ±0.003	41248 ±3678	1.155			
2,3-DCPE hydrochloride	0.115 ±0.001	40257 ±6774	1.127			
2,3-Dehydrokievitone	0.130 ±0.007	43011 ±1995	1.204			
2',3'-Dehydrosalannol	0.131 ±0.004	32777 ±1226	0.918			
2',3'-Didehydro-3'-deoxythymidine	0.104 ±0.008	37086 ±2092	1.038			
2',3'-Dideoxycytidine	0.096 ±0.009	37910 ±1631	1.061			
2',3'-Dideoxyinosine	0.115 ±0.003	46586 ±2163	1.304			
2,3-Dihydroxy-12-ursen-28-oic acid	0.127 ±0.015	44744 ±3882	1.253			
2,3-Dihydroxy-3-(4-hydroxyphenyl)-propanoic acid	0.128 ±0.001	46609 ±4195	1.305			
2',3-Dihydroxy-4,4',6'-trimethoxychalcone	0.110 ±0.012	31886 ±2031	0.893			
2,3-Dihydroxy-4-methoxy-4'-ethoxybenzophenone	0.109 ±0.007	61613 ±5185	1.775			
2,3-Dihydroxy-6,7-dichloroquinoxaline	0.104 ±0.004	27091 ±563	0.759			
2,3-Dihydroxypterodontic acid	0.133 ±0.013	32384 ±2016	0.907			
2,3-Dimercaptosuccinic acid	0.102 ±0.008	30522 ±1468	0.855			
2,3-Dimethoxy-1,4-naphthoquinone	0.097 ±0.008	35353 ±2969	0.990			
2,3-Dinor-6-keto-prostaglandin F1a	0.105 ±0.004	27194 ±1613	0.761			
2,3-Dinor-thromboxane B2	0.109 ±0.003	33794 ±1951	0.946			
2,3-Di-O-methylthiomethyleuscaphic acid	0.125 ±0.003	44949 ±5694	1.259			

2,3-Dioxo-6-nitro-1,2,3,4-tetrahydrobenzo[F]quinoxaline-7-sulfonamide	0.113 ± 0.010	32114 ± 8071	0.899			
2,3-O-Isopropylidenyl euscaphic acid	0.116 ± 0.004	39182 ± 8226	1.097			
2',4',5'-Trimethoxy-2'',2''-dimethylpyrano[5'',6'':6,7]isoflavone	0.108 ± 0.003	34105 ± 2735	0.955			
2,4,5-Trimethoxybenzaldehyde	0.114 ± 0.001	34176 ± 550	0.957			
2,4,6,6-Tetramethyl-3(6H)-pyridinone	0.129 ± 0.001	40958 ± 1773	1.147			
2,4,6-Trimethoxyphenol 1-O-β-D-glucopyranoside	0.123 ± 0.020	39739 ± 4419	1.113			
2,4-Diamino-6-pyrimidinone	0.101 ± 0.005	38250 ± 2832	1.071			
2',4'-Dihydroxy-2,3',6'-trimethoxychalcone	0.118 ± 0.005	38043 ± 2260	1.065			
2',4-Dihydroxy-3,4',6'-trimethoxychalcone	0.102 ± 0.007	42385 ± 902	1.187			
2',4'-Dihydroxy-3',6'-dimethoxychalcone	0.123 ± 0.001	36594 ± 2296	1.025			
2',4'-Dihydroxy-3',6'-dimethoxydihydrochalcone	0.113 ± 0.006	58764 ± 7493	1.645			
2',4'-Dihydroxy-4-Methoxychalcone	0.110 ± 0.003	38002 ± 2855	1.064			
2,4-Dihydroxy-6-methoxyacetophenone	0.114 ± 0.005	49882 ± 3339	1.397			
2',4'-Dihydroxyacetophenone	0.112 ± 0.002	45047 ± 5910	1.261			
2',4-Dihydroxychalcone	0.104 ± 0.007	19723 ± 1684	0.552			
2',4'-Dihydroxychalcone 4'-glucoside	0.118 ± 0.004	21753 ± 2259	0.609			
2,4-Dihydroxyphenylacetic acid	0.118 ± 0.002	40798 ± 3124	1.142			
2,4-Dihydroxyphenylacetyl-L-asparagine	0.106 ± 0.002	33209 ± 5441	0.930			
2,4-Dinitrophenyl 2-fluoro-2-deoxy-β-D-glucopyranoside	0.100 ± 0.009	62991 ± 2674	1.741			
2,4-Pyridinedicarboxylic Acid	0.111 ± 0.012	43943 ± 1307	1.230			
2,5,14-Triacetoxy-3-benzoyloxy-8,15-dihydroxy -7-isobutyroyloxy-9-nicotinoyloxyjatropha-6(17),11E-diene	0.119 ± 0.004	41744 ± 3125	1.169			
2',5,6',7-Tetraacetoxyflavanone	0.121 ± 0.003	36018 ± 5605	1.008			
2',5,6',7-Tetrahydroxyflavanone	0.133 ± 0.009	56364 ± 3474	1.575			
2,5,7,14-Tetraacetoxy-3-benzoyloxy -8,15-dihydroxy-9-nicotinoyloxyjatropha-6(17),11E-diene	0.130 ± 0.010	37680 ± 739	1.055			
2,5,7,8,9,14-Hexaacetoxy-3-benzoyloxy-15-hydroxy-jatropha-6(17),11E-diene	0.120 ± 0.012	40594 ± 884	1.137			
2',5,7-Trihydroxy-8-methoxyflavanone	0.119 ± 0.006	21548 ± 1346	0.603			
2,5,9,14-Tetraacetoxy-3-benzoyloxy -8,15-dihydroxy-7-isobutyroyloxyjatropha-6(17),11E-diene	0.118 ± 0.016	41058 ± 2834	1.150			
2',5'-Dihydroxy-4-methoxychalcone	0.104 ± 0.001	31346 ± 551	0.878			
2,5-Dihydroxybenzaldehyde	0.124 ± 0.003	34323 ± 808	0.961			
2,5-Di-t-butyl-4-Hydroxyanisole	0.108 ± 0.011	43755 ± 668	1.225			

2,5-Ditertbutylhydroquinone	0.110 ± 0.001	22663 ± 1746	0.635			
2,6,16-Kauranetriol	0.133 ± 0.001	35377 ± 2419	0.991			
2,6,16-Kauranetriol 2-O-β-D-allopyranoside	0.118 ± 0.005	28749 ± 2011	0.805			
2',6',7-Triacetoxy-5-hydroxyflavanone	0.128 ± 0.003	38945 ± 235	1.090			
2,6-Difluoro-4-[2-(phenylsulfonylamino)-ethylthio]phenoxyacetamide	0.099 ± 0.009	39167 ± 1750	1.097			
2,6-dihydroxy-4-methoxytoluene	0.118 ± 0.005	32202 ± 4171	0.902			
2,6-Dimethoxy-1,4-benzoquinone	0.128 ± 0.002	33006 ± 1112	0.924			
2,6-Dimethoxy-1-acetonylquinol	0.124 ± 0.014	42411 ± 3374	1.187			
2,6-Dimethoxybenzoic acid	0.122 ± 0.003	36952 ± 900	1.035			
2,6-Dimethoxyquinone	0.107 ± 0.004	37170 ± 4038	1.041			
2,6-Dimethyl-3,7-octadiene-2,6-diol	0.119 ± 0.004	32553 ± 2256	0.911			
2,6-Dimethyl-7-octene-2,3,6-triol	0.127 ± 0.008	30517 ± 2403	0.854			
2,6-Di-O-acetylisohyenanchin	0.130 ± 0.008	46062 ± 6965	1.290			
2,7-Dihydrohomoerysotrine	0.111 ± 0.006	33541 ± 2428	0.939			
2,7-Dihydroxy-2H-1,4-benzoxazin-3(4H)-one	0.115 ± 0.004	39405 ± 4199	1.103			
2-[(4-Phenylpiperazin-1-yl)-methyl] -2,3-dihydroimidazo[1,2-c]quinazolin-5(6H)-one	0.101 ± 0.002	71936 ± 8140	1.924			
2-[[4-(2-Methoxyphenol)-piperazin-1-yl]methyl]- 6-methyl-2,3-dihydroimidazo[1,2C]quinazolin-5(6H)-one	0.103 ± 0.006	60475 ± 2672	1.617			
2-[[b-(4-Hydroxyphenol)-ethyl]aminoethyl]-1-tetralone	0.126 ± 0.006	37126 ± 3720	1.040			
2-[1-(4-Piperonyl)-piperazinyl]benzothiazole	0.106 ± 0.002	30767 ± 1570	0.861			
20(29)-Lupene-3,23-diol	0.130 ± 0.012	30737 ± 1528	0.861			
20,24-Dihydroxydammar-25-en-3-one	0.118 ± 0.004	34628 ± 3013	0.970			
20,24-Epoxy-24-methoxy-23(24-25)-abeo-dammaran-3-one	0.117 ± 0.007	42855 ± 1497	1.200			
20-Carboxy-leukotriene B4	0.108 ± 0.001	26391 ± 1656	0.739			
20-Hydroxy-3-oxo-28-lupanoic acid	0.119 ± 0.005	41178 ± 3118	1.153			
20-Hydroxyecdysone	0.118 ± 0.019	42451 ± 3491	1.189			
20-Hydroxy-leukotriene B4	0.104 ± 0.01	28292 ± 2038	0.792			
20S,24R-Epoxy-dammar-12,25-diol-3-one	0.107 ± 0.007	31613 ± 568	0.885			
21,24-Epoxycycloartane-3,25-diol	0.125 ± 0.001	35197 ± 959	0.985			
21-Acetoxypregnenolone	0.101 ± 0.011	42369 ± 484	1.186			
21-Deoxyneridienone B	0.129 ± 0.002	39250 ± 3519	1.099			
21-Episerratenediol	0.129 ± 0.003	35219 ± 2080	0.986			

21-Episerratriol	0.120 ±0.012	37868 ±5287	1.060			
22-Dehydroclerosterol	0.125 ±0.005	40014 ±7295	1.120			
22-Dehydroclerosterol glucoside	0.117 ±0.001	29587 ±1243	0.828			
22-Dehydroclerosteryl acetate	0.105 ±0.013	34543 ±302	0.967			
22-Hydroxy-3-oxo-12-ursen-30-oic acid	0.115 ±0.005	36511 ±1636	1.022			
22-Hydroxy-3-oxoolean-12-en-29-oic acid	0.122 ±0.001	46621 ±1845	1.305			
23-Deoxojessic acid	0.127 ±0.006	31897 ±3042	0.893			
23-Nor-3-oxo-12-oleanen-28-oic acid	0.119 ±0.002	40012 ±2305	1.120			
24(S),25-Epoxycholesterol	0.113 ±0.006	47083 ±3513	1.318			
24(S)-Hydroxycholesterol	0.118 ±0.001	37512 ±5418	1.050			
24,25-Dihydroxycycloartan-3-one	0.117 ±0.006	37438 ±5352	1.048			
24,25-Dihydroxydammar-20-en-3-one	0.133 ±0.014	37901 ±2124	1.061			
24,25-Dihydroxyvitamin D3	0.113 ±0.002	33631 ±2117	0.942			
24,25-Epoxy-dammar-20(21)-en-3-one	0.113 ±0.011	34698 ±635	0.972			
24,25-Epoxytirucall-7-en-3,23-dione	0.124 ±0.004	39424 ±902	1.104			
24-Hydroxy-25-ethoxy-3,4-seco-cycloart-4(28)-en-3-oic acid methyl ester	0.130 ±0.009	37419 ±451	1.048			
24-Methylenecycloartan-3-ol	0.121 ±0.003	36772 ±3190	1.030			
24-Methylenecycloartanol acetate	0.129 ±0.004	25444 ±1533	0.712			
25-Ethoxy-24-oxo-3,4-secocycloart -4(28)-en-3-oic acid methyl ester	0.129 ±0.001	40331 ±776	1.129			
25-Hydroxycycloart-23-en-3-one	0.110 ±0.007	51393 ±3208	1.439			
25-Hydroxyvitamin D3	0.120 ±0.004	45580 ±2519	1.276			
26-Nor-8-oxo-α-onocerin	0.126 ±0.008	45075 ±5811	1.262			
27200-12-0	0.107 ±0.012	29100 ±1918	0.815			
27-p-Coumaroyloxyursolic acid	0.128 ±0.011	29745 ±1473	0.833			
29-Hydroxyfriedelan-3-one	0.114 ±0.018	37463 ±4796	1.049			
29-Norcycloart-23-ene-3,25-diol	0.127 ±0.004	33609 ±741	0.941			
2-Acetylbenzoic acid	0.116 ±0.009	38555 ±4017	1.080			
2-Acetylpyrrole	0.104 ±0.019	62016 ±6002	1.786			
2-Amino-4-(3-hydroxy-5-methylisoxazol-4-yl)-butyric acid	0.103 ±0.003	33207 ±3333	0.930			
2-Aminobenzenesulfonamide	0.117 ±0.009	44380 ±6855	1.243			
2-Aminopurine	0.123 ±0.002	35640 ±1298	0.998			

2-APB	0.079 ± 0.002 **					
2-Arachidonoylglycerol	0.100 ± 0.006	33338 ± 5440	0.933			
2-Benzoyl-5-methoxybenzoquinone	0.113 ± 0.004	32038 ± 598	0.897			
2-BFI hydrochloride	0.109 ± 0.002	23563 ± 1192	0.660			
2-Caren-10-ol	0.126 ± 0.002	47061 ± 1454	1.318			
2-Chloro-11-(4-methylpiperazino)-dibenz[B,F]oxepin maleate	0.129 ± 0.003 *					
2-Chloro-2'-Deoxyadenosine	0.110 ± 0.003	20841 ± 2394	0.584			
2-Chloro-2-deoxy-D-glucose	0.098 ± 0.006	40230 ± 2293	1.126			
2-Chloroadenosine	0.075 ± 0.002 **					
2-Chloroadenosine triphosphate tetrasodium	0.072 ± 0.009 **					
2-Chloro-N6-cyclopentyladenosine	0.103 ± 0.008	45759 ± 4198	1.281			
2-Chloropyrazine	0.110 ± 0.014	48437 ± 2224	1.356			
2-Cl-IB-MECA	0.106 ± 0.007	29377 ± 732	0.823			
2-CMDO	0.121 ± 0.009	46877 ± 5020	1.313			
2-Cyclooctyl-2-hydroxyethylamine hydrochloride	0.100 ± 0.015	36739 ± 2872	1.029			
2-DCHA-PAF	0.100 ± 0.007	36860 ± 1813	1.032			
2-Deacetoxytaxinine B	0.120 ± 0.010	50810 ± 2865	1.423			
2-Desoxy-4-epi-pulchellin	0.120 ± 0.006	25405 ± 1893	0.711			
2-DHLA-PAF	0.103 ± 0.001	39079 ± 577	1.094			
2-EPA-PAF	0.112 ± 0.003	49522 ± 1434	1.528			
2-Epitormentic acid	0.132 ± 0.006	48653 ± 6033	1.362			
2-Fluoropalmitic acid	0.106 ± 0.004	33033 ± 2129	0.925			
2H-Indol-2-one, 1,3-dihydro-1-phenyl-3,3-bis(4-pyridinylmethyl)	0.106 ± 0.003	67060 ± 6539	1.878			
2-Hydroxy-1,8-cineole	0.118 ± 0.003	24767 ± 1424	0.693			
2-hydroxy-3,4-dimethoxybenzoic acid	0.098 ± 0.005	36833 ± 2741	1.031			
2-hydroxy-5 (6)-epoxy-tetrahydrocaryophyllene	0.108 ± 0.009	35268 ± 3140	0.987			
2'-Hydroxy-5'-methoxyacetophenone	0.113 ± 0.001	30042 ± 1791	0.841			
2-Hydroxy-7-O-methylscillascillin	0.117 ± 0.004	51065 ± 1497	1.430			
2'-Hydroxydaidzein	0.123 ± 0.002	50299 ± 3012	1.408			
2'-Hydroxygenistein	0.132 ± 0.001	56876 ± 6776	1.592			
2-Hydroxymyristic acid	0.104 ± 0.006	36540 ± 683	1.023			
2-Hydroxysaclofen	0.099 ± 0.003	29886 ± 1717	0.837			

2-Hydroxytetracosanoic acid ethyl ester	0.111 ± 0.010	30774 ± 1516	0.862			
2-Iodomelatonin	0.102 ± 0.010	42290 ± 8177	1.184			
2-Iodo-N-Butanoyl-5-methoxytryptamine	0.102 ± 0.013	44989 ± 8694	1.260			
2'-MeCCPA	0.103 ± 0.010	36053 ± 1517	1.009			
2-mercaptobenzothiazole	0.077 ± 0.002 **					
2-Methoxy-5 (6)-epoxy-tetrahydrocaryophyllene	0.107 ± 0.008	40145 ± 3231	1.124			
2-Methoxyantimycin A3	0.114 ± 0.004	37891 ± 2438	1.061			
2-Methoxyestradiol	0.108 ± 0.010	41443 ± 6681	1.160			
2'-Methoxyformonetin	0.121 ± 0.003	40273 ± 1942	1.128			
2-Methoxyresorcinol	0.100 ± 0.006	33662 ± 1659	0.943			
2-Methoxystypandrone	0.118 ± 0.006	19450 ± 1952	0.544			
2-Methyl gramine	0.107 ± 0.002	18111 ± 1582	0.522			
2-Methyl-4-(piperidin-1-ylcarboxy)-5-isopropylphenyltrimethylammonium chloride	0.111 ± 0.004	41506 ± 367	1.162			
2-Methyl-5,7,8-trimethoxyisoflavone	0.105 ± 0.002	40362 ± 4678	1.130			
2-Methyl-5-hydroxytryptamine hydrochloride	0.106 ± 0.002	31259 ± 1514	0.875			
2-Methyl-5-hydroxytryptamine maleate	0.100 ± 0.006	41182 ± 2822	1.153			
2-Methyl-6-(phenylethynyl)-pyridine	0.110 ± 0.008	31081 ± 2195	0.870			
2-Methylbutane-1,2,3,4-tetrol	0.126 ± 0.008	30093 ± 2086	0.843			
2-Methylene-5-(2,5-dioxotetrahydrofuran-3-yl)<br>-6-oxo-10,10-dimethylbicyclo[7: 2: 0]undecane	0.104 ± 0.006	29232 ± 2489	0.818			
2-Methylserotonin hydrochloride	0.101 ± 0.007	33429 ± 5444	0.936			
2-Methylthioadenosine triphosphate tetrasodium	0.101 ± 0.003	42197 ± 2589	1.181			
2-O-Acetyltutin	0.126 ± 0.006	26046 ± 802	0.729			
2'-O-Methylbroussonin C	0.127 ± 0.012	45255 ± 3957	1.267			
2'-O-Methylisoliquiritigenin	0.117 ± 0.001	44676 ± 2781	1.251			
2'-O-Methylperlatolic acid	0.117 ± 0.001	37079 ± 688	1.038			
2-Oxopomolic acid	0.120 ± 0.004	40939 ± 3582	1.146			
2-Phenylaminoadenosine	0.105 ± 0.007	39822 ± 1349	1.115			
2-Phenylmelatonin	0.100 ± 0.004	40310 ± 7639	1.129			
2-PMDQ	0.125 ± 0.009	23472 ± 1585	0.657			
2-Pyridylethylamine dihydrochloride	0.114 ± 0.003	19301 ± 914	0.534			
2-TEDC	0.107 ± 0.006	26966 ± 1464	0.755			

2-Thiouracil	0.112 ± 0.004	18145 ± 1433	0.508			
2-α-Hydroxy pterodontic acid methyl ester	0.120 ± 0.000	30522 ± 1988	0.855			
3 α-Bis-(4-fluorophenyl)-methoxytropane hydrochloride	0.117 ± 0.003	24982 ± 2741	0.699			
3-(1H-Imidazol-4-yl)-propyl di(p-fluorophenyl)-methyl ether hydrochloride	0.104 ± 0.005	36191 ± 4440	1.013			
3-(2,4-Dihydroxyphenyl)-propionic acid	0.117 ± 0.003	43957 ± 6450	1.231			
3(2H)-Pyridazinone, 6-[4-(difluoromethoxy)-3-methoxyphenyl]	0.122 ± 0.008	32135 ± 1499	0.900			
3-(2-Hydroxyphenyl)-2-propenal	0.107 ± 0.006	29263 ± 1750	0.819			
3-(3-Hydroxy-3-methylbutanyl)-2,4,6-trihydroxybenzophenone	0.115 ± 0.005	50331 ± 1926	1.409			
3-(4-Allylpiperazin-1-yl)-2-quinoxalinecarbonitrile maleate	0.105 ± 0.007	39264 ± 5597	1.099			
3-(4-Hydroxy-3,5-dimethoxyphenyl)-1,2-propanediol	0.118 ± 0.006	40234 ± 3127	1.127			
3-(4-Hydroxy-3-methoxyphenyl)-propyl tetracosanoate	0.118 ± 0.004	42354 ± 2468	1.186			
3-(4-Hydroxyphenyl)-1-propanol	0.119 ± 0.002	44715 ± 2070	1.252			
3-(Hydroxymethyl)-cyclopentanol	0.133 ± 0.013	35805 ± 326	1.003			
3-(Hydroxymethyl)-cyclopentanone	0.121 ± 0.005	30981 ± 2177	0.867			
3,10-Dihydroxy-5,11-dielmenthadiene-4,9-dione	0.117 ± 0.006	37299 ± 4797	1.044			
3,19-Dihydroxy-6,23-dioxo-12-ursen-28-oic acid	0.128 ± 0.004	33170 ± 5326	0.929			
3,21-Dihydroxy-14-serraten-16-one	0.122 ± 0.003	31891 ± 1230	0.893			
3,22-Dihydroxyolean-12-en-29-oic acid	0.127 ± 0.002	33356 ± 3408	0.934			
3,23-dioxo-9,19-Cyclolanost-24-en-26-oic acid	0.115 ± 0.002	34672 ± 3217	0.971			
3,27-Dihydroxy-20(29)-lupen-28-oic acid methyl ester	0.128 ± 0.007	29865 ± 1686	0.836			
3,3',4',5,5',7-Hexahydroxyflavan	0.130 ± 0.015	34461 ± 1946	0.965			
3,3'-Diindolylmethane	0.097 ± 0.007	21298 ± 483	0.596			
3,3'-Di-O-methylellagic acid	0.125 ± 0.014	44985 ± 8445	1.260			
3,4,4',7-Tetrahydroxyflavan	0.116 ± 0.006	51335 ± 4484	1.437			
3',4',5',3,5,6,7-Heptamethoxyflavone	0.138 ± 0.007	36550 ± 3588	1.023			
3',4',5',3,5,7,8-Heptamethoxyflavone	0.119 ± 0.003	34934 ± 2116	0.978			
3,4',5,6,7-pentamethoxyflavone	0.111 ± 0.015	35278 ± 930	0.988			
3,4,5-Trimethoxycinnamyl alcohol	0.112 ± 0.003	38140 ± 3046	1.068			
3',4',7-Trimethoxyflavan	0.108 ± 0.009	51107 ± 2398	1.431			
3,4-Diacetoxycinnamamide	0.117 ± 0.005	40177 ± 3112	1.125			
3,4-Dichloroisocoumarin	0.121 ± 0.001	30833 ± 677	0.863			
3,4-didesmethyl-5-deshydroxy-3'-ethoxyscleroin	0.101 ± 0.008	17423 ± 581	0.502			

3,4-Dihydro-2,2-dimethyl-2H-naphtho[1,2-b]pyran	0.115 ± 0.005	38135 ± 2191	1.068			
3,4-Dihydro-3,4-dihydroxynaphthalen-1(2H)-one	0.114 ± 0.003	45407 ± 3434	1.271			
3,4'-Dihydroxy-3',5,7-trimethoxyflavan	0.119 ± 0.008	33993 ± 2900	0.952			
3,4'-Dihydroxy-3',5'-dimethoxypropiophenone	0.125 ± 0.005	27234 ± 2378	0.763			
3,4-Dihydroxybenzaldehyde	0.116 ± 0.007	30321 ± 1225	0.849			
3,4-Dihydroxybenzoic acid	0.133 ± 0.002	22058 ± 1268	0.618			
3,4-Dihydroxycinnamamide	0.121 ± 0.003	43830 ± 1037	1.227			
3,4-Dihydroxyphenylacetic acid	0.094 ± 0.005	34086 ± 2687	0.954			
3,4-Dimethoxybenzoic acid	0.131 ± 0.012	25593 ± 561	0.717			
3,4-Dimethoxycinnamic acid	0.093 ± 0.009	31196 ± 2094	0.873			
3,4-Dimethoxydalbergione	0.101 ± 0.007	32419 ± 2039	0.908			
3,4'-Dimethoxyflavone	0.108 ± 0.002	30069 ± 3239	0.842			
3,4-Dimethoxyphenol	0.117 ± 0.008	33526 ± 1299	0.939			
3,4-Dimethoxyphenyl β-D-glucoside	0.123 ± 0.015	32279 ± 4496	0.904			
3,4-O-Isopropylidene shikimic acid	0.123 ± 0.002	37887 ± 3428	1.061			
3,4-O-Isopropylidene-2-methylbutane-1,2,3,4-tetrol	0.121 ± 0.017	36025 ± 3229	1.009			
3,4-Secocucurbita-4,24-diene-3,26,29-trioic acid	0.124 ± 0.005	28529 ± 1709	0.799			
3,4-Secotirucalla-4(28),7,24-triene-3,26-dioic acid	0.118 ± 0.004	38929 ± 3201	1.090			
3',5,5',7-Tetraacetoxyflavanone	0.122 ± 0.002	32546 ± 2235	0.911			
3',5,5',7-Tetrahydroxy-4',6-dimethoxyflavone	0.126 ± 0.001	36888 ± 2655	1.033			
3',5,5',7-Tetrahydroxyflavanone	0.108 ± 0.002	35085 ± 3362	0.982			
3,5,9-Trihydroxyergosta-7,22-dien-6-one	0.123 ± 0.007	33789 ± 2885	0.946			
3,5-Cycloergosta-6,8(14),22-triene	0.123 ± 0.003	33098 ± 3741	0.927			
3',5-Dihydroxy-4',5',6,7-tetramethoxyflavone	0.121 ± 0.004	31286 ± 2065	0.876			
3,5-Dihydroxy-4',7-dimethoxyflavone	0.123 ± 0.005	34169 ± 1902	0.957			
3,5-Dihydroxyergosta-7,22-dien-6-one	0.133 ± 0.010	30966 ± 4181	0.867			
3,5-Diiodo-4-hydroxyphenylpropionic acid	0.117 ± 0.005	38349 ± 641	1.074			
3,5-Diiodo-L-thyronine	0.127 ± 0.005	43232 ± 4255	1.210			
3,5-Diiodo-L-tyrosine dihydrate	0.115 ± 0.003	49394 ± 3814	1.383			
3,5-Dinitrocatechol	0.089 ± 0.004 *					
3,5-Diprenyl-4-hydroxybenzaldehyde	0.121 ± 0.005	36241 ± 2383	1.015			

3,6,19,23-Tetrahydroxy-12-ursen-28-oic acid	0.119 ±0.007	28961 ±1266	0.811			
3,6,19-Trihydroxy-23-oxo-12-ursen-28-oic acid	0.126 ±0.009	38361 ±2387	1.074			
3,6-Caryolanediol	0.124 ±0.001	36298 ±5867	1.016			
3,6-Dimethoxyflavone	0.101 ±0.007	38287 ±3389	1.072			
3,7,16-Trihydroxystigmast-5-ene	0.112 ±0.011	38841 ±4000	1.088			
3,7-Dihydroxyflavone	0.116 ±0.002	40457 ±4410	1.133			
3,7-Dimethoxyflavone	0.102 ±0.008	24228 ±912	0.678			
3,7-Dimethyl-1-propargylxanthine	0.094 ±0.005	35170 ±764	0.985			
3,7-Epoxycaryophyllan-6-ol	0.105 ±0.007	44161 ±3229	1.236			
3,7-Epoxycaryophyllan-6-one	0.105 ±0.005	48842 ±4557	1.368			
3,7-O-Diacetylpinobanksin	0.122 ±0.005	33014 ±4175	0.924			
3,8''-Biapigenin	0.120 ±0.005	44592 ±2832	1.249			
3,9-Dihydroxypterocarpan	0.125 ±0.004	65398 ±4317	1.828			
3-[2-[4-(2-Methoxyphenyl)-piperazin-1-yl]ethyl]pyrimido[5,4-B]indole-2,4-dione	0.110 ±0.008	57341 ±3026	1.533			
3-[2-[4-(2-Methoxyphenyl)-piperazin-1-yl]pyrimido[5,4-B]indole-2,4-dione	0.110 ±0.006	50058 ±5965	1.402			
3-[3,5-Dibromo-4-hydroxybenzoyl]-2-ethylbenzofuran	0.129 ±0.004	53213 ±1441	1.482			
30-Hydroxylup-20(29)-en-3-one	0.135 ±0.008	34797 ±2326	0.974			
3a, 5a-Androstanol	0.113 ±0.006	43815 ±3314	1.227			
3a, 5a-Androstenol	0.112 ±0.003	51373 ±2500	1.438			
3-acetamidocoumarin	0.105 ±0.015	36319 ±3356	1.017			
3-Acetoxy-11-ursen-28,13-olide	0.114 ±0.005	36097 ±2043	1.011			
3-Acetoxy-24-hydroxydammara-20,25-diene	0.111 ±0.002	38421 ±1831	1.076			
3-Acetoxy-27-hydroxy-20(29)-lupen-28-oic acid methyl ester	0.124 ±0.006	35786 ±3585	1.002			
3-Acetoxy-4,7(11)-cadinadien-8-one	0.120 ±0.002	36045 ±2446	1.009			
3-Acetoxy-4-cadinen-8-one	0.118 ±0.006	41053 ±2842	1.149			
3-Acetoxy-8(17),13E-labdadien-15-oic acid	0.123 ±0.002	38309 ±2927	1.073			
3-Acetylgedunol	0.114 ±0.004	30177 ±3150	0.845			
3-Amino-1,2,4-triazole	0.090 ±0.003 **					
3-Amino-1-propanesulfonic acid sodium	0.097 ±0.002	42204 ±5311	1.182			
3-Aminobenzamide	0.099 ±0.001	49745 ±6748	1.393			
3-Aminopropionitrile fumarate	0.099 ±0.001	36636 ±309	1.026			
3-Aminopropylphosphonic acid	0.098 ±0.006	31850 ±901	0.892			

3-Amino-β-pinene	0.106 ± 0.006	37489 ± 3490	1.050			
3-AQC	0.107 ± 0.001	40399 ± 4535	1.131			
3'-Azido-3'-deoxythymidine	0.098 ± 0.002	36384 ± 1528	1.019			
3-Bromo-7-nitroindazole	0.100 ± 0.002	34579 ± 1493	0.968			
3-CPMT	0.114 ± 0.005	19356 ± 3274	0.542			
3-Deazaadenosine	0.100 ± 0.002	31821 ± 4810	0.891			
3-Dehydro-15-deoxoeucosterol	0.118 ± 0.002	49612 ± 1750	1.389			
3-Deoxo-3-β-hydroxymexicanolide 16-enol ether	0.114 ± 0.004	32860 ± 1052	0.920			
3-Deoxy-3-β-hydroxyangolensic acid methyl ester	0.111 ± 0.005	30206 ± 1616	0.846			
3'-Deoxydenosine	0.119 ± 0.007	35844 ± 4347	1.004			
3-Deoxysappanone B	0.111 ± 0.004	42850 ± 3399	1.200			
3-Deshydroxysappanol trimethyl ether	0.108 ± 0.007	37135 ± 4975	1.040			
3-Epicabraleadiol	0.125 ± 0.004	37654 ± 3241	1.054			
3-Epicabraleahydroxylactone	0.124 ± 0.007	50342 ± 5396	1.410			
3-Epiglochidiol	0.114 ± 0.006	38910 ± 2341	1.089			
3-Epiglochidiol diacetate	0.125 ± 0.003	34063 ± 2768	0.954			
3-Epikatonic acid	0.117 ± 0.003	41262 ± 5846	1.155			
3-Epiturraeanthin	0.119 ± 0.001	35290 ± 5263	0.988			
3-Epiwightianol A tetraacetate	0.122 ± 0.006	34936 ± 1517	0.978			
3-Epiwilsonine	0.117 ± 0.002	34234 ± 1944	0.959			
3'-Fluorobenzylspiperone maleate	0.074 ± 0.001 **					
3-Formylrifamycin	0.103 ± 0.003	20659 ± 1520	0.578			
3-Furfuryl 2-pyrrolecarboxylate	0.114 ± 0.008	36545 ± 5514	1.023			
3-Geranyl-4-methoxybenzoic acid	0.126 ± 0.003	37185 ± 1798	1.041			
3h-1,2-dithiole-3-thione	0.105 ± 0.006	29063 ± 4419	0.814			
3-Hydroxy-1,2-dimethyl-4(1H)-pyridone	0.107 ± 0.008	35101 ± 5776	0.983			
3-Hydroxy-11-ursen-28,13-olide	0.104 ± 0.016	27318 ± 3133	0.765			
3-Hydroxy-12-oleanene-23,28-dioic acid	0.121 ± 0.002	37629 ± 3062	1.054			
3'-Hydroxy-3,9-dihydroeucomin	0.116 ± 0.005	62312 ± 683	1.742			
3-Hydroxy-4-(succin-2-yl)-caryolane δ-lactone	0.104 ± 0.010	36519 ± 2161	1.023			
3-Hydroxy-4,15-dinor-1(5)-xanthen-12,8-olide	0.128 ± 0.003	35492 ± 5939	0.994			
3-Hydroxy-4',5,7-trimethoxyflavanone	0.112 ± 0.025	22147 ± 870	0.620			

3-Hydroxy-4-methoxy-benzenepropanol	0.118 ± 0.005	50046 ± 1531	1.401			
3-Hydroxy-4-methoxyphenethylamine	0.114 ± 0.010	34893 ± 2822	0.977			
3-Hydroxy-5,7-dimethoxy-3',4'-methylenedioxyflavan	0.118 ± 0.003	43023 ± 1841	1.205			
3-Hydroxycatalponol	0.118 ± 0.006	34289 ± 2169	0.960			
3-Hydroxyfenazepam	0.108 ± 0.009	35523 ± 1279	0.995			
3-Hydroxyflavone	0.101 ± 0.013	27634 ± 1412	0.774			
3-Hydroxylanost-9(11),24-dien-26-oic acid	0.122 ± 0.011	36729 ± 2085	1.028			
3-Hydroxymethyl-β-carboline	0.107 ± 0.004	39312 ± 4808	1.101			
3-Hydroxyphenethylamine hydrochloride	0.110 ± 0.003	26088 ± 2180	0.730			
3'-Hydroxyrocaglamide	0.130 ± 0.012	34213 ± 3571	0.958			
3-Hydroxysarpagine	0.132 ± 0.005	29887 ± 1061	0.837			
3-Hydroxytyramine	0.112 ± 0.008	29011 ± 2420	0.812			
3-Iodo-L-tyrosine	0.102 ± 0.005	36430 ± 4864	1.020			
3-Isobutyl-1-methylxanthine	0.109 ± 0.002	44437 ± 2417	1.244			
3-Isomangostin hydrate	0.116 ± 0.007	43363 ± 4017	1.214			
3-Isomangostin hydrate formate	0.119 ± 0.006	41079 ± 3592	1.150			
3-Matida	0.108 ± 0.004	30957 ± 5621	0.867			
3-Methoxy-4,5-methylenedioxycinnamaldehyde	0.118 ± 0.003	38378 ± 2545	1.075			
3-Methoxy-5-heneicosylphenol	0.121 ± 0.004	31833 ± 488	0.891			
3-Methoxycatechol	0.102 ± 0.003	19897 ± 1515	0.573			
3-Methoxyfuran	0.123 ± 0.003	31649 ± 2783	0.886			
3-Methoxy-L-tyrosine	0.115 ± 0.008	34575 ± 774	0.968			
3-Methoxy-morphanin hydrochloride	0.104 ± 0.007	26602 ± 1776	0.745			
3'-Methoxyrocaglamide	0.131 ± 0.015	42136 ± 1899	1.180			
3-Methoxytyramine	0.113 ± 0.013	32331 ± 4074	0.905			
3-Methylcholanthrene	0.119 ± 0.003	30895 ± 5525	0.865			
3-Methyl-GABA	0.099 ± 0.011	19516 ± 665	0.522			
3-Methylorsellinic acid	0.112 ± 0.007	22812 ± 2406	0.639			
3-Morpholinosydnonimine hydrochloride	0.106 ± 0.030	41144 ± 1901	1.152			
3-MPPI	0.112 ± 0.007	30967 ± 2772	0.867			
3-Nitropropionic acid	0.100 ± 0.004	51720 ± 4372	1.430			

3-Nor-3-oxopanasinsan-6-ol	0.119 ± 0.011	30212 ± 2596	0.846			
3-n-Propylxanthine	0.097 ± 0.005	31161 ± 3375	0.872			
3-O-(E)-p-Coumaroylbetulin	0.121 ± 0.008	33606 ± 1795	0.941			
3-O-Acetyloleanderolide	0.128 ± 0.006	35332 ± 2739	0.989			
3-O-Acetyloleanolic acid	0.120 ± 0.006	38875 ± 1170	1.088			
3-O-Acetylpinobanksin	0.114 ± 0.003	23345 ± 539	0.654			
3-O-Methylducheside A	0.115 ± 0.005	23880 ± 2939	0.669			
3'-O-Methylorobol	0.124 ± 0.004	70141 ± 9028	1.960			
3-O-Methylquercetin	0.115 ± 0.002	41287 ± 4045	1.156			
3-O-Methylquercetin tetraacetate	0.137 ± 0.001	28866 ± 417	0.808			
3-O-Methyltagitinin F	0.127 ± 0.007	21386 ± 3745	0.599			
3-O-Methyltirotundin	0.129 ± 0.007	29499 ± 3912	0.826			
3-Oxo-24,25,26,27-tetranortirucall-7-en-23,21-olide	0.121 ± 0.002	32743 ± 2597	0.917			
3-Oxoursan (28-13)-olide	0.101 ± 0.002	35545 ± 3163	0.995			
3-Phenyl-2-propen-1-ol	0.121 ± 0.005	29179 ± 1425	0.817			
3-Phenylsydnone	0.111 ± 0.007	35752 ± 4716	1.001			
3-Prenyl-2,4,6-trihydroxybenzophenone	0.102 ± 0.002	30768 ± 2172	0.861			
3-Pyridinemethanol	0.127 ± 0.007	43037 ± 3799	1.205			
3-Tropanyl-3,5-dichlorobenzoate	0.107 ± 0.003	25344 ± 1359	0.710			
3-Tropanyl-indole-3-carboxylate hydrochloride	0.112 ± 0.001	31274 ± 1923	0.876			
3-Tropanylindole-3-carboxylate methiodide	0.112 ± 0.003	38400 ± 1796	1.075			
3-α,21-Dihydroxy-5-α-pregnan-20-one	0.093 ± 0.006	33932 ± 3467	0.950			
3-α-[(4-Chlorophenyl)-phenylmethoxy]tropane hydrochloride	0.076 ± 0.007 **					
3-α-Acetoxydihydrodeoxygedunin	0.113 ± 0.001	33167 ± 1113	0.929			
3-α-Akebonoic acid	0.118 ± 0.004	45182 ± 6672	1.265			
3-α-bis-(4-Fluorophenyl)-methoxytropane hydrochloride	0.119 ± 0.006	23243 ± 2403	0.651			
3-α-hydroxy-3-deoxyangolensic acid methyl ester	0.105 ± 0.005	34483 ± 1858	0.966			
3-α-hydroxy-4,4-bisnor-8,11,13-podocarpatriene	0.106 ± 0.002	24917 ± 5044	0.698			
3-α-Hydroxy-5-β-androstan-17-one	0.108 ± 0.007	37773 ± 1388	1.058			
3-β-hydroxy-23,24-bisnorchol-5-enic acid	0.103 ± 0.005	33644 ± 4265	0.942			
3-β-hydroxydeoxodihydrodeoxygedunin	0.113 ± 0.007	36442 ± 3327	1.020			
3-β-Hydroxyergost-5-en-7-one	0.130 ± 0.002	31369 ± 1714	0.878			

3-β-Hydroxyporiferast-5-en-7-one	0.131 ± 0.012	30904 ± 507	0.865			
4(15),11-Oppositadien-1-ol	0.116 ± 0.007	35489 ± 2192	0.994			
4(15),5,10(14)-Germacratrien-1-ol	0.126 ± 0.003	40256 ± 969	1.127			
4(15)-Oppositene-1,7-diol	0.114 ± 0.002	44689 ± 2234	1.251			
4-(2-Aminoethyl)-benzenesulfonyl fluoride hydrochloride	0.107 ± 0.002	35166 ± 2708	0.985			
4-(2-Hydroxy-1-methoxyethyl)-1,2-benzenediol	0.116 ± 0.002	35130 ± 636	0.984			
4-(3,4-Dihydroxyphenyl)-2-butanone	0.121 ± 0.001	46737 ± 2459	1.309			
4-(3,4-Dimethoxyphenyl)-3-buten-1-ol	0.124 ± 0.004	48923 ± 6994	1.370			
4-(3,4-Dimethoxyphenyl)-3-butene-1,2-diol	0.121 ± 0.002	46467 ± 2320	1.301			
4-(3-Butoxy-4-methoxybenzyl)-imidazolidin-2-one	0.101 ± 0.004	34751 ± 860	0.973			
4-(4-Fluorobenzoyl)-1-(4-phenylbutyl)-piperidine oxalate	0.119 ± 0.008	34619 ± 3835	0.969			
4-(Methyl-nitrosamino)-1-(3-pyridyl)-1-butanone	0.128 ± 0.004 *					
4(Z),7(Z),10(Z),13(Z),16(Z),19(Z)-Docosahexaenoic acid	0.096 ± 0.006 *					
4,10-Aromadendranediol	0.118 ± 0.001	44728 ± 816	1.252			
4,15-Dihydroxy-18-nor-8,11,13-abietatrien-7-one	0.117 ± 0.002	38943 ± 602	1.090			
4,4'-Diisothiocyanostilbene-2,2'-sufonic acid sodium salt	0.104 ± 0.022	42416 ± 1930	1.188			
4,4'-dimethoxydalbergione	0.103 ± 0.003	38522 ± 1153	1.079			
4',4'''-Di-O-methylcupressuflavone	0.138 ± 0.013	33576 ± 3010	0.940			
4,4-Pentamethylenepiperidine hydrochloride	0.114 ± 0.005	35611 ± 555	0.997			
4,5,6,7-Tetrahydroisoxazolo[5,4-C]pyridin-3-ol	0.106 ± 0.005	28606 ± 2955	0.801			
4',5,7-Trihydroxy-6-prenylflavone	0.124 ± 0.003	40812 ± 2393	1.143			
4,5-Dihydroblumenol A	0.120 ± 0.004	58389 ± 4055	1.632			
4',5-Dihydroxy-3',5',6,7-tetramethoxyflavone	0.119 ± 0.003	37914 ± 2168	1.062			
4',5-Dihydroxyflavone	0.136 ± 0.001	33246 ± 2709	0.931			
4,5-Dimethoxycanthin-6-one	0.130 ± 0.009	33067 ± 3077	0.926			
4,5-Epoxyartemisinic acid	0.115 ± 0.001	46141 ± 1807	1.292			
4,6,7-Trimethoxy-5-methylcoumarin	0.119 ± 0.001	31889 ± 1895	0.893			
4',7-Di-O-methylnaringenin	0.129 ± 0.002	46046 ± 827	1.289			
4,8-Dihydroxyeudesm-7(11)-en-12,8-olide	0.120 ± 0.007	42343 ± 3400	1.186			
4,9,9'-Trihydroxy-3,3'-dimethoxy-8,4'-oxyneolignan	0.127 ± 0.001	31274 ± 1572	0.876			
4',9,9'-Trihydroxy-3'-methoxy-3,7'-epoxy-4,8'-oxyneolignan	0.131 ± 0.004	29018 ± 1408	0.812			

4,9-Dihydroxy-α-lapachone	0.119 ±0.007	28478 ±3043	0.797			
4,R-ajmalicine N-oxide	0.122 ±0.003	38319 ±2067	1.073			
4-Acetoxy-11(13)-pseudoguaien-12,8-olide	0.117 ±0.006	32184 ±2531	0.901			
4-Acetoxyphenol	0.111 ±0.004	20657 ±1079	0.578			
4-Acetyl-1,1-dimethylpiperazinium iodide	0.095 ±0.005	33311 ±4441	0.933			
4-Acetyl-3,6,8-trihydroxy-3-methyldihydronaphthalenone	0.124 ±0.008	35646 ±2076	0.998			
4-Allylpyrocatechol	0.114 ±0.004	27201 ±2194	0.762			
4-Amino-1,8-naphthalimide	0.099 ±0.004	34096 ±1770	0.955			
4-Aminobenzamidine dihydrochloride	0.098 ±0.001	34295 ±1200	0.960			
4-Amino-D, L-benzylsuccinic acid	0.111 ±0.001	33920 ±887	0.950			
4-Aminopyridine	0.108 ±0.002	25458 ±1428	0.713			
4-Aminosalicylic acid	0.119 ±0.006	50241 ±1249	1.407			
4-Androsten-4-ol-3,17-dione	0.097 ±0.000	36351 ±3939	1.018			
4-Cadinen-7-ol	0.112 ±0.003	37157 ±24	1.040			
4-Chloromercuribenzoic acid	0.093 ±0.010	49178 ±2259	1.377			
4-Chloro-N-(2-morpholin-4-yl-ethyl)-benzamide	0.108 ±0.012	33210 ±3436	0.930			
4-Chlorophenylguanidine hydrochloride	0.109 ±0.005	26912 ±408	0.754			
4-Cyano-3-methylisoquinoline	0.126 ±0.003	42041 ±3726	1.177			
4-DAMP	0.111 ±0.002	30648 ±1607	0.858			
4-DAMP methiodide	0.098 ±0.003	35768 ±1697	1.001			
4'-Demethyl-3,9-dihydroeucomin	0.112 ±0.004	52378 ±2690	1.467			
4'-demethylepipodophyllotoxin	0.097 ±0.003	25751 ±1268	0.721			
4'-Demethyleucomin	0.120 ±0.020	38943 ±2491	1.090			
4-Diphenylacetoxy-N-(2-chloroethyl)-piperidine hydrochloride	0.099 ±0.002	28798 ±3604	0.806			
4-Diphenylacetoxy-N-methylpiperidine methiodide	0.101 ±0.009	39470 ±4754	1.105			
4-Epialyxialactone	0.123 ±0.004	49086 ±2034	1.374			
4-Epicommunic acid	0.118 ±0.002	39705 ±4028	1.112			
4-Epi-isoinuviscolide	0.120 ±0.005	31679 ±1372	0.887			
4-HQN	0.109 ±0.012	42593 ±11877	1.193			
4-Hydroxy-2,6,6-trimethyl-1-cyclohexenecarboxylic acid	0.116 ±0.007	35319 ±1455	0.989			
4-Hydroxy-2-methoxyphenol 1-O-(6-O-syringoyl)-glucoside	0.126 ±0.006	51637 ±5898	1.446			

4-Hydroxy-3-(3-methyl-2-butenoyl)-<br>5-(3-methyl-2-butenyl)-benzoic acid	0.125 ± 0.007	32981 ± 2603	0.923			
4-Hydroxy-3,5-dimethoxybenzaldehyde	0.122 ± 0.006	38323 ± 2936	1.073			
4-Hydroxy-3-methoxyphenyl O-β-D-(6'-O-syringate)-glucopyranoside	0.120 ± 0.004	44928 ± 1387	1.258			
4-Hydroxy-3-methoxyphenylacetic acid	0.103 ± 0.006	32795 ± 1534	0.918			
4-Hydroxy-3-methoxy-phenylacetic acid	0.107 ± 0.007	37414 ± 3809	1.048			
4-Hydroxy-4-(methoxycarbonylmethyl)-cyclohexanone	0.120 ± 0.005	48849 ± 4521	1.368			
4-Hydroxy-6-methylpyran-2-one	0.101 ± 0.003	36956 ± 3385	1.035			
4'-Hydroxyacetophenone	0.116 ± 0.008	32889 ± 1941	0.921			
4-Hydroxyantipyrine	0.103 ± 0.004	42042 ± 1729	1.177			
4-Hydroxybenzaldehyde	0.127 ± 0.001	33052 ± 1415	0.925			
4-Hydroxybenzamide	0.110 ± 0.003	53146 ± 6785	1.488			
4-Hydroxybenzhydrazide	0.100 ± 0.007	39504 ± 3547	1.106			
4-Hydroxybenzoic acid	0.127 ± 0.010	39060 ± 2618	1.094			
4-Hydroxybenzyl alcohol	0.123 ± 0.006	35562 ± 1104	0.996			
4-Hydroxycephalotaxine	0.121 ± 0.002	35807 ± 476	1.003			
4'-Hydroxychalcone	0.096 ± 0.004	18962 ± 2370	0.546			
4-Hydroxycinnamamide	0.114 ± 0.006	50795 ± 5089	1.422			
4-Hydroxyindole	0.106 ± 0.002	36241 ± 2783	1.015			
4-Hydroxyphenethylamine hydrochloride	0.115 ± 0.008	24907 ± 2174	0.697			
4-Hydroxyphenylretinamide	0.119 ± 0.006	46056 ± 2628	1.290			
4-Hydroxyretinoic acid	0.119 ± 0.001	20526 ± 2250	0.545			
4'-Hydroxywogonin	0.110 ± 0.021	24927 ± 870	0.698			
4-IBP	0.111 ± 0.008	30451 ± 5558	0.853			
4-Imidazoleacrylic acid	0.112 ± 0.002	31808 ± 4051	0.891			
4-Imidazolemethanol hydrochloride	0.100 ± 0.005	65131 ± 4783	1.800			
4-IPP	0.111 ± 0.006	45319 ± 2151	1.269			
4-Methoxy-1-methoxycarbonyl-β-carboline	0.126 ± 0.010	53309 ± 4393	1.493			
4'-Methoxychalcone	0.100 ± 0.011	22809 ± 1910	0.639			
4-Methoxydalbergione	0.111 ± 0.004	18259 ± 1052	0.526			
4'-Methoxyflavone	0.107 ± 0.005	56514 ± 9384	1.582			
4-Methyldaphnetin	0.071 ± 0.015 **					

4-Methylesculetin	0.102 ± 0.015	24241 ± 1359	0.679			
4-Methylhistamine dihydrochloride	0.114 ± 0.014	38034 ± 3727	1.065			
4-Methylpyrazole hydrochloride	0.098 ± 0.010	26243 ± 4957	0.735			
4-Nonylphenol	0.108 ± 0.006	31297 ± 5120	0.876			
4'-O-Methylbroussochalcone B	0.119 ± 0.003	45832 ± 7029	1.283			
4-O-Methylphloracetophenone	0.111 ± 0.008	23079 ± 2371	0.646			
4-O-Methylsappanol	0.133 ± 0.011	37347 ± 3982	1.046			
4-Oxatetradecanoic acid	0.112 ± 0.003	48390 ± 6208	1.355			
4-Oxobedfordiaic acid	0.125 ± 0.001	41145 ± 2506	1.152			
4-Oxododecanedioic acid	0.129 ± 0.002	30580 ± 1599	0.856			
4-Phenyl-1-(4-phenylbutyl)-piperidine maleate	0.108 ± 0.007	35533 ± 1850	0.995			
4-Phenyl-1,2,3,4-tetrahydroisoquinoline hydrochloride	0.111 ± 0.005	34621 ± 5919	0.969			
4-Phenyl-2-propionamidotetralin	0.106 ± 0.007	41584 ± 6995	1.164			
4-PPBP Maleate	0.106 ± 0.007	24324 ± 957	0.681			
4-P-PDOT	0.110 ± 0.008	29822 ± 480	0.835			
4-Thiazolidinecarboxylic acid, 2-oxo-, (R)	0.103 ± 0.006	45527 ± 6098	1.275			
5-(3-Hydroxypropyl)-7-methoxybenzofuran	0.116 ± 0.007	53762 ± 3671	1.505			
5-(6-Hydroxybenzofuran-2-yl)-2-(3-methylbut-1-enyl)-benzene-1,3-diol	0.111 ± 0.003	49898 ± 4263	1.397			
5-(N,N-Dimethyl)-amiloride hydrochloride	0.074 ± 0.003 **					
5-(N,N-hexamethylene)-amiloride	0.103 ± 0.006	50351 ± 6376	1.410			
5'-(N-Cyclopropyl)-carboxamidoadenosine	0.100 ± 0.004	34754 ± 3750	0.973			
5-(N-Ethyl-N-isopropyl)-amiloride	0.096 ± 0.002	34331 ± 5075	0.961			
5-(N-Methyl-N-isobutyl)-amiloride	0.101 ± 0.002	40853 ± 6029	1.144			
5(S),12(R)-DiHETE all trans	0.104 ± 0.010	32324 ± 1458	0.905			
5(S),12(S)-DiHETE all trans	0.105 ± 0.004	31563 ± 1703	0.884			
5(S),15(S)-DiHETE	0.101 ± 0.004	47106 ± 1543	1.319			
5(S),6(R)-DiHETE	0.108 ± 0.005	29336 ± 2962	0.821			
5(S)-HETE	0.103 ± 0.005	42236 ± 2818	1.183			
5(S)-HPETE	0.108 ± 0.007	28536 ± 720	0.799			
5(Z), 8(Z)-Eicosadienoic acid	0.121 ± 0.003	29102 ± 2126	0.815			
5(Z),8(Z),11(Z),14(Z),17(Z)-Eicosapentaenoic acid	0.105 ± 0.005	44789 ± 4228	1.254			

5(Z),8(Z),11(Z),14(Z)-Eicosatetraenoic acid	0.105 ± 0.005	42709 ± 3710	1.196			
5(Z),8(Z),11(Z)-Eicosatrienoic acid	0.101 ± 0.003	44452 ± 1881	1.394			
5(Z)-Eicosenoic acid	0.104 ± 0.002	33885 ± 2671	0.949			
5-(Z-heptadec-8-enyl)-resorcinol	0.109 ± 0.011	29573 ± 1176	0.828			
5,19-Epoxy-19,25-dimethoxycucurbita-6,23-dien-3-ol	0.115 ± 0.003	43664 ± 4823	1.223			
5,19-Epoxy-25-methoxycucurbita-6,23-dien-3-ol	0.118 ± 0.004	40693 ± 1590	1.139			
5,5'-Dimethoxylariciresinol	0.121 ± 0.002	36415 ± 2848	1.020			
5,5'-Dimethoxylariciresinol 4-O-glucoside	0.117 ± 0.002	41821 ± 3138	1.171			
5,5-Dimethyl-1-pyrroline-N-oxide	0.100 ± 0.011	44035 ± 3503	1.233			
5,5-Diphenylhydantoin	0.100 ± 0.017	45136 ± 3785	1.264			
5,6,7,8-Tetramethoxycoumarin	0.107 ± 0.008	42095 ± 2940	1.179			
5,6-Epoxyeicosatrienoic acid	0.106 ± 0.006	29784 ± 1317	0.834			
5,6-O-Isopropylidene-phlorigidoside B	0.120 ± 0.005	44264 ± 3332	1.239			
5,7,3'-Trihydroxy-4'-methoxy-8-prenylflavanone	0.121 ± 0.006	35231 ± 1409	0.986			
5,7,3'-Trihydroxy-6,4',5'-trimethoxyflavanone	0.118 ± 0.011	38853 ± 2817	1.088			
5,7,8-Trimethoxycoumarin	0.123 ± 0.007	37330 ± 834	1.045			
5,7-Diacetoxy-3,4',8-trimethoxyflavone	0.105 ± 0.006	38401 ± 2343	1.075			
5,7-Diacetoxy-8-methoxyflavone	0.126 ± 0.005	34033 ± 1968	0.953			
5,7-Diacetoxyflavone	0.108 ± 0.007	22494 ± 893	0.630			
5,7-Dichlorokynurenic acid	0.106 ± 0.002	40036 ± 3010	1.121			
5,7-Dihydroxy-2-isopropylchromone	0.123 ± 0.002	44400 ± 2908	1.243			
5,7-Dihydroxy-3,4',8-trimethoxyflavone	0.112 ± 0.008	39655 ± 1151	1.110			
5,7-Dihydroxy-6,8-dimethoxyflavone	0.124 ± 0.002	35915 ± 598	1.006			
5,7-Dihydroxychromone	0.123 ± 0.003	35173 ± 3518	0.985			
5,8,11,14-Eicosatetraynoic acid	0.113 ± 0.004	33656 ± 1895	0.942			
5,8,11-Eicosatriynoic acid	0.108 ± 0.003	30625 ± 4254	0.857			
5,8,9,10,14-Pentaacetoxy-3-benzoyloxy-15-hydroxypepluane	0.129 ± 0.009	30815 ± 1783	0.863			
5-[(Dimethylamino)-methyl]-3-(1-methyl-1H-indol-3-yl)-1,2,4-oxadiazole hydrochloride	0.117 ± 0.008	42907 ± 5229	1.201			
5′-Deoxy-5′-methylthioadenosine	0.074 ± 0.010 **					
5-Acetoxy-7-hydroxyflavone	0.124 ± 0.012	40480 ± 1580	1.133			
5-Acetoxymatairesinol dimethyl ether	0.130 ± 0.002	37070 ± 1237	1.038			

5-Amino-2-hydroxy-benzoic acid	0.116 ± 0.005	18917 ± 1789	0.527			
5'-Amino-5'-deoxyadenosine p-toluenesulfonate salt	0.099 ± 0.005	19636 ± 1605	0.543			
5-Aminopentanoic acid hydrochloride	0.096 ± 0.015	42281 ± 2384	1.184			
5-Aminosalicylic acid	0.116 ± 0.011	39499 ± 5352	1.106			
5-Aminovaleric acid hydrochloride	0.104 ± 0.001	35215 ± 2660	0.986			
5-Aza-2-deoxycytidine	0.082 ± 0.008 *					
5-Azacytidine	0.072 ± 0.004 **					
5-BDBD	0.114 ± 0.004	35119 ± 2576	0.983			
5-Benzyloxytryptamine	0.113 ± 0.005	33835 ± 1165	0.947			
5b-Pregnan-3,20-dione	0.118 ± 0.012	53786 ± 6997	1.506			
5-Bromo-2'-deoxyuridine	0.056 ± 0.002 **					
5-Carboxamidotryptamine maleate	0.101 ± 0.009	32448 ± 1415	0.909			
5-Chloroindole-2-carboxylic acid	0.107 ± 0.014	87116 ± 3153	2.329	0.113 ± 0.006	52905 ± 4887	1.619
5'-Demethylaquillochin	0.122 ± 0.002	32225 ± 3175	0.902			
5-Deoxycajanin	0.116 ± 0.004	68366 ± 6399	1.911			
5-Epilithospermoside	0.123 ± 0.003	37184 ± 790	1.041			
5-Fluoro-2-pyrimidone	0.129 ± 0.002	35192 ± 6736	0.985			
5-Fluoro-5'-deoxyuridine	0.109 ± 0.003	27390 ± 1854	0.767			
5-Fluorocytosine	0.104 ± 0.010	13465 ± 851	0.375	0.105 ± 0.013	7483 ± 1371	0.229
5-Fluoroindole-2-carboxylic acid	0.106 ± 0.002	46626 ± 6374	1.306			
5-Fluorouracil	0.091 ± 0.002 *					
5-Glutinen-3-ol	0.125 ± 0.001	48854 ± 4329	1.368			
5HPP-33	0.095 ± 0.003 *					
5-Hydroxy-1,7-diphenyl-6-hepten-3-one	0.110 ± 0.012	32481 ± 810	0.909			
5-Hydroxy-2-pyrrolidinone	0.120 ± 0.003	39157 ± 1620	1.096			
5-Hydroxy-3',4',7-trimethoxyflavone	0.134 ± 0.005	44041 ± 1186	1.233			
5-Hydroxy-4',7-dimethoxyflavone	0.122 ± 0.002	42915 ± 3235	1.202			
5-Hydroxy-4',7-dimethoxyflavone 5-O-β-D-glucopyranoside	0.109 ± 0.002	36051 ± 1478	1.009			
5-Hydroxy-4-methoxycanthin-6-one	0.125 ± 0.015	28278 ± 1787	0.792			
5-Hydroxy-7,8-dimethoxyflavanone	0.122 ± 0.004	42776 ± 2371	1.198			
5-Hydroxy-7-acetoxy-8-methoxyflavone	0.127 ± 0.004	33747 ± 1720	0.945			
5-Hydroxy-7-acetoxyflavone	0.122 ± 0.004	22836 ± 1057	0.639			

5-Hydroxy-7-methoxy-3-(4-hydroxybenzylidene)-chroman-4-one	0.122 ± 0.009	56227 ± 5693	1.574			
5-Hydroxydecanoate	0.112 ± 0.006	35857 ± 1371	1.004			
5-Hydroxydecanoic acid sodium	0.097 ± 0.005	42819 ± 441	1.199			
5-Hydroxy-L-tryptophan	0.091 ± 0.004 *					
5-Hydroxymethyl-7-methoxybenzofuran	0.118 ± 0.005	51466 ± 3375	1.441			
5-Hydroxymethylfurfural	0.125 ± 0.003	34654 ± 3256	0.970			
5-Iodotubercidin	0.114 ± 0.004	31841 ± 1267	0.892			
5-Iodo-α-85380 dihydrochloride	0.111 ± 0.003	36938 ± 2987	1.034			
5-Ketoeicosatetraenoic acid	0.103 ± 0.005	36977 ± 2566	1.035			
5-Methoxy-DL-Tryptophan	0.110 ± 0.008	24548 ± 866	0.687			
5-Methoxy-N,N-dimethyltryptamine	0.117 ± 0.010	29295 ± 2246	0.820			
5-Methoxy-N-cyclopropanoyltryptamine	0.108 ± 0.003	99602 ± 7044	2.663	0.117 ± 0.008	62927 ± 4779	1.926
5-Methoxytryptamine	0.124 ± 0.004	64653 ± 6333	1.800			
5-Methoxytryptamine hydrochloride	0.109 ± 0.010	42830 ± 4629	1.199			
5-Methoxytryptophol	0.109 ± 0.008	40750 ± 3317	1.141			
5-Methylfurmethiodide	0.105 ± 0.009	28580 ± 2300	0.800			
5-Methylhydantoin	0.102 ± 0.004	36478 ± 3477	1.021			
5-Methylurapidil	0.101 ± 0.005	45801 ± 4032	1.282			
5'-N-Ethylcarboxamidoadenosine (NECA)	0.107 ± 0.001	30892 ± 2149	0.865			
5-Nitro-2-(3-phenylpropylamino)-benzoic acid	0.106 ± 0.002	41378 ± 2527	1.159			
5-Nitroisatin	0.105 ± 0.004	12441 ± 530	0.384	0.109 ± 0.005	31872 ± 2914	0.975
5'-N-Methylcarboxamidoadenosine	0.101 ± 0.007	30235 ± 2991	0.847			
5-Nonyloxytryptamine	0.088 ± 0.009 **					
5-Nonyloxytryptamine oxalate	0.059 ± 0.005 **					
5-O-Methylnaringenin	0.113 ± 0.005	41085 ± 2833	1.150			
5-Pentacosylresorcinol	0.125 ± 0.004	32843 ± 3425	0.920			
5'-S-Methyl-5'-thioadenosine	0.114 ± 0.010	23528 ± 2348	0.659			
5-Tricosyl-1,3-benzenediol	0.135 ± 0.004	41059 ± 4851	1.150			
5-α-Androstan-3,17-dione	0.119 ± 0.008	31643 ± 3442	0.886			
5-α-Cholestan-3-β-ol-6-one	0.109 ± 0.007	39951 ± 3273	1.119			
5-α-Cholestanol	0.107 ± 0.005	23527 ± 4791	0.659			
5-α-Epoxyalantolactone	0.111 ± 0.001	11130 ± 801	0.328	0.115 ± 0.004	23482 ± 3157	0.719

5-α-Hydroxycostic acid	0.116 ±0.003	30806 ±2031	0.863			
5-α-Pregnan-3-α-ol-11,20-dione	0.111 ±0.012	46774 ±1788	1.310			
5-α-Pregnan-3-α-ol-20-one	0.107 ±0.008	40540 ±2375	1.135			
5-α-Pregnane-3-α-2,1-diol-20-one	0.107 ±0.009	45721 ±996	1.280			
5-β-Hydroxycostic acid	0.116 ±0.002	23720 ±358	0.664			
6"-O-acetylisovitexin	0.131 ±0.011	40787 ±4224	1.142			
6(5H)-Phenanthridinone	0.113 ±0.007	67122 ±8373	1.855			
6(E)-Octadecenoic acid	0.116 ±0.003	35342 ±560	0.990			
6(Z),9(Z),12(Z),15(Z)-Octadecatetraenoic acid	0.113 ±0.002	30786 ±3490	0.862			
6(Z),9(Z),12(Z)-Octadecatrienoic acid	0.100 ±0.007	44118 ±1464	1.235			
6(Z)-Octadecenoic acid	0.122 ±0.018	27248 ±4558	0.763			
6-(β-D-glucopyranosyloxy)-Salicylic acid methyl ester	0.122 ±0.018	34507 ±2269	0.966			
6,19-Dihydroxyurs-12-en-3-oxo-28-oic acid	0.134 ±0.010	32929 ±1797	0.922			
6,2'-Dimethoxyflavone	0.110 ±0.005	35842 ±4468	1.004			
6,3'-Dimethoxyflavone	0.116 ±0.004	39618 ±5401	1.109			
6,4'-Dihydroxyflavone	0.103 ±0.008	30652 ±3734	0.858			
6,7,2',3',4'-Pentamethoxyisoflavone	0.110 ±0.011	35139 ±2812	0.984			
6,7,8-Trimethoxycoumarin	0.114 ±0.009	35226 ±6447	0.986			
6,7-ADTN hydrobromide	0.105 ±0.004	27529 ±1660	0.771			
6,7-Dichloro-3-hydroxy-2-quinoxalinecarboxylic acid	0.102 ±0.007	34805 ±1392	0.975			
6,7-Dichloroquinoxaline-2,3-dione	0.095 ±0.004	33227 ±1819	0.930			
6',7'-Dihydroxybergamottin	0.121 ±0.018	35595 ±2190	0.997			
6',7'-Dihydroxybergamottin acetonide	0.123 ±0.016	20407 ±396	0.570			
6,7-Dinitroquinoxaline-2,3-dione	0.110 ±0.008	29391 ±5138	0.823			
6,8-Cyclo-1,4-eudesmanediol	0.127 ±0.011	33527 ±1974	0.939			
6,9,10-Trihydroxy-7-megastigmen-3-one	0.114 ±0.005	20681 ±785	0.578			
6-[2-(4-Imidazolyl)-ethylamino]-N -(4-trifluoromethylphenyl)-heptanecarboxamide dimaleate	0.108 ±0.009	20659 ±1841	0.578			
6-Acetonyldihydrochelerythrine	0.105 ±0.008	44800 ±1848	1.254			
6-Acetonyldihydrosanguinarine	0.116 ±0.001	43656 ±798	1.222			
6-Acetonyl-N-methyl-dihydrodecarine	0.120 ±0.009	46555 ±4479	1.304			
6-Acetyl-2,2-dimethylchroman-4-one	0.115 ±0.003	31740 ±901	0.889			

6a-Fluorotestosterone	0.123 ±0.004	52714 ±4133	1.476			
6-Aminohexanoic acid	0.113 ±0.003	39210 ±1449	1.098			
6-Aminoindazole	0.122 ±0.002	30182 ±2562	0.845			
6-Aminonicotinamide	0.102 ±0.005	26512 ±1671	0.742			
6-Azauridine	0.107 ±0.006	25108 ±3062	0.703			
6-Benzoyl-5,7-dihydroxy-2,2-dimethylchromane	0.109 ±0.004	45553 ±5163	1.275			
6-Chlorokynurenic acid	0.101 ±0.006	36380 ±7188	1.019			
6-Chloromelatonin	0.099 ±0.005	39107 ±807	1.095			
6-Cyano-7-nitroquinoxaline-2,3-dione	0.104 ±0.010	35538 ±5083	0.995			
6-Deoxy-3-O-methyl-β-allopyranosyl (1→4)-β-cymaronic acid δ-lactone	0.123 ±0.003	33605 ±2023	0.941			
6-Deoxy-9-α-hydroxycedrodorin	0.120 ±0.002	40607 ±3677	1.137			
6-Epidemethylesquirolin D	0.134 ±0.005	19206 ±1834	0.537			
6-Ethoxygeniposide	0.128 ±0.006	37501 ±2381	1.050			
6-Feruloylcatalpol	0.130 ±0.009	44044 ±5773	1.233			
6-Fluoronorepinephrine hydrochloride	0.098 ±0.004	36187 ±3701	1.013			
6-Formylindolo [3,2-B] carbazole	0.110 ±0.007	42036 ±2181	1.177			
6-Furfurylaminopurine	0.108 ±0.004	52629 ±5190	1.474			
6H-Pyrido[2,3-b][1,4]benzodiazepin-6-one, 11-[[2-[(diethylamino)-methyl]-1-piperidinyl]acetyl]-5,11-dihydro	0.126 ±0.003	33977 ±7924	0.951			
6-Hydroxy-2,6-dimethyl-2,7-octadienoic acid	0.109 ±0.002	37613 ±1935	1.053			
6'-Hydroxy-7'-ethoxybergamottin	0.121 ±0.001	38473 ±2730	1.077			
6-Hydroxybenzofuran-2(3H)-one	0.110 ±0.001	45500 ±2542	1.274			
6-Hydroxy-DL-DOPA	0.106 ±0.001	60527 ±1407	1.673			
6-Hydroxyflavone	0.101 ±0.005	19658 ±638	0.566			
6-Hydroxymelatonin	0.103 ±0.006	31825 ±1807	0.891			
6-Hydroxyrubiadin	0.084 ±0.001 **					
6-Hydroxystigmast-4-en-3-one	0.109 ±0.010	32278 ±3237	0.904			
6-Hydroxystigmasta-4,22-dien-3-one	0.118 ±0.005	41100 ±4652	1.151			
6-Iodo-nordihydrocapsaicin	0.109 ±0.005	28615 ±1269	0.801			
6-Keto-prostaglandin F1a	0.102 ±0.002	30232 ±2390	0.846			
6-Methoxy-1,2,3,4-tetrahydro-9H-pyrido[3,4b] indole	0.100 ±0.001	36414 ±2444	1.020			
6-Methoxynaringenin	0.124 ±0.006	31623 ±1770	0.885			

6-Methoxytryptamine	0.112 ± 0.001	32508 ± 5629	0.910			
6-Methyl-2-(phenylethynyl)-pyridine hydrochloride	0.105 ± 0.017	49223 ± 6452	1.378			
6-Nitroquipazine maleate	0.104 ± 0.011	45981 ± 8167	1.287			
6-Nitroso-1,2-benzopyrone	0.113 ± 0.007	62979 ± 1469	1.741			
6-O-(3",4"-Dimethoxycinnamoyl)-catalpol	0.125 ± 0.003	32221 ± 1687	0.902			
6"-O-Acetylastragalin	0.131 ± 0.009	33682 ± 2097	0.943			
6-O-Acetylcoriatin	0.131 ± 0.017	34759 ± 5388	0.973			
6'-O-Acetylpaniculoside II	0.124 ± 0.008	35570 ± 2515	0.996			
6-O-Caffeoylarbutin	0.130 ± 0.004	41774 ± 2977	1.170			
6-O-Cinnamoylcatalpol	0.119 ± 0.005	41059 ± 2446	1.150			
6-O-Feruloylglucose	0.120 ± 0.010	52131 ± 6189	1.460			
6-O-Methylcerevisterol	0.122 ± 0.007	29867 ± 633	0.836			
6-O-p-Hydroxybenzoylaucubin	0.107 ± 0.012	22228 ± 794	0.622			
6'-O-p-Hydroxybenzoylcatalposide	0.116 ± 0.004	34415 ± 2568	0.964			
6-O-p-Methoxycinnamoylcatalpol	0.118 ± 0.002	44712 ± 6062	1.252			
6-O-Syringoylajugol	0.120 ± 0.006	34570 ± 520	0.968			
6-O-Vanilloylajugol	0.126 ± 0.005	42370 ± 2361	1.186			
6-β-Hydroxyipolamiide	0.127 ± 0.004	35244 ± 3435	0.987			
7-(b-Hydroxyethyl)-theophylline	0.101 ± 0.000	32148 ± 4358	0.900			
7(E)-Nonadecenoic acid	0.118 ± 0.037	32030 ± 3347	0.897			
7-(Hydroxyimino)-cyclopropa[B]chromen-1A-carboxylate ethyl ester	0.103 ± 0.008	36599 ± 3692	1.025			
7(Z),10(Z),13(Z),16(Z)-Ocosatetraenoic acid	0.102 ± 0.005	30813 ± 4676	0.863			
7(Z),7(Z)-Dimethyleicosadienoic acid	0.105 ± 0.018	34551 ± 2136	0.967			
7(Z)-Nonadecenoic acid	0.109 ± 0.017	33842 ± 2481	0.948			
7,15-Dihydroxy-8(14)-podocarpen-13-one	0.119 ± 0.006	41095 ± 3794	1.151			
7,15-Dihydroxydehydroabietic acid methyl ester	0.120 ± 0.005	43123 ± 2138	1.207			
7,2'-Dihydroxyflavone	0.104 ± 0.006	26165 ± 1720	0.733			
7,3',4'-Trihydroxy-3-benzyl-2H-chromene	0.100 ± 0.002	24016 ± 1642	0.672			
7,3'-Dihydroxy-4'-methoxyflavan	0.123 ± 0.001	48543 ± 2846	1.359			
7,3'-Dimethoxyflavone	0.106 ± 0.005	25614 ± 2238	0.717			
7,4'-Dihydroxy-3'-prenylflavan	0.122 ± 0.016	46891 ± 5515	1.313			
7,4'-Di-O-methylapigenin 5-O-xylosylglucoside	0.130 ± 0.008	32736 ± 2414	0.917			

7,7-Dimethyl-(5Z,8Z)-eicosadienoic acid	0.105 ±0.004	30222 ±2548	0.846			
7,7-Dimethyleicosadienoic acid	0.106 ±0.009	32302 ±517	0.904			
7,8,9,9-Tetradehydroisolariciresinol	0.114 ±0.013	29642 ±787	0.830			
7,8-Dihydroxyflavone	0.102 ±0.003	19896 ±555	0.573			
7-Acetoxycoumarin	0.108 ±0.018	35030 ±1654	0.981			
7-Aminocephalosporanic acid	0.107 ±0.009	40875 ±3069	1.144			
7-Chloro-3-methyl-3,4-dihydro-2H-1,2,4-benzothiadiazine-S,S-dioxide	0.107 ±0.009	39009 ±7541	1.092			
7-Chloro-4-hydroxy-2-phenyl-1,8-naphthyridine	0.112 ±0.005	37569 ±2440	1.052			
7-Chlorokynurenic acid	0.114 ±0.011	34643 ±6624	0.970			
7-Cyclopentyl-5-(4-phenoxy)-phenyl-7H-pyrrolo[2,3-d]pyrimidin-4-ylamine	0.094 ±0.008	37167 ±3726	1.041			
7-Deacetoxy-7-oxokhivorin	0.108 ±0.008	8601 ±533	0.248	0.113 ±0.010	9323 ±356	0.285
7-deacetylkhivorin	0.117 ±0.002	50738 ±6041	1.421			
7-Desacetoxy-6,7-dehydrogedunin	0.111 ±0.007	20255 ±1230	0.567			
7-Geranyloxy-5-methoxycoumarin	0.120 ±0.013	38694 ±1496	1.083			
7-Geranyloxy-6-methoxycoumarin	0.130 ±0.001	41714 ±846	1.168			
7-Hydroxy-2',5,8-trimethoxyflavanone	0.129 ±0.007	31836 ±1647	0.891			
7-Hydroxy-2'-methoxyisoflavone	0.108 ±0.006	36654 ±1669	1.026			
7-Hydroxy-3-(4-hydroxybenzylidene)-chroman-4-one	0.113 ±0.003	47163 ±3185	1.321			
7-Hydroxy-3-prenylcoumarin	0.127 ±0.003	37086 ±352	1.038			
7-Hydroxy-5,8-dimethoxyflavanone	0.123 ±0.005	47750 ±4977	1.337			
7-Hydroxy-6-methoxy-3-prenylcoumarin	0.122 ±0.003	34864 ±3000	0.976			
7-Hydroxycoumarin-6-carboxylic acid	0.116 ±0.016	39638 ±1482	1.110			
7-Hydroxydarutigenol	0.133 ±0.013	46644 ±3920	1.306			
7-Hydroxy-DPAT hydrobromide	0.108 ±0.009	27817 ±2470	0.779			
7-Hydroxyethyltheophylline	0.105 ±0.009	38643 ±1686	1.082			
7-Hydroxyflavone	0.098 ±0.003	39622 ±3335	1.109			
7-Hydroxy-PIPAT maleate	0.105 ±0.008	39402 ±6916	1.103			
7-Isopentenyloxy-γ-fagarine	0.125 ±0.003	36943 ±1867	1.034			
7-Methoxy-8-prenyloxycoumarin	0.121 ±0.002	62642 ±8221	1.845			
7-NINA	0.105 ±0.008	34572 ±4972	0.968			
7-Nitroindazole	0.111 ±0.003	39760 ±7438	1.113			

7-O-Demethyl-3-isomangostin hydrate	0.111 ± 0.002	44003 ± 2434	1.232			
7'-O-Ethylmarmin	0.128 ± 0.004	27918 ± 2583	0.782			
7-O-Methyleriodictyol	0.124 ± 0.003	36755 ± 4222	1.029			
7-Oxodehydroabietinol	0.119 ± 0.007	47569 ± 2186	1.332			
7-Oxohinokinin	0.132 ± 0.001	35663 ± 1729	0.999			
7-Oxo-β-sitosterol	0.126 ± 0.003	41496 ± 2437	1.162			
7-α-Hydroxystigmasterol	0.127 ± 0.002	37471 ± 792	1.049			
8753	0.110 ± 0.009	37120 ± 1329	1.039			
8(14),15-Isopimaradien-3-ol	0.133 ± 0.006	34726 ± 1830	0.972			
8(14),15-Isopimaradiene-3,18-diol	0.121 ± 0.003	50292 ± 4804	1.408			
8(17),13-Labdadien-15,16-olide	0.116 ± 0.008	41871 ± 6420	1.172			
8-(3-Chlorostyryl)-caffeine	0.103 ± 0.014	30520 ± 2051	0.855			
8-(4-Chlorophenylthio)-cAMP sodium	0.095 ± 0.005	44137 ± 5206	1.236			
8-(p-Sulfophenyl)-theophylline	0.106 ± 0.001	37907 ± 1725	1.061			
8(R),15(S)-DiHETE all trans	0.104 ± 0.003	30499 ± 2625	0.854			
8(S),15(S)-DiHETE all trans	0.105 ± 0.007	30425 ± 3110	0.852			
8(S)-HETE	0.103 ± 0.005	28708 ± 1685	0.804			
8(Z),11(Z),14(Z)-Eicosatrienoic acid	0.098 ± 0.011	46365 ± 853	1.454			
8(Z)-Eicosenoic acid	0.119 ± 0.006	26533 ± 2429	0.743			
8,11,13-Abietatriene-7,15,18-triol	0.117 ± 0.003	38422 ± 4440	1.076			
8,14-Epoxyergosta-4,22-diene-3,6-dione	0.127 ± 0.001	33832 ± 1224	0.947			
8,9-Epoxyeicosatrienoic acid	0.108 ± 0.003	29215 ± 1845	0.818			
8-Azaspiro[4.5]decane-7,9-dione, 8-[2-[[(2,3-dihydro-1,4-benzodioxin-2-yl)-methyl]amino]ethyl]-, monomethanesulfonate	0.124 ± 0.001	36872 ± 3973	1.032			
8-Benzoyl-5,7-dihydroxy-2,2-dimethylchromane	0.113 ± 0.003	40489 ± 5378	1.134			
8-Bromo-cAMP sodium	0.107 ± 0.004	45931 ± 1069	1.286			
8-Bromo-cGMP sodium	0.109 ± 0.007	49911 ± 3702	1.397			
8-Chloro-11-piperidin-4-ylidene-6,11-dihydro-5H-benzo[5,6]cyclohepta[1,2-b]pyridine	0.145 ± 0.005 **					
8-Cyclopentyl-1,3-dimethylxanthine	0.102 ± 0.010	32442 ± 1510	0.908			
8-Cyclopentyltheophylline	0.101 ± 0.004	37024 ± 5199	1.037			
8-Deoxygartanin	0.123 ± 0.004	48512 ± 6194	1.358			
8-Epi-Prostaglandin F2a	0.115 ± 0.004	34033 ± 1500	0.953			

8-Geranyloxy-5,7-dimethoxycoumarin	0.121 ± 0.006	34979 ± 550	0.979			
8-Geranyloxypsoralen	0.111 ± 0.011	33791 ± 376	0.946			
8-Hydroxy-17-chloro-12-labden-16,15-olide	0.119 ± 0.002	37159 ± 600	1.040			
8-Hydroxy-3,5,7,3',4',5'-hexamethoxyflavone	0.138 ± 0.016	29280 ± 2918	0.820			
8-Hydroxy-4-cadinen-3-one	0.119 ± 0.001	38180 ± 1885	1.069			
8-Hydroxy-5-O-β-D-glucopyranosylpsoralen	0.124 ± 0.002	20286 ± 2118	0.568			
8-hydroxycarapinic acid	0.120 ± 0.005	26568 ± 2355	0.744			
8-Hydroxydigitoxigenin	0.124 ± 0.005	55880 ± 7091	1.565			
8-Hydroxy-DPAT hydrobromide	0.099 ± 0.007	34906 ± 1454	0.977			
8-Hydroxyhyperforin 8,1-hemiacetal	0.123 ± 0.004	30161 ± 331	0.844			
8-Hydroxyodoroside A	0.124 ± 0.008	41752 ± 3438	1.169			
8-Hydroxy-PIPAT oxalate	0.120 ± 0.006	40923 ± 1471	1.146			
8-Isomulberrin hydrate	0.134 ± 0.010	40241 ± 3012	1.127			
8-Methoxy-2-propionamidotetralin	0.106 ± 0.004	61987 ± 8753	1.736			
8-Methoxybonducellin	0.128 ± 0.004	41269 ± 6294	1.156			
8-Methoxymethyl-3-isobutyl-1-methylxanthine	0.106 ± 0.008	32333 ± 2938	0.905			
8-M-PDOT	0.106 ± 0.001	36105 ± 978	1.011			
8-Nitrotheophylline	0.113 ± 0.007	46447 ± 1160	1.300			
8-O-Acetylharpagide	0.131 ± 0.002	40886 ± 1364	1.145			
8-O-Acetylshanzhiside methyl ester	0.112 ± 0.004	42479 ± 4245	1.189			
8-O-Demethyl-7-O-methyl-3,9-dihydropunctatin	0.112 ± 0.002	41534 ± 3339	1.163			
8-Oxoadenine	0.115 ± 0.001	35248 ± 1505	0.987			
8-Prenyldaidzein	0.125 ± 0.006	43356 ± 3076	1.214			
8-α-Hydroxy--α-gurjunene	0.124 ± 0.003	46619 ± 3060	1.305			
8-β-hydroxycarapin, 3,8-hemiacetal	0.120 ± 0.008	44555 ± 3155	1.248			
9(11),12-Oleanadien-3-ol	0.120 ± 0.003	45288 ± 4964	1.268			
9(E),11(Z)-Octadecadienoic acid	0.113 ± 0.002	33809 ± 1889	0.947			
9(E),12(E)-Octadecadienoic acid	0.122 ± 0.005	28998 ± 3414	0.812			
9(E)-Hexadecenoic acid	0.113 ± 0.003	31670 ± 1843	0.887			
9(E)-Octadecenoic acid	0.142 ± 0.006 **					
9(E)-Tetradecenoic acid	0.123 ± 0.008	32971 ± 4734	0.923			
9(S)-HETE	0.102 ± 0.002	34562 ± 995	0.968			

9(S)-HODE	0.114 ± 0.005	30184 ± 1516	0.845			
9(S)-HPODE	0.113 ± 0.006	33075 ± 441	0.926			
9(Z),11(Z)-Octadecadienoic acid	0.109 ± 0.007	34208 ± 3692	0.958			
9(Z),12(Z),15(Z)-Octadecatrienoic acid	0.106 ± 0.003	30625 ± 2977	0.857			
9(Z),17-Octadecadiene-12,14-diyne-1,11,16-triol	0.130 ± 0.004	21758 ± 468	0.609			
9(Z)-Hexadecenoic acid	0.138 ± 0.004 **					
9(Z)-Octadecenoic acid	0.137 ± 0.004 **					
9(Z)-Tetradecenoic acid	0.108 ± 0.002	32263 ± 5131	0.903			
9,10-Octadecenoamide	0.117 ± 0.011	37409 ± 1284	1.047			
9,11,13-Octadecatriynoic acid methyl ester	0.117 ± 0.002	41125 ± 3078	1.151			
9,13-Epidioxy-8(14)-abieten-18-oic acid	0.138 ± 0.001	27027 ± 1006	0.757			
9,16-Dioxo-10,12,14-octadecatrienoic acid	0.132 ± 0.009	37984 ± 2235	1.064			
9,9'-Di-O-(E)-feruloylsecoisolariciresinol	0.130 ± 0.024	29086 ± 1611	0.814			
9,9'-O-isopropyllidene-isolariciresinol	0.115 ± 0.003	30483 ± 1611	0.854			
9-AC	0.108 ± 0.008	30039 ± 3205	0.841			
9-Amino-1,2,3,4-tetrahydroacridine hydrochloride	0.081 ± 0.004 **					
9-cis Retinoic acid	0.112 ± 0.026	30488 ± 1241	0.854			
9-cyclopentyladenine	0.101 ± 0.006	39485 ± 2455	1.106			
9-Dihydroxypropyladenine	0.107 ± 0.013	36822 ± 4894	1.031			
9-Epiblumenol B	0.128 ± 0.006	37483 ± 2240	1.050			
9-Hydroxy-13E-labden-15-oic acid	0.123 ± 0.001	27018 ± 1090	0.756			
9-Hydroxycalabaxanthone	0.121 ± 0.010	41975 ± 4670	1.175			
9-Hydroxycalabaxanthone hydrate	0.125 ± 0.014	42781 ± 3491	1.198			
9-Hydroxydarutigenol	0.130 ± 0.015	48859 ± 3481	1.368			
9-Hydroxy-α-lapachone	0.111 ± 0.007	34768 ± 812	0.973			
9-Methoxy-α-lapachone	0.127 ± 0.006	29145 ± 2591	0.816			
9-O-Feruloyl-5,5'-dimethoxylariciresinol	0.129 ± 0.004	48487 ± 2899	1.358			
9-O-Feruloyllariciresinol	0.120 ± 0.003	38557 ± 2776	1.080			
9-Oxo-10,11-dehydroageraphorone	0.118 ± 0.002	39459 ± 379	1.105			
9-Oxo-2,7-bisaboladien-15-oic acid	0.115 ± 0.004	38178 ± 2474	1.069			
9-Oxoageraphorone	0.124 ± 0.004	35890 ± 1756	1.005			
9-Oxonerolidol	0.105 ± 0.005	27584 ± 1564	0.772			

9-α,11-β-Prostaglandin F2	0.110 ± 0.006	35586 ± 1866	0.996			
9-β,11-α-Prostaglandin F2	0.103 ± 0.006	41661 ± 2944	1.166			
A 205804	0.110 ± 0.002	28927 ± 1232	0.810			
A 61603 hydrobromide	0.121 ± 0.005	45252 ± 1822	1.267			
A 740003	0.118 ± 0.002	30421 ± 2149	0.852			
A 769662	0.108 ± 0.007	46203 ± 1946	1.294			
A 77636 hydrochloride	0.106 ± 0.006	18170 ± 1554	0.503			
a,b-Methyleneadenosine 5'-triphosphate Li	0.098 ± 0.001	38814 ± 4958	1.087			
a,b-Methyleneadenosine-5'-diphosphate Na	0.099 ± 0.002	20434 ± 3010	0.572			
A-134974 dihydrochloride hydrate	0.111 ± 0.017	36326 ± 3394	1.017			
A-23187	0.113 ± 0.011	24228 ± 1089	0.678			
A-3	0.118 ± 0.003	11210 ± 1956	0.341	0.103 ± 0.005	9382 ± 361	0.287
A-315456	0.101 ± 0.001	44019 ± 4327	1.233			
A-7 hydrochloride	0.114 ± 0.003	7240 ± 422	0.200	0.113 ± 0.006	32880 ± 4925	1.006
A77 1726	0.095 ± 0.008	29767 ± 1354	0.833			
A-77636 hydrochloride	0.110 ± 0.007	13745 ± 772	0.368	0.121 ± 0.009	27355 ± 680	0.837
AA-861	0.100 ± 0.001	40708 ± 2116	1.140			
AAF-CMK	0.112 ± 0.005	10670 ± 630	0.330	0.120 ± 0.003	28324 ± 1176	0.867
Abacavir	0.115 ± 0.008	44352 ± 1900	1.242			
Abacavir Sulfate	0.107 ± 0.003	53314 ± 9621	1.493			
Abamectin	0.104 ± 0.002	33773 ± 3468	0.946			
Abiesadine I	0.115 ± 0.005	44192 ± 1804	1.237			
Abiesadine N	0.115 ± 0.004	32074 ± 976	0.898			
Abietic acid	0.107 ± 0.002	35120 ± 1896	0.983			
AB-MECA	0.109 ± 0.003	34050 ± 1583	0.953			
Abscisic acid, (±)-	0.097 ± 0.004 *					
ABT 724 trihydrochloride	0.114 ± 0.002	31464 ± 4506	0.881			
ABT-418 hydrochloride	0.102 ± 0.001	38235 ± 1852	1.071			
ABT-702 dihydrochloride	0.090 ± 0.005 *					
ABT-737	0.118 ± 0.008	35938 ± 1326	1.006			
ABT-869	0.118 ± 0.002	31793 ± 2974	0.890			
AC 264613	0.107 ± 0.003	40318 ± 1566	1.129			

AC 55649	0.108 ± 0.003	32352 ± 2656	0.906			
AC220	0.122 ± 0.008	29506 ± 6732	0.826			
AC-55649	0.112 ± 0.009	30749 ± 451	0.861			
AC-93253 iodide	0.057 ± 0.004 **					
ACA	0.095 ± 0.003	22375 ± 1303	0.626			
Acacetin	0.124 ± 0.004	30216 ± 639	0.846			
Acacetin diacetate	0.100 ± 0.001	45574 ± 1344	1.276			
Acacetine	0.097 ± 0.012	17005 ± 802	0.505			
Acaciin	0.116 ± 0.020	38020 ± 4521	1.065			
Acadesine	0.107 ± 0.008	30414 ± 3428	0.852			
Acamprosate calcium	0.116 ± 0.003	40931 ± 565	1.146			
Acanthoside B	0.125 ± 0.003	42566 ± 2420	1.192			
Acarbose	0.116 ± 0.002	70514 ± 5897	1.963			
ACDPP hydrochloride	0.132 ± 0.003 **					
ACEA	0.112 ± 0.005	28743 ± 1856	0.805			
Acebutolol hydrochloride	0.114 ± 0.001	54474 ± 3584	1.525			
Acecainide hydrochloride	0.102 ± 0.007	30990 ± 2274	0.868			
Aceclidine	0.103 ± 0.004	37341 ± 4386	1.046			
Aceclidine hydrochloride	0.098 ± 0.015	44648 ± 8847	1.250			
Aceclidine Salicylate	0.111 ± 0.009	33590 ± 638	0.941			
Aceclofenac	0.123 ± 0.006	64691 ± 3609	1.840			
Acedapsone	0.107 ± 0.008	28873 ± 4860	0.808			
Acedoben	0.107 ± 0.003	33271 ± 1993	0.932			
Acefylline	0.113 ± 0.004	29163 ± 4017	0.817			
Aceglutamide	0.112 ± 0.014	40586 ± 2774	1.136			
Acemetacin	0.107 ± 0.020	24003 ± 3143	0.672			
Aceneuramic acid	0.106 ± 0.003	32701 ± 4465	0.916			
Acenocoumarol	0.112 ± 0.003	41965 ± 1996	1.175			
Acepromazine maleate	0.111 ± 0.001	24683 ± 665	0.691			
Acesulfame potassium	0.103 ± 0.006	34533 ± 3581	0.967			
Acetamide	0.098 ± 0.002	34233 ± 5322	0.959			
Acetamide, 2-amino-N-(1-methyl-1,2-diphenylethyl)-, (+/-)	0.120 ± 0.003	20267 ± 5356	0.567			

Acetaminophen	0.109 ± 0.004	50242 ± 5641	1.407			
Acetaminosalol	0.117 ± 0.005	26182 ± 914	0.733			
Acetanilide	0.099 ± 0.014	24488 ± 1068	0.686			
Acetarsol	0.106 ± 0.008	34773 ± 2277	0.974			
Acetazolamide	0.097 ± 0.001	34365 ± 1600	0.962			
Acetohexamide	0.103 ± 0.002	36594 ± 1506	1.025			
Acetohydroxamic acid	0.102 ± 0.003	25166 ± 2890	0.705			
Acetophenazine maleate	0.107 ± 0.003	33114 ± 3442	0.927			
Acetopromazine maleate salt	0.109 ± 0.005	31716 ± 2224	0.888			
Acetosyringone	0.111 ± 0.016	32689 ± 530	0.915			
Acetriazoic acid	0.100 ± 0.003	22015 ± 965	0.634			
Acetyl (N)-S-farnesyl-L-cysteine	0.113 ± 0.004	27032 ± 497	0.757			
Acetyl isogambogic acid	0.110 ± 0.004	26783 ± 1250	0.750			
Acetyl-11-keto-β-Boswellic Acid, Boswellia serrata	0.095 ± 0.002	52611 ± 11873	1.473			
Acetylcephalotaxine	0.133 ± 0.007	27420 ± 2858	0.768			
Acetylcholine chloride	0.101 ± 0.005	29825 ± 3997	0.835			
Acetylcysteine	0.118 ± 0.005	45370 ± 2755	1.270			
Acetyldihydromicromelin A	0.121 ± 0.012	34999 ± 335	0.980			
Acetyldisulfanilamide	0.109 ± 0.009	28031 ± 1684	0.785			
Acetylepipodophyllotoxin	0.117 ± 0.002	44093 ± 4819	1.235			
Acetyl-farnesyl-cysteine	0.114 ± 0.002	43084 ± 1394	1.206			
Acetyl-geranygeranyl-cysteine	0.124 ± 0.006	32959 ± 1272	0.923			
Acetyl-geranyl-cysteine	0.113 ± 0.005	47842 ± 2820	1.340			
Acetylglucosamine	0.105 ± 0.006	32448 ± 642	0.909			
Acetylglutamic acid	0.086 ± 0.008 *					
Acetylisocupressic acid	0.116 ± 0.010	29861 ± 1766	0.836			
Acetyl-l-leucine	0.114 ± 0.002	36144 ± 759	1.012			
Acetylphenylalanine	0.100 ± 0.004	37202 ± 1743	1.042			
Acetylsalicylic acid	0.091 ± 0.002 *					
Acetylsalicylsalicylic acid	0.106 ± 0.002	29454 ± 2034	0.825			
Acetylsulfadimethoxine	0.106 ± 0.010	35351 ± 2209	0.990			
Acetylthiocholine chloride	0.099 ± 0.001	36736 ± 2612	1.029			

Acetylursolic acid	0.109 ± 0.010	36222 ± 560	1.014			
Acetyl-β-methylcholine chloride	0.098 ± 0.001	31804 ± 767	0.890			
Acexamic acid	0.118 ± 0.001	34778 ± 1760	0.974			
Acifran	0.110 ± 0.006	31968 ± 538	0.895			
Acipimox	0.112 ± 0.001	40233 ± 2244	1.127			
Acitretin	0.117 ± 0.004	42902 ± 2055	1.201			
Acivicin	0.072 ± 0.002 **					
Aclarubicin	0.107 ± 0.018	2285 ± 268	0.065	0.111 ± 0.008	17376 ± 1409	0.532
Ac-Leu-Leu-Nle-CHO	0.113 ± 0.013	36646 ± 1353	1.026			
Aconiazide	0.114 ± 0.012	23672 ± 3645	0.663			
Aconitic acid	0.111 ± 0.008	36981 ± 3859	1.035			
Aconitine	0.108 ± 0.004	36814 ± 1955	1.031			
ACPA	0.109 ± 0.004	26944 ± 1508	0.754			
Acriflavinium hydrochloride	0.066 ± 0.005 **					
Acrisorcin	0.067 ± 0.009 **					
Acroteben	0.112 ± 0.011	40903 ± 3336	1.145			
Actarit	0.121 ± 0.008	45262 ± 2646	1.267			
Actein	0.128 ± 0.007	38792 ± 2891	1.086			
Acteoside	0.137 ± 0.005	25112 ± 285	0.703			
Actinidic acid	0.116 ± 0.000	51645 ± 3134	1.446			
Actinomycin D	0.102 ± 0.009	22716 ± 1637	0.636			
Actinonin	0.119 ± 0.002	31609 ± 1419	0.885			
Acuminatin	0.128 ± 0.007	39939 ± 3976	1.118			
Acycloguanosine	0.117 ± 0.001	32227 ± 2812	0.902			
Acyclovir	0.100 ± 0.002	35046 ± 2269	0.981			
Adamantamine fumarate	0.114 ± 0.007	41772 ± 1765	1.170			
Adapalene	0.118 ± 0.005	32358 ± 1506	0.906			
Adapromin	0.102 ± 0.005	43202 ± 3699	1.210			
Adenanthin	0.132 ± 0.006	20034 ± 1588	0.561			
Adenine	0.101 ± 0.003	18367 ± 1362	0.529			
Adenosine	0.099 ± 0.003	25001 ± 1993	0.700			
Adenosine 3',5'-cyclic monophosphate	0.100 ± 0.003	44871 ± 2272	1.256			

Adenosine 5'-monophosphate monohydrate	0.111 ± 0.004	43756 ± 2710	1.225			
Adenosine amine congener	0.103 ± 0.003	32137 ± 2842	0.900			
Adenosine Kinase Inhibitor	0.110 ± 0.004	35623 ± 5690	0.997			
Adenosine phosphate	0.107 ± 0.005	19920 ± 1391	0.558			
Adenosine, N-(2-hydroxycyclopentyl)-, (1S-trans)	0.121 ± 0.003	65914 ± 5396	1.835			
Adenosine-5'-diphosphate Na (ADP)	0.097 ± 0.009	21322 ± 1201	0.597			
Adenosine-5'-diphosphoribose	0.106 ± 0.007	22416 ± 913	0.628			
Adenosine-5'-monophosphate Na (AMP)	0.103 ± 0.004	22304 ± 882	0.624			
Adiphenine hydrochloride	0.122 ± 0.008	32482 ± 1133	0.909			
Adipic acid	0.108 ± 0.002	27759 ± 1002	0.777			
Adlumidine	0.110 ± 0.005	48426 ± 2636	1.356			
Adonitol	0.105 ± 0.003	32485 ± 2945	0.910			
Adrenalone	0.114 ± 0.002	43192 ± 2527	1.209			
Adrenic acid (22:4 n-6)	0.103 ± 0.006	38047 ± 2312	1.065			
Adrenochrome	0.129 ± 0.004 *					
Adrenolone hydrochloride	0.104 ± 0.003	35993 ± 3969	1.008			
Adrenosterone	0.106 ± 0.006	45017 ± 6099	1.260			
AEG 3482	0.111 ± 0.005	29980 ± 957	0.839			
a-Ergocryptine	0.109 ± 0.004	25919 ± 2305	0.726			
Afalanine	0.106 ± 0.008	25105 ± 2104	0.703			
AF-DX 116	0.110 ± 0.002	28001 ± 610	0.784			
AFMK	0.098 ± 0.010	35357 ± 1784	0.990			
Afzelin	0.113 ± 0.004	32157 ± 1371	0.900			
AG 1024	0.115 ± 0.007	17989 ± 5960	0.504			
AG 112	0.126 ± 0.014	23815 ± 436	0.667			
AG 126	0.092 ± 0.003 **					
AG 1295	0.129 ± 0.016	26360 ± 760	0.738			
AG 1296	0.122 ± 0.003	38175 ± 3972	1.069			
AG 13958	0.129 ± 0.006	32761 ± 3635	0.917			
AG 1478	0.068 ± 0.015 **					
AG 18	0.099 ± 0.006	18475 ± 873	0.511			
AG 213 (Tyrphostin 47)	0.113 ± 0.006	46630 ± 2469	1.306			

AG 370	0.120 ± 0.001	39563 ± 3130	1.108			
AG 490	0.121 ± 0.012	30235 ± 1047	0.847			
AG 494	0.091 ± 0.003 **					
AG 555	0.108 ± 0.004	35704 ± 1450	1.000			
AG 556	0.108 ± 0.007	43659 ± 2571	1.222			
AG 825	0.114 ± 0.003	30075 ± 2811	0.842			
AG 879	0.123 ± 0.005	34110 ± 3277	0.955			
AG 9	0.118 ± 0.017	33534 ± 2429	0.939			
AG 99	0.107 ± 0.005	31297 ± 3089	0.876			
Agaric acid	0.113 ± 0.005	40671 ± 671	1.139			
Agathadiol diacetate	0.138 ± 0.019	44550 ± 1425	1.247			
Agatholal	0.120 ± 0.002	39775 ± 2028	1.114			
AGC	0.107 ± 0.009	30515 ± 1962	0.854			
Agelasine	0.057 ± 0.009 **					
AGGC	0.107 ± 0.006	30575 ± 3169	0.856			
AGK2	0.102 ± 0.013	24844 ± 5946	0.696			
AGL 2043	0.097 ± 0.003 *					
Aglinin A	0.127 ± 0.005	39850 ± 899	1.116			
Agmatine sulfate	0.097 ± 0.001	26356 ± 2684	0.738			
AGN 192403 hydrochloride	0.118 ± 0.006	29861 ± 1585	0.836			
Agnuside	0.129 ± 0.014	38395 ± 2670	1.075			
Agrimonolide	0.112 ± 0.004	40470 ± 1790	1.133			
Agrimonolide 6-O-glucoside	0.121 ± 0.021	40962 ± 5864	1.147			
Agroclavine	0.107 ± 0.007	24299 ± 127	0.680			
AH 11110 hydrochloride	0.112 ± 0.010	24882 ± 5261	0.697			
AH 11110A	0.124 ± 0.009	12364 ± 383	0.331	0.121 ± 0.006	43841 ± 3359	1.342
AH 6809	0.108 ± 0.002	24521 ± 152	0.687			
AhR Antagonist	0.099 ± 0.003	30739 ± 820	0.861			
AICA-Riboside	0.115 ± 0.014	31415 ± 789	0.880			
AICA-Riboside, 5′-Phosphate	0.102 ± 0.004	24170 ± 447	0.677			
AIDA	0.103 ± 0.005	42226 ± 3272	1.182			
Ailanthone	0.124 ± 0.003	33026 ± 280	0.925			

AJ-76	0.115 ± 0.011	22320 ± 3950	0.625			
Ajmalicine	0.114 ± 0.005	43364 ± 1110	1.214			
Ajmaline	0.111 ± 0.009	17732 ± 1270	0.527			
Aklomide	0.102 ± 0.007	36555 ± 899	1.024			
Akt Inhibitor IV	0.061 ± 0.011 **					
Akt Inhibitor V, Triciribine	0.124 ± 0.010	33210 ± 421	0.930			
Akt Inhibitor VIII, Isozyme-Selective, Akti-1/2	0.115 ± 0.008	34810 ± 2732	0.975			
Akt Inhibitor X	0.137 ± 0.010	23790 ± 3045	0.666			
Akt Inhibitor XII, Isozyme-Selective, Akti-2	0.103 ± 0.003	55115 ± 1360	1.632			
Akt-I-1	0.119 ± 0.005	30432 ± 6085	0.852			
Akt-I-1,2	0.125 ± 0.001	34458 ± 2616	0.965			
AL-8810	0.092 ± 0.004	51442 ± 4430	1.440			
Ala-Ala-Phe-CMK	0.111 ± 0.013	21588 ± 2323	0.604			
Alamethicin	0.097 ± 0.008	128589 ± 9448	3.907	0.105 ± 0.008	22934 ± 3798	0.702
Alanyl-dl-leucine	0.105 ± 0.022	29964 ± 1502	0.839			
Alaproclate	0.103 ± 0.006	19545 ± 537	0.563			
Alaproclate hydrochloride	0.108 ± 0.003	36730 ± 6472	1.028			
Albaspidin AA	0.118 ± 0.003	35565 ± 2930	0.996			
Albendazole	0.115 ± 0.001	36168 ± 2442	1.013			
Albendazole sulfone	0.111 ± 0.013	33484 ± 1648	0.938			
Alboctalol	0.117 ± 0.001	34109 ± 1137	0.955			
Albuterol	0.102 ± 0.005	31601 ± 1072	0.885			
Albuterol hemisulfate	0.065 ± 0.006 **					
Alclometasone dipropionate	0.128 ± 0.012	52256 ± 2850	1.463			
alclometazone dipropionate	0.114 ± 0.002	32461 ± 966	0.909			
Alcuronium chloride	0.118 ± 0.012	32659 ± 459	0.914			
Alendronate	0.116 ± 0.001	38161 ± 1632	1.068			
Alendronate sodium	0.121 ± 0.003	40433 ± 3788	1.132			
Alepterolic acid	0.126 ± 0.002	32141 ± 2891	0.900			
Aleuretic acid	0.108 ± 0.006	33170 ± 2166	0.929			
Alexidine dihydrochloride	0.059 ± 0.008 **					
Alexidine hydrochloride	0.063 ± 0.008 **					

Alfacalcidol	0.112 ± 0.005	46075 ± 2938	1.290			
Alfadolone acetate	0.118 ± 0.004	39596 ± 4428	1.109			
Alfuzosin	0.120 ± 0.007	59263 ± 3724	1.659			
Alfuzosin hydrochloride	0.105 ± 0.007	38215 ± 6527	1.070			
Algestone acetophenide	0.104 ± 0.002	51342 ± 5737	1.438			
Alinidine	0.111 ± 0.019	34670 ± 1205	0.971			
Aliskiren hemifumarate	0.108 ± 0.016	19910 ± 1870	0.574			
Alismol	0.129 ± 0.008	35687 ± 3682	0.999			
Alizapride hydrochloride	0.112 ± 0.008	43465 ± 2415	1.217			
Alizarin	0.075 ± 0.004 **					
Allantoin	0.135 ± 0.006	31222 ± 2053	0.874			
Allitol	0.122 ± 0.005	42396 ± 2071	1.187			
ALLN	0.093 ± 0.003	40494 ± 1370	1.134			
Allocryptopine	0.117 ± 0.003	46428 ± 3147	1.300			
Allopregnanolone	0.111 ± 0.005	32673 ± 1900	0.915			
Allopurinol	0.102 ± 0.001	23494 ± 1967	0.649			
Alloxan	0.103 ± 0.002	28921 ± 533	0.810			
Alloxazine	0.110 ± 0.001	29271 ± 3297	0.820			
All-trans-Retinol	0.110 ± 0.005	23295 ± 2392	0.652			
Allylisothiocyanate	0.112 ± 0.005	21020 ± 1899	0.589			
Allylthiourea	0.105 ± 0.009	27208 ± 1870	0.762			
Almotriptan	0.105 ± 0.002	37881 ± 4262	1.061			
Aloeemodin	0.115 ± 0.004	37212 ± 2922	1.042			
Aloe-emodine	0.111 ± 0.008	17677 ± 376	0.525			
Aloin	0.110 ± 0.010	29380 ± 3453	0.823			
Aloisine A, RP107	0.110 ± 0.005	31325 ± 1117	0.877			
Aloisine, RP106	0.111 ± 0.004	32009 ± 1206	0.896			
Alosetron hydrochloride	0.125 ± 0.005	34725 ± 2027	0.972			
Alphitolic acid	0.124 ± 0.002	25221 ± 2189	0.706			
Alpinetin	0.119 ± 0.008	44813 ± 7750	1.255			
Alpinumisoflavone	0.123 ± 0.007	39511 ± 1447	1.106			
Alprazolam	0.123 ± 0.006	20999 ± 2378	0.585			

Alprenolol	0.103 ± 0.004	30786 ± 2940	0.862			
Alprenolol hydrochloride	0.106 ± 0.010	11733 ± 2007	0.314	0.106 ± 0.005	30974 ± 2677	0.948
Alprostadil	0.108 ± 0.001	39251 ± 4701	1.099			
Alrestatin	0.110 ± 0.001	24808 ± 1665	0.695			
Alsterpaullone	0.104 ± 0.002	39129 ± 2975	1.096			
Alsterpaullone,2-Cyanoethyl	0.114 ± 0.005	32042 ± 662	0.897			
Alstonic acid A	0.125 ± 0.004	50886 ± 6034	1.425			
Alstonic acid B	0.131 ± 0.004	37708 ± 2851	1.056			
Altanserin	0.134 ± 0.002 **					
Altanserin hydrochloride	0.107 ± 0.006	14813 ± 118	0.410	0.120 ± 0.006	47326 ± 8263	1.448
Althiazide	0.117 ± 0.000	28880 ± 2440	0.809			
Altrenogest	0.109 ± 0.008	25948 ± 2072	0.727			
Altretamine	0.097 ± 0.002	43504 ± 1941	1.218			
Alverine citrate	0.108 ± 0.009	26839 ± 4249	0.751			
ALX 5407 hydrochloride	0.104 ± 0.006	36446 ± 1388	1.020			
Alyxialactone	0.127 ± 0.003	26037 ± 443	0.729			
AM 251	0.113 ± 0.005	32038 ± 2765	0.897			
AM 281	0.122 ± 0.012	50141 ± 2341	1.404			
AM 404	0.109 ± 0.007	30827 ± 4385	0.863			
AM 580	0.111 ± 0.002	42134 ± 5762	1.180			
AM 630	0.108 ± 0.010	34460 ± 1111	0.965			
AM 735	0.103 ± 0.005	48558 ± 3788	1.360			
AM 92016	0.119 ± 0.018	37375 ± 2318	1.046			
Amanozine	0.110 ± 0.013	34063 ± 2945	0.954			
Amantadine	0.111 ± 0.009	39336 ± 1077	1.101			
Amantadine hydrochloride	0.111 ± 0.008	39826 ± 1682	1.115			
Amantidine	0.112 ± 0.008	38397 ± 314	1.075			
Amaronol A	0.119 ± 0.002	31295 ± 247	0.876			
Amaronol B	0.132 ± 0.009	25446 ± 425	0.712			
Ambenonium 2Cl	0.101 ± 0.009	44598 ± 7106	1.249			
Ambrisentan	0.115 ± 0.019	54700 ± 3471	1.493			
Ambroxol	0.115 ± 0.005	39311 ± 744	1.101			

Ambroxol hydrochloride	0.113 ± 0.004	58789 ± 6268	1.604			
Amcinonide	0.117 ± 0.004	40477 ± 2791	1.133			
Amendol	0.108 ± 0.010	35471 ± 3269	0.993			
Amentoflavone	0.104 ± 0.004	40151 ± 1780	1.124			
Amethopterin	0.113 ± 0.001	38974 ± 915	1.091			
Amfebutamone	0.114 ± 0.008	50822 ± 2017	1.423			
Amfebutamone hydrochloride	0.121 ± 0.010	54989 ± 4749	1.540			
Amfepramone hydrochloride	0.116 ± 0.003	64223 ± 3015	1.753			
AMG-47a	0.118 ± 0.003	35258 ± 3721	0.987			
AMG-9810	0.104 ± 0.009	34007 ± 2015	0.952			
AMG-Tie2-1	0.127 ± 0.005	36782 ± 659	1.030			
AMI-193	0.110 ± 0.005	35479 ± 3322	0.993			
Amidopyrine	0.113 ± 0.002	39541 ± 3211	1.107			
Amifostine	0.100 ± 0.001	34661 ± 1473	0.970			
Amikacin hydrate	0.110 ± 0.001	36356 ± 202	1.018			
Amikacin sulfate	0.107 ± 0.002	25693 ± 3153	0.719			
Amiloride	0.115 ± 0.006	32859 ± 1956	0.920			
Amiloride hydrochloride	0.097 ± 0.005	53017 ± 6403	1.484			
Amiloride hydrochloride dihydrate	0.119 ± 0.005	27568 ± 2780	0.772			
Aminacrine	0.071 ± 0.002 **					
Amino-1,8-naphthalimide [4-Amino-1,8-naphthalimide]	0.106 ± 0.001	28213 ± 3234	0.790			
Aminobenzamide (3-ABA)[3-Aminobenzamide (3-ABA)]	0.111 ± 0.003	28877 ± 2663	0.809			
Aminobenztropine	0.105 ± 0.001	36797 ± 3604	1.030			
Aminocaproic acid	0.112 ± 0.003	36926 ± 6597	1.034			
Aminoethoxydiphenylborane	0.070 ± 0.002 **					
Aminoglutethimide	0.110 ± 0.003	38335 ± 2324	1.073			
Aminoguanidine	0.105 ± 0.004	29340 ± 1030	0.822			
Aminoguanidine hemisulfate	0.101 ± 0.001	30889 ± 4090	0.865			
Aminoguanidine hydrochloride	0.102 ± 0.002	34084 ± 1769	0.954			
Aminohippuric acid	0.109 ± 0.002	42513 ± 5897	1.190			
Aminohydroxybutyric acid	0.117 ± 0.009	36400 ± 1450	1.019			
Aminolevulinic acid hydrochloride	0.103 ± 0.008	31046 ± 2814	0.869			

Aminopenicillanic acid	0.108 ± 0.007	42011 ± 4102	1.176			
Aminopentamide sulfate	0.108 ± 0.009	32459 ± 1074	0.909			
Aminophosphonobutyric acid	0.112 ± 0.010	44431 ± 1323	1.244			
Aminophosphonopropionic acid	0.104 ± 0.004	48749 ± 1253	1.365			
Aminophylline	0.106 ± 0.007	37277 ± 3554	1.044			
Aminophylline ethylenediamine	0.099 ± 0.002	35167 ± 2141	0.985			
Aminopterin	0.106 ± 0.002	47924 ± 3624	1.342			
Aminopurine, 6-benzyl	0.117 ± 0.006	53685 ± 3335	1.465			
Aminopurvalanol A	0.106 ± 0.020	32733 ± 1454	0.917			
Aminopyrine	0.111 ± 0.008	30203 ± 987	0.846			
Aminoresveratrol	0.102 ± 0.004	41304 ± 3082	1.156			
Aminosalicylate sodium	0.108 ± 0.010	42077 ± 8354	1.178			
Aminothiazole	0.113 ± 0.011	31173 ± 353	0.873			
Amiodarone	0.114 ± 0.013	40487 ± 1901	1.134			
Amiodarone hydrochloride	0.060 ± 0.001 **					
Amiprilose hydrochloride	0.099 ± 0.002	43053 ± 4658	1.205			
Amisulpride	0.113 ± 0.003	28119 ± 1450	0.787			
Amitraz	0.108 ± 0.006	50434 ± 6655	1.412			
Amitriptyline hydrochloride	0.083 ± 0.000 **					
Amlexanox	0.123 ± 0.004	33911 ± 2577	0.949			
Amlodipine	0.109 ± 0.023	27511 ± 4745	0.770			
Amlodipine base	0.122 ± 0.003	20470 ± 673	0.570			
Amlodipine besylate	0.117 ± 0.006	18209 ± 1108	0.504			
Ammonium lactate	0.108 ± 0.007	39314 ± 4932	1.101			
Ammonium pyrrolidinedithiocarbamate	0.097 ± 0.003	29365 ± 2759	0.822			
AMN 082 dihydrochloride	0.057 ± 0.008 **					
AMN082	0.060 ± 0.011 **					
Amodiaquin dihydrochloride dihydrate	0.115 ± 0.003	34986 ± 2579	0.980			
Amodiaquine	0.114 ± 0.002	39345 ± 4767	1.102			
Amorolfine	0.100 ± 0.009	63286 ± 7840	1.800			
Amorolfine hydrochloride	0.106 ± 0.003	44113 ± 6552	1.235			
Amoxapine	0.106 ± 0.007	32058 ± 861	0.898			

Amoxicillin	0.107 ±0.021	46028 ±8785	1.289			
Ampelopsin	0.124 ±0.003	22991 ±1000	0.644			
Amperozide	0.112 ±0.009	19723 ±172	0.552			
Amperozide hydrochloride	0.104 ±0.001	32208 ±2177	0.902			
Amphotericin B	0.107 ±0.007	17912 ±1691	0.532			
Ampicillin sodium	0.120 ±0.008	31148 ±7283	0.872			
Ampicillin trihydrate	0.107 ±0.004	49502 ±1546	1.386			
Ampiroxicam	0.121 ±0.002	43395 ±3236	1.215			
AMPK Activator	0.093 ±0.003 *					
AMPK Inhibitor, Compound C	0.103 ±0.003	28620 ±2048	0.801			
Amprenavir	0.111 ±0.006	47416 ±4539	1.328			
Amprolium	0.103 ±0.003	18181 ±3226	0.509			
Amprolium hydrochloride	0.123 ±0.006	19470 ±1245	0.531			
Ampyrone	0.117 ±0.001	45465 ±3571	1.273			
Ampyzine sulfate	0.103 ±0.004	35316 ±2244	0.989			
Amrinone	0.112 ±0.001	50325 ±5381	1.409			
Amsacrine	0.110 ±0.009	21792 ±2636	0.610			
Amsacrine hydrochloride	0.106 ±0.002	37970 ±4380	1.063			
Amsonate	0.113 ±0.003	39624 ±2132	1.109			
AMT hydrochloride	0.109 ±0.001	31141 ±3732	0.872			
Amthamine	0.108 ±0.015	31561 ±2055	0.884			
Amthamine dihydrobromide	0.105 ±0.004	27783 ±1972	0.778			
Amtizole	0.119 ±0.002	33029 ±306	0.925			
Amygdalin	0.094 ±0.007 *					
Amyleine hydrochloride	0.113 ±0.009	46846 ±216	1.312			
Amylene hydrate	0.105 ±0.009	31410 ±3546	0.879			
Anabasamine hydrochloride	0.113 ±0.004	30180 ±1869	0.845			
Anabasine hydrochloride	0.117 ±0.001	24990 ±1399	0.700			
Anacardic acid	0.108 ±0.002	31973 ±1149	0.895			
Anagrelide	0.108 ±0.002	44259 ±3029	1.239			
Anagrelide hydrochloride	0.119 ±0.007	60297 ±3284	1.679			
Anandamide	0.110 ±0.003	30681 ±464	0.859			

Anandamide (18:2,n-6)	0.108 ± 0.004	31594 ± 858	0.885			
Anandamide (20:3,n-6)	0.104 ± 0.002	31069 ± 899	0.870			
Anandamide (20:4, n-6)	0.110 ± 0.009	29749 ± 1572	0.833			
Anandamide (22:4,n-6)	0.103 ± 0.003	26797 ± 4632	0.750			
Anastrozole	0.120 ± 0.002	63004 ± 3877	1.754			
Ancitabine hydrochloride	0.111 ± 0.004	37865 ± 2229	1.060			
Andrographolide	0.113 ± 0.003	25018 ± 2076	0.700			
Andrographolide, dehydro-	0.099 ± 0.008	52395 ± 2356	1.556			
Andropanolide	0.127 ± 0.004	36344 ± 799	1.018			
Androsta-1,4-dien-3,17-dione	0.110 ± 0.004	40321 ± 1979	1.129			
Androstenedione	0.123 ± 0.006	39663 ± 3292	1.111			
Androsterone	0.124 ± 0.004	50313 ± 2219	1.409			
Androsterone acetate	0.108 ± 0.001	19167 ± 238	0.552			
Anethole	0.109 ± 0.009	29591 ± 821	0.829			
Anethole trithione	0.122 ± 0.010	40912 ± 3111	1.146			
Anethole, trans-	0.111 ± 0.006	32262 ± 2114	0.903			
Anethole-trithione	0.109 ± 0.008	41599 ± 778	1.165			
Angelicain	0.130 ± 0.012	34338 ± 2222	0.961			
Angelicin	0.124 ± 0.002	33564 ± 2383	0.940			
Angeloylisogomisin O	0.117 ± 0.005	36857 ± 3004	1.032			
Angiogenesis Inhibitor	0.097 ± 0.001	39381 ± 1212	1.103			
Angiotensin II (human)	0.109 ± 0.003	20970 ± 1506	0.587			
Angolensin (R)	0.101 ± 0.007	38160 ± 3616	1.068			
Anhydrobrazilic acid	0.094 ± 0.005 *					
Anhydrotuberosin	0.122 ± 0.004	41687 ± 3488	1.167			
Aniracetam	0.107 ± 0.003	42099 ± 3909	1.179			
Anisindione	0.106 ± 0.009	50324 ± 3947	1.409			
Anisodamine	0.095 ± 0.001 *					
Anisodamine hydrobromide	0.114 ± 0.023	37176 ± 5067	1.041			
Anisofolin A	0.122 ± 0.007	49786 ± 9707	1.394			
Anisomycin	0.114 ± 0.009	24164 ± 1757	0.677			
Anpirtoline	0.101 ± 0.012	34308 ± 3965	0.961			

Anpirtoline hydrochloride	0.117 ± 0.002	40490 ± 3390	1.134			
Antazoline hydrochloride	0.115 ± 0.006	37700 ± 362	1.056			
Antazoline phosphate	0.108 ± 0.005	30202 ± 5415	0.846			
Anthothecol	0.109 ± 0.005	37091 ± 1053	1.039			
Anthralin	0.129 ± 0.010	26387 ± 1167	0.739			
Antiarol	0.121 ± 0.012	43016 ± 4267	1.204			
Antiarol rutinoside	0.129 ± 0.009	38612 ± 3572	1.081			
Antibiotic A-23187	0.115 ± 0.009	66162 ± 6222	1.965			
Antidesmone	0.112 ± 0.002	37939 ± 2363	1.062			
Antidiabetic	0.112 ± 0.014	41438 ± 4721	1.160			
Antiinflammatory	0.112 ± 0.006	17035 ± 1352	0.502			
Antimony potassium tartrate trihydrate	0.058 ± 0.010 **					
Antimycin A	0.122 ± 0.002	47589 ± 4199	1.332			
Antimycin A1	0.098 ± 0.006	24038 ± 392	0.673			
Antipyrine	0.116 ± 0.007	42056 ± 3018	1.178			
Antipyrine, 4-hydroxy	0.110 ± 0.022	42837 ± 1036	1.199			
Antozoline hydrochloride	0.107 ± 0.003	38313 ± 2731	1.073			
Aphidicolin	0.106 ± 0.002	25232 ± 1761	0.706			
Aphyllic acid	0.107 ± 0.004	27746 ± 847	0.777			
API-2	0.110 ± 0.001	54233 ± 7240	1.518			
Apicidin	0.114 ± 0.011	32445 ± 2144	0.908			
Apigenin	0.119 ± 0.003	52652 ± 995	1.474			
Apigenin 4'-O-rhamnoside	0.115 ± 0.017	33766 ± 3460	0.945			
Apigenin 5-O-β-D-glucopyranoside	0.120 ± 0.003	45708 ± 2831	1.280			
Apigenin 7-O-methylglucuronide	0.117 ± 0.002	43418 ± 4565	1.216			
Apigenin dimethyl ether	0.104 ± 0.003	26759 ± 1923	0.749			
Apigenin-7-O-glucoside	0.106 ± 0.002	47135 ± 1055	1.400			
Apiole	0.102 ± 0.005	33396 ± 2457	0.935			
Apocynin	0.095 ± 0.001	34537 ± 1436	0.967			
Apocynol A	0.127 ± 0.004	37322 ± 1692	1.045			
Apomorphine hydrochloride	0.103 ± 0.003	23896 ± 6325	0.669			
Apomorphine hydrochloride hemihydrate	0.095 ± 0.003	39625 ± 1781	1.109			

Apomorphine r (-)	0.100 ± 0.004	43465 ± 2088	1.217			
Apoptosis Activator II	0.107 ± 0.012	45991 ± 4438	1.288			
Apoptosis Activator VI, CD437/AHPN	0.090 ± 0.004 *					
Apoptosis Inhibitor II, NS3694	0.094 ± 0.006	44295 ± 1077	1.240			
Apoptosis Inhibitor	0.097 ± 0.005	18207 ± 1330	0.533			
Apotoxicarol	0.109 ± 0.005	33358 ± 2011	0.934			
Apramycin	0.115 ± 0.006	38815 ± 4646	1.087			
Apramycin sulfate	0.109 ± 0.004	26632 ± 4773	0.746			
Aprepitant	0.101 ± 0.007	30704 ± 3432	0.860			
Aprindine hydrochloride	0.118 ± 0.010	32441 ± 1216	0.908			
Aprofene	0.113 ± 0.020	36577 ± 389	1.024			
Apstatin	0.117 ± 0.003	34555 ± 1341	0.968			
AQ-RA 741	0.109 ± 0.002	31728 ± 1541	0.888			
Arabitol(d)	0.104 ± 0.009	30948 ± 2695	0.867			
Arachidonamide	0.111 ± 0.005	35204 ± 1109	0.986			
Arachidonic acid (20:4, n-6)	0.112 ± 0.030	32125 ± 2193	0.899			
Arachidonoyl alanine	0.128 ± 0.001	38148 ± 968	1.068			
Arachidonoyl dopamine	0.102 ± 0.004	28042 ± 3842	0.785			
Arachidonoyl ethanolamide	0.127 ± 0.001	27193 ± 3653	0.761			
Arachidonoyl GABA	0.130 ± 0.003	32178 ± 298	0.901			
Arachidonoyl glycine	0.137 ± 0.007	26080 ± 869	0.730			
Arachidonoyl-PAF	0.099 ± 0.003	26346 ± 2293	0.738			
Araneosol	0.125 ± 0.020	35408 ± 3529	0.991			
Arbutin	0.096 ± 0.004 *					
ARC 239 dihydrochloride	0.107 ± 0.012	61573 ± 2656	1.646			
Arcaine sulfate	0.101 ± 0.002	34969 ± 436	0.979			
Arctigenin	0.103 ± 0.001	42068 ± 749	1.178			
Arctinol B	0.112 ± 0.006	58013 ± 3152	1.621			
Arcyriaflavin A	0.106 ± 0.003	30502 ± 2668	0.854			
Arecaidine but-2-ynyl ester tosylate	0.103 ± 0.004	29148 ± 5476	0.816			
Arecaidine propargyl ester hydrobromide	0.110 ± 0.008	38967 ± 4719	1.091			
Arecaidine propargyl ester tosylate	0.103 ± 0.001	28489 ± 2397	0.798			

Arecoline hydrobromide	0.098 ± 0.007	33022 ± 3588	0.925			
Argatroban	0.112 ± 0.002	41379 ± 4972	1.159			
Arginine hydrochloride	0.103 ± 0.028	47576 ± 1516	1.332			
Arglabin	0.127 ± 0.005	17765 ± 2169	0.523			
Aricine	0.131 ± 0.004	26079 ± 1250	0.730			
Arillatose B	0.113 ± 0.005	44041 ± 4101	1.233			
Aripiprzaole	0.124 ± 0.006	44545 ± 2704	1.247			
Aristolactam AIIIa	0.128 ± 0.002	31468 ± 1632	0.881			
Aristolactam BII	0.120 ± 0.004	36833 ± 1271	1.031			
Aristolactam BIII	0.126 ± 0.006	35661 ± 2259	0.998			
Aristolactam FI	0.126 ± 0.020	25711 ± 5511	0.720			
Aristolochic Acid	0.103 ± 0.006	11119 ± 953	0.325	0.103 ± 0.008	6078 ± 329	0.186
Arjunic acid	0.125 ± 0.006	43649 ± 676	1.222			
Arjunolic acid	0.127 ± 0.004	39039 ± 1620	1.093			
ARL 67156 trisodium salt	0.105 ± 0.002	39494 ± 4976	1.106			
Armodafinil	0.100 ± 0.001	49485 ± 9248	1.386			
Aromadendrin	0.117 ± 0.003	55970 ± 4280	1.567			
ARP 101	0.099 ± 0.007	41705 ± 1218	1.168			
Arphamenine A	0.117 ± 0.003	37396 ± 2262	1.047			
Arsanilic acid	0.105 ± 0.004	38267 ± 4741	1.071			
Arsenic trioxide	0.090 ± 0.003 **					
Artanin	0.130 ± 0.010	36967 ± 2468	1.035			
Arteannuin A	0.120 ± 0.004	36336 ± 493	1.017			
Arteannuin B	0.121 ± 0.001	38650 ± 520	1.082			
Arteannuin L	0.122 ± 0.008	37707 ± 2278	1.056			
Arteannuin M	0.119 ± 0.003	37075 ± 3092	1.038			
Arteannuin N	0.110 ± 0.003	36300 ± 4181	1.016			
Artemether	0.121 ± 0.003	68294 ± 4937	1.902			
Artemetin	0.131 ± 0.018	35799 ± 623	1.002			
Artemetin acetate	0.109 ± 0.005	29644 ± 893	0.830			
Artemisin	0.112 ± 0.006	31787 ± 2963	0.890			
Artemisinic acid	0.125 ± 0.002	37891 ± 2338	1.061			

Artemisinin	0.123 ± 0.001	41693 ± 1960	1.167			
Artenimol	0.107 ± 0.001	48322 ± 3311	1.353			
Artesunate	0.110 ± 0.007	27159 ± 1501	0.760			
Arthonioic acid	0.108 ± 0.008	34305 ± 4088	0.961			
Articaine hydrochloride	0.123 ± 0.023	33823 ± 746	0.947			
Artocarpin	0.124 ± 0.005	38620 ± 1508	1.081			
Artoheterophyllin B	0.113 ± 0.004	47392 ± 3016	1.327			
Arvanil	0.112 ± 0.006	25927 ± 1065	0.726			
AS 1949490	0.122 ± 0.006	33457 ± 4288	0.937			
AS 604850	0.114 ± 0.005	51487 ± 3085	1.423			
AS-252424	0.089 ± 0.004 *					
Asarinin, (-)-	0.103 ± 0.001	23623 ± 517	0.661			
Asarylaldehyde	0.099 ± 0.005	30479 ± 1594	0.853			
Ascleposide E	0.123 ± 0.008	37840 ± 3388	1.059			
Ascomycin (FK-520)	0.101 ± 0.004	30520 ± 520	0.855			
Ascorbic acid	0.112 ± 0.004	49750 ± 1131	1.389			
Ascorbyl palmitate	0.101 ± 0.029	48826 ± 3326	1.367			
Asenapine maleate	0.134 ± 0.007	33082 ± 4131	0.926			
Asiatic acid	0.123 ± 0.002	37174 ± 1941	1.041			
Aspartame	0.103 ± 0.004	33192 ± 4190	0.929			
Aspartic acid (L)	0.095 ± 0.008	21768 ± 4152	0.609			
Asperglaucide	0.121 ± 0.002	33324 ± 1716	0.933			
Aspirin	0.089 ± 0.002 **					
Astaxanthin	0.113 ± 0.008	30372 ± 2430	0.850			
Astemizole	0.099 ± 0.010	57633 ± 9984	1.614			
Astilbin	0.123 ± 0.005	40883 ± 2324	1.145			
Astragalin	0.133 ± 0.005	33232 ± 1344	0.930			
Astragalin 4',2",3",4",6"-pentaacetate	0.118 ± 0.002	46335 ± 4644	1.297			
Astragaloside iv	0.109 ± 0.007	28769 ± 3317	0.806			
AT 101	0.109 ± 0.006	43936 ± 4066	1.230			
AT-7519	0.061 ± 0.003 **					
AT9283	0.128 ± 0.003	31168 ± 2566	0.873			

Atazanavir	0.114 ± 0.013	34545 ± 3458	0.967			
Atenolol	0.110 ± 0.010	27077 ± 3487	0.758			
ATM Kinase Inhibitor	0.113 ± 0.004	22390 ± 1319	0.627			
ATM/ATR Kinase Inhibitor	0.109 ± 0.011	36533 ± 2560	1.023			
Atomoxetine hydrochloride	0.107 ± 0.006	33455 ± 1073	0.937			
Atorvastatin	0.114 ± 0.009	46669 ± 5856	1.307			
Atorvastatin Calcium	0.107 ± 0.008	24003 ± 2207	0.672			
Atovaquone	0.110 ± 0.002	43925 ± 3754	1.230			
ATP disodium	0.101 ± 0.006	19530 ± 328	0.547			
ATPA	0.108 ± 0.006	32668 ± 4737	0.915			
ATPO	0.105 ± 0.002	40045 ± 2612	1.121			
ATRA-BA Hybrid	0.101 ± 0.004	23405 ± 684	0.655			
Atractyloside potassium salt	0.113 ± 0.002	39380 ± 6797	1.103			
Atracurium besylate	0.131 ± 0.005 *					
Atranorin	0.121 ± 0.001	32912 ± 1803	0.922			
Atraric acid	0.126 ± 0.003	51141 ± 3512	1.432			
Atropine	0.122 ± 0.004	44352 ± 3794	1.242			
Atropine methyl bromide	0.096 ± 0.003	42770 ± 1497	1.198			
Atropine methyl nitrate	0.101 ± 0.003	40300 ± 3335	1.128			
Atropine oxide	0.106 ± 0.002	17483 ± 458	0.504			
Atropine sulfate	0.100 ± 0.001	34608 ± 3450	0.969			
Aucubin	0.101 ± 0.008	20906 ± 233	0.621			
Auranofin	0.081 ± 0.008 **					
Aurantiamide	0.121 ± 0.005	32435 ± 4338	0.908			
Auraptene	0.124 ± 0.002	36045 ± 1306	1.009			
Auraptenol	0.121 ± 0.010	20432 ± 1118	0.571			
Aureothin	0.105 ± 0.012	55631 ± 4100	1.638			
Aurin tricarboxylic acid	0.108 ± 0.005	37072 ± 1369	1.038			
Aurintricarboxylic acid	0.100 ± 0.001	36044 ± 1383	1.009			
Aurora Kinase Inhibitor II	0.125 ± 0.013	33853 ± 1003	0.948			
Aurora Kinase Inhibitor III	0.123 ± 0.026	28538 ± 1610	0.799			
Aurora Kinase/Cdk Inhibitor	0.111 ± 0.010	32737 ± 2431	0.917			

Aurothioglucose	0.103 ±0.008	67596 ±4610	1.868			
Australine hydrochloride	0.115 ±0.011	18249 ±2761	0.542			
Austricin	0.098 ±0.003 *					
Autophagy Inhibitor, 3-MA	0.096 ±0.001	27512 ±493	0.770			
AV-412	0.114 ±0.003	33091 ±1627	0.927			
AV-951(KRN951,Tivozanib)	0.137 ±0.019	33157 ±3852	0.928			
Avermectin a1a	0.107 ±0.012	28638 ±1104	0.802			
Avermectin B1a	0.113 ±0.006	36690 ±1282	1.027			
Avicularin	0.122 ±0.017	46087 ±3043	1.290			
Avobenzone	0.108 ±0.005	30028 ±2004	0.841			
Avocadene	0.111 ±0.013	44275 ±5017	1.240			
Avocadene acetate	0.109 ±0.004	37931 ±5681	1.062			
Avocadyne acetate	0.102 ±0.016	19297 ±1492	0.540			
Avocadynofuran	0.109 ±0.007	36438 ±2890	1.020			
Avocatin a	0.094 ±0.003 *					
Avocatin b	0.099 ±0.003	34411 ±3368	0.963			
AWD-19211	0.110 ±0.006	32242 ±877	0.903			
Axillaridine A	0.106 ±0.009	25825 ±898	0.723			
Axitinib	0.123 ±0.003	34849 ±2217	0.976			
AY 9944	0.093 ±0.004	19051 ±552	0.557			
AY-NH_2	0.109 ±0.001	23743 ±1554	0.665			
AZ 10417808	0.104 ±0.004	30159 ±3825	0.844			
AZ-960	0.117 ±0.004	38741 ±1776	1.085			
Azacitidine	0.089 ±0.001 **					
Azacyclonol	0.118 ±0.005	33367 ±2939	0.934			
Azacytidine-5	0.092 ±0.002 **					
Azadirachtin	0.098 ±0.005	37937 ±1609	1.062			
Azaguanine-8	0.120 ±0.009	29201 ±2095	0.818			
Azaperone	0.117 ±0.002	40762 ±3382	1.141			
Azapropazone	0.118 ±0.004	42738 ±5040	1.197			
Azaserine	0.108 ±0.012	23151 ±2642	0.648			
Azasetron	0.132 ±0.003 *					

Azatadine maleate	0.098 ± 0.002	32315 ± 4955	0.905			
Azathioprine	0.094 ± 0.001	35504 ± 3047	0.994			
AZD6244(ARRY-142886)	0.122 ± 0.002	39032 ± 864	1.093			
AZD7762	0.128 ± 0.009	33510 ± 4645	0.938			
Azelaic acid	0.099 ± 0.001	88058 ± 4467	2.434	0.099 ± 0.008	95855 ± 6450	2.933
Azelastine hydrochloride	0.121 ± 0.006	137187 ± 20023	3.820	0.119 ± 0.002	54172 ± 4826	1.658
Azilsartan kamedoxomil	0.098 ± 0.003	29827 ± 1164	0.835			
Azithromycin	0.085 ± 0.006 **					
Azlocillin sodium	0.098 ± 0.008	18692 ± 1022	0.538			
Azobenzene	0.107 ± 0.004	36949 ± 3084	1.035			
Azomycin	0.076 ± 0.002 **					
Aztreonam	0.109 ± 0.001	41523 ± 1764	1.163			
b,g-Methyleneadenosine 5'-triphosphate 2Na	0.099 ± 0.007	39218 ± 4961	1.098			
B2	0.102 ± 0.005	29236 ± 2590	0.819			
B4-Rhodanine	0.122 ± 0.005	36065 ± 2030	1.010			
B581	0.124 ± 0.008	97769 ± 10012	2.971	0.121 ± 0.007	41228 ± 774	1.262
Bacampicillin hydrochloride	0.132 ± 0.020	40470 ± 4041	1.133			
Baccatin III	0.100 ± 0.013	162473 ± 1916	4.825	0.111 ± 0.040	29255 ± 2685	0.894
Baccatin IV	0.116 ± 0.010	32077 ± 317	0.898			
Bacitracin	0.118 ± 0.007	32460 ± 5002	0.909			
Baclofen	0.111 ± 0.003	20634 ± 1421	0.578			
Baclofen (R,S)	0.118 ± 0.007	40612 ± 196	1.137			
Badge	0.106 ± 0.007	29807 ± 1848	0.835			
Bafilomycin A1	0.109 ± 0.004	27860 ± 450	0.780			
Baicalein	0.113 ± 0.010	13540 ± 1205	0.402	0.115 ± 0.005	26157 ± 3482	0.800
Baicalin	0.109 ± 0.003	32410 ± 2558	0.907			
Bakuchiol	0.104 ± 0.002	47139 ± 5103	1.320			
b-Alanine	0.103 ± 0.011	33264 ± 6704	0.931			
Balanophonin	0.113 ± 0.008	32137 ± 5784	0.900			
Balsalazide	0.134 ± 0.004 **					
Balsalazide Sodium	0.110 ± 0.021	38705 ± 3764	1.084			
BAM(8-22)	0.109 ± 0.002	29002 ± 1585	0.812			

Bambuterol	0.113 ± 0.006	53893 ± 2299	1.509			
Bambuterol hydrochloride	0.120 ± 0.008	50480 ± 987	1.378			
Ban orl 24	0.118 ± 0.002	31169 ± 1492	0.873			
BAPTA-AM	0.116 ± 0.004	42042 ± 1604	1.177			
Barbacarpan	0.124 ± 0.009	58606 ± 7112	1.641			
Barbaloin	0.107 ± 0.012	30448 ± 2640	0.853			
Barbital	0.108 ± 0.006	36558 ± 1769	1.024			
Barpisoflavone A	0.122 ± 0.001	30547 ± 1789	0.855			
Batyl alcohol	0.127 ± 0.003 *					
Bauerenol acetate	0.117 ± 0.018	37801 ± 176	1.058			
Bavachin	0.119 ± 0.002	44482 ± 5518	1.245			
Bavachinin	0.123 ± 0.001	34797 ± 2277	0.974			
Bavachinin A	0.113 ± 0.006	28089 ± 519	0.786			
Bax Channel Blocker	0.098 ± 0.001	38176 ± 1908	1.069			
Bay 11-7082	0.058 ± 0.001 **					
Bay 11-7085	0.063 ± 0.004 **					
Bay 11-7821	0.059 ± 0.005 **					
Bay 41-2272	0.126 ± 0.013	31918 ± 1077	0.894			
Bay K-8644	0.114 ± 0.001	50789 ± 4292	1.543			
Bay-1518	0.111 ± 0.003	41532 ± 1121	1.163			
Bbenzyl benzoate	0.107 ± 0.006	33474 ± 3347	0.937			
BBMP	0.061 ± 0.010 **					
b-Casomorphin (human)	0.108 ± 0.008	37332 ± 1185	1.045			
B-Cell Immunosuppressant	0.095 ± 0.002	33175 ± 2167	0.929			
Bcr-abl Inhibitor,GNF-2	0.119 ± 0.009	28893 ± 2736	0.809			
BD 1008	0.105 ± 0.006	26602 ± 5199	0.745			
BD 1008 Dihydrobromide	0.103 ± 0.011	24270 ± 1226	0.680			
BD 1047 Dihydrobromide	0.113 ± 0.005	24466 ± 3549	0.685			
BD 1063 Dihydrochloride	0.111 ± 0.010	27743 ± 1302	0.777			
Beclamide	0.115 ± 0.005	49958 ± 1238	1.399			
Beclomethasone	0.108 ± 0.004	37934 ± 5043	1.062			
Beclomethasone dipropionate	0.114 ± 0.006	40698 ± 1390	1.140			

Befuraline	0.104 ± 0.007	26324 ± 689	0.737			
Bekanamycin sulfate	0.117 ± 0.029	28929 ± 1816	0.810			
Bemegride	0.109 ± 0.002	45792 ± 5978	1.282			
Bemotrizinol	0.103 ± 0.005	51085 ± 1614	1.430			
Benactyzine hydrochloride	0.131 ± 0.004 *					
Benaxibine	0.102 ± 0.020	49000 ± 4770	1.372			
Benazepril hydrochloride	0.102 ± 0.002	19815 ± 233	0.548			
Benazoline oxalate	0.093 ± 0.003	39001 ± 511	1.092			
Bendazol	0.113 ± 0.005	47355 ± 7217	1.326			
Bendroflumethiazide	0.119 ± 0.007	29911 ± 1292	0.837			
Benfluorex hydrochloride	0.116 ± 0.002	46547 ± 3113	1.303			
Benfotiamine	0.113 ± 0.002	41678 ± 1055	1.167			
Benidipine hydrochloride	0.124 ± 0.003	217887 ± 6419	6.067	0.122 ± 0.006	35931 ± 737	1.100
Benorin	0.120 ± 0.008	22398 ± 5586	0.627			
Benoxathian hydrochloride	0.098 ± 0.003	41773 ± 1393	1.170			
Benoxinate hydrochloride	0.112 ± 0.006	60953 ± 1501	1.663			
Benperidol	0.109 ± 0.007	30828 ± 340	0.863			
Benproperine phosphate	0.127 ± 0.006	45255 ± 402	1.267			
Benserazide hydrochloride	0.121 ± 0.001	45254 ± 2432	1.267			
Benurestat	0.112 ± 0.006	34872 ± 3904	0.976			
Benzalkonium chloride	0.055 ± 0.005 **					
Benzamide	0.101 ± 0.002	37278 ± 1633	1.044			
Benzamidine hydrochloride	0.102 ± 0.004	37712 ± 2735	1.056			
Benzamil	0.112 ± 0.033	18918 ± 514	0.530			
Benzamil hydrochloride	0.106 ± 0.002	29530 ± 1810	0.827			
Benzanthrone	0.109 ± 0.011	41057 ± 1379	1.150			
Benzathine benzylpenicillin	0.123 ± 0.004	22725 ± 4229	0.636			
Benzbromarone	0.110 ± 0.006	48751 ± 3600	1.365			
Benzeneacetic acid, 2-[(2,6-dichlorophenyl)-amino]-, monosodium salt	0.111 ± 0.007	33696 ± 2529	0.943			
Benzeneacetic acid, α-(hydroxymethyl)-, 9-methyl-3-oxa-9-azatricyclo[3.3.1.02,4]non-7-yl ester, [7(S)-(1α,2ÃÂ¿,4ÃÂ¿,5α,7ÃÂ¿)]	0.120 ± 0.005	39482 ± 3317	1.105			

Benzeneacetonitrile, α-[3-[[2-(3,4-dimethoxyphenyl)-ethyl]methylamino]propyl]-3,4-dimethoxy-α-(1-methylethyl)-, ®	0.113 ± 0.004	43789 ± 3930	1.226			
Benzeneethanamine, N,α-dimethyl-N-2-propynyl-(R)	0.104 ± 0.002	34211 ± 3113	0.958			
Benzethonium chloride	0.055 ± 0.004 **					
Benzetimide	0.098 ± 0.008	27216 ± 2316	0.762			
Benzo[a]phenanthridine-10,11-diol, 5,6,6a,7,8,12b-hexahydro-, trans	0.135 ± 0.008	48814 ± 148	1.367			
Benzocaine	0.117 ± 0.003	43287 ± 2588	1.212			
Benzoclidine	0.111 ± 0.007	52014 ± 7834	1.456			
Benzoic acid	0.113 ± 0.007	37516 ± 3087	1.050			
Benzomopine	0.106 ± 0.014	39273 ± 4715	1.100			
Benzonatate	0.120 ± 0.008	39266 ± 4155	1.099			
Benzoquinonium 2bromide	0.101 ± 0.010	32902 ± 4942	0.921			
Benzoxiquine	0.117 ± 0.006	39225 ± 5171	1.098			
Benzoyl peroxide	0.113 ± 0.003	48148 ± 3601	1.348			
Benzoylpas	0.107 ± 0.010	28294 ± 447	0.792			
Benzthiazide	0.108 ± 0.005	44950 ± 5553	1.259			
Benztropine hydrobromide	0.110 ± 0.003	44551 ± 3792	1.247			
Benztropine mesylate	0.109 ± 0.004	31231 ± 1885	0.874			
Benzydamine	0.137 ± 0.017	17542 ± 2485	0.526			
Benzydamine hydrochloride	0.118 ± 0.002	39712 ± 3644	1.112			
Benzyl alcohol	0.107 ± 0.006	35422 ± 1677	0.992			
Benzyl isothiocyanate	0.114 ± 0.005	26748 ± 2264	0.749			
Benzylpenicillin	0.072 ± 0.027 **					
Benzylpenicillin sodium	0.121 ± 0.002	12728 ± 1145	0.347	0.113 ± 0.002	31045 ± 2390	0.950
Benzylphosphonic acid	0.120 ± 0.004	32796 ± 514	0.918			
Bephenium hydroxynapthoate	0.101 ± 0.005	62822 ± 7272	1.810			
Bepridil	0.104 ± 0.009	63779 ± 4837	1.938			
Bepridil hydrochloride	0.111 ± 0.002	30993 ± 1267	0.868			
Berbamine	0.118 ± 0.002	24834 ± 747	0.695			
Berbamine dihydrochloride	0.109 ± 0.002	44229 ± 2155	1.238			
Berberine	0.115 ± 0.004	33972 ± 2539	0.951			
Berberine chloride	0.107 ± 0.007	39233 ± 704	1.099			

Bergapten	0.112 ±0.003	26733 ±2332	0.749			
Bergaptol	0.127 ±0.024	21870 ±1665	0.612			
Bergenin	0.126 ±0.002	41859 ±2268	1.172			
Bestatin	0.112 ±0.003	64254 ±5559	1.789			
Bestatin hydrochloride	0.103 ±0.004	39023 ±1331	1.093			
Betahistine	0.111 ±0.003	31202 ±5224	0.874			
Betahistine hydrochloride	0.110 ±0.005	21396 ±1408	0.599			
Betahistine mesylate	0.114 ±0.008	56635 ±2664	1.546			
Betaine aldehyde chloride	0.107 ±0.004	39549 ±1809	1.107			
Betaine hydrochloride	0.101 ±0.003	31980 ±946	0.895			
Betamethasone	0.107 ±0.006	44227 ±1805	1.238			
Betamethasone 17,21-dipropionate	0.102 ±0.007	27834 ±4400	0.779			
Betamethasone acetate	0.102 ±0.003	22224 ±6355	0.622			
Betamethasone dipropionate	0.115 ±0.018	44202 ±4227	1.238			
Betamethasone sodium phosphate	0.086 ±0.002 **					
Betamethasone valerate	0.113 ±0.003	32418 ±5865	0.908			
Betamipron	0.118 ±0.007	30801 ±1063	0.862			
Betaxalol hydrochloride	0.106 ±0.003	43168 ±4110	1.209			
Betaxolol hydrochloride	0.116 ±0.010	65171 ±1333	1.743			
Betazole hydrochloride	0.113 ±0.008	53140 ±7857	1.488			
Bethanechol chloride	0.105 ±0.006	41389 ±3430	1.159			
Betulin	0.122 ±0.022	29071 ±994	0.814			
Betulin caffeate	0.125 ±0.003	27667 ±957	0.775			
Betulin palmitate	0.123 ±0.003	39077 ±1232	1.094			
Betulinic acid	0.111 ±0.007	17376 ±967	0.516			
Betulonic acid	0.113 ±0.001	44237 ±2456	1.239			
Bexarotene	0.113 ±0.002	36293 ±1622	1.016			
BEZ-235	0.136 ±0.003 **					
Bezafibrate	0.133 ±0.012	52724 ±2001	1.476			
BF 2649	0.116 ±0.005	33249 ±1362	0.931			
BF-170 hydrochloride	0.109 ±0.005	68550 ±5100	1.919			
BHA	0.118 ±0.006	31182 ±6286	0.873			

BHQ	0.102 ± 0.004	23191 ± 4343	0.649			
BHT	0.109 ± 0.001	42587 ± 6558	1.192			
B-HT 920 dihydrochloride	0.112 ± 0.007	28911 ± 293	0.809			
B-HT 933 dihydrochloride	0.102 ± 0.007	36919 ± 2182	1.034			
BI 78D3	0.060 ± 0.003 **					
BI-2536 (R-)	0.120 ± 0.004	45361 ± 4252	1.270			
BIA 2-093	0.111 ± 0.010	55183 ± 5968	1.525			
BIBU 1361 dihydrochloride	0.117 ± 0.007	25500 ± 2373	0.714			
BIBX 1382 dihydrochloride	0.114 ± 0.001	26319 ± 1032	0.737			
Bicalutamide	0.103 ± 0.005	31276 ± 964	0.876			
Bicuculline (+)	0.117 ± 0.004	27929 ± 2182	0.782			
Bicuculline(-)-methiodide	0.107 ± 0.006	32351 ± 1248	0.906			
BI-D1870	0.115 ± 0.002	33900 ± 1492	0.949			
Bifemelane	0.113 ± 0.006	21962 ± 1468	0.611			
Bifemelane hydrochloride	0.109 ± 0.007	22433 ± 3311	0.628			
Bifonazole	0.112 ± 0.002	35167 ± 7611	0.985			
Bi-linderone	0.124 ± 0.016	39368 ± 3842	1.102			
Bilirubin	0.115 ± 0.012	27573 ± 1680	0.772			
Bilobalide	0.113 ± 0.008	18962 ± 1451	0.563			
Bilobetin	0.137 ± 0.006	49704 ± 6235	1.392			
BIO	0.097 ± 0.009	50549 ± 1566	1.397			
Biochanin A	0.123 ± 0.004	42003 ± 1444	1.176			
Biondinin C	0.123 ± 0.004	41536 ± 1723	1.163			
Biotin	0.111 ± 0.006	44242 ± 1431	1.239			
Biperiden	0.108 ± 0.002	34334 ± 3677	0.961			
Biperiden hydrochloride	0.106 ± 0.004	33770 ± 3993	0.946			
Bis(2-ethylhexyl)-phthalate	0.127 ± 0.005	30716 ± 763	0.860			
Bisabolol	0.111 ± 0.008	38284 ± 2301	1.072			
Bisacodyl	0.114 ± 0.001	36072 ± 1206	1.010			
Bisandrographolide A	0.124 ± 0.003	41732 ± 2773	1.168			
Bisanhydrorutilantinone	0.110 ± 0.003	39595 ± 4300	1.109			
Bisdehydrotuberostemonine	0.122 ± 0.003	25796 ± 1054	0.722			

Bis-demethoxycurcumin	0.108 ±0.003	45641 ±2814	1.278			
Bisindolylmaleimide I	0.116 ±0.002	23801 ±760	0.666			
Bisindolylmaleimide III, Hydrochloride	0.120 ±0.014	28468 ±1453	0.797			
Bisindolylmaleimide IV	0.119 ±0.023	23567 ±1371	0.660			
Bisindolylmaleimide V	0.128 ±0.004	33817 ±2461	0.947			
Bisindoylmalei mide X, hydrochloride salt	0.119 ±0.004	23571 ±946	0.660			
Bismuth subsalicylate	0.103 ±0.016	48653 ±5917	1.362			
Bisoctrizole	0.099 ±0.006	36886 ±1391	1.033			
Bisoprolol fumarate	0.119 ±0.003	70028 ±5456	1.950			
Bisoprolol hemifumarate salt	0.110 ±0.011	34472 ±3061	0.965			
Bisphenol a	0.101 ±0.003	33557 ±3002	0.940			
Bissalicyl fumarate	0.099 ±0.006	29577 ±749	0.828			
Bithionate sodium	0.061 ±0.008 **					
Bitoscanate	0.115 ±0.001	44402 ±2226	1.243			
BIX 01294 trihydrochloride hydrate	0.105 ±0.004	24804 ±1129	0.694			
BIX-01294	0.113 ±0.006	21361 ±3608	0.598			
Bixin	0.101 ±0.003	34521 ±1411	0.967			
Blebbistatin	0.109 ±0.003	28041 ±2805	0.785			
Bleomycin	0.063 ±0.002 **					
Bleomycin sulfate	0.065 ±0.001 **					
Blumeatin	0.114 ±0.010	33636 ±3144	0.942			
Blumeatin B	0.118 ±0.001	51096 ±6089	1.431			
Blumenol B	0.123 ±0.004	37650 ±1116	1.054			
Blumenol C glucoside	0.105 ±0.006	34706 ±1774	0.972			
BML-190	0.115 ±0.002	43917 ±6426	1.230			
BML-210	0.105 ±0.011	28389 ±1172	0.795			
BML-244	0.106 ±0.003	32116 ±1801	0.899			
BML-257	0.120 ±0.010	36062 ±1435	1.010			
BML-259	0.120 ±0.008	29062 ±1594	0.814			
BML-260	0.116 ±0.002	32895 ±1469	0.921			
BML-265 (Erlotinib analog)	0.075 ±0.006 **					
BML-266	0.125 ±0.012	23111 ±1164	0.647			

BML-267	0.121 ± 0.013	38936 ± 2473	1.090			
BML-268	0.122 ± 0.004	26691 ± 1210	0.747			
BML-278	0.107 ± 0.013	28370 ± 1327	0.794			
BML-281	0.101 ± 0.001	94018 ± 5111	2.916	0.106 ± 0.005	93659 ± 8706	2.866
BMS 182874 hydrochloride	0.108 ± 0.003	28884 ± 3083	0.809			
BMS 191011	0.113 ± 0.010	22390 ± 480	0.627			
BMS 649	0.113 ± 0.006	29789 ± 3557	0.834			
BMS-2	0.122 ± 0.003	36030 ± 2344	1.009			
BMS-3	0.136 ± 0.004 **					
BMS-5	0.122 ± 0.004	37229 ± 1382	1.042			
BMY 14802 hydrochloride	0.111 ± 0.007	33361 ± 1028	0.934			
BMY 45778	0.102 ± 0.006	39287 ± 8329	1.100			
BMY 7378 dihydrochloride	0.091 ± 0.004	53362 ± 2326	1.475			
BN-82002	0.118 ± 0.005	27207 ± 867	0.762			
BNTX Maleate	0.108 ± 0.005	27572 ± 3125	0.772			
Boc-GVV-CHO	0.114 ± 0.004	32658 ± 1169	0.914			
Boehmenan	0.126 ± 0.002	51918 ± 6103	1.454			
Bohemine	0.105 ± 0.003	28994 ± 2340	0.812			
Boldine	0.107 ± 0.001	18737 ± 552	0.556			
Bombiprenone	0.107 ± 0.008	36556 ± 2554	1.024			
Bonaphton	0.102 ± 0.005	37489 ± 1054	1.050			
Bongardol	0.112 ± 0.009	43781 ± 5253	1.226			
Bongkrekic acid	0.110 ± 0.004	25087 ± 2190	0.702			
Bopindolol malonate	0.115 ± 0.009	40329 ± 2607	1.129			
Borneol	0.112 ± 0.001	48198 ± 6683	1.350			
Bornyl acetate	0.109 ± 0.006	36471 ± 2384	1.021			
Borreriagenin	0.117 ± 0.001	46905 ± 5902	1.313			
Bortezomib	0.119 ± 0.012	65787 ± 6383	1.871			
Bosentan	0.098 ± 0.007	57315 ± 6167	1.605			
Bosutinib(SKI606)	0.119 ± 0.005	35286 ± 6956	0.988			
Bourjotinolone A	0.119 ± 0.003	31183 ± 1720	0.873			
Bovinocidin	0.095 ± 0.003	34398 ± 6995	0.963			

BP 554 maleate	0.112 ± 0.001	48794 ± 4640	1.366			
BP 897	0.101 ± 0.005	40323 ± 3802	1.129			
BPIQ-I	0.105 ± 0.006	27423 ± 1011	0.768			
Brachynoside heptaacetate	0.127 ± 0.012	39093 ± 350	1.095			
Brassinin	0.126 ± 0.008	169625 ± 3879	5.038	0.116 ± 0.003	101078±4155	3.093
Braylin	0.125 ± 0.005	42471 ± 3938	1.189			
Brazilin	0.120 ± 0.003	20512 ± 2109	0.574			
Brefeldin A	0.113 ± 0.008	19232 ± 649	0.571			
Brefeldin A from Penicillium brefeldianum	0.101 ± 0.004	40290 ± 2090	1.128			
Bretazenil	0.113 ± 0.008	39607 ± 2959	1.109			
Bretylium tosylate	0.100 ± 0.005	43946 ± 4500	1.230			
Brevilin A	0.123 ± 0.002	23475 ± 2573	0.657			
Brinzolamide	0.116 ± 0.003	45132 ± 892	1.264			
BRL 15572 hydrochloride	0.110 ± 0.008	39930 ± 2066	1.118			
BRL 37344 sodium	0.101 ± 0.002	38903 ± 3018	1.089			
BRL 44408 maleate	0.107 ± 0.001	32281 ± 2665	0.904			
BRL 50481	0.108 ± 0.008	32613 ± 2927	0.913			
BRL 52537 hydrochloride	0.109 ± 0.001	31396 ± 755	0.879			
BRL 54443	0.108 ± 0.008	33978 ± 5641	0.951			
BRL-52537	0.101 ± 0.009	38772 ± 2233	1.086			
Bromhexine hydrochloride	0.115 ± 0.002	65489 ± 4209	1.862			
Bromindione	0.112 ± 0.008	32915 ± 1372	0.922			
Bromo-3-hydroxy-4-(succin-2-yl)-caryolane γ-lactone	0.109 ± 0.007	44081 ± 4004	1.234			
Bromo-7-nitroindazole [3-Bromo-7-nitroindazole]	0.115 ± 0.006	57730 ± 2822	1.754			
Bromoacetyl alprenolol menthane	0.082 ± 0.006 **					
Bromoacetylcholine bromide	0.085 ± 0.009 *					
Bromo-cAMP [8-Bromo-cAMP]	0.107 ± 0.001	31589 ± 3622	0.884			
Bromo-cGMP [8-Bromo-cGMP]	0.107 ± 0.001	26077 ± 1554	0.730			
Bromocriptine mesylate	0.080 ± 0.006 **					
Bromocryptine mesylate	0.102 ± 0.006	18844 ± 3455	0.528			
Bromoenol lactone	0.096 ± 0.003	44377 ± 6463	1.243			
Bromolaudanosine, (±)-6'-	0.101 ± 0.007	45944 ± 82	1.364			

Bromopapaverine	0.113 ±0.007	34141 ±3648	0.956			
Bromopride	0.112 ±0.003	27215 ±1095	0.762			
Bromopropylate	0.105 ±0.008	63102 ±5698	1.858			
Bromotheamine	0.112 ±0.002	40864 ±5331	1.144			
Bromoxan	0.119 ±0.003	43296 ±2333	1.212			
Bromperidol	0.113 ±0.005	51314 ±2116	1.437			
Brompheniramine Maleate	0.108 ±0.013	37629 ±1804	1.054			
Bronopol	0.061 ±0.007 **					
Broussonin A	0.126 ±0.004	47338 ±3146	1.325			
Broussonin B	0.128 ±0.006	44312 ±764	1.241			
Broussonin E	0.122 ±0.007	69764 ±8402	1.950			
Broxaldine	0.099 ±0.006	44089 ±7553	1.234			
Broxuridine	0.112 ±0.023	1117 ±199	0.033	0.109 ±0.005	18062 ±2292	0.553
Broxyquinoline	0.109 ±0.009	39992 ±1589	1.120			
Brucine	0.124 ±0.005	31742 ±2923	0.889			
Brucine n-oxide	0.106 ±0.003	44611 ±633	1.325			
BR-Xanthone A	0.124 ±0.003	38030 ±4228	1.065			
Bryonolic acid	0.127 ±0.003	39146 ±2370	1.096			
BS-2-84	0.111 ±0.005	44786 ±3517	1.254			
BS-4-83	0.109 ±0.013	43252 ±5172	1.211			
BTCP hydrochloride	0.111 ±0.002	26418 ±3258	0.740			
BTCP maleate	0.107 ±0.007	30219 ±3009	0.846			
BTO-1	0.080 ±0.005 **					
BTS	0.105 ±0.004	64350 ±1012	1.883			
BTS 54-505	0.104 ±0.004	31274 ±2683	0.876			
BU 224 hydrochloride	0.107 ±0.011	34228 ±4861	0.958			
BU 226 hydrochloride	0.111 ±0.002	44264 ±1053	1.239			
BU 239 hydrochloride	0.111 ±0.002	35731 ±6045	1.000			
BU99006	0.092 ±0.006	38845 ±729	1.088			
Bucetin	0.109 ±0.009	35717 ±1697	1.000			
Bucladesine	0.109 ±0.002	31945 ±1035	0.894			
Bucladesine sodium salt	0.111 ±0.003	58730 ±5911	1.603			

Budesonide	0.097 ± 0.003	37438 ± 948	1.048			
Bufalin	0.106 ± 0.003	54800 ± 2662	1.628			
Bufexamac	0.113 ± 0.005	37198 ± 1659	1.042			
Buflomedil hydrochloride	0.114 ± 0.006	61766 ± 7081	1.720			
Bullatantriol	0.129 ± 0.010	33926 ± 7738	0.950			
Bulleyaconitine A	0.121 ± 0.003	38111 ± 2999	1.067			
Bulleyanin	0.115 ± 0.009	23308 ± 727	0.653			
Bumecaine	0.118 ± 0.004	33876 ± 3976	0.949			
Bumetanide	0.093 ± 0.004	43225 ± 5627	1.210			
Buphenine	0.110 ± 0.009	34769 ± 463	0.974			
Bupivacaine	0.105 ± 0.009	36536 ± 2134	1.023			
Bupivacaine hydrochloride	0.098 ± 0.008	31035 ± 3302	0.869			
Bupropion	0.109 ± 0.004	42296 ± 7234	1.184			
Bupropion hydrochloride	0.104 ± 0.002	40876 ± 3136	1.145			
Buquinolate	0.108 ± 0.010	43368 ± 2374	1.214			
Buramate	0.106 ± 0.003	25982 ± 1483	0.727			
Buspirone hydrochloride	0.108 ± 0.008	38093 ± 3893	1.067			
Bussein	0.110 ± 0.002	31981 ± 4687	0.895			
Busulfan	0.118 ± 0.005	41797 ± 1621	1.170			
Butabindide oxalate	0.113 ± 0.002	46514 ± 808	1.302			
Butacaine	0.106 ± 0.002	45869 ± 2218	1.284			
Butaclamol (+)	0.111 ± 0.001	49485 ± 6870	1.386			
Butalbital	0.114 ± 0.009	43383 ± 2799	1.215			
Butamben	0.121 ± 0.004	70377 ± 3014	1.921			
Butamirate Citrate	0.117 ± 0.014	24178 ± 1733	0.677			
Butein	0.077 ± 0.006 **					
Butenafine	0.084 ± 0.003 **					
Butenafine Hydrochloride	0.115 ± 0.014	27192 ± 2092	0.761			
Buthionine sulfoximine	0.114 ± 0.013	52334 ± 7377	1.465			
Butinoline	0.101 ± 0.013	36437 ± 5245	1.020			
Butirosin disulfate salt	0.117 ± 0.004	37938 ± 977	1.062			
Butoconazole	0.101 ± 0.004	23401 ± 1615	0.655			

Butoconazole nitrate	0.117 ±0.006	41069 ±5113	1.150			
Butyl paraben	0.105 ±0.009	54253 ±3965	1.519			
Butylated hydroxyanisole	0.110 ±0.005	63940 ±4879	1.842			
Butylated hydroxytoluene	0.112 ±0.010	29739 ±241	0.833			
Butylparaben	0.109 ±0.008	70170 ±11273	1.915			
Butylscopolammonium (n-)-bromide	0.115 ±0.006	43339 ±2004	1.213			
Butyl-β-carboline-3-carboxylate	0.121 ±0.008	47038 ±2034	1.317			
Butyrolactone 3	0.093 ±0.005	17455 ±829	0.541			
Butyrylcholine Cl	0.102 ±0.005	29958 ±3710	0.839			
Buxbodine B	0.135 ±0.008	33036 ±1131	0.925			
Buxbodine D	0.129 ±0.013	37315 ±1737	1.045			
BVT 948	0.082 ±0.002 **					
BW 284c51	0.106 ±0.002	34514 ±4817	0.966			
BW 373U86	0.108 ±0.006	33905 ±5706	0.949			
BW 723C86	0.101 ±0.009	31895 ±2564	0.893			
BW 723C86 hydrochloride	0.110 ±0.003	29744 ±3010	0.833			
BWB70C	0.093 ±0.007	44250 ±6141	1.239			
BX795	0.134 ±0.003 *					
BX912	0.098 ±0.003 *					
BYK 204165	0.114 ±0.005	29393 ±4024	0.823			
Byzantionoside B	0.115 ±0.001	38106 ±1504	1.067			
C-1	0.098 ±0.009	28432 ±1467	0.796			
C16 Ceramide	0.113 ±0.002	42898 ±1814	1.201			
C2 Ceramide	0.120 ±0.004	31751 ±2042	0.889			
C2 Dihydroceramide	0.131 ±0.013	28496 ±1147	0.798			
C2 Phytoceramide	0.089 ±0.004 **					
C5a Receptor Antagonist, W-54011	0.096 ±0.005	28344 ±855	0.794			
C6 Ceramide	0.117 ±0.008	30990 ±3788	0.868			
C8 Ceramine	0.110 ±0.006	26813 ±455	0.751			
C8 Dihydroceramide	0.108 ±0.003	33268 ±3294	0.931			
CA-074	0.114 ±0.004	33675 ±1623	0.943			
CA-074-Me	0.122 ±0.009	33880 ±1068	0.949			

CA-1001	0.103 ± 0.009	46710 ± 859	1.308			
Cabergoline	0.105 ± 0.006	32398 ± 4578	0.907			
Caboxine A	0.124 ± 0.004	34612 ± 3131	0.969			
Cabraleadiol	0.127 ± 0.003	40399 ± 684	1.131			
Cabraleadiol 3-acetate	0.112 ± 0.004	32393 ± 2615	0.907			
Cabraleahydroxylactone	0.129 ± 0.006	33377 ± 2588	0.935			
Cabraleahydroxylactone acetate	0.122 ± 0.003	41082 ± 953	1.150			
Cabralealactone	0.118 ± 0.008	39289 ± 4103	1.100			
Cabraleone	0.122 ± 0.005	47393 ± 5340	1.327			
Cabreuvin	0.108 ± 0.002	34610 ± 3224	0.969			
Cacodylic acid	0.102 ± 0.003	21311 ± 614	0.597			
Cadaverine tartrate	0.109 ± 0.005	32080 ± 2833	0.898			
Cadin-4-en-10-ol	0.120 ± 0.005	51816 ± 8920	1.451			
Cafestol	0.124 ± 0.004	51073 ± 2400	1.517			
Cafestol acetate	0.127 ± 0.003	33142 ± 1701	0.928			
Caffeic Acid	0.101 ± 0.006	43228 ± 2534	1.210			
Caffeic acid phenethyl ester	0.111 ± 0.005	71399 ± 2845	1.973			
Caffeine	0.107 ± 0.003	34889 ± 2264	0.977			
Cajanin	0.120 ± 0.016	30677 ± 208	0.859			
Calanolide E	0.135 ± 0.006	38089 ± 3041	1.066			
Calcifediol	0.115 ± 0.006	38711 ± 4007	1.084			
Calcimycin	0.098 ± 0.006	25751 ± 1977	0.721			
Calcipotriene	0.116 ± 0.012	34380 ± 1944	0.963			
Calcipotriol	0.113 ± 0.003	25199 ± 1771	0.706			
Calcitriol	0.112 ± 0.005	26759 ± 7159	0.749			
Calcium gluceptate	0.102 ± 0.005	25469 ± 5219	0.713			
Calmidazolium chloride	0.063 ± 0.003 **					
Calpain Inhibitor II	0.121 ± 0.005	35076 ± 1273	0.982			
Calpeptin	0.116 ± 0.006	34219 ± 1152	0.958			
Calphostin C	0.109 ± 0.001	29504 ± 2877	0.826			
Calycanthoside	0.124 ± 0.001	41434 ± 2635	1.160			
Calyciphylline A	0.117 ± 0.001	42073 ± 2771	1.178			

Calyculin A	0.109 ± 0.004	35473 ± 517	0.993			
Calyxamine B	0.129 ± 0.006	41852 ± 3073	1.172			
Calyxin B	0.107 ± 0.010	24705 ± 966	0.692			
Calyxin H	0.123 ± 0.012	31244 ± 2518	0.875			
Camaric acid	0.121 ± 0.004	31431 ± 2653	0.880			
Cambinol	0.103 ± 0.005	23285 ± 421	0.644			
CaMKII Inhibitor, CK59	0.112 ± 0.021	43918 ± 1742	1.230			
CaMKP Inhibitor	0.090 ± 0.002 *					
Camphor (1r)	0.098 ± 0.004	19256 ± 1947	0.539			
Camptothecin	0.109 ± 0.004	56383 ± 5177	1.579			
Camptothecine (S,+)	0.137 ± 0.016	25617 ± 3141	0.717			
Camylofine chlorhydrate	0.117 ± 0.007	47847 ± 1625	1.340			
Camylofine dihydrochloride	0.112 ± 0.007	23349 ± 891	0.654			
Canadine	0.120 ± 0.007	46780 ± 774	1.310			
Canavanine	0.107 ± 0.001	35195 ± 1435	0.985			
Candesartan	0.117 ± 0.009	33228 ± 2175	0.930			
Candesartan Cilexetil	0.100 ± 0.004	52265 ± 1135	1.539			
Candesartan Cilextil	0.102 ± 0.003	38854 ± 2603	1.088			
Candicidin	0.101 ± 0.003	30544 ± 1484	0.855			
Cannabichromene	0.134 ± 0.008	42625 ± 4588	1.193			
Cannabidiol	0.118 ± 0.012	41938 ± 4436	1.174			
Cannabidiolic acid	0.133 ± 0.008	52710 ± 7148	1.476			
Cannabigerol	0.118 ± 0.005	38557 ± 2428	1.080			
Cannabisin F	0.135 ± 0.002	29376 ± 467	0.823			
Cannabispiran	0.129 ± 0.002	65114 ± 9165	1.820			
Cannabispirol	0.126 ± 0.001	40616 ± 2480	1.137			
Canniprene	0.129 ± 0.004	38470 ± 4376	1.077			
Canrenoic acid potassium salt	0.115 ± 0.013	38069 ± 1503	1.066			
Canrenone	0.112 ± 0.004	58747 ± 4167	1.671			
Cantharidic acid	0.114 ± 0.003	29174 ± 2386	0.817			
Cantharidin	0.114 ± 0.006	27171 ± 806	0.761			
Canthaxanthin	0.113 ± 0.004	51534 ± 1283	1.439			

Canthin-6-one	0.115 ± 0.007	31849 ± 1062	0.892			
Canusesnol A	0.120 ± 0.012	29779 ± 4176	0.834			
CAPE	0.120 ± 0.009	58715 ± 4756	1.744			
Capecitabine	0.108 ± 0.004	35363 ± 7161	0.990			
Capobenic acid	0.111 ± 0.006	32507 ± 1488	0.910			
Capreomycin sulfate	0.104 ± 0.005	18383 ± 299	0.546			
Capsacin(E)	0.108 ± 0.003	46111 ± 1125	1.291			
Capsaicin	0.120 ± 0.011	28627 ± 1452	0.802			
Capsanthin	0.111 ± 0.001	34196 ± 361	0.957			
Capsazepine	0.107 ± 0.003	66154 ± 2614	1.852			
Captan	0.090 ± 0.003 **					
Captopril	0.115 ± 0.001	37007 ± 447	1.036			
Carabrolactone A	0.125 ± 0.007	44190 ± 1898	1.237			
Carabrolactone B	0.115 ± 0.005	22174 ± 1306	0.621			
Carabrone	0.109 ± 0.010	58753 ± 3715	1.642			
Caraphenol A	0.062 ± 0.008 **					
Carapin	0.109 ± 0.006	33568 ± 2375	0.940			
Carapin-8(9)-ene	0.109 ± 0.006	31782 ± 1053	0.890			
Carbacetam	0.086 ± 0.005 **					
Carbachol	0.099 ± 0.006	43242 ± 4702	1.211			
Carbacyclin	0.117 ± 0.011	32753 ± 4800	0.917			
Carbadox	0.108 ± 0.002	41299 ± 1991	1.156			
Carbamazepine	0.105 ± 0.008	39546 ± 1869	1.107			
Carbamylcholine Cl	0.099 ± 0.012	34163 ± 3778	0.957			
Carbamyl-β-Methylcholine Cl	0.112 ± 0.008	36981 ± 5451	1.035			
Carbarsone	0.111 ± 0.004	42607 ± 501	1.193			
Carbenicillin disodium	0.092 ± 0.005 *					
Carbenoxolone disodium salt	0.123 ± 0.006	21726 ± 1946	0.608			
Carbetapentane citrate	0.098 ± 0.011	32027 ± 2288	0.897			
Carbidopa	0.113 ± 0.008	52636 ± 3531	1.474			
Carbimazole	0.109 ± 0.010	31122 ± 2437	0.871			
Carbinoxamine maleate	0.093 ± 0.009	21006 ± 3860	0.588			

Carboplatin	0.064 ±0.007 **					
Carboxin	0.100 ±0.018	44345 ±5155	1.242			
Cardamonin	0.121 ±0.008	37152 ±2397	1.040			
Cardiogenol C	0.097 ±0.002	34978 ±634	0.979			
Cardiogenol C hydrochloride	0.117 ±0.004	43744 ±4000	1.225			
Carglumic acid	0.121 ±0.005	35746 ±3798	1.001			
Carisoprodol	0.099 ±0.005	30526 ±676	0.855			
Carminic acid	0.121 ±0.018	13222 ±346	0.393	0.119 ±0.016	57673 ±3725	1.765
Carmofur	0.099 ±0.010	6269 ±339	0.175	0.109 ±0.009	2901 ±115	0.089
Carmoxirole hydrochloride	0.110 ±0.017	34474 ±3798	0.965			
Carmustine	0.096 ±0.010	34056 ±2117	0.954			
Carnitine (dl)-hydrochloride	0.109 ±0.005	42639 ±3625	1.194			
Carnitine Cl, (±)-	0.117 ±0.008	21903 ±552	0.651			
Carnosic acid	0.102 ±0.004	45204 ±1964	1.266			
Carnosine	0.113 ±0.004	28514 ±866	0.798			
Caroverine hydrochloride	0.116 ±0.013	34957 ±6509	0.979			
Carpachromene	0.122 ±0.003	31363 ±455	0.878			
Carprofen	0.117 ±0.007	46460 ±4007	1.301			
Carsalam	0.119 ±0.003	21884 ±1588	0.613			
Carteolol hydrochloride	0.111 ±0.003	46373 ±2654	1.298			
Carvedilol	0.069 ±0.009 **					
Carvedilol phosphate	0.092 ±0.007 *					
Carylophyllene oxide	0.120 ±0.005	29075 ±3135	0.814			
Caryophyllene [t(-)]	0.115 ±0.008	33064 ±1327	0.926			
Caryophyllene oxide	0.098 ±0.003	41561 ±1088	1.164			
Carzenide	0.106 ±0.001	38234 ±3099	1.071			
Casanthranol	0.118 ±0.012	42398 ±3497	1.187			
Casein Kinase I Inhibitor, D4476	0.112 ±0.012	37974 ±4822	1.063			
Casein Kinase II Inhibitor I	0.069 ±0.007 **					
Casein Kinase II Inhibitor III, TBCA	0.101 ±0.002	30585 ±4285	0.856			
Casein Kinase II Inhibitor IV	0.108 ±0.005	23749 ±708	0.665			
Cassiachromone	0.121 ±0.003	38103 ±123	1.067			

Cassythicine	0.130 ± 0.007	30328 ± 2123	0.849			
Castanospermine	0.115 ± 0.008	19461 ± 1935	0.578			
Casticin	0.122 ± 0.004	40963 ± 4254	1.147			
Catalpalactone	0.115 ± 0.010	19739 ± 1132	0.552			
Catalpin	0.109 ± 0.003	20707 ± 1428	0.580			
Catalpol	0.107 ± 0.004	34194 ± 1223	0.957			
Catalponol	0.114 ± 0.006	44577 ± 4102	1.248			
Catalponol methylthiomethyl ether	0.115 ± 0.008	36475 ± 1664	1.021			
Catalposide	0.117 ± 0.007	38746 ± 3041	1.085			
Catechin	0.132 ± 0.002	30274 ± 656	0.848			
Catechin 3-rhamnoside	0.129 ± 0.004	29724 ± 761	0.832			
Catechin 7-xyloside	0.114 ± 0.016	25381 ± 1482	0.711			
Catechin hydrate, (+)-	0.102 ± 0.005	39005 ± 1451	1.092			
Catechin pentaacetate	0.126 ± 0.006	44785 ± 2221	1.254			
Catechin tetramethylether	0.111 ± 0.005	37388 ± 3595	1.047			
Catharanthine	0.116 ± 0.009	41446 ± 4589	1.160			
Catharanthine base	0.103 ± 0.004	34712 ± 3113	0.972			
Caudatin	0.130 ± 0.004	28593 ± 2147	0.801			
CB 1954	0.105 ± 0.005	36912 ± 1659	1.034			
CBFβ Inhibitor	0.103 ± 0.003	63570 ± 5805	1.860			
CBIQ	0.101 ± 0.003	43127 ± 4917	1.208			
CC-401	0.122 ± 0.006	35027 ± 2503	0.981			
CCG-2046	0.100 ± 0.008	41453 ± 1834	1.161			
CCG-4986	0.097 ± 0.002	22532 ± 4185	0.631			
CCMI	0.119 ± 0.004	45416 ± 3363	1.272			
CCMQ	0.113 ± 0.003	32262 ± 1311	0.903			
CCPA	0.125 ± 0.004	65379 ± 1839	1.820			
CCR2 Antagonist	0.099 ± 0.001	33399 ± 5505	0.935			
CCR4 Antagonist	0.097 ± 0.004	48937 ± 4677	1.370			
CCT 018159	0.110 ± 0.005	37074 ± 944	1.038			
CD 1530	0.110 ± 0.002	38404 ± 1246	1.075			
CD 437	0.107 ± 0.011	38090 ± 6083	1.066			

CDC	0.101 ± 0.006	29574 ± 3927	0.828			
Cdc2-Like Kinase Inhibitor, TG003	0.103 ± 0.003	26583 ± 1711	0.744			
Cdc7/Cdk9 Inhibitor	0.112 ± 0.012	30756 ± 829	0.861			
Cdk Inhibitor, p35	0.114 ± 0.005	36565 ± 3969	1.024			
Cdk/Crk Inhibitor	0.112 ± 0.006	30954 ± 3632	0.867			
Cdk1 Inhibitor	0.105 ± 0.003	39713 ± 2512	1.112			
Cdk1 Inhibitor IV, RO-3306	0.103 ± 0.016	39341 ± 1503	1.102			
Cdk1 Inhibitor, CGP74514A	0.111 ± 0.002	35769 ± 1571	1.002			
Cdk1/2 Inhibitor III	0.107 ± 0.010	31477 ± 2040	0.881			
Cdk1/5 Inhibitor	0.109 ± 0.008	17896 ± 3055	0.501			
Cdk2 Inhibitor II	0.118 ± 0.011	36058 ± 4478	1.010			
Cdk2 Inhibitor III	0.112 ± 0.010	33388 ± 1141	0.935			
Cdk2 Inhibitor IV, NU6140	0.110 ± 0.024	37311 ± 2011	1.045			
Cdk2/5 Inhibitor	0.130 ± 0.001	38278 ± 1014	1.072			
Cdk2/9 Inhibitor	0.121 ± 0.012	37254 ± 2781	1.043			
Cdk4 Inhibitor	0.118 ± 0.005	28797 ± 1111	0.806			
Cdk4 Inhibitor II, NSC 625987	0.109 ± 0.016	17898 ± 823	0.530			
Cdk4 Inhibitor III	0.112 ± 0.004	16994 ± 571	0.503			
Cdk9 Inhibitor II	0.123 ± 0.008	35934 ± 3812	1.006			
Ceanothic acid	0.121 ± 0.007	34628 ± 4598	0.970			
Ceanothic acid acetate	0.122 ± 0.003	34944 ± 1386	0.978			
Cearoin	0.102 ± 0.009	28197 ± 754	0.790			
Cedrelone	0.132 ± 0.029	42828 ± 4888	1.199			
Cedrol	0.100 ± 0.004	30687 ± 849	0.859			
Cedrusin	0.118 ± 0.010	41959 ± 6220	1.175			
Cedryl acetate	0.108 ± 0.004	34557 ± 3154	0.968			
Cefaclor	0.107 ± 0.007	43209 ± 3876	1.210			
Cefaclor hydrate	0.111 ± 0.004	38110 ± 5275	1.067			
Cefadroxil	0.110 ± 0.001	28422 ± 2726	0.796			
Cefalonium	0.105 ± 0.007	33613 ± 2593	0.941			
Cefalotin	0.062 ± 0.003 **					
Cefamandole nafate	0.068 ± 0.007 **					

Cefamandole sodium	0.101 ± 0.003	38206 ± 2513	1.070			
Cefatrizine propylene glycol	0.124 ± 0.005	68452 ± 7955	1.906			
Cefazolin	0.100 ± 0.005	19810 ± 2471	0.555			
Cefazolin sodium	0.100 ± 0.005	35973 ± 1994	1.007			
Cefdinir	0.111 ± 0.011	70029 ± 6372	1.950			
Cefditorin pivoxil	0.106 ± 0.001	33905 ± 2355	0.949			
Cefepime	0.068 ± 0.009 **					
Cefepime hydrochloride	0.050 ± 0.015 **					
Cefixime	0.061 ± 0.006 **					
Cefixime trihydrate	0.128 ± 0.011	9295 ± 655	0.259	0.113 ± 0.006	19674 ± 826	0.602
Cefmenoxime hydrochloride	0.052 ± 0.003 **					
Cefmetazole sodium	0.103 ± 0.008	37958 ± 4154	1.063			
Cefonicid sodium	0.103 ± 0.011	27848 ± 621	0.780			
Cefoperazone	0.137 ± 0.014	18363 ± 1208	0.529			
Cefoperazone acid	0.104 ± 0.006	39281 ± 959	1.100			
Cefoperazone dihydrate	0.112 ± 0.003	38471 ± 4420	1.077			
Ceforanide	0.057 ± 0.012 **					
Cefotaxime acid	0.060 ± 0.001 **					
Cefotaxime sodium salt	0.051 ± 0.004 **					
Cefotetan	0.127 ± 0.009	25973 ± 237	0.727			
Cefotiam hydrochloride	0.080 ± 0.005 **					
Cefoxitin sodium	0.094 ± 0.006 *					
Cefpiramide	0.101 ± 0.001	17754 ± 1236	0.511			
Cefpodoxime proxetil	0.128 ± 0.010	35798 ± 1741	1.002			
Cefprozil	0.110 ± 0.007	26212 ± 1543	0.734			
Cefsulodin sodium	0.117 ± 0.004	21199 ± 3553	0.611			
Ceftazidime	0.076 ± 0.013 **					
Ceftazidime pentahydrate	0.101 ± 0.008	12051 ± 648	0.329	0.112 ± 0.007	36912 ± 965	1.130
Ceftibuten	0.108 ± 0.004	34843 ± 255	0.976			
Ceftiofur hydrochloride	0.056 ± 0.002 **					
Ceftriaxone sodium	0.055 ± 0.005 **					
Cefuroxime axetil	0.115 ± 0.013	23171 ± 2483	0.649			

Cefuroxime sodium	0.069 ± 0.002 **					
Celaphanol A	0.116 ± 0.002	36015 ± 2312	1.008			
Celastrol	0.104 ± 0.004	35680 ± 3635	0.999			
Celecoxib	0.114 ± 0.006	36094 ± 1566	1.011			
Celiprolol hydrochloride	0.108 ± 0.005	33678 ± 751	0.943			
Cell Sheet Migration Inhibitor, Locostatin	0.097 ± 0.003	18150 ± 1573	0.531			
Cellobiose (d[+])	0.115 ± 0.006	33386 ± 2844	0.935			
Centrolobol	0.118 ± 0.003	49375 ± 3065	1.382			
Centrophenoxine hydrochloride	0.100 ± 0.002	37662 ± 2512	1.055			
Cephalexin	0.101 ± 0.003	34324 ± 4182	0.961			
Cephalexin hydrate	0.100 ± 0.013	42837 ± 2898	1.199			
Cephalexin monohydrate	0.125 ± 0.002	24184 ± 706	0.677			
Cephalocyclidin A	0.117 ± 0.003	34299 ± 1431	0.960			
Cephalosporanic acid, 7-amino	0.112 ± 0.005	45027 ± 1439	1.261			
Cephalosporin c sodium	0.114 ± 0.011	35403 ± 1129	0.991			
Cephalothin sodium	0.090 ± 0.007	33354 ± 4117	0.934			
Cephapirin sodium	0.108 ± 0.003	33546 ± 2620	0.939			
Cepharanthine	0.125 ± 0.005	29873 ± 1339	0.836			
Cephradine	0.107 ± 0.010	39298 ± 4481	1.100			
Ceramide	0.113 ± 0.008	40245 ± 6144	1.127			
Cerberic acid	0.125 ± 0.018	39678 ± 4190	1.111			
Cerberidol	0.123 ± 0.002	32656 ± 2143	0.914			
Cerbinal	0.124 ± 0.005	33375 ± 3024	0.934			
Cerebroside B	0.134 ± 0.005	38442 ± 2045	1.076			
Cerevisterol	0.119 ± 0.024	31802 ± 5480	0.890			
Cerivastatin	0.108 ± 0.002	29648 ± 2766	0.830			
Cerivastatin Na	0.112 ± 0.003	40264 ± 4588	1.127			
Cerulenin	0.101 ± 0.018	23417 ± 1557	0.656			
Cetirizine dihydrochloride	0.105 ± 0.008	37620 ± 848	1.053			
Cetirizine hydrochloride	0.105 ± 0.003	34469 ± 2175	0.965			
Cetraxate hydrochloride	0.129 ± 0.011	59275 ± 8490	1.660			
Cetrimonium bromide	0.063 ± 0.003 **					

Cetylpyridinium chloride	0.066 ± 0.010 **					
Cevadine	0.115 ± 0.006	19642 ± 741	0.583			
CFM 1571 hydrochloride	0.119 ± 0.006	42449 ± 3662	1.189			
CFM-2	0.108 ± 0.003	35270 ± 1250	0.988			
cFMS Receptor Tyrosine Kinase Inhibitor	0.120 ± 0.003	28203 ± 2807	0.790			
CFTR-F508del Corrector, KM11060	0.103 ± 0.005	38978 ± 2650	1.091			
CFTRinh 172	0.113 ± 0.008	42019 ± 4290	1.177			
CGH 2466 Dihydrochloride	0.112 ± 0.006	33421 ± 2485	0.936			
CGK 733	0.116 ± 0.003	35286 ± 2062	0.988			
CGP 12177 Hydrochloride	0.119 ± 0.007	34186 ± 1180	0.957			
CGP 13501	0.115 ± 0.003	42578 ± 3686	1.192			
CGP 20712A Methanesulfonate	0.100 ± 0.011	45232 ± 3477	1.266			
CGP 35348	0.104 ± 0.008	30281 ± 2054	0.848			
CGP 37157	0.115 ± 0.004	48885 ± 2794	1.369			
CGP 37849	0.110 ± 0.002	27200 ± 1793	0.762			
CGP 39551	0.108 ± 0.005	52041 ± 7544	1.457			
CGP 46381	0.103 ± 0.013	27625 ± 537	0.773			
CGP 52411	0.112 ± 0.002	60143 ± 4669	1.664			
CGP 52432	0.104 ± 0.002	28897 ± 1989	0.809			
CGP 53353	0.105 ± 0.007	33639 ± 2002	0.942			
CGP 54626 hydrochloride	0.113 ± 0.003	31442 ± 1716	0.880			
CGP 55845	0.101 ± 0.008	31836 ± 2507	0.891			
CGP 57380	0.110 ± 0.004	33134 ± 1492	0.928			
CGP 71683 hydrochloride	0.105 ± 0.002	13330 ± 979	0.369	0.116 ± 0.009	33862 ± 3382	1.036
CGP 74514A hydrochloride	0.098 ± 0.006	49730 ± 3636	1.392			
CGP 78608	0.112 ± 0.014	40967 ± 5334	1.147			
CGP 7930	0.109 ± 0.010	45563 ± 5555	1.276			
CGS 12066A maleate	0.078 ± 0.012 *					
CGS 12066B dimaleate	0.078 ± 0.007 **					
CGS 15943	0.089 ± 0.005 *					
CGS 19755	0.106 ± 0.006	39565 ± 5896	1.108			
CGS 20625	0.108 ± 0.004	25190 ± 2195	0.705			

CGS 21680 hydrochloride	0.097 ±0.006	41411 ±5068	1.159			
CGS 9343B	0.104 ±0.007	31890 ±2022	0.893			
CH 223191	0.112 ±0.003	33214 ±587	0.930			
CH 55	0.110 ±0.004	32657 ±5234	0.914			
Chaconine,a -	0.100 ±0.004	17789 ±656	0.528			
Chaetomellic acid A	0.090 ±0.002 **					
Chalcone	0.119 ±0.007	27072 ±1638	0.758			
Chartreusin	0.093 ±0.004 *					
Chaulmoogric acid	0.114 ±0.005	33788 ±2650	0.946			
Chaulmosulfone	0.103 ±0.007	42635 ±1128	1.194			
Chelerythrine	0.104 ±0.003	42207 ±706	1.182			
Chelerythrine chloride	0.075 ±0.005 **					
Chelidamic acid	0.104 ±0.005	39258 ±1790	1.099			
Chelidonic acid	0.121 ±0.009	37767 ±7486	1.057			
Chelidonine, (+)-	0.101 ±0.017	22014 ±1071	0.616			
Chenodeoxycholic acid	0.115 ±0.005	49644 ±2603	1.390			
Chenodiol	0.121 ±0.005	43432 ±1503	1.216			
Chetomin, Chaetomium sp.	0.069 ±0.002 **					
Chicago sky blue	0.109 ±0.016	32408 ±3568	0.907			
Chicago sky blue 6B	0.110 ±0.006	19366 ±698	0.529			
Chinensine B	0.127 ±0.004	44431 ±7916	1.244			
Chiniofon	0.106 ±0.002	38275 ±2382	1.072			
CHIR-258(TKI-258)	0.122 ±0.017	32629 ±1646	0.914			
Chitin	0.103 ±0.011	34908 ±2897	0.977			
Chk2 Inhibitor	0.132 ±0.010	35719 ±1503	1.000			
Chk2 Inhibitor II	0.105 ±0.002	29902 ±3009	0.837			
Chlomizol	0.108 ±0.013	33536 ±5474	0.939			
Chloraholide D	0.121 ±0.009	26787 ±1179	0.750			
Chloralose	0.110 ±0.012	32644 ±1084	0.914			
Chlorambucil	0.100 ±0.004	28296 ±2735	0.792			
Chloramine-t	0.120 ±0.005	33923 ±761	0.950			
Chloramphenicol	0.078 ±0.001 **					

Chloramphenicol palmitate	0.131 ± 0.011	17393 ± 1308	0.501			
Chloramphenicol sodium succinate	0.112 ± 0.009	3859 ± 381	0.111	0.115 ± 0.006	31360 ± 3065	0.960
Chloranil	0.080 ± 0.009 **					
Chlorantholide A	0.120 ± 0.006	40646 ± 2144	1.138			
Chlorantholide B	0.117 ± 0.004	34602 ± 1353	0.969			
Chlorantholide C	0.119 ± 0.002	34548 ± 1883	0.967			
Chlorantholide D	0.123 ± 0.005	39176 ± 3619	1.097			
Chlorantholide E	0.116 ± 0.003	44269 ± 1567	1.240			
Chloranthоlide F	0.123 ± 0.005	41206 ± 2368	1.154			
Chlorazanil hydrochloride	0.108 ± 0.003	23271 ± 884	0.652			
Chlorcyclizine hydrochloride	0.117 ± 0.003	41258 ± 2281	1.155			
Chlordiazepoxine	0.118 ± 0.003	23338 ± 1699	0.653			
Chlorhexidine	0.056 ± 0.005 **					
Chlorhexidine dihydrochloride	0.078 ± 0.015 **					
Chlorindanol	0.093 ± 0.003 *					
Chlorindione	0.107 ± 0.009	40738 ± 1690	1.141			
Chlorisondamine diiodide	0.102 ± 0.009	13863 ± 607	0.371	0.110 ± 0.001	24767 ± 1009	0.758
Chlormadinone acetate	0.114 ± 0.004	48434 ± 3002	1.356			
Chlormethiazole hydrochloride	0.101 ± 0.007	28134 ± 2153	0.788			
Chlormezanone	0.103 ± 0.027	40952 ± 2702	1.147			
Chlormidazole	0.060 ± 0.020 **					
Chlorobutanol	0.109 ± 0.007	35524 ± 4439	0.995			
Chlorocresol	0.119 ± 0.006	37541 ± 2427	1.051			
Chloroethylclonidine dihydrochloride	0.103 ± 0.004	88535 ± 7537	2.367	0.103 ± 0.008	51473 ± 2855	1.575
Chlorogenic acid	0.105 ± 0.002	19397 ± 5329	0.543			
Chloroguanide hydrochloride	0.112 ± 0.003	36432 ± 638	1.020			
Chloro-IB-MECA	0.087 ± 0.007 *					
Chlorophyllide cu complex na salt	0.116 ± 0.004	37389 ± 2831	1.047			
Chloropyramine hydrochloride	0.112 ± 0.006	52383 ± 4151	1.467			
Chloroquine diphosphate	0.109 ± 0.004	32606 ± 1816	0.913			
Chloroquine phosphate	0.111 ± 0.003	39679 ± 2121	1.111			
Chlorothiazide	0.100 ± 0.004	35520 ± 2246	0.995			

Chlorotrianisene	0.115 ± 0.011	46435 ± 867	1.300			
Chloroxine	0.120 ± 0.006	60241 ± 3233	1.644			
Chloroxylenol	0.107 ± 0.007	44412 ± 4295	1.244			
Chlorpheniramine	0.100 ± 0.001	25915 ± 906	0.726			
Chlorpheniramine maleate	0.095 ± 0.017	38315 ± 6200	1.073			
Chlorphensin carbamate	0.122 ± 0.017	46041 ± 6117	1.289			
Chlorpromazine	0.108 ± 0.007	17578 ± 1528	0.506			
Chlorpromazine hydrochloride	0.123 ± 0.011	13424 ± 638	0.359	0.119 ± 0.003	32890 ± 2450	1.006
Chlorpropamide	0.094 ± 0.009	38389 ± 1026	1.075			
Chlorprothixene	0.120 ± 0.007	37160 ± 4162	1.040			
Chlorprothixene hydrochloride	0.105 ± 0.003	37448 ± 2181	1.049			
Chlorpyrifos	0.113 ± 0.007	51577 ± 1581	1.444			
Chlorquinaldol	0.116 ± 0.013	32185 ± 1934	0.901			
Chlortetracycline hydrochloride	0.089 ± 0.003 **					
Chlorthalidone	0.109 ± 0.003	52779 ± 3346	1.478			
Chlorzoxazone	0.100 ± 0.004	37663 ± 2377	1.055			
CHM-1 hydrate	0.097 ± 0.004	39539 ± 7375	1.107			
Cholecalciferol	0.109 ± 0.007	35672 ± 4191	0.999			
Cholest-4,6-dien-3-one	0.116 ± 0.007	40276 ± 2770	1.128			
Cholest-5-en-3-one	0.105 ± 0.005	51246 ± 5842	1.435			
Cholestan-3-one	0.109 ± 0.004	33949 ± 959	0.951			
Cholestane	0.116 ± 0.003	37772 ± 3824	1.058			
Cholesterol	0.120 ± 0.004	22588 ± 3513	0.632			
Cholesteryl acetate	0.107 ± 0.009	28765 ± 884	0.805			
Cholic acid	0.108 ± 0.007	35860 ± 2000	1.004			
Cholic acid, methyl ester	0.110 ± 0.003	39405 ± 3788	1.103			
Choline bromide	0.102 ± 0.005	37916 ± 892	1.062			
Choline chloride	0.103 ± 0.010	33661 ± 4833	0.942			
Chonglou Saponin I	0.121 ± 0.006	34074 ± 2101	0.954			
Chonglou Saponin VII	0.119 ± 0.004	30506 ± 5238	0.854			
CHPG	0.110 ± 0.006	27113 ± 1555	0.759			
CHR 2797	0.100 ± 0.008	27155 ± 2693	0.760			

Chromanol 293B	0.109 ±0.010	36782 ±1423	1.030			
Chromeceptin	0.095 ±0.003	22225 ±4348	0.622			
Chromocarb	0.128 ±0.006	41100 ±801	1.151			
Chromomycin A3	0.081 ±0.008 **					
Chrysanthemic acid	0.103 ±0.006	27555 ±2248	0.772			
Chrysanthemic acid, ethyl ester	0.113 ±0.009	35023 ±2895	0.981			
Chrysanthemyl alcohol	0.117 ±0.002	34110 ±3633	0.955			
Chrysarobin	0.109 ±0.004	26112 ±5440	0.731			
Chrysene-1,4-quinone	0.110 ±0.004	46470 ±3308	1.301			
Chrysin	0.114 ±0.002	30742 ±711	0.861			
Chrysin dimethyl ether	0.100 ±0.006	12937 ±940	0.373	0.113 ±0.008	39669 ±2590	1.214
Chrysine	0.104 ±0.003	40623 ±950	1.137			
Chrysoeriol	0.107 ±0.004	22822 ±148	0.639			
Chrysophanol	0.130 ±0.004	39781 ±753	1.114			
Chrysophanol 8-O-glucoside	0.119 ±0.002	35723 ±2706	1.000			
Chrysosplenetin	0.118 ±0.004	53989 ±2409	1.509			
Chrysosplenol D	0.105 ±0.009	37568 ±2657	1.052			
Chrysothol	0.118 ±0.005	41581 ±2375	1.164			
Chukrasin methyl ether	0.108 ±0.003	28071 ±4571	0.786			
CI 966 hydrochloride	0.109 ±0.003	31216 ±1331	0.874			
CI-1033	0.125 ±0.006	34184 ±5696	0.957			
CI-1040(PD184352)	0.123 ±0.005	42655 ±1475	1.194			
CI-976	0.108 ±0.006	37195 ±1327	1.041			
CI-994	0.099 ±0.004	38535 ±1622	1.079			
Cianidanol	0.116 ±0.006	34934 ±1149	0.978			
Cibenzoline succinate	0.108 ±0.003	23371 ±4481	0.654			
Ciclesonide	0.102 ±0.006	39184 ±1919	1.097			
Ciclopirox ethanolamine	0.058 ±0.002 **					
Ciclopirox olamine	0.065 ±0.005 **					
Ciglitazone	0.107 ±0.009	27607 ±2807	0.773			
Cilastatin	0.119 ±0.004	35407 ±1284	0.991			
Cilastatin sodium	0.115 ±0.009	39564 ±4231	1.108			

Cilnidipine	0.101 ±0.006	32026 ±1872	0.897			
Cilostamide	0.102 ±0.009	44028 ±3975	1.233			
Cilostazol	0.108 ±0.006	37562 ±3190	1.052			
Cimaterol	0.110 ±0.002	30654 ±4642	0.858			
Cimetidine	0.107 ±0.004	34089 ±3762	0.954			
Cimicifugoside h1	0.112 ±0.005	30741 ±398	0.861			
Cimidahurinine	0.117 ±0.008	40022 ±1827	1.121			
Cimifugin	0.111 ±0.021	48553 ±2814	1.359			
Cimigenoside	0.130 ±0.003	23397 ±2375	0.655			
Cimiracemoside C	0.127 ±0.004	34441 ±2137	0.964			
Cimiside B	0.134 ±0.008	24765 ±404	0.693			
Cinalukast	0.109 ±0.004	31944 ±2827	0.894			
Cinanserin	0.108 ±0.004	28690 ±861	0.803			
Cinanserin hydrochloride	0.105 ±0.005	25348 ±275	0.710			
Cinazepam	0.113 ±0.008	32474 ±1415	0.909			
Cinchonidine	0.107 ±0.001	26582 ±6032	0.744			
Cinchonidine, (-)-	0.105 ±0.001	25693 ±208	0.719			
Cinchonine	0.107 ±0.005	26876 ±4963	0.753			
Cinchophen	0.112 ±0.003	33294 ±4310	0.932			
Cinnamaldehyde	0.115 ±0.001	48150 ±4455	1.348			
Cinnamamide	0.126 ±0.005	38702 ±2411	1.084			
Cinnamic acid	0.128 ±0.005	30018 ±1109	0.840			
Cinnamyl acetate	0.109 ±0.016	38862 ±2510	1.088			
Cinnarazine	0.112 ±0.004	27769 ±244	0.778			
Cinnarizine	0.104 ±0.008	32766 ±1601	0.917			
CinnGEL	0.117 ±0.001	29021 ±943	0.813			
CinnGEL 2 Me	0.120 ±0.007	34931 ±1632	0.978			
Cinnzeylanol	0.122 ±0.002	35351 ±1700	0.990			
Cinobufagin	0.102 ±0.009	43493 ±1936	1.218			
Cinoctramide	0.121 ±0.007	31592 ±3161	0.885			
Cinoxacin	0.106 ±0.002	38841 ±1708	1.088			
Cinromide	0.118 ±0.001	50024 ±3005	1.401			

Cintriamide	0.066 ±0.002 **					
Ciprofibrate	0.098 ±0.003	56810 ±5954	1.591			
Ciprofloxacin	0.099 ±0.004	36578 ±1417	1.024			
Ciprofloxacin hydrochloride hydrate	0.071 ±0.008 **					
Ciproxifan hydrochloride	0.091 ±0.006	39573 ±4703	1.108			
Cirazoline	0.112 ±0.004	32524 ±3414	0.911			
Cirazoline hydrochloride	0.109 ±0.010	46914 ±7801	1.314			
Cirsimaritin	0.127 ±0.006	38563 ±1359	1.080			
cis(+/-)-8-OH-PBZI Hydrobromide	0.102 ±0.008	29439 ±2136	0.824			
cis-(±)-N-Methyl-N-[2-(3,4-dichlorophenyl)-ethyl]-2-(1-pyrrolidinyl)-cyclohexamine dihydrobromide	0.109 ±0.009	46781 ±1817	1.310			
cis-(Z)-Flupenthixol dihydrochloride	0.078 ±0.009 **					
cis-4-Aminocrotonic acid	0.109 ±0.002	35912 ±2960	1.006			
cis-ACPD	0.107 ±0.004	32885 ±1888	0.921			
Cisapride	0.103 ±0.005	42178 ±3396	1.181			
cis-Azetidine-2,4-dicarboxylic acid	0.101 ±0.002	45627 ±1306	1.278			
cis-Methylisoeugenol	0.120 ±0.004	52567 ±6417	1.472			
cis-PDA	0.103 ±0.009	34927 ±3543	0.978			
Cisplatin	0.068 ±0.016 **					
cis-PPDA	0.106 ±0.003	36221 ±1829	1.014			
Citalopram	0.113 ±0.009	29219 ±3477	0.818			
Citalopram hydrobromide	0.104 ±0.005	48753 ±1884	1.348			
CITCO	0.109 ±0.003	31081 ±3104	0.870			
Citicoline	0.109 ±0.003	32262 ±3300	0.903			
Citiolone	0.110 ±0.008	36821 ±969	1.031			
Citreorosein	0.128 ±0.009	27896 ±2663	0.781			
Citrinin	0.111 ±0.008	49069 ±1966	1.457			
Citropten	0.110 ±0.007	32759 ±4036	0.917			
Citrulline	0.117 ±0.005	33386 ±2499	0.935			
CK2 Inhibitor 2	0.098 ±0.013	37869 ±2070	1.060			
CL 218872	0.108 ±0.005	47065 ±3418	1.318			
CL 316,243	0.108 ±0.009	35150 ±1709	0.984			

CL-82198	0.114 ±0.003	26030 ±1200	0.729			
Cladribine	0.072 ±0.001 **					
Clarithromycin	0.103 ±0.011	4360 ±288	0.121	0.110 ±0.000	18163 ±2642	0.556
Clausine Z	0.123 ±0.003	32800 ±1862	0.918			
Clavulanate lithium	0.102 ±0.004	19230 ±2556	0.554			
Clebopride maleate	0.112 ±0.009	41440 ±1971	1.160			
Clemastine fumarate	0.112 ±0.014	43054 ±3157	1.205			
Clemizole hydrochloride	0.078 ±0.012 **					
Clenbuterol	0.111 ±0.003	43916 ±326	1.230			
Clenbuterol hydrochloride	0.118 ±0.010	69389 ±823	1.855			
Cleomiscosin A	0.134 ±0.019	37555 ±4985	1.052			
Cleomiscosin C	0.121 ±0.003	19765 ±1546	0.553			
Clerodenoside A	0.118 ±0.009	42506 ±3159	1.190			
Clerodermic acid methyl ester	0.121 ±0.008	33427 ±2127	0.936			
Cleroindicin B	0.110 ±0.009	38458 ±3690	1.077			
Cleroindicin C	0.108 ±0.008	39016 ±701	1.092			
Cleroindicin D	0.109 ±0.011	30144 ±1759	0.844			
Cleroindicin E	0.116 ±0.009	32072 ±492	0.898			
Cleroindicin F	0.112 ±0.008	44939 ±3485	1.258			
Clerosterol glucoside	0.120 ±0.002	37588 ±862	1.052			
Clidinium bromide	0.106 ±0.001	49672 ±2577	1.391			
Climbazole	0.128 ±0.003 *					
Clinafloxacin	0.056 ±0.003 **					
Clinafoxacin hydrochloride	0.062 ±0.011 **					
Clindamycin hydrochloride	0.110 ±0.003	65885 ±6950	1.874			
Clindamycin palmitate	0.117 ±0.005	36698 ±4145	1.028			
Clindamycin palmitate hydrochloride	0.102 ±0.001	36536 ±1293	1.023			
Clioquinol	0.098 ±0.002	65092 ±4154	1.904			
Clobenpropit	0.130 ±0.003 *					
Clobenpropit dihydrobromide	0.106 ±0.011	10059 ±755	0.269	0.111 ±0.004	26743 ±1516	0.818
Clobetasol propionate	0.112 ±0.008	34313 ±2125	0.961			
Clobutinol hydrochloride	0.112 ±0.006	51684 ±3523	1.447			

Clocortolone pivalate	0.115 ± 0.013	33426 ± 2244	0.936			
Clodronate	0.113 ± 0.006	29726 ± 380	0.832			
Clodronate disodium	0.119 ± 0.003	26217 ± 1338	0.734			
Clodronic acid	0.098 ± 0.005	46424 ± 6846	1.300			
Clofarabine	0.099 ± 0.003	67915 ± 3891	1.931			
Clofazimine	0.123 ± 0.005	18648 ± 2446	0.519			
Clofibrate	0.114 ± 0.004	32933 ± 2806	0.922			
Clofibric acid	0.116 ± 0.009	24291 ± 1813	0.680			
Clofilium tosylate	0.117 ± 0.003	33455 ± 686	0.937			
Clofoctol	0.116 ± 0.004	32226 ± 1265	0.902			
Clomiphene citrate	0.069 ± 0.005 **					
Clomipramine hydrochloride	0.112 ± 0.006	27355 ± 1551	0.766			
Clonidine	0.112 ± 0.005	25056 ± 1454	0.702			
Clonidine hydrochloride	0.102 ± 0.007	29057 ± 4308	0.814			
Clonixin Lysinate	0.104 ± 0.008	23384 ± 1410	0.655			
Clopamide	0.115 ± 0.003	37154 ± 5191	1.040			
Cloperastine Hydrochloride	0.112 ± 0.003	45897 ± 2401	1.285			
Clopidogrel	0.100 ± 0.009	35328 ± 1682	0.989			
Clopidogrel sulfate	0.113 ± 0.002	37243 ± 3075	1.043			
Clopidol	0.101 ± 0.004	39700 ± 4210	1.112			
Cloprostenol Na	0.109 ± 0.005	34550 ± 807	0.967			
Clorgiline hydrochloride	0.105 ± 0.008	19769 ± 876	0.554			
Clorgyline hydrochloride	0.105 ± 0.005	44893 ± 5319	1.257			
Clorsulon	0.111 ± 0.003	54313 ± 3982	1.482			
Closantel	0.107 ± 0.006	19367 ± 4636	0.542			
Clothiapine	0.115 ± 0.008	18803 ± 830	0.503			
Clotrimazole	0.104 ± 0.006	43354 ± 1322	1.214			
Clovanediol	0.125 ± 0.004	40710 ± 3722	1.140			
Clovanediol diacetate	0.122 ± 0.003	32594 ± 1207	0.913			
Cloxacillin sodium	0.108 ± 0.005	24655 ± 1413	0.690			
Cloxyquin	0.107 ± 0.006	39257 ± 2859	1.099			
Clozapine	0.111 ± 0.003	34423 ± 1219	0.964			

CMPD-1	0.110 ± 0.001	39760 ± 5390	1.113			
c-Myc Inhibitor	0.106 ± 0.009	59848 ± 1496	1.751			
Cnidioside B methyl ester	0.124 ± 0.002	33742 ± 2912	0.945			
CNQX	0.103 ± 0.002	40880 ± 4356	1.145			
CNQX disodium	0.098 ± 0.009	40517 ± 2444	1.134			
CNS-1102	0.103 ± 0.006	28094 ± 4751	0.787			
Co 101244 hydrochloride	0.106 ± 0.013	26774 ± 847	0.750			
Co 102862	0.113 ± 0.001	35518 ± 3283	0.994			
Coccinic acid	0.126 ± 0.002	38030 ± 1704	1.065			
Coclauril	0.110 ± 0.006	42443 ± 4026	1.188			
Coclaurine	0.113 ± 0.010	34194 ± 3277	0.957			
Codaphniphylline	0.121 ± 0.005	31713 ± 1936	0.888			
Coenzyme q10	0.107 ± 0.010	42215 ± 1974	1.182			
Coixol	0.118 ± 0.003	47499 ± 4543	1.330			
Colchiceine	0.107 ± 0.008	68840 ± 8286	1.983			
Colchicine, Colchicum autumnale	0.092 ± 0.006 *					
Colecalciferol	0.113 ± 0.004	34199 ± 3255	0.958			
Coleonol B	0.120 ± 0.006	37928 ± 2543	1.062			
Colesevalam hydrochloride	0.103 ± 0.009	28059 ± 3561	0.786			
Colforsin	0.105 ± 0.003	29393 ± 1786	0.823			
Colistimethate sodium	0.111 ± 0.004	20222 ± 177	0.582			
Colistin sulfate	0.066 ± 0.009 **					
Communic acid	0.122 ± 0.006	38343 ± 2044	1.074			
Compound 401	0.121 ± 0.022	31613 ± 3148	0.885			
Compound 52	0.112 ± 0.009	32711 ± 1916	0.916			
Compound 56	0.105 ± 0.005	39722 ± 1996	1.112			
Compound W	0.117 ± 0.002	27899 ± 1519	0.781			
Condelphine	0.101 ± 0.038	18291 ± 1216	0.543			
Conduritol A	0.114 ± 0.002	41264 ± 4869	1.155			
Conessine	0.104 ± 0.003	25319 ± 406	0.709			
Confluentic acid	0.131 ± 0.006	31776 ± 2020	0.890			
Confluentin	0.119 ± 0.002	43067 ± 2807	1.206			

Coniferaldehyde	0.119 ± 0.001	31066 ± 4629	0.870			
Coniferin	0.133 ± 0.013	45176 ± 6436	1.265			
Conophylline	0.118 ± 0.005	22871 ± 1393	0.640			
Convallatoxin	0.114 ± 0.003	32555 ± 469	0.912			
Convolvamine hydrochloride	0.099 ± 0.008	23602 ± 331	0.661			
Coralyne chloride	0.091 ± 0.005 *					
Corchoionol C	0.135 ± 0.010	49215 ± 6381	1.378			
Corchoionoside C	0.124 ± 0.004	35591 ± 1748	0.997			
Cordycepin	0.104 ± 0.007	40743 ± 3235	1.141			
Corianin	0.126 ± 0.004	42159 ± 5616	1.180			
Coriatin	0.110 ± 0.006	43330 ± 4697	1.213			
Coronalolic acid	0.116 ± 0.006	31861 ± 2435	0.892			
Coronalolide	0.122 ± 0.001	37801 ± 1257	1.058			
Coronalolide methyl ester	0.119 ± 0.009	36477 ± 6156	1.021			
Coronarin A	0.120 ± 0.012	47225 ± 4127	1.322			
Coronarin B	0.120 ± 0.011	33406 ± 3520	0.935			
Coronarin D	0.125 ± 0.006	35180 ± 1763	0.985			
Coronarin D ethyl ether	0.120 ± 0.005	40429 ± 4702	1.132			
Coronarin D methyl ether	0.123 ± 0.004	34629 ± 2514	0.970			
Coronarin E	0.117 ± 0.006	26769 ± 1993	0.750			
Coronopilin	0.117 ± 0.009	17037 ± 1954	0.502			
Corosolic acid	0.121 ± 0.002	34610 ± 2587	0.969			
Cortexolone	0.107 ± 0.007	32867 ± 2382	0.920			
Cortexone phenylpropionate	0.110 ± 0.021	45617 ± 4195	1.277			
Corticosterone	0.101 ± 0.003	45963 ± 1718	1.287			
Cortisol acetate	0.116 ± 0.008	57032 ± 9129	1.597			
Cortisone	0.133 ± 0.003	52283 ± 1589	1.387			
Cortisone 21-acetate	0.106 ± 0.005	29414 ± 1536	0.824			
Cortisone acetate	0.118 ± 0.005	21841 ± 1073	0.612			
Corydaline	0.100 ± 0.004	18720 ± 594	0.556			
Corydalmine	0.131 ± 0.003	31444 ± 7010	0.880			
Corylin	0.120 ± 0.004	34654 ± 3478	0.970			

Corynanthine	0.118 ± 0.004	34393 ± 1331	0.963			
Corynanthine hydrochloride	0.118 ± 0.005	50222 ± 3374	1.343			
Corypalmine	0.115 ± 0.007	38049 ± 4672	1.065			
Cosmosiin	0.122 ± 0.001	41933 ± 921	1.174			
Costunolide	0.120 ± 0.003	24906 ± 1350	0.697			
Cotarnine chloride	0.125 ± 0.004	29263 ± 457	0.819			
Cotinine	0.114 ± 0.009	33223 ± 806	0.930			
Cotinine, (-)-	0.107 ± 0.007	18917 ± 104	0.562			
Cotininecarboxylic acid, trans-4-	0.101 ± 0.006	21076 ± 378	0.626			
Cotoin	0.114 ± 0.006	55523 ± 6318	1.555			
Coumarin	0.107 ± 0.008	34473 ± 2709	0.965			
Coumermycin A1	0.073 ± 0.009 **					
Coumestrol	0.099 ± 0.002	19965 ± 3768	0.559			
Coumophos	0.113 ± 0.005	31139 ± 2890	0.872			
Coumurrayin	0.127 ± 0.002	37453 ± 2182	1.049			
COX-1 Inhibitor IV, TFAP	0.099 ± 0.005	35637 ± 5173	0.998			
COX-1 Inhibitor, FR122047	0.082 ± 0.001 **					
COX-2 Inhibitor II	0.094 ± 0.008	32088 ± 578	0.898			
CP 339818 hydrochloride	0.116 ± 0.004	32862 ± 1788	0.920			
CP 93129 dihydrochloride	0.114 ± 0.007	29401 ± 2504	0.823			
CP 94253 hydrochloride	0.119 ± 0.005	36381 ± 1440	1.019			
CP 41475	0.108 ± 0.008	59608 ± 8778	1.669			
CP 55940	0.100 ± 0.003	39887 ± 2003	1.117			
CP 690550	0.128 ± 0.007	30176 ± 2008	0.845			
CP 724714	0.115 ± 0.013	35645 ± 2320	0.998			
C-PAF	0.107 ± 0.003	33845 ± 2684	0.948			
CPCCOEt	0.112 ± 0.004	55782 ± 6671	1.562			
CPNQ	0.106 ± 0.007	39991 ± 2983	1.120			
CPT 11	0.136 ± 0.004 *					
CR 2249	0.105 ± 0.011	41330 ± 3271	1.157			
CR8, (R)-Isomer	0.130 ± 0.005	64390 ± 5808	1.907			
CR8, (S)-Isomer	0.120 ± 0.002	38148 ± 1085	1.068			

CRAC Channel Inhibitor, BTP2	0.096 ±0.001	32166 ±1982	0.901			
Crategolic acid	0.124 ±0.012	37526 ±2877	1.051			
Creatinine	0.103 ±0.006	40704 ±5515	1.140			
Crebanine	0.126 ±0.006	46257 ±2226	1.295			
Crenatine	0.116 ±0.004	24180 ±1252	0.677			
Cresol	0.107 ±0.002	38822 ±1590	1.087			
Cresopirine	0.108 ±0.006	36877 ±1938	1.033			
Creticoside C	0.126 ±0.007	44871 ±3289	1.256			
Cromakalim	0.111 ±0.005	32003 ±1153	0.896			
Cromolyn disodium salt	0.106 ±0.006	55978 ±6895	1.567			
Crotamiton	0.103 ±0.003	24986 ±1781	0.700			
Crustecdysone	0.107 ±0.002	51004 ±7951	1.428			
Cryoflurane	0.106 ±0.004	29327 ±2328	0.821			
Cryptomeridiol	0.117 ±0.007	34771 ±1583	0.974			
Cryptopine	0.108 ±0.007	42062 ±3537	1.178			
Cryptotanshinone	0.124 ±0.003	22707 ±1012	0.636			
CTPB	0.109 ±0.020	41786 ±1415	1.170			
Cucumegastigmane I	0.127 ±0.002	26052 ±1826	0.729			
Cudratricusxanthone A	0.082 ±0.009 **					
Cudraxanthone B	0.111 ±0.004	27634 ±1513	0.774			
Cudraxanthone D	0.124 ±0.003	37627 ±3138	1.054			
Cudraxanthone L	0.118 ±0.003	32439 ±973	0.908			
Cudraxanthone L triacetate	0.115 ±0.002	29800 ±2198	0.834			
Cumene hydroperoxide	0.110 ±0.012	38839 ±2759	1.087			
Cuniloside B	0.117 ±0.012	43288 ±4167	1.212			
Curculigoside	0.118 ±0.004	36842 ±1385	1.032			
Curcumin, Curcuma longa L.	0.103 ±0.005	127138 ±7775	3.720	0.106 ±0.007	60695 ±9925	1.857
CV 1808	0.103 ±0.015	33746 ±603	0.945			
CV 3988	0.109 ±0.009	18680 ±3314	0.516			
C-Veratroylglycol	0.112 ±0.007	43535 ±952	1.219			
CX 546	0.103 ±0.008	39841 ±6798	1.116			
CXCR4 Antagonist I, AMD3100	0.105 ±0.003	43961 ±427	1.231			

CY 208-243	0.102 ± 0.006	31702 ± 888	0.888			
Cyacetacide	0.111 ± 0.001	41417 ± 5246	1.160			
Cyanocobalamin	0.120 ± 0.003	28424 ± 1434	0.796			
Cyanopindolol hemifumarate	0.101 ± 0.005	59089 ± 4269	1.580			
CYC 116	0.120 ± 0.006	31321 ± 8362	0.877			
Cycinchophene	0.127 ± 0.026	26271 ± 834	0.736			
Cyclamic acid	0.102 ± 0.002	39759 ± 3152	1.113			
Cyclandelate	0.103 ± 0.003	35020 ± 3355	0.981			
Cyclizine	0.107 ± 0.005	28959 ± 2576	0.811			
Cyclizine hydrochloride	0.113 ± 0.003	40183 ± 2301	1.125			
Cyclo [Arg-Gly-Asp-D-Phe-Val]	0.117 ± 0.003	26131 ± 1549	0.732			
Cycloart-22-ene-3,25-diol	0.126 ± 0.004	37962 ± 2624	1.063			
Cycloart-25-ene-3,24-diol	0.127 ± 0.005	36921 ± 934	1.034			
Cycloartane-3,24,25-triol	0.121 ± 0.004	41095 ± 4322	1.151			
Cyclobenzaprine hydrochloride	0.110 ± 0.004	34460 ± 2102	0.965			
Cyclocalopin A	0.126 ± 0.004	40087 ± 3062	1.122			
Cyclocerberidol	0.110 ± 0.005	45119 ± 2827	1.263			
Cyclocreatine	0.099 ± 0.002	43563 ± 5651	1.220			
Cyclocytidine hydrochloride	0.109 ± 0.003	37102 ± 1964	1.039			
Cycloeucalenol	0.129 ± 0.004	36077 ± 1410	1.010			
Cycloheterophyllin	0.132 ± 0.013	43256 ± 5496	1.211			
Cycloheximide	0.110 ± 0.004	44491 ± 3548	1.246			
Cycloheximide-N-ethylethanoate	0.104 ± 0.005	28668 ± 1592	0.803			
Cycloleucine	0.108 ± 0.010	39323 ± 6228	1.101			
Cyclomusalenone	0.128 ± 0.002	33373 ± 1910	0.934			
Cyclopamine	0.118 ± 0.009	31272 ± 1528	0.876			
Cyclopenthiazide	0.130 ± 0.009	33965 ± 1828	0.951			
Cyclopentolate hydrochloride	0.111 ± 0.012	28109 ± 462	0.787			
Cyclophosphamide	0.117 ± 0.022	70371 ± 7917	1.921			
Cyclopiazonic acid	0.118 ± 0.009	43409 ± 1653	1.215			
Cyclopropanecarboxylate-octyl	0.113 ± 0.001	27308 ± 3139	0.765			
Cycloserine (d)	0.079 ± 0.004 **					

Cycloserine, L-	0.099 ± 0.011	21577 ± 1608	0.604			
Cyclosporin A, Tolypocladium inflatum	0.077 ± 0.005 **					
Cyclosporine	0.097 ± 0.002	24856 ± 1020	0.696			
Cyclothiazide	0.109 ± 0.003	36689 ± 997	1.027			
Cycloveratrylene	0.107 ± 0.010	34845 ± 1447	0.976			
Cynaroside	0.107 ± 0.015	36158 ± 5098	1.012			
Cypermethrin	0.127 ± 0.009	28548 ± 1287	0.799			
CyPPA	0.106 ± 0.005	42430 ± 3353	1.188			
Cyproheptadine	0.116 ± 0.001	20387 ± 4164	0.571			
Cyproheptadine hydrochloride	0.107 ± 0.012	24891 ± 2372	0.697			
Cyproterone	0.105 ± 0.008	48270 ± 4064	1.352			
Cyproterone acetate	0.100 ± 0.004	35377 ± 3456	0.991			
Cyromazine	0.109 ± 0.004	33289 ± 1678	0.932			
Cystamine dihydrochloride	0.109 ± 0.003	40839 ± 3006	1.143			
CYT11387	0.138 ± 0.003 **					
Cytarabine	0.115 ± 0.004	26439 ± 1662	0.740			
Cytidine	0.088 ± 0.003 **					
Cytidine 5'-diphosphocholine sodium salt hydrate	0.099 ± 0.003	34700 ± 2367	0.972			
Cytisine	0.100 ± 0.006	32371 ± 1623	0.906			
Cytisine, (-)-	0.118 ± 0.005	32889 ± 750	0.921			
Cytochalasin B	0.120 ± 0.011	24478 ± 1682	0.685			
Cytochalasin D	0.110 ± 0.013	42107 ± 1407	1.179			
Cytochalasin E	0.105 ± 0.011	43292 ± 468	1.212			
Cytosine-1-β-D-arabinofuranoside hydrochloride	0.103 ± 0.002	31758 ± 1991	0.889			
D 4476	0.109 ± 0.005	41614 ± 3608	1.165			
D-(-)-2-Amino-4-phosphonobutanoic acid	0.101 ± 0.009	23581 ± 3561	0.660			
D-(-)-2-Amino-5-phosphonovaleric acid	0.104 ± 0.008	41255 ± 4418	1.155			
D,L-Nornicotine	0.106 ± 0.006	26851 ± 4275	0.752			
d,l-Threo-3-hydroxyaspartic acid	0.110 ± 0.010	29625 ± 2789	0.829			
D12-Prostaglandin J2	0.112 ± 0.004	21929 ± 1395	0.614			
d-3-Methoxy-N-methylmorphinan hydrobromide	0.110 ± 0.004	36878 ± 1273	1.033			
D-58	0.099 ± 0.020	59460 ± 5714	1.751			

D-609	0.106 ± 0.007	62285 ± 5830	1.893			
D-609 potassium	0.108 ± 0.009	39082 ± 2766	1.094			
D-64131	0.117 ± 0.004	31001 ± 3582	0.868			
Dabigatran etexilate mesylate	0.094 ± 0.004 *					
Dacarbazine	0.063 ± 0.002 **					
Dactinomycin	0.118 ± 0.003	18441 ± 293	0.513			
Dadahol A	0.126 ± 0.005	41406 ± 708	1.159			
Daidzein	0.118 ± 0.005	42389 ± 2582	1.187			
Daidzin	0.098 ± 0.011	35657 ± 492	0.998			
D-Alanine	0.107 ± 0.014	29277 ± 1488	0.820			
Dalbergione	0.103 ± 0.006	35880 ± 2553	1.005			
Dalbergione, 4-methoxy-4'-hydroxy-	0.107 ± 0.006	39168 ± 2010	1.097			
Dammar-20(21)-en-3,24,25-triol	0.110 ± 0.018	44382 ± 4788	1.243			
Dammaradienyl acetate	0.124 ± 0.003	33386 ± 605	0.935			
Dammarenediol II	0.121 ± 0.002	39063 ± 2513	1.094			
Damnacanthal	0.109 ± 0.009	52146 ± 8018	1.460			
Danazol	0.100 ± 0.005	35436 ± 2208	0.992			
Danofloxacin Mesylate	0.057 ± 0.003 **					
Danthron	0.122 ± 0.007	18601 ± 1057	0.536			
Dantrolene	0.115 ± 0.011	31149 ± 1541	0.872			
Dantrolene sodium	0.097 ± 0.011	33124 ± 2680	0.927			
Dantron	0.125 ± 0.003	46061 ± 1216	1.290			
D-AP5	0.113 ± 0.003	30817 ± 2532	0.863			
DAPH	0.099 ± 0.003	31708 ± 2932	0.888			
Daphmacrine	0.115 ± 0.010	41610 ± 5019	1.165			
Daphmacropodine	0.129 ± 0.005	27787 ± 599	0.778			
Daphnetin	0.076 ± 0.010 *					
Daphnezomine B	0.122 ± 0.007	46651 ± 4085	1.306			
Daphnilongeranin A	0.115 ± 0.011	40525 ± 2126	1.135			
Daphnilongeranin C	0.124 ± 0.004	36425 ± 3285	1.020			
Daphnilongeridine	0.113 ± 0.004	35149 ± 860	0.984			
Daphniyunnine A	0.117 ± 0.003	40919 ± 1950	1.146			

Daphniyunnine B	0.115 ± 0.003	38560 ± 912	1.080			
Daphnoretin	0.117 ± 0.003	41891 ± 2898	1.173			
Daphylloside	0.111 ± 0.002	44130 ± 3017	1.236			
Dapsone	0.109 ± 0.008	28876 ± 1841	0.809			
Dapt	0.111 ± 0.004	30526 ± 2167	0.855			
Daptomycin	0.113 ± 0.002	27474 ± 1019	0.769			
D-arabinitol	0.124 ± 0.002	34779 ± 4537	0.974			
D-Arginine	0.125 ± 0.006	24141 ± 2119	0.676			
Darifenacin hydrobromide	0.124 ± 0.005	26058 ± 2125	0.730			
Darutigenol	0.104 ± 0.014	37507 ± 2581	1.050			
Darutoside	0.111 ± 0.010	37423 ± 1443	1.048			
Dasatinib	0.121 ± 0.003	34684 ± 945	0.971			
D-Asparagine	0.114 ± 0.007	33936 ± 1472	0.950			
D-Aspartic acid	0.108 ± 0.007	33180 ± 5210	0.929			
Datiscetin	0.071 ± 0.012 **					
Daturabietatriene	0.122 ± 0.004	32417 ± 860	0.908			
Daturametelin I	0.116 ± 0.011	67478 ± 11773	1.886			
Daturataturin A	0.118 ± 0.008	39335 ± 3397	1.101			
Daturataturin A aglycone	0.119 ± 0.003	40044 ± 1822	1.121			
DAU 5884 hydrochloride	0.115 ± 0.005	29105 ± 3399	0.815			
Daucosterol	0.119 ± 0.014	34794 ± 2022	0.974			
Daunorubicin	0.109 ± 0.002	5456 ± 173	0.157	0.108 ± 0.005	28860 ± 3208	0.883
Daunorubicin hydrochloride	0.121 ± 0.006	27156 ± 2257	0.760			
Daurichromenic acid	0.131 ± 0.008	33965 ± 2465	0.951			
Dazmegrel	0.118 ± 0.013	39298 ± 4390	1.100			
D-Biotin	0.104 ± 0.007	34857 ± 1632	0.976			
DBO-83	0.098 ± 0.009	34493 ± 538	0.966			
DCA	0.106 ± 0.002	40354 ± 5193	1.130			
DCB	0.114 ± 0.006	40512 ± 1020	1.134			
DCEBIO	0.101 ± 0.012	35321 ± 2578	0.989			
DCHA	0.131 ± 0.006 *					
DCPIB	0.112 ± 0.013	63952 ± 8663	1.770			

D-cycloserine	0.052 ± 0.005 **					
D-Cysteine	0.113 ± 0.002	41433 ± 3597	1.160			
De-4'-O-methylyangambin	0.138 ± 0.009	39703 ± 1387	1.112			
Deacetoxy(7)-7-oxokhivorinic acid	0.109 ± 0.004	32407 ± 3069	0.907			
Deacetoxy-7-oxogedunin	0.106 ± 0.006	23703 ± 2270	0.664			
Deacetylcolchicine, N-formyl-	0.096 ± 0.005 *					
Deacetylgedunin	0.111 ± 0.007	39890 ± 1297	1.117			
Deacetylpseudolaric acid A	0.118 ± 0.002	30430 ± 795	0.852			
Deacetylsalannin	0.125 ± 0.008	30001 ± 2843	0.840			
Deacetylxylopic acid	0.118 ± 0.001	42578 ± 3391	1.192			
Deanol benzilate	0.103 ± 0.007	43532 ± 2766	1.219			
Debrisoquin sulfate	0.107 ± 0.006	33092 ± 2722	0.927			
Decamethonium bromide	0.116 ± 0.005	38887 ± 2757	1.089			
Decamethonium dibromide	0.096 ± 0.006	36914 ± 2134	1.034			
Decanedioic acid	0.127 ± 0.012	27715 ± 1896	0.776			
Decanoic acid	0.110 ± 0.002	34107 ± 2008	0.955			
Decarine	0.126 ± 0.029	27245 ± 1331	0.763			
Decitabine	0.099 ± 0.004	6587 ± 211	0.182	0.105 ± 0.007	19096 ± 2043	0.584
Decoquinate	0.111 ± 0.004	23876 ± 3909	0.669			
Decoyinine	0.120 ± 0.009	34534 ± 3193	0.967			
Dec-RVKR-CMK	0.114 ± 0.005	47636 ± 394	1.471			
Decylubiquinone	0.109 ± 0.002	42922 ± 2809	1.202			
Deferiprone	0.113 ± 0.001	32843 ± 1788	0.920			
Deferoxamine Mesylate	0.098 ± 0.005	31825 ± 1149	0.891			
Deflazacort	0.107 ± 0.002	55257 ± 4068	1.547			
Deguelin	0.119 ± 0.007	24390 ± 2208	0.683			
Deguelin(-)	0.108 ± 0.006	38288 ± 1359	1.072			
Dehydroabietamide	0.101 ± 0.006	40229 ± 4412	1.126			
Dehydroabietic acid	0.119 ± 0.008	44827 ± 6865	1.255			
Dehydroabietinol	0.120 ± 0.005	40620 ± 3843	1.137			
Dehydroacetic acid	0.096 ± 0.004	26547 ± 3526	0.743			
Dehydroadynerigenin glucosyldigitaloside	0.125 ± 0.002	40189 ± 2960	1.125			

Dehydroadynerigenin β-neritrioside	0.122 ± 0.001	35405 ± 1003	0.991			
Dehydrochol acid	0.116 ± 0.006	57941 ± 3904	1.613			
Dehydrocholate sodium	0.108 ± 0.009	34377 ± 359	0.963			
Dehydrocostus lactone	0.106 ± 0.010	32285 ± 3547	0.904			
Dehydrocrebanine	0.124 ± 0.009	39618 ± 4463	1.109			
Dehydrocrenatidine	0.078 ± 0.006 **					
Dehydrocrenatine	0.081 ± 0.005 **					
Dehydrodiconiferyl alcohol	0.129 ± 0.007	22530 ± 527	0.631			
Dehydrodihydrorotenone	0.111 ± 0.004	33101 ± 2837	0.927			
Dehydroepiandrosterone	0.131 ± 0.012	13339 ± 1568	0.371	0.116 ± 0.006	31664 ± 5034	0.969
Dehydroespeletone	0.122 ± 0.004	44166 ± 5503	1.237			
Dehydroheliobuphthalmin	0.122 ± 0.005	32344 ± 1685	0.906			
Dehydroisoandosterone 3-acetate	0.109 ± 0.002	50824 ± 7018	1.423			
Dehydrokawain, 5,6-	0.127 ± 0.001	55829 ± 5866	1.563			
Dehydrorotenone	0.108 ± 0.006	35356 ± 1279	0.990			
Dehydrovariabilin	0.111 ± 0.007	27632 ± 1985	0.774			
Dehydro-α-lapachone	0.114 ± 0.005	26859 ± 3000	0.752			
Delavirdine mesylate	0.115 ± 0.006	31757 ± 2091	0.889			
Delcorine	0.103 ± 0.006	27189 ± 3155	0.761			
Delorazepam	0.109 ± 0.013	39739 ± 3503	1.113			
Deltaline	0.099 ± 0.005	11406 ± 969	0.339	0.102 ± 0.014	33544 ± 1111	1.026
Deltamethrin	0.118 ± 0.002	36101 ± 1513	1.011			
Deltorphin II	0.106 ± 0.007	44171 ± 1716	1.237			
Demecarium bromide	0.133 ± 0.003 *					
Demeclocycline hydrochloride	0.097 ± 0.001	19731 ± 433	0.545			
Demethoxydeacetoxypseudolaric acid B	0.130 ± 0.005	39867 ± 2356	1.116			
Demethoxyencecalin	0.124 ± 0.006	47208 ± 4203	1.322			
Demethylepipodophyllotoxin, 4'-	0.126 ± 0.014	32446 ± 5872	0.908			
Demethylnobiletin	0.119 ± 0.007	26846 ± 1867	0.752			
Demethyloxyphenonium	0.115 ± 0.008	48403 ± 2053	1.355			
Demissidine	0.106 ± 0.003	23412 ± 308	0.656			
Demoxepam	0.109 ± 0.011	39156 ± 5116	1.096			

Denatonium benzoate	0.115 ± 0.008	34592 ± 2457	0.969			
Denbufylline	0.108 ± 0.004	44384 ± 5218	1.243			
Dendocarbin A	0.123 ± 0.004	39427 ± 2685	1.104			
De-O-methylacetovanillochromene	0.132 ± 0.007	32391 ± 1114	0.907			
Deoxyadenosine	0.108 ± 0.006	12161 ± 306	0.350	0.102 ± 0.009	23486 ± 626	0.719
Deoxyartemisinin	0.115 ± 0.005	36648 ± 1974	1.026			
Deoxycalyciphylline B	0.126 ± 0.006	37916 ± 5290	1.062			
Deoxycholic acid	0.115 ± 0.006	44859 ± 2778	1.256			
Deoxycorticosterone	0.112 ± 0.003	40033 ± 5781	1.121			
Deoxyelephantopin	0.125 ± 0.005	34829 ± 1391	0.975			
Deoxygedunin	0.111 ± 0.007	32282 ± 2788	0.904			
Deoxyisocalyciphylline B	0.115 ± 0.006	39672 ± 2820	1.111			
Deoxykhivorin	0.110 ± 0.011	41476 ± 3219	1.161			
Deoxylapachol	0.116 ± 0.002	25936 ± 1039	0.726			
Deoxymannojirimycin(1)	0.107 ± 0.001	28159 ± 3729	0.788			
Deoxynorjrimycin(1)	0.107 ± 0.002	26858 ± 3893	0.752			
Deoxypeganine hydrochloride	0.099 ± 0.004	46853 ± 931	1.312			
Deoxyphorbol 13-acetate, 12-	0.119 ± 0.008	19473 ± 1631	0.578			
Deoxyphorbol 13-phenylacetate 20-acetate, 12-	0.122 ± 0.012	17609 ± 1567	0.523			
Deoxypodophyllotoxin	0.119 ± 0.003	37292 ± 1826	1.044			
Deoxysappanone b 7,3'-dimethyl ether	0.110 ± 0.004	32743 ± 563	0.917			
Deoxysappanone b 7,3'-dimethyl ether acetate	0.106 ± 0.002	38940 ± 628	1.090			
Deoxysappanone b 7,4'-dimethyl ether	0.116 ± 0.007	28922 ± 3328	0.810			
Deoxysappanone b trimethyl ether	0.112 ± 0.006	42790 ± 2267	1.198			
Deoxyshikonin	0.097 ± 0.021	22312 ± 435	0.625			
Deoxyvasicinone	0.121 ± 0.005	44043 ± 1863	1.233			
Dephostatin	0.102 ± 0.006	26825 ± 1291	0.751			
Deprenyl	0.113 ± 0.002	36112 ± 1582	1.011			
Deptropine citrate	0.116 ± 0.004	31283 ± 1345	0.876			
Dequalinium chloride	0.102 ± 0.006	43838 ± 2749	1.227			
Dequalinium chloride hydrate	0.078 ± 0.004 **					
Dequalinium dichloride	0.064 ± 0.018 **					

Deracoxib	0.121 ±0.006	35726 ±7662	1.000			
Dermorphin	0.103 ±0.006	35937 ±2788	1.006			
Derrone	0.125 ±0.003	41646 ±1210	1.166			
Derrusnin	0.093 ±0.002 *					
Derrustone	0.111 ±0.003	28632 ±5235	0.802			
Desacetyl (7)-khivorinic acid, methyl ester	0.104 ±0.006	28514 ±1408	0.798			
Desacetylcolforsin	0.111 ±0.010	44072 ±2510	1.234			
Desipramine hydrochloride	0.100 ±0.017	28324 ±751	0.793			
Desloratadine	0.102 ±0.007	34663 ±3867	0.971			
Desloratidine	0.102 ±0.005	18071 ±1066	0.521			
Desmethoxycentaureidin	0.128 ±0.016	37895 ±2754	1.061			
Desmethyl YM298198 hydrochloride	0.117 ±0.002	34473 ±2727	0.965			
Desonide	0.100 ±0.004	34242 ±5754	0.959			
Desoximetasone	0.113 ±0.003	56250 ±3135	1.566			
Desoxycorticosterone acetate	0.108 ±0.004	27665 ±1373	0.775			
Desoxycortone	0.116 ±0.014	46186 ±5538	1.293			
Desoxymetasone	0.111 ±0.008	26327 ±2595	0.737			
Desoxypeganine hydrochloride	0.102 ±0.003	42316 ±628	1.185			
Desvenlafaxine succinate	0.100 ±0.006	54498 ±689	1.570			
Dexamethasone	0.117 ±0.005	42655 ±1757	1.194			
Dexamethasone acetate	0.111 ±0.003	53601 ±1863	1.501			
Dexamethasone sodium phosphate	0.120 ±0.003	23629 ±3569	0.662			
Dexbrompheniramine maleate	0.123 ±0.004	37998 ±9246	1.064			
Dexchlorpheniramine maleate	0.104 ±0.003	59881 ±2983	1.667			
Dexfenfluramine hydrochloride	0.110 ±0.004	35574 ±2519	0.996			
Dexfosfoserine	0.102 ±0.006	34103 ±3719	0.955			
Dexibuprofen	0.110 ±0.002	40263 ±2870	1.127			
Dexlansoprazole	0.101 ±0.006	32963 ±4357	0.923			
Dexpanthenol	0.108 ±0.004	37689 ±2566	1.055			
Dexpropranolol hydrochloride	0.111 ±0.006	21039 ±3033	0.589			
Dexrazoxane hydrochloride	0.112 ±0.006	44175 ±1116	1.237			
Dextromethorphan hydrobromide	0.117 ±0.004	28923 ±2029	0.810			

DFB	0.107 ± 0.001	46511 ± 2202	1.302			
D-Glutamic acid	0.113 ± 0.005	28768 ± 3579	0.805			
D-Glutamine	0.109 ± 0.006	27404 ± 5978	0.767			
DH 97	0.116 ± 0.007	51478 ± 6385	1.441			
DHBP dibromide	0.101 ± 0.007	5546 ± 1048	0.153	0.105 ± 0.011	12723 ± 695	0.389
D-Histidine	0.113 ± 0.002	32739 ± 3038	0.917			
Diacerein	0.108 ± 0.008	32656 ± 1579	0.914			
Diacerin	0.109 ± 0.003	25011 ± 1005	0.700			
Diacetamate	0.104 ± 0.001	43417 ± 2715	1.216			
Diacetonamine	0.120 ± 0.002	41147 ± 1955	1.152			
Diacetylkorseveriline	0.100 ± 0.003	29816 ± 568	0.835			
Diacylglycerol kinase inhibitor I	0.105 ± 0.002	39796 ± 1174	1.114			
Diacylglycerol Kinase Inhibitor II	0.116 ± 0.010	3142 ± 694	0.087	0.115 ± 0.011	16954 ± 1945	0.510
Diallyl sulfide	0.107 ± 0.013	36684 ± 577	1.027			
Diallyl trisulfide	0.103 ± 0.004	42844 ± 5899	1.200			
Dianilinopyrimi	0.133 ± 0.003	25217 ± 1036	0.706			
Diatrizoic acid	0.103 ± 0.003	25938 ± 4930	0.726			
Diaveridine	0.109 ± 0.004	28631 ± 4925	0.802			
Diazepam	0.139 ± 0.003 **					
Diazoxide	0.096 ± 0.004	36005 ± 1539	1.008			
Dibekacin	0.103 ± 0.008	41962 ± 3828	1.175			
Dibenzepine hydrochloride	0.102 ± 0.008	34846 ± 1543	0.976			
Dibenzothiophene	0.111 ± 0.006	29706 ± 24	0.832			
Dibucaine	0.118 ± 0.006	36966 ± 2375	1.035			
Dibucaine hydrochloride	0.117 ± 0.006	24352 ± 3926	0.682			
Dibutyl phthalate	0.120 ± 0.004	47971 ± 3629	1.343			
Dibutyryl-cAMP, sodium salt	0.119 ± 0.009	24031 ± 1223	0.673			
Dibutyrylcyclic AMP	0.108 ± 0.003	23914 ± 1441	0.670			
Dibutyrylcyclic GMP	0.105 ± 0.007	23921 ± 1248	0.670			
Dichlorisone acetate	0.111 ± 0.003	30499 ± 1662	0.854			
Dichloroacetic acid	0.108 ± 0.003	38460 ± 5806	1.077			
Dichlorobenzamil	0.099 ± 0.026	62378 ± 5954	1.895			

Dichlorophen	0.074 ± 0.008 **					
Dichlorphenamide	0.109 ± 0.005	40646 ± 3407	1.138			
Dichlorvos	0.109 ± 0.004	22518 ± 4352	0.630			
Diclazuril	0.119 ± 0.004	21280 ± 1230	0.581			
Diclofenac sodium	0.092 ± 0.004 *					
Dicloxacillin sodium	0.109 ± 0.008	18996 ± 2351	0.547			
Dicoumarol	0.101 ± 0.009	39043 ± 244	1.093			
Dictamnine	0.069 ± 0.011 **					
Dicumarol	0.110 ± 0.003	42208 ± 2113	1.182			
Dicyclomine hydrochloride	0.101 ± 0.003	30637 ± 926	0.858			
Didanosine	0.117 ± 0.008	27577 ± 3764	0.772			
Didymin	0.063 ± 0.005 **					
Dienestrol	0.111 ± 0.003	42149 ± 1598	1.180			
Diethylcarbamazine citrate	0.118 ± 0.005	58351 ± 3731	1.592			
Diethylenetriaminepentaacetic acid	0.131 ± 0.004 *					
Diethylstilbestrol	0.109 ± 0.017	63146 ± 2896	1.768			
Diethyltoluamide	0.111 ± 0.008	33001 ± 2217	0.924			
Diferidin	0.108 ± 0.024	31519 ± 2275	0.883			
Diffractaic acid	0.101 ± 0.004	26997 ± 1691	0.756			
Diflorasone Diacetate	0.111 ± 0.002	39935 ± 3977	1.118			
Difloxacin hydrochloride	0.053 ± 0.006 **					
Diflunisal	0.125 ± 0.004	39715 ± 1445	1.112			
Difucol hexamethyl ether	0.108 ± 0.006	28991 ± 724	0.812			
Digitonin	0.099 ± 0.006	33377 ± 1729	0.935			
Digitoxigenin	0.104 ± 0.008	37135 ± 1711	1.040			
Digitoxin	0.118 ± 0.005	23546 ± 3743	0.659			
Digoxigenin	0.110 ± 0.002	38628 ± 3376	1.082			
Digoxin	0.137 ± 0.020	49484 ± 4917	1.386			
Dihomo-g-linolenamide	0.133 ± 0.006	35527 ± 2389	0.995			
Dihomo-g-linolenoyl alanine	0.131 ± 0.001	31745 ± 2778	0.889			
Dihomo-g-linolenoyl dopamine	0.132 ± 0.005	28609 ± 3516	0.801			
Dihomo-g-linolenoyl ethanolamide	0.127 ± 0.002	31685 ± 367	0.887			

Dihomo-g-linolenoyl GABA	0.134 ± 0.004	32817 ± 1208	0.919			
Dihomo-g-linolenoyl glycine	0.131 ± 0.005	36193 ± 1031	1.013			
Dihomo-γ-linolenic acid	0.122 ± 0.004	35843 ± 2393	1.004			
Dihydrexidine hydrochloride	0.111 ± 0.003	47961 ± 8068	1.343			
Dihydroajugapitin	0.117 ± 0.001	37777 ± 1580	1.058			
Dihydroalpinumisoflavone	0.121 ± 0.004	34648 ± 1310	0.970			
Dihydrocapsaicin	0.112 ± 0.007	46230 ± 4504	1.294			
Dihydrocarbamazepine	0.104 ± 0.005	51674 ± 4839	1.447			
Dihydrocassiachromone	0.119 ± 0.003	48402 ± 1788	1.355			
Dihydrocelastrol	0.111 ± 0.001	36919 ± 2614	1.034			
Dihydrocelastryl diacetate	0.106 ± 0.007	38673 ± 414	1.083			
Dihydrocinchonamine	0.125 ± 0.005	29860 ± 1588	0.836			
Dihydrodehydrodiconiferyl alcohol	0.126 ± 0.006	47784 ± 4955	1.338			
Dihydroepistephamiersine 6-acetate	0.119 ± 0.005	34345 ± 1796	0.962			
Dihydroergocristine mesylate	0.123 ± 0.008	69735 ± 2885	1.865			
Dihydroergocristine methanesulfonate	0.096 ± 0.003	33643 ± 532	0.942			
Dihydroergotamine mesylate	0.123 ± 0.012	70561 ± 4938	1.887			
Dihydroergotamine methanesulfonate	0.103 ± 0.012	29203 ± 552	0.818			
Dihydroergotamine tartrate	0.118 ± 0.008	48234 ± 4795	1.351			
Dihydrofenazepam	0.114 ± 0.008	39665 ± 3125	1.111			
Dihydrofissinolide	0.115 ± 0.005	29016 ± 1808	0.812			
Dihydrofolic acid	0.101 ± 0.003	40515 ± 3852	1.134			
Dihydrogambogic acid	0.112 ± 0.006	34168 ± 1805	0.957			
Dihydrogedunic acid, methyl ester	0.122 ± 0.006	30189 ± 2399	0.845			
Dihydrogedunin	0.108 ± 0.007	28308 ± 2075	0.793			
Dihydroguaiaretic acid	0.122 ± 0.010	47353 ± 1586	1.326			
Dihydroisotanshinone II	0.121 ± 0.001	33750 ± 2966	0.945			
Dihydrojasmonic acid, methyl ester	0.096 ± 0.011	31449 ± 1230	0.881			
Dihydrokainic acid	0.112 ± 0.004	39327 ± 1669	1.101			
Dihydrolysergol, 9,10-	0.102 ± 0.004	22669 ± 1469	0.635			
Dihydromethysticin	0.102 ± 0.004	26758 ± 457	0.749			
Dihydromicromelin B	0.110 ± 0.003	28074 ± 1358	0.786			

Dihydromorin	0.115 ± 0.005	37966 ± 1705	1.063			
Dihydromunduletone	0.109 ± 0.006	45464 ± 5854	1.273			
Dihydroniloticin	0.126 ± 0.009	36521 ± 284	1.023			
Dihydroouabain	0.103 ± 0.011	38823 ± 1436	1.087			
Dihydrooxoepistephamiersine	0.120 ± 0.003	48445 ± 3254	1.356			
Dihydroperaksine	0.122 ± 0.003	31986 ± 5183	0.896			
Dihydrophaseic acid	0.123 ± 0.006	42000 ± 3808	1.176			
Dihydropinosylvin	0.123 ± 0.003	47045 ± 3177	1.317			
Dihydropinosylvin methyl ether	0.137 ± 0.007	50333 ± 3006	1.409			
Dihydroresveratrol	0.118 ± 0.003	57991 ± 6048	1.624			
Dihydroresveratrol 3-O-glucoside	0.112 ± 0.005	39988 ± 6422	1.120			
Dihydrorobinetin	0.122 ± 0.000	32661 ± 2149	0.914			
Dihydrorotenone	0.102 ± 0.009	32440 ± 690	0.908			
Dihydrosphingosine	0.105 ± 0.003	29029 ± 1493	0.813			
Dihydrosphingosine-1-phosphate *	0.107 ± 0.007	33002 ± 868	0.924			
Dihydrostreptomycin sulfate	0.108 ± 0.005	40766 ± 876	1.141			
Dihydrotamarixetin	0.118 ± 0.004	34304 ± 3183	0.960			
Dihydrotanshinone	0.125 ± 0.003	38975 ± 4673	1.091			
Dihydrotanshinone i	0.100 ± 0.007	37593 ± 775	1.053			
Dihydroxy (3α,12α)-pregnan-20-one	0.105 ± 0.008	33614 ± 1524	0.941			
Dihydroxy-4'-methoxy-chalcone, 2',6'-	0.108 ± 0.005	44345 ± 3788	1.242			
Dihydroxyflavone, 6,7-	0.104 ± 0.008	25213 ± 976	0.706			
Dihydroxypropyltheobromine	0.121 ± 0.012	22741 ± 2378	0.637			
Dihydro-β-erythroidine hydrobromide	0.106 ± 0.006	41276 ± 1555	1.156			
Diindolylmethane	0.092 ± 0.002 **					
Dilazep	0.115 ± 0.002	48461 ± 2241	1.357			
Dilazep dihydrochloride	0.118 ± 0.010	40979 ± 4787	1.147			
Diloxanide furoate	0.114 ± 0.005	55775 ± 9515	1.562			
Diltiazem	0.114 ± 0.012	49913 ± 3880	1.398			
Diltiazem hydrochloride	0.101 ± 0.010	41188 ± 1346	1.153			
Diludin	0.131 ± 0.011	34988 ± 3326	0.980			
Dimaprit dihydrochloride	0.114 ± 0.002	43534 ± 1845	1.219			

Dimenhydrinate	0.107 ± 0.005	35148 ± 1110	0.984			
Dimercaprol	0.119 ± 0.028	37791 ± 6920	1.058			
Dimeric coniferyl acetate	0.114 ± 0.007	42468 ± 4785	1.189			
Dimethadione	0.118 ± 0.003	70214 ± 3847	1.916			
Dimethisoquin hydrochloride	0.127 ± 0.007	29694 ± 927	0.831			
Dimethyl 4,4-o-phenylene-bis (3-thiophanate)	0.107 ± 0.010	29232 ± 1771	0.818			
Dimethyl fumarate	0.103 ± 0.010	26298 ± 5614	0.736			
Dimethyl phthalate	0.124 ± 0.010	38941 ± 3913	1.090			
Dimethylcaffeic acid	0.107 ± 0.004	17898 ± 942	0.516			
Dimethyloxaloylglycine	0.104 ± 0.008	37517 ± 842	1.050			
Dimetridazole	0.107 ± 0.007	29357 ± 2454	0.822			
Diminazene aceturate	0.111 ± 0.004	19140 ± 1993	0.536			
Diminutol	0.096 ± 0.004	41519 ± 4150	1.163			
Dimpylate	0.108 ± 0.001	39235 ± 3248	1.099			
Dinitolmide	0.113 ± 0.004	26842 ± 2993	0.752			
Dinoprost	0.119 ± 0.005	50787 ± 2338	1.422			
Dinoprost trometamol	0.108 ± 0.006	37651 ± 3139	1.054			
Dinoprostone	0.107 ± 0.001	43464 ± 6879	1.217			
Di-O-methylbergenin	0.119 ± 0.004	46910 ± 5209	1.313			
Diosbulbin B	0.117 ± 0.008	35071 ± 1016	0.982			
Diosbulbin C	0.124 ± 0.006	29503 ± 892	0.826			
Diosbulbin C ethyl ester	0.137 ± 0.014	33785 ± 2295	0.946			
Diosbulbin D	0.125 ± 0.006	40995 ± 4142	1.148			
Diosbulbin G	0.129 ± 0.006	42986 ± 5346	1.204			
Diosbulbin I	0.121 ± 0.002	36222 ± 3871	1.014			
Diosbulbin J	0.125 ± 0.003	31726 ± 2390	0.888			
Diosgenin	0.105 ± 0.006	29632 ± 1586	0.830			
Diosmetine	0.124 ± 0.005	42750 ± 3009	1.197			
Diosmin	0.107 ± 0.003	46833 ± 3051	1.311			
Dioxidine	0.110 ± 0.009	40772 ± 4143	1.142			
Dioxophenanthrene	0.125 ± 0.002	39160 ± 1531	1.096			
Dioxybenzone	0.104 ± 0.003	35822 ± 1933	1.003			

Diperodon hydrochloride	0.118 ± 0.001	42990 ± 4114	1.204			
Diphemanil methylsulfate	0.115 ± 0.005	37351 ± 836	1.046			
Diphenhydramine hydrochloride	0.100 ± 0.011	29854 ± 1445	0.836			
Diphenidol hydrochloride	0.121 ± 0.007	31537 ± 252	0.883			
Diphenoxylate	0.125 ± 0.013	23294 ± 1403	0.652			
Diphenylcyclopropenone	0.111 ± 0.005	23633 ± 1859	0.662			
Diphenyleneiodonium chloride	0.069 ± 0.007 **					
Diphenylpyraline hydrochloride	0.115 ± 0.004	44134 ± 3252	1.236			
Diphenylurea	0.107 ± 0.021	32757 ± 3648	0.917			
Diphyllin	0.107 ± 0.010	49453 ± 1074	1.385			
Dipicolinic acid	0.114 ± 0.007	42466 ± 1877	1.189			
Dipin	0.111 ± 0.007	35837 ± 2385	1.003			
Dipivefrin hydrochloride	0.121 ± 0.002	38858 ± 925	1.088			
Diplosalsalate	0.099 ± 0.009	27440 ± 3020	0.768			
Diprophylline	0.113 ± 0.007	59275 ± 5118	1.618			
Dipropyl-5CT	0.111 ± 0.012	37567 ± 4240	1.052			
Dipropyldopamine hydrobromide	0.097 ± 0.003	36971 ± 3179	1.035			
Dipterocarpol	0.104 ± 0.004	24265 ± 506	0.679			
Dipteryxin	0.112 ± 0.011	18408 ± 1225	0.530			
Dipyridamole	0.097 ± 0.006	29356 ± 1117	0.822			
Dipyrocetyl	0.099 ± 0.007	35610 ± 942	0.997			
Dipyrone	0.107 ± 0.002	22848 ± 571	0.624			
Dirithromycin	0.125 ± 0.004	2396 ± 313	0.065	0.108 ± 0.005	16904 ± 918	0.517
Disodium cromoglycate	0.111 ± 0.004	37630 ± 2826	1.054			
D-Isoleucine	0.115 ± 0.009	25489 ± 1249	0.714			
Disopyramide	0.108 ± 0.002	33279 ± 552	0.932			
Disopyramide phosphate	0.102 ± 0.002	29677 ± 1368	0.831			
Distamycin A	0.104 ± 0.006	22828 ± 621	0.639			
Disulfiram	0.096 ± 0.005 *					
Dixanthogen	0.110 ± 0.007	29342 ± 2787	0.822			
Dizocilpine maleate	0.119 ± 0.004	39565 ± 142	1.108			
Djenkolic acid	0.109 ± 0.008	41192 ± 1853	1.153			

D-Kynurenine	0.109 ±0.003	37501 ±4607	1.050			
DL-2-Amino-3-phosphonopropionic acid	0.100 ±0.006	36860 ±2570	1.032			
DL-2-Amino-5-phosphonovaleric acid	0.103 ±0.008	36858 ±2742	1.032			
DL-4-Hydroxy-3-methoxy-mandelic acid	0.110 ±0.001	41095 ±4911	1.151			
D-lactitol monohydrate	0.112 ±0.012	44440 ±5770	1.244			
Dl-aminoglutethimide	0.116 ±0.001	40064 ±3908	1.122			
DL-AP4	0.109 ±0.002	33481 ±1266	0.937			
DL-AP5	0.111 ±0.002	33369 ±1700	0.934			
DL-Buthionine-[S,R]-sulfoximine	0.101 ±0.003	33742 ±3307	0.945			
DL-Cycloserine	0.086 ±0.001 **					
DL-Dihydrosphingosine	0.117 ±0.005	33890 ±1235	0.949			
DL-erythro-Dihydrosphingosine	0.103 ±0.008	27064 ±1344	0.758			
D-Leucine	0.125 ±0.011	23530 ±2242	0.659			
DL-Homatropine hydrobromide	0.101 ±0.003	47836 ±1216	1.339			
D-limonene	0.117 ±0.007	29110 ±2789	0.815			
DL-Octopamine hydrochloride	0.108 ±0.009	33729 ±4687	0.944			
DL-p-Chlorophenylalanine methyl ester hydrochloride	0.103 ±0.006	36816 ±2797	1.031			
DL-PDMP	0.103 ±0.006	30948 ±1640	0.867			
DL-PPMP	0.103 ±0.004	36026 ±791	1.009			
DL-Stearoylcarnitine chloride	0.097 ±0.008	19930 ±3471	0.551			
DL-Syringaresinol	0.127 ±0.010	39964 ±4584	1.119			
DL-TBOA	0.121 ±0.004	22592 ±1212	0.633			
DL-Thiorphan	0.104 ±0.005	31293 ±1606	0.876			
DL-threo-β-hydroxyaspartic acid	0.103 ±0.005	40822 ±1511	1.143			
D-Lysine	0.107 ±0.009	26557 ±1592	0.744			
DL-α-Difluoromethylornithine hydrochloride	0.095 ±0.005	34354 ±684	0.962			
DL-α-Methyl-p-tyrosine	0.101 ±0.002	27167 ±4321	0.761			
DM 235	0.102 ±0.008	51864 ±5291	1.452			
DMAB-anabaseine dihydrochloride	0.111 ±0.006	12907 ±1436	0.357	0.121 ±0.015	39194 ±1905	1.199
DMBI	0.113 ±0.026	31811 ±882	0.891			
DMeOB	0.110 ±0.002	37300 ±1726	1.044			
D-Methionine	0.120 ±0.014	26515 ±1497	0.742			

DMNB	0.108 ± 0.004	37988 ± 8562	1.064			
DMP 543	0.114 ± 0.011	41840 ± 2921	1.171			
DNA Base Excision Repair Pathway Inhibitor	0.108 ± 0.007	37693 ± 5297	1.055			
DNA Methyltransferase Inhibitor	0.098 ± 0.005	27210 ± 922	0.762			
DNA-PK Inhibitor II	0.121 ± 0.008	28209 ± 1292	0.790			
DNA-PK Inhibitor III	0.114 ± 0.013	36320 ± 2470	1.017			
DNA-PK Inhibitor V	0.117 ± 0.013	26579 ± 964	0.744			
DNQX	0.102 ± 0.003	44930 ± 2787	1.258			
DNQX Disodium Salt	0.109 ± 0.002	33019 ± 1902	0.925			
DO 897/99	0.117 ± 0.008	41201 ± 3390	1.154			
Dobutamine hydrochloride	0.124 ± 0.007	46667 ± 5411	1.307			
Docebenone	0.109 ± 0.002	46187 ± 3373	1.293			
Docetaxel	0.117 ± 0.011	59786 ± 4756	1.665			
Docetaxil	0.138 ± 0.009 *					
Doconexent	0.109 ± 0.006	23468 ± 1264	0.657			
Docosa-4Z,7Z,10Z,13Z,16Z,19Z-hexaenoic acid	0.109 ± 0.003	19155 ± 1096	0.508			
Docosahexaenamide	0.137 ± 0.008	34816 ± 4389	0.975			
Docosahexaenoic acid (22:6 n-3)	0.106 ± 0.003	49906 ± 1443	1.540			
Docosahexaenoyl alanine	0.125 ± 0.013	34314 ± 1237	0.961			
Docosahexaenoyl dopamine	0.124 ± 0.008	36810 ± 1644	1.031			
Docosahexaenoyl ethanolamide	0.123 ± 0.014	27274 ± 3509	0.764			
Docosahexaenoyl GABA	0.130 ± 0.001	31349 ± 1024	0.878			
Docosahexaenoyl glycine	0.127 ± 0.004	32569 ± 3229	0.912			
Docosanoic acid	0.120 ± 0.016	28568 ± 1860	0.800			
Docosanol	0.102 ± 0.015	29277 ± 2537	0.820			
Docosapentaenoic acid	0.105 ± 0.011	28695 ± 4003	0.803			
Docosatetra-7Z,10Z,13Z,16Z-enamide	0.127 ± 0.009	31121 ± 1360	0.871			
Docosatetra-7Z,10Z,13Z,16Z-enoyl alanine	0.136 ± 0.005	38384 ± 1468	1.075			
Docosatetra-7Z,10Z,13Z,16Z-enoyl dopamine	0.125 ± 0.008	30751 ± 2999	0.861			
Docosatetra-7Z,10Z,13Z,16Z-enoyl ethanolamide	0.130 ± 0.006	37941 ± 4576	1.062			
Docosatetra-7Z,10Z,13Z,16Z-enoyl GABA	0.132 ± 0.004	28447 ± 998	0.797			
Docosatetra-7Z,10Z,13Z,16Z-enoyl glycine	0.133 ± 0.004	32619 ± 3098	0.913			

Docosatrienoic acid (22:3 n-3)	0.105 ± 0.001	63012 ± 2362	1.944			
Docosyl caffeate	0.138 ± 0.006	39871 ± 6624	1.116			
Docusate sodium	0.107 ± 0.005	38092 ± 2340	1.067			
Dodecanoic acid	0.116 ± 0.003	36571 ± 3792	1.024			
Dofetilide	0.115 ± 0.001	37531 ± 4611	1.051			
Dolasetron	0.120 ± 0.006	53388 ± 4180	1.495			
Dolasetron mesilate	0.108 ± 0.008	41059 ± 7238	1.150			
Dolasetron mesylate	0.113 ± 0.004	87145 ± 3242	2.426	0.115 ± 0.013	39157 ± 5470	1.198
Domoic acid	0.119 ± 0.012	18626 ± 1301	0.553			
Domperidone	0.099 ± 0.006	29461 ± 1360	0.825			
Donepezil	0.104 ± 0.003	59655 ± 636	1.661			
Donepezil hydrochloride	0.108 ± 0.004	62276 ± 4946	1.771			
Donitriptan hydrochloride	0.111 ± 0.001	24083 ± 1715	0.674			
Dopamine hydrochloride	0.116 ± 0.006	23691 ± 158	0.663			
Doramectin	0.099 ± 0.009	27934 ± 1198	0.782			
Doripenem	0.106 ± 0.005	37022 ± 2695	1.037			
D-Ornithine	0.113 ± 0.009	33899 ± 3059	0.949			
Dorzolamide	0.125 ± 0.010	49526 ± 4190	1.387			
Dorzolamide Hydrochloride	0.113 ± 0.011	34612 ± 1387	0.969			
Dosulepin hydrochloride	0.108 ± 0.004	36629 ± 2221	1.026			
Doxapran hydrochloride	0.132 ± 0.004 *					
Doxazosin mesylate	0.106 ± 0.007	27989 ± 2145	0.784			
Doxepin	0.112 ± 0.004	35832 ± 2751	1.003			
Doxepin hydrochloride	0.104 ± 0.011	31537 ± 3166	0.883			
Doxifluridine	0.111 ± 0.009	31967 ± 1445	0.895			
Doxifluridine Hydrochloride	0.105 ± 0.009	41244 ± 1928	1.155			
Doxofylline	0.108 ± 0.008	33254 ± 360	0.931			
Doxorubicin	0.110 ± 0.002	29674 ± 3022	0.831			
Doxorubicin hydrochloride	0.121 ± 0.010	19378 ± 1250	0.576			
Doxycycline hydrochloride	0.064 ± 0.001 **					
Doxylamine succinate	0.105 ± 0.012	32869 ± 2312	0.920			
DPCPX	0.109 ± 0.002	34208 ± 1477	0.958			

D-Penicillamine	0.113 ±0.004	28955 ±995	0.811			
D-Phenylalanine	0.116 ±0.005	28006 ±1376	0.784			
D-Pinitol	0.124 ±0.002	45098 ±1124	1.263			
DPN	0.113 ±0.002	70581 ±4652	1.976			
DPO-1	0.102 ±0.004	35602 ±1712	0.997			
DPPE fumarate	0.111 ±0.004	26592 ±4354	0.745			
D-Proline	0.116 ±0.010	33528 ±1439	0.939			
D-Pyroglutamic acid	0.109 ±0.011	36507 ±3515	1.022			
DR 2313	0.106 ±0.007	45275 ±3483	1.268			
DRB	0.120 ±0.002	107156 ±9381	3.256	0.116 ±0.010	60191 ±3381	1.842
D-ribofuranosylbenzimidazole	0.090 ±0.008	54919 ±747	1.518			
Drim-7-ene-11,12-diol acetonide	0.118 ±0.002	38526 ±5595	1.079			
Drimiopsin C	0.124 ±0.004	36732 ±2314	1.028			
Drimiopsin D	0.121 ±0.005	41087 ±2377	1.150			
Drofenine hydrochloride	0.116 ±0.001	40522 ±2489	1.135			
Droperidol	0.099 ±0.010	28641 ±795	0.802			
Dropropizine	0.116 ±0.013	34872 ±4097	0.976			
Dropropizine (R,S)	0.112 ±0.007	40044 ±2777	1.121			
Drospirenone	0.106 ±0.004	54466 ±2024	1.569			
DS2	0.109 ±0.005	28467 ±1107	0.797			
D-Serine	0.105 ±0.005	58247 ±4635	1.631			
DSP-4 hydrochloride	0.097 ±0.016	37653 ±3459	1.054			
DTG	0.106 ±0.003	25294 ±3091	0.708			
D-Threonine	0.125 ±0.011	34504 ±2438	0.966			
D-Tryptophan	0.114 ±0.009	31811 ±3501	0.891			
DTT	0.126 ±0.008	50746 ±9128	1.421			
D-Tubocurarine-chloride	0.117 ±0.004	28207 ±1254	0.790			
D-Tyrosine	0.116 ±0.013	24734 ±1299	0.693			
Duartin (-)	0.103 ±0.002	45649 ±1874	1.278			
Duartin, dimethyl ether	0.109 ±0.004	46528 ±2698	1.303			
Dubinidine	0.102 ±0.009	29595 ±853	0.829			
Ducheside A	0.128 ±0.006	30037 ±1252	0.841			

Ducheside A pentaacetate	0.124 ± 0.001	30638 ± 1672	0.858			
Duloxetine	0.117 ± 0.011	37416 ± 3928	1.048			
Duloxetine hydrochloride	0.117 ± 0.005	18783 ± 846	0.541			
Dunnianol	0.117 ± 0.007	32346 ± 1880	0.906			
DuP 697	0.114 ± 0.010	39875 ± 6011	1.116			
Durantoside I	0.130 ± 0.001	28740 ± 2487	0.805			
Durantoside II	0.132 ± 0.004	28947 ± 2439	0.810			
Dutasteride	0.109 ± 0.004	41700 ± 1857	1.168			
D-Valine	0.112 ± 0.007	32055 ± 5543	0.898			
DY131	0.121 ± 0.015	30738 ± 2212	0.861			
Dyclonine hydrochloride	0.114 ± 0.003	43859 ± 3156	1.228			
Dydrogesterone	0.115 ± 0.004	39676 ± 1264	1.111			
Dynamin Inhibitor I, Dynasore	0.091 ± 0.004	31826 ± 801	0.891			
Dynasore	0.107 ± 0.002	45704 ± 3294	1.280			
Dynorphin A (1-13)	0.104 ± 0.006	42662 ± 6287	1.195			
Dynorphin A (1-8)	0.105 ± 0.016	48275 ± 2968	1.352			
Dynorphin A (2-13)	0.102 ± 0.007	38745 ± 4830	1.085			
Dynorphin B	0.104 ± 0.008	37418 ± 4546	1.048			
Dyphylline	0.115 ± 0.010	30304 ± 1947	0.848			
E2F Inhibitor, HLM006474	0.098 ± 0.004	38122 ± 2019	1.067			
E-4031	0.113 ± 0.014	34468 ± 1656	0.965			
E-4031 dihydrochloride	0.112 ± 0.004	25958 ± 457	0.727			
E6 Berbamine	0.115 ± 0.009	20100 ± 1566	0.597			
E-64	0.105 ± 0.006	38511 ± 3547	1.078			
E-64-c	0.115 ± 0.003	35958 ± 504	1.007			
E-64-d	0.116 ± 0.007	33859 ± 227	0.948			
E7080	0.133 ± 0.002	29206 ± 1363	0.818			
Ebastine	0.102 ± 0.007	44084 ± 2256	1.234			
Ebelactone B	0.121 ± 0.002	22922 ± 1335	0.708			
EBPC	0.104 ± 0.007	29571 ± 1716	0.828			
Ebselen	0.103 ± 0.012	31190 ± 1081	0.873			
Eburicol	0.123 ± 0.001	39192 ± 4373	1.097			

Eburnamonine (-)	0.110 ± 0.006	39500 ± 4313	1.106			
Eburnamonine, (-)-	0.109 ± 0.002	29879 ± 448	0.837			
Ecamsule triethanolamine	0.103 ± 0.002	32729 ± 3991	0.916			
Ecdysone	0.104 ± 0.003	44439 ± 1470	1.244			
Ecdysone, b-	0.122 ± 0.006	35832 ± 2603	1.003			
Ecdysterone 2,3:20,22-diacetonide	0.124 ± 0.007	29823 ± 2695	0.835			
Ecdysterone 20,22-monoacetonide	0.110 ± 0.004	26543 ± 1601	0.743			
Echinocystic acid	0.122 ± 0.003	39938 ± 4167	1.118			
Echinomycin	0.072 ± 0.002 **					
Echinophyllin C	0.130 ± 0.006	35164 ± 2005	0.985			
Echinoynethiophene A	0.122 ± 0.002	54911 ± 2758	1.535			
Echinulin	0.115 ± 0.012	32842 ± 3213	0.920			
Echitamine	0.115 ± 0.004	32514 ± 2437	0.910			
Econazole nitrate	0.060 ± 0.001 **					
Edaravone	0.116 ± 0.002	26387 ± 643	0.739			
Edetate disodium	0.115 ± 0.006	33890 ± 3901	0.949			
Edoxudine	0.108 ± 0.008	21733 ± 1912	0.609			
Edrophonium chloride	0.101 ± 0.003	37570 ± 1150	1.052			
eEF-2 Kinase Inhibitor, NH125	0.061 ± 0.008 **					
Efaroxan	0.104 ± 0.006	11398 ± 2372	0.305	0.109 ± 0.011	35728 ± 4821	1.093
Efaroxan hydrochloride	0.102 ± 0.002	41292 ± 1774	1.156			
Efavirenz	0.121 ± 0.002	44531 ± 3204	1.247			
Effusanin A	0.088 ± 0.005 **					
Efloxate	0.110 ± 0.024	35074 ± 1773	0.982			
Efonidipine hydrochloride monoethanolate	0.114 ± 0.006	34007 ± 1007	0.952			
EGFR Inhibitor	0.108 ± 0.002	37405 ± 1121	1.047			
EGFR/ErbB-2 Inhibitor	0.116 ± 0.013	36378 ± 1609	1.019			
EGFR/ErbB-2/ErbB-4 Inhibitor	0.114 ± 0.015	25365 ± 2175	0.710			
EGTA	0.111 ± 0.009	45659 ± 1052	1.278			
EHNA hydrochloride	0.109 ± 0.003	48271 ± 8711	1.352			
Ehoxyquin	0.121 ± 0.007	31580 ± 952	0.884			
Ehretioside B	0.126 ± 0.002	34079 ± 2208	0.954			

Eichlerialactone	0.123 ±0.001	33733 ±3563	0.945			
Eichlerianic acid	0.107 ±0.006	32753 ±1734	0.917			
Eicosa-11Z,14Z-dienamide	0.126 ±0.008	34139 ±1212	0.956			
Eicosa-11Z,14Z-dienoyl alanine	0.129 ±0.008	38389 ±1487	1.075			
Eicosa-11Z,14Z-dienoyl dopamine	0.132 ±0.001	21725 ±3656	0.608			
Eicosa-11Z,14Z-dienoyl ethanolamide	0.124 ±0.002	39005 ±1469	1.092			
Eicosa-11Z,14Z-dienoyl GABA	0.126 ±0.003	35127 ±1749	0.984			
Eicosa-11Z,14Z-dienoyl glycine	0.132 ±0.015	34402 ±3576	0.963			
Eicosa-5,8-dienoic acid (20:2 n-12)	0.108 ±0.006	31172 ±1429	0.873			
Eicosadienoic acid (20:2 n-6)	0.111 ±0.007	36718 ±349	1.028			
Eicosanoic acid	0.125 ±0.005	46073 ±3629	1.290			
Eicosapentaenamide	0.132 ±0.008	40400 ±3236	1.131			
Eicosapentaenoic acid (20:5 n-3)	0.102 ±0.007	31013 ±220	0.868			
Eicosapentaenoyl alanine	0.129 ±0.001	35260 ±1763	0.987			
Eicosapentaenoyl dopamine	0.127 ±0.006	34887 ±1248	0.977			
Eicosapentaenoyl ethanolamide	0.133 ±0.008	28482 ±4702	0.797			
Eicosapentaenoyl GABA	0.136 ±0.013	32958 ±972	0.923			
Eicosapentaenoyl glycine	0.129 ±0.001	29413 ±2707	0.824			
Eicosatrienoic acid (20:3 n-3)	0.106 ±0.004	36182 ±711	1.013			
eIF-2α Inhibitor II, Sal003	0.100 ±0.003	36858 ±1937	1.032			
eIF-2α Inhibitor, Salubrinal	0.106 ±0.010	40956 ±2195	1.147			
elaidylphosphocholine	0.104 ±0.008	34458 ±395	0.965			
Elastatinal	0.124 ±0.001	25627 ±615	0.718			
Eletriptan hydrobromide	0.109 ±0.003	34696 ±4694	0.971			
Eleutheroside C	0.116 ±0.007	34795 ±2417	0.974			
Eliprodil	0.103 ±0.002	30308 ±2515	0.849			
Ellagic acid	0.120 ±0.007	28629 ±699	0.802			
Ellagic acid 7-O-β-D-xylopyranoside-2,3,8-trimethyl ether	0.122 ±0.001	24053 ±446	0.673			
Ellagic Acid, Dihydrate	0.101 ±0.005	55900 ±1369	1.635			
Elliotinol	0.127 ±0.007	46984 ±5813	1.316			
Ellipticine	0.099 ±0.009	35177 ±2004	0.985			
Eltanolone	0.109 ±0.005	36347 ±709	1.018			

Embelin	0.120 ± 0.009	28462 ± 1738	0.797			
EMD 386088 hydrochloride	0.113 ± 0.002	32609 ± 2585	0.913			
EMD 66684	0.107 ± 0.007	48014 ± 2506	1.344			
EMD-20262	0.122 ± 0.002	50181 ± 3102	1.405			
Emedastine	0.117 ± 0.007	39518 ± 1298	1.106			
Emetine dihydrochloride	0.103 ± 0.005	28091 ± 1024	0.787			
Emodin	0.092 ± 0.006 *					
Emtricitabine	0.110 ± 0.006	43681 ± 884	1.223			
Enalapril	0.123 ± 0.007	30359 ± 358	0.850			
Enalapril maleate	0.129 ± 0.011	37149 ± 3537	1.040			
Enalaprilat	0.123 ± 0.009	40935 ± 10593	1.146			
Enantio-7(11)-eudesmen-4-ol	0.127 ± 0.002	35524 ± 3457	0.995			
Enantio-PAF C16	0.107 ± 0.005	36163 ± 2033	1.013			
Encecalin	0.121 ± 0.006	38251 ± 869	1.071			
Enclomifene	0.111 ± 0.008	49121 ± 3733	1.375			
Endo-IWR 1	0.116 ± 0.010	35560 ± 2575	0.996			
Endomorphin-1	0.105 ± 0.004	24732 ± 1174	0.692			
Endomorphin-2	0.102 ± 0.006	40560 ± 3398	1.136			
Endothall	0.121 ± 0.003	33795 ± 1755	0.946			
Engeletin	0.122 ± 0.003	37490 ± 1036	1.050			
Enilconazole	0.123 ± 0.009	38190 ± 2715	1.069			
Enoxacin	0.108 ± 0.004	46391 ± 6247	1.299			
Enoximone	0.100 ± 0.002	37184 ± 3469	1.041			
Enoxolone	0.118 ± 0.005	48193 ± 3047	1.349			
Enrofloxacin	0.056 ± 0.002 **					
ent-11,16-Epoxy-15-hydroxykauran-19-oic acid	0.122 ± 0.002	35828 ± 2855	1.003			
ent-14,15-Dinor-13-oxolabda-8(17),11-dien-18-oic acid	0.118 ± 0.003	33567 ± 3972	0.940			
ent-14,16-Epoxy-8-pimarene-3,15-diol	0.111 ± 0.009	35439 ± 3990	0.992			
ent-16-β,17-Dihydroxy-19-kauranoic acid	0.125 ± 0.004	48007 ± 6474	1.344			
ent-16-β,17-Isopropylidenedioxykaurane	0.125 ± 0.002	31420 ± 1512	0.880			
ent-17-Hydroxykauran-3-one	0.131 ± 0.011	32761 ± 2381	0.917			

ent-3-Oxokauran-17-oic acid	0.118 ± 0.011	33693 ± 2471	0.943			
ent-3-Oxokaurane-16,17-diol	0.124 ± 0.009	51563 ± 7197	1.444			
ent-6,11-Dihydroxy-15-oxo-16-kauren -19-oic acid β-D-glucopyranosyl ester	0.122 ± 0.005	18927 ± 511	0.529			
ent-6,9-Dihydroxy-15-oxo-16-kauren -19-oic acid β-D-glucopyranosyl ester	0.134 ± 0.008	25856 ± 800	0.724			
ent-6,9-Dihydroxy-15-oxo-16-kauren-19-oic acid	0.069 ± 0.007 **					
ent-9-Hydroxy-15-oxo-16-kauren-19-oic acid	0.100 ± 0.007	1824 ± 286	0.051	0.118 ± 0.009	29246 ± 1086	0.895
ent-9-Hydroxy-15-oxo-16-kauren-19-oic acid β-D-glucopyranosyl ester	0.129 ± 0.009	35514 ± 1453	0.994			
ent-9-Hydroxy-15-oxo-19-kauranoic acid	0.123 ± 0.003	33481 ± 2899	0.937			
Entacapone	0.119 ± 0.002	17820 ± 3482	0.522			
Entandrophragmin	0.124 ± 0.003	29012 ± 1692	0.812			
Ent-kauran-17,19-dioic acid	0.126 ± 0.013	29828 ± 3473	0.835			
Ent-kaurane-3,16,17-triol	0.116 ± 0.004	36715 ± 3212	1.028			
EO 1428	0.115 ± 0.006	35574 ± 3293	0.996			
EPA	0.109 ± 0.013	40558 ± 1235	1.136			
Epalrestat	0.105 ± 0.002	52485 ± 2957	1.470			
Ephedrine (1r,2s)-hydrochloride	0.109 ± 0.019	28941 ± 3262	0.810			
Epiafzelechin (2r,3r)(-)	0.105 ± 0.004	36743 ± 2152	1.029			
Epiafzelechin trimethyl ether	0.115 ± 0.008	33040 ± 4062	0.925			
Epiandrosterone	0.111 ± 0.007	47523 ± 3009	1.331			
Epibatidine (±)	0.106 ± 0.003	25985 ± 1810	0.728			
Epibatidine, (±)-	0.113 ± 0.005	26070 ± 937	0.730			
Epibestatin hydrochloride	0.110 ± 0.000	35871 ± 2159	1.004			
Epicannabidiol hydrate	0.117 ± 0.011	30878 ± 3621	0.865			
Epicatechin	0.125 ± 0.006	44275 ± 2626	1.240			
Epicatechin monogallate	0.108 ± 0.002	47598 ± 2260	1.333			
Epicatechin pentaacetate	0.103 ± 0.005	35651 ± 2715	0.998			
Epicatechin, (-)-	0.098 ± 0.001 *					
Epieriocalyxin A	0.120 ± 0.001	38296 ± 1871	1.072			
Epiestriol	0.114 ± 0.003	34828 ± 2228	0.975			
epi-Eudesmol	0.128 ± 0.002	37320 ± 900	1.045			
Epifriedelanol	0.116 ± 0.002	39828 ± 2778	1.115			

Epifriedelanol acetate	0.126 ±0.012	40913 ±1471	1.146			
Epigallocatechin	0.120 ±0.003	33615 ±5134	0.941			
Epigallocatechin 3,5-digallate	0.112 ±0.001	47075 ±7642	1.318			
Epigallocatechin gallate	0.118 ±0.008	36449 ±935	1.021			
Epigallocatechin-3-monogallate	0.098 ±0.003	40833 ±2384	1.143			
Epigenetic Multiple Ligand	0.097 ±0.002	42248 ±887	1.183			
Epinastine hydrochloride	0.102 ±0.009	42468 ±3056	1.189			
Epinephrine bitartrate	0.115 ±0.005	19094 ±2651	0.535			
Epinephrine-(+)-tartrate l (-)	0.118 ±0.004	26148 ±2769	0.732			
Epipterosin L	0.127 ±0.004	42065 ±1273	1.178			
Epipterosin L 2'-O-glucoside	0.126 ±0.001	39625 ±3160	1.109			
Epirizole	0.123 ±0.006	53166 ±1785	1.451			
Epirubicin hydrochloride	0.126 ±0.002	23449 ±1854	0.653			
Epistephamiersine	0.117 ±0.001	36591 ±3046	1.025			
Epitaraxerol	0.124 ±0.004	35331 ±1284	0.989			
Epitestosterone	0.112 ±0.005	38291 ±2217	1.072			
Epitiostanol	0.113 ±0.007	32142 ±2972	0.900			
Epitulipinolide	0.120 ±0.004	25925 ±1436	0.726			
Epitulipinolide diepoxide	0.114 ±0.003	5662 ±198	0.158	0.112 ±0.011	23706 ±3480	0.725
Epivogeloside	0.109 ±0.005	47172 ±3483	1.321			
Eplerenone	0.112 ±0.007	35196 ±1096	0.985			
Epoxy (4,5α)-4,5-dihydrosantonin	0.109 ±0.007	37921 ±3445	1.062			
Epoxygedunin	0.111 ±0.009	34813 ±2120	0.975			
Epoxy-oleic acid	0.106 ±0.002	30060 ±1128	0.842			
Epoxyparvinolide	0.111 ±0.008	36154 ±1202	1.012			
Eprinomectin	0.100 ±0.025	25581 ±1146	0.716			
Eprobemide	0.115 ±0.005	31249 ±851	0.875			
Eprodisate disodium	0.106 ±0.003	28040 ±1270	0.785			
Eprosartan	0.128 ±0.018	28795 ±1409	0.806			
Equilin	0.108 ±0.004	59462 ±1748	1.623			
ER 27319 maleate	0.107 ±0.007	25754 ±2538	0.721			
Erastin	0.099 ±0.004	9920 ±378	0.290	0.112 ±0.009	5779 ±540	0.177

Erbstatin analog	0.117 ± 0.008	36290 ± 5337	1.016			
Erdosteine	0.117 ± 0.007	35091 ± 1400	0.983			
Ergocalciferol	0.110 ± 0.002	29254 ± 2737	0.819			
Ergocornine	0.113 ± 0.012	26486 ± 1539	0.742			
Ergonovine maleate	0.110 ± 0.002	31431 ± 948	0.880			
Ergosta-4,6,8(14),22-tetraen-3-one	0.120 ± 0.003	29269 ± 1795	0.820			
Ergosta-5,24(28)-diene-3,7,16-triol	0.109 ± 0.003	28294 ± 1185	0.792			
Ergosterol	0.123 ± 0.001	39619 ± 1879	1.109			
Ergosterol acetate	0.120 ± 0.002	27452 ± 901	0.769			
Ergosterol peroxide	0.125 ± 0.012	41245 ± 2618	1.155			
Ergosterol peroxide 3-O-β-D-glucopyranoside	0.119 ± 0.002	36670 ± 2601	1.027			
Ergotamine D-tartrate	0.113 ± 0.003	35748 ± 3322	1.001			
Ergotamine tartrate	0.111 ± 0.003	29411 ± 4702	0.823			
Ergothioneine	0.116 ± 0.014	32031 ± 1750	0.897			
Erigeside C	0.118 ± 0.008	34940 ± 4611	0.978			
Eriocalyxin B	0.132 ± 0.007	8591 ± 655	0.240	0.124 ± 0.009	27649 ± 2219	0.846
Eriocitrin	0.101 ± 0.003	26077 ± 437	0.730			
Eriodictyol	0.096 ± 0.003 *					
ERK Inhibitor II	0.109 ± 0.012	18479 ± 315	0.547			
ERK Inhibitor III	0.104 ± 0.002	31330 ± 4040	0.877			
Erlotinib	0.110 ± 0.004	69995 ± 7493	1.991			
Erlotinib Hydrochloride	0.109 ± 0.003	54431 ± 3384	1.603			
Ermanin	0.124 ± 0.004	31947 ± 1773	0.894			
Ervamycine	0.127 ± 0.001	31681 ± 5509	0.887			
Erysotramidine	0.128 ± 0.004	32297 ± 520	0.904			
Erysotrine	0.123 ± 0.003	30893 ± 2325	0.865			
Erysubin A	0.119 ± 0.002	37710 ± 1656	1.056			
Erysubin B	0.121 ± 0.001	36878 ± 3553	1.033			
Erythrartine	0.123 ± 0.003	31788 ± 1457	0.890			
Erythrinin C	0.128 ± 0.003	26729 ± 4210	0.748			
Erythristemine	0.118 ± 0.007	39909 ± 3500	1.117			
Erythritol	0.118 ± 0.004	43065 ± 1761	1.206			

Erythro-9-(2-hydroxy-3-nonyl)-adenine hydrochloride	0.109 ± 0.007	40340 ± 7692	1.129			
Erythrodiol	0.121 ± 0.006	44470 ± 1730	1.245			
Erythrodiol 3-palmitate	0.121 ± 0.005	37895 ± 4308	1.061			
Erythro-guaiacylglycerol	0.118 ± 0.005	58912 ± 2336	1.647			
erythro-Guaiacylglycerol β-coniferyl ether	0.126 ± 0.002	50830 ± 8327	1.423			
Erythromycin	0.117 ± 0.006	5333 ± 844	0.146	0.114 ± 0.005	38225 ± 2510	1.170
Erythromycin estolate	0.097 ± 0.003	26571 ± 1783	0.744			
Erythromycin ethylsuccinate	0.098 ± 0.003	1892 ± 371	0.054	0.103 ± 0.008	5756 ± 154	0.176
Erythromycin stearate	0.111 ± 0.006	2611 ± 11	0.075	0.098 ± 0.007	4362 ± 429	0.133
Erythrose	0.115 ± 0.003	39073 ± 1788	1.094			
Erythrosine sodium	0.051 ± 0.005 **					
Erythroxytriol P	0.129 ± 0.006	33770 ± 4967	0.946			
Escitalopram	0.122 ± 0.004	37884 ± 5006	1.061			
Escitalopram oxalat	0.129 ± 0.005	29504 ± 2114	0.826			
Esculentic acid	0.133 ± 0.023	39290 ± 861	1.100			
Esculetin	0.110 ± 0.009	24411 ± 1401	0.683			
Eserine sulfate, physostigmine sulfate	0.115 ± 0.003	35198 ± 2447	0.986			
Esmolol	0.110 ± 0.005	31784 ± 3056	0.890			
Esmolol hydrochloride	0.126 ± 0.003	39074 ± 703	1.094			
Esomeprazole Mg	0.125 ± 0.005	90866 ± 2869	2.530	0.124 ± 0.011	34180 ± 1167	1.046
Esomeprazole potassium	0.122 ± 0.004	29916 ± 2571	0.838			
Estradiol	0.108 ± 0.004	40343 ± 2935	1.130			
Estradiol benzoate	0.099 ± 0.009	31453 ± 558	0.881			
Estradiol cypionate	0.111 ± 0.002	24037 ± 2499	0.673			
Estradiol dipropionate	0.109 ± 0.008	35222 ± 4294	0.986			
Estradiol methyl ether	0.100 ± 0.006	39827 ± 2962	1.115			
Estradiol Valerate	0.107 ± 0.002	35338 ± 4165	0.989			
Estradiol-17 β	0.108 ± 0.012	69201 ± 2646	1.889			
Estradiol-3-methyl-ether	0.105 ± 0.006	44402 ± 7328	1.243			
Estradiol-3-sulfate, sodium salt	0.105 ± 0.008	29886 ± 1540	0.837			
Estragole	0.108 ± 0.012	36150 ± 2052	1.012			
Estramustine	0.105 ± 0.004	35792 ± 3208	1.002			

Estriol	0.111 ± 0.001	46820 ± 1651	1.311			
Estrone	0.113 ± 0.003	50070 ± 5671	1.402			
Estrone benzoate	0.100 ± 0.001	37777 ± 2591	1.058			
Estropipate	0.108 ± 0.012	51806 ± 3077	1.451			
Eszopiclone	0.103 ± 0.003	32325 ± 2267	0.905			
ET-18-OCH3	0.095 ± 0.002	33158 ± 10590	0.928			
Etafenone	0.118 ± 0.003	51602 ± 1471	1.445			
Etanidazole	0.111 ± 0.002	37613 ± 3788	1.053			
Etazolate	0.109 ± 0.001	47591 ± 5752	1.333			
Etazolate hydrochloride	0.095 ± 0.006	35995 ± 921	1.008			
Eterobarb	0.113 ± 0.010	49455 ± 4417	1.385			
Ethacridine	0.063 ± 0.002 **					
Ethacridine lactate	0.079 ± 0.005 **					
Ethacrynic acid	0.087 ± 0.003 **					
Ethambutol dihydrochloride	0.111 ± 0.024	39634 ± 3593	1.110			
Ethamivan	0.119 ± 0.004	67291 ± 3841	1.836			
Ethamsylate	0.112 ± 0.004	46105 ± 828	1.291			
Ethanolamine oleate	0.110 ± 0.014	21850 ± 3223	0.612			
Ethaverine hydrochloride	0.103 ± 0.002	27765 ± 3260	0.777			
Ethenzamide	0.116 ± 0.009	48106 ± 1668	1.347			
Ethinyl estradiol	0.103 ± 0.004	51730 ± 4954	1.448			
Ethinylestradiol	0.113 ± 0.012	72939 ± 1253	1.991			
Ethionamide	0.114 ± 0.003	23707 ± 1181	0.664			
Ethisterone	0.107 ± 0.002	34444 ± 2292	0.964			
Ethopabate	0.107 ± 0.005	28724 ± 1234	0.804			
Ethopropazine hydrochloride	0.107 ± 0.004	34393 ± 1914	0.963			
Ethosuximide	0.099 ± 0.001	45771 ± 1474	1.282			
Ethotoin	0.112 ± 0.005	48657 ± 2154	1.362			
Ethoxyquin	0.114 ± 0.009	66936 ± 11221	1.827			
Ethoxzolamide	0.112 ± 0.009	31541 ± 857	0.883			
Ethyl 2,4-dihydroxyphenylacetate	0.121 ± 0.005	51755 ± 3202	1.449			
Ethyl caffeate	0.113 ± 0.004	34740 ± 1105	0.973			

Ethyl gallate	0.109 ± 0.005	12737 ± 279	0.356	0.124 ± 0.014	31462 ± 4825	0.963
Ethyl glucoside	0.115 ± 0.005	17994 ± 1550	0.503			
Ethyl paraben	0.103 ± 0.004	28847 ± 2830	0.808			
Ethyl vanillin	0.105 ± 0.027	48256 ± 2661	1.351			
Ethyl β-D-fructofuranoside	0.113 ± 0.002	44257 ± 1369	1.239			
Ethyl β-D-ribo-hex-3-ulopyranoside	0.115 ± 0.012	33601 ± 3908	0.941			
Ethylestrenol	0.107 ± 0.005	51582 ± 1227	1.436			
Ethylferulate	0.132 ± 0.004 *					
Ethyllucidone	0.118 ± 0.006	38618 ± 331	1.081			
Ethylnorantifeine	0.118 ± 0.004	40813 ± 8232	1.143			
Ethylnorepinephrine hydrochloride	0.113 ± 0.011	38497 ± 2723	1.078			
Ethylparaben	0.124 ± 0.005	41028 ± 1015	1.149			
Ethyl-β-carboline-3-carboxylate	0.104 ± 0.015	60678 ± 3561	1.699			
Ethynodiol diacetate	0.108 ± 0.002	44908 ± 6295	1.257			
Ethynylestradiol	0.106 ± 0.008	71465 ± 4860	1.990			
Ethynylestradiol 3-methyl ether	0.110 ± 0.005	59385 ± 2536	1.621			
Eticlopride hydrochloride	0.129 ± 0.006	25743 ± 804	0.721			
Etidronate	0.122 ± 0.004	56077 ± 3561	1.651			
Etidronate 2Na	0.117 ± 0.000	27430 ± 1363	0.768			
Etifenin	0.111 ± 0.003	51687 ± 2444	1.447			
Etilefrine hydrochloride	0.111 ± 0.008	51617 ± 1636	1.409			
Etocarlide	0.107 ± 0.003	28117 ± 2330	0.787			
Etodolac	0.091 ± 0.001 *					
Etofenamate	0.109 ± 0.006	39132 ± 2868	1.096			
Etofylline	0.137 ± 0.007 *					
Etomidate	0.121 ± 0.006	29814 ± 824	0.835			
Etomidate; D-isomer	0.114 ± 0.005	44689 ± 3982	1.251			
Etonitazenyl isothiocyanate	0.102 ± 0.011	28903 ± 2034	0.809			
Etoposide	0.117 ± 0.003	51210 ± 2412	1.434			
Etoricoxib	0.112 ± 0.001	51161 ± 3386	1.432			
Etretinate	0.114 ± 0.005	49872 ± 999	1.396			
Etryptamine	0.118 ± 0.005	64991 ± 5336	1.914			

ETYA	0.105 ± 0.005	61535 ± 4565	1.718			
Eucalyptin	0.121 ± 0.008	32526 ± 1287	0.911			
Eucalyptin acetate	0.129 ± 0.003	31719 ± 2598	0.888			
Eucalyptol	0.104 ± 0.003	36726 ± 2625	1.028			
Eucalyptone	0.105 ± 0.004	11811 ± 2482	0.330	0.107 ± 0.010	30472 ± 1846	0.932
Eucamalol	0.130 ± 0.006	31376 ± 2559	0.879			
Eucatropine hydrochloride	0.112 ± 0.001	35425 ± 1885	0.992			
Eudesmine	0.098 ± 0.007	23441 ± 131	0.656			
Eugenol	0.114 ± 0.006	136610 ± 15726	3.814	0.105 ± 0.012	58761 ± 3894	1.798
Eugenol rutinoside	0.124 ± 0.010	56599 ± 7052	1.585			
Euparin	0.114 ± 0.006	41831 ± 3933	1.171			
Eupatorin	0.137 ± 0.002	26427 ± 1336	0.740			
Eupatoriochromene	0.123 ± 0.004	30976 ± 1467	0.867			
Euphol	0.108 ± 0.003	36531 ± 6162	1.023			
Euphorbiasteroid	0.128 ± 0.004	27354 ± 2615	0.766			
Eurycarpin A	0.131 ± 0.006	40820 ± 1805	1.143			
Euscaphic acid	0.107 ± 0.006	29774 ± 4628	0.834			
Euscaphin B	0.119 ± 0.006	37288 ± 2315	1.044			
Euxanthone	0.122 ± 0.004	43046 ± 1067	1.205			
Evans blue	0.089 ± 0.002 **					
Evernic acid	0.109 ± 0.003	41225 ± 2800	1.154			
Everninic acid	0.104 ± 0.003	37088 ± 1385	1.038			
Evodiamine	0.123 ± 0.001	33544 ± 365	0.939			
Evodol	0.108 ± 0.012	41681 ± 4225	1.167			
Evofolin B	0.114 ± 0.004	40489 ± 3674	1.134			
Evoxine	0.123 ± 0.003	46385 ± 1727	1.299			
EX-527	0.103 ± 0.013	62545 ± 4139	1.940			
Exalamide	0.121 ± 0.008	38215 ± 1852	1.070			
Excavatin M	0.116 ± 0.002	32388 ± 1915	0.907			
Excisanin B	0.076 ± 0.007 **					
Exemestane	0.121 ± 0.003	44085 ± 7082	1.234			
Exo1	0.096 ± 0.003	44765 ± 7149	1.253			

Exoticin	0.128 ± 0.005	36406 ± 2507	1.019			
Ezetimibe	0.111 ± 0.010	40594 ± 4039	1.137			
Fadrozole hydrochloride	0.123 ± 0.010	35760 ± 2434	1.001			
Falcarindiol	0.107 ± 0.010	44144 ± 2970	1.236			
Famciclovir	0.121 ± 0.001	50072 ± 3353	1.402			
Famotidine	0.104 ± 0.005	34191 ± 1117	0.957			
Fampridine	0.098 ± 0.003	22973 ± 1246	0.643			
Famprofazone	0.115 ± 0.003	50059 ± 6105	1.402			
Fananserin	0.111 ± 0.004	35859 ± 1831	1.004			
Farnesol	0.117 ± 0.006	41397 ± 4932	1.159			
Farnesylthioacetic acid	0.124 ± 0.003	45561 ± 3206	1.276			
Fascaplysin, Synthetic	0.113 ± 0.007	28603 ± 2113	0.801			
Fasentin	0.102 ± 0.002	143980 ± 12930	4.212	0.104 ± 0.003	61908 ± 6810	1.894
Fast green fcf	0.101 ± 0.001	19271 ± 1060	0.540			
Fasudil hydrochloride	0.096 ± 0.001 *					
FCCP	0.066 ± 0.002 **					
Febuxostat	0.110 ± 0.004	31152 ± 2758	0.872			
Felbamate	0.099 ± 0.007	43490 ± 1021	1.218			
Felbinac	0.107 ± 0.004	41609 ± 4153	1.165			
Felodipine	0.111 ± 0.031	33459 ± 3878	0.937			
Fenaclon	0.104 ± 0.006	37940 ± 3356	1.062			
Fenazepam	0.119 ± 0.005	58658 ± 7354	1.642			
Fenbendazole	0.116 ± 0.013	41075 ± 6407	1.150			
Fenbufen	0.108 ± 0.005	39720 ± 2546	1.112			
Fenbutyramide	0.100 ± 0.002	38248 ± 2706	1.071			
Fenclonine	0.111 ± 0.004	31686 ± 3661	0.887			
Fendiline hydrochloride	0.112 ± 0.009	20069 ± 2691	0.562			
Fenipentol	0.114 ± 0.006	43047 ± 4592	1.205			
Fenobam	0.104 ± 0.004	35863 ± 1066	1.004			
Fenofibrate	0.111 ± 0.002	35621 ± 963	0.997			
Fenofibric acid	0.105 ± 0.006	23729 ± 4256	0.664			
Fenoldipam mesylate	0.106 ± 0.009	48731 ± 7290	1.364			

Fenoldopam hydrobromide	0.108 ± 0.009	42753 ± 1033	1.197			
Fenoldopam mesylate	0.115 ± 0.005	37807 ± 2920	1.059			
Fenoprofen	0.109 ± 0.004	35011 ± 278	0.980			
Fenoprofen calcium salt dihydrate	0.116 ± 0.014	33159 ± 1611	0.928			
Fenoterol hydrobromide	0.105 ± 0.006	30185 ± 386	0.845			
Fenpiverinium bromide	0.062 ± 0.006 **					
Fenretinide	0.119 ± 0.013	34733 ± 1854	0.973			
Fenspiride hydrochloride	0.110 ± 0.007	43246 ± 1309	1.211			
Fentiazac	0.110 ± 0.004	31981 ± 546	0.895			
Fenticlor	0.053 ± 0.004 **					
Fenvalerate	0.120 ± 0.004	32486 ± 1236	0.910			
FERb 033	0.124 ± 0.008	42287 ± 1540	1.184			
Feretoside	0.126 ± 0.003	51494 ± 3611	1.442			
Feroline	0.093 ± 0.005 *					
Ferulamide	0.128 ± 0.003	43194 ± 4886	1.209			
Ferulic acid	0.102 ± 0.002	21655 ± 363	0.643			
Ferutinin	0.100 ± 0.004	25004 ± 356	0.700			
Fervenulin	0.116 ± 0.013	31032 ± 3250	0.869			
Fexaramine	0.111 ± 0.009	36505 ± 1089	1.022			
Fexofenadine hydrochloride	0.124 ± 0.009	40342 ± 3523	1.130			
FG 7142	0.078 ± 0.005 **					
FGF Receptor Tyrosine Kinase Inhibitor	0.097 ± 0.014	31345 ± 2458	0.878			
FGIN-1-27	0.110 ± 0.014	30468 ± 2469	0.853			
FGIN-1-43	0.103 ± 0.008	32124 ± 2601	0.899			
Ficaprenol 11	0.122 ± 0.005	33756 ± 3014	0.945			
Ficusin A	0.126 ± 0.007	37689 ± 2682	1.055			
Fiduxosin hydrochloride	0.083 ± 0.004 **					
Fillalbin	0.102 ± 0.004	50005 ± 2605	1.485			
Finasteride	0.113 ± 0.005	45169 ± 5778	1.265			
Fipexide	0.113 ± 0.010	39673 ± 2394	1.111			
Fipexide hydrochloride	0.121 ± 0.005	55748 ± 1585	1.521			
Fipronil	0.107 ± 0.004	28156 ± 3064	0.788			

Firocoxib	0.101 ± 0.012	40031 ± 1862	1.121			
Fisetin	0.105 ± 0.011	28533 ± 1446	0.799			
Fissinolide	0.114 ± 0.007	31173 ± 3760	0.873			
FIT	0.108 ± 0.007	38711 ± 4802	1.084			
FK 888	0.104 ± 0.002	33598 ± 2118	0.941			
FK-506	0.105 ± 0.003	22383 ± 100	0.627			
Flavanomarein	0.120 ± 0.002	37226 ± 1519	1.042			
Flavanone	0.100 ± 0.007	37145 ± 1679	1.040			
Flavaprin	0.121 ± 0.015	41330 ± 4602	1.157			
Flavodic acid	0.115 ± 0.01	37482 ± 1713	1.049			
Flavokawain A	0.125 ± 0.010	22964 ± 3277	0.643			
Flavokawain B	0.103 ± 0.007	55709 ± 527	1.655			
Flavopiridol (Alvocidib, HMR-1275)	0.120 ± 0.004	33685 ± 6841	0.943			
Flavoteben	0.110 ± 0.014	32534 ± 2729	0.911			
Flavoxate hydrochloride	0.129 ± 0.005	34285 ± 605	0.960			
Flecainide	0.108 ± 0.008	34168 ± 706	0.957			
Flecainide acetate	0.102 ± 0.004	36083 ± 2017	1.010			
Fleroxacin	0.075 ± 0.004 **					
Flindersine	0.108 ± 0.005	59400 ± 5928	1.749			
Flopropione	0.100 ± 0.002	24536 ± 2709	0.687			
Florfenicol	0.098 ± 0.005 *					
Floxuridine	0.089 ± 0.001 **					
Flt-3 Inhibitor	0.113 ± 0.008	24453 ± 3060	0.685			
Flt-3 Inhibitor II	0.071 ± 0.004 **					
Flt-3 Inhibitor III	0.087 ± 0.017 **					
Flubendazol	0.108 ± 0.002	44672 ± 4515	1.251			
Flubendazole	0.125 ± 0.010	47578 ± 936	1.332			
Flucloxacillin sodium	0.126 ± 0.007	47111 ± 1508	1.319			
Fluconazole	0.114 ± 0.002	43026 ± 1783	1.205			
Flucytosine	0.109 ± 0.009	9682 ± 1986	0.264	0.098 ± 0.008	24557 ± 1047	0.751
Fludarabine	0.080 ± 0.003 **					
Fludarabine phosphate	0.084 ± 0.008 **					

Fludrocortisone acetate	0.117 ± 0.007	50990 ± 1759	1.428			
Flufenamic acid	0.119 ± 0.012	32671 ± 2066	0.915			
Flumazenil	0.109 ± 0.002	45126 ± 9405	1.264			
Flumequine	0.056 ± 0.004 **					
Flumethasone	0.105 ± 0.010	51427 ± 2596	1.440			
Flumethazone pivalate	0.112 ± 0.010	58929 ± 4191	1.697			
Flunarizine	0.058 ± 0.006 **					
Flunarizine dihydrochloride	0.064 ± 0.004 **					
Flunisolide	0.111 ± 0.003	40730 ± 1900	1.140			
Flunixin meglumine	0.091 ± 0.005 **					
Fluocinolone acetonide	0.115 ± 0.006	52506 ± 1781	1.470			
Fluocinonide	0.124 ± 0.007	44861 ± 996	1.256			
Fluorescein	0.087 ± 0.002 **					
Fluorocurarine Cl	0.101 ± 0.005	28831 ± 2574	0.807			
Fluorometholone	0.110 ± 0.004	24371 ± 1130	0.682			
Fluoro-SAHA	0.106 ± 0.012	42269 ± 435	1.184			
Fluorotyrosine	0.075 ± 0.003 **					
Fluorouracil	0.089 ± 0.004 **					
Fluorouracilacetate	0.112 ± 0.005	21165 ± 1910	0.593			
Fluoxetine	0.118 ± 0.005	33217 ± 4223	0.930			
Fluoxetine hydrochloride	0.114 ± 0.002	23816 ± 1062	0.667			
Fluperlapine	0.108 ± 0.004	30682 ± 3193	0.859			
Fluphenazine	0.126 ± 0.011	61999 ± 7772	1.826			
Fluphenazine dihydrochloride	0.105 ± 0.007	37749 ± 2449	1.057			
Fluphenazine hydrochloride	0.122 ± 0.001	31190 ± 1990	0.873			
Flupirtine maleate	0.102 ± 0.009	37915 ± 2238	1.062			
Fluprostenol	0.110 ± 0.005	35228 ± 689	0.986			
Flurandrenolide	0.112 ± 0.003	38355 ± 1573	1.074			
Flurbiprofen	0.113 ± 0.002	40912 ± 3608	1.146			
Flurofamide	0.111 ± 0.006	26576 ± 1579	0.744			
Flurothyl	0.109 ± 0.002	26848 ± 2928	0.752			
Fluroxene	0.117 ± 0.008	26522 ± 3146	0.743			

Fluspirilen	0.108 ± 0.006	49173 ± 7321	1.377			
Fluspirilene	0.107 ± 0.003	34126 ± 1948	0.956			
Fluspiriline	0.117 ± 0.003	60849 ± 5536	1.849			
Flutamide	0.106 ± 0.004	64766 ± 3358	1.790			
Fluticasone propionate	0.129 ± 0.017	25572 ± 2133	0.716			
Fluvastatin	0.112 ± 0.009	35214 ± 2177	0.986			
Fluvastatin Na	0.113 ± 0.011	39373 ± 4339	1.102			
Fluviol A	0.122 ± 0.011	47002 ± 8610	1.316			
Fluvoxamine	0.122 ± 0.005	26744 ± 576	0.749			
Fluvoxamine maleate	0.114 ± 0.004	33703 ± 3763	0.944			
Foliamenthoic acid	0.120 ± 0.003	29246 ± 1629	0.819			
Folic acid	0.107 ± 0.012	43946 ± 1460	1.230			
Folinic acid calcium salt	0.107 ± 0.007	38368 ± 3052	1.074			
Fomepizole	0.125 ± 0.009	41114 ± 4527	1.151			
Fomepizole hydrochloride	0.099 ± 0.010	39495 ± 2850	1.106			
Formestane	0.115 ± 0.006	41414 ± 2634	1.160			
Formononetin	0.133 ± 0.002 *					
Formosanol	0.107 ± 0.003	32387 ± 616	0.907			
Formoterol	0.109 ± 0.002	41832 ± 3564	1.171			
Formoterol fumarate	0.121 ± 0.026	21613 ± 2014	0.605			
Formoterol fumarate dihydrate	0.105 ± 0.003	23999 ± 3924	0.672			
Formoterol hemifumarate	0.107 ± 0.003	48499 ± 8045	1.358			
Forrestin A	0.125 ± 0.003	32882 ± 1480	0.921			
Forskolin	0.107 ± 0.002	36605 ± 1430	1.025			
Forskolin G	0.126 ± 0.003	29251 ± 3050	0.819			
Forskolin J	0.129 ± 0.007	40184 ± 3222	1.125			
Foscarnet sodium	0.105 ± 0.002	26412 ± 1048	0.740			
Fosenazide	0.111 ± 0.024	25278 ± 6107	0.708			
Fosfomycin calcium	0.108 ± 0.010	33432 ± 3020	0.936			
Fosfosal	0.108 ± 0.003	41094 ± 2837	1.151			
Fosinopril	0.115 ± 0.002	18533 ± 763	0.519			
FPA 124	0.066 ± 0.010 **					

FPL 55712	0.106 ± 0.005	31281 ± 1560	0.876			
FPL 64176	0.107 ± 0.005	36121 ± 2308	1.011			
FR 122047 hydrochloride	0.106 ± 0.007	18134 ± 1571	0.502			
FR 139317	0.115 ± 0.010	30057 ± 512	0.842			
FR 180204	0.114 ± 0.009	34855 ± 3975	0.976			
Fragransin A2	0.121 ± 0.004	35128 ± 1164	0.984			
Fraxamoside	0.113 ± 0.016	46234 ± 4584	1.295			
Fraxidin	0.118 ± 0.005	35819 ± 3596	1.003			
Fraxidin methyl ether	0.107 ± 0.010	25020 ± 2691	0.701			
Fraxinol	0.123 ± 0.001	48435 ± 3427	1.356			
Fraxiresinol 1-O-glucoside	0.122 ± 0.005	37481 ± 2248	1.049			
Frentizole	0.107 ± 0.018	32458 ± 2747	0.909			
Friedelanol	0.116 ± 0.004	28868 ± 2171	0.808			
Friedelin	0.107 ± 0.001	19463 ± 844	0.578			
FSCPX	0.097 ± 0.006	26484 ± 451	0.742			
Ftaxilide	0.105 ± 0.007	46286 ± 4220	1.296			
Ftivazide	0.113 ± 0.010	37435 ± 3614	1.048			
Ftorafur	0.120 ± 0.005	35397 ± 3703	0.991			
Fuberidazole	0.117 ± 0.005	33617 ± 5449	0.941			
Fucostanol	0.114 ± 0.013	47939 ± 5665	1.342			
Fuegin	0.118 ± 0.004	36699 ± 3913	1.028			
Fulvestrant	0.110 ± 0.006	38218 ± 2950	1.070			
Fumagillin	0.112 ± 0.004	19428 ± 537	0.600			
Fumaric acid	0.124 ± 0.010	33887 ± 2063	0.949			
Fumarprotocetraric acid	0.111 ± 0.005	47964 ± 1801	1.343			
Fumonisin B1	0.118 ± 0.009	60987 ± 7073	1.708			
Fumonisin B2	0.111 ± 0.004	20385 ± 1650	0.605			
Fupenzic acid	0.118 ± 0.004	36796 ± 1529	1.030			
Furafylline	0.112 ± 0.002	50175 ± 6186	1.387			
Furaltadone	0.105 ± 0.001	19591 ± 1172	0.549			
Furaltadone hydrochloride	0.117 ± 0.007	34261 ± 1834	0.959			
Furan-2-carboxylic acid	0.123 ± 0.001	31696 ± 1229	0.887			

Furazolidone	0.108 ± 0.009	33859 ± 2653	0.948			
Furegrelate sodium	0.103 ± 0.004	38231 ± 4004	1.070			
Furomollugin	0.115 ± 0.005	47827 ± 2815	1.339			
Furosemide	0.105 ± 0.007	33577 ± 1558	0.940			
Furoxan	0.111 ± 0.001	28543 ± 2376	0.799			
Fursultiamine Hydrochloride	0.125 ± 0.004	28254 ± 1444	0.791			
Fusaric acid	0.068 ± 0.004 **					
Fusidic acid	0.083 ± 0.002 **					
Fusidic acid sodium	0.063 ± 0.002 **					
Fustin	0.121 ± 0.006	43627 ± 2341	1.222			
G-1	0.107 ± 0.004	38613 ± 3252	1.081			
G-15	0.109 ± 0.006	33902 ± 1302	0.949			
GABA	0.109 ± 0.014	26966 ± 1852	0.755			
Gabaculine	0.109 ± 0.004	25755 ± 2449	0.721			
Gabapentin	0.110 ± 0.003	30309 ± 1904	0.849			
Gaba-picolinoyl-N	0.110 ± 0.014	28882 ± 3066	0.809			
Gabazine	0.103 ± 0.007	30111 ± 869	0.843			
Gabazine bromide	0.108 ± 0.006	46445 ± 2392	1.300			
Gabexate mesilate	0.130 ± 0.008	47934 ± 4087	1.342			
Gabexate mesylate	0.124 ± 0.001	26237 ± 1643	0.735			
Gaboxadol hydrochloride	0.116 ± 0.004	27002 ± 1125	0.756			
Gadoteridol	0.107 ± 0.003	30090 ± 1500	0.843			
Galactitol	0.111 ± 0.009	48521 ± 2309	1.359			
Galangin	0.117 ± 0.004	47533 ± 3407	1.331			
Galangin 3-o-methyl ether	0.115 ± 0.011	32044 ± 1449	0.897			
Galangin trimethyl ether	0.114 ± 0.001	47748 ± 1404	1.337			
Galangine	0.129 ± 0.001 *					
Galanin (1-16)(Porcine,rat)	0.113 ± 0.003	19432 ± 347	0.544			
Galanolactone	0.120 ± 0.007	42936 ± 2898	1.202			
Galanthamine	0.115 ± 0.006	50542 ± 1044	1.415			
Galanthamine hydrobromide	0.099 ± 0.010	38581 ± 3907	1.080			
Gallamine triethiodide	0.102 ± 0.002	39124 ± 2165	1.095			

Gallic acid	0.125 ± 0.002	53403 ± 8024	1.495			
Galosfen	0.111 ± 0.012	23916 ± 6008	0.670			
Gambogic acid	0.105 ± 0.010	41181 ± 2665	1.153			
Ganaxolone	0.106 ± 0.009	37491 ± 917	1.050			
Ganciclovir	0.103 ± 0.007	48451 ± 4023	1.357			
Gangaleoidin	0.107 ± 0.009	33211 ± 3191	0.930			
Ganoderic acid DM	0.123 ± 0.003	40525 ± 1312	1.135			
Ganoderic acid S	0.131 ± 0.010	35926 ± 1660	1.006			
Ganoderiol F	0.121 ± 0.002	34285 ± 2133	0.960			
Ganodermanondiol	0.119 ± 0.005	30814 ± 3268	0.863			
Ganodermanontriol	0.122 ± 0.004	36866 ± 1185	1.032			
Ganoderol A	0.128 ± 0.004	69713 ± 3985	1.948			
Ganoderol B	0.133 ± 0.012	19086 ± 2575	0.533			
Garbanzol	0.114 ± 0.004	49518 ± 3749	1.386			
Garcinexanthone A	0.122 ± 0.003	44296 ± 1610	1.240			
Garciniaxanthone E	0.129 ± 0.009	35199 ± 1800	0.986			
Garcinol	0.108 ± 0.006	34773 ± 5634	0.974			
Garcinolic acid	0.118 ± 0.006	36734 ± 3586	1.029			
Garcinone C	0.134 ± 0.012	70218 ± 10225	1.963			
Gardenin b	0.103 ± 0.010	34842 ± 2835	0.976			
Gardenine	0.127 ± 0.002	29972 ± 1449	0.839			
Gardneramine	0.116 ± 0.005	50144 ± 4501	1.404			
Garjasmin	0.122 ± 0.005	36922 ± 2179	1.034			
Garlicin	0.109 ± 0.005	44069 ± 1704	1.234			
Gartanin	0.115 ± 0.002	45264 ± 3827	1.267			
Gastrodin	0.104 ± 0.006	32304 ± 3192	0.904			
Gatifloxacin	0.064 ± 0.003 **					
Gavestinel	0.101 ± 0.007	39644 ± 5623	1.110			
GBLD 345	0.114 ± 0.009	40386 ± 1937	1.131			
GBR 12783 dihydrochloride	0.075 ± 0.010 **					
GBR 12909 dihydrochloride	0.076 ± 0.013 **					
GBR 12935 dihydrochloride	0.072 ± 0.009 **					

GBR 13069 dihydrochloride	0.070 ± 0.008 **					
GBR-12909	0.073 ± 0.005 **					
GDC-0941	0.123 ± 0.004	40062 ± 2704	1.122			
g-D-Glutamylaminomethylsulfonic acid	0.093 ± 0.008	72154 ± 9720	1.964			
g-D-Glutamylglycine	0.114 ± 0.005	29616 ± 5124	0.829			
Gedunin	0.107 ± 0.007	34213 ± 2173	0.958			
Gefitinib	0.128 ± 0.003	42684 ± 1968	1.195			
Geldanamycin	0.113 ± 0.003	26555 ± 322	0.744			
Gelsemine	0.119 ± 0.004	45593 ± 989	1.277			
Gelsemine hydrochloride	0.104 ± 0.007	28164 ± 1376	0.789			
Gelsemiol	0.114 ± 0.007	39711 ± 2593	1.112			
Gelsevirine	0.112 ± 0.006	38407 ± 3195	1.075			
Gemcitabine	0.069 ± 0.016 **					
Gemcitabine hydrochloride	0.075 ± 0.004 **					
Gemfibrozil	0.116 ± 0.004	39549 ± 2935	1.107			
Gemifloxacin mesylate	0.060 ± 0.010 **					
Geneticin	0.099 ± 0.004	39135 ± 1111	1.096			
Genipin	0.111 ± 0.005	21888 ± 2062	0.605			
Geniposide	0.100 ± 0.001	26654 ± 427	0.746			
Geniposidic acid	0.122 ± 0.002	27284 ± 785	0.764			
Genistein	0.107 ± 0.004	98132 ± 2075	2.748	0.115 ± 0.004	33801 ± 1157	1.034
Genistin	0.108 ± 0.005	24497 ± 609	0.686			
Genkwanin	0.128 ± 0.004	32140 ± 1038	0.900			
Gentamicin sulfate	0.104 ± 0.001	63241 ± 4100	1.822			
Gentamicine sulfate	0.117 ± 0.005	54537 ± 2635	1.488			
Gentamycin sulfate	0.117 ± 0.004	37811 ± 2384	1.059			
Gentian violet	0.077 ± 0.011 **					
Gentianine	0.119 ± 0.007	53222 ± 1543	1.488			
Gentiopicroside	0.117 ± 0.002	38444 ± 3677	1.076			
Gentisic acid	0.117 ± 0.002	51013 ± 1268	1.424			
Geraldol	0.103 ± 0.002	25147 ± 1220	0.704			
Geranylgeraniol	0.112 ± 0.001	35408 ± 3958	0.991			

Geranylgeranoic acid	0.123 ±0.013	37119 ±1356	1.039			
GERI-BP002A	0.121 ±0.003	33309 ±4220	0.933			
Gestrinone	0.112 ±0.013	54581 ±2223	1.528			
GF 109203X	0.124 ±0.007	33627 ±1469	0.942			
Gibberellic acid	0.112 ±0.012	30643 ±1203	0.858			
Gidazepam	0.108 ±0.009	31765 ±1184	0.889			
Gingerol	0.122 ±0.009	60106 ±4232	1.785			
Ginkgolide A	0.128 ±0.003	40477 ±2145	1.133			
Ginkgolide B	0.119 ±0.010	28496 ±1255	0.798			
Ginkgolide C	0.120 ±0.004	48801 ±8229	1.366			
Giparmen	0.107 ±0.006	35692 ±4897	0.999			
Gitoxigenin	0.100 ±0.003	23438 ±305	0.656			
Gitoxigenin diacetate	0.111 ±0.005	37737 ±3117	1.057			
Gitoxin	0.120 ±0.005	39778 ±3760	1.114			
Glabranin	0.133 ±0.005	68651 ±7946	1.919			
Glabridin	0.075 ±0.015 **					
Glafenine	0.112 ±0.003	29990 ±996	0.840			
Glafenine hydrochloride	0.102 ±0.010	44121 ±4881	1.235			
Glaucin B	0.104 ±0.011	36114 ±3118	1.011			
Glaucine	0.106 ±0.004	37190 ±4117	1.041			
Glibenclamide	0.118 ±0.003	36616 ±2145	1.025			
Gliclazide	0.109 ±0.001	45239 ±1717	1.267			
Glimepiride	0.131 ±0.007	42725 ±3544	1.196			
g-Linolenamide	0.122 ±0.004	36195 ±1116	1.013			
g-Linolenic acid (18:3 n-6)	0.121 ±0.006	33433 ±1534	0.936			
g-Linolenoyl alanine	0.135 ±0.005	35137 ±2251	0.984			
g-Linolenoyl dopamine	0.121 ±0.009	28696 ±2106	0.803			
g-Linolenoyl ethanolamide	0.134 ±0.013	41579 ±2423	1.164			
g-Linolenoyl GABA	0.128 ±0.016	33605 ±1481	0.941			
g-Linolenoyl glycine	0.131 ±0.011	31231 ±3212	0.874			
Gliotoxin	0.062 ±0.003 **					
Glipizide	0.101 ±0.005	40206 ±3836	1.126			

Gliquidone	0.108 ± 0.009	37705 ± 2569	1.056			
Globularin	0.123 ± 0.002	33694 ± 4040	0.943			
Glochicoccin D	0.129 ± 0.012	32952 ± 3441	0.923			
Glochidiol	0.138 ± 0.020	33856 ± 1452	0.948			
Glochidionionol C	0.122 ± 0.012	33556 ± 2960	0.940			
Glochidone	0.122 ± 0.006	38811 ± 1369	1.087			
Glochidonol	0.122 ± 0.001	40763 ± 3313	1.141			
Glochionionol A	0.127 ± 0.003	37553 ± 512	1.051			
Glucagon Receptor Antagonist II	0.098 ± 0.002	34420 ± 562	0.964			
Glucitol-4-gucopyanoside	0.109 ± 0.006	39826 ± 3688	1.115			
Glucocorticoid Receptor Modulator, CpdA	0.074 ± 0.003 **					
Glucokinase Activator, Cpd A	0.096 ± 0.003	56310 ± 6755	1.577			
Gluconolactone	0.095 ± 0.004 *					
Glucosamine hydrochloride	0.107 ± 0.010	36866 ± 2525	1.032			
Glucosaminic acid	0.107 ± 0.006	39851 ± 3311	1.116			
Glucosyringic acid	0.120 ± 0.004	34103 ± 4029	0.955			
Glutamate	0.102 ± 0.005	36606 ± 2002	1.025			
Glutamine (L)	0.105 ± 0.002	33166 ± 1628	0.929			
Glutathione	0.121 ± 0.008	38547 ± 7168	1.079			
Glutethimide, para-amino	0.112 ± 0.006	38231 ± 3085	1.070			
Glutinone	0.131 ± 0.002	35746 ± 3094	1.001			
Glyasperin A	0.114 ± 0.002	50004 ± 2894	1.400			
Glybenclamide	0.139 ± 0.003 **					
Glyburide	0.112 ± 0.003	34472 ± 1338	0.965			
Glycerol 1-(26-hydroxyhexacosanoate)	0.114 ± 0.007	31245 ± 1495	0.875			
Glycine	0.106 ± 0.005	30229 ± 3691	0.846			
Glycine, N-[2-[(acetylthio)-methyl]-1-oxo-3-phenylpropyl]-,phenylmethyl ester	0.102 ± 0.016	18623 ± 703	0.519			
Glycocholic acid	0.103 ± 0.006	33396 ± 976	0.935			
Glycocholic acid hydrate	0.125 ± 0.005	39962 ± 3002	1.119			
Glycodeoxycholic acid monohydrate	0.112 ± 0.006	50063 ± 518	1.402			
Glycogen Phosphorylase Inhibitor	0.095 ± 0.006	47253 ± 4672	1.323			

Glycopyrrolate	0.095 ± 0.005 *					
Glycozolinine	0.129 ± 0.025	41703 ± 4378	1.168			
Glycyrrhetinic acid, 18-b -	0.100 ± 0.001	22128 ± 262	0.620			
Glycyrrhizic acid, ammonium salt	0.104 ± 0.008	30518 ± 1225	0.854			
GM 6001	0.098 ± 0.006	50958 ± 4070	1.427			
GNTI dihydrochloride	0.117 ± 0.005	33966 ± 2066	0.951			
G ö 6976	0.119 ± 0.008	24492 ± 1549	0.686			
G ö 6983	0.112 ± 0.010	22287 ± 3006	0.624			
G ö 7874, Hydrochloride	0.090 ± 0.002 **					
Go6976	0.121 ± 0.005	30210 ± 4536	0.846			
Gomisin A	0.137 ± 0.003	31755 ± 1797	0.889			
Gomisin M2	0.121 ± 0.007	48244 ± 2477	1.351			
Goserelin acetate	0.128 ± 0.001 **					
Gossypetin	0.108 ± 0.002	55407 ± 879	1.596			
Gossypin	0.109 ± 0.008	37642 ± 1239	1.054			
Gossypol	0.099 ± 0.008	37246 ± 2977	1.043			
GP 1a	0.107 ± 0.003	41196 ± 1561	1.153			
GP 2a	0.107 ± 0.008	37162 ± 1206	1.041			
GPR109b Agonist	0.096 ± 0.008	39288 ± 6311	1.100			
GPR30 Agonist, G-1	0.108 ± 0.007	39819 ± 1998	1.115			
GPR40 Agonist	0.098 ± 0.001	37622 ± 1120	1.053			
GR 103691	0.123 ± 0.003	26526 ± 844	0.743			
GR 113808	0.106 ± 0.005	44213 ± 6898	1.238			
GR 125487 Sulfamate	0.111 ± 0.002	26609 ± 2188	0.745			
GR 127935 Hydrochloride	0.088 ± 0.004 **					
GR 135531	0.106 ± 0.006	32132 ± 3511	0.900			
GR 144053 Trihydrochloride	0.111 ± 0.008	36865 ± 2609	1.032			
GR 159897	0.111 ± 0.011	26504 ± 1837	0.742			
GR 46611	0.102 ± 0.002	36488 ± 130	1.022			
GR 55562 Dihydrobromide	0.100 ± 0.007	49198 ± 6301	1.378			
GR 79236	0.107 ± 0.009	37417 ± 371	1.048			
GR 79236X	0.102 ± 0.008	32936 ± 2145	0.922			

GR 89696	0.121 ± 0.002	38692 ± 5461	1.083			
GR-128107	0.114 ± 0.009	37605 ± 3825	1.053			
GR-89696 fumarate	0.103 ± 0.005	37436 ± 3648	1.048			
Gramicidin	0.065 ± 0.003 **					
Gramine	0.101 ± 0.003	21259 ± 2110	0.595			
Grandiflorenic acid	0.117 ± 0.008	30023 ± 3509	0.841			
Grandifloroside	0.128 ± 0.008	48508 ± 5459	1.358			
Granisetron	0.119 ± 0.001	33381 ± 1601	0.935			
Granisetron hydrochloride	0.124 ± 0.004	60423 ± 3024	1.682			
Graveoline	0.102 ± 0.007	23924 ± 617	0.670			
Grayanotoxin i	0.108 ± 0.006	40679 ± 3342	1.139			
Grayanotoxin III	0.122 ± 0.012	24865 ± 1063	0.696			
Griffipavixanthone	0.073 ± 0.005 **					
Grifolic acid	0.119 ± 0.007	36869 ± 1466	1.032			
Griselinoside	0.124 ± 0.005	49200 ± 7972	1.378			
Griseofulvin	0.120 ± 0.011	48504 ± 8224	1.358			
Grossamide	0.126 ± 0.001	34631 ± 1346	0.970			
GS 39783	0.106 ± 0.003	27165 ± 2722	0.761			
GSK 0660 GSK 0660	0.139 ± 0.007 *					
GSK 650394	0.110 ± 0.005	30890 ± 1423	0.865			
GSK-3 Inhibitor IX	0.106 ± 0.005	33500 ± 1703	0.938			
GSK-3 Inhibitor IX, MeBIO	0.118 ± 0.008	40717 ± 2348	1.140			
GSK-3 Inhibitor X	0.108 ± 0.004	31942 ± 797	0.894			
GSK-3 Inhibitor XIII	0.111 ± 0.024	40403 ± 1370	1.131			
GSK-3b Inhibitor I	0.110 ± 0.011	31199 ± 1743	0.874			
GSK-3b Inhibitor II	0.110 ± 0.006	36424 ± 4418	1.020			
GSK-3b Inhibitor VIII	0.115 ± 0.004	41347 ± 1959	1.158			
GSK-3b Inhibitor XI	0.102 ± 0.001	27754 ± 846	0.777			
GSK3b Inhibitor XII, TWS119	0.116 ± 0.009	33223 ± 2691	0.930			
GSK-3β Inhibitor XII, TWS119	0.103 ± 0.004	65172 ± 7388	1.907			
GSK690693	0.122 ± 0.003	36291 ± 3590	1.016			
GT-44	0.106 ± 0.006	55667 ± 6124	1.559			

GTP 14564	0.109 ± 0.001	28750 ± 4750	0.805			
Guaiacol	0.110 ± 0.001	42548 ± 1522	1.191			
Guaiacol cinchophenate	0.124 ± 0.007	31637 ± 4206	0.886			
Guaiazulene	0.110 ± 0.003	47684 ± 1063	1.335			
Guaifenesin	0.117 ± 0.004	43593 ± 1749	1.221			
Guaijaverin	0.124 ± 0.005	42568 ± 1188	1.192			
Guaiol	0.098 ± 0.001 *					
Guajadial	0.129 ± 0.006	30352 ± 2418	0.850			
Guanabenz acetate	0.097 ± 0.007	50658 ± 1088	1.400			
Guanadrel sulfate	0.112 ± 0.005	63328 ± 6751	1.728			
Guanethidine monosulfate	0.106 ± 0.005	28742 ± 5667	0.805			
Guanethidine sulfate	0.120 ± 0.006	37849 ± 2368	1.060			
Guanfacine hydrochloride	0.105 ± 0.012	22227 ± 1635	0.622			
Guanidine hydrochloride	0.108 ± 0.005	44848 ± 3324	1.256			
Guanidine, N-cyano-N'-(1,1-dimethylpropyl)-N''-3-pyridinyl	0.117 ± 0.004	69257 ± 6773	1.928			
Guanidinyl-naltrindole di-trifluoroacetate	0.106 ± 0.005	51304 ± 6864	1.436			
Guanosine-5'-diphosphate Na (GDP)	0.103 ± 0.007	20574 ± 597	0.550			
Guanosine-5'-triphosphate Na (GTP)	0.100 ± 0.010	25992 ± 1611	0.728			
Guggulsterone	0.105 ± 0.002	36360 ± 5648	1.018			
Guvacine hydrochloride	0.105 ± 0.005	35110 ± 1167	0.983			
GW 0742	0.112 ± 0.020	27996 ± 3393	0.784			
GW 3965 hydrochloride	0.113 ± 0.002	35201 ± 1422	0.986			
GW 405833	0.115 ± 0.002	35866 ± 1806	1.004			
GW 4064	0.115 ± 0.003	33248 ± 551	0.931			
GW 441756	0.112 ± 0.002	26452 ± 2139	0.741			
GW 5074	0.127 ± 0.005	35245 ± 1455	0.987			
GW 583340 dihydrochloride	0.107 ± 0.002	36661 ± 1973	1.026			
GW 6471	0.107 ± 0.004	28744 ± 1028	0.805			
GW 7647	0.120 ± 0.003	35692 ± 4030	0.999			
GW 843682X	0.112 ± 0.005	38874 ± 2347	1.088			
GW 9508	0.115 ± 0.004	36443 ± 868	1.020			
GW 9662	0.109 ± 0.015	37081 ± 4288	1.038			

GW 1929	0.102 ± 0.004	37154 ± 1180	1.040			
GW 2974	0.104 ± 0.008	29036 ± 1472	0.813			
GW 405833 Hydrochloride	0.095 ± 0.012	34751 ± 2843	0.973			
GYKI 52466 Hydrochloride	0.103 ± 0.006	38770 ± 2522	1.086			
Gynuramide II	0.112 ± 0.006	35868 ± 3483	1.004			
Gyromitrin	0.109 ± 0.002	35117 ± 3220	0.983			
Gyrophoric acid	0.132 ± 0.003	44253 ± 1215	1.239			
Gβγ Modulator II, Gallein	0.091 ± 0.009	60285 ± 1445	1.764			
H-7	0.111 ± 0.002	30104 ± 4789	0.843			
H-8	0.119 ± 0.010	34368 ± 195	0.962			
H-89	0.121 ± 0.002	23418 ± 1081	0.700			
H-9	0.108 ± 0.002	27575 ± 1876	0.772			
HA 1077, Dihydrochloride fasudil	0.118 ± 0.006	26108 ± 1893	0.731			
HA 1100 Hydrochloride	0.115 ± 0.005	31941 ± 1654	0.894			
HA-100	0.107 ± 0.007	38023 ± 721	1.065			
HA-1004	0.121 ± 0.001	37712 ± 1487	1.056			
HA-1004 Hydrochloride	0.101 ± 0.010	36676 ± 949	1.027			
HA1077	0.107 ± 0.003	25780 ± 3058	0.722			
HA14-1	0.108 ± 0.002	37825 ± 3796	1.059			
Haematommic acid, ethyl ester	0.103 ± 0.007	45108 ± 4500	1.263			
Haematoporphyrin	0.110 ± 0.007	23695 ± 1557	0.663			
Haematoxylin	0.113 ± 0.006	31531 ± 3302	0.883			
Haematoxylin pentaacetate	0.099 ± 0.005	38927 ± 2564	1.090			
Hainanmurpanin	0.136 ± 0.003	28100 ± 437	0.787			
Halazone	0.107 ± 0.004	36614 ± 5211	1.025			
Halcinonide	0.111 ± 0.003	34205 ± 844	0.958			
Halofantrine hydrochloride	0.115 ± 0.009	27507 ± 1736	0.770			
Halometasone monohydrate	0.111 ± 0.011	43476 ± 5392	1.217			
Haloperidol	0.107 ± 0.001	41489 ± 1058	1.162			
Haloperidol hydrochloride	0.106 ± 0.010	27512 ± 2550	0.770			
Haloprogin	0.109 ± 0.005	57159 ± 3461	1.560			
Halothane	0.110 ± 0.012	24300 ± 1066	0.680			

Haplopine	0.110 ± 0.002	59291 ± 9467	1.660			
Hardwickiic acid	0.125 ± 0.012	54580 ± 8271	1.528			
Harmalidine	0.124 ± 0.002	35252 ± 905	0.987			
Harmaline	0.124 ± 0.021	31118 ± 999	0.871			
Harmaline hydrochloride	0.102 ± 0.010	26204 ± 728	0.734			
Harmalol hydrochloride	0.097 ± 0.003	18995 ± 5301	0.532			
Harmane	0.097 ± 0.005	35307 ± 1770	0.989			
Harmane hydrochloride	0.099 ± 0.003	47045 ± 1761	1.317			
Harmane-1,2,3,4-tetrahydro-3-carboxylic acid	0.108 ± 0.011	33602 ± 6132	0.941			
Harmine	0.123 ± 0.004	46890 ± 2382	1.313			
Harmine hydrochloride	0.088 ± 0.004 **					
Harmol hydrochloride	0.069 ± 0.007 **					
Harpagoside	0.108 ± 0.007	37008 ± 2651	1.036			
Harringtonine	0.128 ± 0.006	30009 ± 2143	0.840			
HBDDE	0.117 ± 0.004	31341 ± 2338	0.878			
HBDDE (2,2',3,3',4,4'-Hexahydroxy-1,1'-biphenyl-6,6'-dimethanol dimethyl ether)	0.121 ± 0.006	31569 ± 1858	0.884			
HBED	0.098 ± 0.012	113579 ± 13916	3.171	0.099 ± 0.003	203396 ± 14691	6.224
HDBA (2-Hydroxy-5-(2,5-dihydroxybenzylamino)-benzoic acid)	0.129 ± 0.010	39435 ± 3330	1.104			
Hdm2 E3 Ligase Inhibitor	0.116 ± 0.019	25396 ± 5521	0.711			
HEAT hydrochloride	0.103 ± 0.003	29899 ± 2339	0.837			
Heat Shock Protein Inhibitor I	0.105 ± 0.007	44139 ± 3704	1.236			
Hecogenin	0.114 ± 0.008	29451 ± 2130	0.825			
Hecogenin acetate	0.112 ± 0.007	37356 ± 4480	1.046			
Hederagenin	0.113 ± 0.005	40607 ± 2458	1.137			
Hederagonic acid	0.120 ± 0.002	36705 ± 1831	1.028			
Hedgehog Antagonist VIII	0.109 ± 0.008	51311 ± 3712	1.437			
Hedychenone	0.135 ± 0.008	25565 ± 1064	0.716			
Hedyotisol A	0.117 ± 0.011	38439 ± 2307	1.076			
Helenalin	0.081 ± 0.012 **					
Helenin	0.111 ± 0.006	40103 ± 5612	1.123			
Helenine	0.106 ± 0.001	38694 ± 3205	1.083			

Helichrysetin	0.115 ± 0.023	36010 ± 5293	1.008			
Helicin	0.114 ± 0.001	33801 ± 3384	0.946			
Heliomycin	0.109 ± 0.010	37257 ± 3810	1.043			
Heliotrine	0.099 ± 0.005	18167 ± 191	0.540			
Hemado	0.107 ± 0.007	38651 ± 6698	1.082			
Hematein	0.102 ± 0.015	24404 ± 1668	0.683			
Hemicholinium bromide	0.113 ± 0.006	34465 ± 1418	0.965			
Hemicholinium-3	0.095 ± 0.012	45623 ± 4832	1.277			
Hemiphroside A	0.134 ± 0.003	40489 ± 9816	1.134			
Hemiphroside B	0.129 ± 0.003	32005 ± 6768	0.896			
Heneicosanoic acid	0.135 ± 0.014	38567 ± 1314	1.080			
Henryoside	0.123 ± 0.003	32819 ± 1384	0.919			
Hentriacontane	0.122 ± 0.019	29550 ± 339	0.827			
Hepoxilin A3	0.103 ± 0.003	30547 ± 1035	0.855			
Hepoxilin B3	0.106 ± 0.003	27182 ± 812	0.761			
Heptadecanoic acid	0.132 ± 0.007 *					
Heptaminol hydrochloride	0.118 ± 0.009	39368 ± 2012	1.102			
Heraclenin	0.113 ± 0.005	39073 ± 310	1.094			
Heraclenol	0.126 ± 0.013	43253 ± 1362	1.211			
Heraclenol 3'-O-[β-D-apiofuranosyl-(1-6)-β-D-glucopyranoside]	0.115 ± 0.005	29434 ± 954	0.824			
Heraclenol 3'-O-β-D-glucopyranoside	0.125 ± 0.010	30292 ± 2136	0.848			
Heraclenol acetonide	0.131 ± 0.012	33968 ± 2615	0.951			
Herbimycin A, Streptomyces sp.	0.124 ± 0.032	29001 ± 1000	0.812			
Herkinorin	0.111 ± 0.006	24751 ± 702	0.693			
Hernandezine	0.105 ± 0.006	25254 ± 850	0.707			
Herniarin	0.097 ± 0.006	24361 ± 1336	0.682			
Hesperetin	0.119 ± 0.013	52185 ± 7111	1.461			
Hesperetin 7-O-glucoside	0.115 ± 0.013	43140 ± 1134	1.208			
Hesperetine	0.133 ± 0.006 *					
Hesperidin	0.115 ± 0.002	36491 ± 5384	1.022			
Hesperidine	0.125 ± 0.004	32161 ± 2235	0.900			
Hetacillin potassium	0.058 ± 0.007 **					

Heteratisine	0.097 ± 0.004 *					
Heteronoside	0.109 ± 0.013	35795 ± 549	1.002			
Heteropeucenin, methyl ether	0.105 ± 0.007	33160 ± 4315	0.928			
Heudelotinone	0.118 ± 0.007	39677 ± 6114	1.111			
Heveaflavone	0.128 ± 0.006	28831 ± 701	0.807			
Hexachlorophene	0.104 ± 0.008	20605 ± 3294	0.577			
Hexacosyl (E)-ferulate	0.129 ± 0.015	30226 ± 3541	0.846			
Hexadecanoic acid	0.126 ± 0.025	23539 ± 2128	0.659			
Hexahydrosiladifenidol	0.118 ± 0.003	44244 ± 5331	1.239			
Hexahydro-sila-difenidol hydrochloride, p-fluoro analog	0.100 ± 0.009	45393 ± 2575	1.271			
Hexamethonium	0.097 ± 0.006	43140 ± 5325	1.208			
Hexamethonium bromide	0.105 ± 0.006	36751 ± 1896	1.029			
Hexamethonium dibromide dihydrate	0.108 ± 0.009	37557 ± 1869	1.052			
Hexamethonium dichloride	0.102 ± 0.005	38056 ± 2741	1.066			
Hexamethylene-bis-[dimethyl-(3-phthalimidopropyl)-ammonium] bromide	0.102 ± 0.005	33213 ± 3048	0.930			
Hexamethylquercetagetin	0.108 ± 0.004	37451 ± 2905	1.049			
Hexestrol	0.129 ± 0.004 *					
Hexetidine	0.107 ± 0.006	28332 ± 1559	0.793			
Hexylcaine hydrochloride	0.114 ± 0.003	60396 ± 3478	1.648			
Hexylene glycol	0.105 ± 0.005	46017 ± 3566	1.288			
Hexylresorcinol	0.085 ± 0.003 **					
Hh Signaling Antagonist VII, JK184	0.081 ± 0.001 **					
Hh/Gli Antagonist, GANT58	0.080 ± 0.006 **					
Hh/Gli Antagonist, GANT61	0.098 ± 0.006	34605 ± 1573	0.969			
Hieracin	0.111 ± 0.002	17501 ± 663	0.504			
Hierochin D	0.124 ± 0.008	35728 ± 4505	1.000			
HIF-1 Inhibitor	0.100 ± 0.002	37532 ± 808	1.051			
Himbacine	0.099 ± 0.004	33357 ± 4923	0.934			
Hinokiol	0.121 ± 0.009	23188 ± 2040	0.649			
Hinokitiol	0.105 ± 0.005	17112 ± 1409	0.520			
Hirsutanonol	0.121 ± 0.002	37540 ± 2845	1.051			
Hirsutanonol 5-O-glucoside	0.126 ± 0.008	42078 ± 5054	1.178			

Hirsutenone	0.108 ± 0.004	48604 ± 4791	1.361			
Hirsutine	0.097 ± 0.004 *					
Hispidin	0.086 ± 0.005 *					
Hispidone	0.126 ± 0.003	32505 ± 1839	0.910			
Hispidulin	0.118 ± 0.016	44285 ± 373	1.240			
Histamine	0.115 ± 0.004	26199 ± 775	0.734			
Histamine dihydrochloride	0.102 ± 0.003	31542 ± 979	0.883			
Histamine, R(-)-α-methyl-, dihydrochloride	0.106 ± 0.006	41558 ± 1080	1.164			
Histone Acetyltransferase Activator, CTB	0.107 ± 0.006	46400 ± 2163	1.299			
Histone Deacetylase Inhibitor III	0.102 ± 0.008	48103 ± 623	1.347			
Histone Deacetylase Inhibitor IV	0.102 ± 0.004	51200 ± 2670	1.434			
Histone Deacetylase Inhibitor VI, HNHA	0.135 ± 0.007 *					
Histone Lysine Methyltransferase Inhibitor	0.097 ± 0.002	14117 ± 506	0.413	0.103 ± 0.005	42313 ± 4522	1.295
HMS3229O07	0.107 ± 0.006	4497 ± 520	0.133	0.098 ± 0.009	9025 ± 368	0.276
HNMPA (Hydroxy-2-naphthalenylmethylphosphonic acid)	0.117 ± 0.011	37476 ± 745	1.049			
HNMPA-(AM)3	0.117 ± 0.007	30671 ± 2134	0.859			
Hoechst 33342 (cell permeable) (BisBenzimide)	0.086 ± 0.001 **					
Homalomenol A	0.129 ± 0.007	44605 ± 8976	1.249			
Homaloside D	0.129 ± 0.019	35154 ± 4434	0.984			
Homatropine hydrobromide	0.101 ± 0.006	34939 ± 3424	0.978			
Homatropine methylbromide	0.107 ± 0.003	42226 ± 2736	1.182			
Homidium bromide	0.070 ± 0.003 **					
Homobutein	0.099 ± 0.004	42641 ± 1299	1.194			
Homochlorcyclizine dihydrochloride	0.115 ± 0.006	40965 ± 5163	1.147			
Homoeriodictyol (-)	0.108 ± 0.004	60789 ± 2136	1.702			
Homoharringtonine	0.101 ± 0.001	24689 ± 3029	0.691			
Homoorientin	0.111 ± 0.000	48381 ± 600	1.437			
Homopterocarpin	0.104 ± 0.016	44723 ± 2595	1.252			
Homoquinolinic acid	0.106 ± 0.007	53452 ± 1593	1.497			
Homosalate	0.113 ± 0.011	44950 ± 5100	1.259			
Homoveratrylamine	0.133 ± 0.005 *					
Honokiol	0.129 ± 0.006	63692 ± 1737	1.892			

Hop-17(21)-en-3-ol	0.124 ± 0.021	35366 ± 4056	0.990			
Hordenine sulfate	0.103 ± 0.003	20904 ± 304	0.621			
Horminone	0.134 ± 0.014	33269 ± 3348	0.932			
HSP90 Inhibitor, CCT018159	0.100 ± 0.002	91562 ± 2306	2.679	0.105 ± 0.009	38614 ± 2616	1.183
HTMT	0.116 ± 0.001	17958 ± 931	0.500			
HTMT dimaleate	0.120 ± 0.006	20434 ± 612	0.565			
Humantenine	0.113 ± 0.007	31801 ± 1133	0.890			
Humantenmine	0.110 ± 0.009	40871 ± 1679	1.144			
Humulene epoxide II	0.113 ± 0.007	40863 ± 5117	1.144			
Huperzine A	0.130 ± 0.003 *					
Huperzine A [(-)-Huperzine A]	0.109 ± 0.003	30780 ± 2140	0.862			
Hycanthone	0.113 ± 0.004	39775 ± 2764	1.114			
Hydralazine hydrochloride	0.101 ± 0.004	40403 ± 1428	1.131			
Hydrangenol	0.116 ± 0.000	57940 ± 4674	1.619			
Hydrangenol 8-O-glucoside	0.119 ± 0.003	36096 ± 2395	1.011			
Hydrangenoside A dimethyl acetal	0.130 ± 0.004	32263 ± 1941	0.903			
Hydrastine (1r, 9s)	0.134 ± 0.009 *					
Hydrastine, D-b -	0.119 ± 0.005	20979 ± 130	0.623			
Hydrastinine hydrochloride	0.101 ± 0.007	21128 ± 2282	0.592			
Hydrochlorothiazide	0.103 ± 0.016	34560 ± 1506	0.968			
Hydrocortisone	0.103 ± 0.004	29576 ± 2553	0.828			
Hydrocortisone 21-acetate	0.115 ± 0.013	22440 ± 1485	0.628			
Hydrocortisone acetate	0.104 ± 0.009	47636 ± 6151	1.334			
Hydrocortisone base	0.116 ± 0.004	56269 ± 3012	1.536			
Hydrocortisone butyrate	0.109 ± 0.003	18158 ± 384	0.523			
Hydrocortisone hemisuccinate	0.111 ± 0.006	33593 ± 4724	0.941			
Hydrocortisone phosphate triethylamine	0.115 ± 0.009	66924 ± 5685	1.928			
Hydrocortisone propionate	0.108 ± 0.014	35626 ± 3774	0.998			
Hydrocortisone valerate	0.103 ± 0.010	35361 ± 5099	0.990			
Hydrocotarnine hydrobromide	0.103 ± 0.009	24638 ± 1727	0.690			
Hydroflumethiazide	0.115 ± 0.007	54605 ± 6373	1.529			
Hydrolysis product of bussein	0.118 ± 0.002	32837 ± 2497	0.919			

Hydroprotopine	0.118 ± 0.012	43110 ± 3914	1.207			
Hydroquinidine	0.124 ± 0.003	19224 ± 903	0.554			
Hydroquinine hydrobromide hydrate	0.102 ± 0.006	32769 ± 1760	0.918			
Hydroquinone	0.098 ± 0.007	39304 ± 2816	1.100			
Hydroxyamphetamine hydrobromide	0.102 ± 0.002	35127 ± 2087	0.984			
Hydroxycamptothecin, 10-	0.126 ± 0.013	25834 ± 1658	0.723			
Hydroxychavicol	0.121 ± 0.007	18839 ± 2755	0.527			
Hydroxychloroquine sulfate	0.117 ± 0.003	50676 ± 4259	1.419			
Hydroxyflavone, 7-	0.110 ± 0.014	17527 ± 1178	0.521			
Hydroxygenkwanin	0.127 ± 0.023	39727 ± 4929	1.112			
Hydroxylamine hydrochloride	0.100 ± 0.006	45329 ± 2180	1.269			
Hydroxyprogesterone	0.121 ± 0.001	26911 ± 1192	0.753			
Hydroxyprogesterone caproate	0.110 ± 0.015	48057 ± 3949	1.346			
Hydroxyprogesterone heptanoate	0.111 ± 0.019	29804 ± 1546	0.834			
Hydroxytacrine maleate	0.103 ± 0.003	36083 ± 568	1.010			
Hydroxytacrine maleate (R,S)	0.108 ± 0.009	55683 ± 3176	1.520			
Hydroxytoluic acid	0.114 ± 0.006	25667 ± 429	0.719			
Hydroxytropinone, 6-	0.110 ± 0.001	43055 ± 1011	1.206			
Hydroxytuberosone	0.126 ± 0.004	32457 ± 804	0.909			
Hydroxyurea	0.094 ± 0.007	34758 ± 588	0.973			
Hydroxyzine dihydrochloride	0.119 ± 0.001	41473 ± 3181	1.161			
Hydroxyzine pamoate	0.095 ± 0.008 *					
Hymechrome	0.103 ± 0.016	37631 ± 8677	1.054			
Hymecromone	0.110 ± 0.002	39063 ± 3312	1.094			
Hymecromone methyl ether	0.107 ± 0.019	31724 ± 2134	0.888			
Hyoscyamine	0.102 ± 0.012	24671 ± 2909	0.691			
Hyoscyamine (L)	0.122 ± 0.011	35914 ± 6191	1.006			
Hyperforin	0.118 ± 0.004	35787 ± 3148	1.002			
Hypericin	0.136 ± 0.013	22222 ± 1673	0.664			
Hyperin	0.103 ± 0.006	45011 ± 1281	1.260			
Hyperoside	0.116 ± 0.003	25212 ± 514	0.706			
Hypocrellin A	0.103 ± 0.002	47692 ± 481	1.416			

Hypocrellin B	0.114 ± 0.003	61944 ± 1820	1.840			
Hypophyllanthin	0.121 ± 0.003	35041 ± 1543	0.981			
Hypotaurine	0.102 ± 0.008	36937 ± 2462	1.034			
Hypoxanthine	0.114 ± 0.009	21713 ± 941	0.608			
Hyptadienic acid	0.121 ± 0.001	45628 ± 2040	1.278			
Hyrcanoside	0.105 ± 0.008	34020 ± 849	0.953			
Hythiemoside A	0.125 ± 0.001	39725 ± 2148	1.112			
IAA-94	0.110 ± 0.004	23224 ± 654	0.650			
Ibandronate	0.112 ± 0.002	37755 ± 4757	1.057			
Ibandronate sodium	0.105 ± 0.005	54781 ± 6011	1.534			
IBC 293	0.102 ± 0.002	23955 ± 4172	0.671			
IB-MECA	0.096 ± 0.002	36031 ± 3943	1.009			
IBMX	0.113 ± 0.001	32913 ± 2822	0.922			
Ibogaine	0.125 ± 0.013	33196 ± 2361	0.929			
Ibotenic acid	0.099 ± 0.006	42270 ± 7429	1.184			
Ibudilast	0.103 ± 0.006	40078 ± 4687	1.122			
Ibuprofen	0.109 ± 0.006	42016 ± 2349	1.176			
Ibuproxam	0.106 ± 0.002	54295 ± 7189	1.520			
Ibutilide fumarate	0.125 ± 0.007	42537 ± 1795	1.191			
IC 261	0.108 ± 0.006	31591 ± 3354	0.885			
IC87114	0.132 ± 0.006	27562 ± 1867	0.772			
Icariin	0.121 ± 0.003	37508 ± 2695	1.050			
Icariside E5	0.124 ± 0.002	37740 ± 1535	1.057			
ICI 182,780	0.107 ± 0.004	24978 ± 558	0.699			
ICI 192,605	0.108 ± 0.007	28921 ± 3431	0.810			
ICI 199,441	0.114 ± 0.010	46931 ± 6308	1.314			
ICI 204,448	0.106 ± 0.005	40478 ± 3371	1.133			
ICI 215,001	0.105 ± 0.001	28366 ± 4296	0.794			
ICI 63,137	0.121 ± 0.007	43956 ± 6437	1.231			
ICI 63197	0.120 ± 0.011	40719 ± 1951	1.140			
ICI 89406	0.099 ± 0.011	53152 ± 5051	1.488			
ICI-118,551	0.109 ± 0.004	24731 ± 3489	0.692			

ICI-162,846	0.103 ± 0.011	31070 ± 4604	0.870			
ICI-185,282	0.107 ± 0.004	27129 ± 2796	0.760			
ICI-89406	0.121 ± 0.005	32125 ± 3369	0.899			
Icilin	0.094 ± 0.003	38719 ± 3484	1.084			
ICRF-154	0.115 ± 0.006	35120 ± 2375	0.983			
ICRF-193	0.114 ± 0.006	32682 ± 4907	0.915			
Idarubicin	0.058 ± 0.006 **					
Idarubicin hydrochloride	0.085 ± 0.005 **					
Idazoxan	0.110 ± 0.012	23932 ± 3306	0.670			
Idazoxan hydrochloride	0.095 ± 0.001	29321 ± 4446	0.821			
Idebenone	0.115 ± 0.004	22430 ± 3871	0.628			
Idoxuridine	0.073 ± 0.011 **					
IDRA 21	0.115 ± 0.005	29752 ± 2628	0.833			
Idramantone	0.112 ± 0.008	48947 ± 6564	1.370			
IEM 1460	0.105 ± 0.006	28570 ± 4132	0.800			
Ifenprodil	0.115 ± 0.010	43305 ± 2931	1.213			
Ifenprodil Hemitartrate	0.111 ± 0.002	24584 ± 1367	0.688			
Ifenprodil tartrate	0.110 ± 0.002	28185 ± 3220	0.789			
Ifosfamide	0.115 ± 0.004	26147 ± 1622	0.732			
IGF-1R Inhibitor II	0.061 ± 0.007 **					
Ikarugamyin	0.096 ± 0.007	248949 ± 49780	7.565	0.101 ± 0.005	64859 ± 4691	1.985
IKK 16	0.108 ± 0.001	32831 ± 1123	0.919			
IKK Inhibitor VII	0.060 ± 0.005 **					
IKK Inhibitor X	0.115 ± 0.016	31063 ± 1793	0.870			
IKK-2 Inhibitor IV	0.106 ± 0.007	37295 ± 3562	1.044			
IKK-2 Inhibitor V	0.125 ± 0.005	36441 ± 7754	1.020			
IKK-2 Inhibitor VI	0.109 ± 0.004	33910 ± 1445	0.949			
IKK-2 Inhibitor VIII	0.112 ± 0.004	40671 ± 1548	1.139			
IKK-2 Inhibitor XI	0.122 ± 0.006	53469 ± 5547	1.497			
IKK-3 Inhibitor IX	0.119 ± 0.025	34712 ± 1847	0.972			
Ikshusterol	0.130 ± 0.003	34646 ± 1303	0.970			
Ikshusterol 3-O-β-D-glucopyranoside	0.138 ± 0.007	36026 ± 4595	1.009			

IL-2Rα Antagonist	0.093 ± 0.005 *					
Ilicic acid	0.122 ± 0.006	35040 ± 2443	0.981			
Ilicol	0.115 ± 0.006	33378 ± 1408	0.935			
Iloprost	0.113 ± 0.001	43880 ± 703	1.229			
Imatinib	0.113 ± 0.001	44347 ± 2623	1.242			
Imatinib mesylate	0.124 ± 0.003	45982 ± 5609	1.287			
Imazodan	0.099 ± 0.005	32271 ± 1244	0.904			
Imbricatolic acid	0.135 ± 0.005	34641 ± 5795	0.970			
IMD 0354	0.108 ± 0.004	41820 ± 1542	1.171			
Imetit dihydrobromide	0.102 ± 0.005	22346 ± 983	0.626			
Imexon	0.103 ± 0.004	24231 ± 1735	0.678			
IMID-4F hydrochloride	0.103 ± 0.008	41949 ± 2930	1.175			
Imidazol-4-ylacetic acid sodium salt	0.091 ± 0.002 **					
Imidazole-4-acetic acid hydrochloride	0.106 ± 0.007	28485 ± 2055	0.798			
Imidazole-4-acetic acid sodium salt dihydrate	0.112 ± 0.006	35855 ± 3024	1.004			
Imidurea	0.111 ± 0.002	28862 ± 1274	0.808			
Imiloxan hydrochloride	0.118 ± 0.007	40570 ± 4342	1.136			
Imipenem	0.115 ± 0.003	33172 ± 1760	0.929			
Imipramine hydrochloride	0.107 ± 0.005	25903 ± 4996	0.725			
Imiquimod	0.105 ± 0.015	31352 ± 1626	0.878			
Immepip dihydrobromide	0.115 ± 0.010	30979 ± 3954	0.867			
Immethridine dihydrobromide	0.101 ± 0.004	23634 ± 2179	0.662			
Impentamine dihydrobromide	0.109 ± 0.002	40038 ± 4568	1.121			
Imperatorin	0.098 ± 0.003	36555 ± 2378	1.024			
Imperialine	0.101 ± 0.012	35300 ± 4682	0.988			
Inamrinone	0.106 ± 0.002	41628 ± 4994	1.166			
INCA-6	0.114 ± 0.002	40753 ± 4027	1.141			
Indapamide	0.135 ± 0.003 **					
Indatraline	0.110 ± 0.010	12412 ± 1147	0.346	0.115 ± 0.008	17234 ± 2068	0.527
Indatraline hydrochloride	0.106 ± 0.006	20343 ± 2805	0.562			
Indigo	0.124 ± 0.005	47084 ± 4959	1.318			
Indinavir sulphate	0.113 ± 0.003	31259 ± 1616	0.875			

Indiplon	0.113 ± 0.001	27875 ± 2092	0.780			
Indirubin	0.114 ± 0.009	33123 ± 1786	0.927			
Indirubin Derivative E804	0.104 ± 0.010	22946 ± 1212	0.642			
Indirubin-3′-monoxime, 5-Iodo-	0.115 ± 0.002	30089 ± 8420	0.842			
Indirubin-3'-monoxime	0.115 ± 0.007	21148 ± 2084	0.592			
Indirubin-3'-oxime	0.098 ± 0.001	47418 ± 2489	1.328			
Indocaine	0.111 ± 0.002	45308 ± 803	1.269			
Indole-3-acrylic acid methyl ester	0.121 ± 0.013	71345 ± 8842	1.994			
Indole-3-butyric acid	0.096 ± 0.003 *					
Indole-3-carbinol	0.100 ± 0.021	21483 ± 1117	0.602			
Indomethacin	0.106 ± 0.004	28033 ± 4042	0.785			
Indoprofen	0.128 ± 0.013	45792 ± 2468	1.282			
Ingenol	0.103 ± 0.004	26053 ± 122	0.729			
Ingenol 3,20-dibenzoate	0.123 ± 0.012	46041 ± 2141	1.289			
INH1	0.115 ± 0.008	36948 ± 2724	1.035			
Inosine	0.104 ± 0.007	49814 ± 5507	1.395			
Inositol	0.123 ± 0.007	37885 ± 4958	1.061			
Integrin αMβ2 Ligand	0.112 ± 0.01	47309 ± 6669	1.325			
Iobenguane sulfate	0.107 ± 0.003	38612 ± 1893	1.081			
Iocetamic acid	0.100 ± 0.006	26795 ± 2690	0.750			
Iodipamide	0.110 ± 0.026	36900 ± 2457	1.033			
Iodixanol	0.108 ± 0.003	56466 ± 3494	1.541			
Iodoacetamide	0.088 ± 0.006 *					
Iodophenpropit dihydrobromide	0.105 ± 0.000	11963 ± 1238	0.320	0.109 ± 0.008	20714 ± 1487	0.634
Iodoquinol	0.101 ± 0.002	103852 ± 9635	2.991	0.105 ± 0.006	49846 ± 3816	1.525
Iofetamine hydrochloride	0.108 ± 0.004	31711 ± 4522	0.888			
Iohexol	0.113 ± 0.004	40069 ± 2603	1.122			
I-OMe-Tyrphostin AG 538	0.117 ± 0.006	37721 ± 4648	1.056			
Ionomycin	0.113 ± 0.003	30477 ± 2935	0.853			
Iopamidol	0.129 ± 0.013	39092 ± 1006	1.095			
Iopanic acid	0.105 ± 0.002	26825 ± 1628	0.751			
Iopanoic acid	0.107 ± 0.009	37467 ± 3199	1.049			

Iopromide	0.117 ±0.006	40080 ±4196	1.122			
Iothalamic acid	0.101 ±0.001	18606 ±519	0.536			
Ioversol	0.106 ±0.006	44306 ±4428	1.241			
Ioxaglic acid	0.111 ±0.010	40707 ±1515	1.140			
Ioxilan	0.112 ±0.006	25436 ±1898	0.712			
IP3K Inhibitor	0.126 ±0.013	63517 ±7230	1.881			
IPA 3	0.107 ±0.001	19586 ±759	0.542			
IPAG	0.097 ±0.015	20913 ±1495	0.579			
Ipratropium	0.097 ±0.007	32964 ±2527	0.923			
Ipratropium bromide	0.098 ±0.007	35067 ±1890	0.982			
Ipriflavone	0.127 ±0.005	48043 ±3110	1.345			
Iproheptine	0.107 ±0.007	22634 ±471	0.634			
Iproniazid	0.107 ±0.004	40815 ±1708	1.143			
Iproniazid phosphate	0.105 ±0.001	30692 ±2636	0.859			
Iproniazid sulfate	0.107 ±0.007	38783 ±1605	1.086			
Iproniazide phosphate	0.125 ±0.005	47284 ±2872	1.324			
Ipsapirone	0.112 ±0.007	45318 ±2321	1.269			
IQ-1	0.105 ±0.005	50973 ±6775	1.427			
IRAK-1/4 Inhibitor	0.115 ±0.002	26527 ±2909	0.743			
IRAK-1/4 Inhibitor I	0.105 ±0.006	37530 ±9100	1.051			
IRBESARTAN	0.132 ±0.007	41420 ±5885	1.160			
Iressa	0.109 ±0.009	39440 ±1678	1.104			
Iretol	0.108 ±0.007	40473 ±2531	1.133			
Iridin	0.117 ±0.002	35024 ±1682	0.981			
Iriflophenone	0.132 ±0.002	44835 ±1985	1.255			
Iriflophenone 2-O-α-L-rhamnopyranoside	0.134 ±0.006	28487 ±2477	0.798			
Iriflophenone 3-C-β-D-glucopyranoside	0.114 ±0.002	37475 ±624	1.049			
Irigenin	0.115 ±0.001	45791 ±3979	1.282			
Irigenin trimethyl ether	0.109 ±0.002	35025 ±1835	0.981			
Irigenin, dibenzyl ether	0.111 ±0.007	35512 ±2287	0.994			
Irigenol	0.106 ±0.003	32826 ±1086	0.919			
Iriginol hexaacetate	0.104 ±0.002	33232 ±946	0.930			

Irinotecan	0.119 ±0.007	38401 ±1919	1.075			
Irinotecan Hydrochloride	0.109 ±0.004	40343 ±2397	1.130			
IRL-2500	0.094 ±0.009	40699 ±1175	1.140			
Iron Chelator, Dp44mT	0.081 ±0.007 **					
Irsogladine maleate	0.107 ±0.007	32028 ±3234	0.897			
Isamoltane hemifumarate	0.097 ±0.011	52450 ±4954	1.469			
Isaxonine	0.107 ±0.003	38895 ±1879	1.089			
Isbufylline	0.109 ±0.014	49679 ±3462	1.391			
Isoacteoside	0.116 ±0.003	38429 ±1933	1.076			
Isoapetalic acid	0.114 ±0.009	42276 ±3456	1.184			
Isoastilbin	0.119 ±0.004	38977 ±2025	1.091			
Isobavachin	0.120 ±0.002	50735 ±8294	1.421			
isobergaptene	0.113 ±0.006	27398 ±2933	0.767			
Isobonducellin	0.121 ±0.008	37696 ±2884	1.055			
Isoboonein	0.116 ±0.005	39340 ±1901	1.101			
Isoboonein acetate	0.133 ±0.004	22504 ±947	0.630			
Isobutamben	0.106 ±0.005	29586 ±864	0.828			
Isobutylmethylxanthine	0.106 ±0.003	44006 ±8717	1.232			
Isocalamendiol	0.117 ±0.007	41742 ±3305	1.169			
Isocarapanaubine	0.133 ±0.005	38548 ±1665	1.079			
Isocarboxazid	0.117 ±0.003	42289 ±1582	1.184			
Isoconazole	0.116 ±0.003	56321 ±2391	1.537			
Isocoronarin D	0.126 ±0.004	40190 ±2738	1.125			
Isocoronarin D methylthiomethyl ether	0.122 ±0.005	31117 ±1214	0.871			
Isocorydine hydrochloride	0.100 ±0.002	25976 ±3869	0.727			
Isocostic acid	0.118 ±0.003	43374 ±296	1.214			
Isocupressic acid	0.122 ±0.004	34799 ±1257	0.974			
Isocycloheximide	0.107 ±0.005	37214 ±1895	1.042			
Isodiospyrin	0.125 ±0.002	34689 ±2511	0.971			
Isodunnianol	0.132 ±0.005	41399 ±944	1.159			
Isoetharine mesylate	0.109 ±0.009	43044 ±7568	1.205			
Isoferulic acid	0.125 ±0.002	36962 ±2708	1.035			

Isofervenulin	0.108 ±0.002	32797 ±4429	0.918			
Isoflupredone	0.108 ±0.005	53039 ±5341	1.485			
Isoflupredone acetate	0.121 ±0.006	34467 ±6309	0.965			
Isoformononetin	0.097 ±0.004	24784 ±1435	0.694			
Isofuranodiene	0.125 ±0.001	38595 ±1930	1.081			
Isogarciniaxanthone E	0.129 ±0.005	37633 ±4587	1.054			
Isogomisin O	0.135 ±0.002	28586 ±669	0.800			
Isogosferol	0.124 ±0.001	24762 ±2313	0.693			
Isogranulatimide	0.121 ±0.005	19681 ±674	0.551			
Isoguanosine	0.101 ±0.005	48940 ±1995	1.370			
Isoguvacine hydrochloride	0.107 ±0.013	29658 ±2073	0.830			
Isohemiphloin	0.129 ±0.004	43054 ±1272	1.205			
Isohyenanchin	0.137 ±0.005	21512 ±1409	0.602			
Isokobusone	0.109 ±0.002	41267 ±5980	1.155			
Isolariciresinol	0.120 ±0.002	41597 ±2564	1.165			
Isoleucine (L)	0.104 ±0.009	33210 ±2437	0.930			
Isolicoflavonol	0.123 ±0.006	40341 ±1347	1.130			
Isoliquiritigenin	0.099 ±0.010	61486 ±3764	1.699			
Isomagnolone	0.123 ±0.006	36679 ±2012	1.027			
Isomartynoside	0.120 ±0.002	43637 ±4330	1.222			
Isomedicarpin	0.126 ±0.003	59674 ±3011	1.668			
Isomeranzin	0.119 ±0.004	42205 ±5450	1.182			
Isometheptene mucate	0.122 ±0.006	34938 ±1247	0.978			
Isomexoticin	0.138 ±0.003	28291 ±1011	0.792			
Isoniazid	0.105 ±0.006	36237 ±3945	1.015			
Iso-Nicotinamide	0.105 ±0.010	23195 ±1671	0.649			
Isonicotinic acid	0.107 ±0.001	34484 ±2583	0.966			
Isonicotinoyl Gaba	0.106 ±0.008	38560 ±1944	1.080			
Isonipecotic acid	0.106 ±0.005	24022 ±2169	0.673			
Isonormangostin	0.114 ±0.006	29941 ±3434	0.838			
iso-Olomoucine	0.120 ±0.003	34406 ±4092	0.963			
Isoosajin	0.118 ±0.003	45025 ±3506	1.261			

Isopentenyladenine	0.116 ± 0.005	43292 ± 4519	1.212			
Isopentyl Salicylate	0.114 ± 0.008	32570 ± 2909	0.912			
Isopeonol	0.101 ± 0.011	37284 ± 2165	1.044			
Isopimpinellin	0.106 ± 0.011	34820 ± 2654	0.975			
Isopomiferin	0.105 ± 0.008	42285 ± 2868	1.184			
Isopropamide iodide	0.114 ± 0.004	69167 ± 8457	1.888			
Isoproterenol hydrochloride	0.104 ± 0.002	40253 ± 2723	1.127			
Isopulegol	0.051 ± 0.013 **					
Isopyrin hydrochloride	0.107 ± 0.011	49810 ± 6147	1.395			
Isoquercitrin	0.129 ± 0.003	48422 ± 4066	1.356			
Isoquercitrine	0.099 ± 0.007	21454 ± 495	0.637			
Isoquinoline, 6,7-dimethoxy-1-methyl-1,2,3,4-tetrahydro, hydrochloride	0.106 ± 0.002	46375 ± 1118	1.298			
Isorauhimbine	0.107 ± 0.006	39518 ± 1540	1.106			
Isoreserpine, (-)-	0.101 ± 0.005	25206 ± 364	0.706			
Isorhamnetin	0.124 ± 0.005	43884 ± 1798	1.229			
Isorhamnetine-3-O-glucoside	0.106 ± 0.004	30285 ± 262	0.848			
Isorhamnetine-3-O-rutinoside	0.104 ± 0.023	16890 ± 1202	0.502			
Isorhoifolin	0.121 ± 0.004	36122 ± 1901	1.011			
Isorhoifoline	0.101 ± 0.010	28146 ± 220	0.788			
Isorotenone	0.113 ± 0.009	32637 ± 3940	0.914			
Isosakuranetin	0.098 ± 0.011	62545 ± 877	1.858			
Isosalvipuberulin	0.122 ± 0.006	42500 ± 1878	1.190			
Isosativan	0.116 ± 0.010	40741 ± 4089	1.141			
Isosaxalin	0.125 ± 0.003	40835 ± 3502	1.143			
Isoscopoletine	0.101 ± 0.015	19536 ± 1218	0.580			
Isosorbide	0.111 ± 0.002	34126 ± 3025	0.956			
Isosorbide dinitrate	0.118 ± 0.002	31969 ± 1139	0.895			
Isosorbide mononitrate	0.110 ± 0.011	62490 ± 6007	1.705			
Isosorbide-2-mononitrate	0.117 ± 0.004	36418 ± 3811	1.020			
Isotachioside	0.119 ± 0.004	33218 ± 5278	0.930			
Isotectorigenin, 7-methyl ether	0.118 ± 0.006	31008 ± 3379	0.868			
Isotetrandrine	0.121 ± 0.009	33127 ± 379	0.928			

Isotetrandrine N-2'-oxide	0.120 ± 0.008	43514 ± 3357	1.218			
Isotharine mesylate	0.106 ± 0.002	28875 ± 3232	0.808			
Isothymusin	0.111 ± 0.014	39101 ± 1702	1.095			
Isotretinoin	0.116 ± 0.009	32744 ± 652	0.917			
Isotretinon	0.101 ± 0.005	24532 ± 2971	0.687			
Isotrimethoprim	0.111 ± 0.004	50193 ± 8367	1.405			
Isotschimgin	0.118 ± 0.003	41071 ± 1271	1.150			
Isovaleramide	0.103 ± 0.008	33537 ± 2979	0.939			
Isovitexin	0.104 ± 0.004	26706 ± 366	0.748			
Isowighteone	0.127 ± 0.006	30430 ± 1041	0.852			
Isoxanthopterin	0.106 ± 0.002	19365 ± 2557	0.535			
Isoxicam	0.104 ± 0.003	37959 ± 1225	1.063			
Isoxsuprine hydrochloride	0.115 ± 0.002	35216 ± 2666	0.986			
Isradipine	0.111 ± 0.002	33325 ± 3922	0.933			
Itavastatin Ca	0.111 ± 0.005	25788 ± 2601	0.722			
Itol A	0.115 ± 0.008	32588 ± 1390	0.912			
Itopride	0.109 ± 0.008	35395 ± 241	0.991			
Itopride hydrochloride	0.114 ± 0.003	26314 ± 7098	0.737			
Itraconazole	0.115 ± 0.010	34284 ± 4500	0.960			
ITSA1	0.102 ± 0.005	30559 ± 1550	0.856			
Ivermectin	0.100 ± 0.002	30293 ± 6086	0.848			
Izalpinin	0.095 ± 0.009	32969 ± 2050	0.923			
Jacaranone ethyl ester	0.130 ± 0.004	30903 ± 5365	0.865			
Jaceidin	0.120 ± 0.005	35329 ± 433	0.989			
Jaceidin triacetate	0.120 ± 0.008	27773 ± 1234	0.778			
JAK Inhibitor I	0.124 ± 0.001	22271 ± 2351	0.624			
JAK2 Inhibitor III, SD-1029	0.099 ± 0.005	23137 ± 1002	0.648			
JAK3 Inhibitor II	0.081 ± 0.007 **					
JAK3 Inhibitor IV	0.123 ± 0.008	30198 ± 3522	0.846			
JAK3 Inhibitor VI	0.078 ± 0.013 **					
Jangomolide	0.117 ± 0.005	48530 ± 2440	1.359			
Jasmone	0.109 ± 0.008	38469 ± 3484	1.077			

Jasmonic acid	0.106 ± 0.011	32418 ± 892	0.908			
Jatrorrhizine	0.123 ± 0.002	42052 ± 1806	1.177			
Jervine	0.112 ± 0.006	40967 ± 1475	1.147			
JFD00244	0.102 ± 0.002	43630 ± 3188	1.222			
JK 184	0.106 ± 0.004	50046 ± 3800	1.401			
JL-18	0.095 ± 0.004	36012 ± 3603	1.008			
JLK 6	0.117 ± 0.001	32302 ± 333	0.904			
JNJ 10191584 Maleate	0.111 ± 0.006	23545 ± 3699	0.659			
JNJ 16259685	0.106 ± 0.003	34259 ± 3597	0.959			
JNJ 17203212	0.111 ± 0.002	40200 ± 1735	1.126			
JNJ38877605	0.119 ± 0.003	32670 ± 1656	0.915			
JNJ7706621	0.123 ± 0.005	37836 ± 4350	1.059			
JNK Inhibitor	0.103 ± 0.006	40953 ± 1591	1.147			
JNK Inhibitor II	0.118 ± 0.013	31496 ± 1822	0.882			
JNK Inhibitor IX	0.106 ± 0.003	41419 ± 3952	1.160			
JNK Inhibitor V	0.107 ± 0.006	29014 ± 2907	0.812			
JNK Inhibitor VIII	0.110 ± 0.003	33456 ± 1333	0.937			
Josamycin	0.067 ± 0.003 **					
JS-K	0.099 ± 0.007	44523 ± 4874	1.247			
JTC 801	0.106 ± 0.004	24208 ± 1619	0.678			
JTE 013	0.102 ± 0.004	35436 ± 2491	0.992			
JTE 907	0.107 ± 0.007	28714 ± 990	0.804			
Juarezic acid	0.099 ± 0.002	35626 ± 6692	0.998			
Juglone	0.121 ± 0.027	17337 ± 1416	0.527			
Junipediol B	0.109 ± 0.007	40547 ± 2900	1.135			
JWH-015	0.101 ± 0.004	25274 ± 932	0.708			
JX 401	0.111 ± 0.008	36886 ± 1748	1.033			
K 185	0.105 ± 0.006	29191 ± 3711	0.817			
K 858	0.109 ± 0.006	38960 ± 3779	1.091			
K114	0.101 ± 0.009	34519 ± 711	0.967			
K252A	0.114 ± 0.004	23562 ± 231	0.660			
Kadsuracoccinic acid A	0.115 ± 0.004	30519 ± 2564	0.855			

Kadsuric acid	0.121 ±0.003	29530 ±2126	0.827			
Kaempferide	0.120 ±0.007	20379 ±1599	0.570			
Kaempferitrin	0.105 ±0.010	18785 ±2081	0.525			
Kaempferol	0.098 ±0.006	62332 ±1653	1.851			
Kaempferol 3,4',7-triacetate	0.123 ±0.001	35796 ±3199	1.002			
Kaempferol 3-neohesperidoside	0.123 ±0.005	41347 ±5914	1.158			
Kaempferol 3-O-(6"-O-acetyl)-glucoside-7-O-rhamnoside	0.132 ±0.015	45505 ±4718	1.274			
Kaempferol 3-O-alfa-L-arabinoside	0.122 ±0.001	43368 ±2499	1.214			
Kaempferol tetraacetate	0.122 ±0.002	36525 ±3759	1.023			
Kaempferol-3-O-glucoside	0.098 ±0.006	20708 ±994	0.615			
Kaempferol-7-neohesperidoside	0.101 ±0.003	26449 ±172	0.741			
Kaerophyllin	0.120 ±0.002	40883 ±3270	1.145			
Kahweol	0.097 ±0.003 *					
Kainic Acid	0.110 ±0.002	30929 ±2641	0.866			
Kamebanin	0.132 ±0.007	1751 ±163	0.049	0.113 ±0.006	12338 ±1081	0.378
Kanamycin	0.100 ±0.004	29741 ±825	0.833			
Kanamycin A sulfate	0.114 ±0.002	42892 ±746	1.201			
Karakoline	0.104 ±0.013	34872 ±8145	0.976			
Karatavic acid	0.106 ±0.006	34328 ±899	0.961			
Karavilagenin A	0.115 ±0.002	51237 ±5588	1.435			
Karavilagenin D	0.120 ±0.009	40460 ±2057	1.133			
Kasugamycin hydrochloride	0.114 ±0.004	37157 ±487	1.040			
Kavain (+/-)	0.117 ±0.009	31187 ±2148	0.873			
Kawain	0.101 ±0.004	37405 ±2251	1.047			
Kazinol B	0.132 ±0.002	38569 ±3107	1.080			
Kazinol U	0.124 ±0.005	32097 ±1442	0.899			
KB-R7943	0.099 ±0.002	60514 ±3512	1.770			
KB-R7943 mesylate	0.112 ±0.011	20766 ±2153	0.581			
Kelampayoside A	0.125 ±0.005	45265 ±7599	1.267			
Kenpaullone	0.123 ±0.003	28786 ±2930	0.806			
Keratinocyte Differentiation Inducer	0.114 ±0.003	48690 ±3815	1.363			
Ketanserin Tartrate	0.109 ±0.001	45235 ±2599	1.267			

Ketanserin tartrate hydrate	0.119 ± 0.009	37273 ± 962	1.044			
Ketoconazole	0.110 ± 0.006	29132 ± 5709	0.816			
Ketopinic acid	0.096 ± 0.005	28413 ± 408	0.796			
Ketoprofen	0.102 ± 0.004	29080 ± 761	0.814			
Ketoprofen+D153	0.108 ± 0.003	31215 ± 1775	0.874			
Ketorolac tris salt	0.101 ± 0.006	25641 ± 4225	0.718			
Ketotifen fumarate	0.106 ± 0.004	23888 ± 2512	0.669			
KF 38789	0.104 ± 0.003	38236 ± 3727	1.071			
Khayalenoid E	0.109 ± 0.004	42485 ± 3167	1.190			
Khayanthone	0.116 ± 0.004	27600 ± 1731	0.773			
Khellin	0.111 ± 0.002	25041 ± 446	0.701			
Khivorin	0.113 ± 0.004	32920 ± 1013	0.922			
Ki 8751	0.112 ± 0.006	29680 ± 1828	0.831			
Ki20227 (+/-)	0.117 ± 0.006	37219 ± 3156	1.042			
KIH-402	0.126 ± 0.012	38401 ± 6183	1.075			
Kinetin	0.102 ± 0.001	25451 ± 485	0.713			
Kinetin riboside	0.101 ± 0.009	33301 ± 1201	0.932			
Kirenol	0.134 ± 0.016	40005 ± 3258	1.120			
Kisspeptin-13 (4-13)(human)	0.112 ± 0.004	21084 ± 1589	0.590			
Kitasamycin	0.071 ± 0.006 **					
KN-62	0.128 ± 0.003	39703 ± 1245	1.112			
KN-92	0.133 ± 0.006	12418 ± 411	0.368	0.122 ± 0.005	34182 ± 3364	1.046
KN-93	0.115 ± 0.005	23320 ± 1403	0.697			
Koaburaside	0.119 ± 0.002	32604 ± 1260	0.913			
Koaburaside monomethyl ether	0.119 ± 0.001	34929 ± 391	0.978			
Kobusone	0.123 ± 0.006	42643 ± 2496	1.194			
Kolavenol	0.127 ± 0.004	32451 ± 1333	0.909			
Kongensin A	0.120 ± 0.013	5961 ± 661	0.167	0.115 ± 0.008	12644 ± 554	0.387
Kongensin A acetate	0.101 ± 0.011	19941 ± 2837	0.558			
Koparin	0.100 ± 0.013	18435 ± 945	0.531			
Koumine	0.111 ± 0.010	38944 ± 1720	1.090			
KT5720	0.115 ± 0.018	30249 ± 2292	0.847			

KU 55933	0.114 ± 0.006	33947 ± 741	0.950			
KU0063794	0.123 ± 0.003	26149 ± 7492	0.732			
KU14R	0.128 ± 0.006	29902 ± 2400	0.837			
Kumatakenin	0.129 ± 0.003	48407 ± 3922	1.355			
Kynuramine	0.111 ± 0.009	26084 ± 1073	0.730			
Kynurenic Acid	0.107 ± 0.004	37251 ± 2708	1.043			
Kynurenine	0.111 ± 0.007	34146 ± 2662	0.956			
Kyotorphin	0.107 ± 0.006	43529 ± 3056	1.219			
L 655240	0.101 ± 0.017	35408 ± 1825	0.991			
L 670596	0.101 ± 0.011	38018 ± 3193	1.064			
L 755507	0.106 ± 0.003	29775 ± 3110	0.834			
L-(-)-Epinephrine-(+)-bitartrate	0.122 ± 0.006	25104 ± 3810	0.703			
L-(-)-Norepinephrine bitartrate	0.101 ± 0.022	31347 ± 2295	0.878			
L-(-)-Norepinephrine-(+)-bitartrate	0.104 ± 0.006	23430 ± 2133	0.656			
L-(-)-Threo-3-hydroxyaspartic acid	0.098 ± 0.012	41154 ± 4373	1.152			
L-(-)-vesamicol hydrochloride	0.106 ± 0.001	45028 ± 5817	1.261			
L-(-)-α-Methyldopa	0.107 ± 0.005	37037 ± 2374	1.037			
L-(+)-2-Amino-4-phosphonobutanoic acid	0.106 ± 0.004	43195 ± 4478	1.209			
L-(+)-2-Amino-5-phosphonovaleric acid	0.104 ± 0.010	37481 ± 6043	1.049			
L-(+/-)-alliin	0.122 ± 0.006	36971 ± 1774	1.035			
L-152,804	0.117 ± 0.006	42132 ± 2247	1.180			
L-161,982	0.112 ± 0.002	40542 ± 381	1.135			
L-162,313	0.100 ± 0.006	33799 ± 4021	0.946			
L-165,041	0.101 ± 0.009	45876 ± 4566	1.285			
L-168,049	0.104 ± 0.005	38020 ± 2024	1.065			
L-2-aminoadipic acid	0.102 ± 0.005	44493 ± 3371	1.246			
L-3,4-Dihydroxyphenylalanine	0.098 ± 0.006	31941 ± 504	0.894			
L-3,4-Dihydroxyphenyl-alanine	0.110 ± 0.005	32493 ± 1876	0.910			
L-3,4-Dihydroxyphenylalanine methyl ester hydrochloride	0.099 ± 0.011	32087 ± 1258	0.898			
L-368,899 hydrochloride	0.117 ± 0.001	28937 ± 1555	0.810			
L-655,708	0.102 ± 0.009	35382 ± 1162	0.991			
L-687,384 hydrochloride	0.101 ± 0.003	27419 ± 1070	0.768			

L-689,560	0.105 ±0.007	43448 ±6836	1.217			
L-690,330	0.104 ±0.004	23672 ±2362	0.663			
L-693,403	0.106 ±0.007	37991 ±3113	1.064			
L-694,247	0.121 ±0.004	64350 ±4158	1.792			
L-701,252	0.105 ±0.007	38945 ±9090	1.090			
L-701,324	0.109 ±0.004	32781 ±4384	0.918			
L-732,138	0.101 ±0.004	41759 ±2437	1.169			
L-733,060	0.104 ±0.008	19992 ±2534	0.553			
L-741,626	0.099 ±0.009	32837 ±4820	0.919			
L-741,742	0.114 ±0.004	33913 ±4680	0.950			
L-744,832	0.078 ±0.006 **					
L-745,870	0.116 ±0.010	25534 ±1099	0.715			
L-750,667	0.105 ±0.006	24377 ±1304	0.683			
L-765,314	0.110 ±0.010	39417 ±2079	1.104			
L-798,106	0.103 ±0.001	42851 ±2084	1.200			
Labd-13-ene-8,15-diol	0.123 ±0.002	42393 ±3680	1.187			
Labetalol hydrochloride	0.108 ±0.007	31336 ±3641	0.877			
Laccaic acid a	0.115 ±0.006	17570 ±2023	0.506			
Lacidipine	0.120 ±0.004	50196 ±6133	1.405			
Lacinilene C	0.129 ±0.014	49509 ±3433	1.386			
Lacitol	0.103 ±0.018	26827 ±2423	0.751			
Lactobionic acid	0.107 ±0.002	31188 ±2334	0.873			
Lactose monohydrate	0.109 ±0.007	31077 ±1335	0.870			
Lactulose	0.107 ±0.005	30480 ±4444	0.853			
Lagochilin	0.108 ±0.006	34075 ±2813	0.954			
Lagochiline	0.122 ±0.005	38025 ±2753	1.065			
L-allylglycine	0.110 ±0.010	43204 ±5987	1.210			
Lamiide	0.124 ±0.005	32876 ±2026	0.921			
Lamivudine	0.129 ±0.003	45966 ±2512	1.287			
Lamotrigine	0.103 ±0.006	45618 ±2116	1.277			
Lanatoside C	0.113 ±0.010	31407 ±1097	0.879			
Lancifolin C	0.121 ±0.006	33936 ±2626	0.950			

Lanosterol	0.113 ± 0.011	58238 ± 8902	1.631			
Lanosterol acetate	0.123 ± 0.016	42918 ± 4518	1.202			
Lansiumarin C	0.132 ± 0.005	31323 ± 2973	0.877			
Lansoprazole	0.108 ± 0.007	39360 ± 973	1.102			
L-Anti-endo-3,4-methanopyrrolidinedicarboxylic acid	0.108 ± 0.006	37110 ± 1981	1.039			
L-AP4	0.100 ± 0.004	33232 ± 1945	0.930			
Lapachol	0.120 ± 0.006	29262 ± 1520	0.819			
Lapachone, b -	0.120 ± 0.011	43964 ± 1482	1.231			
Lapatinib	0.129 ± 0.005	37760 ± 3129	1.057			
Lapidine	0.112 ± 0.003	22079 ± 590	0.618			
Lapiferine	0.097 ± 0.005 *					
Lappaconitine	0.123 ± 0.005	37958 ± 2616	1.063			
L-Arginine	0.098 ± 0.002	34014 ± 6100	0.952			
Lariciresinol acetate	0.135 ± 0.009	31658 ± 2528	0.886			
Lariciresinol dimethyl ether	0.130 ± 0.007	34991 ± 3367	0.980			
Lariciresinol-4,4'-dimethyl ether-9-acetate	0.116 ± 0.002	39263 ± 2940	1.099			
Larixinic acid	0.104 ± 0.003	39644 ± 1638	1.110			
Larixol	0.113 ± 0.002	41604 ± 3779	1.165			
Larixol acetate	0.109 ± 0.004	32796 ± 1420	0.918			
Lasalocid A Na	0.081 ± 0.006 **					
L-Aspartic acid	0.108 ± 0.002	26200 ± 3548	0.734			
Latanoprost	0.125 ± 0.03	37088 ± 2067	1.038			
Lathosterol	0.114 ± 0.006	33740 ± 3050	0.945			
Latrunculin A, Latrunculia magnifica	0.100 ± 0.004	46239 ± 4533	1.295			
Latrunculin B	0.113 ± 0.004	42013 ± 3544	1.176			
Laudanosoline hydrobromide	0.094 ± 0.029	61416 ± 3520	1.824			
Laurifoline	0.118 ± 0.005	40636 ± 3880	1.138			
Lauroscholtzine	0.125 ± 0.005	27115 ± 1829	0.759			
Lavendustin A	0.121 ± 0.004	34352 ± 1898	0.962			
Lavendustin B	0.120 ± 0.001	35277 ± 600	0.988			
Lawsone	0.086 ± 0.003 **					
Lazabemide hydrochloride	0.115 ± 0.001	29897 ± 4329	0.837			

L-azetidine-2-carboxylic acid	0.099 ± 0.004	34659 ± 1831	0.970			
L-buthionine sulfoximine	0.101 ± 0.009	43736 ± 3469	1.225			
L-Buthionine-sulfoximine	0.096 ± 0.002	34580 ± 1177	0.968			
L-Canavanine	0.096 ± 0.010	37380 ± 2311	1.047			
L-Canavanine sulfate	0.101 ± 0.004	34906 ± 2075	0.977			
L-cis-Diltiazem	0.106 ± 0.001	42011 ± 2161	1.176			
Lck Inhibitor	0.126 ± 0.004	19857 ± 1510	0.556			
L-Cycloserine	0.099 ± 0.004	33941 ± 5204	0.950			
L-Cysteine sulfinic acid	0.108 ± 0.004	34574 ± 5248	0.968			
L-Cysteinesulfinic Acid	0.104 ± 0.010	33465 ± 1259	0.937			
L-deoxyalliin	0.111 ± 0.012	34976 ± 2652	0.979			
LE 135	0.109 ± 0.003	32081 ± 4133	0.898			
LE 300	0.099 ± 0.011	43026 ± 843	1.205			
Leachianone A	0.120 ± 0.006	26801 ± 339	0.750			
Lecanoric acid	0.113 ± 0.003	45471 ± 2104	1.273			
Leflunomide	0.106 ± 0.004	70761 ± 4154	1.956			
Lenalidomide	0.108 ± 0.012	35574 ± 1613	0.996			
Leoidin	0.113 ± 0.005	38127 ± 1041	1.068			
Lercanidipine Hydrochloride	0.071 ± 0.005 **					
Lercanidipine hydrochloride hemihydrate	0.077 ± 0.006 **					
Lethedioside A	0.115 ± 0.006	37810 ± 4202	1.059			
Lethedoside A	0.114 ± 0.008	43357 ± 2773	1.214			
Letrozole	0.108 ± 0.002	50632 ± 5506	1.418			
Leucomisine	0.106 ± 0.004	26312 ± 183	0.737			
Leuconolam	0.122 ± 0.003	44317 ± 2234	1.241			
Leucoside	0.118 ± 0.002	39537 ± 1947	1.107			
Leucovorin calcium	0.103 ± 0.006	47527 ± 7427	1.331			
Leukotoxin A (9,10-EODE)	0.116 ± 0.006	28193 ± 1156	0.789			
Leukotoxin B (12,13-EODE)	0.108 ± 0.007	31775 ± 1292	0.890			
Leukotriene B3	0.103 ± 0.001	32075 ± 1424	0.898			
Leukotriene B4	0.106 ± 0.006	32658 ± 2016	0.914			
Leukotriene C4	0.106 ± 0.003	29638 ± 2434	0.830			

Leukotriene D4	0.107 ± 0.003	26264 ± 1498	0.735			
Leukotriene E4	0.113 ± 0.005	24620 ± 1174	0.689			
Leupeptin	0.112 ± 0.003	23459 ± 1333	0.657			
Leu-Valorphin-Arg	0.109 ± 0.002	39764 ± 7679	1.113			
Levalbuterol Hydrochloride	0.108 ± 0.005	38586 ± 2869	1.080			
Levallorphan tartrate	0.100 ± 0.007	34044 ± 972	0.953			
Levamisole hydrochloride	0.121 ± 0.004	32558 ± 967	0.912			
Levcromakalim	0.099 ± 0.004 *					
levcycloserine	0.090 ± 0.006	32563 ± 2700	0.912			
Levetiracetam	0.107 ± 0.011	36921 ± 2920	1.034			
Levobunolol hydrochloride	0.104 ± 0.009	24528 ± 1586	0.687			
Levocabastine hydrochloride	0.113 ± 0.001	46075 ± 3765	1.290			
Levocarnitine	0.104 ± 0.016	28007 ± 1218	0.784			
Levocarnitine propionate hydrochloride	0.107 ± 0.007	24600 ± 4078	0.689			
Levocetirizine	0.118 ± 0.005	34772 ± 6283	0.974			
Levocetirizine dihydrochloride	0.100 ± 0.003	32279 ± 1332	0.904			
Levodopa	0.110 ± 0.004	41160 ± 2430	1.152			
Levofloxacin	0.115 ± 0.006	25602 ± 2456	0.717			
Levofloxacin hydrochloride	0.064 ± 0.006 **					
Levomenthol	0.115 ± 0.005	28552 ± 335	0.799			
Levonordefrin	0.117 ± 0.010	51842 ± 3411	1.452			
Levonorgestrel	0.116 ± 0.007	20538 ± 2320	0.575			
Levopropoxyphene napsylate	0.110 ± 0.005	28279 ± 1633	0.792			
Levosimendan	0.092 ± 0.004 *					
Levosulpiride	0.118 ± 0.008	30315 ± 5282	0.849			
Levothyroxine	0.106 ± 0.013	30945 ± 6280	0.866			
Levulinic acid, 3-benzylidenyl-	0.096 ± 0.007	17803 ± 514	0.513			
LFM-A13	0.113 ± 0.006	24287 ± 1927	0.680			
L-Glutamic acid	0.105 ± 0.006	35432 ± 5758	0.992			
L-Glutamic acid hydrochloride	0.103 ± 0.007	35743 ± 846	1.001			
L-Glutamic acid, N-[4-[[(2,4-diamino-6-pteridinyl)-methyl]methylamino]benzoyl]	0.126 ± 0.008	45023 ± 5828	1.261			

L-Glutamic acid, N-phthaloyl-	0.103 ± 0.007	41414 ± 3742	1.160			
L-Glutamine	0.116 ± 0.012	34084 ± 1381	0.954			
L-Histidine hydrochloride	0.101 ± 0.007	40233 ± 5914	1.127			
L-Homocysteinesulfinic acid	0.092 ± 0.010	36983 ± 2948	1.036			
L-Hyoscyamine	0.095 ± 0.007	38144 ± 2419	1.068			
Liangshanin A	0.118 ± 0.006	4296 ± 258	0.120	0.109 ± 0.013	4554 ± 132	0.139
Licarbazepine	0.108 ± 0.011	35187 ± 4366	0.985			
Licarin A	0.120 ± 0.007	50505 ± 3542	1.414			
Licochalcone-A, Synthetic	0.078 ± 0.003 **					
Lidocaine	0.114 ± 0.003	29452 ± 4280	0.825			
Lidocaine hydrochloride	0.104 ± 0.005	39226 ± 4123	1.098			
Lidocaine N-ethyl bromide quaternary salt	0.103 ± 0.009	27062 ± 2950	0.758			
Lidoflazine	0.118 ± 0.005	59336 ± 7218	1.619			
Ligustroside	0.115 ± 0.008	41410 ± 3279	1.159			
Limonexic acid	0.115 ± 0.007	40533 ± 931	1.135			
Limonin	0.126 ± 0.001	36695 ± 1032	1.027			
Limonol	0.114 ± 0.013	34692 ± 700	0.971			
Linagliptin	0.100 ± 0.009	48528 ± 5212	1.359			
Linamarin	0.115 ± 0.006	29493 ± 3056	0.826			
Linarin	0.125 ± 0.006	37331 ± 1567	1.045			
Lincomycin	0.118 ± 0.009	24501 ± 1345	0.686			
Lincomycin hydrochloride	0.114 ± 0.004	42487 ± 1172	1.190			
Lindane	0.099 ± 0.009	25790 ± 1908	0.722			
Linderone	0.126 ± 0.011	44355 ± 3716	1.242			
Linezolid	0.102 ± 0.005	13711 ± 160	0.382	0.118 ± 0.010	24736 ± 5143	0.757
Linoleamide	0.110 ± 0.004	35793 ± 772	1.002			
Linoleic acid	0.114 ± 0.004	41113 ± 2593	1.151			
Linoleoyl alanine	0.124 ± 0.007	38980 ± 862	1.091			
Linoleoyl dopamine	0.128 ± 0.005	28417 ± 2159	0.796			
Linoleoyl ethanolamide	0.125 ± 0.007	41480 ± 1592	1.161			
Linoleoyl GABA	0.132 ± 0.006	35886 ± 918	1.005			
Linoleoyl glycine	0.127 ± 0.004	30525 ± 1118	0.855			

Linomide	0.111 ± 0.004	27749 ± 2570	0.777			
Linopirdine	0.098 ± 0.009	39646 ± 6532	1.110			
Linopirdine dihydrochloride	0.107 ± 0.011	30897 ± 5033	0.865			
Liothyronine	0.100 ± 0.016	38513 ± 3218	1.078			
liothyronine (l-isomer)-sodium	0.120 ± 0.003	18299 ± 796	0.527			
Lipiferolide	0.124 ± 0.003	19561 ± 660	0.547			
Lipoamide	0.111 ± 0.009	29958 ± 440	0.839			
Lipoxin A4	0.111 ± 0.002	38004 ± 782	1.064			
Liranaftate	0.108 ± 0.009	39023 ± 3793	1.093			
Liriodendrin	0.123 ± 0.006	38196 ± 2760	1.069			
Liriodenine	0.119 ± 0.012	33714 ± 3404	0.944			
Lisinopril	0.123 ± 0.002	32717 ± 613	0.916			
Lisofylline	0.106 ± 0.007	35356 ± 5994	0.990			
Lithium Chloride	0.102 ± 0.004	41574 ± 2609	1.164			
Lithium citrate	0.107 ± 0.002	28620 ± 2848	0.801			
Lithocholic acid	0.110 ± 0.004	50265 ± 2671	1.407			
Litseglutine B	0.126 ± 0.005	31007 ± 2801	0.868			
L-Kynurenine sulfate	0.135 ± 0.004 *					
L-Leucinethiol, oxidized dihydrochloride	0.109 ± 0.010	31573 ± 1129	0.884			
L-leucyl-l-alanine	0.105 ± 0.003	36119 ± 1895	1.011			
L-Methionine sulfoximine	0.104 ± 0.005	35371 ± 1840	0.990			
L-N6-(1-Iminoethyl)-lysine hydrochloride	0.101 ± 0.004	35225 ± 2671	0.986			
L-NAME	0.110 ± 0.002	28009 ± 2689	0.784			
L-NAME hydrochloride	0.101 ± 0.003	30369 ± 3161	0.850			
L-NASPA	0.113 ± 0.008	47386 ± 4890	1.327			
L-NIL hydrochloride	0.121 ± 0.006	25900 ± 901	0.725			
L-NMMA	0.108 ± 0.010	44589 ± 853	1.248			
Lobaric acid	0.101 ± 0.002	34539 ± 5162	0.967			
Lobeline	0.108 ± 0.002	48833 ± 6682	1.367			
Lobeline hydrochloride	0.114 ± 0.004	48737 ± 8133	1.365			
Lobendazole	0.109 ± 0.011	19835 ± 1864	0.571			
Lochnerine	0.121 ± 0.001	40892 ± 4144	1.145			

Locustatachykinin Ⅰ	0.109 ±0.008	18200 ±553	0.510			
Lofepramine	0.116 ±0.006	33646 ±2924	0.942			
Lofexidine	0.120 ±0.004	31997 ±2255	0.896			
Lofexidine hydrochloride	0.103 ±0.011	28373 ±3264	0.794			
Loganetin	0.119 ±0.007	37725 ±4129	1.056			
Loganic acid	0.114 ±0.013	30482 ±1817	0.853			
Loganin	0.128 ±0.003	27531 ±1848	0.771			
Lomefloxacin hydrochloride	0.068 ±0.004 **					
Lomerizine hydrochloride	0.116 ±0.008	32338 ±1008	0.905			
Lomifylline	0.124 ±0.004	22230 ±2077	0.622			
Lomofungin	0.106 ±0.005	62775 ±3041	1.785			
Lomustine	0.113 ±0.006	25810 ±887	0.723			
Longifloroside A	0.121 ±0.002	32339 ±2087	0.905			
Longikaurin E	0.121 ±0.006	18687 ±1530	0.522			
Longistylin C	0.122 ±0.003	42332 ±3935	1.185			
Longistylumphylline A	0.125 ±0.003	39810 ±5084	1.115			
Lonidamine	0.102 ±0.003	27740 ±850	0.777			
Lonomycin Calciym Salt	0.106 ±0.002	27894 ±1867	0.781			
Loperamide	0.113 ±0.004	34784 ±2012	0.974			
Loperamide hydrochloride	0.107 ±0.007	32043 ±1013	0.897			
Lophanthoidin B	0.124 ±0.003	27420 ±2050	0.768			
Lophanthoidin E	0.121 ±0.003	29264 ±2116	0.819			
Lophanthoidin F	0.118 ±0.004	42906 ±3097	1.201			
Loracarbef	0.064 ±0.013 **					
Loratadine	0.101 ±0.009	32813 ±1197	0.919			
Loratidine	0.098 ±0.008	38893 ±4429	1.089			
Lorazepam	0.120 ±0.003	68416 ±5065	1.905			
Loreclezole hydrochloride	0.112 ±0.004	38798 ±2528	1.086			
Lorglumide	0.115 ±0.007	7602 ±346	0.216	0.119 ±0.006	30908 ±3708	0.946
Lorglumide sodium	0.096 ±0.011	18832 ±3487	0.521			
L-Ornithine, N5-[imino(methylamino)-methyl]	0.111 ±0.004	23586 ±2674	0.660			
Lornoxicam	0.100 ±0.008	29344 ±2619	0.822			

Losartan	0.124 ±0.023	38642 ±1731	1.082			
Losartan Potassium	0.124 ±0.008	22158 ±1904	0.617			
Loteprednol etabonate	0.126 ±0.004	42909 ±4423	1.201			
Lovastatin	0.118 ±0.006	43489 ±4319	1.218			
Loxapine succinate	0.104 ±0.004	30284 ±1400	0.848			
Loxoprofen	0.099 ±0.006	29320 ±1688	0.821			
Loxoprofen sodium	0.126 ±0.005	34853 ±1676	0.976			
LP44	0.103 ±0.015	53932 ±1298	1.491			
L-p-Bromotetramisole oxalate	0.123 ±0.006	26739 ±1155	0.749			
L-phenylalaninol	0.109 ±0.004	34960 ±734	0.979			
L-Quisqualic acid	0.114 ±0.003	30048 ±2452	0.841			
L-Thio AP4	0.107 ±0.006	44509 ±2896	1.246			
L-thyroxine [(3-[4-(4-hydroxy-3,5-diiodophenoxy)-3,5-diiodophenyl]-l-alanine]	0.126 ±0.014	44513 ±1765	1.246			
L-trans-Pyrrolidine-2,4-dicarboxylic acid	0.102 ±0.012	35081 ±2253	0.982			
L-Tryptophan	0.111 ±0.007	28406 ±2430	0.795			
Lucidone	0.123 ±0.006	35858 ±588	1.004			
Luciferase Inhibitor I	0.100 ±0.002	54293 ±2378	1.588			
Luciferase Inhibitor II	0.086 ±0.002 **					
Lufenuron	0.109 ±0.006	34334 ±3091	0.961			
Lumefantrine	0.104 ±0.010	37143 ±4687	1.040			
Luminol	0.103 ±0.004	46230 ±8064	1.294			
Lumiracoxib	0.100 ±0.007	24936 ±3628	0.698			
Lunarine	0.099 ±0.005	39372 ±3225	1.102			
Lupalbigenin	0.120 ±0.002	24365 ±460	0.682			
Lupanine	0.123 ±0.002	34333 ±789	0.961			
Lupanine perchlorate	0.114 ±0.012	39197 ±3131	1.097			
Lupanyl acid hydrochloride	0.105 ±0.008	40101 ±1387	1.123			
Lupenone	0.125 ±0.006	25027 ±949	0.701			
Lupeol	0.113 ±0.011	41848 ±1846	1.172			
Lupeol acetate	0.106 ±0.004	37322 ±788	1.045			
Lupeol caffeate	0.131 ±0.005	41962 ±3455	1.175			
Lupinine	0.137 ±0.003 *					

Lutein	0.129 ± 0.002	32996 ± 1133	0.924			
Luteinzing Hormone Releasing Hormone (LHRH)	0.113 ± 0.002	21001 ± 186	0.588			
Luteolin	0.105 ± 0.004	44306 ± 1487	1.241			
Luteolin 7-glucoside	0.118 ± 0.006	42542 ± 1323	1.191			
Luteolin-3',7-di-o-glucoside	0.097 ± 0.008	21033 ± 1091	0.625			
Luteone	0.129 ± 0.002	35824 ± 2533	1.003			
Luzindole	0.106 ± 0.007	55463 ± 6311	1.553			
LXRα/β Agonist	0.101 ± 0.003	28001 ± 2012	0.784			
LY 163502	0.109 ± 0.004	21993 ± 3058	0.616			
LY 165163	0.109 ± 0.009	40860 ± 7316	1.144			
LY 171883	0.109 ± 0.012	28931 ± 2128	0.810			
LY 2183240	0.099 ± 0.007	24810 ± 1946	0.695			
LY 225910	0.117 ± 0.004	31969 ± 5476	0.895			
LY 231617	0.102 ± 0.002	140596 ± 5170	4.113	0.112 ± 0.004	44287 ± 4399	1.355
LY 235959	0.113 ± 0.007	33486 ± 8597	0.938			
LY 2365109 hydrochloride	0.112 ± 0.009	42222 ± 2856	1.182			
LY 255283	0.108 ± 0.003	29389 ± 3474	0.823			
LY 288513	0.119 ± 0.006	32075 ± 3164	0.898			
LY 290181	0.106 ± 0.010	47071 ± 4392	1.318			
LY 294002	0.126 ± 0.006	33642 ± 2030	0.942			
LY 294002 hydrochloride	0.115 ± 0.004	36641 ± 2305	1.026			
LY 294002, 4'-NH_2	0.122 ± 0.005	36725 ± 869	1.028			
LY 295427	0.122 ± 0.009	45454 ± 2317	1.273			
LY 303511	0.114 ± 0.008	28485 ± 1503	0.798			
LY 310762 hydrochloride	0.108 ± 0.004	48920 ± 2081	1.370			
LY 320135	0.117 ± 0.005	33841 ± 1285	0.948			
LY 344864 hydrochloride	0.108 ± 0.005	22870 ± 1367	0.640			
LY 354740	0.102 ± 0.005	30495 ± 1711	0.854			
LY 364947	0.106 ± 0.009	33671 ± 3046	0.943			
LY 367265	0.110 ± 0.010	39488 ± 1501	1.106			
LY 367385	0.104 ± 0.003	30603 ± 3955	0.857			
LY 379268	0.119 ± 0.002	56492 ± 777	1.582			

LY 393558	0.111 ± 0.014	46073 ± 5774	1.290			
LY 456236	0.124 ± 0.005	67365 ± 4995	1.886			
LY 83583	0.097 ± 0.001	13171 ± 843	0.400	0.098 ± 0.008	22461 ± 1382	0.687
Lycernuic acid A	0.120 ± 0.003	39901 ± 2212	1.117			
Lyclaninol	0.117 ± 0.005	39714 ± 2433	1.112			
Lycoclavanol	0.129 ± 0.005	39090 ± 4222	1.094			
Lycorine	0.111 ± 0.007	18890 ± 559	0.529			
Lycorine hydrochloride	0.119 ± 0.010	19078 ± 1962	0.567			
Lymecycline	0.115 ± 0.006	37696 ± 1034	1.055			
Lynestrenol	0.122 ± 0.003	49779 ± 4139	1.394			
Lyoniresinol	0.125 ± 0.005	37422 ± 4561	1.048			
Lyoniside	0.130 ± 0.002	34780 ± 3485	0.974			
Lysergol	0.110 ± 0.002	132447 ± 1477	3.934	0.120 ± 0.009	34524 ± 1589	1.056
Lysine (L)-hydrochloride	0.105 ± 0.008	32611 ± 1635	0.913			
Lyso-PAF C16	0.111 ± 0.002	22785 ± 497	0.638			
Lyso-PAF C18 *	0.105 ± 0.003	20100 ± 611	0.563			
Lysophosphatidic acid	0.108 ± 0.001	36124 ± 1802	1.011			
L-α-Methyl DOPA	0.106 ± 0.001	40031 ± 1958	1.121			
L-α-Methyl-p-tyrosine	0.102 ± 0.002	29204 ± 843	0.818			
L-β-threo-benzyl-aspartate	0.112 ± 0.005	34429 ± 2718	0.964			
M-344	0.111 ± 0.004	27509 ± 1019	0.770			
M-3M3FBS	0.104 ± 0.004	42401 ± 1084	1.187			
M-6434	0.101 ± 0.005	65936 ± 2467	1.763			
Maclurin	0.119 ± 0.001	33889 ± 2418	0.949			
Macrocarpal A	0.120 ± 0.004	37952 ± 1021	1.063			
Macrocarpal B	0.099 ± 0.015	37958 ± 935	1.063			
Macrocarpal C	0.108 ± 0.007	24681 ± 3739	0.691			
Macrocarpal D	0.120 ± 0.007	33283 ± 2015	0.932			
Macrocarpal E	0.112 ± 0.004	31811 ± 1909	0.891			
Macrocarpal H	0.115 ± 0.002	32613 ± 1121	0.913			
Macrocarpal I	0.120 ± 0.001	29497 ± 740	0.826			
Macrocarpal J	0.123 ± 0.008	34796 ± 2638	0.974			

Macrocarpal K	0.116 ± 0.002	54519 ± 3745	1.527			
Macrocarpal L	0.121 ± 0.001	38628 ± 913	1.082			
Macrocarpal N	0.117 ± 0.006	25799 ± 916	0.722			
Madecassic acid	0.125 ± 0.007	36715 ± 2601	1.028			
Mafenide	0.105 ± 0.001	34653 ± 4386	0.970			
Mafenide hydrochloride	0.127 ± 0.008	53210 ± 9100	1.490			
Maglifloenone	0.126 ± 0.009	47287 ± 3617	1.324			
Magnoflorine	0.121 ± 0.002	48756 ± 3529	1.365			
Magnolin	0.127 ± 0.016	40643 ± 4100	1.138			
Magnolol	0.107 ± 0.018	96232 ± 14110	2.858	0.107 ± 0.012	55211 ± 6221	1.689
Magnoloside A	0.123 ± 0.003	36076 ± 2112	1.010			
Malaben	0.114 ± 0.005	32647 ± 2898	0.914			
Malathion	0.098 ± 0.005	19103 ± 2020	0.550			
Malic acid 4-Me ester	0.109 ± 0.010	37622 ± 5608	1.053			
Mallorepine	0.127 ± 0.004	40392 ± 4653	1.131			
Maltol	0.110 ± 0.006	34461 ± 1826	0.965			
Mandelic acid, methyl ester	0.106 ± 0.003	39381 ± 2739	1.103			
Mangafodipir trisodium	0.109 ± 0.012	24060 ± 4085	0.674			
Mangiferin	0.129 ± 0.005	36782 ± 3575	1.030			
Mangochinine	0.118 ± 0.002	42447 ± 967	1.188			
Mangostanol	0.123 ± 0.007	41081 ± 3460	1.150			
Mangostin trimethyl ether	0.108 ± 0.003	37675 ± 3077	1.055			
Manidipine	0.084 ± 0.009 **					
Manidipine hydrochloride	0.077 ± 0.005 **					
Mannioside A	0.127 ± 0.003	36770 ± 756	1.030			
Mannitol	0.102 ± 0.004	33114 ± 1698	0.927			
Manoalide	0.109 ± 0.002	31161 ± 2621	0.872			
Manool	0.111 ± 0.002	27863 ± 754	0.780			
Manumycin A, Streptomyces parvulus	0.095 ± 0.008	6383 ± 432	0.187	0.108 ± 0.010	19943 ± 3186	0.610
Maoecrystal B	0.128 ± 0.004	37871 ± 2045	1.060			
MAPP, D-erythro	0.102 ± 0.001	40726 ± 3628	1.140			
MAPP, L-erythro	0.107 ± 0.002	33378 ± 2001	0.935			

Maprotiline hydrochloride	0.102 ±0.011	23792 ±1943	0.666			
Marein	0.099 ±0.004	56634 ±1475	1.682			
Marimastat	0.096 ±0.006	38069 ±1020	1.066			
Maritimein	0.111 ±0.003	22791 ±383	0.638			
Marmin	0.128 ±0.006	40314 ±4028	1.129			
Marmin acetonide	0.124 ±0.022	32257 ±816	0.903			
Marsdenoside F	0.128 ±0.005	41319 ±1268	1.157			
Martynoside	0.128 ±0.008	26838 ±906	0.751			
Masatinib	0.125 ±0.001	26075 ±1568	0.730			
Massonianoside B	0.119 ±0.007	38279 ±1821	1.072			
Massoniresinol	0.112 ±0.004	32345 ±1530	0.906			
Masticadienolic acid	0.114 ±0.002	38206 ±3210	1.070			
Mastoparan	0.054 ±0.003 **					
Matairesinol	0.110 ±0.013	39508 ±3885	1.106			
Matricarin	0.101 ±0.003	36384 ±3341	1.019			
Matrine	0.124 ±0.005	39684 ±2950	1.111			
Mayumbine	0.132 ±0.003	37275 ±4203	1.044			
MBCQ	0.104 ±0.005	33006 ±1684	0.924			
MC-1293	0.108 ±0.011	40254 ±1578	1.127			
M-chlorophenylbiguanide	0.104 ±0.007	27501 ±3351	0.770			
M-chlorophenylbiguanide hydrochloride	0.107 ±0.008	30336 ±3882	0.849			
MCI-186	0.114 ±0.005	36077 ±3981	1.010			
McN A-343	0.106 ±0.005	28434 ±4739	0.796			
MDL 105519	0.101 ±0.010	34200 ±3375	0.958			
MDL 11939	0.112 ±0.007	34523 ±3747	0.967			
MDL 26630 trihydrochloride	0.109 ±0.005	35869 ±732	1.004			
MDL 28170	0.095 ±0.008	32262 ±927	0.903			
MDL 28170(Z-VF-CHO)	0.128 ±0.003	28075 ±1022	0.786			
MDL 29951	0.106 ±0.005	29152 ±5603	0.816			
MDL 72222	0.102 ±0.003	22456 ±1784	0.629			
MDL 72832 hydrochloride	0.111 ±0.004	38082 ±6917	1.066			
MDL 73005EF hydrochloride	0.109 ±0.006	30276 ±5644	0.848			

MDM2 Antagonist, Nutlin-3, Racemic	0.094 ± 0.001 *					
Me-3,4-dephostatin	0.109 ± 0.001	29470 ± 3694	0.825			
Mead acid (20:3 n-9)	0.107 ± 0.003	31507 ± 2424	0.882			
Mead ethanolamide	0.112 ± 0.001	41291 ± 1490	1.156			
Mearnsitrin	0.120 ± 0.006	42289 ± 1182	1.184			
Mebendazol	0.109 ± 0.014	31197 ± 2386	0.873			
Mebendazole	0.137 ± 0.006 *					
Mebeverine hydrochloride	0.121 ± 0.003	35493 ± 1074	0.994			
Mebhydrolin naphthalenesulfonate	0.109 ± 0.018	5086 ± 112	0.147	0.114 ± 0.016	17090 ± 2852	0.523
Mebhydroline 1,5-naphtalenedisulfonate	0.112 ± 0.004	40169 ± 2063	1.125			
Mebicar	0.109 ± 0.003	49110 ± 2854	1.375			
Mecamylamine hydrochloride	0.102 ± 0.008	52089 ± 6120	1.458			
Mecarbinate	0.122 ± 0.008	18285 ± 2650	0.512			
Mechlorethamine	0.103 ± 0.004	41491 ± 4939	1.162			
Mecillinam	0.111 ± 0.007	35342 ± 3704	0.990			
Meclizine hydrochloride	0.076 ± 0.001 **					
Meclocycline sulfosalicylate	0.120 ± 0.004	47613 ± 1555	1.333			
Meclofenamate sodium	0.115 ± 0.003	22451 ± 2507	0.629			
Meclofenoxate hydrochloride	0.106 ± 0.004	42173 ± 2593	1.181			
Mecloqualone	0.112 ± 0.013	38797 ± 615	1.086			
Meclozine dihydrochloride	0.071 ± 0.006 **					
Mecysteine hydrochloride	0.105 ± 0.002	50784 ± 4596	1.422			
Medetomidine hydrochloride	0.105 ± 0.002	34454 ± 2880	0.965			
Medicarpin	0.110 ± 0.012	40394 ± 1669	1.131			
Medioresinol	0.108 ± 0.007	44082 ± 1509	1.234			
Medroxyprogesone acetate	0.111 ± 0.007	33272 ± 4293	0.932			
Medroxyprogesterone	0.134 ± 0.002 *					
Medroxyprogesterone 17-acetate	0.104 ± 0.007	31589 ± 1471	0.884			
Medroxyprogesterone acetate	0.102 ± 0.015	31010 ± 3560	0.868			
Medrysone	0.124 ± 0.007	46722 ± 1506	1.308			
Mefenamic acid	0.107 ± 0.003	42668 ± 2031	1.195			
Mefexamide hydrochloride	0.118 ± 0.004	46839 ± 1682	1.311			

Mefloquine	0.111 ± 0.005	18070 ± 1162	0.521			
Mefloquine hydrochloride	0.136 ± 0.025	21861 ± 1400	0.597			
Megastigm-7-ene-3,5,6,9-tetraol	0.120 ± 0.004	46328 ± 2197	1.297			
Megestrol acetate	0.119 ± 0.009	67647 ± 4843	1.884			
Meglumine	0.115 ± 0.008	37405 ± 2549	1.047			
Meglutol	0.102 ± 0.003	47344 ± 3536	1.326			
MEK Inhibitor I	0.115 ± 0.004	61822 ± 7621	1.831			
MEK Inhibitor II	0.077 ± 0.003 **					
MEK1/2 Inhibitor	0.105 ± 0.002	62414 ± 3620	1.848			
MEK1/2 Inhibitor II	0.118 ± 0.010	36629 ± 4515	1.026			
Melatonin	0.110 ± 0.012	45566 ± 1359	1.276			
Melengestrol acetate	0.106 ± 0.005	37943 ± 8046	1.062			
Melezitose	0.101 ± 0.005	38241 ± 4958	1.071			
Meliasenin B	0.118 ± 0.001	44267 ± 4326	1.239			
Melilotigenin B	0.120 ± 0.002	57870 ± 5632	1.620			
Melilotigenin C	0.125 ± 0.001	47959 ± 3909	1.343			
Meloscandonine	0.118 ± 0.004	35861 ± 1129	1.004			
Meloxicam	0.107 ± 0.004	34166 ± 1197	0.957			
Meloxicam sodium	0.102 ± 0.016	31486 ± 3158	0.882			
Melperone hydrochloride	0.120 ± 0.003	30289 ± 6827	0.848			
Melphalan	0.105 ± 0.005	30698 ± 2163	0.860			
Memantine hydrochloride	0.113 ± 0.001	37669 ± 2534	1.055			
Menadione	0.103 ± 0.002	44624 ± 2188	1.249			
Menaquinone-4	0.104 ± 0.003	44051 ± 2619	1.233			
Menthone	0.110 ± 0.001	34335 ± 2981	0.961			
Menthyl benzoate	0.102 ± 0.003	19438 ± 6904	0.544			
Meparfylon	0.117 ± 0.004	33249 ± 3767	0.931			
Mepartricin	0.102 ± 0.003	25577 ± 1215	0.716			
Mepenzolate bromide	0.122 ± 0.006	34137 ± 1635	0.956			
Mephenesin	0.137 ± 0.010	39035 ± 3958	1.093			
Mephentermine hemisulfate	0.108 ± 0.010	49233 ± 7708	1.378			
Mephentermine sulfate	0.113 ± 0.008	29322 ± 3996	0.821			

Mephenytoin	0.112 ± 0.007	42908 ± 2912	1.201			
Mephetyl tetrazole	0.095 ± 0.003	35178 ± 3680	0.985			
Mepirizole	0.104 ± 0.004	32594 ± 4200	0.913			
Mepiroxol	0.112 ± 0.003	32235 ± 4638	0.903			
Mepivacaine hydrochloride	0.117 ± 0.003	61174 ± 4532	1.703			
Meprylcaine hydrochloride	0.102 ± 0.007	35721 ± 2506	1.000			
Meptazinol hydrochloride	0.136 ± 0.012	45577 ± 4062	1.276			
Mepyramine maleate	0.106 ± 0.005	29814 ± 3186	0.835			
Mequinol	0.099 ± 0.008	27852 ± 1895	0.780			
Mequitazine	0.111 ± 0.001	44277 ± 1552	1.240			
Meranzin	0.125 ± 0.002	42546 ± 2573	1.191			
Meranzin hydrate	0.131 ± 0.004	27908 ± 1418	0.781			
Merbromin	0.057 ± 0.002 **					
Mercaptopurine	0.126 ± 0.005	51196 ± 5959	1.433			
Merck -5	0.138 ± 0.017	42071 ± 3762	1.178			
Meridinol	0.122 ± 0.002	31822 ± 3500	0.891			
Merogedunin	0.121 ± 0.002	32191 ± 2094	0.901			
Meropenem	0.113 ± 0.003	23342 ± 3908	0.654			
Mesalamine	0.113 ± 0.004	39079 ± 1747	1.094			
Mesna	0.108 ± 0.005	42841 ± 3582	1.200			
Mesoridazine	0.118 ± 0.003	33446 ± 1288	0.936			
Mesoridazine besylate	0.114 ± 0.005	20075 ± 1002	0.562			
Mestanolone	0.118 ± 0.008	37563 ± 3071	1.052			
Mestranol	0.121 ± 0.003	48949 ± 4865	1.371			
Mesuaxanthone A	0.116 ± 0.006	39342 ± 4715	1.102			
Mesulergine hydrochloride	0.116 ± 0.006	29862 ± 2723	0.836			
Met Kinase Inhibitor	0.120 ± 0.005	31359 ± 2664	0.878			
Metacetamol	0.111 ± 0.005	35187 ± 2739	0.985			
Meta-cresyl acetate	0.114 ± 0.016	30514 ± 1265	0.854			
Metameconine	0.103 ± 0.014	24754 ± 3165	0.693			
Metaphit	0.110 ± 0.004	34348 ± 1230	0.962			
Metaproterenol	0.103 ± 0.006	42156 ± 5208	1.180			

Metaproterenol hemisulfate	0.100 ±0.018	20095 ±3778	0.555			
Metaproterenol sulfate, orciprenaline sulfate	0.118 ±0.006	53972 ±2341	1.473			
Metaraminol bitartrate	0.122 ±0.005	43184 ±4291	1.209			
Metaxalone	0.110 ±0.009	30681 ±4432	0.859			
Metergoline	0.100 ±0.012	25467 ±4496	0.713			
Metergoline phenylmethyl ester	0.111 ±0.009	20050 ±1184	0.536			
Metformin	0.112 ±0.002	45882 ±3437	1.285			
Metformin hydrochloride	0.116 ±0.009	28723 ±1109	0.804			
Methacholine chloride	0.117 ±0.003	51141 ±6122	1.432			
Methacycline hydrochloride	0.124 ±0.004	39321 ±1910	1.101			
Methanesulfonamide, N-[4-[[1-[2-(6-methyl-2-pyridinyl) -ethyl]-4-piperidinyl]carbonyl]phenyl]-, dihydrochloride	0.111 ±0.004	43236 ±2218	1.211			
Methantheline bromide	0.120 ±0.003	53798 ±5107	1.468			
Methapyrilene hydrochloride	0.103 ±0.012	31000 ±1935	0.868			
Methazolamide	0.111 ±0.001	46283 ±4207	1.296			
Methenamine	0.118 ±0.006	40247 ±3117	1.127			
Methiazole	0.111 ±0.011	43559 ±4995	1.220			
Methicillin sodium	0.112 ±0.001	18398 ±3154	0.530			
Methimazole	0.103 ±0.003	28358 ±2232	0.794			
Methionine sulfoximine (L)	0.103 ±0.005	33130 ±2049	0.928			
Methiothepin maleate	0.107 ±0.005	23303 ±1615	0.652			
Methiothepin mesylate	0.107 ±0.008	32710 ±1060	0.916			
Methocarbamol	0.118 ±0.002	53986 ±2062	1.473			
Methoctramine quadrahydrochloride	0.108 ±0.010	41513 ±3566	1.162			
Methoprene (S)	0.109 ±0.005	40455 ±2647	1.133			
Methoprene acid	0.119 ±0.004	43414 ±2787	1.216			
Methotrexate	0.110 ±0.003	31720 ±1695	0.888			
Methotrexate hydrate	0.106 ±0.001	44408 ±2671	1.243			
Methotrimeprazine maleat salt	0.113 ±0.004	31787 ±2352	0.890			
Methoxamine hydrochloride	0.112 ±0.009	32101 ±2101	0.899			
Methoxsalen	0.111 ±0.006	20944 ±747	0.603			
Methoxy verapamil	0.138 ±0.019	44292 ±1138	1.240			

Methoxyamine hydrochloride	0.114 ± 0.009	31373 ± 3154	0.878			
Methoxydimethyltryptamine	0.113 ± 0.003	33474 ± 903	0.937			
Methoxyflavone, 5-	0.106 ± 0.026	17733 ± 1376	0.527			
Methoxyvone	0.103 ± 0.005	33053 ± 4995	0.925			
Methscopolamine bromide	0.108 ± 0.002	40165 ± 2570	1.125			
Methsuximide	0.104 ± 0.005	40883 ± 1817	1.145			
Methyclothiazide	0.109 ± 0.002	28075 ± 2690	0.786			
Methyl 2-(5-acetyl-2,3-dihydrobenzofuran-2-yl)-propenoate	0.112 ± 0.001	8647 ± 1592	0.242	0.106 ± 0.012	24576 ± 2810	0.752
Methyl 2-(6-acetyl-5-hydroxy-2,3-dihydrobenzofuran-2-yl)-propenoate	0.116 ± 0.002	27994 ± 3925	0.784			
Methyl 2,5-dihydroxycinnamate	0.120 ± 0.003	27326 ± 2009	0.765			
Methyl 3-(2,4-dihydroxyphenyl)-propionate	0.116 ± 0.005	42332 ± 3019	1.185			
Methyl 3-carbazolecarboxylate	0.123 ± 0.004	51194 ± 2234	1.433			
Methyl 3-hydroxy-4,5-dimethoxybenzoate	0.121 ± 0.003	41410 ± 1587	1.159			
Methyl 3-indolecarboxylate	0.126 ± 0.011	36601 ± 1114	1.025			
Methyl 4-hydroxybenzoate	0.134 ± 0.001	36215 ± 1907	1.014			
Methyl 6-acetoxyangolensate	0.118 ± 0.005	39847 ± 1792	1.116			
Methyl 6-hydroxyangolensate	0.115 ± 0.003	38812 ± 1062	1.087			
Methyl 7-deshydroxypyrogallin-4-carboxylate	0.106 ± 0.004	28637 ± 1612	0.802			
Methyl benzethonium chloride	0.052 ± 0.001 **					
Methyl chanofruticosinate	0.114 ± 0.006	27646 ± 1872	0.774			
Methyl demethoxycarbonylchanofruticosinate	0.129 ± 0.014	36947 ± 2075	1.034			
Methyl deoxycholate	0.110 ± 0.008	50385 ± 6041	1.411			
Methyl dodonate A	0.125 ± 0.001	35409 ± 2998	0.991			
Methyl eichlerianate	0.114 ± 0.010	40794 ± 3119	1.142			
Methyl gambogate methyl ether	0.110 ± 0.006	41368 ± 3207	1.158			
Methyl isocostate	0.101 ± 0.008	36656 ± 1690	1.026			
Methyl isodrimeninol	0.118 ± 0.004	45977 ± 2985	1.287			
Methyl levulinate	0.090 ± 0.002 **					
Methyl lycernuate A	0.131 ± 0.002	32368 ± 2408	0.906			
Methyl mandelate glucoside	0.127 ± 0.001	45794 ± 5665	1.282			
Methyl orsellinate	0.120 ± 0.001	46102 ± 2671	1.291			
Methyl pseudolarate A	0.117 ± 0.002	27153 ± 1569	0.760			

Methyl robustone	0.111 ±0.009	46099 ±2759	1.291			
Methyl rosmarinate	0.097 ±0.013	28262 ±6321	0.791			
Methyl salicylate	0.122 ±0.005	26645 ±1746	0.746			
Methyl sinapate	0.126 ±0.000	33501 ±266	0.938			
Methyl syringate	0.130 ±0.016	34562 ±1830	0.968			
Methyl trimethoxycinnamate	0.102 ±0.007	41825 ±2143	1.171			
Methyl β-D-fructofuranoside	0.115 ±0.003	57609 ±567	1.610			
Methyl-6,7-dimethoxy-4-ethyl-β-carboline-3-carboxylate	0.104 ±0.006	32121 ±1446	0.899			
Methylanabasine	0.104 ±0.008	25903 ±2996	0.725			
Methylandrostenediol	0.115 ±0.005	39114 ±1379	1.095			
Methylatropine nitrate	0.106 ±0.003	34605 ±6644	0.969			
Methylbenzethonium chloride	0.050 ±0.007 **					
Methyldopa	0.108 ±0.004	37031 ±5712	1.037			
Methyldopa (L,-)	0.106 ±0.003	55769 ±4676	1.522			
Methyldopate hydrochloride	0.111 ±0.019	31117 ±1450	0.871			
Methylene adenosine 5'-triphosphate dilithium	0.100 ±0.005	40779 ±1912	1.142			
Methylene blue	0.084 ±0.004 **					
Methylergometrine maleate	0.110 ±0.006	31944 ±335	0.894			
Methylergonovine	0.103 ±0.003	23345 ±389	0.654			
Methylergonovine maleate	0.101 ±0.007	25357 ±2162	0.710			
Methylhydantoin-5-(D)	0.107 ±0.005	44051 ±1585	1.233			
Methylhydantoin-5-(L)	0.106 ±0.003	55515 ±3132	1.515			
Methylisopelletierine	0.116 ±0.006	30520 ±3289	0.855			
Methyllinderone	0.118 ±0.003	40505 ±1378	1.134			
Methyllucidone	0.120 ±0.003	47092 ±1047	1.319			
Methyllycaconitine citrate	0.100 ±0.007	38696 ±1260	1.083			
Methylphenidate hydrochloride	0.102 ±0.003	33869 ±3544	0.948			
Methylphenobarbital	0.103 ±0.014	26293 ±3905	0.736			
Methylprednisolone	0.124 ±0.011	39973 ±1827	1.119			
Methylprednisolone acetate	0.117 ±0.006	53323 ±2180	1.570			
Methylprednisolone sodium succinate	0.112 ±0.008	36075 ±596	1.010			
Methylprednisolone, 6-α	0.127 ±0.012	45568 ±3540	1.276			

Methylpseudolarate B	0.111 ± 0.003	25904 ± 923	0.725			
Methyltestosterone	0.119 ± 0.009	24127 ± 3237	0.676			
Methylthiouracil	0.125 ± 0.008	18947 ± 2136	0.546			
Methyl-β-carboline-3-carboxylate	0.104 ± 0.001	52346 ± 1526	1.466			
Methysergide	0.116 ± 0.005	24997 ± 2201	0.700			
Methysergide maleate	0.105 ± 0.015	38906 ± 3637	1.089			
Methysticin	0.129 ± 0.002 *					
Metiapril	0.120 ± 0.008	33314 ± 2805	0.933			
Meticrane	0.123 ± 0.007	39738 ± 789	1.113			
Metitepine maleate	0.111 ± 0.010	18353 ± 1146	0.529			
Metixene hydrochloride	0.107 ± 0.003	25499 ± 932	0.714			
Metoclopramide hydrochloride	0.112 ± 0.009	29081 ± 1387	0.814			
Metolazone	0.095 ± 0.014	48407 ± 1127	1.338			
Metoprolol tartrate	0.106 ± 0.005	38816 ± 4962	1.087			
Metrazoline oxalate	0.102 ± 0.007	40535 ± 2339	1.135			
Metrifudil	0.100 ± 0.009	66914 ± 2539	1.850			
Metrizamide	0.111 ± 0.007	49038 ± 4665	1.373			
Metronidazole	0.106 ± 0.002	35332 ± 3095	0.989			
Metronidazole Benzoate	0.107 ± 0.021	35947 ± 2473	1.006			
Metylperon	0.124 ± 0.002	45202 ± 3730	1.266			
Metyrapone	0.110 ± 0.003	72109 ± 4198	1.968			
Mevalonic acid lactone	0.104 ± 0.006	42609 ± 7231	1.193			
Mevalonic-D, L acid lactone	0.117 ± 0.003	50926 ± 3428	1.426			
Mevastatin	0.103 ± 0.007	25399 ± 426	0.711			
Mevastatin, Sodium Salt	0.100 ± 0.011	40269 ± 1757	1.128			
Mevinolin (Lovastatin)	0.110 ± 0.004	23691 ± 1825	0.663			
Mexamine	0.100 ± 0.004	43741 ± 4605	1.225			
Mexeneone	0.118 ± 0.008	29789 ± 2644	0.834			
Mexicanolide	0.107 ± 0.005	34911 ± 1702	0.977			
Mexiletene hydrochloride	0.102 ± 0.010	31007 ± 1196	0.868			
Mexiletine hydrochloride	0.111 ± 0.006	36858 ± 3415	1.032			
Mezerein	0.064 ± 0.012 **					

MG-132	0.114 ± 0.015	29377 ± 1346	0.823			
MG-624	0.101 ± 0.006	22844 ± 919	0.640			
MHPG piperazine	0.095 ± 0.004	26096 ± 2368	0.731			
MHPG sulfate potassium	0.099 ± 0.009	44096 ± 5168	1.235			
Mianserin hydrochloride	0.105 ± 0.006	27153 ± 228	0.760			
Mibefradil dihydrochloride	0.104 ± 0.007	38355 ± 226	1.074			
Miconazole	0.105 ± 0.006	49501 ± 2816	1.386			
Miconazole nitrate	0.053 ± 0.011 **					
Micranoic acid A	0.127 ± 0.005	31471 ± 1976	0.881			
Micromelin	0.128 ± 0.010	48439 ± 1644	1.356			
Midodrine hydrochloride	0.118 ± 0.006	49501 ± 2851	1.386			
MIF Antagonist, ISO-1	0.095 ± 0.011	34258 ± 1655	0.959			
Mifepristone	0.125 ± 0.004	41382 ± 2588	1.159			
Miglitol	0.117 ± 0.002	29809 ± 597	0.835			
Miglustat	0.111 ± 0.004	30246 ± 4351	0.847			
Milnacipran	0.114 ± 0.006	50770 ± 5301	1.422			
Milnacipran hydrochloride	0.100 ± 0.001	43775 ± 1060	1.226			
Milrinone	0.111 ± 0.010	46630 ± 6842	1.306			
Miltefosine	0.108 ± 0.004	62299 ± 3028	1.772			
Mimosine	0.108 ± 0.011	30066 ± 1989	0.842			
Mimosine, L-	0.101 ± 0.009	40320 ± 6233	1.129			
Minaprine dihydrochloride	0.113 ± 0.008	49394 ± 2838	1.383			
Minecoside	0.117 ± 0.006	39227 ± 1129	1.098			
Minocycline	0.065 ± 0.003 **					
Minocycline hydrochloride	0.060 ± 0.004 **					
Minoxidil	0.108 ± 0.009	33096 ± 2422	0.927			
Minoxidil sulfate	0.110 ± 0.002	30312 ± 2045	0.849			
Minumicrolin	0.125 ± 0.005	30700 ± 1460	0.860			
m-Iodobenzylguanidine hemisulfate	0.107 ± 0.003	33717 ± 3065	0.944			
MIRA-1	0.093 ± 0.007 *					
Mirtazapine	0.106 ± 0.004	31178 ± 2974	0.873			
Mirtazepine	0.104 ± 0.012	35380 ± 2585	0.991			

Misoprostol	0.107 ± 0.003	39540 ± 4474	1.107			
Misoprostol, free acid	0.116 ± 0.001	28066 ± 2250	0.786			
Mithramycin A	0.081 ± 0.009 **					
Mitiglinide	0.103 ± 0.005	34374 ± 3817	0.962			
Mitochondrial Division Inhibitor, mdivi-1	0.102 ± 0.006	29191 ± 1633	0.817			
Mitomycin	0.061 ± 0.007 **					
Mitomycin C	0.077 ± 0.004 **					
Mitotane	0.120 ± 0.008	26382 ± 1764	0.739			
Mitoxantrone	0.116 ± 0.007	26702 ± 918	0.748			
Mitoxantrone dihydrochloride	0.119 ± 0.002	1208 ± 161	0.034	0.121 ± 0.006	5068 ± 531	0.155
Mitraphylline	0.107 ± 0.005	27862 ± 681	0.780			
Mizolastine	0.109 ± 0.004	35865 ± 707	1.004			
Mizoribine	0.101 ± 0.014	25850 ± 861	0.724			
MK 212 hydrochloride	0.107 ± 0.006	29075 ± 2549	0.814			
MK-2 Inhibitor III	0.110 ± 0.021	44080 ± 2914	1.234			
MK2a Inhibitor	0.111 ± 0.015	56653 ± 3909	1.678			
MK-886	0.117 ± 0.008	29122 ± 674	0.815			
MK-912	0.104 ± 0.009	63368 ± 4597	1.752			
ML-10302	0.115 ± 0.006	29878 ± 2275	0.837			
ML-7	0.121 ± 0.003	29142 ± 2169	0.816			
ML-7, Hydrochloride	0.118 ± 0.029	32987 ± 1795	0.924			
ML-9	0.122 ± 0.005	34185 ± 1352	0.957			
ML-9 Hydrochloride	0.115 ± 0.003	13343 ± 530	0.369	0.115 ± 0.004	25931 ± 1706	0.794
MM-77 dihydrochloride	0.119 ± 0.004	41030 ± 7445	1.149			
M-Methoxyphenol	0.108 ± 0.005	30691 ± 3028	0.859			
Mmpip	0.110 ± 0.002	33858 ± 1680	0.948			
Mmpx	0.106 ± 0.005	39109 ± 2625	1.095			
Mn-cpx 3	0.105 ± 0.009	39822 ± 552	1.115			
M-Nifedipine	0.117 ± 0.003	33935 ± 3643	0.950			
Mnitmt	0.110 ± 0.010	28790 ± 1891	0.806			
MNK1 Inhibitor	0.107 ± 0.002	23843 ± 3956	0.668			
MNS	0.104 ± 0.009	29245 ± 3096	0.819			

MnTBAP	0.113 ±0.001	30188 ±2111	0.845			
Moclobemide	0.099 ±0.005	31842 ±2475	0.892			
Modafinil	0.127 ±0.012	35212 ±3557	0.986			
Modaline sulfate	0.098 ±0.016	38417 ±2367	1.076			
Moexipril hydrochloride	0.107 ±0.010	34334 ±2607	0.961			
Moguisteine	0.114 ±0.017	27092 ±2622	0.759			
Molindone hydrochloride	0.106 ±0.007	28166 ±3919	0.789			
Mollugin	0.114 ±0.004	36505 ±887	1.022			
Molsidomine	0.104 ±0.011	28044 ±3794	0.785			
Moluccanin	0.136 ±0.004	30150 ±1080	0.844			
Moluccanin diacetate	0.136 ±0.001	25206 ±2085	0.706			
Mometasone Furoate	0.103 ±0.012	43389 ±2645	1.215			
Momor-cerebroside I	0.133 ±0.021	37834 ±3162	1.059			
Momordicoside G	0.122 ±0.003	39907 ±1537	1.117			
Momordicoside I aglycone	0.115 ±0.001	41312 ±5013	1.157			
Monastrol	0.111 ±0.002	29651 ±2544	0.830			
Monensin	0.111 ±0.004	29051 ±430	0.813			
Monensin Na	0.129 ±0.030	97301 ±5119	2.890	0.126 ±0.011	50811 ±974	1.555
Monobenzone	0.113 ±0.004	71787 ±12981	1.959			
Monocrotaline	0.109 ±0.003	42224 ±3147	1.182			
Monomethyl kolavate	0.136 ±0.010	31383 ±619	0.879			
Montelukast	0.105 ±0.011	42044 ±6488	1.177			
Montelukast sodium	0.106 ±0.006	30223 ±2149	0.846			
Morachalcone A	0.112 ±0.004	50867 ±4917	1.424			
Moracin C	0.103 ±0.002	34393 ±2784	0.963			
Moracin D	0.117 ±0.002	32535 ±3412	0.911			
Morantel citrate	0.100 ±0.001	27686 ±725	0.775			
Morantel tartrate	0.118 ±0.003	26434 ±2381	0.740			
Moricizine hydrochloride	0.108 ±0.003	27059 ±1493	0.758			
Morin	0.100 ±0.009	68715 ±2626	1.899			
Moronic acid	0.113 ±0.007	42873 ±3355	1.200			
Moroxidine hydrochloride	0.115 ±0.002	43793 ±1931	1.226			

Morphiceptin	0.098 ± 0.010	34956 ± 7595	0.979			
Morusin	0.120 ± 0.001	38719 ± 1656	1.084			
Morusinol	0.137 ± 0.004	31141 ± 2363	0.872			
Mosapride citrate	0.126 ± 0.002	67701 ± 8781	1.896			
Moslosooflavone	0.111 ± 0.007	41976 ± 4708	1.175			
Motesanib	0.125 ± 0.005	6725 ± 470	0.200	0.114 ± 0.007	7990 ± 519	0.244
Moxalactam disodium	0.123 ± 0.002	40304 ± 5016	1.128			
Moxidectin	0.108 ± 0.002	44423 ± 5791	1.244			
Moxifloxacin	0.074 ± 0.012 **					
Moxifloxacin hydrochloride	0.067 ± 0.005 **					
Moxisylyte hydrochoride	0.114 ± 0.008	51294 ± 2463	1.436			
Moxonidine	0.114 ± 0.007	41335 ± 1226	1.157			
Moxonidine hydrochloride	0.120 ± 0.001	37602 ± 2775	1.053			
MP-470	0.126 ± 0.009	33981 ± 236	0.951			
MPEP hydrochloride	0.120 ± 0.002	62643 ± 4467	1.754			
MPMQ hydrochloride	0.106 ± 0.010	37423 ± 2458	1.048			
MR 16728 hydrochloride	0.106 ± 0.011	53676 ± 3587	1.503			
MRS 1220	0.104 ± 0.013	34466 ± 1734	0.965			
MRS 1334	0.121 ± 0.005	38458 ± 763	1.077			
MRS 1523	0.102 ± 0.010	38779 ± 1957	1.086			
MRS 1845	0.097 ± 0.009	42180 ± 1568	1.181			
MRS 2159	0.107 ± 0.013	29788 ± 1113	0.834			
MRS 2179	0.103 ± 0.010	62667 ± 8476	1.732			
MRS 3777 hemioxalate	0.119 ± 0.003	40385 ± 5260	1.131			
MTEP hydrochloride	0.113 ± 0.003	45800 ± 1605	1.282			
MTPG	0.106 ± 0.001	27485 ± 3744	0.770			
Mucic acid	0.090 ± 0.002 **					
Mucrolidin	0.123 ± 0.007	27470 ± 264	0.769			
Mulberrin	0.102 ± 0.005	43631 ± 3338	1.222			
Mullilam diol	0.126 ± 0.004	48408 ± 3647	1.355			
Mundoserone	0.106 ± 0.006	41871 ± 4745	1.172			
Mundulone	0.119 ± 0.005	29848 ± 2042	0.836			

Mupirocin	0.051 ± 0.006 **					
Muristerone A, Ipomoea spp.	0.098 ± 0.006	41186 ± 3490	1.153			
Murralongin	0.112 ± 0.009	37450 ± 1994	1.049			
Murrangatin	0.106 ± 0.007	31325 ± 2063	0.877			
Murrangatin diacetate	0.127 ± 0.009	31735 ± 1502	0.889			
Murraol	0.129 ± 0.003	40253 ± 2044	1.127			
Murraxocin	0.125 ± 0.003	37787 ± 1960	1.058			
Muscarine Cl, (+)-	0.103 ± 0.003	23114 ± 248	0.647			
Muscimol	0.108 ± 0.003	30455 ± 1495	0.853			
Muscimol hydrobromide	0.100 ± 0.003	33885 ± 3785	0.949			
Mussaenoside	0.127 ± 0.004	30327 ± 1333	0.849			
muurolladie-3-one	0.113 ± 0.012	32990 ± 2525	0.924			
MY-5445	0.112 ± 0.003	27585 ± 611	0.772			
Myclobutanil	0.101 ± 0.004	54299 ± 4958	1.520			
Mycophenolate mofetil	0.122 ± 0.015	53551 ± 1756	1.499			
Mycophenolic acid	0.090 ± 0.004 *					
Myoseverin	0.121 ± 0.014	25282 ± 1276	0.708			
Myosmine	0.120 ± 0.011	27723 ± 501	0.776			
Myricadiol	0.120 ± 0.019	34077 ± 1376	0.954			
Myricananin A	0.118 ± 0.008	49288 ± 4649	1.380			
Myricanol	0.121 ± 0.014	43283 ± 9437	1.212			
Myricanol triacetate	0.119 ± 0.002	40619 ± 2785	1.137			
Myricanone	0.131 ± 0.004	34984 ± 2325	0.980			
Myriceric acid B	0.117 ± 0.006	50616 ± 2351	1.417			
Myriceric acid C	0.116 ± 0.002	44459 ± 3369	1.245			
Myricetin	0.105 ± 0.013	68740 ± 10498	1.900			
Myricitrin	0.102 ± 0.008	26346 ± 295	0.738			
Myriocin	0.110 ± 0.006	29853 ± 809	0.836			
Myristicin	0.118 ± 0.003	31234 ± 982	0.875			
N α-Methylhistamine dihydrochloride	0.106 ± 0.004	30848 ± 312	0.864			
N-(2-[4-(4-Chlorophenyl)-piperazin-1-yl]ethyl)-3-methoxybenzamide	0.108 ± 0.007	42844 ± 6863	1.200			
N-(2-Hydroxy-4-methoxyphenyl)-acetamide	0.116 ± 0.011	44973 ± 6774	1.259			

N-(3,3-Diphenylpropyl)-glycinamide	0.113 ± 0.003	42543 ± 4902	1.191			
N-(3-Aminopropyl)-1,10-decanediamine trihydrochloride	0.113 ± 0.010	34164 ± 5597	0.957			
N-(4-Amino-2-chlorophenyl)-phthalimide	0.105 ± 0.005	46357 ± 2618	1.298			
N-(4-Bromobenzyl)-5-methoxytryptamine oxalate	0.097 ± 0.009	30981 ± 1602	0.867			
N-(N-Benzylpiperidin-4-YL)-4-iodobenzamide	0.110 ± 0.004	42647 ± 2809	1.194			
N,N,N',N'-Tetramethylazodicarboxamide	0.100 ± 0.006	35270 ± 899	0.988			
N,N,N-trimethyl-1-(4-trans-stilbenoxy)-2-propylammonium iodide	0.113 ± 0.006	28397 ± 2267	0.795			
N,N'-Diacetyl-1,6-diaminohexane	0.112 ± 0.006	36688 ± 1348	1.027			
N,N-Diethyl-2-[4-(phenylmethyl)-phenoxy]ethanamine	0.096 ± 0.010	18107 ± 3898	0.507			
N,N-Dihexyl-2-(4-fluorophenyl)-indole-3-acetamide	0.101 ± 0.003	32441 ± 1709	0.908			
N,N-Dimethylsphingosine	0.106 ± 0.004	37836 ± 2738	1.059			
N,N-Dipropyl-5-carboxamidotryptamine maleate	0.101 ± 0.003	31821 ± 667	0.891			
N-[2-(Acetoxy)-ethyl]-3-pyridinecarboxamide	0.115 ± 0.004	29302 ± 1387	0.820			
N-[2-(Piperidinylamino)-ethyl]-4-iodobenzamide	0.105 ± 0.003	42702 ± 2269	1.196			
N^G,N^G-Dimethylarginine hydrochloride	0.102 ± 0.009	39832 ± 4417	1.115			
N1,N10-Bis(p-coumaroyl)-spermidine	0.118 ± 0.006	32995 ± 3573	0.924			
N1-Methoxymethyl picrinine	0.124 ± 0.019	30646 ± 1090	0.858			
N20C Hydrochloride	0.110 ± 0.004	27863 ± 3739	0.780			
N6-(4-Aminobenzyl)-N-methylcarboxamidoadenosine	0.104 ± 0.008	50264 ± 6352	1.407			
N6-2-(4-Aminophenyl)-ethyladenosine	0.106 ± 0.003	36672 ± 1550	1.027			
N6-Cyclohexyladenosine	0.100 ± 0.009	31688 ± 965	0.887			
N6-Cyclopentyladenosine	0.109 ± 0.006	45049 ± 2385	1.261			
N6-Methyl-2'-deoxy-adenosine	0.104 ± 0.010	60953 ± 7943	1.707			
N6-Methyladenosine	0.101 ± 0.006	40873 ± 2742	1.144			
N6-Methyladenosine-5'-monophosphate Na	0.100 ± 0.005	25399 ± 220	0.711			
N6-Phenyladenosine	0.095 ± 0.014	36393 ± 1763	1.019			
N6-P-Sulfophenyladenosine Na	0.108 ± 0.005	37564 ± 2982	1.052			
N9-Isopropylolomoucine	0.113 ± 0.002	30180 ± 1367	0.845			
Na+/H+ Exchanger Isoform-1 Inhibitor	0.106 ± 0.022	34230 ± 2843	0.958			
NAADP Receptor Modulator	0.109 ± 0.020	36645 ± 4614	1.026			
Nabumetone	0.107 ± 0.004	52049 ± 504	1.457			
N-Ac-Cysteine	0.118 ± 0.005	38926 ± 1824	1.090			

N-Acetyl-5-hydroxytryptamine	0.102 ± 0.002	34866 ± 1026	0.976			
N-Acetyl-DL-homocysteine Thiolactone	0.109 ± 0.001	33919 ± 736	0.950			
N-Acetylglycyl-D-glutamic acid	0.109 ± 0.008	21148 ± 2536	0.592			
N-Acetyl-L-Cysteine	0.104 ± 0.001	46484 ± 2099	1.302			
N-Acetyl-leukotriene E4	0.110 ± 0.003	29355 ± 2090	0.822			
N-Acetyl-L-leucine	0.107 ± 0.005	44785 ± 3428	1.254			
N-Acetylprocainamide hydrochloride	0.098 ± 0.004	35059 ± 3344	0.982			
N-acetylproline	0.107 ± 0.003	32729 ± 1217	0.916			
N-Acetyltryptamine	0.101 ± 0.011	54633 ± 2568	1.510			
NADA	0.109 ± 0.003	25849 ± 1571	0.724			
Nadide	0.110 ± 0.006	45285 ± 3651	1.268			
Nadifloxacin	0.062 ± 0.001 **					
Nadolol	0.110 ± 0.007	33756 ± 3628	0.945			
NADPH tetrasodium	0.107 ± 0.012	19792 ± 1065	0.547			
Nafadotride	0.124 ± 0.003	51504 ± 3937	1.442			
Nafamostat mesylate	0.091 ± 0.012 *					
Nafamostst mesylate	0.111 ± 0.004	31684 ± 2156	0.887			
Nafcillin sodium	0.115 ± 0.001	20941 ± 1031	0.603			
Nafronyl oxalate	0.105 ± 0.007	42793 ± 1805	1.198			
Naftifine hydrochloride	0.112 ± 0.003	21062 ± 487	0.575			
Naftopidil	0.123 ± 0.004	62194 ± 7142	1.732			
Naftopidil dihydrochloride	0.104 ± 0.007	25171 ± 3373	0.705			
Nalbuphine	0.112 ± 0.002	37617 ± 6408	1.053			
Nalbuphine hydrochloride	0.108 ± 0.002	52192 ± 2131	1.424			
Nalidixic acid	0.080 ± 0.002 **					
Nalmefene hydrochloride	0.125 ± 0.018	43716 ± 4355	1.224			
Naloxonazine dihydrochloride	0.102 ± 0.012	29841 ± 821	0.836			
Naloxone benzoylhydrazone	0.101 ± 0.004	28471 ± 1607	0.797			
Naloxone hydrochloride	0.105 ± 0.007	36170 ± 2146	1.013			
Naloxone methiodide	0.105 ± 0.007	49388 ± 2991	1.383			
Naltrexone hydrochloride	0.108 ± 0.001	37570 ± 1494	1.052			
Naltrexone hydrochloride dihydrate	0.115 ± 0.005	58604 ± 3061	1.599			

Naltriben mesylate	0.105 ± 0.005	42925 ± 1201	1.202			
Naltriben methanesulfonate	0.102 ± 0.011	33591 ± 1596	0.941			
Naltriben methanesulfonate hydrate	0.117 ± 0.008	50308 ± 3544	1.409			
Naltrindole	0.107 ± 0.007	29894 ± 3483	0.837			
Naltrindole hydrochloride	0.104 ± 0.007	34784 ± 2389	0.974			
NAN-190	0.104 ± 0.006	31756 ± 3759	0.889			
NAN-190 hydrobromide	0.108 ± 0.006	59656 ± 2064	1.649			
Nandrolone	0.127 ± 0.010	36020 ± 225	1.009			
Naphazoline hydrochloride	0.114 ± 0.004	37134 ± 4909	1.040			
Naphthylin	0.092 ± 0.009	38829 ± 4011	1.087			
Naproxen	0.103 ± 0.000	39494 ± 2303	1.106			
Naproxen sodium	0.118 ± 0.008	47727 ± 3856	1.336			
Naproxol	0.106 ± 0.003	26782 ± 2382	0.750			
Napsul-Ile-Trp-CHO	0.124 ± 0.002	30312 ± 920	0.849			
Na-p-Tosyl-L-lysine chloromethyl ketone hydrochloride	0.111 ± 0.013	33567 ± 831	0.940			
N-Arachidonoylglycine	0.106 ± 0.009	32066 ± 932	0.898			
Narasin	0.099 ± 0.001 *					
Naringenin	0.094 ± 0.004 *					
Naringenin triacetate	0.122 ± 0.001	43030 ± 1928	1.205			
Naringenin trimethyl ether	0.125 ± 0.001	40473 ± 1715	1.133			
Naringenin-4',7-diacetate	0.123 ± 0.009	36893 ± 2385	1.033			
Naringin	0.123 ± 0.009	48515 ± 3568	1.358			
Narirutin	0.109 ± 0.004	23141 ± 742	0.648			
Natamycin	0.106 ± 0.004	37520 ± 1241	1.051			
Nateglinide	0.124 ± 0.006	62871 ± 5365	1.751			
N-Benzylnaltrindole hydrochloride	0.063 ± 0.004 **					
NBI 27914	0.105 ± 0.010	48729 ± 4221	1.364			
NBI 27914 hydrochloride	0.106 ± 0.009	32404 ± 3573	0.907			
NBQX	0.107 ± 0.005	39567 ± 3100	1.108			
NBQX disodium salt	0.104 ± 0.005	26734 ± 1467	0.749			
N-Bromoacetamide	0.101 ± 0.003	47146 ± 5564	1.320			
NCH-51	0.108 ± 0.005	30842 ± 885	0.864			

N-Chlorophenyl-N-hexylpiperazine	0.100 ± 0.008	35727 ± 6481	1.000			
NCS-356	0.087 ± 0.003 *					
NCS-382	0.088 ± 0.008 *					
N-Demethylechitamine	0.136 ± 0.005	21678 ± 2004	0.607			
N-Desisopropylpropranolol hydrochloride	0.105 ± 0.008	55245 ± 3718	1.547			
N-Desmethylclozapine	0.113 ± 0.007	28874 ± 1499	0.808			
NDGA	0.089 ± 0.003 **					
Nebivolol Hydrochloride	0.100 ± 0.024	53474 ± 4077	1.497			
NECA	0.095 ± 0.005 *					
Necrostatin-1	0.132 ± 0.021	52498 ± 1455	1.555			
Nedaplatin	0.115 ± 0.001	30779 ± 280	0.862			
Nefazodone	0.128 ± 0.006	89649 ± 6486	2.496	0.119 ± 0.006	38467 ± 2482	1.177
Nefazodone hydrochloride	0.105 ± 0.009	24369 ± 4628	0.682			
Nefiracetam	0.105 ± 0.002	37906 ± 2961	1.061			
Nefopam	0.104 ± 0.005	31290 ± 4486	0.876			
Nefopam hydrochloride	0.118 ± 0.014	51220 ± 4670	1.434			
Nelarabin	0.099 ± 0.013	48260 ± 5678	1.351			
Nelfinavir mesylate	0.135 ± 0.005 *					
Nemadipine-A	0.094 ± 0.011	31719 ± 716	0.888			
Nemonapride	0.111 ± 0.009	34970 ± 2842	0.979			
Nemoralisin	0.124 ± 0.005	47940 ± 1164	1.342			
Neoandrographolide	0.122 ± 0.011	35086 ± 2256	0.982			
Neobyakangelicol	0.131 ± 0.007	30938 ± 1806	0.866			
Neochlorogenic acid	0.128 ± 0.005	41218 ± 2107	1.154			
Neoechinulin A	0.111 ± 0.003	33027 ± 1999	0.925			
Neohesperidin	0.107 ± 0.001	18570 ± 562	0.552			
Neohesperidin dihydrochalcone	0.109 ± 0.009	30588 ± 1977	0.856			
Neomycin sulfate	0.102 ± 0.004	19671 ± 712	0.584			
Neoprzewaquinone A	0.125 ± 0.003	30991 ± 3083	0.868			
Neosophoramine	0.118 ± 0.009	36100 ± 1889	1.011			
Neostenine	0.123 ± 0.003	28544 ± 2388	0.799			
Neostigmine bromide	0.106 ± 0.011	43907 ± 2771	1.229			

Neostigmine Mesilate	0.116 ±0.003	49165 ±452	1.377			
Neotuberostemonine	0.130 ±0.005	32822 ±2123	0.919			
Neotuberostemonone	0.116 ±0.005	52972 ±2800	1.483			
Neridienone B	0.119 ±0.005	37494 ±3243	1.050			
Neritaloside	0.117 ±0.001	50748 ±3011	1.421			
Nerol	0.109 ±0.003	30309 ±457	0.849			
Nerolidol	0.123 ±0.003	36671 ±706	1.027			
Nervogenic acid	0.118 ±0.003	37491 ±3063	1.050			
N-Ethylmaleimide	0.103 ±0.003	44400 ±3748	1.243			
Netilmicin sulfate	0.104 ±0.009	28716 ±3016	0.804			
Neurodazine	0.094 ±0.004 *					
Neurokinin A	0.111 ±0.005	46604 ±2518	1.305			
Neuronal Differentiation Inducer III	0.103 ±0.009	46700 ±1983	1.308			
Neuropathiazol	0.097 ±0.003	42845 ±4638	1.200			
Neuropeptide S (Mouse)	0.109 ±0.001	26216 ±2326	0.734			
Neuropeptide Y 13-36(porcine)	0.110 ±0.004	30939 ±1440	0.866			
Nevirapine	0.107 ±0.005	26510 ±1904	0.742			
NF 023	0.100 ±0.010	38442 ±2784	1.076			
NF 279	0.111 ±0.005	52522 ±4511	1.471			
NFAT Activation Inhibitor III	0.104 ±0.006	57676 ±5328	1.687			
N-Feruloyloctopamine	0.130 ±0.004	31269 ±437	0.876			
NF-kB Activation Inhibitor	0.077 ±0.005 **					
NF-κB Activation Inhibitor II, JSH-23	0.115 ±0.010	41628 ±4696	1.166			
NF-κB Activation Inhibitor III	0.086 ±0.006 **					
NF-κB Activation Inhibitor IV	0.110 ±0.011	43832 ±1846	1.227			
NGB 2904	0.115 ±0.006	23648 ±1183	0.662			
NG-Monomethyl-L-arginine acetate	0.100 ±0.007	36239 ±3282	1.015			
NG-Nitro-L-arginine	0.100 ±0.005	38319 ±1064	1.073			
NG-Nitro-L-arginine methyl ester hydrochloride	0.106 ±0.005	38246 ±4164	1.071			
NH 125	0.121 ±0.014	42653 ±3253	1.194			
Niacin	0.112 ±0.003	58861 ±7445	1.606			
Niacinamide	0.107 ±0.001	40101 ±7695	1.123			

Nialamide	0.105 ±0.005	34988 ±3001	0.980			
Nicardipine	0.085 ±0.002 **					
Nicardipine hydrochloride	0.105 ±0.006	90088 ±7904	2.490	0.121 ±0.003	63185 ±4961	1.906
Nicergoline	0.128 ±0.008	11833 ±1427	0.316	0.119 ±0.014	46276 ±2468	1.416
Niclosamide	0.105 ±0.011	51597 ±8980	1.445			
Nicopholine	0.109 ±0.008	29884 ±2772	0.837			
Nicorandil	0.109 ±0.002	25651 ±3718	0.718			
Nicotiflorin	0.133 ±0.012	37353 ±1413	1.046			
Nicotinamide	0.122 ±0.002	23307 ±251	0.653			
Nicotine bitartrate	0.103 ±0.006	28089 ±2492	0.786			
Nicotine, (-)-	0.106 ±0.002	42032 ±1240	1.177			
Nicotinic Acid	0.107 ±0.003	23390 ±973	0.655			
Nicotinyl alcohol tartrate	0.105 ±0.007	28336 ±268	0.793			
Nifedipine	0.089 ±0.002 **					
Nifekalant hydrochloride	0.111 ±0.015	32204 ±4405	0.902			
Nifenazone	0.122 ±0.005	31484 ±2502	0.882			
Niflumic acid	0.102 ±0.002	33796 ±682	0.946			
Nifuroxazide	0.098 ±0.004	43499 ±3279	1.218			
Nifursol	0.095 ±0.013	27000 ±1644	0.756			
Nifurtimox	0.119 ±0.005	28321 ±2868	0.793			
Nigericin	0.106 ±0.003	27365 ±684	0.766			
Nigericin, Sodium Salt, Streptomyces hygroscopicus	0.099 ±0.003	33299 ±2568	0.932			
Nigracin	0.123 ±0.004	37589 ±2716	1.052			
Nigrolineaxanthone V	0.136 ±0.009	29120 ±2518	0.815			
Niguldipine	0.055 ±0.002 **					
Niguldipine hydrochloride	0.066 ±0.006 **					
Nikethamide	0.109 ±0.022	21075 ±4403	0.590			
Niloticin	0.110 ±0.006	33535 ±1448	0.939			
Nilotinib	0.129 ±0.003	28975 ±2950	0.811			
Nilutamide	0.112 ±0.007	115491 ±6887	3.192	0.116 ±0.005	31089 ±1513	0.951
Nilvadipine	0.112 ±0.003	29661 ±2035	0.830			
Nimbin	0.127 ±0.003	29847 ±1548	0.836			

Nimesulide	0.103 ± 0.010	70444 ± 4806	2.223	0.118 ± 0.005	63907 ± 5219	1.945
Nimetazepam	0.119 ± 0.003	41367 ± 9523	1.158			
Nimodipine	0.111 ± 0.001	28312 ± 2887	0.793			
Nimustine	0.128 ± 0.008	25054 ± 240	0.701			
Nimustine hydrochloride	0.103 ± 0.007	28572 ± 1486	0.800			
NIP-200	0.117 ± 0.002	39280 ± 3017	1.100			
Nipecotic acid	0.106 ± 0.004	25371 ± 1850	0.710			
Niranthin	0.126 ± 0.005	40201 ± 3030	1.126			
Niridazole	0.108 ± 0.006	51194 ± 5290	1.433			
Nisoldipine	0.118 ± 0.008	54612 ± 6852	1.529			
Nisoxetine hydrochloride	0.127 ± 0.012	30988 ± 5366	0.868			
Nitarsone	0.106 ± 0.006	25521 ± 3062	0.715			
Nitazoxanide	0.131 ± 0.003 *					
Nithiamide	0.114 ± 0.014	29439 ± 1102	0.824			
Nitidanin	0.129 ± 0.011	49442 ± 4992	1.384			
Nitrarine dihydrochloride	0.100 ± 0.002	4145 ± 60	0.123	0.114 ± 0.005	29205 ± 344	0.894
Nitrazepam	0.125 ± 0.003	65635 ± 4638	1.828			
Nitrendipine	0.109 ± 0.002	28778 ± 1250	0.806			
Nitrocaramiphen hydrochloride	0.101 ± 0.004	32792 ± 5083	0.918			
Nitrofural	0.116 ± 0.002	34249 ± 1068	0.959			
Nitrofurantoin	0.112 ± 0.006	22318 ± 1883	0.625			
Nitrofurazone	0.114 ± 0.010	21418 ± 839	0.600			
Nitroglycerin	0.108 ± 0.001	32185 ± 1259	0.901			
Nitromide	0.111 ± 0.003	19757 ± 2859	0.553			
Nitroxoline	0.057 ± 0.001 **					
Nizatidine	0.122 ± 0.004	41798 ± 3834	1.170			
NKY80	0.095 ± 0.002	24831 ± 1230	0.695			
N-Linoleoylglycine	0.103 ± 0.004	35643 ± 2034	0.998			
NMDA	0.105 ± 0.005	46906 ± 1360	1.313			
N-methyl (-)-ephedrine [1r,2s]	0.107 ± 0.006	31237 ± 457	0.875			
N-Methyl-1-deoxynojirimycin	0.102 ± 0.012	30971 ± 532	0.867			
N-methylanthranilic acid	0.097 ± 0.009	37073 ± 1209	1.038			

n-methylbenzylamine hydrochloride	0.109 ± 0.006	34276 ± 2418	0.960			
N-Methylcalycinine	0.113 ± 0.003	24389 ± 1065	0.683			
N-Methyl-D-aspartic acid	0.103 ± 0.020	34884 ± 4590	0.977			
N-Methyldopamine hydrochloride	0.113 ± 0.007	24509 ± 2363	0.686			
N-Methylhistaprodifen dioxalate salt	0.104 ± 0.004	14133 ± 198	0.391	0.107 ± 0.008	32669 ± 3506	1.000
N-methylisoleucine	0.113 ± 0.002	35320 ± 5047	0.989			
N-Methyllidocaine iodide	0.110 ± 0.001	39181 ± 1089	1.097			
N-Methyllindcarpine	0.136 ± 0.008	31892 ± 1524	0.893			
N-Methyl-N-[(1S)-1-phenyl-2-(1-pyrrolidinyl)-ethyl]phenylacetamide hydrochloride	0.104 ± 0.007	35297 ± 1079	0.988			
N-Methylquipazine dimaleate	0.100 ± 0.011	42109 ± 5003	1.179			
N-Methylsarpagine methosalt	0.130 ± 0.005	19850 ± 2703	0.556			
N-Methyl-β-carboline-3-carboxamide	0.089 ± 0.005 *					
N-MPPP Hydrochloride	0.113 ± 0.012	35771 ± 5016	1.002			
NNC 05-2090 hydrochloride	0.100 ± 0.006	18168 ± 2783	0.503			
NNC 55-0396	0.057 ± 0.005 **					
NNC 55-0396 dihydrochloride	0.067 ± 0.004 **					
NNC 63-0532	0.103 ± 0.009	28365 ± 2373	0.794			
NNC 711	0.110 ± 0.006	36202 ± 1147	1.014			
NNGH (BML-205)	0.100 ± 0.004 *					
N'-Nitrosonornicotine	0.108 ± 0.008	26552 ± 3788	0.743			
NO-711 hydrochloride	0.105 ± 0.009	50042 ± 1641	1.383			
Nobiletin	0.107 ± 0.005	32916 ± 1931	0.922			
Nociceptin	0.106 ± 0.005	25907 ± 4163	0.725			
Nociceptin (1-13)-NH_2	0.117 ± 0.007	37702 ± 1203	1.056			
Nocistatin	0.102 ± 0.004	50590 ± 1231	1.416			
Nocodazole	0.098 ± 0.008	32305 ± 5219	0.905			
N-Octyl caffeate	0.102 ± 0.005	70448 ± 5520	1.967			
Nodakenetin	0.116 ± 0.004	41680 ± 5652	1.167			
Nodosin	0.070 ± 0.006 **					
N-Oleoyldopamine	0.100 ± 0.009	24884 ± 4083	0.697			
N-Oleoylethanolamine	0.092 ± 0.002 *					

N-omega-Methyl-5-hydroxytryptamine oxalate salt	0.099 ± 0.005	34113 ± 4917	0.955			
Nomegestrol acetate	0.116 ± 0.013	30404 ± 2033	0.851			
Nomifensine maleate	0.104 ± 0.007	29036 ± 1086	0.813			
Nomilin	0.127 ± 0.003	44748 ± 2824	1.253			
Nonactin	0.102 ± 0.001	36221 ± 800	1.014			
Nonadecanoic acid	0.103 ± 0.003	38764 ± 8304	1.085			
Nonic acid	0.100 ± 0.003	32686 ± 1733	0.915			
Nonivamide	0.104 ± 0.006	34948 ± 2550	0.979			
Nonoxynol-9	0.104 ± 0.010	27274 ± 3807	0.764			
Nootkatone	0.132 ± 0.005	30202 ± 2789	0.846			
Norandrostenedione-19	0.120 ± 0.001	30380 ± 3496	0.851			
Norantiffein	0.112 ± 0.005	37163 ± 3203	1.041			
Norathyriol	0.109 ± 0.003	22321 ± 3646	0.625			
Nor-Binaltorphimine dihydrochloride	0.103 ± 0.008	30912 ± 2626	0.866			
Norbraylin	0.129 ± 0.008	36737 ± 2887	1.029			
Norcantharidin	0.104 ± 0.008	43193 ± 3740	1.209			
Norcepharadione B	0.117 ± 0.007	36552 ± 1327	1.023			
Norcyclobenzaprine	0.113 ± 0.001	41396 ± 2365	1.159			
Nordazepam	0.099 ± 0.009	52143 ± 7986	1.460			
Nordihydroguaiaretic acid from Larrea divaricata (creosote bush)	0.107 ± 0.012	120489 ± 5255	3.330	0.119 ± 0.012	53891 ± 3096	1.649
Noreleagnine	0.108 ± 0.003	18324 ± 260	0.544			
Norepinephrine	0.101 ± 0.003	36895 ± 2180	1.033			
Norepinephrine-(+)-tartrate l (-)	0.112 ± 0.006	43783 ± 2817	1.226			
Norethindrone	0.112 ± 0.002	39473 ± 3302	1.105			
Norethindrone acetate	0.109 ± 0.002	24602 ± 1692	0.689			
Norethynodrel	0.129 ± 0.006	43354 ± 303	1.214			
Noreugenin	0.124 ± 0.014	56811 ± 2716	1.588			
Norfloxacin	0.071 ± 0.009 **					
Norfluorocurarine	0.099 ± 0.008	23661 ± 1638	0.662			
Norgestimate	0.113 ± 0.005	36556 ± 1924	1.024			
Norgestrel	0.108 ± 0.006	30013 ± 3301	0.840			
Norgestrel-(-)-D	0.112 ± 0.002	61144 ± 5063	1.669			

Norharman	0.105 ± 0.009	18781 ± 585	0.541			
Norharmane	0.104 ± 0.005	19907 ± 440	0.591			
Norketamine hydrochloride	0.115 ± 0.004	31446 ± 2602	0.880			
Norkhellol	0.126 ± 0.008	41298 ± 2756	1.156			
Norlichexanthone	0.126 ± 0.021	32623 ± 4542	0.913			
Nornicotine	0.104 ± 0.002	38915 ± 2557	1.090			
Norstictic acid	0.102 ± 0.008	43309 ± 3533	1.213			
Nortetraphyllicine	0.127 ± 0.025	32501 ± 3161	0.910			
Nortrachelogenin	0.135 ± 0.005	28364 ± 1424	0.794			
Nortriptyline hydrochloride	0.112 ± 0.002	40749 ± 3579	1.141			
Norviburtinal	0.121 ± 0.006	27334 ± 980	0.765			
Noscapine hydrochloride	0.109 ± 0.005	35891 ± 2567	1.005			
Noscapine, (±)-	0.102 ± 0.003	22079 ± 549	0.618			
Novobiocin Na	0.057 ± 0.003 **					
NP-000002	0.115 ± 0.007	35922 ± 383	1.006			
NP-000003	0.123 ± 0.002	56517 ± 6247	1.582			
NP-000004	0.115 ± 0.002	53730 ± 8306	1.504			
NP-000008	0.119 ± 0.003	44556 ± 2589	1.248			
NP-000021	0.120 ± 0.002	35632 ± 1455	0.998			
NP-000023	0.122 ± 0.004	30435 ± 1779	0.852			
NP-000024	0.119 ± 0.006	36179 ± 1639	1.013			
NP-000029	0.115 ± 0.002	24528 ± 6902	0.687			
NP-000031	0.122 ± 0.006	36011 ± 2029	1.008			
NP-000032	0.124 ± 0.003	49585 ± 5873	1.388			
NP-000033	0.109 ± 0.001	43109 ± 6292	1.207			
NP-000037	0.120 ± 0.003	27118 ± 2651	0.759			
NP-000038	0.114 ± 0.006	41902 ± 2547	1.173			
NP-000041	0.118 ± 0.003	60314 ± 5770	1.615			
NP-000042	0.131 ± 0.005	46421 ± 884	1.300			
NP-000047	0.121 ± 0.004	27207 ± 787	0.762			
NP-000049	0.119 ± 0.007	41496 ± 3370	1.162			
NP-000053	0.117 ± 0.003	37694 ± 4305	1.055			

NP-000056	0.114 ±0.007	40316 ±4095	1.129			
NP-000057	0.125 ±0.001	46021 ±1952	1.289			
NP-000058	0.118 ±0.007	36009 ±1017	1.008			
NP-000060	0.130 ±0.006	46979 ±7275	1.315			
NP-000061	0.117 ±0.003	39888 ±3629	1.117			
NP-000062	0.106 ±0.003	41418 ±3242	1.160			
NP-000064	0.116 ±0.003	36133 ±1159	1.012			
NP-000065	0.111 ±0.004	35944 ±1422	1.006			
NP-000074	0.138 ±0.004	49824 ±2971	1.395			
NP-000084	0.118 ±0.010	28649 ±2687	0.802			
NP-000086	0.128 ±0.008	36598 ±1640	1.025			
NP-000087	0.115 ±0.002	35351 ±3909	0.990			
NP-000088	0.120 ±0.002	38373 ±2141	1.074			
NP-000090	0.113 ±0.002	25757 ±685	0.721			
NP-000091	0.117 ±0.011	39336 ±525	1.101			
NP-000092	0.123 ±0.008	31468 ±397	0.881			
NP-000093	0.119 ±0.003	34686 ±3338	0.971			
NP-000095	0.115 ±0.002	35857 ±2319	1.004			
NP-000097	0.116 ±0.004	49366 ±4979	1.382			
NP-000098	0.112 ±0.003	35192 ±1631	0.985			
NP-000099	0.112 ±0.009	46291 ±3046	1.296			
NP-000100	0.105 ±0.006	49578 ±2035	1.388			
NP-000104	0.127 ±0.007	40128 ±1117	1.124			
NP-000112	0.120 ±0.008	34581 ±8949	0.968			
NP-000114	0.124 ±0.005	29413 ±498	0.824			
NP-000116	0.112 ±0.007	38068 ±2769	1.066			
NP-000118	0.123 ±0.005	39792 ±2737	1.114			
NP-000120	0.114 ±0.005	49201 ±3427	1.378			
NP-000124	0.107 ±0.006	23644 ±2282	0.662			
NP-000129	0.118 ±0.006	49481 ±7518	1.385			
NP-000130	0.117 ±0.006	49343 ±4606	1.382			
NP-000135	0.124 ±0.014	52250 ±4931	1.463			

NP-000136	0.114 ±0.001	32925 ±733	0.922			
NP-000139	0.120 ±0.001	56164 ±5663	1.504			
NP-000142	0.118 ±0.002	43802 ±2643	1.226			
NP-000143	0.119 ±0.006	52175 ±4608	1.461			
NP-000145	0.105 ±0.004	43068 ±1355	1.206			
NP-000147	0.112 ±0.001	33843 ±2543	0.948			
NP-000150	0.121 ±0.006	35941 ±3460	1.006			
NP-000151	0.113 ±0.004	30585 ±4167	0.856			
NP-000157	0.113 ±0.003	24667 ±998	0.691			
NP-000158	0.113 ±0.009	35989 ±4188	1.008			
NP-000159	0.121 ±0.007	44295 ±3919	1.240			
NP-000167	0.111 ±0.004	42572 ±7178	1.192			
NP-000168	0.113 ±0.004	35066 ±2529	0.982			
NP-000172	0.115 ±0.003	40289 ±4367	1.128			
NP-000173	0.120 ±0.007	45047 ±6456	1.261			
NP-000176	0.112 ±0.002	50170 ±3109	1.405			
NP-000180	0.119 ±0.005	38512 ±1976	1.078			
NP-000181	0.122 ±0.008	42319 ±2868	1.185			
NP-000186	0.115 ±0.001	32503 ±4748	0.910			
NP-000187	0.116 ±0.003	54687 ±10598	1.531			
NP-000192	0.117 ±0.013	25000 ±3690	0.700			
NP-000200	0.113 ±0.004	36603 ±1177	1.025			
NP-000205	0.121 ±0.006	49062 ±891	1.374			
NP-000206	0.132 ±0.002	74555 ±9248	1.996			
NP-000211	0.126 ±0.005	53727 ±7051	1.504			
NP-000212	0.117 ±0.004	38778 ±3174	1.086			
NP-000213	0.117 ±0.003	40044 ±1956	1.121			
NP-000216	0.110 ±0.008	28825 ±311	0.807			
NP-000218	0.103 ±0.004	41954 ±3102	1.175			
NP-000223	0.112 ±0.009	41854 ±2127	1.172			
NP-000224	0.114 ±0.002	50646 ±1245	1.418			
NP-000226	0.112 ±0.006	41379 ±6529	1.159			

NP-000227	0.124 ±0.007	38675 ±799	1.083			
NP-000232	0.115 ±0.006	41167 ±2853	1.153			
NP-000233	0.122 ±0.009	40335 ±2061	1.129			
NP-000234	0.114 ±0.004	26067 ±5673	0.730			
NP-000239	0.112 ±0.003	28829 ±1463	0.807			
NP-000240	0.117 ±0.003	47968 ±1307	1.343			
NP-000244	0.116 ±0.004	33488 ±2227	0.938			
NP-000247	0.115 ±0.002	39161 ±3968	1.096			
NP-000248	0.125 ±0.006	31357 ±1252	0.878			
NP-000249	0.124 ±0.004	24174 ±449	0.677			
NP-000251	0.118 ±0.003	34310 ±6243	0.961			
NP-000252	0.119 ±0.006	39660 ±9335	1.110			
NP-000253	0.133 ±0.005	49451 ±3586	1.385			
NP-000255	0.116 ±0.009	45614 ±4336	1.277			
NP-000257	0.129 ±0.005	49540 ±2410	1.387			
NP-000258	0.113 ±0.003	38764 ±4089	1.085			
NP-000261	0.118 ±0.006	55396 ±2030	1.483			
NP-000262	0.124 ±0.003	40758 ±4253	1.141			
NP-000264	0.118 ±0.004	31906 ±1673	0.893			
NP-000271	0.112 ±0.006	33450 ±7322	0.937			
NP-000279	0.128 ±0.010	36043 ±7033	1.009			
NP-000282	0.116 ±0.006	42918 ±3796	1.202			
NP-000283	0.111 ±0.004	42140 ±798	1.180			
NP-000286	0.116 ±0.003	71269 ±12315	1.944			
NP-000287	0.115 ±0.003	40152 ±3067	1.124			
NP-000288	0.112 ±0.005	40941 ±3205	1.146			
NP-000291	0.116 ±0.003	45014 ±4982	1.260			
NP-000292	0.117 ±0.006	45947 ±1774	1.286			
NP-000294	0.125 ±0.006	45443 ±9896	1.272			
NP-000296	0.116 ±0.003	35615 ±5302	0.997			
NP-000298	0.125 ±0.001	39660 ±2355	1.110			
NP-000302	0.133 ±0.012	37217 ±2885	1.042			

NP-000303	0.109 ±0.014	44248 ±4643	1.239			
NP-000305	0.119 ±0.011	38663 ±4586	1.083			
NP-000309	0.135 ±0.013	39072 ±1464	1.094			
NP-000310	0.121 ±0.002	27307 ±1200	0.765			
NP-000313	0.123 ±0.003	37808 ±1766	1.059			
NP-000314	0.125 ±0.005	37031 ±2995	1.037			
NP-000315	0.121 ±0.009	34669 ±1750	0.971			
NP-000318	0.112 ±0.003	49276 ±2269	1.380			
NP-000319	0.123 ±0.017	23975 ±1603	0.671			
NP-000327	0.113 ±0.004	34041 ±3465	0.953			
NP-000330	0.110 ±0.006	42045 ±4184	1.177			
NP-000333	0.116 ±0.006	43144 ±2170	1.208			
NP-000334	0.119 ±0.003	32889 ±1478	0.921			
NP-000337	0.120 ±0.003	36862 ±3961	1.032			
NP-000341	0.115 ±0.004	55492 ±3745	1.486			
NP-000342	0.122 ±0.001	37799 ±2408	1.058			
NP-000343	0.113 ±0.001	19087 ±500	0.511			
NP-000347	0.114 ±0.008	35270 ±2687	0.988			
NP-000353	0.120 ±0.007	55302 ±3245	1.481			
NP-000357	0.118 ±0.010	31253 ±249	0.875			
NP-000358	0.119 ±0.005	31390 ±3093	0.879			
NP-000359	0.122 ±0.014	51501 ±6777	1.442			
NP-000360	0.116 ±0.002	56194 ±2684	1.573			
NP-000362	0.115 ±0.001	42459 ±500	1.189			
NP-000371	0.115 ±0.007	28941 ±3031	0.810			
NP-000373	0.113 ±0.001	96814 ±7043	2.592	0.105 ±0.007	34175 ±4689	1.046
NP-000374	0.113 ±0.008	59548 ±1257	1.594			
NP-000375	0.126 ±0.006	36364 ±2227	1.018			
NP-000376	0.117 ±0.003	32449 ±1634	0.909			
NP-000378	0.120 ±0.002	40401 ±903	1.131			
NP-000380	0.118 ±0.002	33710 ±2004	0.944			
NP-000381	0.110 ±0.008	32505 ±1344	0.910			

NP-000383	0.118 ±0.003	42673 ±1518	1.195			
NP-000386	0.124 ±0.002	43495 ±310	1.218			
NP-000387	0.128 ±0.004	50805 ±1796	1.423			
NP-000389	0.119 ±0.005	38718 ±847	1.084			
NP-000390	0.117 ±0.011	36421 ±1888	1.020			
NP-000391	0.117 ±0.009	49148 ±6966	1.376			
NP-000394	0.127 ±0.004	45263 ±1476	1.267			
NP-000396	0.117 ±0.006	52739 ±5096	1.477			
NP-000408	0.119 ±0.005	43649 ±585	1.222			
NP-000409	0.118 ±0.004	43848 ±7313	1.228			
NP-000416	0.111 ±0.001	39235 ±259	1.099			
NP-000417	0.114 ±0.003	36182 ±4601	1.013			
NP-000420	0.115 ±0.007	34712 ±5252	0.972			
NP-000422	0.118 ±0.004	34648 ±1971	0.970			
NP-000423	0.121 ±0.004	64268 ±7219	1.721			
NP-000424	0.116 ±0.005	58959 ±4818	1.579			
NP-000426	0.108 ±0.006	36965 ±1014	1.035			
NP-000427	0.120 ±0.007	38248 ±3165	1.071			
NP-000434	0.116 ±0.003	25182 ±899	0.705			
NP-000437	0.122 ±0.002	61755 ±5726	1.654			
NP-000442	0.123 ±0.005	57019 ±8238	1.597			
NP-000445	0.121 ±0.004	39656 ±3695	1.110			
NP-000447	0.110 ±0.010	28569 ±3120	0.800			
NP-000448	0.137 ±0.012	21916 ±1748	0.560			
NP-000453	0.109 ±0.012	33609 ±702	0.941			
NP-000454	0.112 ±0.005	39409 ±3980	1.103			
NP-000455	0.117 ±0.002	38754 ±2426	1.085			
NP-000460	0.116 ±0.001	32072 ±761	0.898			
NP-000461	0.123 ±0.009	42613 ±3972	1.193			
NP-000463	0.119 ±0.006	50886 ±4166	1.425			
NP-000466	0.118 ±0.004	53448 ±9596	1.497			
NP-000468	0.108 ±0.004	40189 ±3125	1.125			

NP-000472	0.112 ± 0.005	28631 ± 599	0.802			
NP-000474	0.122 ± 0.006	33535 ± 2038	0.939			
NP-000476	0.123 ± 0.007	38114 ± 1049	1.067			
NP-000477	0.123 ± 0.001	37603 ± 2677	1.053			
NP-000479	0.118 ± 0.004	36600 ± 5235	1.025			
NP-000481	0.116 ± 0.005	36757 ± 2201	1.029			
NP-000484	0.134 ± 0.008	31713 ± 1038	0.888			
NP-000495	0.109 ± 0.006	27024 ± 606	0.757			
NP-000497	0.116 ± 0.007	45785 ± 3590	1.282			
NP-000498	0.113 ± 0.005	48740 ± 780	1.365			
NP-000502	0.120 ± 0.003	40059 ± 5609	1.122			
NP-000505	0.111 ± 0.001	38858 ± 6489	1.088			
NP-000512	0.120 ± 0.007	34804 ± 1666	0.974			
NP-000518	0.115 ± 0.005	57258 ± 1708	1.533			
NP-000519	0.106 ± 0.003	41310 ± 1247	1.157			
NP-000520	0.121 ± 0.001	50764 ± 2056	1.421			
NP-000521	0.114 ± 0.008	32547 ± 4169	0.911			
NP-000524	0.110 ± 0.001	50338 ± 2595	1.409			
NP-000528	0.117 ± 0.005	27588 ± 1124	0.772			
NP-000529	0.116 ± 0.002	43237 ± 505	1.211			
NP-000531	0.128 ± 0.004	42516 ± 3986	1.190			
NP-000533	0.121 ± 0.003	34931 ± 2282	0.978			
NP-000535	0.123 ± 0.005	45321 ± 4842	1.269			
NP-000536	0.115 ± 0.004	26139 ± 550	0.732			
NP-000539	0.113 ± 0.004	32640 ± 2098	0.914			
NP-000541	0.120 ± 0.011	46182 ± 1599	1.293			
NP-000542	0.115 ± 0.001	53204 ± 2944	1.490			
NP-000545	0.117 ± 0.002	46033 ± 4568	1.289			
NP-000547	0.122 ± 0.016	28896 ± 2680	0.809			
NP-000557	0.134 ± 0.005	32613 ± 5213	0.913			
NP-000560	0.113 ± 0.009	41985 ± 11269	1.176			
NP-000562	0.109 ± 0.003	59547 ± 3224	1.594			

NP-000563	0.106 ±0.009	34523 ±4192	0.967			
NP-000564	0.119 ±0.003	41497 ±1519	1.162			
NP-000565	0.110 ±0.010	40306 ±5872	1.129			
NP-000567	0.116 ±0.003	46582 ±3663	1.304			
NP-000568	0.115 ±0.006	73476 ±6133	1.967			
NP-000569	0.123 ±0.007	33882 ±875	0.949			
NP-000572	0.117 ±0.003	23930 ±1055	0.670			
NP-000574	0.122 ±0.009	34281 ±3895	0.960			
NP-000576	0.117 ±0.004	47503 ±2266	1.330			
NP-000580	0.106 ±0.007	40792 ±3315	1.142			
NP-000588	0.123 ±0.002	36324 ±1059	1.017			
NP-000591	0.117 ±0.007	54249 ±2753	1.453			
NP-000592	0.124 ±0.004	45906 ±1302	1.285			
NP-000593	0.117 ±0.006	37696 ±1881	1.055			
NP-000594	0.111 ±0.003	39974 ±5588	1.119			
NP-000595	0.121 ±0.003	51179 ±3129	1.433			
NP-000597	0.129 ±0.010	33658 ±809	0.942			
NP-000598	0.122 ±0.002	31172 ±1302	0.873			
NP-000609	0.124 ±0.013	25942 ±2622	0.726			
NP-000612	0.116 ±0.009	32665 ±3202	0.915			
NP-000613	0.115 ±0.012	41862 ±4277	1.172			
NP-000615	0.136 ±0.013	45471 ±4448	1.273			
NP-000616	0.125 ±0.005	74203 ±8354	1.987			
NP-000629	0.106 ±0.006	32368 ±1577	0.906			
NP-000633	0.127 ±0.009	38260 ±5045	1.071			
NP-000635	0.116 ±0.004	38705 ±1226	1.084			
NP-000642	0.115 ±0.004	54640 ±9752	1.530			
NP-000644	0.126 ±0.003	51304 ±4340	1.436			
NP-000645	0.121 ±0.005	56492 ±4912	1.513			
NP-000648	0.110 ±0.002	41397 ±2657	1.159			
NP-000654	0.116 ±0.003	72262 ±4289	1.935			
NP-000658	0.117 ±0.007	26947 ±2240	0.755			

NP-000668	0.120 ± 0.004	58026 ± 5050	1.554			
NP-000679	0.120 ± 0.006	38258 ± 3486	1.071			
NP-000680	0.120 ± 0.003	28323 ± 1611	0.793			
NP-000682	0.119 ± 0.006	41251 ± 3745	1.155			
NP-000685	0.112 ± 0.011	37840 ± 4441	1.059			
NP-000688	0.117 ± 0.007	32875 ± 1281	0.920			
NP-000690	0.120 ± 0.010	43618 ± 2298	1.221			
NP-000694	0.122 ± 0.006	60040 ± 3998	1.608			
NP-000695	0.106 ± 0.002	35152 ± 3083	0.984			
NP-000702	0.115 ± 0.002	34405 ± 1676	0.963			
NP-000703	0.115 ± 0.004	43231 ± 3322	1.210			
NP-000704	0.123 ± 0.004	36566 ± 3862	1.024			
NP-000708	0.116 ± 0.002	34668 ± 4165	0.971			
NP-000712	0.113 ± 0.002	41338 ± 1347	1.157			
NP-000715	0.110 ± 0.006	49220 ± 7720	1.378			
NP-000720	0.127 ± 0.009	33123 ± 5422	0.927			
NP-000724	0.121 ± 0.007	29548 ± 1116	0.827			
NP-000730	0.115 ± 0.016	27720 ± 3394	0.776			
NP-000732	0.110 ± 0.003	40444 ± 6518	1.132			
NP-000734	0.112 ± 0.006	25607 ± 1386	0.717			
NP-000739	0.116 ± 0.003	33620 ± 8183	0.941			
NP-000743	0.107 ± 0.007	41256 ± 4744	1.155			
NP-000745	0.112 ± 0.004	54412 ± 3775	1.524			
NP-000747	0.122 ± 0.010	42355 ± 1567	1.186			
NP-000748	0.128 ± 0.012	34503 ± 1997	0.966			
NP-000749	0.114 ± 0.005	40333 ± 1626	1.129			
NP-000750	0.119 ± 0.004	33662 ± 911	0.943			
NP-000753	0.119 ± 0.002	41623 ± 4043	1.165			
NP-000754	0.114 ± 0.008	27341 ± 2283	0.766			
NP-000759	0.111 ± 0.001	71414 ± 7714	1.912			
NP-000760	0.118 ± 0.005	73242 ± 3235	1.961			
NP-000762	0.122 ± 0.007	52851 ± 7128	1.480			

NP-000763	0.115 ±0.004	32673 ±1380	0.915			
NP-000765	0.120 ±0.004	44493 ±666	1.246			
NP-000767	0.123 ±0.010	59734 ±6190	1.599			
NP-000770	0.113 ±0.006	37105 ±2778	1.039			
NP-000781	0.116 ±0.005	51612 ±4843	1.445			
NP-000784	0.116 ±0.004	33314 ±2008	0.933			
NP-000788	0.117 ±0.008	33659 ±5504	0.942			
NP-000789	0.114 ±0.001	41262 ±3404	1.155			
NP-000790	0.113 ±0.003	25352 ±1377	0.710			
NP-000792	0.122 ±0.003	53379 ±4617	1.495			
NP-000794	0.119 ±0.001	39921 ±337	1.118			
NP-000795	0.122 ±0.003	26123 ±4047	0.731			
NP-000796	0.118 ±0.006	49664 ±4378	1.391			
NP-000803	0.122 ±0.004	46590 ±1982	1.304			
NP-000805	0.118 ±0.003	40209 ±2282	1.126			
NP-000808	0.114 ±0.003	43686 ±5952	1.223			
NP-000809	0.114 ±0.007	38518 ±2048	1.078			
NP-000811	0.118 ±0.002	45911 ±2140	1.285			
NP-000812	0.115 ±0.003	74511 ±6571	1.995			
NP-000814	0.120 ±0.002	30487 ±1187	0.854			
NP-000816	0.119 ±0.005	31313 ±4746	0.877			
NP-000824	0.111 ±0.004	31597 ±1827	0.885			
NP-000825	0.123 ±0.005	48973 ±7135	1.371			
NP-000831	0.114 ±0.006	52741 ±6743	1.477			
NP-000833	0.119 ±0.008	50190 ±1248	1.405			
NP-000835	0.121 ±0.003	35520 ±1521	0.995			
NP-000836	0.117 ±0.004	42119 ±1317	1.179			
NP-000838	0.118 ±0.004	35576 ±2811	0.996			
NP-000839	0.114 ±0.013	42989 ±1719	1.204			
NP-000841	0.107 ±0.008	42616 ±3813	1.193			
NP-000842	0.120 ±0.005	34214 ±1927	0.958			
NP-000843	0.128 ±0.008	54609 ±8770	1.529			

NP-000845	0.119 ±0.002	35446 ±616	0.992			
NP-000846	0.117 ±0.005	36922 ±5565	1.034			
NP-000849	0.113 ±0.002	35953 ±1843	1.007			
NP-000850	0.109 ±0.002	41673 ±366	1.167			
NP-000851	0.112 ±0.004	33464 ±899	0.937			
NP-000852	0.134 ±0.007	44304 ±2007	1.240			
NP-000853	0.126 ±0.009	63673 ±7635	1.705			
NP-000854	0.124 ±0.010	31421 ±1973	0.880			
NP-000861	0.124 ±0.005	31542 ±8044	0.883			
NP-000863	0.119 ±0.005	59984 ±4435	1.606			
NP-000871	0.107 ±0.003	59738 ±4233	1.599			
NP-000872	0.116 ±0.001	40224 ±1454	1.126			
NP-000874	0.116 ±0.003	31073 ±1189	0.870			
NP-000875	0.117 ±0.002	41073 ±4970	1.150			
NP-000876	0.113 ±0.004	48341 ±4011	1.354			
NP-000884	0.110 ±0.007	44545 ±2111	1.247			
NP-000886	0.115 ±0.003	31814 ±1140	0.891			
NP-000887	0.115 ±0.002	36681 ±3009	1.027			
NP-000888	0.129 ±0.006	32145 ±2896	0.900			
NP-000890	0.121 ±0.014	45983 ±4946	1.287			
NP-000891	0.108 ±0.004	47511 ±5593	1.330			
NP-000892	0.124 ±0.001	41596 ±1255	1.165			
NP-000896	0.116 ±0.003	34018 ±7767	0.952			
NP-000897	0.112 ±0.005	56003 ±2160	1.499			
NP-000900	0.117 ±0.004	43138 ±3252	1.208			
NP-000902	0.113 ±0.012	37884 ±3019	1.061			
NP-000907	0.120 ±0.005	30154 ±2238	0.844			
NP-000914	0.113 ±0.005	44719 ±3238	1.252			
NP-000916	0.116 ±0.002	38627 ±3613	1.082			
NP-000919	0.105 ±0.005	40998 ±1653	1.148			
NP-000921	0.121 ±0.005	26284 ±379	0.736			
NP-000922	0.103 ±0.003	49259 ±2846	1.379			

NP-000923	0.113 ±0.003	44224 ±3741	1.238			
NP-000927	0.124 ±0.009	42078 ±958	1.178			
NP-000928	0.108 ±0.009	40593 ±1920	1.137			
NP-000930	0.111 ±0.005	57500 ±3737	1.540			
NP-000934	0.110 ±0.005	42350 ±2305	1.186			
NP-000936	0.120 ±0.008	33624 ±3300	0.941			
NP-000946	0.112 ±0.002	31909 ±2100	0.893			
NP-000947	0.117 ±0.008	59989 ±1862	1.606			
NP-000955	0.108 ±0.006	47179 ±2013	1.321			
NP-000956	0.113 ±0.001	35581 ±9515	0.996			
NP-000958	0.105 ±0.004	47455 ±5374	1.329			
NP-000959	0.118 ±0.002	48758 ±6687	1.365			
NP-000960	0.121 ±0.002	37501 ±3157	1.050			
NP-000961	0.116 ±0.001	31720 ±177	0.888			
NP-000963	0.129 ±0.005	30431 ±2436	0.852			
NP-000967	0.127 ±0.008	52567 ±7303	1.472			
NP-000968	0.112 ±0.003	36408 ±938	1.019			
NP-000971	0.116 ±0.004	33616 ±1269	0.941			
NP-000973	0.126 ±0.008	30717 ±826	0.860			
NP-000974	0.132 ±0.012	58419 ±10451	1.636			
NP-000975	0.120 ±0.002	28002 ±905	0.784			
NP-000978	0.113 ±0.001	38192 ±2032	1.069			
NP-000979	0.123 ±0.005	32465 ±4961	0.909			
NP-000987	0.12 ±0.004	53110 ±2944	1.487			
NP-000992	0.121 ±0.005	47455 ±1485	1.329			
NP-000996	0.120 ±0.006	44730 ±7508	1.252			
NP-000997	0.114 ±0.005	29805 ±1659	0.835			
NP-001000	0.115 ±0.003	41907 ±6129	1.173			
NP-001001	0.112 ±0.006	37848 ±5345	1.060			
NP-001002	0.122 ±0.006	46439 ±4853	1.300			
NP-001003	0.127 ±0.006	43283 ±4226	1.212			
NP-001005	0.129 ±0.003	63456 ±8916	1.777			

NP-001007	0.119 ±0.006	72219 ±10706	1.934			
NP-001008	0.128 ±0.007	28444 ±1553	0.796			
NP-001009	0.120 ±0.008	30980 ±2684	0.867			
NP-001010	0.096 ±0.004 *					
NP-001014	0.102 ±0.003	26803 ±2630	0.750			
NP-001015	0.090 ±0.004 **					
NP-001019	0.118 ±0.007	36318 ±5894	1.017			
NP-001022	0.119 ±0.012	33588 ±1866	0.940			
NP-001023	0.122 ±0.005	54049 ±2364	1.447			
NP-001024	0.121 ±0.003	25139 ±6743	0.704			
NP-001030	0.116 ±0.004	44580 ±2466	1.248			
NP-001031	0.115 ±0.007	31508 ±2087	0.882			
NP-001034	0.109 ±0.005	35046 ±4656	0.981			
NP-001038	0.122 ±0.004	35392 ±1691	0.991			
NP-001040	0.111 ±0.006	37632 ±1753	1.054			
NP-001046	0.118 ±0.006	37793 ±2833	1.058			
NP-001048	0.120 ±0.011	42512 ±3958	1.190			
NP-001049	0.124 ±0.005	41185 ±6375	1.153			
NP-001050	0.108 ±0.001	20451 ±2049	0.573			
NP-001053	0.108 ±0.003	34466 ±2434	0.965			
NP-001057	0.109 ±0.005	70111 ±3605	1.877			
NP-001058	0.113 ±0.006	31314 ±1150	0.877			
NP-001060	0.134 ±0.007	29994 ±2576	0.840			
NP-001061	0.112 ±0.003	60293 ±1197	1.614			
NP-001065	0.124 ±0.008	46187 ±4300	1.293			
NP-001066	0.118 ±0.003	55528 ±4840	1.555			
NP-001068	0.115 ±0.005	19365 ±1146	0.519			
NP-001071	0.111 ±0.004	39414 ±4062	1.104			
NP-001076	0.118 ±0.001	43609 ±3941	1.221			
NP-001079	0.124 ±0.004	40751 ±702	1.141			
NP-001081	0.119 ±0.005	36671 ±1882	1.027			
NP-001088	0.118 ±0.011	31248 ±2018	0.875			

NP-001089	0.115 ± 0.003	29419 ± 1174	0.824			
NP-001090	0.063 ± 0.007 **					
NP-001097	0.119 ± 0.004	36265 ± 1215	1.015			
NP-001099	0.109 ± 0.006	25128 ± 4035	0.704			
NP-001100	0.117 ± 0.007	39862 ± 1217	1.116			
NP-001106	0.110 ± 0.003	33433 ± 706	0.936			
NP-001107	0.109 ± 0.010	50969 ± 7821	1.427			
NP-001109	0.116 ± 0.007	45492 ± 4192	1.274			
NP-001112	0.110 ± 0.001	41019 ± 3203	1.149			
NP-001122	0.113 ± 0.002	37388 ± 1979	1.047			
NP-001130	0.118 ± 0.002	43099 ± 1635	1.207			
NP-001134	0.112 ± 0.005	33951 ± 548	0.951			
NP-001140	0.111 ± 0.007	34564 ± 4816	0.968			
NP-001150	0.137 ± 0.006	30165 ± 2362	0.845			
NP-001151	0.118 ± 0.009	25209 ± 811	0.706			
NP-001155	0.113 ± 0.003	32463 ± 6195	0.909			
NP-001156	0.113 ± 0.005	48283 ± 7031	1.352			
NP-001159	0.123 ± 0.007	57704 ± 4182	1.616			
NP-001161	0.137 ± 0.006	45128 ± 3000	1.264			
NP-001173	0.115 ± 0.001	47773 ± 8074	1.338			
NP-001174	0.128 ± 0.009	38135 ± 9918	1.068			
NP-001176	0.111 ± 0.008	30947 ± 3013	0.866			
NP-001177	0.110 ± 0.001	33458 ± 2465	0.937			
NP-001179	0.114 ± 0.003	34088 ± 4078	0.954			
NP-001181	0.121 ± 0.008	40626 ± 1535	1.138			
NP-001184	0.109 ± 0.002	55796 ± 5616	1.562			
NP-001185	0.111 ± 0.008	41166 ± 490	1.153			
NP-001187	0.111 ± 0.006	27520 ± 1739	0.771			
NP-001195	0.116 ± 0.002	49172 ± 1386	1.377			
NP-001196	0.117 ± 0.005	36672 ± 2703	1.027			
NP-001200	0.117 ± 0.001	41993 ± 5585	1.176			
NP-001203	0.112 ± 0.007	36446 ± 1681	1.020			

NP-001204	0.105 ±0.006	37763 ±5822	1.057			
NP-001207	0.118 ±0.008	50663 ±3468	1.419			
NP-001209	0.120 ±0.003	30401 ±1075	0.851			
NP-001211	0.116 ±0.007	23936 ±1542	0.670			
NP-001216	0.117 ±0.010	44784 ±2764	1.254			
NP-001218	0.138 ±0.007	47858 ±3174	1.340			
NP-001224	0.123 ±0.004	37619 ±416	1.053			
NP-001229	0.121 ±0.003	47982 ±1579	1.343			
NP-001232	0.114 ±0.001	54240 ±9463	1.519			
NP-001236	0.122 ±0.007	27672 ±1585	0.775			
NP-001240	0.119 ±0.004	44654 ±2597	1.250			
NP-001244	0.116 ±0.004	43268 ±3867	1.211			
NP-001246	0.116 ±0.002	43849 ±824	1.228			
NP-001249	0.118 ±0.003	38053 ±3778	1.065			
NP-001251	0.113 ±0.003	33596 ±1632	0.941			
NP-001257	0.117 ±0.006	34316 ±1332	0.961			
NP-001258	0.116 ±0.004	33479 ±3368	0.937			
NP-001263	0.113 ±0.005	34118 ±4943	0.955			
NP-001265	0.122 ±0.002	37530 ±6695	1.051			
NP-001266	0.115 ±0.003	29889 ±1289	0.837			
NP-001267	0.117 ±0.001	53317 ±2027	1.428			
NP-001268	0.116 ±0.003	36209 ±2448	1.014			
NP-001269	0.121 ±0.004	52659 ±4226	1.474			
NP-001271	0.119 ±0.013	53148 ±2831	1.423			
NP-001273	0.116 ±0.003	38866 ±2333	1.088			
NP-001277	0.122 ±0.007	35107 ±670	0.983			
NP-001279	0.110 ±0.005	46434 ±3667	1.300			
NP-001281	0.110 ±0.002	43328 ±2548	1.213			
NP-001282	0.129 ±0.005	35394 ±1919	0.991			
NP-001291	0.096 ±0.002 *					
NP-001293	0.121 ±0.002	41714 ±3658	1.168			
NP-001297	0.121 ±0.009	52125 ±2165	1.459			

NP-001302	0.112 ± 0.002	37393 ± 2546	1.047			
NP-001303	0.110 ± 0.001	34082 ± 7569	0.954			
NP-001305	0.114 ± 0.005	36044 ± 2476	1.009			
NP-001306	0.119 ± 0.004	42128 ± 6622	1.180			
NP-001307	0.111 ± 0.005	32963 ± 1995	0.923			
NP-001308	0.115 ± 0.002	29727 ± 1073	0.832			
NP-001309	0.111 ± 0.008	36514 ± 2330	1.022			
NP-001310	0.118 ± 0.004	31818 ± 1275	0.891			
NP-001314	0.122 ± 0.001	31237 ± 2855	0.875			
NP-001315	0.122 ± 0.008	35618 ± 1857	0.997			
NP-001317	0.122 ± 0.004	35260 ± 805	0.987			
NP-001319	0.109 ± 0.002	41064 ± 3752	1.150			
NP-001321	0.113 ± 0.010	37195 ± 2426	1.041			
NP-001325	0.115 ± 0.001	27651 ± 4632	0.774			
NP-001328	0.116 ± 0.003	41391 ± 2623	1.159			
NP-001331	0.114 ± 0.002	38110 ± 4386	1.067			
NP-001332	0.113 ± 0.002	46004 ± 2390	1.288			
NP-001333	0.120 ± 0.005	29979 ± 2061	0.839			
NP-001338	0.112 ± 0.003	39513 ± 1082	1.106			
NP-001362	0.119 ± 0.010	35159 ± 3378	0.984			
NP-001363	0.120 ± 0.004	35031 ± 575	0.981			
NP-001364	0.098 ± 0.018	44336 ± 2330	1.241			
NP-001365	0.115 ± 0.004	31387 ± 3259	0.879			
NP-001366	0.117 ± 0.008	48986 ± 4457	1.372			
NP-001372	0.127 ± 0.006	34392 ± 1941	0.963			
NP-001373	0.137 ± 0.007	64715 ± 9986	1.812			
NP-001376	0.121 ± 0.007	44072 ± 1077	1.234			
NP-001377	0.125 ± 0.005	34046 ± 454	0.953			
NP-001383	0.134 ± 0.009	37916 ± 3952	1.062			
NP-001384	0.118 ± 0.002	32700 ± 1959	0.916			
NP-001392	0.118 ± 0.003	21957 ± 1895	0.615			
NP-001393	0.114 ± 0.007	44583 ± 3510	1.248			

NP-001401	0.111 ±0.004	41510 ±4246	1.162			
NP-001402	0.095 ±0.005 *					
NP-001403	0.123 ±0.001	42850 ±915	1.200			
NP-001409	0.123 ±0.007	53969 ±5628	1.511			
NP-001415	0.115 ±0.002	29701 ±990	0.832			
NP-001421	0.118 ±0.002	41290 ±2323	1.156			
NP-001422	0.055 ±0.001 **					
NP-001425	0.118 ±0.005	33509 ±580	0.938			
NP-001426	0.118 ±0.003	41486 ±2643	1.162			
NP-001427	0.125 ±0.002	38471 ±1148	1.077			
NP-001431	0.128 ±0.002	44776 ±1344	1.254			
NP-001433	0.128 ±0.001	39996 ±3520	1.120			
NP-001434	0.117 ±0.005	36036 ±2993	1.009			
NP-001436	0.116 ±0.007	36251 ±2955	1.015			
NP-001443	0.118 ±0.008	23814 ±730	0.667			
NP-001445	0.113 ±0.005	63401 ±7323	1.698			
NP-001446	0.117 ±0.006	39041 ±3715	1.093			
NP-001451	0.106 ±0.010	40514 ±1508	1.134			
NP-001452	0.113 ±0.002	50287 ±2064	1.408			
NP-001454	0.112 ±0.004	44162 ±8431	1.237			
NP-001455	0.122 ±0.010	49534 ±4019	1.387			
NP-001456	0.119 ±0.004	50467 ±3480	1.413			
NP-001458	0.121 ±0.004	40406 ±1685	1.131			
NP-001459	0.121 ±0.004	55743 ±5270	1.561			
NP-001471	0.125 ±0.010	31289 ±2914	0.876			
NP-001473	0.111 ±0.004	27455 ±3416	0.769			
NP-001475	0.124 ±0.010	20812 ±1619	0.557			
NP-001476	0.121 ±0.002	39008 ±4432	1.092			
NP-001478	0.119 ±0.008	43303 ±3498	1.212			
NP-001480	0.126 ±0.011	39901 ±2098	1.117			
NP-001482	0.118 ±0.003	37814 ±800	1.059			
NP-001487	0.121 ±0.007	34544 ±2069	0.967			

NP-001491	0.120 ± 0.003	28321 ± 8830	0.793			
NP-001494	0.118 ± 0.002	47549 ± 2333	1.331			
NP-001509	0.114 ± 0.006	44712 ± 3564	1.252			
NP-001514	0.121 ± 0.004	38643 ± 2632	1.082			
NP-001516	0.114 ± 0.005	42136 ± 934	1.180			
NP-001522	0.109 ± 0.003	37729 ± 7122	1.056			
NP-001523	0.116 ± 0.006	41383 ± 9287	1.159			
NP-001527	0.118 ± 0.004	37716 ± 7518	1.056			
NP-001531	0.116 ± 0.003	49035 ± 6224	1.373			
NP-001533	0.122 ± 0.005	34121 ± 2833	0.955			
NP-001547	0.111 ± 0.002	34281 ± 4486	0.960			
NP-001548	0.093 ± 0.007 *					
NP-001551	0.115 ± 0.004	40372 ± 6191	1.130			
NP-001558	0.115 ± 0.003	54737 ± 5630	1.533			
NP-001559	0.116 ± 0.003	37911 ± 1647	1.061			
NP-001561	0.123 ± 0.006	52353 ± 2703	1.466			
NP-001562	0.110 ± 0.010	46081 ± 6991	1.290			
NP-001566	0.119 ± 0.006	41576 ± 1292	1.164			
NP-001571	0.118 ± 0.002	24631 ± 2454	0.690			
NP-001572	0.109 ± 0.006	25834 ± 2977	0.723			
NP-001577	0.123 ± 0.010	45390 ± 4399	1.271			
NP-001578	0.115 ± 0.002	51020 ± 3808	1.429			
NP-001580	0.119 ± 0.008	21727 ± 1305	0.608			
NP-001581	0.134 ± 0.006	48726 ± 2999	1.364			
NP-001582	0.112 ± 0.005	27206 ± 1960	0.762			
NP-001584	0.107 ± 0.003	49271 ± 7065	1.380			
NP-001586	0.115 ± 0.010	38368 ± 2619	1.074			
NP-001595	0.111 ± 0.004	41007 ± 6481	1.148			
NP-001600	0.113 ± 0.010	32212 ± 1517	0.902			
NP-001602	0.117 ± 0.001	60725 ± 212	1.626			
NP-001604	0.119 ± 0.005	32112 ± 2253	0.899			
NP-001605	0.122 ± 0.002	36872 ± 2711	1.032			

NP-001610	0.116 ±0.004	29625 ±4656	0.829			
NP-001615	0.116 ±0.001	43950 ±3237	1.231			
NP-001617	0.112 ±0.001	50790 ±7293	1.422			
NP-001622	0.115 ±0.003	26662 ±5004	0.747			
NP-001623	0.118 ±0.012	36993 ±3511	1.036			
NP-001624	0.116 ±0.005	30995 ±3518	0.868			
NP-001625	0.112 ±0.007	48336 ±2685	1.353			
NP-001626	0.121 ±0.003	31895 ±807	0.893			
NP-001629	0.114 ±0.006	31578 ±2160	0.884			
NP-001636	0.116 ±0.002	42726 ±1739	1.196			
NP-001637	0.109 ±0.006	44446 ±2745	1.244			
NP-001639	0.117 ±0.005	65524 ±379	1.754			
NP-001640	0.117 ±0.005	41112 ±2970	1.151			
NP-001642	0.120 ±0.010	48507 ±5519	1.358			
NP-001647	0.119 ±0.003	40707 ±693	1.140			
NP-001648	0.115 ±0.008	33036 ±716	0.925			
NP-001652	0.109 ±0.001	32480 ±896	0.909			
NP-001653	0.116 ±0.005	36283 ±1609	1.016			
NP-001657	0.116 ±0.002	25116 ±374	0.703			
NP-001662	0.115 ±0.004	33972 ±988	0.951			
NP-001664	0.123 ±0.005	60222 ±1992	1.612			
NP-001671	0.124 ±0.020	47577 ±2626	1.332			
NP-001672	0.120 ±0.006	34935 ±1740	0.978			
NP-001674	0.111 ±0.007	32268 ±391	0.903			
NP-001679	0.123 ±0.007	58787 ±9006	1.646			
NP-001680	0.119 ±0.004	40648 ±5155	1.138			
NP-001682	0.119 ±0.002	32842 ±3193	0.920			
NP-001683	0.124 ±0.004	52185 ±1286	1.461			
NP-001685	0.121 ±0.005	38162 ±4440	1.069			
NP-001686	0.125 ±0.008	38444 ±3063	1.076			
NP-001687	0.125 ±0.003	38456 ±1778	1.077			
NP-001689	0.114 ±0.004	39903 ±4527	1.117			

NP-001692	0.112 ± 0.002	36640 ± 1125	1.026			
NP-001698	0.119 ± 0.002	33085 ± 1222	0.926			
NP-001701	0.116 ± 0.004	48727 ± 11971	1.364			
NP-001702	0.116 ± 0.006	55305 ± 3880	1.481			
NP-001703	0.131 ± 0.007	24870 ± 1639	0.696			
NP-001704	0.114 ± 0.009	36625 ± 5453	1.025			
NP-001717	0.121 ± 0.003	35558 ± 1438	0.996			
NP-001718	0.113 ± 0.006	47479 ± 3484	1.329			
NP-001730	0.112 ± 0.002	32875 ± 8475	0.920			
NP-001733	0.124 ± 0.002	56051 ± 7117	1.569			
NP-001735	0.108 ± 0.009	48247 ± 3479	1.351			
NP-001740	0.117 ± 0.003	24792 ± 1594	0.694			
NP-001741	0.118 ± 0.003	21959 ± 415	0.588			
NP-001742	0.124 ± 0.011	40270 ± 4519	1.128			
NP-001743	0.112 ± 0.004	55397 ± 2459	1.483			
NP-001745	0.117 ± 0.002	34467 ± 2469	0.965			
NP-001752	0.119 ± 0.003	35878 ± 2796	1.005			
NP-001753	0.124 ± 0.012	31648 ± 1087	0.886			
NP-001755	0.116 ± 0.003	42740 ± 4935	1.197			
NP-001757	0.116 ± 0.003	34147 ± 1303	0.956			
NP-001758	0.113 ± 0.002	27810 ± 253	0.779			
NP-001765	0.101 ± 0.010	34974 ± 2546	0.979			
NP-001770	0.113 ± 0.003	39919 ± 937	1.118			
NP-001781	0.111 ± 0.003	34481 ± 1076	0.965			
NP-001782	0.108 ± 0.005	26922 ± 3927	0.754			
NP-001787	0.126 ± 0.008	51729 ± 9626	1.448			
NP-001788	0.122 ± 0.005	34474 ± 4683	0.965			
NP-001789	0.120 ± 0.004	37003 ± 1107	1.036			
NP-001790	0.109 ± 0.002	32473 ± 2762	0.909			
NP-001797	0.117 ± 0.001	38993 ± 3081	1.092			
NP-001798	0.114 ± 0.002	42986 ± 5878	1.204			
NP-001802	0.114 ± 0.003	32250 ± 2255	0.903			

NP-001807	0.121 ± 0.007	21924 ± 1109	0.614			
NP-001810	0.119 ± 0.005	39520 ± 2178	1.107			
NP-001816	0.108 ± 0.005	27931 ± 201	0.782			
NP-001821	0.104 ± 0.005	29030 ± 2283	0.813			
NP-001823	0.121 ± 0.004	35007 ± 4676	0.980			
NP-001824	0.116 ± 0.005	36951 ± 1710	1.035			
NP-001831	0.121 ± 0.001	48611 ± 2808	1.361			
NP-001833	0.113 ± 0.004	27642 ± 587	0.774			
NP-001834	0.126 ± 0.003	29800 ± 2509	0.834			
NP-001839	0.122 ± 0.007	52387 ± 6025	1.467			
NP-001843	0.131 ± 0.008	36245 ± 3378	1.015			
NP-001848	0.114 ± 0.008	39876 ± 2300	1.117			
NP-001850	0.120 ± 0.002	44458 ± 3108	1.245			
NP-001854	0.120 ± 0.009	23064 ± 3803	0.646			
NP-001864	0.110 ± 0.005	32390 ± 1309	0.907			
NP-001865	0.120 ± 0.003	36173 ± 2140	1.013			
NP-001872	0.111 ± 0.003	48202 ± 1353	1.350			
NP-001873	0.117 ± 0.001	46025 ± 1987	1.289			
NP-001890	0.126 ± 0.009	45068 ± 1400	1.262			
NP-001894	0.135 ± 0.007	47953 ± 6137	1.343			
NP-001895	0.116 ± 0.005	34337 ± 4526	0.961			
NP-001899	0.094 ± 0.004 *					
NP-001911	0.122 ± 0.004	30141 ± 3138	0.844			
NP-001913	0.120 ± 0.006	50313 ± 4197	1.409			
NP-001916	0.119 ± 0.001	49934 ± 2979	1.398			
NP-001917	0.114 ± 0.007	44689 ± 3014	1.251			
NP-001919	0.122 ± 0.007	35607 ± 1569	0.997			
NP-001920	0.128 ± 0.005	38670 ± 4584	1.083			
NP-001924	0.115 ± 0.003	31955 ± 7849	0.895			
NP-001925	0.118 ± 0.006	43057 ± 3212	1.206			
NP-001930	0.113 ± 0.014	46945 ± 1279	1.314			
NP-001933	0.121 ± 0.008	50116 ± 7293	1.403			

NP-001957	0.117 ±0.005	50464 ±6061	1.413			
NP-001960	0.126 ±0.005	30124 ±1636	0.843			
NP-001962	0.114 ±0.002	41860 ±6089	1.172			
NP-001963	0.116 ±0.004	32706 ±2104	0.916			
NP-001964	0.124 ±0.006	33186 ±1632	0.929			
NP-001966	0.114 ±0.003	57130 ±7195	1.530			
NP-001967	0.125 ±0.007	61969 ±7514	1.659			
NP-001972	0.112 ±0.003	66052 ±4902	1.769			
NP-001975	0.130 ±0.006	43700 ±3361	1.224			
NP-001976	0.104 ±0.006	38691 ±4602	1.083			
NP-001978	0.114 ±0.016	27972 ±5343	0.783			
NP-001984	0.116 ±0.008	33637 ±1289	0.942			
NP-001985	0.113 ±0.001	53126 ±6591	1.487			
NP-001986	0.122 ±0.005	40474 ±758	1.133			
NP-001987	0.118 ±0.002	39579 ±764	1.108			
NP-001989	0.118 ±0.003	42351 ±3564	1.186			
NP-001990	0.124 ±0.010	51343 ±4569	1.438			
NP-001991	0.118 ±0.005	55570 ±8971	1.556			
NP-002000	0.116 ±0.006	57344 ±3433	1.535			
NP-002011	0.114 ±0.009	46287 ±1838	1.296			
NP-002013	0.111 ±0.004	31416 ±4989	0.880			
NP-002015	0.121 ±0.007	35134 ±1730	0.984			
NP-002021	0.138 ±0.029	24664 ±353	0.691			
NP-002022	0.119 ±0.007	37803 ±5760	1.058			
NP-002023	0.119 ±0.004	36187 ±847	1.013			
NP-002026	0.115 ±0.002	42271 ±2880	1.184			
NP-002027	0.115 ±0.005	52780 ±3650	1.478			
NP-002029	0.124 ±0.005	45776 ±2515	1.282			
NP-002031	0.117 ±0.006	36125 ±3122	1.011			
NP-002037	0.128 ±0.007	54913 ±5833	1.538			
NP-002038	0.114 ±0.002	40965 ±2832	1.147			
NP-002039	0.116 ±0.004	32972 ±1352	0.923			

NP-002043	0.109 ±0.001	41451 ±979	1.161				
NP-002044	0.125 ±0.003	63449 ±11290	1.777				
NP-002050	0.124 ±0.006	27271 ±965	0.764				
NP-002055	0.128 ±0.003	45008 ±548	1.260				
NP-002066	0.118 ±0.002	48635 ±1448	1.362				
NP-002073	0.106 ±0.002	43599 ±5312	1.221				
NP-002074	0.117 ±0.003	42541 ±3541	1.191				
NP-002075	0.112 ±0.011	37694 ±4212	1.055				
NP-002082	0.127 ±0.004	50095 ±2650	1.403				
NP-002092	0.125 ±0.011	45565 ±5579	1.276				
NP-002093	0.120 ±0.008	28376 ±1344	0.795				
NP-002094	0.113 ±0.003	42551 ±1358	1.191				
NP-002095	0.126 ±0.006	56562 ±10009	1.584				
NP-002096	0.114 ±0.004	32081 ±3485	0.898				
NP-002098	0.122 ±0.005	47668 ±2155	1.335				
NP-002099	0.123 ±0.006	34820 ±1339	0.975				
NP-002100	0.106 ±0.002	41552 ±3801	1.163				
NP-002101	0.124 ±0.007	49711 ±1752	1.392				
NP-002104	0.127 ±0.004	26929 ±434	0.754				
NP-002105	0.121 ±0.005	36650 ±2887	1.026				
NP-002106	0.129 ±0.008	36462 ±1752	1.021				
NP-002107	0.116 ±0.005	28805 ±1257	0.807				
NP-002108	0.126 ±0.005	39697 ±1676	1.111				
NP-002110	0.133 ±0.006	33480 ±1527	0.937				
NP-002114	0.122 ±0.003	42700 ±1512	1.196				
NP-002116	0.113 ±0.003	42060 ±6496	1.178				
NP-002117	0.122 ±0.009	34688 ±3700	0.971				
NP-002118	0.118 ±0.001	41276 ±1601	1.156				
NP-002121	0.110 ±0.004	39976 ±5089	1.119				
NP-002124	0.117 ±0.025	55067 ±6802	1.542				
NP-002144	0.114 ±0.002	32200 ±2241	0.902				
NP-002146	0.120 ±0.008	62469 ±4127	1.673				

NP-002151	0.116 ±0.010	29249 ±1182	0.819			
NP-002152	0.114 ±0.009	43212 ±5106	1.210			
NP-002153	0.106 ±0.003	33356 ±2840	0.934			
NP-002154	0.104 ±0.006	40862 ±2068	1.144			
NP-002158	0.113 ±0.005	35344 ±8499	0.990			
NP-002171	0.114 ±0.001	37561 ±2546	1.052			
NP-002172	0.111 ±0.002	46513 ±2048	1.302			
NP-002173	0.124 ±0.004	55329 ±4859	1.549			
NP-002175	0.108 ±0.003	33002 ±3235	0.924			
NP-002176	0.116 ±0.009	50649 ±6656	1.418			
NP-002182	0.126 ±0.002	39989 ±1613	1.120			
NP-002183	0.120 ±0.006	35239 ±4659	0.987			
NP-002184	0.107 ±0.002	50741 ±7372	1.421			
NP-002187	0.123 ±0.003	35657 ±772	0.998			
NP-002193	0.113 ±0.003	47512 ±7754	1.330			
NP-002194	0.122 ±0.011	45729 ±2316	1.280			
NP-002195	0.111 ±0.003	49807 ±5658	1.395			
NP-002196	0.124 ±0.004	59839 ±3461	1.602			
NP-002198	0.115 ±0.004	38036 ±1126	1.065			
NP-002200	0.119 ±0.004	39484 ±5671	1.106			
NP-002201	0.114 ±0.009	34066 ±1602	0.954			
NP-002202	0.112 ±0.003	33390 ±6969	0.935			
NP-002203	0.111 ±0.008	40077 ±2952	1.122			
NP-002204	0.112 ±0.004	38600 ±3164	1.081			
NP-002209	0.121 ±0.007	39166 ±1889	1.097			
NP-002211	0.116 ±0.003	31925 ±1076	0.894			
NP-002214	0.109 ±0.003	35691 ±4296	0.999			
NP-002217	0.119 ±0.004	35562 ±9498	0.996			
NP-002218	0.119 ±0.005	48512 ±4884	1.358			
NP-002223	0.119 ±0.004	35648 ±4080	0.998			
NP-002227	0.126 ±0.004	44191 ±2663	1.237			
NP-002231	0.117 ±0.005	39541 ±2058	1.107			

NP-002232	0.122 ± 0.003	40512 ± 4284	1.134			
NP-002236	0.108 ± 0.003	51194 ± 1207	1.433			
NP-002239	0.122 ± 0.004	45505 ± 4985	1.274			
NP-002243	0.115 ± 0.007	31024 ± 2114	0.869			
NP-002251	0.122 ± 0.003	41319 ± 5851	1.157			
NP-002252	0.112 ± 0.013	55290 ± 2953	1.548			
NP-002254	0.108 ± 0.005	35886 ± 3747	1.005			
NP-002257	0.122 ± 0.004	42401 ± 347	1.187			
NP-002264	0.118 ± 0.005	33046 ± 1513	0.925			
NP-002265	0.112 ± 0.007	29428 ± 1554	0.824			
NP-002270	0.126 ± 0.005	46285 ± 3292	1.296			
NP-002273	0.125 ± 0.004	40702 ± 1841	1.140			
NP-002276	0.117 ± 0.006	39625 ± 822	1.109			
NP-002291	0.120 ± 0.004	36978 ± 2974	1.035			
NP-002293	0.114 ± 0.003	37724 ± 3748	1.056			
NP-002294	0.125 ± 0.004	50423 ± 4571	1.412			
NP-002295	0.105 ± 0.007	55253 ± 1975	1.547			
NP-002297	0.112 ± 0.013	40006 ± 4205	1.120			
NP-002298	0.122 ± 0.006	49974 ± 5271	1.399			
NP-002301	0.112 ± 0.004	26914 ± 2772	0.754			
NP-002305	0.121 ± 0.003	35078 ± 833	0.982			
NP-002308	0.113 ± 0.002	39143 ± 4790	1.096			
NP-002309	0.128 ± 0.009	31484 ± 628	0.882			
NP-002310	0.113 ± 0.001	38356 ± 4962	1.074			
NP-002314	0.114 ± 0.002	31605 ± 3640	0.885			
NP-002315	0.118 ± 0.007	69209 ± 5494	1.853			
NP-002325	0.114 ± 0.003	54354 ± 8085	1.522			
NP-002326	0.110 ± 0.002	38018 ± 6351	1.064			
NP-002327	0.124 ± 0.008	35911 ± 2248	1.005			
NP-002328	0.120 ± 0.005	42693 ± 3679	1.195			
NP-002329	0.128 ± 0.004	33964 ± 2138	0.951			
NP-002330	0.129 ± 0.004	29090 ± 3933	0.815			

NP-002331	0.121 ± 0.009	36475 ± 2068	1.021			
NP-002332	0.112 ± 0.002	53835 ± 5816	1.507			
NP-002333	0.109 ± 0.003	27328 ± 2585	0.765			
NP-002341	0.111 ± 0.003	40458 ± 4106	1.133			
NP-002342	0.116 ± 0.001	46823 ± 5366	1.311			
NP-002343	0.117 ± 0.007	35451 ± 2246	0.993			
NP-002344	0.113 ± 0.005	34135 ± 2132	0.956			
NP-002347	0.116 ± 0.007	52792 ± 4285	1.478			
NP-002350	0.121 ± 0.006	41008 ± 1467	1.148			
NP-002351	0.116 ± 0.003	37084 ± 2275	1.038			
NP-002353	0.119 ± 0.002	45233 ± 304	1.266			
NP-002357	0.115 ± 0.007	43743 ± 6347	1.225			
NP-002359	0.113 ± 0.002	45389 ± 2734	1.271			
NP-002360	0.107 ± 0.003	39265 ± 2665	1.099			
NP-002364	0.107 ± 0.006	38029 ± 2250	1.065			
NP-002365	0.107 ± 0.008	27977 ± 4882	0.783			
NP-002366	0.109 ± 0.001	38967 ± 2660	1.091			
NP-002374	0.112 ± 0.004	39590 ± 4707	1.108			
NP-002381	0.124 ± 0.006	32321 ± 1405	0.905			
NP-002391	0.095 ± 0.004 *					
NP-002392	0.116 ± 0.002	46035 ± 3510	1.289			
NP-002397	0.109 ± 0.014	32924 ± 8574	0.922			
NP-002398	0.122 ± 0.002	34770 ± 3506	0.974			
NP-002399	0.092 ± 0.004 *					
NP-002405	0.126 ± 0.006	33840 ± 1548	0.948			
NP-002406	0.119 ± 0.011	62750 ± 4388	1.680			
NP-002409	0.115 ± 0.002	36849 ± 1503	1.032			
NP-002425	0.120 ± 0.006	31074 ± 3880	0.870			
NP-002426	0.114 ± 0.002	26929 ± 3541	0.754			
NP-002433	0.118 ± 0.009	47887 ± 2903	1.341			
NP-002436	0.118 ± 0.005	36509 ± 2395	1.022			
NP-002437	0.117 ± 0.012	38607 ± 2099	1.081			

NP-002438	0.118 ± 0.004	54424 ± 1440	1.457			
NP-002439	0.118 ± 0.007	34830 ± 2489	0.975			
NP-002442	0.117 ± 0.014	23124 ± 4831	0.647			
NP-002443	0.120 ± 0.001	43801 ± 1894	1.226			
NP-002445	0.121 ± 0.003	48298 ± 2098	1.352			
NP-002447	0.109 ± 0.004	47832 ± 2955	1.339			
NP-002450	0.118 ± 0.006	43630 ± 1615	1.222			
NP-002451	0.110 ± 0.002	45339 ± 2600	1.269			
NP-002456	0.113 ± 0.003	34915 ± 2174	0.978			
NP-002458	0.114 ± 0.002	35498 ± 2171	0.994			
NP-002464	0.116 ± 0.005	28451 ± 1664	0.797			
NP-002465	0.116 ± 0.004	44742 ± 2135	1.253			
NP-002466	0.120 ± 0.003	39953 ± 5451	1.119			
NP-002467	0.136 ± 0.003	25471 ± 1512	0.713			
NP-002475	0.121 ± 0.005	43492 ± 2584	1.218			
NP-002478	0.112 ± 0.007	35408 ± 2671	0.991			
NP-002480	0.129 ± 0.008	47747 ± 1096	1.337			
NP-002481	0.116 ± 0.001	51671 ± 5714	1.447			
NP-002483	0.115 ± 0.004	38493 ± 704	1.078			
NP-002486	0.120 ± 0.001	38404 ± 2747	1.075			
NP-002488	0.109 ± 0.002	39552 ± 1767	1.107			
NP-002489	0.127 ± 0.004	36817 ± 973	1.031			
NP-002490	0.116 ± 0.009	28988 ± 3198	0.812			
NP-002491	0.120 ± 0.006	30012 ± 506	0.840			
NP-002492	0.121 ± 0.007	48539 ± 7803	1.359			
NP-002493	0.116 ± 0.003	40308 ± 2980	1.129			
NP-002495	0.114 ± 0.003	35187 ± 3743	0.985			
NP-002508	0.120 ± 0.002	35685 ± 3336	0.999			
NP-002509	0.115 ± 0.001	37840 ± 2644	1.059			
NP-002510	0.119 ± 0.004	38906 ± 1409	1.089			
NP-002511	0.124 ± 0.003	56858 ± 3784	1.592			
NP-002515	0.121 ± 0.012	25996 ± 2448	0.728			

NP-002516	0.122 ± 0.005	45754 ± 3055	1.281			
NP-002517	0.122 ± 0.005	27211 ± 2105	0.762			
NP-002518	0.124 ± 0.007	55552 ± 7486	1.555			
NP-002520	0.116 ± 0.006	56629 ± 3232	1.516			
NP-002522	0.117 ± 0.003	37526 ± 2805	1.051			
NP-002523	0.121 ± 0.003	42424 ± 1414	1.188			
NP-002524	0.111 ± 0.003	30435 ± 3553	0.852			
NP-002525	0.109 ± 0.001	50276 ± 5430	1.408			
NP-002543	0.117 ± 0.002	27237 ± 1766	0.763			
NP-002545	0.118 ± 0.007	38685 ± 2510	1.083			
NP-002547	0.111 ± 0.007	48526 ± 1724	1.359			
NP-002550	0.127 ± 0.009	33088 ± 1349	0.926			
NP-002551	0.127 ± 0.006	49557 ± 8550	1.388			
NP-002558	0.121 ± 0.007	48728 ± 3796	1.364			
NP-002559	0.114 ± 0.005	45640 ± 4542	1.278			
NP-002560	0.123 ± 0.009	36831 ± 1810	1.031			
NP-002561	0.111 ± 0.003	37729 ± 1889	1.056			
NP-002563	0.115 ± 0.004	53519 ± 679	1.433			
NP-002575	0.117 ± 0.005	41064 ± 7162	1.150			
NP-002582	0.124 ± 0.006	32740 ± 1888	0.917			
NP-002584	0.116 ± 0.005	63442 ± 11062	1.776			
NP-002585	0.127 ± 0.005	29687 ± 2428	0.831			
NP-002586	0.121 ± 0.002	29468 ± 992	0.825			
NP-002587	0.118 ± 0.006	41789 ± 1861	1.170			
NP-002588	0.121 ± 0.001	37088 ± 1941	1.038			
NP-002592	0.109 ± 0.002	36146 ± 4754	1.012			
NP-002598	0.124 ± 0.003	38871 ± 3833	1.088			
NP-002606	0.138 ± 0.023	36707 ± 5257	1.028			
NP-002608	0.125 ± 0.008	37076 ± 2000	1.038			
NP-002616	0.119 ± 0.001	51488 ± 5949	1.442			
NP-002618	0.123 ± 0.005	43465 ± 1785	1.217			
NP-002626	0.114 ± 0.006	46824 ± 9172	1.311			

NP-002628	0.119 ± 0.006	38230 ± 4168	1.070			
NP-002633	0.122 ± 0.008	33851 ± 4114	0.948			
NP-002635	0.128 ± 0.002	43690 ± 2442	1.223			
NP-002642	0.123 ± 0.003	31246 ± 2388	0.875			
NP-002649	0.117 ± 0.005	50535 ± 8592	1.415			
NP-002650	0.125 ± 0.006	33468 ± 2753	0.937			
NP-002651	0.123 ± 0.001	31130 ± 1176	0.872			
NP-002653	0.125 ± 0.006	34410 ± 773	0.963			
NP-002655	0.123 ± 0.004	39497 ± 1962	1.106			
NP-002657	0.120 ± 0.005	56762 ± 2795	1.520			
NP-002658	0.122 ± 0.004	44491 ± 3816	1.246			
NP-002661	0.126 ± 0.004	51442 ± 3026	1.440			
NP-002662	0.117 ± 0.008	54205 ± 3259	1.518			
NP-002663	0.128 ± 0.004	59752 ± 7262	1.673			
NP-002664	0.108 ± 0.003	44808 ± 3935	1.255			
NP-002672	0.133 ± 0.012	46553 ± 5811	1.303			
NP-002673	0.115 ± 0.001	41743 ± 353	1.169			
NP-002675	0.138 ± 0.015	35486 ± 5715	0.994			
NP-002676	0.118 ± 0.005	40335 ± 4323	1.129			
NP-002678	0.111 ± 0.007	50537 ± 5116	1.415			
NP-002687	0.115 ± 0.005	29729 ± 2524	0.832			
NP-002688	0.118 ± 0.025	42715 ± 3428	1.196			
NP-002690	0.117 ± 0.002	35599 ± 1144	0.997			
NP-002695	0.120 ± 0.001	39904 ± 3993	1.117			
NP-002699	0.117 ± 0.006	48709 ± 8735	1.364			
NP-002700	0.109 ± 0.005	52369 ± 3792	1.466			
NP-002701	0.112 ± 0.006	42304 ± 4816	1.184			
NP-002702	0.115 ± 0.005	49369 ± 8669	1.382			
NP-002703	0.118 ± 0.034	60168 ± 10318	1.685			
NP-002708	0.112 ± 0.003	41820 ± 4564	1.171			
NP-002712	0.128 ± 0.009	38621 ± 662	1.081			
NP-002714	0.113 ± 0.004	50790 ± 1037	1.422			

NP-002719	0.113 ±0.005	38622 ±4568	1.081			
NP-002720	0.128 ±0.008	39675 ±2186	1.111			
NP-002724	0.109 ±0.003	35358 ±5009	0.990			
NP-002735	0.120 ±0.010	47903 ±5166	1.341			
NP-002736	0.124 ±0.005	52347 ±4264	1.466			
NP-002737	0.116 ±0.003	69131 ±6657	1.851			
NP-002738	0.111 ±0.001	39842 ±2126	1.116			
NP-002739	0.121 ±0.002	56945 ±4417	1.525			
NP-002740	0.122 ±0.003	46468 ±2037	1.301			
NP-002741	0.119 ±0.005	37383 ±848	1.047			
NP-002743	0.122 ±0.004	33916 ±2400	0.950			
NP-002749	0.119 ±0.005	39842 ±1587	1.116			
NP-002752	0.118 ±0.003	42689 ±2325	1.195			
NP-002754	0.126 ±0.010	46692 ±7414	1.307			
NP-002756	0.107 ±0.009	33794 ±4214	0.946			
NP-002766	0.115 ±0.003	53216 ±4566	1.425			
NP-002767	0.115 ±0.006	28612 ±8321	0.801			
NP-002768	0.124 ±0.005	31453 ±3414	0.881			
NP-002769	0.113 ±0.008	30153 ±2949	0.844			
NP-002776	0.120 ±0.005	44506 ±7375	1.246			
NP-002786	0.125 ±0.004	34736 ±2818	0.973			
NP-002789	0.114 ±0.002	58861 ±5377	1.648			
NP-002799	0.121 ±0.001	29472 ±2227	0.825			
NP-002802	0.110 ±0.006	34267 ±3985	0.959			
NP-002803	0.115 ±0.002	40929 ±3356	1.146			
NP-002804	0.119 ±0.002	44481 ±3016	1.245			
NP-002807	0.113 ±0.005	44776 ±3401	1.254			
NP-002808	0.119 ±0.001	35624 ±734	0.997			
NP-002809	0.120 ±0.003	38506 ±2127	1.078			
NP-002816	0.119 ±0.001	35357 ±3812	0.990			
NP-002829	0.116 ±0.002	27111 ±2042	0.759			
NP-002830	0.107 ±0.005	19200 ±860	0.514			

NP-002831	0.134 ± 0.019	20773 ± 1671	0.556			
NP-002832	0.110 ± 0.005	23637 ± 2935	0.662			
NP-002837	0.124 ± 0.004	41653 ± 4162	1.166			
NP-002840	0.114 ± 0.010	35624 ± 3010	0.997			
NP-002841	0.117 ± 0.003	60632 ± 6198	1.623			
NP-002852	0.124 ± 0.002	49292 ± 8272	1.380			
NP-002853	0.108 ± 0.009	51736 ± 5102	1.449			
NP-002854	0.114 ± 0.005	36797 ± 629	1.030			
NP-002856	0.105 ± 0.006	41014 ± 4878	1.148			
NP-002857	0.121 ± 0.002	28530 ± 353	0.799			
NP-002859	0.117 ± 0.007	58695 ± 5195	1.643			
NP-002860	0.116 ± 0.001	33444 ± 2196	0.936			
NP-002862	0.120 ± 0.005	37293 ± 1585	1.044			
NP-002863	0.115 ± 0.002	32530 ± 2129	0.911			
NP-002872	0.112 ± 0.002	38560 ± 5347	1.080			
NP-002873	0.109 ± 0.002	43092 ± 3322	1.207			
NP-002874	0.114 ± 0.001	43745 ± 486	1.225			
NP-002875	0.118 ± 0.003	34210 ± 2593	0.958			
NP-002876	0.112 ± 0.005	39585 ± 702	1.108			
NP-002881	0.119 ± 0.002	33042 ± 4087	0.925			
NP-002886	0.130 ± 0.009	68957 ± 10739	1.846			
NP-002890	0.123 ± 0.005	42450 ± 1475	1.189			
NP-002893	0.126 ± 0.005	24697 ± 6856	0.692			
NP-002894	0.125 ± 0.007	30538 ± 2755	0.855			
NP-002895	0.122 ± 0.001	52162 ± 3183	1.461			
NP-002896	0.125 ± 0.008	42557 ± 3030	1.192			
NP-002897	0.118 ± 0.012	37233 ± 5389	1.043			
NP-002898	0.11 ± 0.004	48591 ± 3104	1.361			
NP-002901	0.125 ± 0.003	11643 ± 869	0.312	0.122 ± 0.005	18570 ± 1302	0.568
NP-002912	0.120 ± 0.007	37520 ± 2411	1.051			
NP-002920	0.120 ± 0.002	28857 ± 1384	0.808			
NP-002922	0.125 ± 0.002	33870 ± 3830	0.948			

NP-002930	0.118 ± 0.005	40415 ± 2367	1.132			
NP-002940	0.110 ± 0.004	32571 ± 1421	0.912			
NP-002943	0.109 ± 0.011	33662 ± 2043	0.943			
NP-002944	0.111 ± 0.003	19946 ± 5141	0.534			
NP-002945	0.120 ± 0.004	58050 ± 6626	1.554			
NP-002946	0.119 ± 0.006	36761 ± 5160	1.029			
NP-002957	0.115 ± 0.002	34806 ± 650	0.975			
NP-002959	0.115 ± 0.004	36791 ± 2794	1.030			
NP-002961	0.110 ± 0.003	36014 ± 4524	1.008			
NP-002963	0.118 ± 0.003	43981 ± 2038	1.231			
NP-002965	0.118 ± 0.001	40942 ± 2258	1.146			
NP-002969	0.112 ± 0.005	40547 ± 2258	1.135			
NP-002970	0.125 ± 0.005	37746 ± 208	1.057			
NP-002973	0.112 ± 0.006	43646 ± 2359	1.222			
NP-002989	0.111 ± 0.006	32297 ± 3206	0.904			
NP-002999	0.121 ± 0.008	44881 ± 5249	1.257			
NP-003000	0.122 ± 0.009	54597 ± 6623	1.529			
NP-003001	0.111 ± 0.001	38102 ± 2242	1.067			
NP-003006	0.112 ± 0.005	36061 ± 2549	1.010			
NP-003011	0.117 ± 0.005	45269 ± 4456	1.268			
NP-003021	0.123 ± 0.008	37253 ± 2020	1.043			
NP-003024	0.116 ± 0.004	29878 ± 2372	0.837			
NP-003027	0.125 ± 0.004	29838 ± 2005	0.835			
NP-003030	0.113 ± 0.003	41373 ± 4697	1.158			
NP-003031	0.117 ± 0.009	33620 ± 2464	0.941			
NP-003037	0.118 ± 0.002	31214 ± 2267	0.874			
NP-003039	0.121 ± 0.005	34070 ± 1315	0.954			
NP-003040	0.121 ± 0.012	49280 ± 5108	1.380			
NP-003041	0.116 ± 0.004	38436 ± 6952	1.076			
NP-003055	0.097 ± 0.002 *					
NP-003057	0.078 ± 0.009 **					
NP-003062	0.118 ± 0.008	31876 ± 2085	0.893			

NP-003063	0.117 ±0.006	39143 ±2887	1.096			
NP-003067	0.126 ±0.005	54551 ±2889	1.461			
NP-003070	0.124 ±0.001	36363 ±9141	1.018			
NP-003071	0.112 ±0.002	34806 ±1985	0.975			
NP-003072	0.118 ±0.003	42412 ±5265	1.188			
NP-003075	0.128 ±0.005	32803 ±1944	0.918			
NP-003081	0.114 ±0.004	38140 ±2645	1.068			
NP-003082	0.118 ±0.006	37468 ±9722	1.049			
NP-003090	0.125 ±0.008	62833 ±1981	1.682			
NP-003094	0.125 ±0.006	47928 ±4130	1.342			
NP-003096	0.123 ±0.008	26979 ±3358	0.755			
NP-003101	0.114 ±0.002	32988 ±2386	0.924			
NP-003102	0.120 ±0.005	31972 ±2375	0.895			
NP-003105	0.112 ±0.008	38544 ±4843	1.079			
NP-003106	0.120 ±0.007	54739 ±8379	1.533			
NP-003108	0.123 ±0.003	39434 ±405	1.104			
NP-003110	0.120 ±0.002	40783 ±2800	1.142			
NP-003111	0.126 ±0.004	44971 ±2343	1.259			
NP-003123	0.115 ±0.004	29049 ±576	0.813			
NP-003124	0.119 ±0.003	33890 ±4053	0.949			
NP-003125	0.113 ±0.003	49364 ±9865	1.382			
NP-003127	0.120 ±0.010	38283 ±606	1.072			
NP-003128	0.113 ±0.004	45437 ±4488	1.272			
NP-003130	0.093 ±0.002 **					
NP-003134	0.115 ±0.005	58754 ±9692	1.645			
NP-003135	0.134 ±0.005	50503 ±789	1.414			
NP-003136	0.120 ±0.003	38821 ±1101	1.087			
NP-003138	0.123 ±0.004	33848 ±1143	0.948			
NP-003139	0.115 ±0.002	39145 ±1771	1.096			
NP-003143	0.117 ±0.004	34163 ±5537	0.957			
NP-003146	0.113 ±0.003	33700 ±1864	0.944			
NP-003153	0.115 ±0.005	44675 ±3260	1.251			

NP-003156	0.121 ±0.005	36916 ±2743	1.034			
NP-003164	0.113 ±0.004	47243 ±2634	1.323			
NP-003166	0.125 ±0.011	35175 ±2610	0.985			
NP-003180	0.112 ±0.003	30818 ±3883	0.863			
NP-003181	0.114 ±0.005	44940 ±3757	1.258			
NP-003186	0.114 ±0.004	37258 ±2985	1.043			
NP-003188	0.115 ±0.002	40778 ±1368	1.142			
NP-003190	0.123 ±0.004	45445 ±4963	1.272			
NP-003191	0.122 ±0.003	44074 ±1403	1.234			
NP-003195	0.110 ±0.004	50148 ±2293	1.404			
NP-003207	0.113 ±0.002	37068 ±5786	1.038			
NP-003216	0.127 ±0.006	32597 ±3701	0.913			
NP-003222	0.112 ±0.002	39963 ±2950	1.119			
NP-003223	0.115 ±0.006	36652 ±1425	1.026			
NP-003224	0.122 ±0.003	34484 ±1941	0.966			
NP-003225	0.115 ±0.004	25016 ±4137	0.700			
NP-003227	0.111 ±0.002	37454 ±5901	1.049			
NP-003235	0.116 ±0.001	20807 ±1624	0.583			
NP-003236	0.119 ±0.005	39019 ±6462	1.093			
NP-003240	0.119 ±0.003	34702 ±1391	0.972			
NP-003241	0.117 ±0.001	30586 ±1262	0.856			
NP-003242	0.119 ±0.005	35662 ±699	0.999			
NP-003244	0.116 ±0.003	50786 ±4504	1.422			
NP-003252	0.119 ±0.006	37557 ±2597	1.052			
NP-003253	0.119 ±0.008	19258 ±820	0.516			
NP-003256	0.119 ±0.003	31275 ±2226	0.876			
NP-003257	0.125 ±0.009	42957 ±5288	1.203			
NP-003262	0.117 ±0.003	58816 ±3572	1.575			
NP-003271	0.114 ±0.006	32085 ±2271	0.898			
NP-003273	0.092 ±0.007 **					
NP-003285	0.114 ±0.006	43121 ±2290	1.207			
NP-003288	0.116 ±0.007	38241 ±1702	1.071			

NP-003291	0.116 ±0.010	43938 ±6200	1.230			
NP-003296	0.121 ±0.006	32135 ±2331	0.900			
NP-003298	0.108 ±0.003	61516 ±8112	1.647			
NP-003299	0.108 ±0.007	130179 ±13136	3.486	0.115 ±0.001	32789 ±1619	1.003
NP-003301	0.114 ±0.006	30277 ±2724	0.848			
NP-003315	0.122 ±0.005	24823 ±2168	0.695			
NP-003316	0.113 ±0.005	50985 ±3304	1.428			
NP-003317	0.116 ±0.007	40380 ±2214	1.131			
NP-003319	0.115 ±0.006	38081 ±1430	1.066			
NP-003320	0.110 ±0.003	44456 ±5732	1.245			
NP-003321	0.110 ±0.003	55041 ±1680	1.474			
NP-003322	0.113 ±0.001	42772 ±1695	1.198			
NP-003325	0.116 ±0.002	24124 ±684	0.675			
NP-003331	0.115 ±0.006	51325 ±3166	1.437			
NP-003332	0.094 ±0.004 *					
NP-003337	0.114 ±0.007	38472 ±4344	1.077			
NP-003340	0.113 ±0.007	72467 ±8191	1.940			
NP-003341	0.115 ±0.009	71352 ±7438	1.910			
NP-003343	0.116 ±0.006	53808 ±8889	1.507			
NP-003344	0.117 ±0.001	51235 ±5736	1.435			
NP-003346	0.128 ±0.012	41268 ±5977	1.155			
NP-003347	0.119 ±0.008	43706 ±2464	1.224			
NP-003362	0.109 ±0.002	37988 ±1839	1.064			
NP-003366	0.112 ±0.001	28702 ±3636	0.804			
NP-003367	0.129 ±0.010	34954 ±1664	0.979			
NP-003370	0.130 ±0.009	37233 ±787	1.043			
NP-003375	0.111 ±0.014	44897 ±945	1.257			
NP-003376	0.114 ±0.003	58032 ±4041	1.554			
NP-003377	0.112 ±0.002	35758 ±1843	1.001			
NP-003378	0.112 ±0.003	33000 ±1709	0.924			
NP-003381	0.118 ±0.008	35243 ±2456	0.987			
NP-003387	0.114 ±0.002	39301 ±1191	1.100			

NP-003388	0.116 ±0.003	39477 ±1325	1.105			
NP-003389	0.112 ±0.002	19712 ±702	0.552			
NP-003390	0.122 ±0.005	25881 ±1360	0.725			
NP-003391	0.125 ±0.008	29631 ±2535	0.830			
NP-003392	0.114 ±0.004	36614 ±3858	1.025			
NP-003398	0.107 ±0.004	38846 ±1724	1.088			
NP-003400	0.118 ±0.005	38963 ±4286	1.091			
NP-003407	0.117 ±0.002	37648 ±4447	1.054			
NP-003411	0.117 ±0.002	33265 ±1747	0.931			
NP-003413	0.116 ±0.003	32347 ±611	0.906			
NP-003414	0.128 ±0.001	38080 ±529	1.066			
NP-003415	0.112 ±0.002	44621 ±6052	1.249			
NP-003423	0.115 ±0.003	31857 ±5817	0.892			
NP-003429	0.123 ±0.003	39552 ±6244	1.107			
NP-003430	0.114 ±0.004	45904 ±2530	1.285			
NP-003431	0.120 ±0.004	34845 ±2378	0.976			
NP-003432	0.113 ±0.008	36036 ±794	1.009			
NP-003433	0.122 ±0.004	29172 ±1130	0.817			
NP-003452	0.109 ±0.006	37648 ±4546	1.054			
NP-003453	0.118 ±0.009	43484 ±8134	1.218			
NP-003454	0.120 ±0.005	52664 ±6511	1.475			
NP-003457	0.126 ±0.006	39241 ±3125	1.099			
NP-003458	0.127 ±0.009	41558 ±5856	1.164			
NP-003462	0.103 ±0.006	37365 ±3940	1.046			
NP-003464	0.109 ±0.001	39228 ±2729	1.098			
NP-003465	0.119 ±0.006	42792 ±5840	1.198			
NP-003466	0.111 ±0.003	47660 ±1119	1.334			
NP-003468	0.114 ±0.007	36369 ±7538	1.018			
NP-003470	0.116 ±0.002	41216 ±3232	1.154			
NP-003472	0.111 ±0.002	36195 ±4852	1.013			
NP-003486	0.121 ±0.006	32489 ±1574	0.910			
NP-003489	0.117 ±0.004	36376 ±1355	1.019			

NP-003490	0.108 ± 0.002	48249 ± 6259	1.351			
NP-003491	0.117 ± 0.000	41079 ± 1339	1.150			
NP-003492	0.116 ± 0.003	43884 ± 671	1.229			
NP-003497	0.112 ± 0.005	45279 ± 7206	1.268			
NP-003501	0.119 ± 0.005	38122 ± 2661	1.067			
NP-003502	0.113 ± 0.004	39888 ± 3075	1.117			
NP-003511	0.112 ± 0.006	58535 ± 6043	1.567			
NP-003517	0.125 ± 0.019	31307 ± 1421	0.877			
NP-003518	0.123 ± 0.003	41823 ± 2199	1.171			
NP-003521	0.122 ± 0.006	37671 ± 932	1.055			
NP-003522	0.110 ± 0.006	42661 ± 3342	1.194			
NP-003524	0.112 ± 0.011	33066 ± 2740	0.926			
NP-003534	0.118 ± 0.005	31266 ± 235	0.875			
NP-003535	0.117 ± 0.002	44286 ± 2047	1.240			
NP-003536	0.126 ± 0.004	50356 ± 7413	1.410			
NP-003544	0.132 ± 0.016	66601 ± 435	1.783			
NP-003546	0.116 ± 0.004	141894 ± 6908	3.799	0.116 ± 0.007	35621 ± 4835	0.998
NP-003548	0.121 ± 0.006	49104 ± 5557	1.375			
NP-003550	0.112 ± 0.007	42981 ± 1893	1.203			
NP-003551	0.111 ± 0.013	47572 ± 3949	1.332			
NP-003552	0.116 ± 0.003	38154 ± 2401	1.068			
NP-003553	0.111 ± 0.002	38348 ± 3110	1.074			
NP-003555	0.124 ± 0.001	59540 ± 6266	1.594			
NP-003558	0.109 ± 0.003	46481 ± 6643	1.301			
NP-003562	0.116 ± 0.004	31780 ± 798	0.890			
NP-003563	0.116 ± 0.004	37090 ± 5966	1.038			
NP-003572	0.117 ± 0.006	44261 ± 8228	1.239			
NP-003578	0.111 ± 0.002	42143 ± 2689	1.180			
NP-003579	0.104 ± 0.006	34727 ± 5044	0.972			
NP-003584	0.120 ± 0.002	35844 ± 1899	1.004			
NP-003587	0.116 ± 0.003	39472 ± 5525	1.105			
NP-003588	0.117 ± 0.002	33996 ± 4264	0.952			

NP-003589	0.116 ±0.005	37540 ±1459	1.051			
NP-003594	0.112 ±0.002	71103 ±5693	1.904			
NP-003595	0.091 ±0.005 **					
NP-003600	0.121 ±0.002	38080 ±5134	1.066			
NP-003601	0.114 ±0.003	40194 ±2759	1.125			
NP-003602	0.122 ±0.005	40081 ±2805	1.122			
NP-003611	0.113 ±0.003	37979 ±1585	1.063			
NP-003613	0.116 ±0.004	50120 ±4303	1.403			
NP-003614	0.126 ±0.008	49707 ±6797	1.392			
NP-003617	0.116 ±0.007	43015 ±4384	1.204			
NP-003618	0.117 ±0.004	34432 ±2971	0.964			
NP-003619	0.124 ±0.003	39472 ±2473	1.105			
NP-003620	0.117 ±0.006	43083 ±2544	1.206			
NP-003624	0.123 ±0.004	38505 ±1162	1.078			
NP-003628	0.110 ±0.003	38284 ±8531	1.072			
NP-003630	0.108 ±0.005	39157 ±3435	1.096			
NP-003632	0.116 ±0.003	42159 ±3794	1.180			
NP-003636	0.115 ±0.007	37695 ±3562	1.055			
NP-003637	0.117 ±0.004	38083 ±3500	1.066			
NP-003638	0.114 ±0.003	51634 ±3197	1.446			
NP-003643	0.119 ±0.021	26143 ±3861	0.732			
NP-003644	0.114 ±0.004	48820 ±4032	1.367			
NP-003645	0.113 ±0.003	54608 ±4467	1.529			
NP-003646	0.118 ±0.006	51130 ±6865	1.432			
NP-003649	0.122 ±0.005	33718 ±2054	0.944			
NP-003657	0.114 ±0.008	43827 ±2929	1.227			
NP-003660	0.110 ±0.017	32141 ±3058	0.900			
NP-003665	0.115 ±0.001	24834 ±1769	0.695			
NP-003667	0.121 ±0.003	35161 ±1460	0.984			
NP-003668	0.118 ±0.002	23553 ±666	0.659			
NP-003669	0.122 ±0.003	51203 ±3307	1.434			
NP-003686	0.108 ±0.004	67606 ±1497	1.810			

NP-003691	0.119 ±0.005	39888 ±8315	1.117			
NP-003692	0.110 ±0.002	42398 ±1061	1.187			
NP-003696	0.112 ±0.004	67738 ±6208	1.814			
NP-003697	0.122 ±0.006	44218 ±3289	1.238			
NP-003701	0.119 ±0.004	60655 ±2704	1.624			
NP-003704	0.118 ±0.002	41389 ±5012	1.159			
NP-003720	0.127 ±0.004	46993 ±2624	1.316			
NP-003725	0.115 ±0.008	38044 ±4975	1.065			
NP-003727	0.111 ±0.007	55276 ±4357	1.548			
NP-003729	0.107 ±0.007	83619 ±2823	2.239	0.105 ±0.010	38172 ±2614	1.168
NP-003730	0.123 ±0.009	31261 ±3027	0.875			
NP-003731	0.113 ±0.009	26835 ±1062	0.751			
NP-003741	0.114 ±0.002	46140 ±7542	1.292			
NP-003742	0.110 ±0.003	33000 ±3012	0.924			
NP-003743	0.129 ±0.001	39831 ±1599	1.115			
NP-003750	0.120 ±0.004	47474 ±1700	1.329			
NP-003752	0.131 ±0.004	42180 ±1925	1.181			
NP-003758	0.115 ±0.003	52411 ±5189	1.467			
NP-003767	0.111 ±0.006	45635 ±2588	1.278			
NP-003773	0.108 ±0.002	48350 ±6908	1.354			
NP-003776	0.125 ±0.006	72590 ±8774	1.944			
NP-003787	0.112 ±0.005	68270 ±5523	1.828			
NP-003800	0.115 ±0.001	41050 ±4844	1.149			
NP-003804	0.121 ±0.006	52738 ±7226	1.477			
NP-003808	0.119 ±0.004	39863 ±517	1.116			
NP-003813	0.118 ±0.003	34913 ±3420	0.978			
NP-003819	0.115 ±0.006	27235 ±3581	0.763			
NP-003820	0.115 ±0.002	38495 ±2004	1.078			
NP-003823	0.116 ±0.005	72151 ±3665	1.932			
NP-003824	0.113 ±0.005	38712 ±1264	1.084			
NP-003826	0.113 ±0.002	46083 ±1380	1.290			
NP-003829	0.116 ±0.001	36449 ±1598	1.021			

NP-003831	0.110 ± 0.004	38119 ± 10217	1.067			
NP-003833	0.115 ± 0.004	39249 ± 3278	1.099			
NP-003841	0.120 ± 0.004	59258 ± 4235	1.587			
NP-003845	0.114 ± 0.003	37930 ± 1375	1.062			
NP-003852	0.116 ± 0.006	65538 ± 4075	1.755			
NP-003855	0.121 ± 0.013	60472 ± 4138	1.619			
NP-003859	0.113 ± 0.001	45450 ± 3528	1.273			
NP-003860	0.115 ± 0.004	50576 ± 5866	1.416			
NP-003861	0.114 ± 0.003	35675 ± 1654	0.999			
NP-003862	0.124 ± 0.006	43105 ± 1709	1.207			
NP-003864	0.111 ± 0.004	26018 ± 2055	0.728			
NP-003869	0.120 ± 0.003	53136 ± 1972	1.423			
NP-003871	0.114 ± 0.003	35455 ± 340	0.993			
NP-003872	0.112 ± 0.006	41378 ± 9184	1.159			
NP-003879	0.129 ± 0.007	49944 ± 7041	1.398			
NP-003881	0.129 ± 0.005	29563 ± 1191	0.828			
NP-003886	0.111 ± 0.002	54560 ± 3823	1.528			
NP-003887	0.109 ± 0.005	28995 ± 2383	0.812			
NP-003889	0.115 ± 0.004	32536 ± 2996	0.911			
NP-003890	0.109 ± 0.005	38163 ± 1282	1.069			
NP-003891	0.119 ± 0.010	38307 ± 3589	1.073			
NP-003892	0.108 ± 0.005	31239 ± 2088	0.875			
NP-003895	0.131 ± 0.007	55777 ± 8398	1.562			
NP-003902	0.120 ± 0.008	40885 ± 930	1.145			
NP-003906	0.116 ± 0.005	59610 ± 920	1.596			
NP-003909	0.107 ± 0.004	57252 ± 2160	1.533			
NP-003917	0.128 ± 0.006	23178 ± 1927	0.649			
NP-003918	0.111 ± 0.001	36877 ± 790	1.033			
NP-003920	0.112 ± 0.003	45267 ± 1857	1.267			
NP-003922	0.116 ± 0.001	34229 ± 992	0.958			
NP-003927	0.11 ± 0.001	36742 ± 1799	1.029			
NP-003939	0.124 ± 0.006	39914 ± 2375	1.118			

NP-003940	0.115 ±0.009	40356 ±2721	1.130			
NP-003942	0.115 ±0.005	31634 ±5497	0.886			
NP-003952	0.111 ±0.010	46313 ±9007	1.297			
NP-003955	0.120 ±0.005	43652 ±5043	1.222			
NP-003960	0.116 ±0.004	40690 ±4191	1.139			
NP-003962	0.126 ±0.005	36692 ±1234	1.027			
NP-003965	0.116 ±0.008	48036 ±1145	1.345			
NP-003970	0.115 ±0.005	39591 ±3490	1.109			
NP-003974	0.115 ±0.005	33185 ±2048	0.929			
NP-003976	0.116 ±0.003	33428 ±822	0.936			
NP-003977	0.115 ±0.001	41115 ±3258	1.151			
NP-003978	0.111 ±0.001	35714 ±5361	1.000			
NP-004005	0.124 ±0.007	53736 ±6066	1.505			
NP-004011	0.122 ±0.010	19302 ±440	0.517			
NP-004015	0.124 ±0.005	60054 ±9076	1.681			
NP-004017	0.125 ±0.005	38436 ±1349	1.076			
NP-004019	0.116 ±0.003	38006 ±6580	1.064			
NP-004021	0.115 ±0.001	28064 ±1297	0.786			
NP-004022	0.118 ±0.002	37637 ±1067	1.054			
NP-004024	0.118 ±0.005	45245 ±1132	1.267			
NP-004025	0.114 ±0.002	40738 ±1675	1.141			
NP-004029	0.115 ±0.004	50332 ±9005	1.409			
NP-004031	0.114 ±0.004	57839 ±3726	1.619			
NP-004034	0.118 ±0.003	38867 ±2813	1.088			
NP-004035	0.121 ±0.002	30102 ±360	0.843			
NP-004037	0.107 ±0.005	39787 ±3553	1.114			
NP-004041	0.114 ±0.006	34257 ±2173	0.959			
NP-004042	0.114 ±0.002	31598 ±1998	0.885			
NP-004045	0.128 ±0.008	58179 ±3785	1.558			
NP-004046	0.125 ±0.008	27598 ±6163	0.773			
NP-004047	0.117 ±0.007	50937 ±8345	1.426			
NP-004052	0.116 ±0.007	38033 ±6901	1.065			

NP-004061	0.116 ± 0.003	37026 ± 3270	1.037			
NP-004074	0.111 ± 0.004	36410 ± 4546	1.019			
NP-004088	0.113 ± 0.006	32104 ± 5817	0.899			
NP-004089	0.112 ± 0.003	37572 ± 4843	1.052			
NP-004093	0.131 ± 0.004	36568 ± 1033	1.024			
NP-004095	0.127 ± 0.009	31631 ± 1010	0.886			
NP-004099	0.108 ± 0.003	55034 ± 6599	1.541			
NP-004101	0.119 ± 0.006	47102 ± 3401	1.319			
NP-004108	0.114 ± 0.001	29563 ± 1003	0.828			
NP-004110	0.110 ± 0.009	37408 ± 2470	1.047			
NP-004121	0.114 ± 0.005	39382 ± 5210	1.103			
NP-004128	0.113 ± 0.006	48788 ± 5565	1.366			
NP-004138	0.131 ± 0.009	31057 ± 2045	0.870			
NP-004144	0.120 ± 0.005	46685 ± 4070	1.307			
NP-004148	0.112 ± 0.006	44288 ± 4719	1.240			
NP-004154	0.122 ± 0.007	35633 ± 1405	0.998			
NP-004184	0.122 ± 0.005	50243 ± 7221	1.407			
NP-004193	0.123 ± 0.008	43764 ± 2821	1.225			
NP-004194	0.123 ± 0.007	47529 ± 1461	1.331			
NP-004202	0.131 ± 0.013	36769 ± 3933	1.030			
NP-004204	0.117 ± 0.003	37032 ± 1856	1.037			
NP-004206	0.115 ± 0.002	47784 ± 2792	1.338			
NP-004210	0.133 ± 0.017	46345 ± 6687	1.298			
NP-004213	0.119 ± 0.002	41433 ± 3163	1.160			
NP-004214	0.117 ± 0.003	37705 ± 648	1.056			
NP-004218	0.113 ± 0.004	56135 ± 5749	1.572			
NP-004221	0.123 ± 0.004	64799 ± 4758	1.735			
NP-004231	0.116 ± 0.002	29861 ± 1782	0.836			
NP-004232	0.116 ± 0.007	60324 ± 8471	1.689			
NP-004246	0.123 ± 0.002	49508 ± 4418	1.386			
NP-004247	0.130 ± 0.009	31176 ± 935	0.873			
NP-004248	0.113 ± 0.003	31082 ± 1815	0.870			

NP-004255	0.128 ±0.005	60109 ±5668	1.609			
NP-004263	0.116 ±0.002	38423 ±3484	1.076			
NP-004266	0.113 ±0.001	32434 ±1860	0.908			
NP-004271	0.109 ±0.006	43829 ±2566	1.227			
NP-004273	0.116 ±0.003	40843 ±9821	1.144			
NP-004274	0.111 ±0.006	31930 ±6696	0.894			
NP-004275	0.112 ±0.004	36441 ±4613	1.020			
NP-004278	0.118 ±0.007	34905 ±4640	0.977			
NP-004281	0.117 ±0.002	42977 ±3385	1.203			
NP-004283	0.125 ±0.003	40089 ±2890	1.122			
NP-004284	0.116 ±0.004	51194 ±11639	1.433			
NP-004286	0.118 ±0.009	35375 ±9310	0.990			
NP-004290	0.122 ±0.005	34196 ±2518	0.957			
NP-004301	0.118 ±0.004	40500 ±172	1.134			
NP-004305	0.117 ±0.002	23113 ±968	0.647			
NP-004306	0.114 ±0.003	41490 ±7917	1.162			
NP-004313	0.112 ±0.016	36284 ±3263	1.016			
NP-004321	0.116 ±0.004	46837 ±3008	1.311			
NP-004323	0.114 ±0.005	29112 ±1053	0.815			
NP-004332	0.132 ±0.006	26054 ±6523	0.729			
NP-004338	0.119 ±0.012	27298 ±4052	0.764			
NP-004339	0.117 ±0.006	45721 ±1932	1.280			
NP-004347	0.115 ±0.003	27412 ±1304	0.768			
NP-004350	0.123 ±0.003	33867 ±2026	0.948			
NP-004351	0.118 ±0.002	39986 ±6344	1.120			
NP-004355	0.116 ±0.015	42774 ±7378	1.198			
NP-004363	0.127 ±0.004	26574 ±1008	0.744			
NP-004365	0.114 ±0.006	24464 ±2381	0.685			
NP-004366	0.114 ±0.006	27621 ±2010	0.773			
NP-004367	0.112 ±0.003	12903 ±247	0.345	0.114 ±0.007	28647 ±1359	0.877
NP-004369	0.107 ±0.005	12734 ±1351	0.341	0.113 ±0.003	20029 ±508	0.613
NP-004379	0.123 ±0.007	40983 ±961	1.148			

NP-004380	0.112 ±0.016	40882 ±6682	1.145			
NP-004381	0.112 ±0.005	40382 ±2248	1.131			
NP-004382	0.116 ±0.005	34354 ±7597	0.962			
NP-004387	0.113 ±0.004	42931 ±2177	1.202			
NP-004391	0.115 ±0.005	48923 ±7726	1.370			
NP-004404	0.113 ±0.001	44906 ±4312	1.257			
NP-004405	0.117 ±0.009	51310 ±3257	1.437			
NP-004406	0.134 ±0.018	53803 ±7850	1.506			
NP-004409	0.114 ±0.003	48765 ±1703	1.365			
NP-004413	0.116 ±0.003	39386 ±910	1.103			
NP-004414	0.119 ±0.010	31844 ±9089	0.892			
NP-004416	0.112 ±0.002	48824 ±3098	1.367			
NP-004419	0.115 ±0.004	38284 ±3959	1.072			
NP-004420	0.125 ±0.002	58670 ±4398	1.643			
NP-004421	0.108 ±0.001	66680 ±7482	1.867			
NP-004423	0.120 ±0.002	38141 ±4758	1.068			
NP-004425	0.117 ±0.002	69969 ±3732	1.873			
NP-004427	0.120 ±0.004	34286 ±1955	0.960			
NP-004428	0.111 ±0.007	56510 ±5467	1.582			
NP-004429	0.109 ±0.004	48497 ±1360	1.358			
NP-004430	0.112 ±0.002	36147 ±1706	1.012			
NP-004431	0.123 ±0.006	23064 ±862	0.646			
NP-004432	0.112 ±0.003	45482 ±3480	1.273			
NP-004438	0.114 ±0.006	43649 ±1635	1.222			
NP-004442	0.125 ±0.006	37779 ±303	1.058			
NP-004446	0.110 ±0.001	36009 ±1480	1.008			
NP-004451	0.105 ±0.008	66597 ±3840	1.783			
NP-004452	0.130 ±0.012	39624 ±2404	1.109			
NP-004455	0.113 ±0.002	31837 ±1725	0.891			
NP-004456	0.111 ±0.002	44726 ±577	1.252			
NP-004459	0.115 ±0.005	33271 ±1504	0.932			
NP-004466	0.121 ±0.005	44556 ±5738	1.248			

NP-004485	0.120 ± 0.008	44087 ± 3638	1.234			
NP-004493	0.088 ± 0.009 **					
NP-004495	0.113 ± 0.003	45923 ± 8742	1.286			
NP-004497	0.118 ± 0.007	41321 ± 1612	1.157			
NP-004499	0.118 ± 0.004	56180 ± 8424	1.504			
NP-004500	0.116 ± 0.006	37180 ± 4590	1.041			
NP-004504	0.120 ± 0.006	26700 ± 906	0.748			
NP-004508	0.122 ± 0.013	33127 ± 2106	0.928			
NP-004509	0.118 ± 0.003	33374 ± 1603	0.934			
NP-004524	0.116 ± 0.003	63424 ± 4625	1.698			
NP-004527	0.114 ± 0.001	45489 ± 6945	1.274			
NP-004529	0.112 ± 0.001	34274 ± 7312	0.960			
NP-004536	0.116 ± 0.003	72069 ± 8577	1.930			
NP-004543	0.122 ± 0.006	38627 ± 3477	1.082			
NP-004545	0.113 ± 0.003	34861 ± 1596	0.976			
NP-004549	0.117 ± 0.005	33057 ± 2051	0.926			
NP-004550	0.112 ± 0.005	37121 ± 7605	1.039			
NP-004559	0.122 ± 0.005	52781 ± 7895	1.478			
NP-004560	0.118 ± 0.004	39610 ± 3379	1.109			
NP-004561	0.117 ± 0.007	43263 ± 1366	1.211			
NP-004565	0.121 ± 0.004	47962 ± 1783	1.343			
NP-004567	0.117 ± 0.004	49717 ± 5600	1.392			
NP-004571	0.121 ± 0.003	40242 ± 3990	1.127			
NP-004573	0.125 ± 0.006	48089 ± 4132	1.346			
NP-004574	0.122 ± 0.002	44924 ± 870	1.258			
NP-004575	0.106 ± 0.001	38665 ± 2055	1.083			
NP-004576	0.118 ± 0.005	34752 ± 2084	0.973			
NP-004577	0.118 ± 0.005	45309 ± 2006	1.269			
NP-004585	0.112 ± 0.003	31594 ± 4753	0.885			
NP-004586	0.125 ± 0.003	30700 ± 2592	0.860			
NP-004593	0.115 ± 0.004	43003 ± 7773	1.204			
NP-004597	0.118 ± 0.004	32272 ± 2017	0.904			

NP-004604	0.087 ±0.003 **					
NP-004605	0.098 ±0.015	19960 ±1024	0.534			
NP-004611	0.118 ±0.004	34314 ±676	0.961			
NP-004612	0.126 ±0.006	41036 ±5301	1.149			
NP-004614	0.123 ±0.003	38351 ±2675	1.074			
NP-004621	0.117 ±0.002	32250 ±3005	0.903			
NP-004633	0.125 ±0.005	32032 ±1316	0.897			
NP-004636	0.121 ±0.010	22547 ±2552	0.631			
NP-004646	0.132 ±0.006	42424 ±1110	1.188			
NP-004647	0.115 ±0.005	37767 ±1266	1.057			
NP-004661	0.133 ±0.008	28852 ±615	0.808			
NP-004662	0.122 ±0.001	53456 ±5072	1.497			
NP-004668	0.116 ±0.001	33947 ±2287	0.950			
NP-004669	0.116 ±0.004	32985 ±178	0.924			
NP-004670	0.123 ±0.007	43216 ±1748	1.210			
NP-004674	0.117 ±0.006	43853 ±1563	1.228			
NP-004675	0.127 ±0.007	49043 ±1937	1.373			
NP-004676	0.120 ±0.003	48319 ±4349	1.353			
NP-004682	0.123 ±0.002	41233 ±2662	1.155			
NP-004687	0.108 ±0.005	36105 ±5062	1.011			
NP-004705	0.131 ±0.014	27836 ±1063	0.779			
NP-004713	0.122 ±0.003	32385 ±5112	0.907			
NP-004714	0.125 ±0.010	35216 ±2287	0.986			
NP-004715	0.115 ±0.001	34599 ±5888	0.969			
NP-004720	0.121 ±0.003	39052 ±3186	1.093			
NP-004721	0.113 ±0.004	44012 ±4837	1.232			
NP-004753	0.119 ±0.007	18694 ±814	0.501			
NP-004754	0.119 ±0.013	25291 ±3610	0.708			
NP-004757	0.120 ±0.009	38492 ±5672	1.078			
NP-004761	0.110 ±0.002	53221 ±9534	1.490			
NP-004762	0.115 ±0.004	50444 ±7093	1.412			
NP-004763	0.116 ±0.002	40243 ±899	1.127			

NP-004768	0.115 ±0.007	58476 ±5296	1.637			
NP-004769	0.126 ±0.004	55941 ±4261	1.566			
NP-004779	0.120 ±0.003	31968 ±1334	0.895			
NP-004781	0.131 ±0.013	40233 ±5675	1.127			
NP-004782	0.111 ±0.007	34127 ±1966	0.956			
NP-004783	0.111 ±0.005	52780 ±419	1.413			
NP-004788	0.114 ±0.004	39924 ±5955	1.118			
NP-004790	0.119 ±0.001	41439 ±3244	1.160			
NP-004792	0.113 ±0.004	35067 ±2595	0.982			
NP-004794	0.126 ±0.005	40433 ±3902	1.132			
NP-004802	0.115 ±0.003	41437 ±2601	1.160			
NP-004808	0.111 ±0.002	50838 ±2707	1.423			
NP-004810	0.112 ±0.003	35671 ±1339	0.999			
NP-004811	0.117 ±0.003	38523 ±2673	1.079			
NP-004813	0.115 ±0.002	29862 ±2356	0.836			
NP-004815	0.121 ±0.006	35827 ±5525	1.003			
NP-004825	0.112 ±0.002	35481 ±9876	0.993			
NP-004826	0.116 ±0.006	19497 ±2858	0.522			
NP-004833	0.111 ±0.009	33491 ±1520	0.938			
NP-004835	0.115 ±0.004	34465 ±936	0.965			
NP-004838	0.111 ±0.004	52604 ±1844	1.473			
NP-004848	0.123 ±0.008	34810 ±2104	0.975			
NP-004852	0.119 ±0.002	63350 ±5081	1.696			
NP-004853	0.115 ±0.005	67361 ±6103	1.804			
NP-004858	0.120 ±0.003	31968 ±5771	0.895			
NP-004865	0.115 ±0.001	33879 ±1591	0.949			
NP-004871	0.115 ±0.008	45127 ±3838	1.264			
NP-004872	0.135 ±0.005	32343 ±1959	0.906			
NP-004879	0.113 ±0.003	37888 ±2338	1.061			
NP-004882	0.109 ±0.003	43521 ±676	1.219			
NP-004889	0.121 ±0.009	40325 ±2441	1.129			
NP-004891	0.115 ±0.007	46589 ±2698	1.304			

NP-004914	0.125 ± 0.004	38098 ± 2544	1.067			
NP-004917	0.111 ± 0.002	49703 ± 3404	1.392			
NP-004918	0.117 ± 0.004	32934 ± 653	0.922			
NP-004931	0.121 ± 0.008	24733 ± 993	0.693			
NP-004935	0.117 ± 0.001	32863 ± 3077	0.920			
NP-004936	0.110 ± 0.003	34548 ± 630	0.967			
NP-004937	0.120 ± 0.005	49671 ± 5040	1.391			
NP-004949	0.113 ± 0.004	38440 ± 351	1.076			
NP-004950	0.109 ± 0.003	40289 ± 4163	1.128			
NP-004954	0.116 ± 0.004	33780 ± 3042	0.946			
NP-004961	0.117 ± 0.006	45235 ± 2326	1.267			
NP-004966	0.119 ± 0.001	50210 ± 2563	1.406			
NP-004969	0.127 ± 0.009	37619 ± 6711	1.053			
NP-004972	0.108 ± 0.006	38526 ± 831	1.079			
NP-004973	0.136 ± 0.014	32638 ± 4749	0.914			
NP-004975	0.110 ± 0.006	34293 ± 1952	0.960			
NP-004979	0.117 ± 0.002	48026 ± 5763	1.345			
NP-004980	0.125 ± 0.002	44109 ± 3768	1.235			
NP-004981	0.110 ± 0.004	53326 ± 5251	1.493			
NP-004993	0.111 ± 0.005	44545 ± 2607	1.247			
NP-004999	0.124 ± 0.005	50621 ± 3315	1.417			
NP-005001	0.113 ± 0.003	39871 ± 1866	1.116			
NP-005003	0.115 ± 0.004	41019 ± 2545	1.149			
NP-005004	0.117 ± 0.003	42332 ± 2380	1.185			
NP-005009	0.113 ± 0.006	47772 ± 6963	1.338			
NP-005012	0.121 ± 0.004	46224 ± 7271	1.294			
NP-005022	0.120 ± 0.006	34292 ± 1243	0.960			
NP-005023	0.116 ± 0.002	39176 ± 6391	1.097			
NP-005029	0.117 ± 0.002	39052 ± 960	1.093			
NP-005034	0.122 ± 0.001	36418 ± 2202	1.020			
NP-005036	0.121 ± 0.003	39948 ± 1699	1.119			
NP-005040	0.120 ± 0.001	33010 ± 2171	0.924			

NP-005047	0.136 ± 0.006	36430 ± 1338	1.020			
NP-005049	0.119 ± 0.003	45793 ± 2225	1.282			
NP-005051	0.127 ± 0.008	61523 ± 6341	1.723			
NP-005052	0.118 ± 0.002	30615 ± 1955	0.857			
NP-005054	0.111 ± 0.002	41150 ± 5237	1.152			
NP-005058	0.121 ± 0.004	65836 ± 4353	1.763			
NP-005059	0.113 ± 0.004	31329 ± 1569	0.877			
NP-005062	0.116 ± 0.004	46849 ± 7082	1.312			
NP-005064	0.121 ± 0.002	43301 ± 1986	1.212			
NP-005068	0.106 ± 0.012	33564 ± 4862	0.940			
NP-005070	0.125 ± 0.003	38024 ± 1258	1.065			
NP-005076	0.118 ± 0.018	36528 ± 972	1.023			
NP-005086	0.115 ± 0.007	36785 ± 3143	1.030			
NP-005088	0.125 ± 0.005	36570 ± 1581	1.024			
NP-005092	0.110 ± 0.006	37157 ± 3317	1.040			
NP-005095	0.110 ± 0.002	40380 ± 1501	1.131			
NP-005096	0.121 ± 0.002	46802 ± 1203	1.310			
NP-005097	0.120 ± 0.003	29600 ± 1856	0.829			
NP-005098	0.118 ± 0.004	39480 ± 3435	1.105			
NP-005099	0.114 ± 0.004	42282 ± 4820	1.184			
NP-005103	0.120 ± 0.001	70613 ± 5897	1.891			
NP-005104	0.113 ± 0.008	51406 ± 3356	1.439			
NP-005106	0.124 ± 0.008	40372 ± 2936	1.130			
NP-005107	0.120 ± 0.005	60060 ± 3667	1.608			
NP-005109	0.114 ± 0.004	35246 ± 338	0.987			
NP-005110	0.114 ± 0.003	40155 ± 1681	1.124			
NP-005114	0.116 ± 0.008	65640 ± 7220	1.758			
NP-005115	0.105 ± 0.002	43222 ± 2861	1.210			
NP-005117	0.120 ± 0.007	50753 ± 2417	1.421			
NP-005118	0.110 ± 0.004	50971 ± 8713	1.427			
NP-005119	0.108 ± 0.002	36352 ± 7158	1.018			
NP-005126	0.110 ± 0.004	32555 ± 1059	0.912			

NP-005127	0.113 ±0.004	51895 ±7170	1.453			
NP-005128	0.114 ±0.002	34163 ±3202	0.957			
NP-005129	0.113 ±0.004	41290 ±1637	1.156			
NP-005132	0.120 ±0.003	56631 ±3125	1.516			
NP-005136	0.112 ±0.006	38398 ±4912	1.075			
NP-005142	0.112 ±0.002	52710 ±1803	1.476			
NP-005143	0.122 ±0.010	31195 ±429	0.873			
NP-005147	0.115 ±0.001	31542 ±1621	0.883			
NP-005155	0.116 ±0.007	43671 ±1217	1.223			
NP-005157	0.123 ±0.002	42987 ±954	1.204			
NP-005159	0.110 ±0.005	37652 ±2989	1.054			
NP-005161	0.114 ±0.006	66297 ±4177	1.775			
NP-005162	0.119 ±0.003	44011 ±4249	1.232			
NP-005173	0.122 ±0.005	38466 ±2723	1.077			
NP-005183	0.115 ±0.004	49037 ±1931	1.373			
NP-005192	0.121 ±0.003	35248 ±2682	0.987			
NP-005206	0.118 ±0.002	55456 ±3387	1.553			
NP-005212	0.117 ±0.003	48018 ±1947	1.344			
NP-005213	0.119 ±0.001	36545 ±2283	1.023			
NP-005214	0.122 ±0.006	39918 ±2149	1.118			
NP-005215	0.125 ±0.005	34874 ±381	0.976			
NP-005217	0.119 ±0.004	42652 ±884	1.194			
NP-005218	0.121 ±0.004	34301 ±8690	0.960			
NP-005220	0.119 ±0.003	36048 ±5685	1.009			
NP-005221	0.112 ±0.004	49937 ±3196	1.398			
NP-005233	0.118 ±0.003	58217 ±3902	1.559			
NP-005236	0.103 ±0.007	44952 ±1919	1.259			
NP-005239	0.117 ±0.003	46692 ±2669	1.307			
NP-005240	0.123 ±0.002	54572 ±3149	1.528			
NP-005241	0.118 ±0.014	28011 ±4965	0.784			
NP-005248	0.121 ±0.005	29775 ±2479	0.834			
NP-005252	0.115 ±0.006	30553 ±740	0.855			

NP-005258	0.111 ±0.002	43340 ±9039	1.213			
NP-005260	0.122 ±0.003	45576 ±4300	1.276			
NP-005265	0.114 ±0.002	41761 ±1462	1.169			
NP-005271	0.119 ±0.006	46776 ±4109	1.310			
NP-005273	0.096 ±0.002 *					
NP-005280	0.112 ±0.006	40127 ±3023	1.124			
NP-005281	0.118 ±0.007	38522 ±2042	1.079			
NP-005283	0.113 ±0.001	35279 ±8640	0.988			
NP-005288	0.123 ±0.002	31637 ±1820	0.886			
NP-005298	0.118 ±0.001	37557 ±1359	1.052			
NP-005301	0.111 ±0.000	47802 ±851	1.338			
NP-005304	0.115 ±0.009	33018 ±2553	0.924			
NP-005305	0.118 ±0.006	45806 ±4865	1.283			
NP-005306	0.117 ±0.009	38643 ±1660	1.082			
NP-005308	0.124 ±0.003	33033 ±2718	0.925			
NP-005313	0.116 ±0.007	41711 ±1694	1.168			
NP-005320	0.115 ±0.005	38122 ±4528	1.067			
NP-005323	0.120 ±0.010	22923 ±1266	0.642			
NP-005326	0.112 ±0.011	44545 ±4723	1.247			
NP-005328	0.116 ±0.002	29839 ±1490	0.835			
NP-005331	0.111 ±0.008	23238 ±1155	0.651			
NP-005332	0.109 ±0.002	36222 ±1235	1.014			
NP-005334	0.120 ±0.009	48425 ±3279	1.356			
NP-005337	0.115 ±0.005	47249 ±3720	1.323			
NP-005339	0.114 ±0.002	62778 ±5041	1.681			
NP-005340	0.126 ±0.010	43016 ±2626	1.204			
NP-005342	0.117 ±0.003	45648 ±3217	1.278			
NP-005344	0.121 ±0.004	53086 ±2847	1.486			
NP-005359	0.116 ±0.002	39665 ±1222	1.111			
NP-005366	0.117 ±0.005	29159 ±1444	0.816			
NP-005369	0.119 ±0.002	47108 ±3787	1.319			
NP-005373	0.113 ±0.002	35779 ±1617	1.002			

NP-005376	0.121 ±0.024	34483 ±1330	0.966			
NP-005377	0.126 ±0.007	40433 ±1856	1.132			
NP-005379	0.114 ±0.003	37871 ±333	1.060			
NP-005382	0.120 ±0.003	50550 ±4748	1.415			
NP-005394	0.125 ±0.003	36714 ±3684	1.028			
NP-005397	0.116 ±0.003	51276 ±9672	1.436			
NP-005399	0.136 ±0.012	31313 ±440	0.877			
NP-005402	0.114 ±0.005	28355 ±4362	0.794			
NP-005403	0.115 ±0.001	32041 ±2166	0.897			
NP-005404	0.119 ±0.012	29754 ±2181	0.833			
NP-005405	0.115 ±0.005	42020 ±1352	1.177			
NP-005410	0.119 ±0.002	68489 ±9329	1.834			
NP-005418	0.112 ±0.007	25480 ±8447	0.713			
NP-005429	0.117 ±0.002	49495 ±1227	1.386			
NP-005438	0.120 ±0.002	36781 ±2304	1.030			
NP-005439	0.109 ±0.002	42044 ±2643	1.177			
NP-005442	0.125 ±0.006	49853 ±8096	1.396			
NP-005444	0.125 ±0.007	54837 ±7708	1.535			
NP-005445	0.114 ±0.003	40368 ±3619	1.130			
NP-005446	0.121 ±0.002	45458 ±2455	1.273			
NP-005455	0.118 ±0.001	39021 ±1501	1.093			
NP-005457	0.112 ±0.004	45374 ±4735	1.270			
NP-005460	0.096 ±0.003 *					
NP-005462	0.117 ±0.006	53701 ±3390	1.504			
NP-005464	0.110 ±0.005	45506 ±4353	1.274			
NP-005480	0.110 ±0.003	46842 ±4971	1.312			
NP-005491	0.110 ±0.001	30695 ±1929	0.859			
NP-005498	0.121 ±0.005	37725 ±1666	1.056			
NP-005501	0.117 ±0.006	38394 ±1250	1.075			
NP-005504	0.113 ±0.001	34126 ±9250	0.956			
NP-005508	0.133 ±0.008	45208 ±1343	1.266			
NP-005513	0.110 ±0.004	27585 ±2466	0.772			

NP-005515	0.114 ±0.004	34550 ±1050	0.967			
NP-005516	0.114 ±0.003	35033 ±1381	0.981			
NP-005517	0.110 ±0.001	35357 ±1587	0.990			
NP-005518	0.125 ±0.005	40118 ±363	1.123			
NP-005519	0.123 ±0.008	31186 ±1014	0.873			
NP-005520	0.118 ±0.005	35620 ±1264	0.997			
NP-005522	0.112 ±0.006	31365 ±2302	0.878			
NP-005534	0.114 ±0.001	40327 ±1269	1.129			
NP-005546	0.126 ±0.008	45938 ±9551	1.286			
NP-005548	0.118 ±0.003	37742 ±5181	1.057			
NP-005551	0.124 ±0.007	45726 ±5651	1.280			
NP-005552	0.119 ±0.001	42824 ±2090	1.199			
NP-005559	0.130 ±0.007	28469 ±1947	0.797			
NP-005562	0.119 ±0.003	39388 ±5488	1.103			
NP-005563	0.117 ±0.003	48511 ±1623	1.358			
NP-005566	0.112 ±0.002	44766 ±4955	1.253			
NP-005567	0.112 ±0.006	31890 ±1583	0.893			
NP-005579	0.124 ±0.006	54201 ±7897	1.518			
NP-005582	0.114 ±0.006	39154 ±1350	1.096			
NP-005584	0.115 ±0.003	39288 ±5075	1.100			
NP-005586	0.134 ±0.004	45868 ±4090	1.284			
NP-005587	0.119 ±0.002	39585 ±5019	1.108			
NP-005607	0.133 ±0.010	43160 ±3871	1.208			
NP-005609	0.115 ±0.005	46146 ±2711	1.292			
NP-005610	0.117 ±0.021	45527 ±2936	1.275			
NP-005611	0.129 ±0.007	26886 ±1340	0.753			
NP-005613	0.112 ±0.008	65047 ±3664	1.742			
NP-005624	0.114 ±0.003	37513 ±1657	1.050			
NP-005626	0.107 ±0.010	25132 ±2615	0.704			
NP-005634	0.127 ±0.006	31232 ±2059	0.874			
NP-005635	0.119 ±0.001	19084 ±377	0.511			
NP-005637	0.117 ±0.004	34602 ±5490	0.969			

NP-005644	0.111 ±0.004	35464 ±2287	0.993			
NP-005647	0.114 ±0.004	37352 ±3951	1.046			
NP-005650	0.116 ±0.003	36353 ±1459	1.018			
NP-005651	0.115 ±0.010	50275 ±4989	1.408			
NP-005654	0.112 ±0.007	45809 ±1432	1.283			
NP-005655	0.123 ±0.004	33036 ±1324	0.925			
NP-005656	0.117 ±0.001	33750 ±758	0.945			
NP-005657	0.119 ±0.005	40134 ±2764	1.124			
NP-005666	0.124 ±0.006	36558 ±2252	1.024			
NP-005668	0.120 ±0.007	35999 ±4154	1.008			
NP-005690	0.115 ±0.007	28911 ±2510	0.809			
NP-005697	0.117 ±0.002	44160 ±2636	1.236			
NP-005700	0.107 ±0.007	50743 ±5043	1.421			
NP-005704	0.118 ±0.005	39946 ±788	1.118			
NP-005707	0.106 ±0.007	39266 ±2102	1.099			
NP-005709	0.123 ±0.001	32522 ±1874	0.911			
NP-005716	0.138 ±0.021	49663 ±4714	1.391			
NP-005725	0.129 ±0.009	41792 ±3812	1.170			
NP-005727	0.114 ±0.004	35641 ±620	0.998			
NP-005729	0.112 ±0.011	66848 ±10669	1.790			
NP-005751	0.116 ±0.016	34467 ±476	0.965			
NP-005752	0.124 ±0.010	42224 ±717	1.182			
NP-005755	0.121 ±0.003	37185 ±839	1.041			
NP-005756	0.116 ±0.003	53710 ±2819	1.504			
NP-005759	0.123 ±0.007	31825 ±6226	0.891			
NP-005776	0.112 ±0.005	31458 ±4323	0.881			
NP-005777	0.127 ±0.005	35489 ±4615	0.994			
NP-005779	0.118 ±0.003	29663 ±2218	0.831			
NP-005785	0.126 ±0.004	46099 ±6995	1.291			
NP-005787	0.111 ±0.007	47079 ±2960	1.318			
NP-005789	0.114 ±0.009	32122 ±5049	0.899			
NP-005796	0.129 ±0.002	46828 ±1617	1.311			

NP-005798	0.116 ± 0.003	45070 ± 4803	1.262			
NP-005801	0.124 ± 0.004	48441 ± 3367	1.356			
NP-005805	0.119 ± 0.006	40418 ± 4578	1.132			
NP-005807	0.118 ± 0.004	49769 ± 1295	1.394			
NP-005808	0.116 ± 0.003	52001 ± 1614	1.456			
NP-005809	0.130 ± 0.009	31218 ± 3225	0.874			
NP-005810	0.114 ± 0.001	71199 ± 10336	1.906			
NP-005818	0.117 ± 0.005	34846 ± 5814	0.976			
NP-005821	0.116 ± 0.006	56344 ± 3611	1.509			
NP-005822	0.116 ± 0.006	20194 ± 1111	0.541			
NP-005823	0.111 ± 0.008	24630 ± 2179	0.690			
NP-005827	0.111 ± 0.003	42781 ± 5953	1.198			
NP-005828	0.119 ± 0.002	43370 ± 2363	1.214			
NP-005831	0.117 ± 0.004	33806 ± 2940	0.947			
NP-005836	0.128 ± 0.020	47401 ± 7724	1.327			
NP-005840	0.117 ± 0.004	55254 ± 3870	1.479			
NP-005841	0.111 ± 0.001	28990 ± 1813	0.812			
NP-005843	0.117 ± 0.005	49250 ± 7505	1.379			
NP-005845	0.115 ± 0.003	32386 ± 3046	0.907			
NP-005847	0.121 ± 0.003	39849 ± 3242	1.116			
NP-005852	0.124 ± 0.007	40849 ± 1617	1.144			
NP-005854	0.117 ± 0.003	31716 ± 691	0.888			
NP-005855	0.119 ± 0.001	43224 ± 4998	1.210			
NP-005857	0.129 ± 0.002	38167 ± 9385	1.069			
NP-005872	0.119 ± 0.004	27796 ± 997	0.778			
NP-005892	0.111 ± 0.007	38573 ± 1752	1.080			
NP-005896	0.123 ± 0.003	40702 ± 3811	1.140			
NP-005898	0.119 ± 0.006	47766 ± 3836	1.337			
NP-005901	0.110 ± 0.003	39765 ± 1742	1.113			
NP-005902	0.117 ± 0.008	39803 ± 2492	1.114			
NP-005904	0.117 ± 0.001	36071 ± 3157	1.010			
NP-005916	0.118 ± 0.008	60848 ± 1702	1.629			

NP-005923	0.126 ±0.008	40495 ±4096	1.134			
NP-005926	0.121 ±0.003	45842 ±1438	1.284			
NP-005931	0.125 ±0.007	30460 ±355	0.853			
NP-005932	0.109 ±0.005	34878 ±3586	0.977			
NP-005938	0.119 ±0.001	57060 ±569	1.528			
NP-005946	0.114 ±0.001	33156 ±1052	0.928			
NP-005953	0.110 ±0.003	55408 ±1499	1.484			
NP-005954	0.118 ±0.007	47255 ±1208	1.323			
NP-005955	0.118 ±0.004	50947 ±8603	1.426			
NP-005957	0.111 ±0.001	35004 ±5579	0.980			
NP-005966	0.111 ±0.006	41960 ±2888	1.175			
NP-005967	0.117 ±0.003	38448 ±1430	1.077			
NP-005968	0.114 ±0.003	46063 ±6583	1.290			
NP-005970	0.121 ±0.005	36435 ±4424	1.020			
NP-005976	0.118 ±0.004	63735 ±10072	1.785			
NP-005981	0.119 ±0.006	41566 ±3707	1.164			
NP-005982	0.113 ±0.003	37012 ±1027	1.036			
NP-005999	0.125 ±0.002	34493 ±2892	0.966			
NP-006000	0.114 ±0.003	42189 ±5247	1.181			
NP-006001	0.126 ±0.005	39874 ±2875	1.116			
NP-006002	0.110 ±0.005	43188 ±4418	1.209			
NP-006003	0.114 ±0.019	42682 ±8970	1.195			
NP-006021	0.115 ±0.006	68972 ±5125	1.847			
NP-006028	0.118 ±0.002	27856 ±1411	0.780			
NP-006032	0.118 ±0.004	37545 ±2231	1.051			
NP-006033	0.126 ±0.012	48852 ±2953	1.368			
NP-006036	0.116 ±0.005	40547 ±5968	1.135			
NP-006037	0.121 ±0.007	50272 ±2193	1.408			
NP-006038	0.111 ±0.003	27688 ±4234	0.775			
NP-006039	0.127 ±0.005	42909 ±2421	1.201			
NP-006040	0.125 ±0.003	51009 ±2341	1.428			
NP-006043	0.123 ±0.006	42915 ±3619	1.202			

NP-006048	0.127 ± 0.012	31128 ± 659	0.872			
NP-006067	0.108 ± 0.003	43925 ± 1891	1.230			
NP-006068	0.130 ± 0.005	36053 ± 4345	1.009			
NP-006069	0.120 ± 0.003	46341 ± 4527	1.298			
NP-006070	0.123 ± 0.001	45276 ± 2817	1.268			
NP-006071	0.117 ± 0.006	46872 ± 2477	1.312			
NP-006079	0.118 ± 0.011	31487 ± 5086	0.882			
NP-006089	0.123 ± 0.010	36382 ± 894	1.019			
NP-006098	0.114 ± 0.001	50044 ± 2376	1.401			
NP-006099	0.124 ± 0.005	40543 ± 2727	1.135			
NP-006102	0.123 ± 0.006	37565 ± 5143	1.052			
NP-006103	0.114 ± 0.003	45960 ± 1353	1.287			
NP-006105	0.116 ± 0.001	36564 ± 7730	1.024			
NP-006106	0.120 ± 0.004	56845 ± 2740	1.522			
NP-006109	0.122 ± 0.004	48748 ± 3223	1.365			
NP-006110	0.114 ± 0.001	52580 ± 3574	1.472			
NP-006112	0.124 ± 0.007	31044 ± 4139	0.869			
NP-006128	0.117 ± 0.001	41214 ± 3670	1.154			
NP-006130	0.115 ± 0.000	60113 ± 6936	1.610			
NP-006135	0.121 ± 0.002	36196 ± 3527	1.013			
NP-006143	0.114 ± 0.003	36775 ± 529	1.030			
NP-006144	0.115 ± 0.003	37222 ± 4082	1.042			
NP-006145	0.118 ± 0.001	40043 ± 6198	1.121			
NP-006148	0.117 ± 0.006	31272 ± 1805	0.876			
NP-006159	0.107 ± 0.004	41675 ± 3311	1.167			
NP-006160	0.121 ± 0.008	50406 ± 6297	1.411			
NP-006161	0.122 ± 0.006	40677 ± 1450	1.139			
NP-006163	0.117 ± 0.008	39775 ± 1584	1.114			
NP-006165	0.117 ± 0.003	32464 ± 3606	0.909			
NP-006169	0.118 ± 0.003	61967 ± 4358	1.659			
NP-006173	0.114 ± 0.004	44162 ± 3439	1.237			
NP-006174	0.114 ± 0.003	40445 ± 7910	1.132			

NP-006175	0.122 ± 0.004	35180 ± 2479	0.985			
NP-006183	0.116 ± 0.003	34899 ± 9755	0.977			
NP-006184	0.108 ± 0.010	30427 ± 2260	0.852			
NP-006189	0.126 ± 0.008	26291 ± 6057	0.736			
NP-006195	0.120 ± 0.002	35810 ± 1308	1.003			
NP-006200	0.113 ± 0.009	42823 ± 2216	1.199			
NP-006205	0.112 ± 0.002	34103 ± 1533	0.955			
NP-006215	0.122 ± 0.008	33158 ± 3607	0.928			
NP-006222	0.126 ± 0.002	42093 ± 2756	1.179			
NP-006234	0.123 ± 0.004	46527 ± 3662	1.303			
NP-006249	0.117 ± 0.010	35633 ± 1139	0.998			
NP-006269	0.111 ± 0.003	41641 ± 6491	1.166			
NP-006276	0.120 ± 0.002	35772 ± 1073	1.002			
NP-006288	0.119 ± 0.004	31888 ± 643	0.893			
NP-006291	0.118 ± 0.005	31796 ± 1939	0.890			
NP-006292	0.121 ± 0.007	47664 ± 3732	1.335			
NP-006293	0.111 ± 0.003	33334 ± 7952	0.933			
NP-006295	0.118 ± 0.003	35584 ± 1557	0.996			
NP-006296	0.121 ± 0.004	45893 ± 4128	1.285			
NP-006298	0.116 ± 0.005	26934 ± 945	0.754			
NP-006299	0.110 ± 0.007	31299 ± 1604	0.876			
NP-006304	0.115 ± 0.006	49807 ± 4679	1.395			
NP-006308	0.115 ± 0.003	38277 ± 3252	1.072			
NP-006316	0.123 ± 0.003	41439 ± 3842	1.160			
NP-006322	0.105 ± 0.012	47080 ± 4590	1.318			
NP-006324	0.108 ± 0.007	52847 ± 3936	1.480			
NP-006325	0.104 ± 0.011	30046 ± 3572	0.841			
NP-006326	0.119 ± 0.009	32010 ± 3023	0.896			
NP-006330	0.123 ± 0.001	39664 ± 1791	1.111			
NP-006331	0.111 ± 0.004	28576 ± 1482	0.800			
NP-006332	0.110 ± 0.002	31785 ± 5013	0.890			
NP-006336	0.122 ± 0.002	44229 ± 4060	1.238			

NP-006355	0.118 ±0.003	46404 ±5347	1.299			
NP-006358	0.122 ±0.015	35333 ±2350	0.989			
NP-006363	0.114 ±0.010	35052 ±3568	0.981			
NP-006370	0.114 ±0.003	46979 ±6836	1.315			
NP-006371	0.126 ±0.006	53007 ±5370	1.484			
NP-006372	0.115 ±0.007	39530 ±3802	1.107			
NP-006378	0.129 ±0.010	32837 ±2483	0.919			
NP-006380	0.119 ±0.003	44153 ±520	1.236			
NP-006392	0.111 ±0.004	34940 ±4215	0.978			
NP-006394	0.118 ±0.004	36471 ±3064	1.021			
NP-006400	0.114 ±0.005	39370 ±4081	1.102			
NP-006410	0.114 ±0.001	39988 ±5756	1.120			
NP-006412	0.119 ±0.005	26771 ±503	0.750			
NP-006419	0.113 ±0.004	54718 ±7567	1.532			
NP-006421	0.116 ±0.001	31823 ±850	0.891			
NP-006423	0.115 ±0.009	62518 ±6274	1.674			
NP-006429	0.128 ±0.005	70272 ±6850	1.882			
NP-006438	0.120 ±0.004	39987 ±764	1.120			
NP-006440	0.116 ±0.009	36514 ±1833	1.022			
NP-006442	0.128 ±0.003	27275 ±614	0.764			
NP-006443	0.108 ±0.003	26528 ±1697	0.743			
NP-006444	0.113 ±0.004	53214 ±3850	1.490			
NP-006447	0.112 ±0.002	50059 ±5057	1.402			
NP-006452	0.115 ±0.009	45991 ±2552	1.288			
NP-006453	0.121 ±0.008	36338 ±2116	1.017			
NP-006454	0.118 ±0.008	29691 ±1468	0.831			
NP-006458	0.120 ±0.003	38688 ±1945	1.083			
NP-006459	0.116 ±0.012	40685 ±5269	1.139			
NP-006462	0.115 ±0.002	36649 ±6709	1.026			
NP-006464	0.122 ±0.005	45011 ±6500	1.260			
NP-006465	0.127 ±0.008	29092 ±526	0.815			
NP-006473	0.118 ±0.009	36441 ±1819	1.020			

NP-006475	0.127 ±0.012	28948 ±923	0.811			
NP-006477	0.115 ±0.005	40521 ±2260	1.135			
NP-006485	0.117 ±0.009	94521 ±3202	2.531	0.12 ±0.016	106997 ±9389	3.274
NP-006488	0.124 ±0.006	37199 ±3129	1.042			
NP-006495	0.124 ±0.008	40595 ±2640	1.137			
NP-006496	0.113 ±0.001	23459 ±1812	0.657			
NP-006497	0.118 ±0.004	39643 ±3278	1.110			
NP-006498	0.122 ±0.006	34399 ±1765	0.963			
NP-006503	0.126 ±0.023	31704 ±5949	0.888			
NP-006504	0.127 ±0.011	36135 ±889	1.012			
NP-006507	0.125 ±0.007	31423 ±2367	0.880			
NP-006521	0.118 ±0.004	28201 ±2427	0.790			
NP-006522	0.122 ±0.006	52310 ±3543	1.465			
NP-006524	0.109 ±0.005	42891 ±2628	1.201			
NP-006530	0.122 ±0.006	32596 ±4126	0.913			
NP-006531	0.116 ±0.006	29384 ±2072	0.823			
NP-006532	0.117 ±0.001	35505 ±3046	0.994			
NP-006534	0.114 ±0.003	39218 ±3401	1.098			
NP-006538	0.091 ±0.002 *					
NP-006553	0.114 ±0.003	41814 ±4199	1.171			
NP-006565	0.113 ±0.006	40008 ±2656	1.120			
NP-006566	0.121 ±0.006	42662 ±1108	1.195			
NP-006568	0.118 ±0.003	55978 ±7077	1.567			
NP-006572	0.124 ±0.006	30778 ±1541	0.862			
NP-006574	0.120 ±0.002	46684 ±3555	1.307			
NP-006576	0.121 ±0.008	38120 ±1982	1.067			
NP-006580	0.113 ±0.021	37730 ±4830	1.056			
NP-006581	0.116 ±0.006	29693 ±2320	0.831			
NP-006582	0.120 ±0.003	36396 ±3369	1.019			
NP-006583	0.115 ±0.005	24391 ±5175	0.683			
NP-006584	0.127 ±0.004	33509 ±2424	0.938			
NP-006585	0.126 ±0.005	33862 ±1996	0.948			

NP-006586	0.125 ±0.007	43607 ±5556	1.221			
NP-006587	0.115 ±0.005	33060 ±3807	0.926			
NP-006591	0.093 ±0.009 *					
NP-006592	0.109 ±0.004	41192 ±4101	1.153			
NP-006593	0.112 ±0.003	32723 ±2573	0.916			
NP-006595	0.109 ±0.001	38401 ±5277	1.075			
NP-006596	0.112 ±0.004	33294 ±4844	0.932			
NP-006597	0.118 ±0.003	42868 ±4215	1.200			
NP-006598	0.117 ±0.003	40470 ±990	1.133			
NP-006602	0.120 ±0.005	35646 ±7703	0.998			
NP-006608	0.119 ±0.006	43130 ±3676	1.208			
NP-006616	0.113 ±0.002	37734 ±1293	1.057			
NP-006617	0.119 ±0.001	64935 ±3683	1.739			
NP-006620	0.118 ±0.004	42770 ±1473	1.198			
NP-006621	0.117 ±0.006	37925 ±515	1.062			
NP-006624	0.117 ±0.003	43661 ±4636	1.222			
NP-006626	0.137 ±0.008	22334 ±1289	0.598			
NP-006633	0.113 ±0.002	43423 ±5592	1.216			
NP-006637	0.122 ±0.009	34410 ±1032	0.963			
NP-006641	0.124 ±0.005	45148 ±3231	1.264			
NP-006643	0.105 ±0.001	35896 ±1083	1.005			
NP-006654	0.109 ±0.002	49041 ±2603	1.373			
NP-006661	0.120 ±0.002	44720 ±2466	1.252			
NP-006671	0.121 ±0.013	31939 ±1021	0.894			
NP-006673	0.109 ±0.012	38258 ±3486	1.071			
NP-006679	0.117 ±0.003	35480 ±2823	0.993			
NP-006680	0.121 ±0.003	50488 ±1088	1.414			
NP-006686	0.120 ±0.002	44567 ±5929	1.248			
NP-006692	0.112 ±0.017	37375 ±3997	1.046			
NP-006693	0.124 ±0.003	32496 ±2019	0.910			
NP-006695	0.117 ±0.013	41354 ±4446	1.158			
NP-006696	0.120 ±0.004	38077 ±2592	1.066			

NP-006702	0.109 ± 0.003	34778 ± 1017	0.974			
NP-006714	0.119 ± 0.004	29339 ± 1074	0.821			
NP-006715	0.111 ± 0.004	46576 ± 4151	1.304			
NP-006720	0.117 ± 0.010	37064 ± 4172	1.038			
NP-006721	0.118 ± 0.001	39184 ± 3784	1.097			
NP-006724	0.130 ± 0.011	46695 ± 2777	1.307			
NP-006725	0.118 ± 0.005	35619 ± 663	0.997			
NP-006726	0.113 ± 0.003	34887 ± 3189	0.977			
NP-006728	0.122 ± 0.004	35669 ± 2424	0.999			
NP-006732	0.112 ± 0.005	40509 ± 553	1.134			
NP-006733	0.115 ± 0.004	50171 ± 3330	1.405			
NP-006741	0.126 ± 0.009	51503 ± 5870	1.442			
NP-006754	0.133 ± 0.005	41568 ± 1572	1.164			
NP-006757	0.121 ± 0.003	50047 ± 3594	1.401			
NP-006759	0.113 ± 0.004	41427 ± 4421	1.160			
NP-006760	0.129 ± 0.008	39295 ± 2291	1.100			
NP-006762	0.127 ± 0.012	39323 ± 3116	1.101			
NP-006763	0.111 ± 0.006	40032 ± 2871	1.121			
NP-006766	0.112 ± 0.005	45550 ± 3522	1.275			
NP-006767	0.117 ± 0.002	44879 ± 8167	1.257			
NP-006768	0.124 ± 0.003	21731 ± 5522	0.608			
NP-006769	0.119 ± 0.003	45022 ± 777	1.261			
NP-006770	0.122 ± 0.002	25481 ± 2387	0.713			
NP-006771	0.123 ± 0.002	23206 ± 997	0.650			
NP-006775	0.114 ± 0.002	39910 ± 8680	1.117			
NP-006776	0.127 ± 0.009	35987 ± 1331	1.008			
NP-006780	0.117 ± 0.003	42594 ± 8269	1.193			
NP-006783	0.119 ± 0.002	32663 ± 1855	0.915			
NP-006784	0.130 ± 0.008	38576 ± 2443	1.080			
NP-006788	0.131 ± 0.008	38308 ± 4344	1.073			
NP-006799	0.120 ± 0.004	39217 ± 1066	1.098			
NP-006800	0.112 ± 0.004	39219 ± 637	1.098			

NP-006801	0.119 ±0.001	30477 ±1172	0.853			
NP-006802	0.118 ±0.004	54631 ±3338	1.530			
NP-006807	0.116 ±0.004	44053 ±4213	1.233			
NP-006811	0.119 ±0.001	48093 ±1992	1.347			
NP-006813	0.115 ±0.002	38849 ±7594	1.088			
NP-006817	0.120 ±0.009	34668 ±2453	0.971			
NP-006819	0.117 ±0.006	34672 ±1877	0.971			
NP-006842	0.117 ±0.006	47307 ±3056	1.325			
NP-006843	0.120 ±0.004	54551 ±3133	1.461			
NP-006852	0.136 ±0.018	39423 ±3367	1.104			
NP-006860	0.109 ±0.004	48572 ±2176	1.360			
NP-006862	0.116 ±0.006	32936 ±1766	0.922			
NP-006863	0.109 ±0.013	21869 ±2572	0.612			
NP-006868	0.110 ±0.006	30561 ±206	0.856			
NP-006870	0.121 ±0.002	38366 ±2538	1.074			
NP-006871	0.113 ±0.004	55072 ±6528	1.542			
NP-006873	0.111 ±0.002	39153 ±7264	1.096			
NP-006878	0.117 ±0.005	55543 ±4020	1.555			
NP-006881	0.116 ±0.003	42661 ±2216	1.194			
NP-006884	0.113 ±0.011	32935 ±5044	0.922			
NP-006886	0.120 ±0.011	33719 ±3038	0.944			
NP-006899	0.121 ±0.003	33043 ±2129	0.925			
NP-006901	0.117 ±0.005	52370 ±7156	1.466			
NP-006902	0.127 ±0.009	33651 ±2969	0.942			
NP-006903	0.116 ±0.004	43489 ±6237	1.218			
NP-006904	0.113 ±0.002	61788 ±3393	1.654			
NP-006907	0.114 ±0.001	36753 ±2674	1.029			
NP-006913	0.113 ±0.004	30231 ±2151	0.846			
NP-006914	0.110 ±0.006	40760 ±1163	1.141			
NP-006921	0.128 ±0.004	44046 ±3778	1.233			
NP-006923	0.118 ±0.002	30075 ±2326	0.842			
NP-006926	0.117 ±0.004	40504 ±2048	1.134			

NP-006929	0.115 ±0.003	25643 ±5086	0.718			
NP-006931	0.116 ±0.005	53336 ±2665	1.493			
NP-006932	0.129 ±0.003	24772 ±2867	0.694			
NP-006933	0.138 ±0.009	30782 ±2335	0.862			
NP-006934	0.116 ±0.002	44203 ±1437	1.238			
NP-006935	0.119 ±0.011	53549 ±5476	1.499			
NP-006945	0.120 ±0.002	33000 ±4508	0.924			
NP-006946	0.123 ±0.007	41084 ±7701	1.150			
NP-006947	0.118 ±0.004	32813 ±5683	0.919			
NP-006949	0.125 ±0.005	36761 ±2400	1.029			
NP-006950	0.123 ±0.001	41448 ±5219	1.161			
NP-006952	0.120 ±0.004	38165 ±3719	1.069			
NP-006953	0.121 ±0.002	41873 ±3222	1.172			
NP-006956	0.111 ±0.013	33698 ±2077	0.944			
NP-006965	0.120 ±0.004	31228 ±2584	0.874			
NP-006979	0.127 ±0.011	39148 ±1447	1.096			
NP-006983	0.114 ±0.008	50925 ±7761	1.426			
NP-006988	0.119 ±0.002	42944 ±808	1.202			
NP-006989	0.120 ±0.004	40344 ±1832	1.130			
NP-006990	0.122 ±0.006	42163 ±6375	1.181			
NP-006991	0.116 ±0.004	38859 ±7043	1.088			
NP-006992	0.116 ±0.003	51323 ±11086	1.437			
NP-006995	0.118 ±0.003	33106 ±1385	0.927			
NP-007002	0.112 ±0.007	29642 ±2678	0.830			
NP-007007	0.117 ±0.007	36731 ±2503	1.028			
NP-007010	0.114 ±0.014	27563 ±3531	0.772			
NP-007011	0.118 ±0.001	44494 ±5519	1.246			
NP-007015	0.109 ±0.015	43289 ±3071	1.212			
NP-007016	0.115 ±0.003	28388 ±1341	0.795			
NP-007017	0.115 ±0.004	24234 ±2111	0.679			
NP-007020	0.111 ±0.004	32987 ±6098	0.924			
NP-007023	0.124 ±0.006	27342 ±946	0.766			

NP-007024	0.117 ±0.003	46223 ±3456	1.294			
NP-007029	0.112 ±0.004	60804 ±4625	1.628			
NP-007031	0.119 ±0.003	32644 ±3466	0.914			
NP-007042	0.117 ±0.007	26263 ±557	0.735			
NP-007045	0.109 ±0.001	39790 ±2708	1.114			
NP-007050	0.114 ±0.003	37453 ±2705	1.049			
NP-007053	0.116 ±0.002	35399 ±3924	0.991			
NP-007057	0.117 ±0.005	46016 ±1954	1.288			
NP-007060	0.120 ±0.005	31376 ±2647	0.879			
NP-007064	0.119 ±0.003	25952 ±2347	0.727			
NP-007072	0.122 ±0.005	42749 ±1164	1.197			
NP-007073	0.120 ±0.002	46266 ±2924	1.295			
NP-007075	0.124 ±0.007	33047 ±4086	0.925			
NP-007081	0.127 ±0.008	19125 ±3044	0.512			
NP-007087	0.119 ±0.007	41811 ±2458	1.171			
NP-007088	0.114 ±0.004	39404 ±4293	1.103			
NP-007093	0.133 ±0.014	19601 ±584	0.525			
NP-007094	0.115 ±0.004	25436 ±2646	0.712			
NP-007095	0.106 ±0.005	19892 ±3536	0.533			
NP-007098	0.138 ±0.006	13006 ±641	0.348	0.126 ±0.016	20029 ±1701	0.613
NP-007101	0.116 ±0.004	35136 ±3347	0.984			
NP-007105	0.120 ±0.002	39642 ±2683	1.110			
NP-007109	0.112 ±0.005	31148 ±9492	0.872			
NP-007111	0.125 ±0.004	48761 ±5189	1.365			
NP-007112	0.117 ±0.002	35757 ±4113	1.001			
NP-007114	0.113 ±0.004	30603 ±1471	0.857			
NP-007115	0.113 ±0.014	19740 ±1282	0.529			
NP-007117	0.118 ±0.002	44598 ±1642	1.249			
NP-007118	0.124 ±0.007	53178 ±3307	1.489			
NP-007120	0.122 ±0.009	33873 ±970	0.948			
NP-007121	0.123 ±0.006	35678 ±1232	0.999			
NP-007126	0.115 ±0.003	28232 ±1386	0.790			

NP-007133	0.111 ± 0.001	44402 ± 2187	1.243			
NP-007134	0.121 ± 0.009	46340 ± 4371	1.297			
NP-007136	0.121 ± 0.002	29920 ± 1900	0.838			
NP-007147	0.113 ± 0.005	33473 ± 1510	0.937			
NP-007148	0.121 ± 0.017	41840 ± 3608	1.171			
NP-007153	0.125 ± 0.009	35732 ± 376	1.000			
NP-007154	0.120 ± 0.008	38812 ± 2581	1.087			
NP-007158	0.105 ± 0.003	41624 ± 6224	1.165			
NP-007161	0.114 ± 0.003	49790 ± 6205	1.394			
NP-007166	0.119 ± 0.007	40073 ± 2244	1.122			
NP-007167	0.115 ± 0.022	32021 ± 2766	0.897			
NP-007169	0.120 ± 0.003	39868 ± 1924	1.116			
NP-007170	0.118 ± 0.001	29037 ± 2186	0.813			
NP-007175	0.126 ± 0.001	39456 ± 5358	1.105			
NP-007179	0.110 ± 0.002	45289 ± 7182	1.268			
NP-007180	0.130 ± 0.005	39043 ± 2955	1.093			
NP-007181	0.129 ± 0.008	70408 ± 591	1.885			
NP-007183	0.118 ± 0.005	37647 ± 1670	1.054			
NP-007184	0.118 ± 0.004	33263 ± 2242	0.931			
NP-007187	0.114 ± 0.017	33547 ± 7762	0.939			
NP-007198	0.114 ± 0.008	41880 ± 7980	1.173			
NP-007200	0.116 ± 0.004	48840 ± 3606	1.367			
NP-007202	0.096 ± 0.003 *					
NP-007208	0.115 ± 0.002	32078 ± 2975	0.898			
NP-007209	0.111 ± 0.005	40425 ± 6467	1.132			
NP-007210	0.112 ± 0.001	48125 ± 8853	1.347			
NP-007212	0.117 ± 0.006	34857 ± 1523	0.976			
NP-007213	0.125 ± 0.000	51545 ± 785	1.443			
NP-007217	0.115 ± 0.007	35383 ± 1453	0.991			
NP-007218	0.115 ± 0.004	47672 ± 2912	1.335			
NP-007227	0.111 ± 0.001	48484 ± 2348	1.358			
NP-007231	0.121 ± 0.006	39066 ± 1307	1.094			

NP-007232	0.111 ±0.006	39135 ±2767	1.096			
NP-007233	0.115 ±0.001	57725 ±9366	1.616			
NP-007234	0.114 ±0.013	39707 ±7841	1.112			
NP-007240	0.116 ±0.017	45910 ±4464	1.285			
NP-007243	0.114 ±0.006	41438 ±6936	1.160			
NP-007249	0.116 ±0.007	33010 ±1777	0.924			
NP-007254	0.115 ±0.003	29644 ±1085	0.830			
NP-007256	0.110 ±0.003	35132 ±2139	0.984			
NP-007257	0.125 ±0.012	26256 ±663	0.735			
NP-007258	0.121 ±0.005	37637 ±3152	1.054			
NP-007259	0.123 ±0.003	36414 ±2895	1.020			
NP-007260	0.122 ±0.008	50325 ±4374	1.409			
NP-007263	0.112 ±0.001	45981 ±3016	1.287			
NP-007267	0.112 ±0.007	38721 ±978	1.084			
NP-007268	0.117 ±0.002	31639 ±1962	0.886			
NP-007270	0.117 ±0.002	34561 ±3500	0.968			
NP-007271	0.134 ±0.007	48514 ±7484	1.358			
NP-007283	0.111 ±0.006	34271 ±2471	0.960			
NP-007284	0.114 ±0.003	43637 ±3982	1.222			
NP-007285	0.115 ±0.002	40491 ±1573	1.134			
NP-007288	0.115 ±0.008	44472 ±2207	1.245			
NP-007289	0.123 ±0.005	42854 ±8854	1.200			
NP-007291	0.123 ±0.001	35239 ±899	0.987			
NP-007297	0.124 ±0.009	47000 ±6630	1.316			
NP-007299	0.124 ±0.005	52851 ±2012	1.480			
NP-007300	0.109 ±0.002	41673 ±3499	1.167			
NP-007301	0.109 ±0.005	32177 ±1439	0.901			
NP-007305	0.110 ±0.005	37430 ±1901	1.048			
NP-007310	0.124 ±0.005	38913 ±4547	1.090			
NP-007313	0.114 ±0.006	28477 ±1919	0.797			
NP-007316	0.119 ±0.005	47533 ±3531	1.331			
NP-007320	0.124 ±0.007	28841 ±1837	0.808			

NP-007323	0.107 ±0.003	46401 ±4792	1.299			
NP-007324	0.126 ±0.006	35530 ±1575	0.995			
NP-007327	0.115 ±0.002	39082 ±3020	1.094			
NP-007329	0.116 ±0.003	35911 ±2842	1.005			
NP-007334	0.117 ±0.006	48878 ±1189	1.369			
NP-007336	0.111 ±0.002	31501 ±1213	0.882			
NP-007341	0.117 ±0.001	21207 ±2093	0.594			
NP-007346	0.116 ±0.001	32001 ±1798	0.896			
NP-007347	0.118 ±0.006	38843 ±7818	1.088			
NP-007350	0.135 ±0.013	45748 ±421	1.281			
NP-007353	0.124 ±0.002	27353 ±2557	0.766			
NP-007356	0.118 ±0.005	34445 ±1523	0.964			
NP-007357	0.113 ±0.001	40906 ±1074	1.145			
NP-007358	0.115 ±0.003	39907 ±6219	1.117			
NP-007365	0.122 ±0.005	55224 ±2922	1.479			
NP-007366	0.122 ±0.010	46188 ±5074	1.293			
NP-007367	0.132 ±0.014	38473 ±939	1.077			
NP-007368	0.115 ±0.005	37811 ±705	1.059			
NP-007372	0.116 ±0.006	48536 ±6265	1.359			
NP-007374	0.123 ±0.003	37086 ±3301	1.038			
NP-007375	0.115 ±0.006	57406 ±6203	1.537			
NP-007377	0.136 ±0.011	35266 ±1081	0.987			
NP-007383	0.122 ±0.014	32617 ±3841	0.913			
NP-007385	0.119 ±0.007	50626 ±5804	1.417			
NP-007386	0.110 ±0.006	33851 ±1234	0.948			
NP-007388	0.117 ±0.003	34185 ±6297	0.957			
NP-007389	0.115 ±0.003	31933 ±1524	0.894			
NP-007391	0.123 ±0.005	37305 ±1406	1.045			
NP-007393	0.122 ±0.005	35737 ±499	1.001			
NP-007394	0.117 ±0.005	31751 ±3230	0.889			
NP-007395	0.120 ±0.002	37134 ±8848	1.040			
NP-007396	0.117 ±0.002	59627 ±9512	1.670			

NP-007397	0.123 ± 0.001	37739 ± 1375	1.057			
NP-007398	0.117 ± 0.004	49768 ± 8277	1.393			
NP-007401	0.116 ± 0.004	39385 ± 3474	1.103			
NP-007406	0.117 ± 0.007	33632 ± 1589	0.942			
NP-007410	0.110 ± 0.005	40183 ± 3750	1.125			
NP-007411	0.121 ± 0.006	32964 ± 1971	0.923			
NP-007413	0.123 ± 0.003	50646 ± 1784	1.418			
NP-007414	0.118 ± 0.002	48406 ± 6537	1.355			
NP-007415	0.112 ± 0.011	56120 ± 2251	1.503			
NP-007417	0.114 ± 0.007	47911 ± 537	1.341			
NP-007422	0.107 ± 0.002	36516 ± 4263	1.022			
NP-007423	0.116 ± 0.007	50188 ± 5622	1.405			
NP-007424	0.121 ± 0.004	44742 ± 3867	1.253			
NP-007425	0.116 ± 0.007	32875 ± 537	0.920			
NP-007426	0.112 ± 0.004	37323 ± 2236	1.045			
NP-007427	0.112 ± 0.011	45580 ± 4640	1.276			
NP-007431	0.118 ± 0.005	51266 ± 8274	1.435			
NP-007434	0.122 ± 0.005	47071 ± 6005	1.318			
NP-007436	0.118 ± 0.006	44403 ± 4868	1.243			
NP-007437	0.115 ± 0.002	26822 ± 1936	0.751			
NP-007438	0.107 ± 0.006	37772 ± 4987	1.058			
NP-007439	0.125 ± 0.002	40871 ± 3067	1.144			
NP-007442	0.111 ± 0.008	48843 ± 858	1.368			
NP-007443	0.115 ± 0.004	46257 ± 3682	1.295			
NP-007447	0.123 ± 0.002	63411 ± 8349	1.698			
NP-007460	0.124 ± 0.001	44712 ± 1670	1.252			
NP-007462	0.117 ± 0.005	32234 ± 1323	0.903			
NP-007463	0.110 ± 0.008	28448 ± 1358	0.797			
NP-007464	0.117 ± 0.002	47412 ± 3620	1.328			
NP-007465	0.128 ± 0.011	40757 ± 3872	1.141			
NP-007466	0.113 ± 0.002	35514 ± 1052	0.994			
NP-007468	0.114 ± 0.005	35386 ± 741	0.991			

NP-007471	0.115 ± 0.002	33249 ± 1306	0.931			
NP-007472	0.112 ± 0.003	40706 ± 781	1.140			
NP-007474	0.130 ± 0.006	28289 ± 4539	0.792			
NP-007481	0.118 ± 0.008	35419 ± 1935	0.992			
NP-007482	0.129 ± 0.006	37809 ± 1808	1.059			
NP-007484	0.121 ± 0.003	32287 ± 689	0.904			
NP-007488	0.117 ± 0.002	39038 ± 1732	1.093			
NP-007489	0.123 ± 0.007	23470 ± 6152	0.657			
NP-007490	0.120 ± 0.004	53019 ± 2249	1.485			
NP-007494	0.111 ± 0.002	53906 ± 2881	1.443			
NP-007496	0.120 ± 0.007	28221 ± 1120	0.790			
NP-007499	0.114 ± 0.004	20677 ± 5983	0.579			
NP-007510	0.124 ± 0.004	47320 ± 5904	1.325			
NP-007511	0.119 ± 0.004	48013 ± 722	1.344			
NP-007513	0.096 ± 0.003 *					
NP-007514	0.122 ± 0.006	35983 ± 1111	1.008			
NP-007515	0.115 ± 0.003	44343 ± 6181	1.242			
NP-007520	0.114 ± 0.011	51837 ± 2400	1.451			
NP-007524	0.121 ± 0.004	43860 ± 2905	1.228			
NP-007529	0.124 ± 0.003	37584 ± 2891	1.052			
NP-007534	0.108 ± 0.003	67341 ± 2135	1.803			
NP-007538	0.113 ± 0.005	44593 ± 3850	1.249			
NP-007544	0.120 ± 0.004	31367 ± 867	0.878			
NP-007545	0.124 ± 0.002	31796 ± 2636	0.890			
NP-007548	0.111 ± 0.006	27491 ± 1864	0.770			
NP-007552	0.113 ± 0.005	29557 ± 978	0.828			
NP-007553	0.108 ± 0.007	22440 ± 4928	0.628			
NP-007554	0.106 ± 0.003	32974 ± 980	0.923			
NP-007555	0.113 ± 0.004	37777 ± 2480	1.058			
NP-007556	0.114 ± 0.010	47853 ± 2801	1.340			
NP-007557	0.113 ± 0.004	37097 ± 2479	1.039			
NP-007558	0.120 ± 0.004	40393 ± 3348	1.131			

NP-007559	0.108 ± 0.002	29030 ± 6164	0.813			
NP-007560	0.136 ± 0.015	31256 ± 620	0.875			
NP-007561	0.116 ± 0.002	31147 ± 1108	0.872			
NP-007562	0.117 ± 0.009	36008 ± 5826	1.008			
NP-007563	0.117 ± 0.004	26624 ± 2091	0.745			
NP-007565	0.111 ± 0.001	50886 ± 2459	1.425			
NP-007572	0.122 ± 0.006	40717 ± 335	1.140			
NP-007573	0.110 ± 0.001	38615 ± 3980	1.081			
NP-007576	0.126 ± 0.003	31074 ± 3199	0.870			
NP-007580	0.119 ± 0.005	22429 ± 5536	0.628			
NP-007581	0.121 ± 0.003	33927 ± 1990	0.950			
NP-007582	0.117 ± 0.001	43324 ± 1572	1.213			
NP-007583	0.117 ± 0.002	32321 ± 2166	0.905			
NP-007584	0.112 ± 0.004	51447 ± 2439	1.440			
NP-007585	0.122 ± 0.006	35816 ± 4129	1.003			
NP-007586	0.116 ± 0.003	37796 ± 4903	1.058			
NP-007587	0.121 ± 0.011	32859 ± 1605	0.920			
NP-007588	0.123 ± 0.006	49913 ± 3898	1.398			
NP-007594	0.125 ± 0.002	36690 ± 3283	1.027			
NP-007600	0.110 ± 0.005	41859 ± 4012	1.172			
NP-007603	0.114 ± 0.003	33109 ± 1034	0.927			
NP-007612	0.119 ± 0.007	21234 ± 1895	0.569			
NP-007618	0.120 ± 0.002	33779 ± 3456	0.946			
NP-007620	0.118 ± 0.007	42381 ± 1149	1.187			
NP-007628	0.127 ± 0.005	34112 ± 2889	0.955			
NP-007629	0.123 ± 0.011	39898 ± 523	1.117			
NP-007632	0.117 ± 0.006	32507 ± 4939	0.910			
NP-007638	0.121 ± 0.007	35380 ± 4758	0.991			
NP-007639	0.124 ± 0.007	33196 ± 1125	0.929			
NP-007640	0.111 ± 0.002	38780 ± 1766	1.086			
NP-007641	0.115 ± 0.004	45984 ± 8747	1.288			
NP-007642	0.120 ± 0.008	35571 ± 290	0.996			

NP-007643	0.124 ± 0.003	33278 ± 1078	0.932			
NP-007644	0.125 ± 0.001	37614 ± 753	1.053			
NP-007648	0.132 ± 0.010	33056 ± 1623	0.926			
NP-007652	0.111 ± 0.007	51008 ± 5212	1.428			
NP-007654	0.114 ± 0.002	29439 ± 3888	0.824			
NP-007657	0.116 ± 0.001	48450 ± 1821	1.357			
NP-007664	0.113 ± 0.017	35793 ± 6502	1.002			
NP-007671	0.112 ± 0.003	39054 ± 3434	1.093			
NP-007673	0.115 ± 0.007	29406 ± 5231	0.823			
NP-007676	0.118 ± 0.003	44129 ± 6387	1.236			
NP-007680	0.112 ± 0.009	35691 ± 5231	0.999			
NP-007684	0.116 ± 0.001	30975 ± 5493	0.867			
NP-007686	0.111 ± 0.003	25837 ± 2760	0.723			
NP-007693	0.121 ± 0.008	39462 ± 7590	1.105			
NP-007695	0.113 ± 0.002	34932 ± 4544	0.978			
NP-007700	0.125 ± 0.006	43971 ± 1922	1.231			
NP-007701	0.111 ± 0.003	47859 ± 2500	1.340			
NP-007707	0.107 ± 0.001	33815 ± 6615	0.947			
NP-007710	0.128 ± 0.003	29339 ± 1411	0.821			
NP-007713	0.113 ± 0.006	26295 ± 684	0.736			
NP-007718	0.117 ± 0.002	31902 ± 1039	0.893			
NP-007720	0.123 ± 0.004	34729 ± 3696	0.972			
NP-007722	0.115 ± 0.005	33564 ± 2334	0.940			
NP-007724	0.106 ± 0.012	26081 ± 3358	0.730			
NP-007725	0.100 ± 0.011	45295 ± 9945	1.268			
NP-007726	0.119 ± 0.006	48125 ± 4116	1.347			
NP-007727	0.077 ± 0.009 **					
NP-007728	0.117 ± 0.005	47524 ± 9641	1.331			
NP-007735	0.110 ± 0.005	32432 ± 4442	0.908			
NP-007737	0.112 ± 0.002	39936 ± 7476	1.118			
NP-007740	0.121 ± 0.007	35044 ± 4042	0.981			
NP-007743	0.108 ± 0.010	39582 ± 1110	1.108			

NP-007746	0.137 ± 0.031	41032 ± 2325	1.149			
NP-007748	0.118 ± 0.008	42021 ± 4288	1.177			
NP-007749	0.116 ± 0.003	31284 ± 8987	0.876			
NP-007751	0.114 ± 0.003	41681 ± 3355	1.167			
NP-007753	0.114 ± 0.002	47223 ± 1270	1.322			
NP-007755	0.113 ± 0.002	44428 ± 2343	1.244			
NP-007768	0.122 ± 0.003	27198 ± 1942	0.762			
NP-007770	0.119 ± 0.005	38147 ± 2612	1.068			
NP-007771	0.106 ± 0.003	38925 ± 1815	1.090			
NP-007782	0.109 ± 0.001	43337 ± 3368	1.213			
NP-007785	0.118 ± 0.002	60212 ± 3257	1.612			
NP-007806	0.119 ± 0.002	37744 ± 2609	1.057			
NP-007809	0.126 ± 0.004	38197 ± 5110	1.069			
NP-007812	0.113 ± 0.002	41549 ± 3445	1.163			
NP-007820	0.127 ± 0.007	35843 ± 1749	1.004			
NP-007823	0.118 ± 0.004	36531 ± 990	1.023			
NP-007834	0.109 ± 0.005	50057 ± 897	1.402			
NP-007845	0.113 ± 0.003	56201 ± 6740	1.574			
NP-007846	0.124 ± 0.002	37351 ± 2079	1.046			
NP-007847	0.124 ± 0.006	40998 ± 1111	1.148			
NP-007851	0.107 ± 0.001	28363 ± 1312	0.794			
NP-007852	0.108 ± 0.003	33610 ± 1736	0.941			
NP-007858	0.120 ± 0.003	29683 ± 4616	0.831			
NP-007865	0.105 ± 0.002	49525 ± 2090	1.387			
NP-007866	0.116 ± 0.007	36949 ± 2878	1.035			
NP-007872	0.118 ± 0.007	26669 ± 5111	0.747			
NP-007874	0.117 ± 0.006	42082 ± 1154	1.178			
NP-007876	0.115 ± 0.002	44989 ± 4489	1.260			
NP-007878	0.129 ± 0.005	36144 ± 3219	1.012			
NP-007883	0.111 ± 0.003	21266 ± 2355	0.595			
NP-007884	0.118 ± 0.001	20507 ± 999	0.549			
NP-007885	0.113 ± 0.003	25742 ± 1198	0.721			

NP-007886	0.113 ± 0.008	34153 ± 2916	0.956			
NP-007887	0.120 ± 0.005	49245 ± 778	1.379			
NP-007889	0.115 ± 0.003	33867 ± 287	0.948			
NP-007891	0.126 ± 0.009	36829 ± 733	1.031			
NP-007892	0.119 ± 0.004	33319 ± 2606	0.933			
NP-007894	0.124 ± 0.007	36623 ± 3883	1.025			
NP-007895	0.126 ± 0.007	29855 ± 6365	0.836			
NP-007896	0.116 ± 0.005	33000 ± 3664	0.924			
NP-007897	0.113 ± 0.001	47703 ± 3425	1.336			
NP-007898	0.138 ± 0.008	37872 ± 881	1.060			
NP-007901	0.125 ± 0.003	29337 ± 539	0.821			
NP-007906	0.113 ± 0.004	43913 ± 1383	1.230			
NP-007909	0.118 ± 0.004	33447 ± 5149	0.936			
NP-007912	0.117 ± 0.002	39636 ± 1266	1.110			
NP-007919	0.105 ± 0.006	25442 ± 2354	0.712			
NP-007923	0.133 ± 0.009	31059 ± 5497	0.870			
NP-007926	0.112 ± 0.006	40928 ± 1214	1.146			
NP-007930	0.116 ± 0.001	36195 ± 502	1.013			
NP-007931	0.126 ± 0.004	41074 ± 3464	1.150			
NP-007932	0.116 ± 0.004	37315 ± 2853	1.045			
NP-007935	0.114 ± 0.005	37045 ± 678	1.037			
NP-007943	0.105 ± 0.006	53208 ± 6378	1.490			
NP-007944	0.123 ± 0.006	31097 ± 4246	0.871			
NP-007945	0.117 ± 0.003	52030 ± 3497	1.457			
NP-007949	0.111 ± 0.006	32199 ± 556	0.902			
NP-007951	0.128 ± 0.010	37353 ± 1013	1.046			
NP-007953	0.121 ± 0.004	30786 ± 1669	0.862			
NP-007956	0.115 ± 0.008	49655 ± 989	1.390			
NP-007958	0.125 ± 0.006	28593 ± 879	0.801			
NP-007960	0.110 ± 0.002	33889 ± 1360	0.949			
NP-007961	0.130 ± 0.010	36032 ± 6072	1.009			
NP-007966	0.113 ± 0.011	31197 ± 941	0.873			

NP-007969	0.104 ±0.010	28883 ±4259	0.809			
NP-007970	0.115 ±0.003	21548 ±6134	0.603			
NP-007973	0.117 ±0.004	49463 ±1611	1.385			
NP-007974	0.119 ±0.006	39325 ±1477	1.101			
NP-007975	0.112 ±0.003	49015 ±4699	1.372			
NP-007979	0.115 ±0.006	31580 ±1132	0.884			
NP-007980	0.116 ±0.007	44755 ±5270	1.253			
NP-007982	0.118 ±0.007	44383 ±2732	1.243			
NP-007994	0.115 ±0.006	37603 ±834	1.053			
NP-007996	0.116 ±0.002	38943 ±364	1.090			
NP-007999	0.122 ±0.003	41506 ±2816	1.162			
NP-008003	0.121 ±0.004	33379 ±4868	0.935			
NP-008009	0.119 ±0.004	52443 ±4663	1.468			
NP-008010	0.126 ±0.014	36334 ±3184	1.017			
NP-008014	0.112 ±0.005	33346 ±941	0.934			
NP-008016	0.119 ±0.004	44581 ±2506	1.248			
NP-008026	0.120 ±0.004	30817 ±2191	0.863			
NP-008035	0.113 ±0.001	36305 ±669	1.017			
NP-008036	0.111 ±0.003	35795 ±120	1.002			
NP-008037	0.115 ±0.005	32750 ±1738	0.917			
NP-008050	0.115 ±0.001	49147 ±2483	1.376			
NP-008055	0.105 ±0.007	36001 ±2532	1.008			
NP-008058	0.126 ±0.001	27183 ±1010	0.761			
NP-008066	0.124 ±0.006	35770 ±1029	1.002			
NP-008069	0.111 ±0.007	30903 ±2829	0.865			
NP-008072	0.122 ±0.002	36514 ±2123	1.022			
NP-008073	0.115 ±0.001	36706 ±1940	1.028			
NP-008081	0.108 ±0.007	37979 ±2151	1.063			
NP-008084	0.120 ±0.001	37327 ±849	1.045			
NP-008091	0.119 ±0.006	41085 ±4040	1.150			
NP-008092	0.117 ±0.002	39726 ±2986	1.112			
NP-008093	0.114 ±0.006	30598 ±3506	0.857			

NP-008095	0.116 ±0.006	42530 ±3206	1.191			
NP-008097	0.119 ±0.007	40890 ±1334	1.145			
NP-008098	0.124 ±0.003	33632 ±1141	0.942			
NP-008099	0.126 ±0.005	38837 ±865	1.087			
NP-008100	0.136 ±0.029	39901 ±1507	1.117			
NP-008102	0.126 ±0.001	40530 ±2552	1.135			
NP-008103	0.114 ±0.004	43160 ±4283	1.208			
NP-008104	0.124 ±0.002	40360 ±2650	1.130			
NP-008105	0.112 ±0.003	43043 ±4735	1.205			
NP-008106	0.127 ±0.004	39738 ±2531	1.113			
NP-008107	0.135 ±0.008	34258 ±3184	0.959			
NP-008108	0.118 ±0.004	33119 ±2815	0.927			
NP-008111	0.123 ±0.005	44713 ±3253	1.252			
NP-008121	0.124 ±0.005	37212 ±2856	1.042			
NP-008125	0.121 ±0.003	43307 ±1536	1.213			
NP-008126	0.111 ±0.012	31438 ±1450	0.880			
NP-008130	0.114 ±0.005	37425 ±7467	1.048			
NP-008134	0.134 ±0.012	25611 ±472	0.717			
NP-008137	0.112 ±0.001	51265 ±6100	1.435			
NP-008138	0.113 ±0.002	35267 ±2406	0.987			
NP-008183	0.113 ±0.006	42493 ±598	1.190			
NP-008189	0.105 ±0.006	20618 ±1686	0.552			
NP-008190	0.120 ±0.004	42121 ±2613	1.179			
NP-008192	0.118 ±0.006	38460 ±8017	1.077			
NP-008195	0.113 ±0.005	38539 ±2810	1.079			
NP-008198	0.108 ±0.003	26245 ±7613	0.735			
NP-008199	0.115 ±0.011	30104 ±2456	0.843			
NP-008200	0.119 ±0.008	39088 ±884	1.094			
NP-008201	0.113 ±0.002	33746 ±1665	0.945			
NP-008202	0.110 ±0.009	42557 ±1507	1.192			
NP-008203	0.117 ±0.002	55706 ±3385	1.492			
NP-008205	0.114 ±0.006	38171 ±3110	1.069			

NP-008207	0.113 ± 0.003	47487 ± 1144	1.330			
NP-008208	0.109 ± 0.002	30563 ± 570	0.856			
NP-008219	0.117 ± 0.004	27395 ± 4311	0.767			
NP-008225	0.117 ± 0.010	25711 ± 3596	0.720			
NP-008227	0.113 ± 0.011	35686 ± 1770	0.999			
NP-008232	0.125 ± 0.003	30811 ± 286	0.863			
NP-008233	0.115 ± 0.011	49139 ± 3870	1.376			
NP-008234	0.113 ± 0.007	31622 ± 1652	0.885			
NP-008235	0.133 ± 0.007	41014 ± 1908	1.148			
NP-008236	0.108 ± 0.007	32747 ± 639	0.917			
NP-008237	0.118 ± 0.033	56379 ± 7931	1.579			
NP-008244	0.114 ± 0.003	52923 ± 9033	1.482			
NP-008246	0.115 ± 0.012	38616 ± 4656	1.081			
NP-008247	0.119 ± 0.003	38565 ± 2160	1.080			
NP-008248	0.132 ± 0.009	27354 ± 1575	0.766			
NP-008257	0.119 ± 0.006	31995 ± 511	0.896			
NP-008261	0.113 ± 0.006	22620 ± 2339	0.633			
NP-008262	0.117 ± 0.003	29773 ± 1657	0.834			
NP-008266	0.111 ± 0.008	54679 ± 3957	1.531			
NP-008267	0.120 ± 0.013	29960 ± 3182	0.839			
NP-008269	0.113 ± 0.007	29857 ± 4774	0.836			
NP-008270	0.123 ± 0.013	45984 ± 2781	1.288			
NP-008271	0.111 ± 0.002	34183 ± 1279	0.957			
NP-008272	0.119 ± 0.005	8539 ± 293	0.229	0.116 ± 0.009	17168 ± 2533	0.525
NP-008273	0.117 ± 0.006	32341 ± 1414	0.906			
NP-008275	0.112 ± 0.004	39873 ± 2165	1.116			
NP-008280	0.125 ± 0.007	58449 ± 2704	1.565			
NP-008282	0.120 ± 0.002	34545 ± 2290	0.967			
NP-008283	0.114 ± 0.005	32931 ± 916	0.922			
NP-008284	0.120 ± 0.006	36624 ± 866	1.025			
NP-008285	0.117 ± 0.008	30750 ± 1670	0.861			
NP-008287	0.112 ± 0.007	31189 ± 2519	0.873			

NP-008290	0.117 ± 0.003	57459 ± 5827	1.538			
NP-008293	0.109 ± 0.007	40096 ± 1934	1.123			
NP-008294	0.124 ± 0.005	52771 ± 7390	1.478			
NP-008295	0.117 ± 0.004	36628 ± 4748	1.026			
NP-008296	0.123 ± 0.002	45511 ± 8571	1.274			
NP-008297	0.127 ± 0.007	56445 ± 4259	1.511			
NP-008298	0.115 ± 0.011	35684 ± 4087	0.999			
NP-008299	0.116 ± 0.004	64984 ± 4589	1.740			
NP-008300	0.122 ± 0.006	47751 ± 7833	1.337			
NP-008301	0.110 ± 0.003	48000 ± 2274	1.344			
NP-008303	0.115 ± 0.002	27763 ± 607	0.777			
NP-008309	0.118 ± 0.002	46523 ± 2242	1.303			
NP-008314	0.108 ± 0.005	57201 ± 9037	1.602			
NP-008315	0.118 ± 0.005	31938 ± 5507	0.894			
NP-008317	0.123 ± 0.002	43463 ± 2650	1.217			
NP-008321	0.120 ± 0.002	29162 ± 4066	0.817			
NP-008329	0.110 ± 0.007	45008 ± 3251	1.260			
NP-008330	0.109 ± 0.002	97816 ± 8452	2.619	0.106 ± 0.002	43568 ± 5951	1.333
NP-008332	0.093 ± 0.007 *					
NP-008340	0.116 ± 0.007	34304 ± 1915	0.960			
NP-008350	0.127 ± 0.006	33659 ± 947	0.942			
NP-008353	0.125 ± 0.003	56644 ± 3726	1.517			
NP-008358	0.110 ± 0.002	38037 ± 2040	1.065			
NP-008359	0.112 ± 0.002	42094 ± 1345	1.179			
NP-008361	0.124 ± 0.004	49231 ± 6715	1.378			
NP-008363	0.130 ± 0.011	38675 ± 5375	1.083			
NP-008375	0.113 ± 0.004	33813 ± 4706	0.947			
NP-008377	0.124 ± 0.003	70877 ± 2289	1.898			
NP-008379	0.122 ± 0.006	38849 ± 973	1.088			
NP-008382	0.123 ± 0.006	44647 ± 7278	1.250			
NP-008383	0.116 ± 0.007	25987 ± 1836	0.728			
NP-008384	0.113 ± 0.004	57930 ± 7788	1.622			

NP-008392	0.125 ±0.003	58996 ±3060	1.580			
NP-008393	0.113 ±0.016	32804 ±5650	0.918			
NP-008394	0.112 ±0.002	35544 ±8455	0.995			
NP-008395	0.118 ±0.003	38549 ±4138	1.079			
NP-008396	0.121 ±0.004	35436 ±2498	0.992			
NP-008400	0.117 ±0.005	53691 ±2740	1.503			
NP-008401	0.113 ±0.003	42797 ±2510	1.198			
NP-008402	0.118 ±0.002	44153 ±502	1.236			
NP-008410	0.124 ±0.001	45333 ±4090	1.269			
NP-008416	0.113 ±0.001	30345 ±1525	0.850			
NP-008418	0.119 ±0.003	28287 ±3040	0.792			
NP-008419	0.131 ±0.007	40894 ±1793	1.145			
NP-008433	0.114 ±0.006	44435 ±1319	1.244			
NP-008434	0.120 ±0.006	52272 ±2911	1.464			
NP-008438	0.134 ±0.003	38891 ±4675	1.089			
NP-008441	0.113 ±0.001	40233 ±855	1.127			
NP-008445	0.108 ±0.011	63671 ±1453	1.705			
NP-008450	0.117 ±0.002	37171 ±3783	1.041			
NP-008454	0.121 ±0.005	58288 ±2312	1.561			
NP-008456	0.114 ±0.002	40840 ±3103	1.143			
NP-008464	0.119 ±0.002	38020 ±2790	1.065			
NP-008465	0.123 ±0.003	54296 ±6027	1.520			
NP-008472	0.120 ±0.004	31239 ±1013	0.875			
NP-008477	0.120 ±0.007	52592 ±9925	1.473			
NP-008481	0.114 ±0.003	28521 ±102	0.799			
NP-008482	0.112 ±0.005	37224 ±6376	1.042			
NP-008484	0.112 ±0.013	47138 ±3282	1.320			
NP-008487	0.115 ±0.002	44140 ±2414	1.236			
NP-008488	0.116 ±0.001	50560 ±2063	1.416			
NP-008494	0.115 ±0.001	54007 ±3090	1.446			
NP-008498	0.112 ±0.004	36617 ±1411	1.025			
NP-008501	0.117 ±0.002	38511 ±350	1.078			

NP-008502	0.121 ±0.004	38107 ±2435	1.067				
NP-008503	0.115 ±0.004	43086 ±7640	1.206				
NP-008504	0.131 ±0.012	35051 ±2693	0.981				
NP-008505	0.112 ±0.002	29849 ±1591	0.836				
NP-008511	0.113 ±0.005	40883 ±6373	1.145				
NP-008513	0.111 ±0.003	51185 ±9040	1.433				
NP-008516	0.124 ±0.006	50399 ±3519	1.411				
NP-008517	0.110 ±0.001	36309 ±2598	1.017				
NP-008518	0.120 ±0.002	40589 ±2480	1.136				
NP-008524	0.122 ±0.004	41061 ±1660	1.150				
NP-008532	0.113 ±0.004	34328 ±4233	0.961				
NP-008534	0.127 ±0.007	47724 ±2386	1.336				
NP-008537	0.117 ±0.002	32248 ±702	0.903				
NP-008544	0.110 ±0.011	30371 ±1668	0.850				
NP-008547	0.116 ±0.012	28071 ±1266	0.786				
NP-008548	0.120 ±0.005	46720 ±3652	1.308				
NP-008552	0.118 ±0.007	55609 ±5921	1.557				
NP-008553	0.115 ±0.007	36906 ±2824	1.033				
NP-008555	0.113 ±0.007	37064 ±3051	1.038				
NP-008557	0.134 ±0.009	33821 ±2506	0.947				
NP-008559	0.121 ±0.004	42600 ±3840	1.193				
NP-008562	0.120 ±0.017	28768 ±7758	0.805				
NP-008563	0.121 ±0.005	37821 ±3038	1.059				
NP-008565	0.127 ±0.006	49416 ±4485	1.384				
NP-008567	0.123 ±0.005	50254 ±2591	1.407				
NP-008568	0.115 ±0.004	46693 ±3808	1.307				
NP-008570	0.112 ±0.005	37151 ±5310	1.040				
NP-008575	0.123 ±0.005	38217 ±4287	1.070				
NP-008579	0.125 ±0.016	41889 ±4615	1.173				
NP-008581	0.123 ±0.005	46955 ±5562	1.315				
NP-008586	0.111 ±0.010	39156 ±1339	1.096				
NP-008587	0.124 ±0.007	35190 ±4505	0.985				

NP-008588	0.116 ±0.002	31496 ±3282	0.882			
NP-008590	0.120 ±0.003	42768 ±5451	1.197			
NP-008592	0.118 ±0.007	46645 ±2720	1.306			
NP-008593	0.119 ±0.003	54213 ±8760	1.518			
NP-008595	0.114 ±0.003	36311 ±1982	1.017			
NP-008598	0.129 ±0.006	35408 ±2324	0.991			
NP-008601	0.125 ±0.006	36182 ±1141	1.013			
NP-008603	0.118 ±0.004	46081 ±6472	1.290			
NP-008606	0.123 ±0.002	50135 ±7782	1.404			
NP-008610	0.127 ±0.001	39398 ±2327	1.103			
NP-008611	0.122 ±0.002	42626 ±2631	1.194			
NP-008613	0.119 ±0.005	46133 ±3140	1.292			
NP-008615	0.116 ±0.004	35405 ±2103	0.991			
NP-008616	0.127 ±0.004	50612 ±2837	1.417			
NP-008618	0.118 ±0.004	53308 ±4565	1.493			
NP-008621	0.122 ±0.002	32813 ±1193	0.919			
NP-008622	0.118 ±0.007	56007 ±6158	1.568			
NP-008623	0.117 ±0.002	48745 ±5102	1.365			
NP-008624	0.111 ±0.005	41138 ±2140	1.152			
NP-008628	0.120 ±0.001	41399 ±3002	1.159			
NP-008629	0.116 ±0.008	46452 ±1722	1.301			
NP-008633	0.116 ±0.012	28942 ±5008	0.810			
NP-008634	0.119 ±0.003	40963 ±5130	1.147			
NP-008635	0.137 ±0.015	31510 ±2441	0.882			
NP-008639	0.111 ±0.001	41221 ±664	1.154			
NP-008640	0.115 ±0.006	39028 ±2480	1.093			
NP-008641	0.118 ±0.003	38777 ±6653	1.086			
NP-008647	0.116 ±0.008	46785 ±4110	1.310			
NP-008650	0.116 ±0.004	58066 ±3198	1.626			
NP-008660	0.125 ±0.010	32899 ±1752	0.921			
NP-008661	0.119 ±0.004	47059 ±3776	1.318			
NP-008662	0.124 ±0.009	56114 ±491	1.502			

NP-008679	0.117 ±0.000	43782 ±1862	1.226			
NP-008680	0.117 ±0.008	29351 ±1087	0.822			
NP-008682	0.124 ±0.002	34214 ±1093	0.958			
NP-008687	0.108 ±0.002	36326 ±5989	1.017			
NP-008689	0.119 ±0.004	67951 ±13716	1.819			
NP-008700	0.127 ±0.012	43679 ±2695	1.223			
NP-008701	0.126 ±0.006	61155 ±8428	1.637			
NP-008702	0.118 ±0.004	37032 ±1863	1.037			
NP-008703	0.127 ±0.005	45921 ±4128	1.286			
NP-008708	0.111 ±0.004	36914 ±3598	1.034			
NP-008711	0.115 ±0.007	28232 ±2573	0.790			
NP-008712	0.111 ±0.004	32869 ±2033	0.920			
NP-008713	0.111 ±0.005	31952 ±2250	0.895			
NP-008719	0.114 ±0.004	45531 ±3303	1.275			
NP-008720	0.116 ±0.006	35092 ±4340	0.983			
NP-008721	0.117 ±0.009	43328 ±2539	1.213			
NP-008729	0.128 ±0.002	52459 ±2421	1.469			
NP-008731	0.123 ±0.003	38806 ±3319	1.087			
NP-008736	0.120 ±0.004	35913 ±5381	1.006			
NP-008737	0.111 ±0.003	40989 ±2406	1.148			
NP-008739	0.109 ±0.002	23649 ±360	0.662			
NP-008742	0.114 ±0.004	36318 ±4826	1.017			
NP-008744	0.123 ±0.003	36556 ±2994	1.024			
NP-008749	0.116 ±0.009	57411 ±3512	1.607			
NP-008750	0.121 ±0.009	42072 ±2165	1.178			
NP-008751	0.120 ±0.007	54039 ±1371	1.447			
NP-008752	0.108 ±0.005	50255 ±3094	1.407			
NP-008756	0.125 ±0.004	71999 ±12404	1.928			
NP-008761	0.114 ±0.005	37775 ±2071	1.058			
NP-008763	0.113 ±0.005	34063 ±342	0.954			
NP-008774	0.113 ±0.006	41081 ±3802	1.150			
NP-008781	0.119 ±0.003	43841 ±1519	1.228			

NP-008782	0.114 ±0.008	37800 ±3226	1.058			
NP-008783	0.128 ±0.007	55261 ±6525	1.547			
NP-008785	0.121 ±0.011	39893 ±2146	1.117			
NP-008787	0.117 ±0.004	48648 ±8708	1.362			
NP-008789	0.119 ±0.006	27602 ±2013	0.773			
NP-008791	0.115 ±0.009	35474 ±1473	0.993			
NP-008792	0.123 ±0.006	44425 ±2226	1.244			
NP-008793	0.115 ±0.004	38491 ±2140	1.078			
NP-008795	0.116 ±0.008	33625 ±2586	0.941			
NP-008798	0.121 ±0.005	32648 ±897	0.914			
NP-008799	0.116 ±0.006	37870 ±4061	1.060			
NP-008800	0.120 ±0.005	33115 ±3179	0.927			
NP-008807	0.111 ±0.002	28509 ±1576	0.798			
NP-008813	0.118 ±0.018	42988 ±1037	1.204			
NP-008815	0.124 ±0.004	40233 ±5405	1.127			
NP-008817	0.123 ±0.002	44386 ±4801	1.243			
NP-008821	0.112 ±0.007	28947 ±2067	0.810			
NP-008822	0.112 ±0.005	31683 ±2563	0.887			
NP-008823	0.112 ±0.006	36827 ±1830	1.031			
NP-008824	0.119 ±0.007	37426 ±972	1.048			
NP-008826	0.112 ±0.008	39062 ±4292	1.094			
NP-008828	0.113 ±0.007	30557 ±1593	0.856			
NP-008829	0.115 ±0.015	36408 ±3275	1.019			
NP-008830	0.110 ±0.006	36429 ±1619	1.020			
NP-008831	0.125 ±0.007	48971 ±3845	1.371			
NP-008833	0.118 ±0.010	44555 ±1232	1.248			
NP-008837	0.124 ±0.005	60576 ±11529	1.696			
NP-008839	0.108 ±0.003	40565 ±2820	1.136			
NP-008842	0.120 ±0.006	52991 ±2847	1.484			
NP-008848	0.123 ±0.003	37764 ±3701	1.057			
NP-008854	0.132 ±0.005	40280 ±5210	1.128			
NP-008856	0.106 ±0.004	38113 ±4009	1.067			

NP-008857	0.114 ±0.004	39060 ±5396	1.094			
NP-008864	0.075 ±0.002 **					
NP-008868	0.118 ±0.006	43121 ±3193	1.207			
NP-008873	0.119 ±0.004	42787 ±6412	1.198			
NP-008882	0.108 ±0.005	32135 ±2270	0.900			
NP-008884	0.118 ±0.002	45882 ±8327	1.285			
NP-008887	0.115 ±0.003	41660 ±2282	1.166			
NP-008888	0.124 ±0.007	61030 ±4263	1.634			
NP-008889	0.115 ±0.006	29809 ±1728	0.835			
NP-008892	0.109 ±0.004	34457 ±1459	0.965			
NP-008894	0.117 ±0.003	31973 ±4340	0.895			
NP-008899	0.132 ±0.009	34758 ±858	0.973			
NP-008900	0.111 ±0.005	33839 ±1853	0.947			
NP-008905	0.118 ±0.003	33542 ±1902	0.939			
NP-008906	0.108 ±0.001	40453 ±2122	1.133			
NP-008907	0.121 ±0.007	43393 ±2323	1.215			
NP-008910	0.123 ±0.004	43368 ±4015	1.214			
NP-008915	0.122 ±0.009	40901 ±7880	1.145			
NP-008924	0.107 ±0.007	58424 ±4068	1.564			
NP-008926	0.120 ±0.001	37262 ±877	1.043			
NP-008928	0.118 ±0.003	35208 ±2118	0.986			
NP-008932	0.124 ±0.003	13287 ±358	0.356	0.117 ±0.003	20083 ±1535	0.615
NP-008937	0.114 ±0.006	21336 ±1318	0.571			
NP-008939	0.114 ±0.004	54067 ±716	1.448			
NP-008941	0.096 ±0.003 *					
NP-008942	0.115 ±0.002	44677 ±2897	1.251			
NP-008944	0.109 ±0.003	53808 ±7371	1.507			
NP-008946	0.120 ±0.001	40641 ±979	1.138			
NP-008949	0.112 ±0.003	56069 ±7643	1.570			
NP-008952	0.117 ±0.007	41098 ±5903	1.151			
NP-008959	0.107 ±0.004	42972 ±1177	1.203			
NP-008960	0.121 ±0.007	26811 ±2517	0.751			

NP-008961	0.118 ±0.013	40239 ±5135	1.127			
NP-008962	0.111 ±0.002	42666 ±3775	1.195			
NP-008967	0.134 ±0.013	25917 ±1067	0.726			
NP-008968	0.116 ±0.004	44353 ±499	1.242			
NP-008972	0.115 ±0.003	35464 ±1094	0.993			
NP-008974	0.111 ±0.004	44041 ±3538	1.233			
NP-008975	0.136 ±0.010	51033 ±6506	1.429			
NP-008976	0.117 ±0.006	31271 ±2406	0.876			
NP-008977	0.115 ±0.003	45581 ±4952	1.276			
NP-008980	0.111 ±0.017	27605 ±4383	0.773			
NP-008982	0.125 ±0.002	35656 ±2602	0.998			
NP-008983	0.116 ±0.006	49249 ±1739	1.379			
NP-008984	0.113 ±0.006	33686 ±2534	0.943			
NP-008989	0.118 ±0.007	46233 ±4206	1.294			
NP-008990	0.112 ±0.005	36475 ±2530	1.021			
NP-008991	0.119 ±0.003	39336 ±1979	1.101			
NP-008995	0.126 ±0.006	36932 ±2068	1.034			
NP-008998	0.112 ±0.005	42502 ±7467	1.190			
NP-008999	0.122 ±0.004	47114 ±4355	1.319			
NP-009000	0.120 ±0.010	56940 ±3419	1.525			
NP-009001	0.119 ±0.002	51220 ±2746	1.434			
NP-009002	0.126 ±0.001	60020 ±7768	1.681			
NP-009004	0.117 ±0.005	49994 ±5774	1.400			
NP-009005	0.116 ±0.001	65689 ±6544	1.759			
NP-009007	0.118 ±0.004	46832 ±1485	1.311			
NP-009008	0.115 ±0.008	43411 ±2583	1.215			
NP-009025	0.130 ±0.008	32491 ±2213	0.910			
NP-009035	0.114 ±0.003	31873 ±4556	0.892			
NP-009036	0.127 ±0.006	31346 ±3384	0.878			
NP-009046	0.126 ±0.005	19668 ±2151	0.527			
NP-009048	0.126 ±0.004	46753 ±2969	1.309			
NP-009049	0.129 ±0.007	37471 ±6309	1.049			

NP-009051	0.120 ±0.004	30895 ±6098	0.865			
NP-009052	0.117 ±0.002	35646 ±1193	0.998			
NP-009055	0.113 ±0.003	35875 ±1358	1.004			
NP-009062	0.138 ±0.009	30025 ±4533	0.841			
NP-009066	0.122 ±0.003	31970 ±5980	0.895			
NP-009072	0.114 ±0.002	66532 ±5728	1.781			
NP-009082	0.116 ±0.003	34410 ±1851	0.963			
NP-009084	0.114 ±0.005	35116 ±2378	0.983			
NP-009087	0.117 ±0.005	47550 ±2799	1.331			
NP-009088	0.113 ±0.002	30572 ±2969	0.856			
NP-009102	0.134 ±0.018	33796 ±3634	0.946			
NP-009103	0.109 ±0.006	38226 ±3468	1.070			
NP-009120	0.110 ±0.005	40999 ±2158	1.148			
NP-009142	0.113 ±0.008	40592 ±7059	1.137			
NP-009143	0.120 ±0.009	65019 ±5018	1.741			
NP-009146	0.118 ±0.002	56340 ±4291	1.509			
NP-009169	0.110 ±0.003	19140 ±897	0.512			
NP-009179	0.112 ±0.007	46095 ±4954	1.291			
NP-009185	0.113 ±0.003	39481 ±3461	1.105			
NP-009189	0.120 ±0.006	44803 ±3729	1.254			
NP-009209	0.120 ±0.005	36974 ±3501	1.035			
NP-009211	0.121 ±0.005	37354 ±6476	1.046			
NP-009212	0.124 ±0.007	32174 ±2520	0.901			
NP-009233	0.122 ±0.004	35960 ±1355	1.007			
NP-009245	0.118 ±0.001	38659 ±6745	1.082			
NP-009255	0.111 ±0.009	36759 ±2077	1.029			
NP-009259	0.121 ±0.005	45711 ±2852	1.280			
NP-009260	0.112 ±0.003	32223 ±2191	0.902			
NP-009265	0.119 ±0.003	37713 ±2326	1.056			
NP-009266	0.114 ±0.004	34497 ±3804	0.966			
NP-009276	0.131 ±0.013	32504 ±3778	0.910			
NP-009278	0.124 ±0.008	35850 ±2451	1.004			

NP-009280	0.126 ± 0.008	40131 ± 2704	1.124			
NP-009307	0.122 ± 0.008	49028 ± 2477	1.373			
NP-009314	0.113 ± 0.005	40397 ± 1239	1.131			
NP-009320	0.116 ± 0.003	50020 ± 2559	1.401			
NP-009325	0.117 ± 0.002	30354 ± 1437	0.850			
NP-009329	0.124 ± 0.007	49327 ± 3602	1.381			
NP-009336	0.095 ± 0.003 *					
NP-009337	0.117 ± 0.001	51711 ± 2439	1.448			
NP-009349	0.122 ± 0.004	44643 ± 1319	1.250			
NP-009367	0.120 ± 0.005	30565 ± 992	0.856			
NP-009373	0.117 ± 0.001	37810 ± 7822	1.059			
NP-009382	0.114 ± 0.001	36879 ± 628	1.033			
NP-009383	0.109 ± 0.009	31170 ± 1651	0.873			
NP-009385	0.110 ± 0.004	49847 ± 5495	1.396			
NP-009387	0.112 ± 0.003	37077 ± 880	1.038			
NP-009390	0.117 ± 0.005	57231 ± 3282	1.532			
NP-009393	0.119 ± 0.006	35776 ± 2473	1.002			
NP-009394	0.124 ± 0.006	39170 ± 3688	1.097			
NP-009409	0.110 ± 0.005	43533 ± 4464	1.219			
NP-009410	0.116 ± 0.003	34810 ± 1599	0.975			
NP-009421	0.113 ± 0.004	42667 ± 4948	1.195			
NP-009435	0.118 ± 0.002	36855 ± 4970	1.032			
NP-009444	0.116 ± 0.005	34381 ± 4225	0.963			
NP-009447	0.109 ± 0.006	37256 ± 1592	1.043			
NP-009449	0.108 ± 0.003	37016 ± 5087	1.036			
NP-009455	0.120 ± 0.012	27456 ± 3156	0.769			
NP-009464	0.126 ± 0.006	32612 ± 3265	0.913			
NP-009481	0.127 ± 0.005	32577 ± 3050	0.912			
NP-009485	0.115 ± 0.001	35405 ± 2005	0.991			
NP-009488	0.116 ± 0.002	45394 ± 9916	1.271			
NP-009489	0.116 ± 0.004	39354 ± 1336	1.102			
NP-009491	0.129 ± 0.008	46638 ± 3244	1.306			

NP-009492	0.135 ±0.004	30881 ±2238	0.865			
NP-009493	0.113 ±0.005	54450 ±9447	1.525			
NP-009502	0.123 ±0.002	64396 ±2660	1.724			
NP-009507	0.120 ±0.005	36093 ±4853	1.011			
NP-009509	0.112 ±0.007	51124 ±4168	1.431			
NP-009515	0.114 ±0.003	34944 ±3784	0.978			
NP-009521	0.113 ±0.004	31281 ±4508	0.876			
NP-009522	0.122 ±0.007	28695 ±8202	0.803			
NP-009526	0.116 ±0.006	33743 ±568	0.945			
NP-009565	0.116 ±0.002	33681 ±2752	0.943			
NP-009570	0.122 ±0.005	31043 ±566	0.869			
NP-009571	0.119 ±0.001	42792 ±8639	1.198			
NP-009572	0.113 ±0.002	33136 ±4749	0.928			
NP-009574	0.121 ±0.009	42258 ±3085	1.183			
NP-009580	0.120 ±0.007	31546 ±5202	0.883			
NP-009581	0.120 ±0.005	33990 ±1808	0.952			
NP-009584	0.119 ±0.006	46433 ±2863	1.300			
NP-009587	0.107 ±0.004	36631 ±6331	1.026			
NP-009603	0.118 ±0.002	40026 ±829	1.121			
NP-009604	0.114 ±0.006	40561 ±7413	1.136			
NP-009607	0.114 ±0.005	43619 ±1653	1.221			
NP-009609	0.125 ±0.005	51100 ±7775	1.431			
NP-009610	0.105 ±0.006	33393 ±3911	0.935			
NP-009611	0.127 ±0.011	32830 ±4382	0.919			
NP-009616	0.124 ±0.007	36135 ±3289	1.012			
NP-009619	0.122 ±0.006	34940 ±2526	0.978			
NP-009621	0.121 ±0.003	47823 ±6779	1.339			
NP-009629	0.132 ±0.005	43679 ±5303	1.223			
NP-009645	0.111 ±0.006	43650 ±3706	1.222			
NP-009658	0.110 ±0.005	55288 ±8041	1.548			
NP-009661	0.112 ±0.005	37846 ±4024	1.060			
NP-009662	0.115 ±0.007	37353 ±2649	1.046			

NP-009666	0.124 ± 0.004	29345 ± 830	0.822			
NP-009669	0.119 ± 0.008	42744 ± 3335	1.197			
NP-009674	0.110 ± 0.003	41538 ± 1330	1.163			
NP-009675	0.126 ± 0.006	43861 ± 728	1.228			
NP-009678	0.121 ± 0.005	41104 ± 1000	1.151			
NP-009679	0.115 ± 0.005	34364 ± 2247	0.962			
NP-009680	0.126 ± 0.004	39097 ± 3573	1.095			
NP-009683	0.132 ± 0.008	38639 ± 5147	1.082			
NP-009694	0.118 ± 0.002	36268 ± 1806	1.015			
NP-009699	0.119 ± 0.002	30254 ± 1624	0.847			
NP-009701	0.117 ± 0.006	37390 ± 2177	1.047			
NP-009705	0.121 ± 0.003	21584 ± 1246	0.604			
NP-009707	0.113 ± 0.005	36022 ± 1318	1.009			
NP-009712	0.113 ± 0.003	19532 ± 2068	0.523			
NP-009713	0.121 ± 0.004	31677 ± 642	0.887			
NP-009718	0.120 ± 0.003	28681 ± 3042	0.803			
NP-009729	0.127 ± 0.008	44608 ± 5071	1.249			
NP-009730	0.114 ± 0.002	35562 ± 2491	0.996			
NP-009734	0.117 ± 0.010	31674 ± 2144	0.887			
NP-009735	0.122 ± 0.004	35699 ± 1951	1.000			
NP-009741	0.120 ± 0.007	32602 ± 6167	0.913			
NP-009746	0.117 ± 0.008	50157 ± 6408	1.404			
NP-009752	0.114 ± 0.008	24793 ± 3338	0.694			
NP-009757	0.124 ± 0.002	31125 ± 2166	0.871			
NP-009768	0.111 ± 0.006	31030 ± 6216	0.869			
NP-009771	0.119 ± 0.004	29816 ± 1046	0.835			
NP-009772	0.121 ± 0.004	34604 ± 8022	0.969			
NP-009775	0.119 ± 0.004	25571 ± 1223	0.716			
NP-009776	0.118 ± 0.007	39481 ± 5750	1.105			
NP-009784	0.123 ± 0.009	41446 ± 3844	1.160			
NP-009791	0.127 ± 0.003	39119 ± 2149	1.095			
NP-009792	0.114 ± 0.001	47290 ± 5816	1.324			

NP-009795	0.112 ±0.004	31157 ±4083	0.872			
NP-009800	0.118 ±0.002	41373 ±3769	1.158			
NP-009803	0.113 ±0.004	38467 ±6724	1.077			
NP-009806	0.115 ±0.007	30477 ±3707	0.853			
NP-009807	0.122 ±0.004	5078 ±390	0.136	0.122 ±0.008	7477 ±541	0.229
NP-009815	0.112 ±0.001	30930 ±3587	0.866			
NP-009821	0.118 ±0.004	30319 ±3026	0.849			
NP-009822	0.115 ±0.002	27038 ±1929	0.757			
NP-009823	0.116 ±0.002	29455 ±999	0.825			
NP-009825	0.123 ±0.009	32393 ±2051	0.907			
NP-009826	0.127 ±0.005	34910 ±799	0.977			
NP-009832	0.110 ±0.002	39904 ±4314	1.117			
NP-009833	0.120 ±0.010	41582 ±1772	1.164			
NP-009836	0.137 ±0.008	47732 ±1927	1.336			
NP-009837	0.109 ±0.005	32627 ±1365	0.914			
NP-009843	0.122 ±0.004	34602 ±2153	0.969			
NP-009846	0.115 ±0.003	33509 ±410	0.938			
NP-009848	0.117 ±0.007	27390 ±2794	0.767			
NP-009849	0.117 ±0.005	42116 ±1315	1.179			
NP-009850	0.115 ±0.001	38743 ±1781	1.085			
NP-009851	0.111 ±0.001	63559 ±4091	1.702			
NP-009852	0.123 ±0.003	60770 ±4835	1.627			
NP-009853	0.114 ±0.004	40105 ±3537	1.123			
NP-009856	0.117 ±0.005	66427 ±1358	1.779			
NP-009858	0.115 ±0.003	40322 ±3777	1.129			
NP-009859	0.114 ±0.002	33111 ±1911	0.927			
NP-009860	0.128 ±0.007	23330 ±144	0.653			
NP-009861	0.116 ±0.006	33143 ±423	0.928			
NP-009863	0.121 ±0.003	46446 ±3729	1.300			
NP-009864	0.115 ±0.014	34997 ±5135	0.980			
NP-009865	0.115 ±0.004	33497 ±1700	0.938			
NP-009867	0.118 ±0.003	36512 ±2934	1.022			

NP-009870	0.116 ±0.003	11256 ±193	0.301	0.109 ±0.015	16802 ±859	0.514
NP-009882	0.117 ±0.007	30634 ±3315	0.858			
NP-009885	0.123 ±0.005	35056 ±619	0.982			
NP-009886	0.124 ±0.009	36025 ±2208	1.009			
NP-009887	0.113 ±0.003	48851 ±1349	1.368			
NP-009888	0.123 ±0.003	59328 ±2457	1.589			
NP-009889	0.130 ±0.012	43554 ±4195	1.219			
NP-009890	0.113 ±0.001	48224 ±571	1.350			
NP-009898	0.121 ±0.003	26558 ±2021	0.744			
NP-009907	0.124 ±0.005	37187 ±640	1.041			
NP-009911	0.129 ±0.011	19127 ±1952	0.512			
NP-009912	0.119 ±0.008	31833 ±935	0.891			
NP-009917	0.114 ±0.009	58071 ±6258	1.626			
NP-009923	0.118 ±0.001	40708 ±2239	1.140			
NP-009924	0.117 ±0.002	32319 ±1392	0.905			
NP-009928	0.121 ±0.006	28299 ±1112	0.792			
NP-009931	0.108 ±0.007	32941 ±2376	0.922			
NP-009932	0.111 ±0.006	99168 ±5328	2.655	0.117 ±0.013	33614 ±1705	1.029
NP-009937	0.113 ±0.004	38301 ±5082	1.072			
NP-009948	0.115 ±0.004	45986 ±5828	1.288			
NP-009950	0.122 ±0.002	40092 ±2863	1.123			
NP-009976	0.115 ±0.013	48145 ±5209	1.348			
NP-009993	0.107 ±0.008	31107 ±1308	0.871			
NP-010004	0.108 ±0.002	28047 ±440	0.785			
NP-010006	0.114 ±0.006	39182 ±1851	1.097			
NP-010008	0.116 ±0.010	37588 ±1036	1.052			
NP-010009	0.127 ±0.008	37769 ±4650	1.058			
NP-010011	0.116 ±0.004	40513 ±2629	1.134			
NP-010013	0.113 ±0.005	44281 ±2167	1.240			
NP-010017	0.118 ±0.004	32000 ±558	0.896			
NP-010021	0.116 ±0.008	56830 ±8164	1.591			
NP-010022	0.115 ±0.011	39235 ±5917	1.099			

NP-010023	0.120 ± 0.003	51104 ± 2585	1.431			
NP-010025	0.120 ± 0.007	39005 ± 2377	1.092			
NP-010026	0.114 ± 0.003	36430 ± 3444	1.020			
NP-010028	0.116 ± 0.003	22463 ± 1462	0.629			
NP-010031	0.111 ± 0.003	42095 ± 2721	1.179			
NP-010032	0.097 ± 0.004 *					
NP-010035	0.119 ± 0.003	38349 ± 2339	1.074			
NP-010049	0.113 ± 0.002	60815 ± 3541	1.628			
NP-010052	0.129 ± 0.006	30802 ± 783	0.862			
NP-010058	0.125 ± 0.002	26608 ± 1072	0.745			
NP-010061	0.118 ± 0.018	22988 ± 6601	0.644			
NP-010065	0.119 ± 0.010	46601 ± 2053	1.305			
NP-010067	0.116 ± 0.003	47593 ± 285	1.333			
NP-010070	0.115 ± 0.003	45364 ± 5126	1.270			
NP-010071	0.113 ± 0.001	12841 ± 594	0.344	0.115 ± 0.007	20576 ± 875	0.630
NP-010075	0.129 ± 0.005	53527 ± 4217	1.499			
NP-010076	0.122 ± 0.003	32817 ± 1771	0.919			
NP-010080	0.114 ± 0.006	28890 ± 387	0.809			
NP-010083	0.117 ± 0.003	35514 ± 1954	0.994			
NP-010086	0.117 ± 0.004	43867 ± 2171	1.228			
NP-010091	0.115 ± 0.006	35969 ± 2996	1.007			
NP-010103	0.120 ± 0.007	54440 ± 1801	1.458			
NP-010104	0.124 ± 0.007	54014 ± 4957	1.512			
NP-010107	0.121 ± 0.004	33226 ± 2307	0.930			
NP-010113	0.123 ± 0.013	39401 ± 2779	1.103			
NP-010131	0.121 ± 0.003	32059 ± 2450	0.898			
NP-010143	0.116 ± 0.002	36640 ± 2165	1.026			
NP-010150	0.118 ± 0.003	38864 ± 2242	1.088			
NP-010155	0.121 ± 0.003	34920 ± 3673	0.978			
NP-010159	0.121 ± 0.004	33338 ± 1740	0.933			
NP-010162	0.108 ± 0.008	45977 ± 1045	1.287			
NP-010163	0.111 ± 0.008	26738 ± 1317	0.749			

NP-010172	0.126 ±0.005	49839 ±2412	1.395			
NP-010176	0.120 ±0.005	28879 ±2248	0.809			
NP-010200	0.119 ±0.002	45370 ±1173	1.270			
NP-010225	0.128 ±0.013	39916 ±5041	1.118			
NP-010246	0.122 ±0.003	53642 ±3072	1.436			
NP-010262	0.122 ±0.003	41029 ±2234	1.149			
NP-010281	0.123 ±0.001	32316 ±51	0.905			
NP-010287	0.137 ±0.027	25390 ±790	0.711			
NP-010289	0.114 ±0.002	36847 ±1335	1.032			
NP-010308	0.118 ±0.003	40948 ±2713	1.147			
NP-010312	0.114 ±0.001	39618 ±827	1.109			
NP-010324	0.109 ±0.002	41565 ±7460	1.164			
NP-010330	0.122 ±0.002	30447 ±2180	0.852			
NP-010407	0.123 ±0.003	28454 ±989	0.797			
NP-010431	0.103 ±0.005	39640 ±1670	1.110			
NP-010435	0.118 ±0.011	32722 ±2856	0.916			
NP-010498	0.123 ±0.006	51538 ±3862	1.443			
NP-010521	0.117 ±0.004	19693 ±2752	0.527			
NP-010529	0.123 ±0.008	20635 ±1205	0.553			
NP-010540	0.123 ±0.006	31184 ±2362	0.873			
NP-010543	0.117 ±0.002	26840 ±1625	0.752			
NP-010569	0.114 ±0.003	36397 ±2486	1.019			
NP-010593	0.121 ±0.006	36882 ±1425	1.033			
NP-010596	0.116 ±0.006	39555 ±2749	1.108			
NP-010620	0.110 ±0.002	60599 ±4791	1.697			
NP-010639	0.118 ±0.007	53077 ±3954	1.486			
NP-010642	0.125 ±0.008	26585 ±4180	0.744			
NP-010643	0.123 ±0.008	34191 ±532	0.957			
NP-010644	0.071 ±0.003 **					
NP-010650	0.116 ±0.002	35962 ±2860	1.007			
NP-010652	0.116 ±0.003	32903 ±1555	0.921			
NP-010653	0.117 ±0.002	38206 ±2888	1.070			

NP-010655	0.124 ±0.006	52539 ±3247	1.471			
NP-010656	0.111 ±0.002	48134 ±2802	1.348			
NP-010657	0.102 ±0.004	35902 ±3178	1.005			
NP-010660	0.110 ±0.002	25376 ±7310	0.711			
NP-010662	0.110 ±0.010	38358 ±1961	1.074			
NP-010664	0.114 ±0.006	30053 ±2241	0.841			
NP-010668	0.114 ±0.003	24512 ±878	0.686			
NP-010678	0.115 ±0.007	27414 ±877	0.768			
NP-010682	0.131 ±0.016	32795 ±6290	0.918			
NP-010693	0.113 ±0.018	31925 ±1019	0.894			
NP-010695	0.109 ±0.008	53010 ±2094	1.419			
NP-010696	0.121 ±0.001	46170 ±4428	1.293			
NP-010698	0.118 ±0.002	56161 ±3199	1.572			
NP-010710	0.115 ±0.003	33565 ±3960	0.940			
NP-010713	0.115 ±0.010	35692 ±2978	0.999			
NP-010714	0.117 ±0.009	34655 ±4220	0.970			
NP-010716	0.115 ±0.021	32642 ±2388	0.914			
NP-010717	0.114 ±0.002	29324 ±1395	0.821			
NP-010725	0.124 ±0.004	38616 ±2914	1.081			
NP-010728	0.116 ±0.001	29717 ±887	0.832			
NP-010732	0.112 ±0.002	37820 ±911	1.059			
NP-010733	0.121 ±0.002	43297 ±772	1.212			
NP-010736	0.114 ±0.003	27938 ±5099	0.782			
NP-010741	0.106 ±0.006	40220 ±4357	1.126			
NP-010750	0.115 ±0.007	29391 ±1152	0.823			
NP-010759	0.118 ±0.006	29010 ±4762	0.812			
NP-010770	0.118 ±0.003	42567 ±1738	1.192			
NP-010771	0.119 ±0.006	37515 ±1081	1.050			
NP-010776	0.110 ±0.005	54243 ±6536	1.519			
NP-010812	0.123 ±0.010	29779 ±2708	0.834			
NP-010862	0.113 ±0.006	52560 ±8225	1.472			
NP-010863	0.117 ±0.004	36765 ±504	1.029			

NP-010885	0.125 ± 0.009	46268 ± 4285	1.295				
NP-010886	0.127 ± 0.004	48428 ± 779	1.356				
NP-010904	0.119 ± 0.005	40863 ± 2168	1.144				
NP-010913	0.126 ± 0.005	44905 ± 4159	1.257				
NP-010914	0.116 ± 0.006	28809 ± 4445	0.807				
NP-010919	0.120 ± 0.006	35424 ± 2427	0.992				
NP-010923	0.112 ± 0.004	65863 ± 2301	1.763				
NP-010950	0.117 ± 0.006	49249 ± 3057	1.379				
NP-010953	0.122 ± 0.010	51037 ± 7744	1.429				
NP-010955	0.121 ± 0.005	51669 ± 466	1.447				
NP-010957	0.119 ± 0.003	51708 ± 3720	1.448				
NP-010965	0.119 ± 0.007	46175 ± 1005	1.293				
NP-010970	0.117 ± 0.008	42146 ± 2435	1.180				
NP-010981	0.114 ± 0.014	48612 ± 6043	1.361				
NP-010984	0.110 ± 0.003	45769 ± 6895	1.282				
NP-011016	0.114 ± 0.007	39365 ± 3061	1.102				
NP-011138	0.119 ± 0.002	29510 ± 1862	0.826				
NP-011148	0.112 ± 0.009	39752 ± 5037	1.113				
NP-011150	0.116 ± 0.003	45350 ± 8034	1.270				
NP-011165	0.125 ± 0.004	33135 ± 1373	0.928				
NP-011186	0.119 ± 0.006	35205 ± 1053	0.986				
NP-011187	0.115 ± 0.002	36241 ± 2044	1.015				
NP-011189	0.121 ± 0.002	38227 ± 2000	1.070				
NP-011190	0.119 ± 0.006	51795 ± 4079	1.450				
NP-011191	0.122 ± 0.010	45673 ± 2985	1.279				
NP-011193	0.115 ± 0.002	46148 ± 2117	1.292				
NP-011195	0.119 ± 0.003	34611 ± 2580	0.969				
NP-011196	0.109 ± 0.003	39423 ± 4038	1.104				
NP-011198	0.126 ± 0.008	40754 ± 2161	1.141				
NP-011202	0.116 ± 0.004	40703 ± 1841	1.140				
NP-011210	0.122 ± 0.010	41210 ± 5422	1.154				
NP-011213	0.121 ± 0.005	50372 ± 2702	1.410				

NP-011216	0.117 ±0.004	33561 ±4789	0.940			
NP-011217	0.119 ±0.007	37856 ±1338	1.060			
NP-011218	0.112 ±0.003	54170 ±4708	1.517			
NP-011219	0.110 ±0.002	43936 ±5418	1.230			
NP-011220	0.122 ±0.007	32808 ±1421	0.919			
NP-011221	0.115 ±0.006	37382 ±5032	1.047			
NP-011222	0.117 ±0.004	32104 ±8589	0.899			
NP-011223	0.119 ±0.002	36742 ±3276	1.029			
NP-011224	0.113 ±0.002	35253 ±1462	0.987			
NP-011225	0.120 ±0.007	38652 ±2013	1.082			
NP-011226	0.118 ±0.014	37405 ±2307	1.047			
NP-011227	0.124 ±0.021	28773 ±543	0.806			
NP-011228	0.125 ±0.007	38037 ±4913	1.065			
NP-011232	0.117 ±0.008	29776 ±2710	0.834			
NP-011235	0.113 ±0.004	35083 ±6622	0.982			
NP-011239	0.117 ±0.004	50825 ±5566	1.423			
NP-011242	0.124 ±0.008	31281 ±3474	0.876			
NP-011297	0.118 ±0.005	31666 ±4518	0.887			
NP-011309	0.125 ±0.009	52086 ±5371	1.458			
NP-011328	0.119 ±0.009	18737 ±1424	0.502			
NP-011329	0.118 ±0.003	20474 ±1696	0.548			
NP-011342	0.115 ±0.007	33149 ±2260	0.928			
NP-011345	0.118 ±0.006	39958 ±1115	1.119			
NP-011356	0.120 ±0.005	19648 ±1223	0.526			
NP-011363	0.118 ±0.003	49312 ±5957	1.381			
NP-011368	0.125 ±0.003	44919 ±910	1.258			
NP-011371	0.117 ±0.004	31537 ±2346	0.883			
NP-011389	0.120 ±0.002	38122 ±2275	1.067			
NP-011395	0.121 ±0.003	48276 ±4424	1.352			
NP-011396	0.125 ±0.004	21673 ±520	0.580			
NP-011419	0.113 ±0.010	42692 ±1835	1.195			
NP-011421	0.100 ±0.004	35923 ±2560	1.006			

NP-011427	0.115 ±0.002	64713 ±3282	1.733			
NP-011428	0.116 ±0.003	35894 ±2337	1.005			
NP-011443	0.108 ±0.005	48183 ±3359	1.349			
NP-011444	0.115 ±0.003	49993 ±1811	1.400			
NP-011482	0.127 ±0.004	37542 ±2720	1.051			
NP-011483	0.127 ±0.012	29911 ±4714	0.837			
NP-011489	0.116 ±0.004	36245 ±679	1.015			
NP-011574	0.122 ±0.004	10296 ±547	0.276	0.119 ±0.005	18218 ±1135	0.557
NP-011694	0.099 ±0.001 *					
NP-011767	0.109 ±0.005	46726 ±1258	1.308			
NP-011780	0.121 ±0.005	30577 ±1475	0.856			
NP-011836	0.116 ±0.002	40332 ±1597	1.129			
NP-011846	0.122 ±0.002	42799 ±3673	1.198			
NP-011847	0.118 ±0.007	39661 ±4347	1.110			
NP-011855	0.119 ±0.001	40805 ±1287	1.143			
NP-011876	0.123 ±0.004	30780 ±1703	0.862			
NP-011878	0.113 ±0.003	41039 ±821	1.149			
NP-011898	0.112 ±0.002	60347 ±1074	1.616			
NP-011900	0.095 ±0.003 *					
NP-011928	0.122 ±0.002	44716 ±3609	1.252			
NP-011934	0.125 ±0.010	38076 ±7632	1.066			
NP-011940	0.116 ±0.008	33206 ±1938	0.930			
NP-011941	0.128 ±0.006	47594 ±2938	1.333			
NP-011953	0.109 ±0.004	51654 ±699	1.446			
NP-011958	0.115 ±0.001	72402 ±9616	1.939			
NP-011959	0.110 ±0.010	38865 ±3405	1.088			
NP-011966	0.119 ±0.004	47334 ±4969	1.325			
NP-011972	0.106 ±0.005	41563 ±3156	1.164			
NP-011979	0.101 ±0.005	38515 ±2647	1.078			
NP-011981	0.118 ±0.001	42249 ±2308	1.183			
NP-011987	0.123 ±0.004	55838 ±5213	1.563			
NP-011993	0.107 ±0.009	54480 ±2927	1.459			

NP-011996	0.115 ± 0.003	27207 ± 2692	0.762			
NP-011998	0.120 ± 0.006	31638 ± 1215	0.886			
NP-012001	0.121 ± 0.006	29938 ± 4754	0.838			
NP-012006	0.116 ± 0.004	38771 ± 6833	1.086			
NP-012008	0.124 ± 0.004	38725 ± 3092	1.084			
NP-012009	0.116 ± 0.022	28682 ± 4951	0.803			
NP-012018	0.109 ± 0.003	33945 ± 1112	0.950			
NP-012020	0.122 ± 0.006	29451 ± 927	0.825			
NP-012024	0.112 ± 0.002	31215 ± 3183	0.874			
NP-012032	0.120 ± 0.005	38286 ± 2248	1.072			
NP-012033	0.120 ± 0.003	28649 ± 1169	0.802			
NP-012036	0.113 ± 0.005	43368 ± 1592	1.214			
NP-012038	0.126 ± 0.010	29084 ± 5030	0.814			
NP-012039	0.117 ± 0.013	51781 ± 3396	1.450			
NP-012040	0.107 ± 0.002	39816 ± 792	1.115			
NP-012042	0.123 ± 0.009	28574 ± 852	0.800			
NP-012046	0.119 ± 0.005	32416 ± 6139	0.908			
NP-012048	0.110 ± 0.005	45378 ± 1720	1.271			
NP-012058	0.123 ± 0.002	39256 ± 2593	1.099			
NP-012060	0.112 ± 0.006	38584 ± 4129	1.080			
NP-012065	0.121 ± 0.005	31786 ± 5212	0.890			
NP-012068	0.114 ± 0.005	38565 ± 2468	1.080			
NP-012070	0.119 ± 0.008	39067 ± 6403	1.094			
NP-012075	0.115 ± 0.008	57812 ± 4578	1.619			
NP-012079	0.113 ± 0.004	45384 ± 5383	1.271			
NP-012083	0.118 ± 0.008	50862 ± 4666	1.424			
NP-012087	0.116 ± 0.003	42895 ± 2428	1.201			
NP-012091	0.116 ± 0.002	37706 ± 3473	1.056			
NP-012092	0.123 ± 0.002	53764 ± 5870	1.505			
NP-012095	0.117 ± 0.006	47550 ± 3966	1.331			
NP-012100	0.118 ± 0.006	51203 ± 8265	1.434			
NP-012102	0.112 ± 0.004	50564 ± 3774	1.416			

NP-012103	0.113 ±0.005	44009 ±1242	1.232			
NP-012105	0.123 ±0.006	45241 ±3355	1.267			
NP-012106	0.121 ±0.007	39812 ±5212	1.115			
NP-012107	0.123 ±0.001	44004 ±4198	1.232			
NP-012109	0.114 ±0.004	34495 ±4430	0.966			
NP-012112	0.125 ±0.008	48704 ±4867	1.364			
NP-012114	0.124 ±0.006	59181 ±6838	1.657			
NP-012117	0.118 ±0.001	48105 ±4337	1.347			
NP-012121	0.114 ±0.006	36319 ±3147	1.017			
NP-012131	0.107 ±0.005	32958 ±982	0.923			
NP-012133	0.109 ±0.005	39607 ±5594	1.109			
NP-012134	0.113 ±0.004	45998 ±2163	1.288			
NP-012135	0.117 ±0.004	38292 ±1325	1.072			
NP-012137	0.115 ±0.002	36207 ±3285	1.014			
NP-012139	0.126 ±0.004	45229 ±5350	1.266			
NP-012140	0.109 ±0.005	34696 ±3744	0.971			
NP-012144	0.124 ±0.003	37531 ±6210	1.051			
NP-012145	0.111 ±0.001	54309 ±9185	1.521			
NP-012147	0.117 ±0.002	33154 ±1302	0.928			
NP-012150	0.120 ±0.009	30666 ±2203	0.859			
NP-012151	0.115 ±0.008	39461 ±1755	1.105			
NP-012158	0.107 ±0.007	43596 ±3682	1.221			
NP-012161	0.115 ±0.004	35319 ±5311	0.989			
NP-012163	0.134 ±0.011	55374 ±2067	1.483			
NP-012164	0.124 ±0.005	35619 ±2351	0.997			
NP-012165	0.118 ±0.004	54108 ±3842	1.515			
NP-012166	0.113 ±0.003	30105 ±1456	0.843			
NP-012167	0.119 ±0.003	52098 ±1085	1.459			
NP-012176	0.130 ±0.010	43949 ±6112	1.231			
NP-012178	0.129 ±0.009	34014 ±5964	0.952			
NP-012183	0.116 ±0.006	39108 ±4086	1.095			
NP-012184	0.110 ±0.004	37503 ±2125	1.050			

NP-012185	0.115 ±0.003	34973 ±1895	0.979			
NP-012186	0.109 ±0.012	31248 ±2254	0.875			
NP-012187	0.112 ±0.008	27533 ±1856	0.771			
NP-012189	0.109 ±0.004	36542 ±221	1.023			
NP-012190	0.128 ±0.007	27488 ±490	0.770			
NP-012191	0.117 ±0.002	53188 ±716	1.424			
NP-012193	0.116 ±0.005	45676 ±5096	1.279			
NP-012194	0.124 ±0.004	58852 ±7371	1.648			
NP-012195	0.121 ±0.003	41034 ±5869	1.149			
NP-012196	0.118 ±0.006	37913 ±1855	1.062			
NP-012206	0.110 ±0.011	29361 ±644	0.822			
NP-012207	0.124 ±0.002	62441 ±9746	1.748			
NP-012208	0.106 ±0.007	35704 ±976	1.000			
NP-012209	0.108 ±0.002	33544 ±1577	0.939			
NP-012211	0.111 ±0.008	45189 ±9182	1.265			
NP-012213	0.124 ±0.002	31938 ±1490	0.894			
NP-012216	0.111 ±0.005	37864 ±504	1.060			
NP-012218	0.119 ±0.001	38121 ±4410	1.067			
NP-012219	0.112 ±0.002	38777 ±1459	1.086			
NP-012220	0.116 ±0.008	31997 ±1159	0.896			
NP-012221	0.109 ±0.001	47954 ±9109	1.343			
NP-012228	0.110 ±0.002	33631 ±3360	0.942			
NP-012230	0.114 ±0.004	34439 ±1176	0.964			
NP-012231	0.110 ±0.003	41265 ±1184	1.155			
NP-012232	0.120 ±0.003	65052 ±3549	1.742			
NP-012233	0.134 ±0.012	36005 ±2271	1.008			
NP-012235	0.122 ±0.002	52533 ±1751	1.471			
NP-012236	0.114 ±0.001	50817 ±5980	1.423			
NP-012237	0.116 ±0.007	39733 ±1606	1.113			
NP-012239	0.113 ±0.003	52253 ±1667	1.463			
NP-012245	0.120 ±0.004	49393 ±3455	1.383			
NP-012246	0.114 ±0.004	46063 ±1501	1.290			

NP-012247	0.110 ± 0.004	36599 ± 7201	1.025			
NP-012248	0.112 ± 0.003	27307 ± 2578	0.765			
NP-012253	0.125 ± 0.001	58642 ± 4891	1.570			
NP-012254	0.126 ± 0.011	47837 ± 1587	1.339			
NP-012255	0.129 ± 0.011	35273 ± 6562	0.988			
NP-012256	0.126 ± 0.006	40840 ± 4641	1.143			
NP-012260	0.109 ± 0.008	37814 ± 4502	1.059			
NP-012262	0.116 ± 0.006	40235 ± 3999	1.127			
NP-012268	0.117 ± 0.006	29305 ± 632	0.821			
NP-012269	0.122 ± 0.002	43001 ± 1614	1.204			
NP-012271	0.109 ± 0.005	39590 ± 1202	1.108			
NP-012273	0.127 ± 0.005	37748 ± 2428	1.057			
NP-012283	0.110 ± 0.003	28149 ± 2131	0.788			
NP-012286	0.124 ± 0.005	38350 ± 7190	1.074			
NP-012288	0.124 ± 0.010	25270 ± 3040	0.708			
NP-012295	0.117 ± 0.003	33846 ± 5391	0.948			
NP-012298	0.121 ± 0.005	39988 ± 3314	1.120			
NP-012306	0.107 ± 0.005	21583 ± 2415	0.604			
NP-012310	0.122 ± 0.001	56334 ± 6589	1.577			
NP-012311	0.118 ± 0.004	45301 ± 9192	1.268			
NP-012315	0.114 ± 0.002	32764 ± 1020	0.917			
NP-012318	0.123 ± 0.015	22749 ± 1226	0.609			
NP-012320	0.113 ± 0.008	33872 ± 1610	0.948			
NP-012321	0.116 ± 0.007	28262 ± 489	0.791			
NP-012324	0.119 ± 0.006	51664 ± 3591	1.447			
NP-012335	0.113 ± 0.005	40119 ± 2156	1.123			
NP-012336	0.123 ± 0.004	30573 ± 1739	0.856			
NP-012338	0.125 ± 0.004	45510 ± 6957	1.274			
NP-012340	0.126 ± 0.013	37902 ± 4292	1.061			
NP-012345	0.116 ± 0.025	44346 ± 6800	1.242			
NP-012347	0.128 ± 0.009	40437 ± 6760	1.132			
NP-012348	0.125 ± 0.006	58529 ± 4449	1.567			

NP-012349	0.118 ±0.005	38789 ±1563	1.086			
NP-012352	0.119 ±0.007	33412 ±679	0.936			
NP-012353	0.117 ±0.001	47024 ±212	1.317			
NP-012354	0.116 ±0.007	56374 ±3554	1.509			
NP-012355	0.118 ±0.003	46077 ±3100	1.290			
NP-012356	0.119 ±0.001	54087 ±814	1.448			
NP-012357	0.113 ±0.001	50588 ±324	1.416			
NP-012358	0.119 ±0.008	59751 ±1878	1.600			
NP-012359	0.119 ±0.002	38357 ±1511	1.074			
NP-012362	0.118 ±0.003	51067 ±1642	1.430			
NP-012363	0.122 ±0.001	38122 ±1871	1.067			
NP-012371	0.122 ±0.006	48954 ±4281	1.371			
NP-012374	0.119 ±0.008	38987 ±1294	1.092			
NP-012379	0.114 ±0.017	62866 ±4367	1.683			
NP-012380	0.119 ±0.008	52974 ±2748	1.483			
NP-012386	0.119 ±0.001	53671 ±917	1.437			
NP-012388	0.114 ±0.001	45921 ±3257	1.286			
NP-012389	0.121 ±0.003	53578 ±4925	1.435			
NP-012390	0.115 ±0.025	29717 ±4239	0.832			
NP-012392	0.123 ±0.003	49031 ±876	1.373			
NP-012394	0.120 ±0.001	48997 ±6916	1.372			
NP-012395	0.115 ±0.005	35126 ±3830	0.984			
NP-012401	0.115 ±0.003	37918 ±3409	1.062			
NP-012405	0.118 ±0.002	37856 ±2781	1.060			
NP-012406	0.116 ±0.002	40131 ±1541	1.124			
NP-012409	0.122 ±0.003	39334 ±3269	1.101			
NP-012411	0.110 ±0.004	48497 ±1937	1.358			
NP-012414	0.125 ±0.007	37167 ±4029	1.041			
NP-012415	0.115 ±0.012	41201 ±2402	1.154			
NP-012416	0.118 ±0.004	40753 ±2692	1.141			
NP-012417	0.109 ±0.005	51954 ±5049	1.455			
NP-012418	0.116 ±0.007	25304 ±3379	0.708			

NP-012419	0.130 ± 0.010	26380 ± 3496	0.739			
NP-012420	0.118 ± 0.005	34166 ± 7895	0.957			
NP-012421	0.110 ± 0.008	38639 ± 772	1.082			
NP-012425	0.117 ± 0.007	45079 ± 2913	1.262			
NP-012427	0.111 ± 0.005	29942 ± 3298	0.838			
NP-012430	0.110 ± 0.006	56133 ± 6070	1.572			
NP-012432	0.121 ± 0.006	35766 ± 1623	1.001			
NP-012433	0.112 ± 0.004	33487 ± 5618	0.938			
NP-012437	0.118 ± 0.001	44035 ± 7722	1.233			
NP-012438	0.108 ± 0.004	35078 ± 1175	0.982			
NP-012439	0.111 ± 0.003	37248 ± 1569	1.043			
NP-012440	0.122 ± 0.006	48877 ± 6618	1.369			
NP-012445	0.115 ± 0.004	34758 ± 388	0.973			
NP-012448	0.115 ± 0.001	35459 ± 3994	0.993			
NP-012452	0.111 ± 0.006	44432 ± 1666	1.244			
NP-012454	0.112 ± 0.003	37115 ± 5365	1.039			
NP-012455	0.124 ± 0.003	52762 ± 4989	1.477			
NP-012466	0.123 ± 0.005	44817 ± 2423	1.255			
NP-012476	0.096 ± 0.003 *					
NP-012477	0.113 ± 0.003	39205 ± 3388	1.098			
NP-012483	0.116 ± 0.004	58180 ± 2502	1.558			
NP-012484	0.111 ± 0.002	52641 ± 2268	1.474			
NP-012485	0.124 ± 0.008	35963 ± 1657	1.007			
NP-012488	0.126 ± 0.005	27153 ± 2665	0.760			
NP-012489	0.114 ± 0.003	43991 ± 2879	1.232			
NP-012490	0.116 ± 0.006	41315 ± 456	1.157			
NP-012491	0.107 ± 0.002	43459 ± 2764	1.217			
NP-012492	0.112 ± 0.004	55589 ± 4984	1.556			
NP-012493	0.111 ± 0.001	27751 ± 5166	0.777			
NP-012503	0.115 ± 0.003	42405 ± 2540	1.187			
NP-012505	0.117 ± 0.004	36939 ± 2539	1.034			
NP-012510	0.116 ± 0.003	45226 ± 3279	1.266			

NP-012511	0.116 ±0.007	42642 ±993	1.194			
NP-012516	0.113 ±0.007	40422 ±5108	1.132			
NP-012520	0.120 ±0.006	48551 ±5331	1.359			
NP-012529	0.119 ±0.007	51252 ±7051	1.435			
NP-012532	0.120 ±0.013	45971 ±1972	1.287			
NP-012533	0.113 ±0.004	46858 ±5358	1.312			
NP-012534	0.118 ±0.001	35450 ±663	0.993			
NP-012538	0.117 ±0.014	28052 ±1687	0.785			
NP-012541	0.110 ±0.001	37810 ±1795	1.059			
NP-012542	0.117 ±0.002	44755 ±338	1.253			
NP-012543	0.123 ±0.001	47938 ±1022	1.342			
NP-012544	0.118 ±0.004	46939 ±3577	1.314			
NP-012545	0.114 ±0.005	41300 ±803	1.156			
NP-012548	0.119 ±0.006	43271 ±1545	1.212			
NP-012549	0.124 ±0.003	47828 ±4806	1.339			
NP-012552	0.112 ±0.002	46408 ±4984	1.299			
NP-012566	0.102 ±0.013	10038 ±371	0.269	0.104 ±0.012	30682 ±2599	0.939
NP-012567	0.109 ±0.010	35700 ±2507	1.000			
NP-012570	0.121 ±0.002	42899 ±2278	1.201			
NP-012572	0.117 ±0.005	30840 ±1602	0.864			
NP-012573	0.119 ±0.004	52106 ±4828	1.459			
NP-012574	0.121 ±0.008	45778 ±6627	1.282			
NP-012575	0.117 ±0.017	30959 ±2902	0.867			
NP-012576	0.111 ±0.001	38826 ±4908	1.087			
NP-012577	0.115 ±0.001	54001 ±1718	1.512			
NP-012580	0.122 ±0.007	48366 ±2967	1.354			
NP-012581	0.123 ±0.008	46901 ±4496	1.313			
NP-012583	0.123 ±0.004	48473 ±2267	1.357			
NP-012584	0.115 ±0.003	44541 ±2457	1.247			
NP-012588	0.121 ±0.003	39160 ±3187	1.096			
NP-012590	0.126 ±0.005	20847 ±7774	0.584			
NP-012591	0.111 ±0.006	49307 ±6615	1.381			

NP-012592	0.121 ±0.003	60685 ±3186	1.625			
NP-012593	0.117 ±0.002	44012 ±1939	1.232			
NP-012594	0.108 ±0.004	56818 ±1149	1.521			
NP-012595	0.133 ±0.015	47125 ±5294	1.319			
NP-012596	0.112 ±0.005	47832 ±2041	1.339			
NP-012597	0.113 ±0.011	41820 ±4487	1.171			
NP-012598	0.112 ±0.005	32673 ±1799	0.915			
NP-012599	0.119 ±0.003	48847 ±2300	1.368			
NP-012602	0.121 ±0.002	31590 ±2725	0.885			
NP-012606	0.113 ±0.002	42600 ±3522	1.193			
NP-012609	0.120 ±0.006	31225 ±1933	0.874			
NP-012611	0.125 ±0.005	55494 ±3218	1.554			
NP-012612	0.108 ±0.004	54186 ±3804	1.517			
NP-012613	0.126 ±0.010	33270 ±791	0.932			
NP-012614	0.117 ±0.003	44678 ±8323	1.251			
NP-012615	0.111 ±0.002	34244 ±1064	0.959			
NP-012616	0.117 ±0.002	41525 ±8528	1.163			
NP-012634	0.112 ±0.004	34936 ±6853	0.978			
NP-012637	0.120 ±0.008	34002 ±631	0.952			
NP-012642	0.116 ±0.003	46626 ±641	1.306			
NP-012643	0.120 ±0.006	62207 ±5621	1.666			
NP-012644	0.118 ±0.007	47933 ±1429	1.342			
NP-012645	0.118 ±0.007	58059 ±6869	1.626			
NP-012648	0.113 ±0.002	98216 ±3796	2.630	0.115 ±0.002	44165 ±6408	1.351
NP-012649	0.126 ±0.004	65912 ±8425	1.765			
NP-012650	0.108 ±0.002	44066 ±8073	1.234			
NP-012654	0.106 ±0.004	47890 ±805	1.341			
NP-012659	0.109 ±0.003	44620 ±547	1.249			
NP-012662	0.129 ±0.002	50848 ±7908	1.424			
NP-012664	0.124 ±0.003	69607 ±7281	1.864			
NP-012665	0.124 ±0.002	50452 ±3164	1.413			
NP-012666	0.123 ±0.002	34210 ±1611	0.958			

NP-012667	0.120 ± 0.005	35949 ± 6806	1.007			
NP-012672	0.116 ± 0.006	28792 ± 2536	0.806			
NP-012678	0.126 ± 0.007	32730 ± 2032	0.916			
NP-012682	0.128 ± 0.005	45900 ± 3793	1.285			
NP-012688	0.122 ± 0.007	22519 ± 1507	0.631			
NP-012692	0.116 ± 0.004	39953 ± 577	1.119			
NP-012697	0.115 ± 0.002	37467 ± 4115	1.049			
NP-012701	0.122 ± 0.003	41578 ± 1441	1.164			
NP-012702	0.136 ± 0.002	20037 ± 611	0.536			
NP-012707	0.112 ± 0.007	35448 ± 1930	0.993			
NP-012708	0.118 ± 0.006	38406 ± 6844	1.075			
NP-012709	0.126 ± 0.008	57245 ± 3229	1.533			
NP-012718	0.112 ± 0.004	35246 ± 1332	0.987			
NP-012719	0.126 ± 0.006	42357 ± 6448	1.186			
NP-012722	0.118 ± 0.008	30984 ± 1051	0.868			
NP-012725	0.124 ± 0.007	42161 ± 5995	1.180			
NP-012727	0.111 ± 0.004	41303 ± 2451	1.156			
NP-012729	0.114 ± 0.004	32840 ± 5028	0.920			
NP-012730	0.115 ± 0.017	35228 ± 837	0.986			
NP-012738	0.123 ± 0.006	43832 ± 1005	1.227			
NP-012757	0.110 ± 0.004	41450 ± 3663	1.161			
NP-012759	0.124 ± 0.002	32884 ± 2771	0.921			
NP-012764	0.116 ± 0.002	28939 ± 1547	0.810			
NP-012765	0.118 ± 0.010	40237 ± 2828	1.127			
NP-012775	0.107 ± 0.007	43607 ± 5011	1.221			
NP-012776	0.119 ± 0.006	38880 ± 3066	1.089			
NP-012777	0.108 ± 0.005	49022 ± 1852	1.373			
NP-012778	0.125 ± 0.001	34244 ± 3421	0.959			
NP-012781	0.121 ± 0.005	30496 ± 1499	0.854			
NP-012782	0.120 ± 0.007	40830 ± 4242	1.143			
NP-012785	0.116 ± 0.002	36970 ± 2092	1.035			
NP-012788	0.118 ± 0.002	44675 ± 3316	1.251			

NP-012791	0.120 ±0.003	43026 ±1976	1.205			
NP-012795	0.127 ±0.005	49096 ±4078	1.375			
NP-012796	0.122 ±0.001	48012 ±6906	1.344			
NP-012797	0.119 ±0.004	26315 ±4185	0.737			
NP-012798	0.118 ±0.004	42037 ±846	1.177			
NP-012799	0.117 ±0.007	32999 ±5551	0.924			
NP-012800	0.122 ±0.002	48142 ±3946	1.348			
NP-012802	0.113 ±0.006	43364 ±6592	1.214			
NP-012804	0.122 ±0.001	33219 ±1171	0.930			
NP-012805	0.133 ±0.007	38458 ±3775	1.077			
NP-012810	0.121 ±0.003	42398 ±2348	1.187			
NP-012812	0.115 ±0.004	28266 ±4791	0.791			
NP-012815	0.113 ±0.004	45425 ±3150	1.272			
NP-012817	0.125 ±0.003	39741 ±3525	1.113			
NP-012818	0.116 ±0.003	61301 ±5623	1.641			
NP-012822	0.114 ±0.007	37417 ±2742	1.048			
NP-012823	0.112 ±0.003	46026 ±4333	1.289			
NP-012825	0.120 ±0.003	51199 ±7280	1.434			
NP-012826	0.118 ±0.018	29750 ±8932	0.833			
NP-012827	0.109 ±0.006	26238 ±2944	0.735			
NP-012831	0.116 ±0.002	37898 ±2821	1.061			
NP-012832	0.118 ±0.006	39727 ±1338	1.112			
NP-012833	0.115 ±0.004	41139 ±2948	1.152			
NP-012835	0.121 ±0.005	42828 ±5943	1.199			
NP-012836	0.134 ±0.010	40020 ±4957	1.121			
NP-012844	0.119 ±0.003	36213 ±2129	1.014			
NP-012845	0.115 ±0.005	40799 ±7999	1.142			
NP-012851	0.124 ±0.005	52305 ±2396	1.465			
NP-012854	0.118 ±0.004	50908 ±6389	1.425			
NP-012855	0.119 ±0.004	54576 ±9779	1.528			
NP-012856	0.121 ±0.005	41818 ±963	1.171			
NP-012858	0.122 ±0.008	37037 ±797	1.037			

NP-012860	0.127 ±0.004	34575 ±1795	0.968			
NP-012861	0.115 ±0.005	56257 ±7132	1.575			
NP-012863	0.120 ±0.008	35088 ±5151	0.982			
NP-012868	0.116 ±0.003	40713 ±1374	1.140			
NP-012870	0.118 ±0.003	36726 ±2883	1.028			
NP-012878	0.118 ±0.006	55418 ±6955	1.552			
NP-012879	0.118 ±0.003	39382 ±5563	1.103			
NP-012880	0.115 ±0.005	38645 ±895	1.082			
NP-012882	0.110 ±0.009	30108 ±1482	0.843			
NP-012883	0.096 ±0.005 *					
NP-012884	0.111 ±0.002	38940 ±1403	1.090			
NP-012889	0.102 ±0.009	36655 ±2925	1.026			
NP-012890	0.106 ±0.003	46246 ±3240	1.295			
NP-012894	0.118 ±0.012	24144 ±1026	0.676			
NP-012899	0.130 ±0.009	51186 ±823	1.433			
NP-012901	0.110 ±0.022	25891 ±2007	0.725			
NP-012905	0.112 ±0.002	37215 ±1933	1.042			
NP-012906	0.121 ±0.006	48225 ±2938	1.350			
NP-012908	0.107 ±0.005	43117 ±2855	1.207			
NP-012909	0.110 ±0.002	48405 ±984	1.355			
NP-012910	0.113 ±0.001	52087 ±2717	1.458			
NP-012912	0.110 ±0.004	29175 ±2037	0.817			
NP-012913	0.109 ±0.005	70487 ±7779	1.887			
NP-012915	0.117 ±0.007	36483 ±1189	1.022			
NP-012916	0.113 ±0.005	56221 ±4074	1.505			
NP-012918	0.121 ±0.012	49308 ±6296	1.381			
NP-012919	0.111 ±0.002	29080 ±5852	0.814			
NP-012920	0.105 ±0.005	32861 ±3788	0.920			
NP-012921	0.113 ±0.002	58861 ±1730	1.576			
NP-012922	0.112 ±0.005	57885 ±4109	1.550			
NP-012923	0.119 ±0.003	40889 ±2449	1.145			
NP-012924	0.117 ±0.008	22382 ±6286	0.627			

NP-012925	0.117 ± 0.004	37251 ± 1964	1.043			
NP-012926	0.113 ± 0.003	40901 ± 5919	1.145			
NP-012930	0.118 ± 0.003	33099 ± 3742	0.927			
NP-012932	0.119 ± 0.004	44296 ± 586	1.240			
NP-012934	0.117 ± 0.005	35960 ± 504	1.007			
NP-012938	0.115 ± 0.004	56882 ± 4106	1.593			
NP-012939	0.109 ± 0.003	38907 ± 5238	1.089			
NP-012940	0.119 ± 0.006	58551 ± 5517	1.639			
NP-012941	0.115 ± 0.003	46220 ± 872	1.294			
NP-012946	0.120 ± 0.001	66279 ± 6505	1.775			
NP-012947	0.132 ± 0.010	42764 ± 5015	1.197			
NP-012948	0.111 ± 0.002	42401 ± 3321	1.187			
NP-012949	0.123 ± 0.004	45145 ± 2691	1.264			
NP-012950	0.096 ± 0.005 *					
NP-012951	0.120 ± 0.003	43012 ± 2038	1.204			
NP-012952	0.121 ± 0.003	33632 ± 2825	0.942			
NP-012953	0.112 ± 0.003	32015 ± 1275	0.896			
NP-012954	0.116 ± 0.007	58433 ± 8149	1.636			
NP-012955	0.124 ± 0.005	20561 ± 1037	0.551			
NP-012956	0.120 ± 0.006	46238 ± 4209	1.295			
NP-012958	0.120 ± 0.003	47542 ± 419	1.331			
NP-012959	0.115 ± 0.004	52564 ± 3046	1.472			
NP-012960	0.115 ± 0.002	47801 ± 5648	1.338			
NP-012963	0.119 ± 0.005	37289 ± 1406	1.044			
NP-012964	0.113 ± 0.004	49992 ± 3754	1.400			
NP-012967	0.121 ± 0.003	42863 ± 1381	1.200			
NP-012970	0.114 ± 0.004	55005 ± 3198	1.473			
NP-012971	0.125 ± 0.009	42522 ± 4324	1.191			
NP-012972	0.119 ± 0.005	41801 ± 1590	1.170			
NP-012977	0.122 ± 0.003	33387 ± 2119	0.935			
NP-012978	0.113 ± 0.007	45686 ± 2424	1.279			
NP-012979	0.110 ± 0.010	35103 ± 1181	0.983			

NP-012981	0.118 ±0.003	37471 ±3511	1.049			
NP-012982	0.112 ±0.003	43221 ±2046	1.210			
NP-012983	0.120 ±0.002	41599 ±6146	1.165			
NP-012984	0.136 ±0.013	38765 ±2410	1.085			
NP-012985	0.120 ±0.006	41713 ±4388	1.168			
NP-012991	0.121 ±0.003	49273 ±7110	1.380			
NP-012992	0.116 ±0.003	49291 ±4264	1.380			
NP-012993	0.120 ±0.007	30780 ±4378	0.862			
NP-012994	0.118 ±0.005	40829 ±1266	1.143			
NP-012999	0.117 ±0.007	39002 ±1800	1.092			
NP-013004	0.122 ±0.006	38699 ±2717	1.084			
NP-013012	0.111 ±0.008	43476 ±3518	1.217			
NP-013013	0.117 ±0.002	41590 ±636	1.164			
NP-013014	0.112 ±0.003	47611 ±4084	1.333			
NP-013015	0.128 ±0.004	41999 ±3748	1.176			
NP-013016	0.113 ±0.005	43916 ±6153	1.230			
NP-013017	0.124 ±0.006	36999 ±4592	1.036			
NP-013019	0.109 ±0.008	41634 ±9925	1.166			
NP-013024	0.121 ±0.009	42746 ±1107	1.197			
NP-013026	0.120 ±0.007	42051 ±8190	1.177			
NP-013030	0.116 ±0.001	50962 ±1595	1.427			
NP-013032	0.128 ±0.013	39422 ±5701	1.104			
NP-013033	0.104 ±0.003	27604 ±4015	0.773			
NP-013034	0.134 ±0.013	60590 ±795	1.622			
NP-013036	0.115 ±0.007	49436 ±3648	1.384			
NP-013037	0.119 ±0.003	49253 ±7266	1.379			
NP-013038	0.121 ±0.007	32555 ±4492	0.912			
NP-013039	0.115 ±0.005	44573 ±2231	1.248			
NP-013040	0.111 ±0.007	38823 ±5240	1.087			
NP-013041	0.116 ±0.004	26424 ±5358	0.740			
NP-013046	0.117 ±0.006	36550 ±7302	1.023			
NP-013052	0.116 ±0.012	38939 ±2031	1.090			

NP-013055	0.123 ±0.008	41194 ±2474	1.153			
NP-013057	0.113 ±0.012	26101 ±4161	0.731			
NP-013058	0.109 ±0.007	43061 ±1100	1.206			
NP-013060	0.136 ±0.009	39581 ±3747	1.108			
NP-013061	0.066 ±0.004 **					
NP-013062	0.122 ±0.004	48534 ±3407	1.359			
NP-013064	0.115 ±0.004	48226 ±4250	1.350			
NP-013065	0.118 ±0.008	45354 ±2241	1.270			
NP-013067	0.130 ±0.007	35339 ±3610	0.989			
NP-013068	0.116 ±0.002	50421 ±101	1.412			
NP-013070	0.114 ±0.002	53731 ±3248	1.504			
NP-013073	0.114 ±0.004	42140 ±3237	1.180			
NP-013075	0.112 ±0.002	50119 ±6541	1.403			
NP-013079	0.113 ±0.003	48622 ±3483	1.361			
NP-013080	0.120 ±0.004	38412 ±2591	1.076			
NP-013089	0.126 ±0.003	44200 ±6552	1.238			
NP-013093	0.119 ±0.001	47128 ±6006	1.320			
NP-013094	0.111 ±0.005	42869 ±2498	1.200			
NP-013098	0.117 ±0.001	65066 ±10554	1.822			
NP-013099	0.112 ±0.002	31018 ±436	0.868			
NP-013100	0.118 ±0.005	50857 ±6350	1.424			
NP-013101	0.112 ±0.018	36020 ±3181	1.009			
NP-013104	0.112 ±0.007	36417 ±5301	1.020			
NP-013105	0.122 ±0.006	24735 ±4877	0.693			
NP-013107	0.112 ±0.004	37827 ±1858	1.059			
NP-013109	0.120 ±0.010	41480 ±2309	1.161			
NP-013110	0.122 ±0.006	41520 ±1428	1.163			
NP-013111	0.117 ±0.008	52381 ±2199	1.467			
NP-013112	0.112 ±0.001	42974 ±2206	1.203			
NP-013113	0.111 ±0.006	34767 ±6868	0.973			
NP-013114	0.112 ±0.003	50640 ±3145	1.418			
NP-013115	0.112 ±0.001	37890 ±3217	1.061			

NP-013117	0.113 ±0.006	27544 ±1432	0.771			
NP-013118	0.123 ±0.002	41048 ±4750	1.149			
NP-013119	0.119 ±0.007	35652 ±3115	0.998			
NP-013121	0.119 ±0.006	45760 ±1927	1.281			
NP-013123	0.118 ±0.003	33427 ±1711	0.936			
NP-013124	0.114 ±0.007	38965 ±1103	1.091			
NP-013125	0.112 ±0.017	43084 ±6366	1.206			
NP-013126	0.109 ±0.011	21460 ±217	0.575			
NP-013127	0.123 ±0.003	39453 ±3918	1.105			
NP-013129	0.113 ±0.006	32279 ±2334	0.904			
NP-013130	0.120 ±0.001	51690 ±5253	1.447			
NP-013133	0.118 ±0.002	41541 ±3815	1.163			
NP-013134	0.120 ±0.006	21244 ±2117	0.595			
NP-013135	0.122 ±0.008	28113 ±2825	0.787			
NP-013136	0.110 ±0.003	30258 ±1682	0.847			
NP-013137	0.112 ±0.004	31928 ±3085	0.894			
NP-013138	0.118 ±0.002	42158 ±2899	1.180			
NP-013140	0.113 ±0.005	36387 ±6609	1.019			
NP-013141	0.113 ±0.005	38688 ±5122	1.083			
NP-013146	0.109 ±0.004	48352 ±3474	1.354			
NP-013156	0.109 ±0.001	39047 ±6500	1.093			
NP-013157	0.116 ±0.003	23689 ±3050	0.663			
NP-013158	0.113 ±0.002	29974 ±244	0.839			
NP-013160	0.113 ±0.002	31274 ±5536	0.876			
NP-013161	0.113 ±0.004	34752 ±4819	0.973			
NP-013167	0.128 ±0.008	44451 ±4746	1.245			
NP-013168	0.112 ±0.004	39672 ±7480	1.111			
NP-013173	0.114 ±0.011	31623 ±4459	0.885			
NP-013174	0.117 ±0.007	34668 ±2670	0.971			
NP-013175	0.110 ±0.001	36167 ±3242	1.013			
NP-013176	0.114 ±0.008	34806 ±2139	0.975			
NP-013178	0.114 ±0.002	33262 ±2248	0.931			

NP-013180	0.121 ±0.005	39546 ±1378	1.107			
NP-013181	0.128 ±0.011	38217 ±4497	1.070			
NP-013182	0.116 ±0.005	39747 ±4069	1.113			
NP-013183	0.113 ±0.003	34455 ±5497	0.965			
NP-013184	0.114 ±0.007	36540 ±5769	1.023			
NP-013189	0.110 ±0.001	31104 ±5441	0.871			
NP-013191	0.122 ±0.012	31792 ±4283	0.890			
NP-013192	0.118 ±0.008	35853 ±5675	1.004			
NP-013193	0.112 ±0.006	39595 ±6650	1.109			
NP-013194	0.117 ±0.009	46002 ±4939	1.288			
NP-013199	0.122 ±0.002	42949 ±2312	1.203			
NP-013203	0.110 ±0.002	36408 ±4550	1.019			
NP-013206	0.121 ±0.003	56071 ±1473	1.501			
NP-013208	0.114 ±0.002	34881 ±301	0.977			
NP-013210	0.119 ±0.005	36380 ±2315	1.019			
NP-013211	0.123 ±0.003	42053 ±2401	1.177			
NP-013212	0.114 ±0.013	53471 ±3805	1.497			
NP-013213	0.125 ±0.008	43763 ±1044	1.225			
NP-013215	0.113 ±0.006	40732 ±2851	1.140			
NP-013217	0.121 ±0.006	45761 ±2050	1.281			
NP-013218	0.116 ±0.006	49276 ±2575	1.380			
NP-013219	0.125 ±0.006	57893 ±1430	1.550			
NP-013221	0.116 ±0.004	33262 ±2379	0.931			
NP-013222	0.123 ±0.001	38953 ±1057	1.091			
NP-013223	0.121 ±0.005	42290 ±3595	1.184			
NP-013225	0.121 ±0.010	25916 ±1179	0.726			
NP-013229	0.123 ±0.003	32319 ±2110	0.905			
NP-013231	0.127 ±0.004	32963 ±2337	0.923			
NP-013238	0.113 ±0.005	46190 ±4035	1.293			
NP-013239	0.113 ±0.010	40914 ±1975	1.146			
NP-013241	0.110 ±0.001	38945 ±3775	1.090			
NP-013242	0.116 ±0.008	22302 ±474	0.597			

NP-013247	0.112 ± 0.003	40455 ± 2975	1.133			
NP-013248	0.118 ± 0.004	55266 ± 2634	1.480			
NP-013251	0.114 ± 0.004	53376 ± 3639	1.494			
NP-013254	0.110 ± 0.002	50091 ± 6384	1.403			
NP-013259	0.111 ± 0.004	35400 ± 5987	0.991			
NP-013262	0.114 ± 0.007	49946 ± 7261	1.398			
NP-013263	0.118 ± 0.002	44981 ± 5640	1.259			
NP-013264	0.134 ± 0.014	29755 ± 1557	0.833			
NP-013265	0.124 ± 0.010	38231 ± 2477	1.070			
NP-013266	0.112 ± 0.007	38417 ± 1656	1.076			
NP-013267	0.113 ± 0.007	31575 ± 2258	0.884			
NP-013268	0.119 ± 0.001	42220 ± 2640	1.182			
NP-013272	0.131 ± 0.010	48219 ± 2169	1.350			
NP-013273	0.111 ± 0.005	36588 ± 2985	1.024			
NP-013274	0.117 ± 0.003	42174 ± 1520	1.181			
NP-013277	0.110 ± 0.005	25627 ± 2527	0.718			
NP-013281	0.125 ± 0.005	35460 ± 3383	0.993			
NP-013282	0.114 ± 0.004	34969 ± 1312	0.979			
NP-013285	0.113 ± 0.003	50592 ± 7954	1.417			
NP-013289	0.116 ± 0.003	41643 ± 4339	1.166			
NP-013291	0.129 ± 0.007	24678 ± 3124	0.691			
NP-013293	0.116 ± 0.002	32422 ± 354	0.908			
NP-013296	0.064 ± 0.009 **					
NP-013307	0.121 ± 0.009	35951 ± 4034	1.007			
NP-013312	0.116 ± 0.003	42023 ± 2411	1.177			
NP-013314	0.119 ± 0.011	36494 ± 2621	1.022			
NP-013315	0.110 ± 0.004	45528 ± 1535	1.275			
NP-013316	0.135 ± 0.017	38930 ± 3107	1.090			
NP-013318	0.115 ± 0.004	40334 ± 3032	1.129			
NP-013319	0.115 ± 0.002	37389 ± 3910	1.047			
NP-013320	0.114 ± 0.003	50556 ± 1453	1.416			
NP-013321	0.120 ± 0.007	39271 ± 1754	1.100			

NP-013323	0.113 ±0.003	40423 ±5778	1.132			
NP-013325	0.115 ±0.005	29269 ±2385	0.820			
NP-013326	0.109 ±0.003	34319 ±516	0.961			
NP-013330	0.112 ±0.001	45121 ±3389	1.263			
NP-013332	0.117 ±0.006	38320 ±4385	1.073			
NP-013334	0.108 ±0.002	53515 ±8150	1.498			
NP-013336	0.104 ±0.008	39513 ±2595	1.106			
NP-013337	0.117 ±0.003	53535 ±3983	1.499			
NP-013338	0.113 ±0.003	46486 ±8046	1.302			
NP-013339	0.119 ±0.007	43252 ±4520	1.211			
NP-013343	0.126 ±0.016	30202 ±4425	0.846			
NP-013344	0.117 ±0.002	39833 ±651	1.115			
NP-013345	0.115 ±0.004	40823 ±4975	1.143			
NP-013349	0.111 ±0.004	52723 ±6520	1.476			
NP-013351	0.119 ±0.005	38595 ±6683	1.081			
NP-013352	0.111 ±0.002	33576 ±5616	0.940			
NP-013354	0.099 ±0.006	62754 ±9007	1.757			
NP-013357	0.114 ±0.004	45635 ±974	1.278			
NP-013359	0.115 ±0.006	19460 ±2425	0.545			
NP-013366	0.108 ±0.005	55405 ±1313	1.483			
NP-013367	0.112 ±0.005	31526 ±2304	0.883			
NP-013369	0.117 ±0.005	63245 ±4065	1.693			
NP-013370	0.112 ±0.020	36017 ±5174	1.008			
NP-013373	0.116 ±0.005	22645 ±628	0.606			
NP-013378	0.106 ±0.005	58905 ±8687	1.649			
NP-013379	0.113 ±0.004	46543 ±4152	1.303			
NP-013381	0.117 ±0.010	43545 ±4706	1.219			
NP-013386	0.113 ±0.000	38912 ±3591	1.090			
NP-013387	0.114 ±0.005	31350 ±1086	0.878			
NP-013388	0.118 ±0.004	40843 ±2445	1.144			
NP-013391	0.097 ±0.003 *					
NP-013397	0.113 ±0.005	30675 ±3288	0.859			

NP-013398	0.109 ±0.005	43448 ±1470	1.217			
NP-013399	0.112 ±0.002	46980 ±9006	1.315			
NP-013400	0.110 ±0.001	48883 ±493	1.369			
NP-013403	0.114 ±0.005	49074 ±3238	1.374			
NP-013412	0.122 ±0.006	39563 ±2384	1.108			
NP-013413	0.112 ±0.003	42742 ±3585	1.197			
NP-013424	0.126 ±0.006	39652 ±4051	1.110			
NP-013425	0.119 ±0.003	37524 ±2727	1.051			
NP-013426	0.114 ±0.011	61328 ±4587	1.642			
NP-013428	0.120 ±0.007	37471 ±1644	1.049			
NP-013433	0.113 ±0.004	51271 ±3774	1.436			
NP-013434	0.115 ±0.003	43842 ±2306	1.228			
NP-013435	0.129 ±0.009	38419 ±1065	1.076			
NP-013436	0.112 ±0.002	37200 ±3099	1.042			
NP-013437	0.118 ±0.002	34173 ±2309	0.957			
NP-013440	0.114 ±0.007	36548 ±7061	1.023			
NP-013443	0.109 ±0.001	39610 ±3741	1.109			
NP-013445	0.114 ±0.002	49046 ±4672	1.373			
NP-013447	0.117 ±0.009	36261 ±6832	1.015			
NP-013451	0.108 ±0.002	38612 ±2557	1.081			
NP-013453	0.112 ±0.008	39553 ±4788	1.107			
NP-013454	0.114 ±0.003	50899 ±1093	1.425			
NP-013456	0.115 ±0.015	29433 ±696	0.824			
NP-013460	0.124 ±0.008	53996 ±8291	1.512			
NP-013470	0.115 ±0.003	31142 ±4811	0.872			
NP-013477	0.111 ±0.003	40890 ±3342	1.145			
NP-013478	0.120 ±0.003	23990 ±2397	0.672			
NP-013488	0.112 ±0.005	64833 ±6245	1.736			
NP-013495	0.115 ±0.007	52860 ±2432	1.480			
NP-013499	0.107 ±0.002	22491 ±873	0.630			
NP-013500	0.110 ±0.008	57825 ±2704	1.548			
NP-013504	0.120 ±0.004	53708 ±4158	1.504			

NP-013507	0.116 ±0.001	39348 ±1882	1.102			
NP-013508	0.117 ±0.005	39584 ±306	1.108			
NP-013513	0.119 ±0.004	31179 ±3490	0.873			
NP-013514	0.112 ±0.002	53143 ±4272	1.488			
NP-013515	0.115 ±0.003	26780 ±6903	0.750			
NP-013518	0.120 ±0.013	36841 ±4710	1.032			
NP-013520	0.116 ±0.004	26276 ±6112	0.736			
NP-013521	0.122 ±0.002	36611 ±1580	1.025			
NP-013522	0.119 ±0.003	51104 ±2342	1.431			
NP-013524	0.119 ±0.002	50896 ±3540	1.425			
NP-013525	0.113 ±0.009	44011 ±1363	1.232			
NP-013526	0.117 ±0.008	30008 ±4258	0.840			
NP-013527	0.114 ±0.005	26841 ±8813	0.752			
NP-013534	0.114 ±0.001	43277 ±3536	1.212			
NP-013535	0.120 ±0.002	49533 ±1640	1.387			
NP-013536	0.109 ±0.003	43484 ±2438	1.218			
NP-013537	0.122 ±0.004	30614 ±2179	0.857			
NP-013539	0.115 ±0.001	42053 ±2274	1.177			
NP-013541	0.111 ±0.004	38596 ±8036	1.081			
NP-013544	0.121 ±0.004	62226 ±8029	1.742			
NP-013545	0.111 ±0.003	46343 ±3653	1.298			
NP-013546	0.115 ±0.001	44863 ±3588	1.256			
NP-013547	0.130 ±0.010	50751 ±1356	1.421			
NP-013551	0.123 ±0.018	44045 ±4246	1.233			
NP-013552	0.114 ±0.002	30673 ±3776	0.859			
NP-013553	0.115 ±0.001	42597 ±2427	1.193			
NP-013554	0.117 ±0.011	36191 ±373	1.013			
NP-013555	0.118 ±0.012	51499 ±3659	1.442			
NP-013558	0.108 ±0.009	33556 ±389	0.940			
NP-013560	0.113 ±0.003	40054 ±3431	1.121			
NP-013562	0.111 ±0.004	34576 ±2768	0.968			
NP-013563	0.114 ±0.003	46832 ±4994	1.311			

NP-013564	0.108 ±0.008	46897 ±1732	1.313			
NP-013565	0.116 ±0.003	38348 ±3895	1.074			
NP-013567	0.125 ±0.003	36575 ±2581	1.024			
NP-013568	0.113 ±0.010	40089 ±9908	1.122			
NP-013571	0.120 ±0.006	71191 ±4053	1.906			
NP-013572	0.120 ±0.004	44964 ±5526	1.259			
NP-013574	0.127 ±0.003	39969 ±2521	1.119			
NP-013575	0.116 ±0.003	41344 ±1105	1.158			
NP-013580	0.117 ±0.005	48985 ±3034	1.372			
NP-013595	0.111 ±0.003	63644 ±2707	1.704			
NP-013597	0.120 ±0.001	44898 ±2083	1.257			
NP-013601	0.119 ±0.002	38955 ±6555	1.091			
NP-013634	0.120 ±0.003	45797 ±1208	1.282			
NP-013635	0.119 ±0.008	49524 ±2090	1.387			
NP-013654	0.109 ±0.004	33304 ±1420	0.932			
NP-013656	0.109 ±0.001	32097 ±5764	0.899			
NP-013657	0.113 ±0.003	36108 ±7660	1.011			
NP-013663	0.119 ±0.007	46786 ±4174	1.310			
NP-013664	0.118 ±0.002	40889 ±2019	1.145			
NP-013674	0.110 ±0.003	43324 ±2381	1.213			
NP-013681	0.118 ±0.003	45476 ±150	1.273			
NP-013682	0.120 ±0.004	38817 ±1585	1.087			
NP-013683	0.117 ±0.003	20026 ±581	0.536			
NP-013685	0.115 ±0.004	37839 ±9192	1.059			
NP-013686	0.114 ±0.007	51535 ±4238	1.443			
NP-013687	0.114 ±0.006	44582 ±3095	1.248			
NP-013688	0.126 ±0.003	40219 ±1682	1.126			
NP-013691	0.124 ±0.006	9281 ±550	0.249	0.116 ±0.008	24611 ±3573	0.753
NP-013693	0.101 ±0.012	35717 ±1071	1.000			
NP-013694	0.119 ±0.006	43785 ±1542	1.226			
NP-013699	0.124 ±0.003	56739 ±4802	1.589			
NP-013700	0.124 ±0.002	50839 ±2909	1.423			

NP-013701	0.115 ±0.004	49305 ±4755	1.381			
NP-013702	0.123 ±0.011	44206 ±6310	1.238			
NP-013703	0.117 ±0.002	35379 ±2474	0.991			
NP-013705	0.123 ±0.003	27711 ±1731	0.776			
NP-013713	0.115 ±0.002	38292 ±2687	1.072			
NP-013716	0.115 ±0.005	57026 ±4119	1.527			
NP-013717	0.127 ±0.005	27743 ±2943	0.777			
NP-013718	0.113 ±0.002	63325 ±4219	1.696			
NP-013719	0.122 ±0.004	32487 ±3732	0.910			
NP-013720	0.115 ±0.003	44694 ±325	1.251			
NP-013723	0.112 ±0.003	52644 ±4044	1.474			
NP-013725	0.117 ±0.003	30439 ±1679	0.852			
NP-013736	0.119 ±0.001	57665 ±2766	1.544			
NP-013744	0.117 ±0.006	29537 ±3704	0.827			
NP-013745	0.109 ±0.006	48872 ±5528	1.368			
NP-013746	0.114 ±0.005	36064 ±1441	1.010			
NP-013747	0.115 ±0.009	38548 ±5797	1.079			
NP-013749	0.120 ±0.006	40381 ±885	1.131			
NP-013751	0.109 ±0.002	48215 ±2482	1.350			
NP-013760	0.110 ±0.002	36471 ±6780	1.021			
NP-013761	0.115 ±0.004	49548 ±3351	1.387			
NP-013767	0.114 ±0.006	39466 ±6338	1.105			
NP-013768	0.120 ±0.006	35807 ±1113	1.003			
NP-013770	0.117 ±0.006	37955 ±7882	1.063			
NP-013773	0.119 ±0.003	30597 ±6075	0.857			
NP-013774	0.123 ±0.004	44758 ±5358	1.253			
NP-013779	0.124 ±0.006	25074 ±5363	0.702			
NP-013780	0.109 ±0.003	44256 ±1520	1.239			
NP-013805	0.125 ±0.007	45076 ±4818	1.262			
NP-013808	0.128 ±0.010	28358 ±5067	0.794			
NP-013845	0.118 ±0.002	38132 ±2010	1.068			
NP-013854	0.115 ±0.006	24822 ±765	0.695			

NP-013874	0.125 ±0.011	27239 ±2882	0.763			
NP-013932	0.133 ±0.009	35828 ±1520	1.003			
NP-013935	0.125 ±0.011	34785 ±4268	0.974			
NP-013989	0.117 ±0.002	40720 ±4870	1.140			
NP-013990	0.118 ±0.002	32580 ±1780	0.912			
NP-014048	0.136 ±0.010	56279 ±6431	1.576			
NP-014097	0.117 ±0.004	28925 ±3473	0.810			
NP-014100	0.116 ±0.015	31175 ±536	0.873			
NP-014103	0.116 ±0.003	34282 ±2835	0.960			
NP-014109	0.116 ±0.005	41665 ±2401	1.167			
NP-014114	0.114 ±0.008	43369 ±1639	1.214			
NP-014122	0.115 ±0.011	20578 ±392	0.551			
NP-014123	0.116 ±0.002	35659 ±1317	0.998			
NP-014130	0.129 ±0.017	31086 ±2275	0.870			
NP-014133	0.111 ±0.002	43058 ±1066	1.206			
NP-014140	0.121 ±0.008	36100 ±5075	1.011			
NP-014141	0.121 ±0.005	28646 ±1074	0.802			
NP-014144	0.115 ±0.005	39646 ±2144	1.110			
NP-014152	0.115 ±0.016	39752 ±3077	1.113			
NP-014156	0.117 ±0.005	23509 ±221	0.658			
NP-014194	0.119 ±0.005	42126 ±5123	1.180			
NP-014211	0.081 ±0.007 **					
NP-014212	0.115 ±0.002	42674 ±2517	1.195			
NP-014213	0.121 ±0.008	49721 ±7785	1.392			
NP-014222	0.114 ±0.005	60330 ±5742	1.615			
NP-014225	0.120 ±0.007	32480 ±2060	0.909			
NP-014228	0.102 ±0.009	34865 ±3992	0.976			
NP-014250	0.116 ±0.008	36714 ±1379	1.028			
NP-014282	0.117 ±0.002	22542 ±4738	0.631			
NP-014284	0.116 ±0.004	45762 ±3742	1.281			
NP-014286	0.116 ±0.003	33467 ±1840	0.937			
NP-014288	0.116 ±0.004	38826 ±3289	1.087			

NP-014290	0.112 ± 0.002	61734 ± 1194	1.653			
NP-014291	0.116 ± 0.002	54992 ± 766	1.472			
NP-014294	0.112 ± 0.003	66311 ± 3945	1.775			
NP-014295	0.131 ± 0.013	23370 ± 1767	0.654			
NP-014298	0.115 ± 0.002	47078 ± 4412	1.318			
NP-014300	0.112 ± 0.003	47437 ± 3311	1.328			
NP-014301	0.118 ± 0.005	27767 ± 972	0.777			
NP-014322	0.102 ± 0.004	95138 ± 6497	2.547	0.105 ± 0.009	142577 ± 6414	4.363
NP-014325	0.116 ± 0.006	36061 ± 2636	1.010			
NP-014331	0.124 ± 0.002	36149 ± 1268	1.012			
NP-014335	0.121 ± 0.003	37532 ± 3113	1.051			
NP-014336	0.117 ± 0.006	37166 ± 2875	1.041			
NP-014338	0.124 ± 0.003	46836 ± 1600	1.311			
NP-014339	0.123 ± 0.001	28402 ± 371	0.795			
NP-014341	0.121 ± 0.002	31629 ± 601	0.886			
NP-014345	0.118 ± 0.003	35683 ± 2711	0.999			
NP-014346	0.118 ± 0.004	28844 ± 824	0.808			
NP-014347	0.124 ± 0.009	19294 ± 860	0.517			
NP-014348	0.117 ± 0.004	36222 ± 5049	1.014			
NP-014374	0.135 ± 0.014	32110 ± 3278	0.899			
NP-014387	0.119 ± 0.008	44739 ± 742	1.253			
NP-014424	0.114 ± 0.005	34390 ± 3347	0.963			
NP-014425	0.121 ± 0.003	26182 ± 4198	0.733			
NP-014427	0.112 ± 0.014	27534 ± 2690	0.771			
NP-014428	0.104 ± 0.006	31257 ± 4570	0.875			
NP-014429	0.128 ± 0.019	9258 ± 896	0.248	0.112 ± 0.012	27365 ± 1689	0.837
NP-014430	0.110 ± 0.003	34480 ± 2812	0.965			
NP-014431	0.110 ± 0.010	24459 ± 1435	0.685			
NP-014434	0.131 ± 0.009	36929 ± 2778	1.034			
NP-014435	0.119 ± 0.007	20110 ± 540	0.538			
NP-014436	0.119 ± 0.002	34103 ± 2216	0.955			
NP-014463	0.127 ± 0.007	52862 ± 5448	1.480			

NP-014464	0.111 ±0.001	37225 ±1246	1.042			
NP-014465	0.117 ±0.009	33465 ±2091	0.937			
NP-014466	0.123 ±0.008	52539 ±4804	1.407			
NP-014467	0.121 ±0.003	35927 ±1771	1.006			
NP-014470	0.121 ±0.008	39940 ±3046	1.118			
NP-014472	0.120 ±0.005	41439 ±3500	1.160			
NP-014473	0.122 ±0.003	38504 ±772	1.078			
NP-014477	0.121 ±0.002	32053 ±1066	0.897			
NP-014480	0.117 ±0.004	42923 ±3714	1.202			
NP-014481	0.128 ±0.001	39917 ±4575	1.118			
NP-014484	0.111 ±0.003	41282 ±4264	1.156			
NP-014485	0.110 ±0.001	36040 ±2312	1.009			
NP-014486	0.118 ±0.006	30201 ±2910	0.846			
NP-014495	0.118 ±0.003	38382 ±2247	1.075			
NP-014497	0.120 ±0.002	64205 ±2934	1.719			
NP-014499	0.123 ±0.001	50413 ±5101	1.412			
NP-014505	0.129 ±0.006	40169 ±1840	1.125			
NP-014507	0.106 ±0.005	21768 ±2749	0.583			
NP-014508	0.137 ±0.013	58497 ±4220	1.566			
NP-014511	0.120 ±0.006	45416 ±3193	1.272			
NP-014513	0.119 ±0.002	66358 ±4344	1.777			
NP-014514	0.120 ±0.019	34212 ±2501	0.958			
NP-014526	0.115 ±0.002	37559 ±5603	1.052			
NP-014527	0.114 ±0.002	43373 ±8403	1.214			
NP-014529	0.113 ±0.003	39046 ±6637	1.093			
NP-014534	0.106 ±0.002	38472 ±6013	1.077			
NP-014535	0.108 ±0.004	39414 ±4692	1.104			
NP-014539	0.108 ±0.003	39803 ±6440	1.114			
NP-014545	0.109 ±0.001	49456 ±5399	1.385			
NP-014548	0.118 ±0.009	21658 ±2501	0.580			
NP-014549	0.122 ±0.009	36280 ±5274	1.016			
NP-014553	0.113 ±0.007	50712 ±7304	1.420			

NP-014566	0.115 ± 0.004	52215 ± 1435	1.398			
NP-014576	0.109 ± 0.004	42540 ± 6623	1.191			
NP-014584	0.114 ± 0.003	48456 ± 5776	1.357			
NP-014585	0.110 ± 0.001	12943 ± 1205	0.347	0.108 ± 0.008	13921 ± 520	0.426
NP-014590	0.120 ± 0.001	40191 ± 2516	1.125			
NP-014591	0.117 ± 0.013	36302 ± 2638	1.016			
NP-014593	0.116 ± 0.005	44688 ± 3162	1.251			
NP-014595	0.115 ± 0.002	47365 ± 3501	1.326			
NP-014597	0.121 ± 0.004	53323 ± 3584	1.493			
NP-014598	0.116 ± 0.003	35077 ± 1549	0.982			
NP-014599	0.110 ± 0.003	45613 ± 4578	1.277			
NP-014600	0.087 ± 0.005 **					
NP-014603	0.136 ± 0.007	47919 ± 4507	1.342			
NP-014613	0.124 ± 0.009	33795 ± 4549	0.946			
NP-014621	0.111 ± 0.018	30650 ± 1501	0.858			
NP-014627	0.125 ± 0.005	36536 ± 1400	1.023			
NP-014630	0.116 ± 0.001	58760 ± 3495	1.573			
NP-014631	0.119 ± 0.011	66805 ± 3425	1.789			
NP-014648	0.119 ± 0.008	45562 ± 1924	1.276			
NP-014653	0.103 ± 0.009	36124 ± 4661	1.011			
NP-014654	0.122 ± 0.013	41758 ± 3509	1.169			
NP-014655	0.097 ± 0.002 *					
NP-014665	0.122 ± 0.008	50611 ± 9254	1.417			
NP-014693	0.117 ± 0.005	47098 ± 2271	1.319			
NP-014695	0.137 ± 0.006	35022 ± 3090	0.981			
NP-014696	0.118 ± 0.004	47839 ± 6519	1.339			
NP-014756	0.119 ± 0.005	36949 ± 2589	1.035			
NP-014766	0.115 ± 0.004	57124 ± 2439	1.599			
NP-014782	0.112 ± 0.002	52021 ± 2543	1.457			
NP-014783	0.122 ± 0.004	32741 ± 8637	0.917			
NP-014793	0.122 ± 0.005	41519 ± 9814	1.163			
NP-014796	0.119 ± 0.006	49487 ± 6808	1.386			

	NP-014797	0.123 ± 0.007	51832 ± 2171	1.451			
	NP-014821	0.128 ± 0.007	39518 ± 3776	1.106			
	NP-014829	0.097 ± 0.005 *					
	NP-014830	0.114 ± 0.011	43178 ± 3917	1.209			
	NP-014834	0.110 ± 0.013	26402 ± 5867	0.739			
	NP-014835	0.119 ± 0.005	28074 ± 1177	0.786			
	NP-014837	0.098 ± 0.001 *					
	NP-014838	0.120 ± 0.002	53904 ± 1824	1.509			
	NP-014839	0.116 ± 0.007	42727 ± 3116	1.196			
	NP-014841	0.120 ± 0.001	58513 ± 9285	1.638			
	NP-014850	0.120 ± 0.001	32619 ± 3094	0.913			
	NP-014857	0.131 ± 0.009	41048 ± 5286	1.149			
	NP-014860	0.113 ± 0.002	27540 ± 4195	0.771			
	NP-014861	0.116 ± 0.004	38329 ± 4083	1.073			
	NP-014869	0.119 ± 0.003	63974 ± 7418	1.713			
	NP-014870	0.111 ± 0.004	42482 ± 2342	1.189			
	NP-014895	0.122 ± 0.005	44193 ± 1026	1.237			
	NP-014897	0.117 ± 0.003	31451 ± 1825	0.881			
	NP-014899	0.113 ± 0.003	43302 ± 2211	1.212			
	NP-014902	0.117 ± 0.007	38033 ± 2982	1.065			
	NP-014903	0.116 ± 0.003	50404 ± 5415	1.411			
	NP-014904	0.115 ± 0.001	37727 ± 474	1.056			
	NP-014906	0.117 ± 0.001	46193 ± 2011	1.293			
	NP-014907	0.104 ± 0.015	41312 ± 4550	1.157			
	NP-014909	0.119 ± 0.004	48598 ± 4242	1.361			
	NP-014910	0.117 ± 0.004	48830 ± 7815	1.367			
	NP-014911	0.115 ± 0.002	33244 ± 7672	0.931			
	NP-014920	0.112 ± 0.004	34192 ± 2274	0.957			
	NP-014921	0.113 ± 0.009	35385 ± 2467	0.991			
	NP-014922	0.117 ± 0.013	37277 ± 4656	1.044			
	NP-014934	0.114 ± 0.004	35922 ± 1702	1.006			
	NP-014935	0.111 ± 0.003	41350 ± 6013	1.158			

NP-014936	0.114 ± 0.000	40854 ± 1541	1.144			
NP-014937	0.118 ± 0.009	31281 ± 2268	0.876			
NP-014938	0.113 ± 0.009	43957 ± 9763	1.231			
NP-014966	0.124 ± 0.003	51234 ± 1312	1.435			
NP-014967	0.126 ± 0.004	53511 ± 6186	1.498			
NP-014979	0.129 ± 0.004	41416 ± 3627	1.160			
NP-014980	0.122 ± 0.008	29471 ± 2258	0.825			
NP-014981	0.122 ± 0.006	49680 ± 1202	1.391			
NP-014984	0.120 ± 0.001	21763 ± 2891	0.609			
NP-014986	0.115 ± 0.006	31710 ± 4247	0.888			
NP-014987	0.117 ± 0.005	43908 ± 5066	1.229			
NP-014997	0.116 ± 0.003	30613 ± 6307	0.857			
NP-014998	0.113 ± 0.006	49430 ± 9006	1.384			
NP-014999	0.116 ± 0.003	49709 ± 2589	1.392			
NP-015001	0.122 ± 0.006	31848 ± 5783	0.892			
NP-015002	0.112 ± 0.008	35759 ± 2230	1.001			
NP-015003	0.116 ± 0.003	46192 ± 9368	1.293			
NP-015006	0.123 ± 0.008	34948 ± 4851	0.979			
NP-015009	0.121 ± 0.004	40391 ± 5926	1.131			
NP-015010	0.115 ± 0.004	32960 ± 8471	0.923			
NP-015011	0.124 ± 0.002	45190 ± 3948	1.265			
NP-015012	0.124 ± 0.005	30882 ± 1366	0.865			
NP-015014	0.111 ± 0.006	33491 ± 4527	0.938			
NP-015019	0.110 ± 0.001	26865 ± 6883	0.752			
NP-015023	0.118 ± 0.006	40991 ± 6388	1.148			
NP-015044	0.105 ± 0.010	34927 ± 2941	0.978			
NP-015045	0.134 ± 0.011	34412 ± 1397	0.964			
NP-015046	0.107 ± 0.002	34570 ± 2045	0.968			
NP-015047	0.106 ± 0.004	41362 ± 5750	1.158			
NP-015048	0.107 ± 0.006	34532 ± 2451	0.967			
NP-015049	0.126 ± 0.008	39107 ± 3306	1.095			
NP-015050	0.126 ± 0.010	31119 ± 2375	0.871			

NP-015051	0.126 ±0.006	40195 ±4435	1.125			
NP-015052	0.107 ±0.004	44594 ±2374	1.249			
NP-015053	0.106 ±0.007	28191 ±1950	0.789			
NP-015055	0.111 ±0.005	34980 ±1445	0.979			
NP-015056	0.120 ±0.007	24536 ±1348	0.687			
NP-015057	0.124 ±0.003	48900 ±2730	1.369			
NP-015062	0.121 ±0.003	39531 ±1563	1.107			
NP-015063	0.132 ±0.006	53655 ±1656	1.437			
NP-015064	0.114 ±0.005	37275 ±1488	1.044			
NP-015065	0.125 ±0.005	46144 ±3540	1.292			
NP-015066	0.125 ±0.003	43383 ±2916	1.215			
NP-015069	0.131 ±0.008	40977 ±2367	1.147			
NP-015072	0.118 ±0.001	61782 ±1652	1.654			
NP-015073	0.124 ±0.017	66095 ±4445	1.770			
NP-015074	0.121 ±0.002	54105 ±4316	1.515			
NP-015078	0.121 ±0.005	50147 ±5707	1.404			
NP-015083	0.116 ±0.006	49894 ±2435	1.397			
NP-015088	0.117 ±0.003	29616 ±501	0.829			
NP-015089	0.122 ±0.004	27053 ±3423	0.757			
NP-015113	0.119 ±0.003	38197 ±2129	1.069			
NP-015114	0.120 ±0.002	26482 ±1503	0.741			
NP-015115	0.120 ±0.002	36450 ±2459	1.021			
NP-015116	0.119 ±0.001	50653 ±2207	1.418			
NP-015117	0.125 ±0.006	31162 ±837	0.873			
NP-015118	0.130 ±0.008	46556 ±8627	1.304			
NP-015119	0.126 ±0.005	30142 ±388	0.844			
NP-015121	0.118 ±0.002	33742 ±607	0.945			
NP-015122	0.110 ±0.013	53878 ±2849	1.509			
NP-015123	0.137 ±0.010	27395 ±628	0.767			
NP-015124	0.112 ±0.002	53862 ±1858	1.442			
NP-015125	0.115 ±0.009	35888 ±1949	1.005			
NP-015126	0.135 ±0.019	38746 ±4632	1.085			

NP-015127	0.115 ±0.002	39729 ±4552	1.112			
NP-015129	0.121 ±0.004	42539 ±1884	1.191			
NP-015134	0.124 ±0.006	37546 ±3339	1.051			
NP-015136	0.121 ±0.001	48672 ±2998	1.363			
NP-015137	0.119 ±0.004	30156 ±1259	0.844			
NP-015139	0.121 ±0.003	47423 ±2820	1.328			
NP-015140	0.116 ±0.003	27742 ±1064	0.777			
NP-015141	0.119 ±0.001	39336 ±1507	1.101			
NP-015142	0.128 ±0.007	33327 ±1984	0.933			
NP-015145	0.129 ±0.008	37114 ±4329	1.039			
NP-015146	0.122 ±0.003	36099 ±3197	1.011			
NP-015147	0.114 ±0.006	43113 ±1918	1.207			
NP-015148	0.117 ±0.001	44081 ±1160	1.234			
NP-015151	0.126 ±0.005	48649 ±3686	1.362			
NP-015152	0.118 ±0.002	71523 ±4407	1.915			
NP-015153	0.115 ±0.004	44422 ±2882	1.244			
NP-015154	0.122 ±0.002	62949 ±3669	1.685			
NP-015155	0.117 ±0.001	50010 ±4325	1.400			
NP-015156	0.124 ±0.002	44744 ±4351	1.253			
NP-015157	0.121 ±0.001	44606 ±3215	1.249			
NP-015164	0.122 ±0.002	41544 ±1180	1.163			
NP-015165	0.124 ±0.007	28005 ±1526	0.784			
NP-015166	0.119 ±0.004	40166 ±2419	1.125			
NP-015167	0.118 ±0.003	39254 ±1344	1.099			
NP-015183	0.117 ±0.003	51099 ±1739	1.431			
NP-015184	0.118 ±0.003	33134 ±3293	0.928			
NP-015185	0.116 ±0.004	45478 ±1907	1.273			
NP-015186	0.130 ±0.008	41633 ±3108	1.166			
NP-015188	0.121 ±0.004	34840 ±895	0.976			
NP-015189	0.118 ±0.004	36613 ±2755	1.025			
NP-015190	0.118 ±0.008	71437 ±7509	1.913			
NP-015191	0.125 ±0.002	38931 ±7253	1.090			

NP-015192	0.122 ± 0.009	40262 ± 6663	1.127			
NP-015205	0.127 ± 0.007	45095 ± 835	1.263			
NP-015208	0.115 ± 0.002	46842 ± 2036	1.312			
NP-015210	0.118 ± 0.003	65605 ± 2154	1.757			
NP-015211	0.116 ± 0.004	30307 ± 1695	0.849			
NP-015212	0.116 ± 0.002	30002 ± 1568	0.840			
NP-015214	0.121 ± 0.003	37614 ± 1141	1.053			
NP-015215	0.115 ± 0.004	37710 ± 2244	1.056			
NP-015216	0.111 ± 0.028	36262 ± 1982	1.015			
NP-015217	0.125 ± 0.009	36926 ± 3721	1.034			
NP-015218	0.120 ± 0.003	36193 ± 1513	1.013			
NP-015222	0.116 ± 0.003	42171 ± 1106	1.181			
NP-015223	0.118 ± 0.001	40225 ± 2221	1.126			
NP-015224	0.119 ± 0.001	34162 ± 1775	0.957			
NP-015225	0.121 ± 0.001	47369 ± 3850	1.326			
NP-015226	0.122 ± 0.002	52434 ± 2656	1.468			
NP-015227	0.119 ± 0.007	44583 ± 2729	1.248			
NP-015228	0.115 ± 0.004	31723 ± 795	0.888			
NP-015230	0.123 ± 0.005	31704 ± 2240	0.888			
NP-015231	0.121 ± 0.004	26900 ± 838	0.753			
NP-015233	0.120 ± 0.005	38135 ± 1914	1.068			
NP-015241	0.118 ± 0.001	46008 ± 4403	1.288			
NP-015242	0.117 ± 0.002	37426 ± 2345	1.048			
NP-015244	0.118 ± 0.005	34587 ± 4717	0.968			
NP-015247	0.120 ± 0.005	37854 ± 1331	1.060			
NP-015248	0.121 ± 0.003	31188 ± 676	0.873			
NP-015249	0.117 ± 0.001	42891 ± 6129	1.201			
NP-015250	0.118 ± 0.006	60192 ± 4304	1.612			
NP-015252	0.115 ± 0.003	36122 ± 3949	1.011			
NP-015253	0.122 ± 0.003	29142 ± 2080	0.816			
NP-015254	0.128 ± 0.005	39476 ± 5082	1.105			
NP-015257	0.118 ± 0.002	23873 ± 920	0.668			

NP-015258	0.122 ± 0.007	42015 ± 2348	1.176			
NP-015259	0.059 ± 0.003 **					
NP-015260	0.121 ± 0.004	51293 ± 2931	1.436			
NP-015262	0.116 ± 0.005	21914 ± 974	0.587			
NP-015263	0.117 ± 0.002	33791 ± 1344	0.946			
NP-015265	0.117 ± 0.003	38678 ± 1212	1.083			
NP-015266	0.125 ± 0.009	35979 ± 4243	1.007			
NP-015267	0.119 ± 0.006	53110 ± 9227	1.487			
NP-015268	0.116 ± 0.006	32174 ± 768	0.901			
NP-015270	0.113 ± 0.004	36476 ± 1937	1.021			
NP-015274	0.125 ± 0.005	33491 ± 2300	0.938			
NP-015275	0.131 ± 0.008	35714 ± 870	1.000			
NP-015276	0.117 ± 0.002	41071 ± 3421	1.150			
NP-015277	0.124 ± 0.005	39624 ± 2970	1.109			
NP-015278	0.117 ± 0.004	47897 ± 838	1.341			
NP-015279	0.117 ± 0.001	34406 ± 2553	0.963			
NP-015280	0.120 ± 0.002	57825 ± 2554	1.548			
NP-015285	0.128 ± 0.002	50169 ± 5018	1.405			
NP-015286	0.121 ± 0.003	54968 ± 1531	1.472			
NP-015287	0.128 ± 0.008	42961 ± 4961	1.203			
NP-015293	0.117 ± 0.002	28830 ± 4213	0.807			
NP-015300	0.111 ± 0.002	50465 ± 868	1.413			
NP-015301	0.124 ± 0.005	38701 ± 4683	1.084			
NP-015302	0.111 ± 0.004	43872 ± 1818	1.228			
NP-015312	0.126 ± 0.003	43406 ± 2895	1.215			
NP-015313	0.118 ± 0.001	49058 ± 3479	1.374			
NP-015314	0.124 ± 0.003	37205 ± 2225	1.042			
NP-015315	0.137 ± 0.011	26426 ± 3425	0.740			
NP-015323	0.117 ± 0.015	46722 ± 2645	1.308			
NP-015325	0.119 ± 0.003	50250 ± 2809	1.407			
NP-015326	0.119 ± 0.006	52709 ± 3302	1.476			
NP-015327	0.125 ± 0.007	36030 ± 3448	1.009			

NP-015328	0.119 ±0.006	36262 ±2533	1.015			
NP-015329	0.138 ±0.005	35593 ±1991	0.997			
NP-015330	0.106 ±0.011	33292 ±1943	0.932			
NP-015331	0.122 ±0.001	45387 ±6567	1.271			
NP-015333	0.119 ±0.006	50527 ±1113	1.415			
NP-015336	0.122 ±0.003	49111 ±3173	1.375			
NP-015337	0.112 ±0.002	34828 ±2282	0.975			
NP-015338	0.112 ±0.011	48419 ±4918	1.356			
NP-015341	0.120 ±0.003	46045 ±3436	1.289			
NP-015347	0.130 ±0.008	25439 ±2091	0.712			
NP-015348	0.122 ±0.006	38957 ±1785	1.091			
NP-015349	0.118 ±0.002	38203 ±1965	1.070			
NP-015350	0.124 ±0.003	46246 ±2114	1.295			
NP-015351	0.116 ±0.005	46695 ±4663	1.307			
NP-015352	0.118 ±0.001	32370 ±1695	0.906			
NP-015353	0.122 ±0.004	55676 ±3652	1.559			
NP-015354	0.114 ±0.006	38773 ±1742	1.086			
NP-015356	0.131 ±0.007	24835 ±2297	0.695			
NP-015365	0.115 ±0.003	43990 ±2508	1.232			
NP-015368	0.116 ±0.001	47682 ±996	1.335			
NP-015369	0.122 ±0.004	49533 ±3685	1.387			
NP-015468	0.120 ±0.004	39047 ±4271	1.093			
NP-015469	0.124 ±0.006	37757 ±2321	1.057			
NP-015470	0.129 ±0.006	44090 ±5759	1.234			
NP-015471	0.138 ±0.003 *					
NP-015473	0.122 ±0.005	34966 ±5351	0.979			
NP-015474	0.122 ±0.004	58965 ±2281	1.579			
NP-015475	0.118 ±0.005	47535 ±2486	1.331			
NP-015476	0.118 ±0.003	52963 ±3361	1.483			
NP-015477	0.122 ±0.004	43232 ±2284	1.210			
NP-015478	0.116 ±0.009	45997 ±5573	1.288			
NP-015479	0.121 ±0.003	33618 ±2060	0.941			

NP-015480	0.119 ± 0.003	39236 ± 2605	1.099			
NP-015481	0.119 ± 0.002	38165 ± 1273	1.069			
NP-015482	0.121 ± 0.002	36502 ± 1800	1.022			
NP-015483	0.117 ± 0.020	36187 ± 2615	1.013			
NP-015484	0.134 ± 0.003	46035 ± 4032	1.289			
NP-015485	0.122 ± 0.005	39722 ± 1408	1.112			
NP-015486	0.126 ± 0.005	27230 ± 253	0.762			
NP-015488	0.114 ± 0.002	28893 ± 303	0.809			
NP-015489	0.114 ± 0.019	66943 ± 7451	1.792			
NP-015491	0.116 ± 0.005	45986 ± 2272	1.288			
NP-015492	0.117 ± 0.002	36606 ± 771	1.025			
NP-015493	0.113 ± 0.003	42080 ± 473	1.178			
NP-015494	0.123 ± 0.006	37142 ± 2105	1.040			
NP-015495	0.114 ± 0.004	29570 ± 190	0.828			
NP-015496	0.112 ± 0.002	69350 ± 6683	1.942			
NP-015497	0.111 ± 0.002	49396 ± 2618	1.383			
NP-015498	0.122 ± 0.001	33912 ± 1033	0.950			
NP-015499	0.114 ± 0.010	40965 ± 707	1.147			
NP-015500	0.120 ± 0.010	25896 ± 899	0.725			
NP-015502	0.121 ± 0.003	39962 ± 2303	1.119			
NP-015504	0.123 ± 0.001	38452 ± 621	1.077			
NP-015505	0.123 ± 0.001	44817 ± 1636	1.255			
NP-015506	0.125 ± 0.003	57450 ± 4281	1.609			
NP-015507	0.122 ± 0.006	57471 ± 4815	1.539			
NP-015508	0.123 ± 0.003	45229 ± 1137	1.266			
NP-015509	0.128 ± 0.004	35946 ± 3377	1.006			
NP-015510	0.118 ± 0.006	31914 ± 4966	0.894			
NP-015511	0.120 ± 0.004	31602 ± 2087	0.885			
NP-015513	0.115 ± 0.005	40446 ± 4323	1.132			
NP-015528	0.120 ± 0.006	41231 ± 1494	1.154			
NP-015529	0.129 ± 0.005	41099 ± 3594	1.151			
NP-015530	0.121 ± 0.005	47893 ± 563	1.341			

NP-015531	0.137 ± 0.016	30830 ± 3468	0.863			
NP-015532	0.123 ± 0.011	35686 ± 426	0.999			
NP-015534	0.134 ± 0.022	24971 ± 2758	0.699			
NP-015536	0.131 ± 0.011	40863 ± 3140	1.144			
NP-015537	0.130 ± 0.005	50376 ± 6022	1.410			
NP-015538	0.123 ± 0.007	37640 ± 4438	1.054			
NP-015539	0.123 ± 0.002	55809 ± 7308	1.563			
NP-015540	0.117 ± 0.002	51489 ± 3210	1.442			
NP-015541	0.127 ± 0.004	53152 ± 4731	1.488			
NP-015544	0.124 ± 0.004	35578 ± 776	0.996			
NP-015546	0.120 ± 0.009	50972 ± 6292	1.427			
NP-015547	0.127 ± 0.005	26613 ± 3470	0.745			
NP-015548	0.121 ± 0.007	52604 ± 9305	1.473			
NP-015555	0.124 ± 0.005	55440 ± 2710	1.484			
NP-015558	0.123 ± 0.003	43348 ± 3372	1.214			
NP-015560	0.120 ± 0.002	51147 ± 4236	1.432			
NP-015561	0.128 ± 0.006	51193 ± 6965	1.433			
NP-015563	0.126 ± 0.007	36306 ± 1064	1.017			
NP-015564	0.132 ± 0.008	42887 ± 4484	1.201			
NP-015565	0.125 ± 0.003	42250 ± 2783	1.183			
NP-015566	0.115 ± 0.002	40900 ± 1887	1.145			
NP-015567	0.127 ± 0.005	67275 ± 2639	1.801			
NP-015568	0.122 ± 0.001	58209 ± 9731	1.630			
NP-015569	0.125 ± 0.004	44367 ± 3465	1.242			
NP-015570	0.127 ± 0.002	22099 ± 1351	0.619			
NP-015571	0.116 ± 0.004	50011 ± 2070	1.400			
NP-015572	0.119 ± 0.002	56490 ± 3694	1.582			
NP-015573	0.111 ± 0.002	47802 ± 6322	1.338			
NP-015574	0.123 ± 0.001	32545 ± 1961	0.911			
NP-015575	0.137 ± 0.012	40949 ± 6239	1.147			
NP-015576	0.127 ± 0.002	43965 ± 4432	1.231			
NP-015577	0.126 ± 0.003	46886 ± 4019	1.313			

NP-015578	0.121 ±0.002	55255 ±7120	1.547			
NP-015579	0.129 ±0.007	38445 ±5541	1.076			
NP-015580	0.120 ±0.001	48647 ±2030	1.362			
NP-015583	0.130 ±0.006	41702 ±1615	1.168			
NP-015585	0.127 ±0.003	63597 ±6364	1.703			
NP-015586	0.125 ±0.007	39787 ±3760	1.114			
NP-015587	0.125 ±0.003	40421 ±1424	1.132			
NP-015593	0.116 ±0.003	69578 ±1990	1.863			
NP-015596	0.118 ±0.004	33924 ±2617	0.950			
NP-015598	0.120 ±0.003	42400 ±1082	1.187			
NP-015608	0.119 ±0.003	55639 ±5523	1.558			
NP-015609	0.115 ±0.004	19819 ±1664	0.531			
NP-015623	0.124 ±0.003	40102 ±5127	1.123			
NP-015624	0.106 ±0.005	55662 ±815	1.490			
NP-015625	0.122 ±0.002	48886 ±3612	1.369			
NP-015627	0.128 ±0.006	60127 ±4526	1.610			
NP-015628	0.117 ±0.005	53002 ±5097	1.484			
NP-015629	0.112 ±0.003	46085 ±3535	1.290			
NP-015636	0.120 ±0.003	37718 ±3077	1.056			
NP-015637	0.136 ±0.013	45277 ±5295	1.268			
NP-015638	0.116 ±0.004	38652 ±1400	1.082			
NP-015639	0.119 ±0.004	35890 ±1543	1.005			
NP-015643	0.113 ±0.005	30399 ±593	0.851			
NP-015650	0.124 ±0.006	28524 ±3162	0.799			
NP-015651	0.126 ±0.006	31078 ±2144	0.870			
NP-015652	0.122 ±0.004	68870 ±1127	1.844			
NP-015655	0.128 ±0.006	61212 ±6774	1.639			
NP-015656	0.121 ±0.002	38721 ±430	1.084			
NP-015657	0.122 ±0.008	49563 ±2000	1.388			
NP-015658	0.128 ±0.006	35129 ±2275	0.984			
NP-015661	0.126 ±0.005	29888 ±2346	0.837			
NP-015662	0.126 ±0.003	58595 ±5411	1.569			

NP-015663	0.122 ±0.004	48110 ±2239	1.347			
NP-015664	0.129 ±0.005	54119 ±5515	1.515			
NP-015665	0.124 ±0.005	50337 ±5043	1.409			
NP-015666	0.126 ±0.004	63862 ±8500	1.710			
NP-015679	0.127 ±0.009	72146 ±9177	1.932			
NP-015680	0.121 ±0.002	69016 ±3399	1.848			
NP-015681	0.122 ±0.003	47150 ±3960	1.320			
NP-015682	0.121 ±0.006	52122 ±6960	1.459			
NP-015683	0.137 ±0.013	49575 ±4038	1.388			
NP-015684	0.121 ±0.004	40994 ±2537	1.148			
NP-015685	0.117 ±0.008	34803 ±2485	0.974			
NP-015688	0.114 ±0.002	37538 ±5013	1.051			
NP-015689	0.116 ±0.001	37362 ±3445	1.046			
NP-015690	0.115 ±0.006	43282 ±1575	1.212			
NP-015691	0.120 ±0.004	61740 ±4487	1.653			
NP-015706	0.123 ±0.002	30835 ±1968	0.863			
NP-015707	0.118 ±0.006	50738 ±2571	1.421			
NP-015724	0.117 ±0.003	43548 ±9650	1.219			
NP-015733	0.133 ±0.001	44588 ±3306	1.248			
NP-015739	0.114 ±0.005	41349 ±1462	1.158			
NP-015745	0.115 ±0.004	48659 ±3323	1.362			
NP-015751	0.114 ±0.019	39215 ±3748	1.098			
NP-015771	0.123 ±0.007	57922 ±688	1.551			
NP-015772	0.123 ±0.007	48704 ±1454	1.364			
NP-015790	0.110 ±0.004	45347 ±2934	1.270			
NP-015793	0.125 ±0.004	65687 ±7539	1.759			
NP-015794	0.128 ±0.004	40988 ±1885	1.148			
NP-015795	0.126 ±0.002	40240 ±1626	1.127			
NP-015796	0.110 ±0.002	36803 ±1864	1.030			
NP-015797	0.124 ±0.006	50294 ±4009	1.408			
NP-015801	0.118 ±0.003	33531 ±2372	0.939			
NP-015803	0.127 ±0.005	54435 ±3692	1.524			

NP-015804	0.135 ±0.005	47924 ±3980	1.342			
NP-015806	0.135 ±0.006	49477 ±2995	1.385			
NP-015807	0.115 ±0.013	47605 ±3864	1.333			
NP-015808	0.111 ±0.002	38931 ±1398	1.090			
NP-015809	0.124 ±0.008	37506 ±6117	1.050			
NP-015812	0.131 ±0.008	41606 ±2402	1.165			
NP-015813	0.113 ±0.002	37553 ±434	1.051			
NP-015814	0.119 ±0.008	43020 ±797	1.205			
NP-015815	0.128 ±0.004	48424 ±7543	1.356			
NP-015816	0.129 ±0.015	24856 ±593	0.696			
NP-015817	0.107 ±0.003	38476 ±3904	1.077			
NP-015819	0.123 ±0.004	24377 ±6777	0.683			
NP-015820	0.124 ±0.006	40157 ±4031	1.124			
NP-015821	0.126 ±0.002	50487 ±1706	1.414			
NP-015822	0.120 ±0.003	50808 ±8741	1.423			
NP-015823	0.126 ±0.002	55912 ±1434	1.497			
NP-015825	0.126 ±0.012	44637 ±2258	1.250			
NP-015826	0.121 ±0.001	43668 ±3509	1.223			
NP-015834	0.122 ±0.004	38699 ±2628	1.084			
NP-015835	0.112 ±0.007	55495 ±2333	1.486			
NP-015836	0.108 ±0.004	38463 ±2449	1.077			
NP-015837	0.113 ±0.009	30921 ±1866	0.866			
NP-015838	0.129 ±0.007	60222 ±13929	1.686			
NP-015841	0.114 ±0.002	41687 ±3339	1.167			
NP-015842	0.119 ±0.002	46364 ±3595	1.298			
NP-015859	0.118 ±0.004	56799 ±982	1.521			
NP-015860	0.127 ±0.009	51388 ±6604	1.439			
NP-015862	0.137 ±0.010	50407 ±7264	1.411			
NP-015863	0.124 ±0.003	56515 ±2298	1.513			
NP-015864	0.119 ±0.001	41424 ±206	1.160			
NP-015865	0.118 ±0.001	59244 ±2393	1.586			
NP-015866	0.115 ±0.007	63296 ±6632	1.772			

NP-015869	0.132 ± 0.008	44001 ± 5097	1.232			
NP-015875	0.120 ± 0.002	68686 ± 10623	1.839			
NP-015876	0.127 ± 0.007	50592 ± 6703	1.417			
NP-015880	0.125 ± 0.002	49449 ± 898	1.385			
NP-015881	0.123 ± 0.029	36637 ± 2473	1.026			
NP-015882	0.117 ± 0.003	41730 ± 3671	1.168			
NP-015883	0.116 ± 0.003	54100 ± 2681	1.449			
NP-015884	0.122 ± 0.005	52246 ± 2644	1.463			
NP-015885	0.122 ± 0.004	58103 ± 668	1.556			
NP-015886	0.122 ± 0.005	40032 ± 4930	1.121			
NP-015887	0.129 ± 0.007	56508 ± 1622	1.513			
NP-015888	0.126 ± 0.004	54481 ± 5669	1.525			
NP-015902	0.128 ± 0.006	54721 ± 4058	1.532			
NP-015903	0.127 ± 0.008	49139 ± 4170	1.376			
NP-015904	0.127 ± 0.006	47752 ± 7214	1.337			
NP-015906	0.113 ± 0.007	41156 ± 1824	1.152			
NP-015923	0.121 ± 0.004	42831 ± 2241	1.199			
NP-015933	0.125 ± 0.003	51675 ± 8309	1.447			
NP-015934	0.124 ± 0.003	63113 ± 2511	1.690			
NP-015935	0.132 ± 0.005	60986 ± 5485	1.633			
NP-015936	0.119 ± 0.006	53637 ± 3062	1.502			
NP-015937	0.126 ± 0.008	44323 ± 2821	1.241			
NP-015938	0.126 ± 0.003	49089 ± 2713	1.374			
NP-015939	0.129 ± 0.002	35335 ± 2417	0.989			
NP-015942	0.115 ± 0.006	40327 ± 2479	1.129			
NP-015948	0.124 ± 0.002	53080 ± 9206	1.486			
NP-015950	0.138 ± 0.007	59221 ± 9054	1.658			
NP-015957	0.122 ± 0.006	50597 ± 6058	1.417			
NP-015958	0.110 ± 0.005	39536 ± 3324	1.107			
NP-015959	0.117 ± 0.006	44971 ± 819	1.259			
NP-015963	0.127 ± 0.010	48863 ± 6090	1.368			
NP-015964	0.117 ± 0.007	65989 ± 3067	1.767			

NP-015965	0.121 ± 0.002	37549 ± 634	1.051			
NP-015966	0.110 ± 0.004	32687 ± 2517	0.915			
NP-015967	0.117 ± 0.007	45638 ± 4061	1.278			
NP-015968	0.128 ± 0.006	51342 ± 4183	1.438			
NP-015972	0.118 ± 0.001	41912 ± 966	1.174			
NP-015973	0.129 ± 0.008	58346 ± 759	1.562			
NP-015974	0.123 ± 0.004	47164 ± 1181	1.321			
NP-015975	0.118 ± 0.004	46572 ± 2250	1.304			
NP-016009	0.121 ± 0.002	66034 ± 8499	1.768			
NP-016014	0.127 ± 0.009	49426 ± 6230	1.384			
NP-016016	0.115 ± 0.001	27949 ± 3294	0.783			
NP-016017	0.108 ± 0.003	30187 ± 1661	0.845			
NP-016018	0.118 ± 0.003	46721 ± 2955	1.308			
NP-016019	0.109 ± 0.008	53493 ± 7345	1.498			
NP-016021	0.131 ± 0.008	44492 ± 5011	1.246			
NP-016031	0.120 ± 0.002	52227 ± 4277	1.462			
NP-016036	0.122 ± 0.007	38935 ± 7585	1.090			
NP-016037	0.117 ± 0.004	46272 ± 8101	1.296			
NP-016038	0.121 ± 0.009	69625 ± 2866	1.864			
NP-016055	0.126 ± 0.005	60175 ± 4305	1.685			
NP-016056	0.117 ± 0.003	43651 ± 2571	1.222			
NP-016078	0.115 ± 0.006	49719 ± 3804	1.392			
NP-016079	0.135 ± 0.009	39901 ± 1101	1.117			
NP-016086	0.120 ± 0.003	45429 ± 1928	1.272			
NP-016090	0.115 ± 0.012	31363 ± 1494	0.878			
NP-016091	0.117 ± 0.005	61851 ± 7502	1.656			
NP-016095	0.121 ± 0.005	36960 ± 6130	1.035			
NP-016096	0.126 ± 0.008	45251 ± 1220	1.267			
NP-016098	0.117 ± 0.004	38072 ± 5027	1.066			
NP-016099	0.121 ± 0.005	28788 ± 4844	0.806			
NP-016100	0.118 ± 0.004	63030 ± 5659	1.688			
NP-016101	0.126 ± 0.009	39897 ± 3682	1.117			

NP-016104	0.129 ± 0.004	37873 ± 4409	1.060				
NP-016116	0.128 ± 0.004	42021 ± 4253	1.177				
NP-016123	0.120 ± 0.004	45935 ± 7355	1.286				
NP-016124	0.122 ± 0.003	32632 ± 4994	0.914				
NP-016131	0.120 ± 0.004	45337 ± 3280	1.269				
NP-016132	0.121 ± 0.005	40355 ± 809	1.130				
NP-016136	0.120 ± 0.005	50866 ± 4983	1.424				
NP-016137	0.123 ± 0.007	46817 ± 6873	1.311				
NP-016180	0.121 ± 0.003	63524 ± 12666	1.779				
NP-016181	0.121 ± 0.003	39967 ± 4381	1.119				
NP-016183	0.129 ± 0.005	54164 ± 6378	1.517				
NP-016184	0.120 ± 0.002	62619 ± 4254	1.677				
NP-016185	0.115 ± 0.005	40975 ± 3078	1.147				
NP-016186	0.138 ± 0.015	38004 ± 7271	1.064				
NP-016187	0.127 ± 0.006	59847 ± 8559	1.676				
NP-016189	0.123 ± 0.003	39992 ± 3120	1.120				
NP-016190	0.120 ± 0.003	43440 ± 3705	1.216				
NP-016191	0.131 ± 0.006	52234 ± 4960	1.463				
NP-016192	0.126 ± 0.006	41847 ± 3582	1.172				
NP-016193	0.117 ± 0.005	34022 ± 3278	0.953				
NP-016194	0.106 ± 0.003	47011 ± 4310	1.316				
NP-016195	0.121 ± 0.005	52869 ± 2313	1.416				
NP-016196	0.122 ± 0.001	52109 ± 3370	1.459				
NP-016197	0.118 ± 0.005	37379 ± 7600	1.047				
NP-016201	0.123 ± 0.004	52335 ± 1989	1.465				
NP-016202	0.121 ± 0.003	39069 ± 1927	1.094				
NP-016204	0.131 ± 0.009	24325 ± 3840	0.681				
NP-016205	0.129 ± 0.003	35388 ± 2057	0.991				
NP-016206	0.124 ± 0.006	56369 ± 2086	1.509				
NP-016207	0.127 ± 0.004	45793 ± 3017	1.282				
NP-016383	0.121 ± 0.003	42532 ± 2668	1.191				
NP-016386	0.112 ± 0.007	20164 ± 1438	0.540				

NP-016560	0.115 ± 0.005	48934 ± 1964	1.370			
NP-016639	0.115 ± 0.009	37105 ± 2739	1.039			
NP-016667	0.115 ± 0.006	43296 ± 479	1.212			
NP-016668	0.120 ± 0.002	44589 ± 1698	1.248			
NPC 15199	0.101 ± 0.003	29016 ± 1110	0.812			
NPC-189	0.121 ± 0.005	27180 ± 1734	0.761			
N-p-coumaroyl-N'-caffeoylputrescine	0.118 ± 0.001	34635 ± 1538	0.970			
N-p-Coumaroyloctopamine	0.126 ± 0.013	36726 ± 1779	1.028			
N-Phenyl-7-(hydroxyimino)-cyclopropa[B]chromen-1A-carboxamide	0.102 ± 0.007	58579 ± 9392	1.640			
N-Phenylanthranilic acid	0.092 ± 0.006	70153 ± 6019	1.939			
NPPB	0.108 ± 0.004	36842 ± 1321	1.032			
N-p-Tosyl-L-phenylalanine chloromethyl ketone	0.081 ± 0.006 **					
N-p-trans-Coumaroyltyramine	0.126 ± 0.009	40664 ± 2378	1.139			
NPY 5RA972	0.114 ± 0.006	35705 ± 1088	1.000			
NQ-12	0.115 ± 0.002	56345 ± 8322	1.578			
NS 2028	0.097 ± 0.008	45090 ± 3614	1.262			
NS 521 oxalate	0.112 ± 0.006	47515 ± 6397	1.330			
NS-1619	0.087 ± 0.002 *					
NS-398	0.110 ± 0.002	29213 ± 2548	0.818			
NS8593 hydrochloride	0.107 ± 0.002	5165 ± 185	0.143	0.107 ± 0.003	16926 ± 2408	0.518
NSC 23766	0.071 ± 0.005 **					
NSC 625987	0.112 ± 0.003	48877 ± 8243	1.369			
NSC 632839 hydrochloride	0.082 ± 0.004 **					
NSC 663284	0.106 ± 0.006	25955 ± 2520	0.727			
NSC 693868	0.105 ± 0.011	34782 ± 1816	0.974			
NSC 721648	0.101 ± 0.007	42799 ± 1935	1.198			
NSC 95397	0.059 ± 0.002 **					
NSC-3852	0.076 ± 0.008 **					
NSC-649900	0.120 ± 0.008	34881 ± 2809	0.977			
NSC-682011	0.117 ± 0.005	39743 ± 5396	1.113			
NSC-95397	0.074 ± 0.008 **					
N-Succinyl-L-proline	0.101 ± 0.018	24585 ± 6056	0.688			

NTNCB hydrochloride	0.116 ± 0.002	42252 ± 3723	1.183			
N-trans-Feruloyl-3-methoxytyramine	0.132 ± 0.007	26972 ± 3946	0.755			
N-trans-Feruloyltyramine	0.122 ± 0.005	38486 ± 3663	1.078			
nTZDpa	0.105 ± 0.007	34769 ± 1445	0.974			
NU 1025	0.114 ± 0.003	48354 ± 3517	1.354			
NU 7026	0.109 ± 0.002	27373 ± 1975	0.766			
NU6027	0.107 ± 0.009	32859 ± 3526	0.920			
Nuezhenide	0.123 ± 0.006	40803 ± 4263	1.142			
Nullscript	0.101 ± 0.011	19958 ± 526	0.619			
NVP DPP 728 dihydrochloride	0.121 ± 0.008	30767 ± 2104	0.861			
NXY 059	0.113 ± 0.004	30809 ± 3529	0.863			
Nyasicol	0.126 ± 0.006	38415 ± 4050	1.076			
Nyasicol 1,2-acetonide	0.119 ± 0.005	35509 ± 3197	0.994			
Nyasicoside	0.132 ± 0.005	36580 ± 3553	1.024			
Nylidrin	0.108 ± 0.003	38584 ± 4373	1.080			
Nylidrin hydrochloride	0.126 ± 0.012	29468 ± 2867	0.825			
Nyssoside	0.126 ± 0.010	40243 ± 1591	1.127			
Nystatin	0.109 ± 0.002	30135 ± 2036	0.844			
Nystatine	0.113 ± 0.007	39784 ± 1437	1.114			
O-(Carboxymethyl)-hydroxylamine hemihydrochloride	0.092 ± 0.001 *					
O-1918	0.112 ± 0.003	28366 ± 2262	0.794			
O-3M3FBS	0.102 ± 0.011	37814 ± 1919	1.059			
O6-benzylguanine	0.096 ± 0.004	50061 ± 3269	1.402			
O-Acetylcyclocalopin A	0.120 ± 0.004	42612 ± 2392	1.193			
OBA	0.115 ± 0.006	35858 ± 595	1.004			
OBA Ester	0.128 ± 0.003	33895 ± 770	0.949			
OBAA	0.117 ± 0.005	24776 ± 619	0.694			
Obacunone	0.122 ± 0.003	57779 ± 4881	1.618			
O-benzyl-l-serine	0.104 ± 0.002	42576 ± 3950	1.192			
Obestain	0.115 ± 0.005	18835 ± 1441	0.527			
Obliquin	0.105 ± 0.003	32370 ± 3737	0.906			
Oblongine	0.117 ± 0.004	38229 ± 412	1.070			

Obtusaquinone	0.109 ± 0.008	32651 ± 3079	0.914			
Ochratoxin A	0.093 ± 0.003 *					
Ocotillone	0.115 ± 0.030	31817 ± 2502	0.891			
Octacosyl (E)-ferulate	0.114 ± 0.001	33863 ± 2451	0.948			
Octadecanoic acid	0.110 ± 0.006	34002 ± 3259	0.952			
Octinoxate	0.110 ± 0.002	34347 ± 1538	0.962			
Octisalate	0.101 ± 0.003	37564 ± 4785	1.052			
Octocrylene	0.113 ± 0.005	36798 ± 2337	1.030			
Octodrine	0.095 ± 0.027	37697 ± 5197	1.055			
Octopamine hydrochloride	0.111 ± 0.007	33023 ± 2735	0.925			
Octreotide	0.110 ± 0.011	46069 ± 3424	1.290			
O-Demethylforbexanthone	0.116 ± 0.003	34858 ± 3235	0.976			
Odonicin	0.109 ± 0.011	45573 ± 4499	1.276			
Odoratone	0.120 ± 0.002	33978 ± 1214	0.951			
Odorine	0.114 ± 0.010	37078 ± 1539	1.038			
Odorinol	0.118 ± 0.013	27130 ± 781	0.760			
Odoroside H	0.115 ± 0.008	42731 ± 1326	1.196			
ODQ	0.111 ± 0.005	45101 ± 2893	1.263			
Ofloxacin	0.077 ± 0.008 **					
Ohchinin	0.133 ± 0.003	52004 ± 561	1.456			
Ohchinin acetate	0.117 ± 0.010	35741 ± 4105	1.001			
Okadaic acid	0.117 ± 0.003	26606 ± 1546	0.745			
Olanzapine	0.129 ± 0.004	21557 ± 1124	0.604			
OLDA	0.105 ± 0.011	29515 ± 2416	0.826			
Oleamide	0.125 ± 0.001	29802 ± 2460	0.834			
Olean-12-ene-3,11-diol	0.119 ± 0.001	42093 ± 2171	1.179			
Olean-12-ene-3,11-dione	0.122 ± 0.005	48932 ± 2950	1.370			
Olean-12-ene-3,24-diol	0.116 ± 0.002	40300 ± 2789	1.128			
Oleandomycin phosphate	0.103 ± 0.012	19109 ± 2315	0.535			
Oleandrin	0.134 ± 0.010	36445 ± 3528	1.020			
Oleanoic acid	0.104 ± 0.006	34346 ± 3015	0.962			
Oleic Acid	0.100 ± 0.010	33051 ± 1241	0.925			

Oleonuezhenide	0.119 ± 0.001	40897 ± 1840	1.145			
Oleoside	0.124 ± 0.003	43447 ± 1059	1.216			
Oleoyl alanine	0.127 ± 0.001	30704 ± 293	0.860			
Oleoyl dopamine	0.105 ± 0.002	31777 ± 855	0.890			
Oleoyl ethanolamide	0.130 ± 0.010	36040 ± 1258	1.009			
Oleoyl GABA	0.128 ± 0.002	32358 ± 595	0.906			
Oleoyl glycine	0.127 ± 0.008	26154 ± 1395	0.732			
Oleuropeic acid	0.122 ± 0.009	44167 ± 6092	1.237			
Oleuropein	0.113 ± 0.005	38222 ± 1884	1.070			
Oleylethanolamide	0.114 ± 0.005	31564 ± 3239	0.884			
Oligomycin A	0.117 ± 0.010	17195 ± 1026	0.511			
Oligomycin C	0.132 ± 0.004 *					
Olivil	0.127 ± 0.002	32847 ± 1685	0.920			
Olivil monoacetate	0.124 ± 0.003	33074 ± 283	0.926			
Olmesartan	0.113 ± 0.005	47152 ± 2290	1.320			
Olmesartan medoxomil	0.124 ± 0.004	47293 ± 3845	1.324			
Olomoucine	0.118 ± 0.007	39237 ± 3171	1.099			
Olomoucine II	0.137 ± 0.009	57227 ± 5959	1.695			
Olopatadine	0.132 ± 0.002 *					
Olopatadine hydrochloride	0.127 ± 0.004	18627 ± 1714	0.519			
Olprinone hydrochloride	0.112 ± 0.007	32334 ± 2236	0.905			
Olsalazine sodium	0.104 ± 0.004	34929 ± 7331	0.978			
Oltipraz	0.106 ± 0.002	36391 ± 2088	1.019			
Olvanil	0.106 ± 0.011	40271 ± 657	1.128			
Ombuin	0.127 ± 0.023	58517 ± 7587	1.636			
OMDM-2	0.108 ± 0.010	38247 ± 1411	1.071			
Omega-3-acid esters (epa shown)	0.101 ± 0.006	36625 ± 4236	1.025			
Omeprazole	0.111 ± 0.003	54292 ± 3271	1.512			
O-Methylserotonin hydrochloride	0.104 ± 0.005	34330 ± 2438	0.961			
Oncrasin 1	0.111 ± 0.006	34744 ± 4094	0.973			
Ondansetron	0.116 ± 0.004	41415 ± 5008	1.160			
Ondansetron Hydrochloride	0.112 ± 0.003	35234 ± 2229	0.987			

Onitin	0.127 ± 0.006	33983 ± 1175	0.952			
Onitin 2'-O-glucoside	0.126 ± 0.006	52135 ± 3921	1.460			
Onitisin	0.127 ± 0.002	33644 ± 178	0.942			
Onitisin 2'-O-glucoside	0.122 ± 0.006	51796 ± 4469	1.450			
Ononetin	0.098 ± 0.004	41110 ± 6617	1.151			
O-Phospho-L-serine	0.097 ± 0.003	25445 ± 2513	0.712			
Opiniazide	0.119 ± 0.005	33754 ± 3164	0.945			
Oplodiol	0.136 ± 0.008	33306 ± 2872	0.933			
Oplopanone	0.118 ± 0.003	32991 ± 2944	0.924			
OR-486	0.104 ± 0.002	21933 ± 756	0.607			
Orbifloxacin	0.096 ± 0.009	19912 ± 1140	0.558			
Orcinol	0.125 ± 0.008	40551 ± 2600	1.135			
Org 24598 lithium salt	0.101 ± 0.002	34406 ± 906	0.963			
Org 27569	0.109 ± 0.003	28213 ± 1047	0.790			
Orienticin C	0.087 ± 0.002 **					
Orlistat	0.112 ± 0.013	45608 ± 4683	1.277			
Ormetoprim	0.128 ± 0.011	28815 ± 3874	0.807			
Ornidazole	0.123 ± 0.007	43003 ± 5833	1.204			
Ornithine hydrochloride	0.114 ± 0.002	33919 ± 692	0.950			
Orobol	0.115 ± 0.002	45436 ± 1151	1.272			
Orotic acid	0.116 ± 0.004	49521 ± 1716	1.387			
Orphenadrine citrate	0.107 ± 0.016	38461 ± 4410	1.077			
Orphenadrine hydrochloride	0.105 ± 0.010	30795 ± 3179	0.862			
Orsellinic acid	0.107 ± 0.006	38458 ± 3891	1.077			
Orsellinic acid, ethyl ester	0.116 ± 0.002	21176 ± 1620	0.593			
Osaiin	0.113 ± 0.007	47725 ± 5519	1.336			
Osajin 4'-methyl ether	0.106 ± 0.009	28778 ± 1099	0.806			
Oseltamivir	0.112 ± 0.005	39960 ± 428	1.119			
Oseltamivir phosphate	0.103 ± 0.003	31023 ± 2327	0.869			
Osthol	0.123 ± 0.003	37427 ± 1308	1.048			
Osthole	0.102 ± 0.003	24601 ± 135	0.689			
Ouabain	0.099 ± 0.015	38087 ± 5813	1.066			

Ouabain (-)-	0.115 ±0.009	21978 ±1262	0.615			
Ouabain, Octahydrate	0.097 ±0.002	52429 ±3198	1.468			
OXA-22	0.099 ±0.013	34144 ±337	0.956			
Oxacillin sodium	0.114 ±0.025	23580 ±1266	0.660			
Oxalamine citrate salt	0.110 ±0.005	40596 ±4592	1.137			
Oxaliplatin	0.118 ±0.003	32642 ±1195	0.914			
Oxamflatin	0.102 ±0.004	43361 ±2597	1.214			
Oxandrolone	0.113 ±0.004	50207 ±2297	1.406			
Oxantel pamoate	0.098 ±0.010	27463 ±3058	0.769			
Oxaprozin	0.098 ±0.006	36555 ±5591	1.024			
Oxatomide	0.110 ±0.018	30492 ±2301	0.854			
Oxazepam	0.113 ±0.010	48788 ±2945	1.366			
Oxcarbazepine	0.111 ±0.002	47915 ±1753	1.342			
Oxedrine	0.112 ±0.006	36293 ±3093	1.016			
Oxeladin Citrate	0.125 ±0.003	33661 ±2811	0.942			
Oxelaidin citrate	0.111 ±0.005	38153 ±3103	1.068			
Oxethazaine	0.116 ±0.004	45960 ±2962	1.287			
Oxfendazole	0.117 ±0.002	27954 ±2134	0.783			
Oxibendazole	0.109 ±0.002	35298 ±766	0.988			
Oxiconazole nitrate	0.064 ±0.001 **					
Oxidopamine hydrochloride	0.106 ±0.005	30204 ±5013	0.846			
Oxiniacic acid	0.108 ±0.009	34657 ±348	0.970			
Oxiracetam	0.100 ±0.008	40299 ±5258	1.128			
Oxiranecarboxylic acid, 2-[6-(4-chlorophenoxy)-hexyl]-ethyl ester	0.122 ±0.013	26355 ±1189	0.738			
Oxitriptan	0.081 ±0.006 **					
Oxocafestol, 16-	0.124 ±0.005	32945 ±1325	0.922			
Oxoepistephamiersine	0.118 ±0.017	31989 ±2492	0.896			
Oxokahweol, 16-	0.105 ±0.003	50237 ±1634	1.492			
Oxolamine citrate	0.105 ±0.002	42256 ±5228	1.183			
Oxolinic acid	0.065 ±0.010 **					
Oxonitine	0.108 ±0.006	41983 ±1599	1.176			
Oxotremorine methiodide	0.101 ±0.005	37326 ±3592	1.045			

Oxotremorine sesquifumarate	0.098 ± 0.010	27383 ± 2840	0.767			
Oxprenolol hydrochloride	0.112 ± 0.012	33007 ± 1736	0.924			
Oxtriphylline	0.109 ± 0.001	32840 ± 2120	0.920			
Oxyacanthine sulfate	0.118 ± 0.002	24761 ± 1678	0.693			
Oxybenzone	0.106 ± 0.007	40321 ± 3033	1.129			
Oxybutynin Chloride	0.103 ± 0.009	50650 ± 1871	1.400			
Oxyclozanide	0.064 ± 0.007 **					
Oxymatrine	0.117 ± 0.006	37334 ± 2411	1.045			
Oxymetazoline hydrochloride	0.098 ± 0.009	21527 ± 2225	0.595			
Oxymetholone	0.119 ± 0.005	22338 ± 1209	0.625			
Oxyphemedol	0.115 ± 0.015	47662 ± 4839	1.335			
Oxyphenbutazone	0.120 ± 0.004	19815 ± 547	0.541			
Oxyphencyclimine hydrochloride	0.105 ± 0.003	39909 ± 3624	1.117			
Oxyphenisatine	0.102 ± 0.012	6930 ± 519	0.204	0.111 ± 0.008	16536 ± 516	0.506
Oxyphenonium bromide	0.106 ± 0.002	19332 ± 1971	0.538			
Oxyquinoline sulfate	0.072 ± 0.006 **					
Oxyresveratrol	0.115 ± 0.033	32240 ± 1692	0.903			
Oxytetracycline	0.095 ± 0.002 *					
Oxytetracycline dihydrate	0.109 ± 0.007	39807 ± 2301	1.115			
Oxytetracycline, a -apo-	0.101 ± 0.004	58538 ± 3495	1.739			
Oxythiamine chloride hydrochloride	0.106 ± 0.006	33363 ± 2463	0.934			
Oxytocin(free acid)	0.120 ± 0.002	43621 ± 8470	1.221			
Ozagrel	0.117 ± 0.008	50142 ± 4365	1.404			
Ozagrel hydrochloride	0.113 ± 0.003	57031 ± 2397	1.588			
P1,P4-Di(adenosine-5')-tetraphosphate triammonium	0.099 ± 0.004	19416 ± 1023	0.537			
p21-Activated Kinase Inhibitor III, IPA-3	0.121 ± 0.013	26788 ± 3647	0.750			
P32/98	0.114 ± 0.005	36402 ± 994	1.019			
p38 MAP Kinase Inhibitor	0.114 ± 0.010	27841 ± 2398	0.780			
p38 MAP Kinase Inhibitor III	0.060 ± 0.017 **					
p38 MAP Kinase Inhibitor IV	0.067 ± 0.001 **					
p38 MAP Kinase Inhibitor VI, JX401	0.113 ± 0.002	40323 ± 5041	1.129			
p38 MAP Kinase Inhibitor VII, SD-169	0.119 ± 0.011	41032 ± 1896	1.149			

p38 MAP Kinase Inhibitor VIII	0.133 ± 0.019	108412 ± 8972	3.211	0.121 ± 0.018	31173 ± 2478	0.954
p53 Activator III, RITA	0.094 ± 0.008	24260 ± 1223	0.679			
PAC 1	0.105 ± 0.001	38126 ± 700	1.068			
Pachyaximine A	0.110 ± 0.005	28590 ± 2445	0.801			
Pachypodol	0.109 ± 0.018	30142 ± 1341	0.844			
Pachyrrhizin	0.100 ± 0.005	37633 ± 1419	1.054			
Paclitaxel	0.115 ± 0.003	27707 ± 1477	0.776			
Pacocf3	0.112 ± 0.002	32431 ± 3112	0.908			
Padmatin	0.114 ± 0.011	44285 ± 4110	1.240			
Paeonol	0.113 ± 0.008	38166 ± 928	1.069			
PAF C16	0.110 ± 0.002	19507 ± 359	0.602			
PAF C18	0.103 ± 0.002	31500 ± 1283	0.882			
PAF C18:1	0.108 ± 0.002	26647 ± 1182	0.746			
Paliperidone	0.097 ± 0.007	34215 ± 2490	0.958			
Palmatine	0.118 ± 0.003	43983 ± 3422	1.231			
palmatine chloride	0.111 ± 0.003	28454 ± 2306	0.797			
Palmidrol	0.116 ± 0.005	31561 ± 1215	0.884			
Palmitamide	0.123 ± 0.012	34681 ± 1223	0.971			
Palmitoyl alanine	0.126 ± 0.003	39501 ± 2107	1.106			
Palmitoyl dopamine	0.104 ± 0.001	36513 ± 3346	1.022			
Palmitoyl ethanolamide	0.126 ± 0.010	40056 ± 1047	1.122			
Palmitoyl GABA	0.126 ± 0.005	33986 ± 1351	0.952			
Palmitoyl glycine	0.131 ± 0.008	28525 ± 2534	0.799			
Palmitoyl-DL-Carnitine chloride	0.120 ± 0.003	32151 ± 4085	0.900			
Palmitoylethanolamide	0.098 ± 0.010	42468 ± 3902	1.189			
Palmitylethanolamide	0.103 ± 0.005	31945 ± 1478	0.894			
Palonosetron hydrochloride	0.114 ± 0.007	58136 ± 3275	1.628			
Palustrol	0.121 ± 0.001	35503 ± 2383	0.994			
Pamabrom	0.099 ± 0.013	32012 ± 870	0.896			
Pamidronic acid	0.124 ± 0.006	38450 ± 3796	1.077			
p-Aminoclonidine hydrochloride	0.098 ± 0.002	34221 ± 2956	0.958			
Panaxadiol	0.109 ± 0.003	47088 ± 1049	1.398			

Panaxatriol	0.136 ±0.003	39417 ±2124	1.104			
Pancuronium	0.106 ±0.005	40362 ±4397	1.130			
Pancuronium bromide	0.101 ±0.010	34194 ±2826	0.957			
Pandamarilactonine B	0.117 ±0.005	47625 ±5880	1.333			
Pangamic acid sodium	0.102 ±0.005	33725 ±2041	0.944			
Paniculidine A	0.118 ±0.002	35694 ±4027	0.999			
Paniculidine B	0.123 ±0.008	45125 ±3313	1.263			
Paniculidine C	0.118 ±0.007	46511 ±4197	1.302			
Paniculoside I	0.119 ±0.003	43462 ±5420	1.217			
Paniculoside II	0.135 ±0.011	33371 ±3301	0.934			
Pantethine	0.109 ±0.009	32058 ±2041	0.898			
Panthenol (D)	0.118 ±0.008	45719 ±1051	1.280			
Panthenol (dl)	0.103 ±0.020	27735 ±7216	0.777			
Pantoprazole	0.102 ±0.009	64794 ±5905	1.843			
Pantoprazole Sodium	0.095 ±0.006 *					
Pantothenic acid(d)-na salt	0.101 ±0.007	28214 ±5774	0.790			
Papaverine hydrochloride	0.111 ±0.003	18849 ±239	0.560			
PAPP	0.113 ±0.005	38702 ±1554	1.084			
Parachlorophenol	0.093 ±0.005 *					
Paramethadione	0.103 ±0.002	37756 ±5437	1.057			
Paraoxon	0.107 ±0.007	30543 ±913	0.855			
Paraxanthine	0.107 ±0.003	31607 ±3953	0.885			
Parbendazole	0.110 ±0.019	27337 ±1140	0.765			
Parecoxib Na	0.125 ±0.003	34220 ±3533	0.958			
Pargyline hydrochloride	0.105 ±0.004	51996 ±7598	1.456			
Paromomycin sulfate	0.105 ±0.012	27330 ±1183	0.765			
Paroxetine	0.122 ±0.002	20430 ±1652	0.569			
Paroxetine hydrochloride	0.107 ±0.016	27970 ±5837	0.783			
Paroxetine hydrochloride hemihydrate	0.104 ±0.003	19961 ±2276	0.552			
Paroxetine maleate	0.115 ±0.008	27006 ±3683	0.756			
Paroxypropione	0.105 ±0.009	50584 ±7223	1.416			
PARP Inhibitor I, 3-ABA	0.092 ±0.003 *					

PARP Inhibitor VI, NU1025	0.118 ± 0.015	45465 ± 4025	1.273			
PARP Inhibitor VIII, PJ34	0.100 ± 0.007	45809 ± 4287	1.283			
PARP Inhibitor XI, DR2313	0.111 ± 0.019	35214 ± 2931	0.986			
PARP Inhibitor XII	0.102 ± 0.005	37979 ± 2251	1.063			
Parthenolide	0.083 ± 0.004 **					
Pashanone	0.121 ± 0.008	36847 ± 3592	1.032			
pasiniazid	0.107 ± 0.004	25178 ± 334	0.705			
Patulin	0.062 ± 0.002 **					
Paulownin	0.117 ± 0.001	32982 ± 1892	0.923			
Paxilline	0.111 ± 0.005	53108 ± 2558	1.487			
Paxiphylline D	0.116 ± 0.004	45214 ± 1878	1.266			
Paxiphylline E	0.121 ± 0.004	35687 ± 1205	0.999			
Pazopanib	0.126 ± 0.003	38990 ± 3729	1.092			
Pazufloxacin	0.062 ± 0.007 **					
pazufloxacin mesylate	0.063 ± 0.021 **					
p-Benzoquinone	0.105 ± 0.002	35785 ± 3187	1.002			
PCA 4248	0.113 ± 0.005	33924 ± 458	0.950			
PCO-400	0.110 ± 0.001	36838 ± 3237	1.031			
p-Coumaric acid ethyl ester	0.119 ± 0.011	45600 ± 2645	1.277			
PD 04217903	0.130 ± 0.020	32709 ± 1681	0.916			
PD 102807	0.111 ± 0.027	30887 ± 1967	0.865			
PD 123319 ditrifluoroacetate	0.113 ± 0.004	36804 ± 623	1.030			
PD 144795	0.068 ± 0.003 **					
PD 158780	0.121 ± 0.009	21113 ± 1457	0.591			
PD 160170	0.118 ± 0.016	29801 ± 5441	0.834			
PD 161570	0.112 ± 0.003	32810 ± 3019	0.919			
PD 166,948	0.111 ± 0.005	64219 ± 6768	1.798			
PD 166793	0.115 ± 0.002	51025 ± 2331	1.429			
PD 168077 maleate	0.113 ± 0.014	33567 ± 2176	0.940			
PD 169316	0.102 ± 0.007	43943 ± 2316	1.230			
PD 173955	0.126 ± 0.004	40087 ± 3080	1.122			
PD 174265	0.129 ± 0.003	30025 ± 1029	0.841			

PD 198306	0.118 ±0.012	39393 ±1005	1.103			
PD 404182	0.105 ±0.002	49951 ±3915	1.399			
PD 407824	0.130 ±0.012	21431 ±1963	0.600			
PD 81723	0.128 ±0.002	45379 ±2198	1.271			
PD 98059	0.107 ±0.004	34007 ±1267	0.952			
PDGF Receptor Tyrosine Kinase Inhibitor II	0.112 ±0.016	35499 ±2075	0.994			
PDGF Receptor Tyrosine Kinase Inhibitor III	0.110 ±0.001	29393 ±2694	0.823			
PDGF Receptor Tyrosine Kinase Inhibitor IV	0.104 ±0.007	28781 ±3788	0.806			
PDGF RTK Inhibitor	0.126 ±0.027	31926 ±1196	0.894			
PDK1/Akt/Flt Dual Pathway Inhibitor	0.114 ±0.006	29402 ±1947	0.823			
Pectolinarigenin	0.105 ±0.013	34658 ±2290	0.970			
Pectolinarin	0.113 ±0.007	35242 ±2016	0.987			
Pedatisectine F	0.113 ±0.006	37316 ±6155	1.045			
Pefloxacin mesylate	0.070 ±0.007 **					
Pefloxacine	0.104 ±0.009	42373 ±2425	1.186			
Pefloxacine mesylate	0.061 ±0.006 **					
Peganine	0.122 ±0.006	30785 ±609	0.862			
Peganole	0.105 ±0.003	46088 ±327	1.369			
Pelletierine hydrochloride	0.113 ±0.006	32899 ±2410	0.921			
Pellitorine	0.113 ±0.007	26607 ±335	0.745			
Pemetrexed	0.102 ±0.003	36085 ±3641	1.010			
Pemetrexed Disodium Heptahydrate	0.107 ±0.010	45323 ±3786	1.269			
Pemirolast potassium	0.104 ±0.004	25914 ±686	0.726			
Pemoline	0.121 ±0.004	31204 ±2340	0.874			
Pempidine tartrate	0.109 ±0.001	51905 ±1993	1.417			
Penbutolol sulfate	0.115 ±0.006	24250 ±1356	0.679			
Penciclovir	0.108 ±0.004	48831 ±2990	1.367			
Penduletin	0.130 ±0.014	35174 ±2868	0.985			
Penfluridol	0.058 ±0.007 **					
Penicillamine	0.118 ±0.005	35033 ±1636	0.981			
Penicillamine, L-	0.100 ±0.003	34554 ±639	0.967			
Penicillin g potassium	0.085 ±0.005 **					

Penicillin v potassium	0.086 ± 0.004 **					
Penitrem A	0.102 ± 0.005	52529 ± 1882	1.596			
Penta-acetate bergenin	0.118 ± 0.008	35765 ± 6074	1.001			
Pentadecanoic acid	0.110 ± 0.026	28462 ± 1722	0.797			
Pentagastrin	0.107 ± 0.003	26649 ± 2736	0.746			
Pentamidine	0.110 ± 0.004	9268 ± 1535	0.303	0.118 ± 0.011	36432 ± 3120	1.115
Pentamidine isethionate	0.102 ± 0.018	50262 ± 5940	1.407			
Pentetic acid	0.098 ± 0.013	159676 ± 3689	4.358	0.099 ± 0.005	98181 ± 10323	3.004
Pentobarbital	0.123 ± 0.006	41780 ± 350	1.170			
Pentolinium bitartrate	0.119 ± 0.005	47863 ± 6011	1.340			
Pentolinium di[L-(+)-tartrate]	0.094 ± 0.011	37619 ± 1339	1.053			
Pentoxifylline	0.102 ± 0.006	44063 ± 2639	1.234			
Pentylenetetrazol	0.106 ± 0.011	29304 ± 3581	0.820			
Pentylenetetrazole	0.101 ± 0.011	44512 ± 4092	1.246			
Peoniflorin	0.103 ± 0.009	34255 ± 972	0.959			
Peonol methyl ether	0.114 ± 0.002	50214 ± 9282	1.406			
Pepluanin A	0.123 ± 0.021	38160 ± 2672	1.068			
Pepstatin	0.108 ± 0.003	32614 ± 1125	0.913			
Pepstatin A	0.117 ± 0.019	32192 ± 2989	0.901			
Perakine	0.126 ± 0.002	36797 ± 3071	1.030			
Peraksine	0.114 ± 0.006	29754 ± 2173	0.833			
Perforatumone	0.115 ± 0.003	50710 ± 3880	1.420			
Pergolide mesylate	0.107 ± 0.001	42611 ± 2794	1.193			
Pergolide methanesulfonate	0.105 ± 0.017	42546 ± 2223	1.191			
Perhexiline maleate	0.117 ± 0.007	48339 ± 2333	1.353			
Periciazine	0.114 ± 0.008	27542 ± 2967	0.771			
Periglaucine A	0.126 ± 0.019	42237 ± 4762	1.183			
Periglaucine B	0.120 ± 0.002	35674 ± 2501	0.999			
Perillic acid	0.117 ± 0.010	24018 ± 643	0.672			
Perillic acid (-)	0.097 ± 0.002	32158 ± 1347	0.900			
Perillyl alcohol	0.109 ± 0.024	42405 ± 2204	1.187			
Perindopril	0.118 ± 0.007	37463 ± 1969	1.049			

Perindopril erbumine	0.084 ± 0.003 **					
Perindopril tert-Butylamine salt	0.103 ± 0.011	37684 ± 3570	1.055			
Periplogenin	0.116 ± 0.007	42741 ± 3993	1.197			
Permethrin	0.103 ± 0.007	45729 ± 5421	1.280			
Perospirone hydrochloride	0.113 ± 0.008	103042 ± 11375	2.869	0.116 ± 0.009	37684 ± 4195	1.153
Perphenazine	0.093 ± 0.008	31649 ± 264	0.886			
Perseitol	0.107 ± 0.002	35658 ± 3254	0.998			
Peruvoside	0.101 ± 0.005	50620 ± 1488	1.417			
Petcm	0.106 ± 0.015	47152 ± 4028	1.320			
Peuarenin	0.127 ± 0.007	31608 ± 6904	0.885			
Peucedanin	0.106 ± 0.014	44177 ± 2689	1.237			
Peucenin	0.107 ± 0.005	33584 ± 1851	0.940			
PF431396	0.123 ± 0.006	43182 ± 5259	1.209			
PF562271	0.121 ± 0.004	34503 ± 2438	0.966			
p-Fluoro-L-phenylalanine	0.103 ± 0.005	25797 ± 877	0.722			
PG-9 maleate	0.109 ± 0.001	28844 ± 3636	0.808			
PHA 665752	0.121 ± 0.007	28866 ± 1728	0.808			
Phaclofen	0.099 ± 0.001	33690 ± 1960	0.943			
Phaseollidin	0.126 ± 0.002	36733 ± 1792	1.029			
Phccc	0.112 ± 0.003	45131 ± 2264	1.264			
Phebalosin	0.122 ± 0.001	41582 ± 1605	1.164			
Phellamurin	0.107 ± 0.010	43841 ± 676	1.228			
Phellochin	0.134 ± 0.004	26406 ± 325	0.739			
Phenacemide	0.109 ± 0.003	52349 ± 3198	1.466			
Phenacetin	0.110 ± 0.002	42907 ± 2513	1.201			
Phenacylamine hydrochloride	0.097 ± 0.017	28766 ± 3053	0.805			
Phenamil	0.107 ± 0.002	33581 ± 2213	0.940			
Phenamil methanesulfonate	0.105 ± 0.015	36026 ± 2225	1.009			
Phenanthridinone [6(5H)-Phenanthridinone]	0.109 ± 0.004	27445 ± 1535	0.768			
Phenazopyridine hydrochloride	0.105 ± 0.009	27757 ± 2290	0.777			
Phenelzine sulfate	0.106 ± 0.007	30136 ± 2251	0.844			
Phenethicillin potassium	0.102 ± 0.006	24805 ± 1148	0.695			

Phenethyl caffeate (cape)	0.076 ±0.007 **					
Phenethylamine hydrochloride	0.102 ±0.009	24544 ±1418	0.687			
Phenformin Hydrochloride	0.105 ±0.011	47450 ±3837	1.329			
Phenidone	0.100 ±0.003	21761 ±2662	0.609			
Phenindione	0.110 ±0.005	55510 ±1470	1.515			
Pheniramine maleate	0.107 ±0.015	40241 ±2320	1.127			
Phenolphthalein	0.101 ±0.012	34343 ±2457	0.962			
Phenothiazine	0.124 ±0.004	66422 ±4139	1.849			
Phenothrin	0.104 ±0.008	43381 ±4898	1.215			
Phenoxybenzamine	0.111 ±0.003	33209 ±1080	0.930			
Phenoxybenzamine hydrochloride	0.121 ±0.008	33826 ±2872	0.947			
Phenoxymethylpenicillin	0.111 ±0.019	44627 ±7542	1.250			
Phenprobamate	0.128 ±0.003	42884 ±2628	1.201			
Phenserine	0.100 ±0.002	40953 ±3909	1.147			
Phensuccimide	0.102 ±0.008	41348 ±6702	1.158			
Phensuximide	0.110 ±0.009	48553 ±2240	1.359			
Phentermine hydrochloride	0.107 ±0.016	29289 ±1977	0.820			
Phentolamine	0.109 ±0.005	30160 ±541	0.844			
Phentolamine hydrochloride	0.120 ±0.005	38341 ±3646	1.074			
Phentolamine mesylate	0.108 ±0.005	37064 ±3461	1.038			
Phentolaminehydrochloride	0.111 ±0.001	39071 ±3136	1.094			
phenyl aminosalicylate	0.106 ±0.008	50625 ±1515	1.417			
phenylalaniine (L)-hydrochloride	0.107 ±0.009	26318 ±731	0.737			
Phenylalanine betaine	0.116 ±0.005	32096 ±521	0.899			
Phenylbenzene-omega-phosphono-α-amino acid	0.105 ±0.007	35368 ±954	0.990			
Phenylbutazone	0.106 ±0.007	55045 ±2572	1.521			
Phenylbutyrate Na	0.090 ±0.002 **					
Phenylephrine hydrochloride	0.097 ±0.010	32133 ±3407	0.900			
Phenylethyl alcohol	0.109 ±0.004	24516 ±4742	0.686			
Phenylmercuric acetate	0.053 ±0.007 **					
Phenylpropanolamine	0.110 ±0.004	31840 ±4552	0.892			
Phenylpropanolamine hydrochloride	0.109 ±0.008	39356 ±1841	1.102			

Phenytoin	0.112 ± 0.002	38494 ± 1854	1.078			
Phenytoin sodium	0.100 ± 0.008	36440 ± 904	1.020			
Phlegmanol C	0.126 ± 0.003	35125 ± 2957	0.983			
phloracetophenone	0.113 ± 0.013	8230 ± 347	0.237	0.115 ± 0.006	27280 ± 1543	0.835
Phloretin	0.109 ± 0.006	116516 ± 12968	3.221	0.122 ± 0.003	49023 ± 5519	1.500
Phloridzin	0.125 ± 0.001	26444 ± 1425	0.740			
Phlorigidoside B	0.121 ± 0.008	46731 ± 4909	1.308			
Phlorin	0.122 ± 0.024	39017 ± 2402	1.092			
Phlorizine	0.103 ± 0.005	20024 ± 1033	0.595			
Phorbol	0.102 ± 0.004	20196 ± 182	0.600			
Phorbol 12 myristate 13 acetate	0.109 ± 0.009	22199 ± 785	0.622			
Phorbol 12,13-dibutyrate	0.095 ± 0.019	33630 ± 398	0.942			
Phorbol 12-myristate 13-acetate	0.101 ± 0.004	36800 ± 6226	1.030			
Phorbol 12-myristate 13-acetate, 4-a -	0.102 ± 0.001	43878 ± 1747	1.229			
Phorbol-12-myristate-13-acetate	0.097 ± 0.006	45297 ± 5020	1.268			
Phosphatidic acid, dipalmitoyl	0.107 ± 0.001	36075 ± 1058	1.010			
Phospho-Glycogen Synthase Peptide-2	0.107 ± 0.002	37211 ± 1336	1.042			
Phospholipase Activators	0.096 ± 0.004	122392 ± 16570	3.581	0.103 ± 0.011	35742 ± 3615	1.094
Phosphomycin disodium	0.101 ± 0.012	30180 ± 1299	0.845			
Phosphonoacetic acid	0.107 ± 0.001	46736 ± 6030	1.309			
Phosphoramidon	0.122 ± 0.002	28608 ± 2102	0.801			
Phosphoramidon disodium	0.108 ± 0.006	47594 ± 790	1.333			
Phthalylsulfacetamide	0.099 ± 0.003	49960 ± 1663	1.399			
Phthalylsulfapyridazine	0.103 ± 0.001	28440 ± 3505	0.796			
Phthalylsulfathiazole	0.102 ± 0.003	41309 ± 949	1.157			
Phthiocol	0.087 ± 0.005 **					
Phtpp	0.111 ± 0.002	31520 ± 1136	0.883			
Phyllanthin	0.120 ± 0.007	40585 ± 3709	1.136			
Phyllostadimer A	0.129 ± 0.003	34288 ± 1857	0.960			
Physcion	0.117 ± 0.006	32124 ± 1449	0.899			
Physostigmine	0.111 ± 0.003	26925 ± 514	0.754			
physostigmine salicylate	0.102 ± 0.004	44143 ± 5148	1.236			

Physostigmine sulfate	0.101 ± 0.006	26848 ± 4147	0.752			
Phytol	0.126 ± 0.003	35875 ± 912	1.004			
Phytolaccagenin	0.117 ± 0.002	39646 ± 6142	1.110			
Phytolaccoside B	0.112 ± 0.006	38724 ± 2688	1.084			
phytonadione	0.104 ± 0.007	26721 ± 4374	0.748			
Phytosphingosine	0.102 ± 0.002	22191 ± 827	0.621			
PI 3-Kb Inhibitor II	0.113 ± 0.011	39382 ± 4657	1.103			
PI 3-Kg Inhibitor	0.118 ± 0.014	39265 ± 1812	1.099			
PI 3-Kα Inhibitor IV	0.112 ± 0.023	36086 ± 2413	1.010			
PI 3-Kα Inhibitor VIII	0.123 ± 0.004	33829 ± 2021	0.947			
PI 3-Kβ Inhibitor VI, TGX-221	0.130 ± 0.018	24765 ± 2441	0.693			
PI 3-Kγ Inhibitor VII	0.122 ± 0.006	40193 ± 319	1.125			
PI 3-Kγ/CKII Inhibitor	0.112 ± 0.004	16945 ± 1106	0.502			
PI 828	0.110 ± 0.005	33973 ± 2389	0.951			
PI-103	0.119 ± 0.006	28094 ± 991	0.787			
PI-93	0.117 ± 0.002	31358 ± 9266	0.878			
Piceatannol	0.117 ± 0.004	38290 ± 1706	1.072			
PICEID	0.125 ± 0.004	49922 ± 429	1.398			
Picolamine	0.109 ± 0.007	33687 ± 3601	0.943			
Piconol	0.106 ± 0.011	25442 ± 2437	0.712			
Picotamide	0.107 ± 0.008	46412 ± 4223	1.300			
Picralinal	0.122 ± 0.008	47366 ± 936	1.326			
Picrasidine I	0.081 ± 0.004 **					
Picrasidine J	0.132 ± 0.006	21895 ± 5305	0.613			
Picrasidine S	0.053 ± 0.002 **					
Picrasidine T	0.125 ± 0.006	31661 ± 1239	0.886			
Picrasin B	0.115 ± 0.004	38882 ± 4169	1.089			
Picrasin B acetate	0.121 ± 0.002	38261 ± 1433	1.071			
Picrasinol B	0.125 ± 0.001	32641 ± 2240	0.914			
Picrinine	0.127 ± 0.004	25732 ± 1244	0.720			
picropodophyllin	0.113 ± 0.003	41467 ± 6023	1.161			
picropodophyllin acetate	0.108 ± 0.008	29929 ± 597	0.838			

Picrotin	0.103 ± 0.010	25274 ± 353	0.708			
Picrotin -Picrotoxinin	0.109 ± 0.003	57507 ± 4468	1.601			
Picrotoxin	0.103 ± 0.009	26550 ± 4453	0.743			
Picrotoxinin	0.102 ± 0.008	31574 ± 2383	0.884			
pidolic acid	0.116 ± 0.006	25309 ± 2168	0.709			
Pidotimod	0.118 ± 0.007	27733 ± 2912	0.777			
Pifithrin	0.107 ± 0.003	34646 ± 5377	0.970			
Pifithrin->m	0.107 ± 0.028	14306 ± 165	0.396	0.113 ± 0.003	34182 ± 2109	1.046
Pifithrin-mu	0.095 ± 0.014	25383 ± 759	0.711			
Pifithrin-α hydrobromide	0.113 ± 0.004	37509 ± 3425	1.050			
Pifithrin-α, Cyclic-	0.091 ± 0.004 *					
Pifithrin-μ	0.088 ± 0.004 *					
PIH	0.104 ± 0.005	91999 ± 9837	2.692	0.104 ± 0.007	51355 ± 7327	1.571
PIK-75	0.134 ± 0.004	26064 ± 3807	0.730			
PIK-90	0.115 ± 0.004	18918 ± 2032	0.562			
PIKfyve Inhibitor	0.114 ± 0.012	42167 ± 1516	1.181			
Pilocarpine hydrochloride	0.103 ± 0.004	40584 ± 5808	1.136			
Pilocarpine nitrate	0.103 ± 0.008	38449 ± 3799	1.077			
PIM 1 Inhibitor 2	0.112 ± 0.004	37178 ± 4124	1.041			
PIM1 Kinase Inhibitor II	0.118 ± 0.006	19518 ± 1649	0.546			
PIM1 Kinase Inhibitor IV	0.119 ± 0.015	36801 ± 5946	1.030			
PIM1/2 Kinase Inhibitor V	0.087 ± 0.004 **					
PIM1/2 Kinase Inhibitor VI	0.116 ± 0.004	47915 ± 971	1.342			
Pimagedine hydrochloride	0.110 ± 0.003	31372 ± 2717	0.878			
Pimaric acid	0.124 ± 0.003	30941 ± 1055	0.866			
Pimaricin	0.126 ± 0.002	40524 ± 1628	1.135			
Pimethixene maleate	0.116 ± 0.005	54700 ± 8538	1.532			
Pimobendan	0.082 ± 0.005 **					
Pimozide	0.100 ± 0.007	32324 ± 2455	0.905			
Pimpinellin	0.122 ± 0.001	41481 ± 2868	1.161			
Pinacidil	0.104 ± 0.003	37755 ± 3082	1.057			
Pindolol	0.106 ± 0.009	24235 ± 1604	0.679			

Pinobanksin	0.103 ± 0.006	38497 ± 4000	1.078			
Pinocembrin	0.109 ± 0.007	63473 ± 3123	1.885			
Pinocembrin 7-acetate	0.119 ± 0.005	37614 ± 2419	1.053			
Pinocembrin diacetate	0.109 ± 0.019	37953 ± 1309	1.063			
Pinostilbene	0.104 ± 0.010	53082 ± 1972	1.486			
Pinostilbenoside	0.121 ± 0.004	39564 ± 1790	1.108			
Pinostrobin	0.132 ± 0.006	37050 ± 350	1.037			
Pinostrobin chalcone	0.121 ± 0.002	41230 ± 2121	1.154			
Pinosylvin	0.120 ± 0.006	52219 ± 3112	1.462			
Pinusolide	0.126 ± 0.001	34556 ± 611	0.968			
Pinusolidic acid	0.135 ± 0.014	34273 ± 2102	0.960			
p-Iodoclonidine hydrochloride	0.106 ± 0.001	34590 ± 4487	0.969			
Pioglitazone	0.135 ± 0.004 **					
Pioglitazone hydrochloride	0.129 ± 0.004	28451 ± 1397	0.797			
Pipamperone	0.102 ± 0.001	29867 ± 1438	0.836			
Pipemidic acid	0.108 ± 0.004	48716 ± 8394	1.364			
Pipenzolate bromide	0.109 ± 0.004	64074 ± 8374	1.749			
Piperacetazine	0.107 ± 0.013	21610 ± 1422	0.605			
Piperacillin	0.113 ± 0.017	34158 ± 5182	0.956			
Piperacillin sodium	0.100 ± 0.011	19354 ± 2279	0.557			
Piperazide	0.112 ± 0.011	29453 ± 7186	0.825			
Piperic acid	0.107 ± 0.003	38588 ± 2197	1.080			
Piperidolate hydrochloride	0.112 ± 0.003	31376 ± 1709	0.879			
Piperine	0.104 ± 0.001	37396 ± 61	1.047			
Piperonyl butoxide	0.110 ± 0.009	36063 ± 3417	1.010			
Piplartine	0.116 ± 0.004	31815 ± 1770	0.891			
Pipobroman	0.110 ± 0.006	30002 ± 1332	0.840			
Piracetam	0.096 ± 0.012	43068 ± 7982	1.206			
Pirenperone	0.106 ± 0.005	36779 ± 881	1.030			
Pirenzepine dihydrochloride	0.100 ± 0.011	30701 ± 1958	0.860			
Piretanide	0.110 ± 0.001	42601 ± 2728	1.193			
Pirfenidone	0.095 ± 0.010	34371 ± 2823	0.962			

Piribedil	0.115 ±0.007	56322 ±9460	1.577			
Piribedil hydrochloride	0.108 ±0.003	40694 ±2204	1.139			
Piribedil maleate	0.098 ±0.010	38576 ±1019	1.080			
Pirlindole mesylate	0.121 ±0.004	33463 ±1509	0.937			
Piroctone olamine	0.067 ±0.010 **					
Piromidic acid	0.107 ±0.004	39307 ±3804	1.101			
Piroxicam	0.101 ±0.008	26541 ±3644	0.743			
Piscidic acid	0.114 ±0.003	35891 ±1274	1.005			
Piscidinol A	0.130 ±0.005	42848 ±1064	1.200			
PIT	0.100 ±0.006	32526 ±1855	0.911			
Pitavastatin calcium	0.097 ±0.006	33098 ±2274	0.927			
Pivampicillin	0.125 ±0.002	39062 ±794	1.094			
Pivmecillinam hydrochloride	0.116 ±0.010	36481 ±4096	1.021			
Pizotifen malate	0.111 ±0.012	31521 ±8078	0.883			
Pizotyline	0.114 ±0.007	23802 ±3584	0.666			
Pizotyline malate	0.113 ±0.008	23412 ±1709	0.656			
PK 11195	0.105 ±0.003	50256 ±7601	1.407			
PKC-412	0.117 ±0.003	34876 ±1857	0.977			
PKCb Inhibitor	0.132 ±0.007	20269 ±1251	0.568			
PKCbII/EGFR Inhibitor	0.107 ±0.004	33004 ±723	0.924			
PKR Inhibitor	0.106 ±0.003	22997 ±2889	0.644			
PKSI-527	0.120 ±0.002	28900 ±2000	0.809			
Platyphyllenone	0.114 ±0.001	45522 ±3507	1.275			
Platyphyllonol	0.112 ±0.009	38226 ±564	1.070			
Plicamycin	0.081 ±0.002 **					
Plumbagin	0.073 ±0.009 **					
Plumieride	0.130 ±0.004	34977 ±261	0.979			
PLX4720	0.120 ±0.004	45316 ±762	1.346			
p-Menth-8-ene-1,2-diol	0.128 ±0.012	48943 ±2882	1.370			
p-Menthan-1,3,8-triol	0.125 ±0.001	29738 ±224	0.833			
p-Menthane-1,2,8-triol	0.121 ±0.002	44642 ±2874	1.250			
p-Methoxyphenylethylamine	0.110 ±0.009	37013 ±3296	1.036			

PMPA (NAALADase inhibitor)	0.113 ±0.005	40566 ±4043	1.136			
p-MPPF dihydrochloride	0.103 ±0.002	56635 ±6396	1.586			
p-MPPI hydrochloride	0.096 ±0.020	3194 ±332	0.088	0.102 ±0.007	17816 ±2957	0.545
PNU 120596	0.111 ±0.002	40570 ±3729	1.136			
PNU 22394 hydrochloride	0.112 ±0.007	25980 ±3437	0.727			
PNU 282987	0.106 ±0.001	36187 ±954	1.013			
PNU 37883 hydrochloride	0.111 ±0.006	36003 ±4619	1.008			
PNU 74654	0.127 ±0.021	35110 ±3612	0.983			
PNU-282987	0.097 ±0.003	40159 ±3732	1.124			
PNU-37883A	0.104 ±0.008	33822 ±2126	0.947			
Podocarpic acid	0.099 ±0.001 *					
Podocarpusflavone A	0.117 ±0.009	37981 ±8860	1.063			
Podofilox	0.105 ±0.005	19811 ±1876	0.555			
Podophyllin acetate	0.113 ±0.004	30591 ±770	0.857			
Podophyllotoxin	0.109 ±0.002	44540 ±3259	1.247			
Podophylotoxin	0.122 ±0.005	35410 ±3635	0.991			
Polo-like Kinase Inhibitor I	0.127 ±0.004	39339 ±2095	1.101			
Polo-like Kinase Inhibitor II, BTO-1	0.105 ±0.003	47572 ±4262	1.332			
Polygonal	0.115 ±0.002	49067 ±4660	1.374			
Polymyxin b sulfate	0.069 ±0.005 **					
Pomiferin	0.110 ±0.007	35238 ±2062	0.987			
Pomiferin dimethyl ether	0.113 ±0.004	40856 ±5166	1.144			
Pomiferin triacetate	0.110 ±0.004	50037 ±8568	1.401			
Pomiferin trimethyl ether	0.116 ±0.003	42844 ±6072	1.200			
Pomolic acid	0.095 ±0.016	33445 ±3586	0.936			
Pomolic acid 28-O-β-D-glucopyranosyl ester	0.119 ±0.002	47512 ±3613	1.330			
Ponasterone A	0.126 ±0.008	52701 ±5866	1.476			
Porson	0.112 ±0.007	40575 ±2701	1.136			
Potassium clavulanate	0.112 ±0.001	25231 ±1271	0.706			
Potassium p-aminobenzoate	0.113 ±0.006	42566 ±2553	1.192			
PP1	0.123 ±0.004	31530 ±1684	0.883			
PP1 Analog II, 1NM-PP1	0.114 ±0.024	36006 ±1695	1.008			

PP2	0.120 ± 0.003	36790 ± 3874	1.030			
PP3	0.104 ± 0.004	34529 ± 1410	0.967			
Ppack	0.119 ± 0.007	44316 ± 2210	1.241			
Ppads	0.104 ± 0.006	23345 ± 1133	0.645			
Ppads tetrasodium	0.111 ± 0.005	22245 ± 5072	0.623			
p-Phlorizin	0.126 ± 0.005	29130 ± 2472	0.816			
PPIase-Parvulin Inhibitor	0.114 ± 0.013	42901 ± 4039	1.201			
Ppnds tetrasodium	0.100 ± 0.007	21341 ± 367	0.590			
PPT	0.075 ± 0.006 **					
PQ 401	0.104 ± 0.008	34314 ± 2352	0.961			
Practolol	0.114 ± 0.009	24885 ± 4047	0.697			
Pralidoxime chloride	0.111 ± 0.007	27556 ± 7606	0.772			
Pramipexole	0.112 ± 0.004	27275 ± 3094	0.764			
Pramoxine hydrochloride	0.128 ± 0.007	35547 ± 599	0.995			
Pranlukast	0.116 ± 0.004	34473 ± 1099	0.965			
Pranoprofen	0.116 ± 0.010	35051 ± 2384	0.981			
Prasterone	0.112 ± 0.004	36955 ± 2405	1.035			
Prasterone acetate	0.107 ± 0.001	54603 ± 2654	1.573			
Prasugrel	0.099 ± 0.004	21500 ± 692	0.602			
Pratol	0.104 ± 0.027	37125 ± 1061	1.039			
Pravadoline	0.115 ± 0.007	3217 ± 337	0.091	0.122 ± 0.007	20742 ± 2166	0.635
Pravastatin	0.115 ± 0.027	36784 ± 1876	1.030			
Pravastatin lactone	0.109 ± 0.002	41373 ± 5448	1.158			
Pravastatin sodium salt	0.104 ± 0.005	31520 ± 2145	0.883			
Praziquantel	0.103 ± 0.011	29244 ± 1743	0.819			
Prazocin	0.118 ± 0.003	26955 ± 912	0.755			
Prazosin hydrochloride	0.107 ± 0.007	52894 ± 3625	1.481			
PRE-084	0.112 ± 0.001	47747 ± 5168	1.337			
PRE-084 hydrochloride	0.105 ± 0.001	25179 ± 632	0.705			
Prednicarbate	0.115 ± 0.004	37197 ± 3063	1.041			
Prednisolone	0.114 ± 0.003	30122 ± 2429	0.843			
Prednisolone acetate	0.109 ± 0.011	37589 ± 894	1.052			

Prednisolone hemisuccinate	0.111 ±0.004	54142 ±4872	1.516			
prednisolone sodium phosphate	0.103 ±0.008	20611 ±2679	0.577			
Prednisolone trimethylacetate	0.111 ±0.012	52725 ±6126	1.476			
Prednisolone-21-propionate	0.111 ±0.006	28464 ±2577	0.797			
Prednisone	0.114 ±0.001	36671 ±2245	1.027			
Prednisone acetate	0.108 ±0.006	47694 ±3782	1.335			
Pregabalin	0.104 ±0.005	23442 ±1008	0.656			
Pregnenolone	0.116 ±0.017	32902 ±5057	0.921			
Pregnenolone 16a carbonitrile	0.108 ±0.003	34385 ±4768	0.963			
pregnenolone succinate	0.100 ±0.012	25356 ±2914	0.710			
Pregnenolone sulfate sodium	0.102 ±0.008	30954 ±3614	0.867			
Pregnenolone-16a-carbonitrile	0.118 ±0.003	53439 ±2180	1.496			
Preisocalamendiol	0.116 ±0.013	41526 ±4362	1.163			
Prenylamine lactate	0.100 ±0.007	31747 ±1464	0.889			
Prenyletin	0.102 ±0.004	18211 ±1315	0.525			
Prenylpiperitol	0.119 ±0.008	43345 ±2195	1.214			
Pre-schisanartanin B	0.120 ±0.005	34466 ±335	0.965			
pridinol methanesulfonate	0.105 ±0.005	41259 ±3617	1.155			
Prilocaine hydrochloride	0.098 ±0.001	26903 ±5718	0.753			
Prima-1	0.112 ±0.011	16270 ±1794	0.513			
Primaquine diphosphate	0.115 ±0.004	32435 ±393	0.908			
Primidone	0.106 ±0.008	33729 ±364	0.944			
prim-O-Glucosylangelicain	0.123 ±0.003	25629 ±2260	0.718			
Primuletin	0.111 ±0.006	33675 ±2595	0.943			
Pristimerin	0.107 ±0.016	42359 ±460	1.186			
PRL-3 Inhibitor	0.093 ±0.006	18744 ±625	0.548			
Proadifen hydrochloride	0.110 ±0.019	42571 ±5511	1.192			
Probenecid	0.109 ±0.001	43267 ±2031	1.211			
Probucol	0.118 ±0.001	55429 ±6947	1.552			
Procainamide	0.112 ±0.004	40481 ±2339	1.133			
Procainamide hydrochloride	0.108 ±0.003	47455 ±1301	1.329			
Procaine hydrochloride	0.098 ±0.007	29446 ±1735	0.824			

Procarbazine hydrochloride	0.127 ± 0.009	31417 ± 2559	0.880			
Procaterol hydrochloride	0.121 ± 0.012	42595 ± 4070	1.193			
Prochlorperazine dimaleate	0.102 ± 0.010	20380 ± 1869	0.563			
prochlorperazine edisylate	0.110 ± 0.006	21788 ± 2255	0.610			
Procodazole	0.108 ± 0.008	40310 ± 4123	1.129			
Procodazole-ethyl-ester	0.113 ± 0.006	47417 ± 4519	1.328			
Procyclidine hydrochloride	0.119 ± 0.008	30998 ± 1258	0.868			
Procysteine	0.116 ± 0.021	38777 ± 8358	1.086			
proflavine hemisulfate	0.068 ± 0.006 **					
Progesterone	0.117 ± 0.008	40462 ± 6563	1.133			
Proglumide	0.104 ± 0.004	44580 ± 8452	1.248			
Proguanil hydrochloride	0.130 ± 0.006	52146 ± 4101	1.460			
Proline	0.113 ± 0.007	24098 ± 4677	0.675			
Promazine hydrochloride	0.102 ± 0.002	30932 ± 2172	0.866			
Prometaphanine	0.116 ± 0.003	28189 ± 1465	0.789			
Promethazine hydrochloride	0.098 ± 0.003	27946 ± 5600	0.782			
pronetalol hydrochloride	0.111 ± 0.009	30143 ± 2502	0.844			
Propafenone	0.115 ± 0.002	55353 ± 3006	1.682			
Propafenone hydrochloride	0.105 ± 0.007	33215 ± 3940	0.930			
Propantheline bromide	0.108 ± 0.004	41927 ± 6361	1.174			
Proparacaine hydrochloride	0.112 ± 0.002	42924 ± 8180	1.202			
Propentofylline	0.106 ± 0.009	37814 ± 3993	1.059			
Propidium iodide	0.133 ± 0.006 *					
Propiolactone	0.100 ± 0.010	18108 ± 2079	0.522			
Propionylpromazine hydrochloride	0.118 ± 0.005					
Propofol	0.096 ± 0.004	31065 ± 2991	0.870			
Propoxur	0.117 ± 0.003	43279 ± 1406	1.212			
Propoxycaine hydrochloride	0.108 ± 0.007	36641 ± 1731	1.026			
Propranolol	0.113 ± 0.003	29411 ± 3772	0.823			
Propranolol (S-)	0.116 ± 0.001	25028 ± 1318	0.701			
Propranolol glycol	0.125 ± 0.009	26108 ± 2739	0.731			
Propranolol hydrochloride	0.101 ± 0.004	21167 ± 1055	0.593			

Propranolol hydrochloride s(-)	0.113 ±0.006	35245 ±221	0.987			
Propyl gallate	0.088 ±0.004 **					
Propylthiouracil	0.125 ±0.011	27859 ±2214	0.780			
Propyl-β-carboline-3-carboxylate	0.103 ±0.006	62856 ±3032	1.760			
Proscillaridin	0.110 ±0.002	29412 ±5280	0.824			
Proscillaridin A	0.120 ±0.005	31128 ±1013	0.872			
Prostaglandin A1	0.091 ±0.002 **					
Prostaglandin A2	0.119 ±0.011	40077 ±2765	1.122			
Prostaglandin B1	0.103 ±0.003	28420 ±1376	0.796			
Prostaglandin B2	0.100 ±0.006	30636 ±657	0.858			
Prostaglandin D2	0.111 ±0.004	37738 ±586	1.057			
Prostaglandin E1	0.107 ±0.004	60580 ±6302	1.869			
Prostaglandin E2	0.107 ±0.012	28512 ±741	0.798			
Prostaglandin F1a	0.102 ±0.005	26665 ±974	0.747			
Prostaglandin F2a	0.110 ±0.006	38040 ±1218	1.065			
Prostaglandin I2 Na	0.117 ±0.008	40328 ±1270	1.129			
Prostaglandin J2	0.102 ±0.008	44027 ±2001	1.233			
Prostephanaberrine	0.125 ±0.002	30628 ±1490	0.858			
Protein Methyltransferase Inhibitor, AMI-5	0.098 ±0.005	24066 ±1838	0.674			
Prothionamide	0.121 ±0.003	18389 ±491	0.523			
Protionamide	0.117 ±0.003	18602 ±1208	0.536			
Protirelin	0.109 ±0.009	39673 ±5618	1.111			
Protocatechuic acid	0.111 ±0.003	37485 ±6224	1.050			
Protopine	0.116 ±0.001	45337 ±2843	1.269			
Protopine hydrochloride	0.100 ±0.004	26798 ±659	0.750			
protoporphyrin ix	0.111 ±0.005	38251 ±817	1.071			
Protoporphyrin IX disodium	0.101 ±0.004	24786 ±4020	0.694			
Protosappanin B	0.137 ±0.012	64691 ±9984	1.808			
Protoveratrine B	0.107 ±0.007	18814 ±404	0.559			
Protozide	0.118 ±0.022	28877 ±2330	0.809			
Protriptyline hydrochloride	0.109 ±0.006	32230 ±2373	0.902			
protryptyline hydrochloride	0.108 ±0.008	22252 ±981	0.623			

Proxyfan Oxalate	0.115 ± 0.002	32432 ± 2423	0.908			
Proxymetacaine Hydrochloride	0.104 ± 0.006	44475 ± 1794	1.245			
Proxyphylline	0.117 ± 0.002	36459 ± 2824	1.021			
Prudomestin	0.119 ± 0.010	42895 ± 947	1.201			
Prulifloxacin	0.051 ± 0.002 **					
Prunasin	0.122 ± 0.001	33291 ± 1871	0.932			
Prunin	0.133 ± 0.006	67390 ± 9814	1.884			
Przewalskin	0.123 ± 0.003	39449 ± 4214	1.105			
PSB 06126	0.115 ± 0.003	21986 ± 559	0.616			
PSB 11	0.102 ± 0.001	31969 ± 4148	0.895			
PSB 1115	0.109 ± 0.005	35384 ± 5727	0.991			
PSB 36	0.104 ± 0.001	32200 ± 4232	0.902			
Pseudo-anisatin	0.112 ± 0.002	37902 ± 5504	1.061			
Pseudoionone	0.117 ± 0.005	22745 ± 4080	0.637			
Pseudolaric acid D	0.110 ± 0.007	29068 ± 856	0.814			
Pseudopalmatine	0.135 ± 0.012	31042 ± 2831	0.869			
Pseudopelletierin hydrochloride	0.115 ± 0.006	31119 ± 1558	0.871			
Pseudotaraxasterol	0.113 ± 0.016	38819 ± 2925	1.087			
Psidial A	0.117 ± 0.005	38955 ± 2032	1.091			
PSN 375963 hydrochloride	0.108 ± 0.005	47420 ± 2295	1.328			
Psora-4	0.106 ± 0.005	36139 ± 2094	1.012			
Psoralen	0.123 ± 0.003	35924 ± 1567	1.006			
ptaeroxylin	0.118 ± 0.005	23472 ± 1615	0.657			
Ptelatoside B	0.124 ± 0.001	34686 ± 1631	0.971			
Pterodondiol	0.117 ± 0.003	38346 ± 1928	1.074			
Pterodontic acid	0.123 ± 0.004	39576 ± 2986	1.108			
Pteroside D	0.130 ± 0.008	28103 ± 2537	0.787			
Pterosin D	0.117 ± 0.002	40643 ± 1119	1.138			
Pterosin D 3-O-glucoside	0.122 ± 0.005	35829 ± 2227	1.003			
Pterosin Z	0.124 ± 0.006	32540 ± 2203	0.911			
Pterostilbene, Pterocarpus marsupium	0.107 ± 0.007	129599 ± 12832	3.792	0.106 ± 0.007	38106 ± 2248	1.166
Pteryxin	0.110 ± 0.016	23868 ± 4628	0.668			

PTP Inhibitor V, PHPS1	0.107 ± 0.015	19028 ± 1520	0.557			
PTP1B Inhibitor	0.101 ± 0.006	32007 ± 1662	0.896			
Puerarin	0.127 ± 0.010	31914 ± 707	0.894			
Purmorphamine	0.092 ± 0.014	36864 ± 3385	1.032			
Puromycin	0.099 ± 0.005	28162 ± 606	0.789			
Puromycin dihydrochloride	0.111 ± 0.000	40023 ± 1320	1.121			
Purpurin	0.085 ± 0.001 **					
Purpurogallin	0.079 ± 0.007 **					
Purpurogallin-4-carboxylic acid	0.082 ± 0.006 *					
Purvalanol A	0.130 ± 0.004	30569 ± 756	0.856			
Purvalanol B	0.126 ± 0.002	24163 ± 3446	0.677			
Putraflavone	0.135 ± 0.011	42044 ± 1985	1.177			
Putrescine dihydrochloride	0.104 ± 0.003	28965 ± 1172	0.811			
p-Vinylphenyl O-[β-D-apiofuranosyl-(1-6)]-β-D-glucopyranoside	0.114 ± 0.002	46531 ± 2227	1.303			
p-Vinylphenyl O-β-D-glucopyranoside	0.128 ± 0.001	41732 ± 2117	1.168			
Pyrantel pamoate	0.107 ± 0.001	32040 ± 2404	0.897			
Pyrantel tartrate	0.125 ± 0.004	38535 ± 6365	1.079			
Pyrazinamide	0.112 ± 0.004	45004 ± 1170	1.260			
Pyrazinecarboxamide	0.110 ± 0.006	32730 ± 939	0.916			
Pyrazinecarboxamide, 3,5-diamino-N-(aminoiminomethyl)-6-chloro	0.110 ± 0.001	71406 ± 11874	1.988			
Pyrethrins	0.098 ± 0.023	29964 ± 2834	0.839			
Pyridostigmine bromide	0.102 ± 0.002	30156 ± 2943	0.844			
Pyridostigmine iodid	0.114 ± 0.001	29918 ± 558	0.838			
Pyridoxal-5'-phosphate-6-(2'-naphthylazo-6'-nitro-4',8'-disulfonate)4Na	0.104 ± 0.005	22015 ± 2551	0.616			
Pyridoxalphosphate-6-azophenyl-2',4'-disulfonic acid 4Na	0.103 ± 0.007	20266 ± 1410	0.542			
Pyridoxine	0.101 ± 0.006	35773 ± 1037	1.002			
Pyridoxine hydrochloride	0.132 ± 0.012	37492 ± 708	1.050			
Pyrilamine maleate	0.102 ± 0.008	29039 ± 2353	0.813			
Pyrimethamine	0.113 ± 0.009	48089 ± 1862	1.346			
Pyrimidant	0.125 ± 0.005	39724 ± 1188	1.112			
Pyrimidinylpiperazine	0.114 ± 0.003	31830 ± 3658	0.891			

pyrithione zinc	0.061 ± 0.003 **					
Pyrithyldione	0.109 ± 0.002	52800 ± 2317	1.441			
Pyritinol	0.111 ± 0.019	28604 ± 2234	0.801			
Pyrocatechol	0.102 ± 0.005	41316 ± 4956	1.157			
Pyrocatechol monoglucoside	0.130 ± 0.001	30665 ± 1782	0.859			
Pyrocatechuic acid	0.100 ± 0.006	27872 ± 1276	0.780			
Pyrocincholic acid methyl ester	0.122 ± 0.004	43551 ± 2102	1.219			
Pyrogallin	0.067 ± 0.003 **					
Pyrogallol	0.107 ± 0.008	29970 ± 1761	0.839			
Pyronaridine tetraphosphate	0.107 ± 0.002	25941 ± 1558	0.726			
Pyrrolidinedithiocarbamate ammonium	0.104 ± 0.004	19392 ± 2261	0.537			
Pyrromycin	0.101 ± 0.011	30302 ± 1957	0.848			
Pyrvinium pamoate	0.111 ± 0.006	28804 ± 1221	0.806			
Qingyanshengenin	0.123 ± 0.005	35210 ± 1233	0.986			
Quassin	0.103 ± 0.004	29536 ± 2770	0.827			
Quazinone	0.113 ± 0.006	31965 ± 4361	0.895			
Quebrachitol	0.106 ± 0.003	54251 ± 6263	1.519			
Quercetagetin	0.086 ± 0.003 **					
Quercetin	0.119 ± 0.004	53826 ± 568	1.636			
Quercetin 3,4'-dimethyl ether	0.134 ± 0.007	49798 ± 6168	1.394			
Quercetin 3-O-robinobioside	0.116 ± 0.009	40611 ± 1967	1.137			
Quercetin 3-O-α-L-rhamnoside	0.115 ± 0.003	33237 ± 507	0.931			
quercetin 5,7,3',4'-tetramethyl ether	0.110 ± 0.007	30380 ± 2968	0.851			
Quercetin dihydrate	0.122 ± 0.003	29590 ± 5004	0.829			
Quercitrin	0.102 ± 0.004	24604 ± 266	0.689			
Quercitrin 3',4',2",3",4"-pentaacetate	0.114 ± 0.008	42447 ± 1524	1.188			
Quercitrin hexaacetate	0.137 ± 0.002	24966 ± 1276	0.699			
Quetiapine	0.110 ± 0.008	32652 ± 1806	0.914			
Quetiapine fumarate	0.110 ± 0.006	45098 ± 3478	1.263			
Quetiapine hemifumarate	0.107 ± 0.008	38696 ± 1795	1.083			
Quiditene	0.107 ± 0.016	27718 ± 1261	0.776			
Quinacrine dihydrochloride	0.087 ± 0.018	28160 ± 4437	0.788			

Quinacrine dihydrochloride dihydrate	0.120 ± 0.007	28768 ± 2393	0.805			
Quinacrine hydrochloride	0.115 ± 0.009	20134 ± 2444	0.580			
Quinacrine, Dihydrochloride	0.085 ± 0.004 **					
Quinalizarin	0.105 ± 0.003	50649 ± 3740	1.418			
Quinapril hydrochloride	0.120 ± 0.006	32769 ± 2430	0.918			
Quinaprilat	0.102 ± 0.001	46500 ± 2071	1.302			
Quinelorane dihydrochloride	0.104 ± 0.007	26339 ± 208	0.737			
Quinestrol	0.104 ± 0.004	41062 ± 2704	1.150			
Quinethazone	0.107 ± 0.014	33983 ± 2119	0.952			
Quinic acid	0.105 ± 0.001	33242 ± 2812	0.931			
Quinidine	0.111 ± 0.002	32743 ± 3487	0.917			
Quinidine gluconate	0.112 ± 0.016	26757 ± 3846	0.749			
Quinidine hydrochloride	0.103 ± 0.002	33160 ± 1858	0.928			
Quinidine sulfate	0.104 ± 0.005	25516 ± 1080	0.714			
Quinine	0.106 ± 0.002	33958 ± 1631	0.951			
Quinine ethyl carbonate	0.111 ± 0.005	28513 ± 2134	0.798			
Quinine hydrochloride	0.101 ± 0.002	32765 ± 1151	0.917			
Quinine sulfate	0.104 ± 0.004	25404 ± 3087	0.711			
Quinizarin	0.130 ± 0.011	20184 ± 4716	0.565			
Quinolinic acid	0.106 ± 0.002	42186 ± 1312	1.181			
Quinovic acid	0.118 ± 0.011	46304 ± 5633	1.296			
Quinovic acid 3-O-(3',4'-O-isopropylidene)-β-D-fucopyranoside	0.127 ± 0.002	31089 ± 6220	0.870			
Quinovic acid 3-O-(6-deoxy-β-D-glucopyranoside)28-O-β-D-glucopyranosyl ester	0.108 ± 0.011	37257 ± 4558	1.043			
Quinovic acid 3-O-.α.-L-rhamnopyranoside	0.133 ± 0.006	43339 ± 8678	1.213			
Quinovic acid 3-O-β-D-glucoside	0.106 ± 0.010	32062 ± 1363	0.898			
Quinovin	0.108 ± 0.014	25970 ± 1365	0.727			
Quinoxidine	0.112 ± 0.013	36471 ± 449	1.021			
Quipazine dimaleate	0.117 ± 0.010	56108 ± 3354	1.571			
Quipazine, 6-nitro-, maleate	0.107 ± 0.011	30565 ± 2514	0.856			
Quipazine, N-methyl-, dimaleate	0.105 ± 0.003	31345 ± 3002	0.878			
Quisqualic acid	0.103 ± 0.008	27724 ± 2721	0.776			

QX 222	0.112 ± 0.007	36050 ± 697	1.009			
QX 314 Bromide	0.107 ± 0.006	31195 ± 4904	0.873			
QX 314 Chloride	0.108 ± 0.005	29295 ± 736	0.820			
R 59-022	0.102 ± 0.002	34358 ± 5060	0.962			
R-(-)-2,10,11-Trihydroxyaporphine hybrobromide	0.094 ± 0.007	37376 ± 2660	1.047			
R-(-)-2,10,11-Trihydroxy-N-propyl-noraporphine hydrobromide	0.110 ± 0.014	41785 ± 1727	1.170			
R-(-)-2,11-Dihydroxy-10-methoxyaporphine hydrochloride	0.078 ± 0.009 **					
R-(-)-Apocodeine hydrochloride	0.100 ± 0.005	32244 ± 830	0.903			
R-(-)-Apomorphine	0.110 ± 0.002	31149 ± 1489	0.872			
R-(-)-apomorphine hydrochloride hemihydrate	0.106 ± 0.003	44958 ± 1694	1.259			
R-(-)-Denopamine	0.102 ± 0.001	26664 ± 4246	0.747			
R-(-)-Desmethyldeprenyl hydrochloride	0.100 ± 0.007	31870 ± 1386	0.892			
R-(-)-Isoproterenol (+)-bitartrate	0.097 ± 0.011	34314 ± 1831	0.961			
R-(-)-Me5	0.104 ± 0.010	33571 ± 1697	0.940			
R-(-)-N6-(2-Phenylisopropyl)-adenosine	0.101 ± 0.004	40168 ± 1924	1.125			
R-(-)-N-Allylnorapomorphine hydrobromide	0.090 ± 0.002 *					
R-(-)-Propylnorapomorphine hydrochloride	0.114 ± 0.013	37745 ± 1415	1.057			
R-(-)-SCH-12679 Maleate	0.109 ± 0.004	37697 ± 1520	1.055			
R-(+)-3PPP Hydrochloride	0.105 ± 0.007	41855 ± 5515	1.172			
R-(+)-6-Bromo-APB Hydrobromide	0.109 ± 0.004	45655 ± 1850	1.278			
R-(+)-7-Hydroxy-DPAT Hydrobromide	0.098 ± 0.005	45404 ± 3129	1.271			
R-(+)-8-Hydroxy-DPAT Hydrobromide	0.102 ± 0.004	38620 ± 225	1.081			
R-(+)-Atenolol	0.096 ± 0.007	34593 ± 3691	0.969			
R-(+)-Butylindazone	0.094 ± 0.003	39683 ± 4582	1.111			
R-(+)-Gomisin M1	0.123 ± 0.002	44694 ± 2784	1.251			
R-(+)-IAA-94	0.101 ± 0.003	21466 ± 2807	0.601			
R-(+)-Propranolol	0.122 ± 0.005	19357 ± 2967	0.542			
R-(+)-SCH-23390 Hydrochloride	0.101 ± 0.001	37136 ± 4076	1.040			
R-(+)-SKF-81297	0.115 ± 0.010	36761 ± 2160	1.029			
R-(+)-Terguride	0.107 ± 0.002	32331 ± 3212	0.905			
R-(+)-UH-301 Hydrochloride	0.105 ± 0.004	37591 ± 244	1.053			
R1487	0.126 ± 0.005	35608 ± 1840	0.997			

R-96544 Hydrochloride	0.114 ±0.010	24910 ±1221	0.697			
Rabeprazole	0.123 ±0.004	35745 ±3229	1.001			
Rabeprazole sodium	0.112 ±0.003	27253 ±2960	0.763			
rac-2-Ethoxy-3-hexadecanamido-1-propylphosphocholine	0.100 ±0.009	63418 ±4315	1.753			
rac-2-Ethoxy-3-octadecanamido-1-propylphosphocholine	0.109 ±0.008	35379 ±1522	0.991			
Racecadotril	0.119 ±0.001	20866 ±2293	0.584			
Racephedrine hydrochloride	0.104 ±0.012	30176 ±2651	0.845			
Raclopride	0.113 ±0.008	31700 ±4490	0.888			
Raclopride l-tartrate s(-)	0.114 ±0.006	33793 ±3175	0.946			
Ractopamine hydrochloride	0.101 ±0.003	30129 ±2937	0.844			
Radicicol	0.057 ±0.001 **					
Raloxifene hydrochloride	0.058 ±0.012 **					
Raltitrexed	0.127 ±0.004	64463 ±4883	1.795			
Ramelteon	0.116 ±0.024	37245 ±6582	1.043			
Ramifenazone	0.118 ±0.004	29859 ±1395	0.836			
Ramipril	0.111 ±0.007	24717 ±2998	0.692			
Ramoplanin	0.052 ±0.002 **					
Ranitidine hydrochloride	0.104 ±0.008	29525 ±2975	0.827			
Ranolazine	0.115 ±0.005	38550 ±2393	1.079			
Ranolazine dihydrochloride	0.114 ±0.005	25499 ±748	0.714			
Rapamycin	0.110 ±0.002	66939 ±7944	1.874			
Ras/Rac Transformation Blocker, SCH 51344	0.113 ±0.015	40318 ±753	1.129			
Rasagiline	0.099 ±0.003	35775 ±463	1.002			
Ras-Net (Elk-3)-pathway Inhibitor, XRP44X	0.097 ±0.004	52713 ±1139	1.542			
Raucaffricine	0.121 ±0.004	33912 ±2141	0.950			
Rauwolscine	0.100 ±0.004	28537 ±898	0.799			
Rauwolscine hydrochloride	0.103 ±0.011	52470 ±2728	1.450			
Reactive Blue 2	0.129 ±0.016	25407 ±2773	0.711			
Rebamipide	0.114 ±0.008	21528 ±3025	0.603			
Reboxetine Mesylate	0.112 ±0.003	31472 ±2574	0.881			
Rediocide A	0.110 ±0.003	36168 ±2294	1.013			
Rediocide C	0.114 ±0.003	46721 ±2684	1.308			

Rehmaglutin D	0.123 ± 0.004	46083 ± 1817	1.290			
Rehmannic acid	0.122 ± 0.004	43366 ± 730	1.214			
ReichsteinÂś substanceÂ S	0.126 ± 0.006	51454 ± 3556	1.441			
Relaxant	0.099 ± 0.014	43685 ± 2094	1.223			
Remacemide hydrochloride	0.121 ± 0.006	30748 ± 4856	0.861			
Remerine hydrochloride	0.094 ± 0.004 *					
Remoxipride	0.109 ± 0.013	32081 ± 2234	0.898			
Remoxipride Hydrochloride	0.122 ± 0.007	46551 ± 4560	1.303			
Reneilmol	0.122 ± 0.002	35565 ± 4007	0.996			
Rengynic acid	0.123 ± 0.012	39610 ± 1799	1.109			
Rengyol	0.129 ± 0.009	36588 ± 1052	1.024			
Repaglinide	0.115 ± 0.008	51797 ± 2580	1.450			
Reserpine	0.099 ± 0.002	30156 ± 578	0.844			
Resorcinol	0.123 ± 0.002	34723 ± 2556	0.972			
Resorcinol monoacetate	0.096 ± 0.005	19911 ± 1960	0.574			
Resveratrol	0.106 ± 0.015	97012 ± 6798	2.681	0.117 ± 0.005	107751 ± 5222	3.297
Resveratrol 4'-methyl ether	0.113 ± 0.003	39230 ± 1710	1.098			
Reticuline	0.119 ± 0.002	30489 ± 1676	0.854			
Retinoic acid	0.104 ± 0.012	36451 ± 1193	1.021			
Retinoic acid (all trans)	0.098 ± 0.001 *					
Retinoic acid p-hydroxyanilide	0.102 ± 0.005	37009 ± 3737	1.036			
Retinoic acid, 13-cis-	0.104 ± 0.003	26742 ± 473	0.749			
Retinoic acid, 9-cis-	0.100 ± 0.003	31877 ± 1177	0.893			
Retinoic acid, all trans	0.111 ± 0.005	28759 ± 911	0.805			
Retinol	0.093 ± 0.009	21443 ± 3211	0.600			
Retinyl acetate	0.122 ± 0.001	31223 ± 2040	0.874			
Retinyl palmitate	0.138 ± 0.012	40233 ± 8317	1.127			
Retrorsine	0.099 ± 0.003	27340 ± 315	0.766			
Retusin	0.111 ± 0.002	24672 ± 1720	0.691			
Retusin 7-methyl ether	0.108 ± 0.003	41821 ± 6688	1.171			
REV-2686	0.103 ± 0.008	43565 ± 3027	1.220			
REV-5901	0.108 ± 0.004	36329 ± 1743	1.017			

Reversine	0.122 ± 0.007	46306 ± 4828	1.297			
Reynosin	0.138 ± 0.005	26686 ± 1280	0.747			
RG-14620	0.124 ± 0.002	29804 ± 1765	0.834			
Rhamnetine	0.101 ± 0.018	18730 ± 1141	0.556			
Rhapontin	0.099 ± 0.003	31075 ± 165	0.870			
RHC 80267	0.103 ± 0.012	47629 ± 2071	1.334			
Rhein	0.109 ± 0.005	33317 ± 1053	0.933			
Rhetsinine	0.107 ± 0.003	28650 ± 2676	0.802			
Rhizocarpic acid	0.110 ± 0.008	42144 ± 2436	1.180			
Rho Kinase Inhibitor	0.112 ± 0.019	49606 ± 1820	1.389			
Rho Kinase Inhibitor II	0.136 ± 0.020	32101 ± 1310	0.899			
Rho Kinase Inhibitor III, Rockout	0.116 ± 0.007	49026 ± 3055	1.373			
Rho Kinase Inhibitor IV	0.138 ± 0.008	28893 ± 2156	0.809			
Rho Kinase Inhibitor V	0.123 ± 0.001	48299 ± 2927	1.352			
RHO-15	0.121 ± 0.005	36140 ± 1945	1.012			
Rhodinyl acetate	0.105 ± 0.006	37657 ± 3358	1.054			
Rhodocladonic acid	0.126 ± 0.010	36778 ± 3305	1.030			
Rhododendrol	0.121 ± 0.004	51715 ± 3268	1.448			
Rhoifolin	0.110 ± 0.031	41757 ± 2050	1.169			
Rhuscholide A	0.124 ± 0.004	38777 ± 1500	1.086			
Ribavirin	0.080 ± 0.011 **					
Riboflavin	0.119 ± 0.003	27890 ± 3706	0.781			
Riboflavin 5-phosphate sodium	0.105 ± 0.003	26209 ± 5840	0.734			
Ribostamycin sulfate	0.106 ± 0.001	37678 ± 2068	1.055			
Ribostamycin sulfate salt	0.113 ± 0.019	66863 ± 6550	1.825			
Richenoic acid	0.128 ± 0.005	37975 ± 1720	1.063			
Ricinine	0.110 ± 0.006	43585 ± 2853	1.220			
Ricinoleic acid	0.117 ± 0.005	29969 ± 1404	0.839			
Ricobendazole	0.111 ± 0.002	48859 ± 1916	1.368			
Rifabutin	0.106 ± 0.002	40029 ± 2584	1.121			
Rifampicin	0.105 ± 0.006	39419 ± 4502	1.104			
Rifampin	0.098 ± 0.007	33063 ± 1969	0.926			

Rifamycin sv	0.099 ± 0.007	32509 ± 331	0.910			
Rifamycin SV-NA	0.105 ± 0.006	21142 ± 395	0.628			
Rifapentine	0.106 ± 0.004	26390 ± 5739	0.739			
Rifaximin	0.106 ± 0.003	19320 ± 1225	0.538			
Rilmenidine hemifumarate	0.108 ± 0.002	34600 ± 3504	0.969			
Riluzole	0.111 ± 0.003	49679 ± 3541	1.391			
Riluzole hydrochloride	0.128 ± 0.004	52710 ± 3483	1.476			
Rimantadine hydrochloride	0.117 ± 0.002	25873 ± 232	0.724			
Rimcazole	0.112 ± 0.004	12653 ± 895	0.352	0.109 ± 0.002	37872 ± 4399	1.159
Rimexolone	0.112 ± 0.009	43649 ± 1075	1.222			
Rimonabant Hydrochloride	0.113 ± 0.007	55346 ± 3803	1.630			
Risedronate sodium	0.103 ± 0.004	37798 ± 3789	1.058			
Risedronic acid	0.109 ± 0.008	35506 ± 2274	0.994			
Risperidone	0.110 ± 0.010	33395 ± 2505	0.935			
Ritanserin	0.104 ± 0.007	47673 ± 4939	1.335			
Ritodrine hydrochloride	0.106 ± 0.005	39503 ± 2920	1.106			
Ritonavir	0.108 ± 0.002	40000 ± 5974	1.120			
Rivastigmine	0.116 ± 0.002	39180 ± 3841	1.097			
Rivastigmine tartrate	0.117 ± 0.007	28662 ± 3991	0.803			
Rivulobirin B	0.136 ± 0.003	32465 ± 1843	0.909			
Rivulobirin E	0.122 ± 0.004	34828 ± 3044	0.975			
Rizatriptan benzoate	0.131 ± 0.003 *					
RJR 2403 fumarate	0.101 ± 0.008	31426 ± 3770	0.880			
RK-682	0.129 ± 0.002	28583 ± 2216	0.800			
RN 1734	0.113 ± 0.005	32943 ± 2189	0.922			
RN 1747	0.118 ± 0.006	43576 ± 6590	1.220			
RNA Polymerase III Inhibitor	0.106 ± 0.007	36148 ± 2915	1.012			
Ro 04-5595	0.103 ± 0.003	36641 ± 5434	1.026			
RO 04-6790 dihydrochloride	0.103 ± 0.002	39518 ± 4814	1.106			
Ro 08-2750	0.109 ± 0.007	38169 ± 1669	1.069			
Ro 10-5824	0.108 ± 0.005	34933 ± 4279	0.978			
Ro 106-9920	0.062 ± 0.006 **					

Ro 15-4513	0.102 ±0.018	26664 ±3200	0.747			
Ro 19-4605	0.110 ±0.002	27174 ±916	0.761			
Ro 20-1724	0.104 ±0.002	51861 ±3287	1.452			
Ro 25-6981 hydrochloride	0.107 ±0.007	32056 ±4164	0.898			
Ro 25-6981 maleate	0.113 ±0.007	37486 ±1377	1.050			
Ro 31-8220	0.096 ±0.004 *					
Ro 31-8220 mesylate	0.068 ±0.003 **					
Ro 41-0960	0.106 ±0.006	46495 ±3937	1.302			
Ro 41-1049 hydrochloride	0.104 ±0.000	23429 ±2407	0.656			
Ro 60-0175 fumarate	0.106 ±0.006	25514 ±2001	0.714			
Ro 8-4304	0.098 ±0.008	59871 ±2045	1.655			
Ro 90-7501	0.104 ±0.009	7623 ±2143	0.211	0.115 ±0.003	20724 ±3180	0.634
Ro-31-8220	0.071 ±0.017 **					
Robinetin	0.111 ±0.004	37830 ±2568	1.059			
Robinetine	0.106 ±0.005	40510 ±1410	1.134			
Robinin	0.119 ±0.005	42039 ±5476	1.177			
Robtin	0.121 ±0.006	36596 ±2977	1.025			
Robustic acid	0.120 ±0.007	36492 ±403	1.022			
Rocaglamide	0.129 ±0.014	43913 ±4302	1.230			
Rocaglaol	0.135 ±0.018	34180 ±3737	0.957			
Roccellic acid	0.109 ±0.006	44320 ±3548	1.241			
ROCK Inhibitor, Y-27632	0.110 ±0.003	31802 ±2260	0.890			
Rocuronium bromide	0.109 ±0.001	48034 ±3449	1.345			
Rofecoxib	0.118 ±0.014	53507 ±6755	1.498			
Rolipram	0.107 ±0.004	48993 ±2217	1.372			
Rolitetracycline	0.116 ±0.004	19540 ±294	0.544			
Ronidazole	0.112 ±0.007	32590 ±4424	0.913			
Ropinirole hydrochloride	0.103 ±0.006	42575 ±2316	1.192			
Ropivacaine hydrochloride	0.117 ±0.003	59493 ±6328	1.666			
Roscovitine	0.126 ±0.004	37580 ±1530	1.052			
Roscovitine, (S)-Isomer	0.116 ±0.017	43277 ±1396	1.212			
Rosenonolactone	0.114 ±0.003	31862 ±2122	0.892			

Roseoside	0.123 ± 0.002	56235 ± 2498	1.575			
Rosiglitazone hydrochloride	0.122 ± 0.002	60688 ± 3592	1.690			
Rosiglitazone maleate	0.110 ± 0.004	85734 ± 3093	2.387	0.113 ± 0.007	41726 ± 4370	1.277
Rosmarinic acid	0.099 ± 0.003 *					
Rosolic acid	0.100 ± 0.005	23167 ± 1833	0.649			
Rosthornin A	0.113 ± 0.000	13351 ± 1458	0.373	0.115 ± 0.007	38075 ± 3517	1.165
Rosthornin B	0.122 ± 0.001	18481 ± 987	0.517			
Rosuvastatin Calcium	0.108 ± 0.004	33536 ± 849	0.939			
Rotenone	0.107 ± 0.009	35711 ± 4610	1.000			
Rottlerin	0.124 ± 0.005	26695 ± 632	0.747			
Roxarsone	0.116 ± 0.007	32505 ± 775	0.910			
Roxatidine acetate hydrochloride	0.103 ± 0.003	37273 ± 4754	1.044			
Roxithromycin	0.076 ± 0.004 **					
RS 100329 hydrochloride	0.112 ± 0.003	40094 ± 1957	1.123			
RS 102221 hydrochloride	0.111 ± 0.005	33956 ± 300	0.951			
RS 102895 hydrochloride	0.120 ± 0.002	32036 ± 4663	0.897			
RS 16566 dihydrochloride	0.116 ± 0.011	34539 ± 4342	0.967			
RS 17053 hydrochloride	0.123 ± 0.006	6131 ± 635	0.164	0.122 ± 0.009	29726 ± 3348	0.910
RS 23597-190 hydrochloride	0.109 ± 0.017	31580 ± 1051	0.884			
RS 39604 hydrochloride	0.104 ± 0.004	31364 ± 4792	0.878			
RS 45041-190 hydrochloride	0.109 ± 0.005	56117 ± 4951	1.553			
RS 504393	0.108 ± 0.001	31683 ± 843	0.887			
RS 56812 hydrochloride	0.099 ± 0.009	31176 ± 2060	0.873			
RS 67333 hydrochloride	0.123 ± 0.003	43857 ± 3059	1.228			
RS 67506 hydrochloride	0.110 ± 0.007	33040 ± 4067	0.925			
RS 79948 hydrochloride	0.112 ± 0.007	32986 ± 4387	0.924			
RSK Inhibitor, SL0101	0.113 ± 0.014	34329 ± 897	0.961			
RU 24969	0.126 ± 0.004	43737 ± 1274	1.225			
RU 24969 hemisuccinate	0.102 ± 0.007	59330 ± 7956	1.661			
RU 28318 potassium salt	0.106 ± 0.004	31939 ± 2720	0.894			
Rubelloside B	0.125 ± 0.003	37152 ± 2418	1.040			
Rubescensin A	0.081 ± 0.012 **					

Rubiadin	0.115 ± 0.003	44064 ± 2471	1.234			
Rubiadin 1-methyl ether	0.121 ± 0.005	44072 ± 5599	1.234			
Rubianthraquinone	0.120 ± 0.016	25534 ± 659	0.715			
Rubiarbonol B	0.121 ± 0.007	31838 ± 4610	0.891			
Rubifolic acid	0.127 ± 0.008	39843 ± 3705	1.116			
Rubranol	0.115 ± 0.002	40596 ± 2561	1.137			
Rufinamide	0.109 ± 0.006	35031 ± 2711	0.981			
Rufloxacin	0.066 ± 0.003 **					
Rufloxacin hydrochloride	0.067 ± 0.005 **					
Rutaecarpine	0.107 ± 0.017	40030 ± 3150	1.121			
Ruthenium red	0.126 ± 0.013	24405 ± 1962	0.683			
Rutilantinone	0.111 ± 0.003	44128 ± 7086	1.236			
Rutin	0.123 ± 0.001	40254 ± 1263	1.127			
RWJ 21757	0.097 ± 0.003	37941 ± 1755	1.062			
RWJ-60475	0.119 ± 0.004	51284 ± 1664	1.674			
RWJ-60475-(AM)3	0.104 ± 0.016	32427 ± 5803	0.908			
RWJ-67657	0.123 ± 0.002	28130 ± 8109	0.788			
RX 821002 Hydrochloride	0.098 ± 0.006	51877 ± 2153	1.434			
Ryanodine	0.107 ± 0.003	38372 ± 338	1.074			
Ryuvidine	0.114 ± 0.002	35786 ± 664	1.002			
S 14506	0.088 ± 0.005 **					
S-(-)-3PPP hydrochloride	0.108 ± 0.007	49584 ± 1121	1.371			
S-(-)-Atenolol	0.103 ± 0.003	39645 ± 1318	1.110			
S-(-)-Carbidopa	0.103 ± 0.016	35055 ± 1121	0.982			
S-(-)-Eticlopride hydrochloride	0.106 ± 0.001	28408 ± 500	0.795			
S-(-)-Lisuride	0.114 ± 0.009	28305 ± 1599	0.793			
S-(-)-p-Bromotetramisole oxalate	0.099 ± 0.001	38017 ± 270	1.064			
S-(-)-Pindolol	0.101 ± 0.010	37787 ± 2949	1.058			
S-(-)-Propranolol hydrochloride	0.115 ± 0.014	21881 ± 2614	0.613			
S-(-)-Raclopride L-tartrate	0.110 ± 0.006	32000 ± 1831	0.896			
S-(-)-Sulpiride	0.101 ± 0.005	28333 ± 3662	0.793			
S-(-)-Timolol maleate	0.118 ± 0.012	28764 ± 1852	0.805			

S-(-)-UH-301 Hydrochloride	0.119 ± 0.003	37720 ± 3912	1.056			
S-(-)-Willardiine	0.110 ± 0.008	34440 ± 3224	0.964			
S-(+)-Fluoxetine hydrochloride	0.106 ± 0.005	20764 ± 1364	0.574			
S-(+)-Ibuprofen	0.092 ± 0.002 *					
S-(+)-Isoproterenol (+)-bitartrate	0.102 ± 0.003	29730 ± 2498	0.832			
S-(+)-PD 123177 trifluoroacetate salt hydrate	0.105 ± 0.008	40789 ± 1410	1.142			
S-(+)-PD 128,907 hydrochloride	0.108 ± 0.014	70473 ± 10865	1.948			
S-(+)-Raclopride L-tartrate	0.107 ± 0.012	63434 ± 4032	1.753			
S-(+)-Terguride	0.111 ± 0.009	23842 ± 252	0.668			
S-(4-Nitrobenzyl)-6-thioguanosine	0.106 ± 0.001	62713 ± 4290	1.733			
S-(4-Nitrobenzyl)-6-thioinosine	0.101 ± 0.015	32005 ± 604	0.896			
S-(p-Azidophenacyl)-glutathione	0.098 ± 0.002	36854 ± 1880	1.032			
S0693	0.100 ± 0.009	120534 ± 7156	3.332	0.121 ± 0.009	113552 ± 6048	3.475
S15535	0.103 ± 0.008	34118 ± 462	0.955			
S1P1 Receptor Agonist, SEW2871	0.102 ± 0.002	33777 ± 636	0.946			
S26948	0.109 ± 0.007	36556 ± 1193	1.024			
Saccharin	0.101 ± 0.008	39508 ± 9496	1.106			
Saclofen	0.109 ± 0.005	28845 ± 1749	0.808			
Saclofen hydrochloride	0.107 ± 0.004	26167 ± 991	0.733			
S-Adenosyl-L-homocysteine	0.101 ± 0.006	41786 ± 2245	1.170			
Safrole	0.107 ± 0.004	34887 ± 4070	0.977			
Safrolglycol	0.127 ± 0.006	37361 ± 3117	1.046			
Saha	0.110 ± 0.008	33353 ± 211	0.934			
Saikosaponin B2	0.125 ± 0.003	41619 ± 1794	1.165			
Sakakin	0.116 ± 0.003	38136 ± 1332	1.068			
Sakuranetin	0.106 ± 0.003	24273 ± 2421	0.680			
Sal 003	0.113 ± 0.006	25604 ± 1744	0.717			
Salbutamol	0.098 ± 0.007	28256 ± 6246	0.791			
Salbutamol sulfate	0.123 ± 0.004	60531 ± 6712	1.695			
Salermide	0.106 ± 0.014	29435 ± 775	0.824			
Salicin	0.117 ± 0.005	37480 ± 3165	1.049			
Salicyl alcohol	0.123 ± 0.004	34345 ± 3598	0.962			

Salicylamide	0.122 ± 0.005	37347 ± 5430	1.046			
Salicylanilide	0.103 ± 0.006	17888 ± 1626	0.515			
Salicylidene salicylhydrazide	0.104 ± 0.012	63842 ± 6693	1.880			
Salidroside	0.111 ± 0.002	23847 ± 883	0.668			
Salinazid	0.117 ± 0.008	50989 ± 9881	1.428			
Salinomycin	0.092 ± 0.001 **					
Salinomycin, sodium	0.098 ± 0.002	52994 ± 2365	1.484			
Salirepin	0.119 ± 0.001	39118 ± 3521	1.095			
Salmeterol	0.126 ± 0.003	28577 ± 3949	0.800			
Salmeterol xinafoate	0.097 ± 0.008	70472 ± 10538	1.948			
Salsalate	0.093 ± 0.003 *					
Salsolidine	0.126 ± 0.001	35770 ± 2109	1.002			
Salsoline	0.122 ± 0.010	27977 ± 327	0.783			
Salsolinol hydrobromide	0.102 ± 0.001	36463 ± 1582	1.021			
Salsolinol-1-carboxylic acid	0.115 ± 0.007	31109 ± 677	0.871			
Salubrinal	0.107 ± 0.005	37772 ± 5604	1.058			
Salvianolic acid B	0.125 ± 0.019	37984 ± 743	1.064			
Salvigenin	0.123 ± 0.010	43038 ± 4497	1.205			
Salvinorin A	0.106 ± 0.002	34976 ± 3361	0.979			
Sambutoxin	0.126 ± 0.006	26013 ± 1449	0.728			
Sams peptide	0.107 ± 0.012	21211 ± 1519	0.594			
Sandoz 58-035	0.101 ± 0.001	36209 ± 3809	1.014			
Sanguinarine	0.106 ± 0.005	36533 ± 2481	1.023			
Sanguinarine chloride	0.066 ± 0.010 **					
Sanguinarine sulfate	0.075 ± 0.007 **					
SANT-1	0.101 ± 0.005	32999 ± 805	0.924			
SANT-2	0.105 ± 0.004	33462 ± 1821	0.937			
Santamarine	0.108 ± 0.009	30756 ± 349	0.861			
Santonin	0.126 ± 0.003	60847 ± 4181	1.807			
Saponarin	0.111 ± 0.005	20340 ± 1705	0.604			
Sappanchalcone	0.129 ± 0.023	23728 ± 1808	0.664			
sappanone a dimethyl ether	0.104 ± 0.004	34877 ± 1719	0.977			

Saquinavir mesylate	0.111 ± 0.005	34121 ± 2616	0.955			
Sarafloxacin	0.069 ± 0.012 **					
Sarafloxacin hydrochloride	0.068 ± 0.002 **					
Sarcandrone A	0.122 ± 0.008	45955 ± 5451	1.287			
Sarcandrone B	0.131 ± 0.005	33387 ± 3387	0.935			
Sarmentosin	0.108 ± 0.010	40328 ± 2160	1.129			
Sarpagine	0.121 ± 0.014	49147 ± 6759	1.376			
Sarpogrelate Hydrochloride	0.105 ± 0.008	40122 ± 1277	1.123			
Sarsasapogenin	0.104 ± 0.004	23178 ± 335	0.649			
Savinin	0.118 ± 0.009	41541 ± 3283	1.163			
Saxagliptin	0.110 ± 0.002	26625 ± 1337	0.745			
SB 200646 hydrochloride	0.108 ± 0.003	48775 ± 1762	1.366			
SB 202190	0.134 ± 0.008	31070 ± 798	0.870			
SB 202474	0.105 ± 0.004	24293 ± 1586	0.680			
SB 203186 hydrochloride	0.111 ± 0.002	34194 ± 4811	0.957			
SB 203580	0.109 ± 0.002	22445 ± 3840	0.628			
SB 203580, Sulfone	0.126 ± 0.013	28957 ± 1463	0.811			
SB 205384	0.103 ± 0.003	32187 ± 8258	0.901			
SB 205607	0.097 ± 0.002 *					
SB 206553 hydrochloride	0.114 ± 0.000	36483 ± 4280	1.022			
SB 215505	0.101 ± 0.006	33546 ± 1555	0.939			
SB 216641 hydrochloride	0.098 ± 0.009	21760 ± 854	0.602			
SB 216763	0.105 ± 0.010	35137 ± 2513	0.984			
SB 218078	0.113 ± 0.006	25496 ± 2378	0.714			
SB 218795	0.103 ± 0.003	32299 ± 1779	0.904			
SB 220025	0.109 ± 0.010	25863 ± 1588	0.724			
SB 221284	0.111 ± 0.004	29904 ± 2312	0.837			
SB 222200	0.101 ± 0.005	39174 ± 1425	1.097			
SB 224289 hydrochloride	0.141 ± 0.005 **					
SB 225002	0.100 ± 0.001	67735 ± 10265	1.982			
SB 228357	0.116 ± 0.002	35370 ± 2593	0.990			
SB 239063	0.112 ± 0.018	43670 ± 2702	1.223			

SB 242084 dihydrochloride	0.076 ± 0.011 **					
SB 242235	0.121 ± 0.006	41326 ± 3239	1.157			
SB 258585 hydrochloride	0.111 ± 0.007	37229 ± 863	1.042			
SB 269970 hydrochloride	0.102 ± 0.008	22823 ± 5725	0.639			
SB 328437	0.109 ± 0.001	33668 ± 2010	0.943			
SB 334867	0.099 ± 0.005	26899 ± 1012	0.753			
SB 366791	0.116 ± 0.005	41297 ± 2593	1.156			
SB 408124	0.111 ± 0.011	19301 ± 2622	0.534			
SB 415286	0.109 ± 0.002	60361 ± 6128	1.834			
SB 431542	0.108 ± 0.010	46653 ± 4171	1.306			
SB 525334	0.106 ± 0.003	25485 ± 3359	0.714			
SB 590885	0.127 ± 0.006	27768 ± 1560	0.777			
SB 657510	0.108 ± 0.006	31872 ± 4230	0.892			
SC 12757	0.116 ± 0.010	29938 ± 4534	0.838			
SC 19220	0.104 ± 0.006	28967 ± 4926	0.811			
SC 514	0.112 ± 0.005	38022 ± 6328	1.065			
SC 560	0.105 ± 0.012	46755 ± 3475	1.309			
SC 68376	0.115 ± 0.012	25463 ± 1486	0.713			
SC 9	0.114 ± 0.005	33313 ± 1516	0.933			
SC-10	0.114 ± 0.020	30847 ± 1369	0.864			
Scabertopin	0.127 ± 0.001	27538 ± 2344	0.771			
Scandine	0.118 ± 0.006	36071 ± 2244	1.010			
SCH 23390	0.111 ± 0.008	30063 ± 1524	0.842			
SCH 28080	0.113 ± 0.007	32728 ± 2327	0.916			
SCH 50911	0.107 ± 0.008	26804 ± 961	0.750			
SCH 58261	0.113 ± 0.001	49340 ± 5762	1.381			
SCH-202676	0.099 ± 0.009	43426 ± 2687	1.216			
Schisandrin A, R(+)-	0.103 ± 0.001	41726 ± 794	1.168			
Schisandrin B, S(-)-	0.104 ± 0.006	36125 ± 718	1.011			
Schisantherin A	0.124 ± 0.007	30813 ± 2001	0.863			
Schizandrin	0.117 ± 0.007	33881 ± 1171	0.949			
Schleicheol 1	0.124 ± 0.001	33955 ± 369	0.951			

Schleicheol 2	0.131 ±0.002	28260 ±6432	0.791			
Scholaricine	0.123 ±0.003	36037 ±1605	1.009			
Sciadopitysin	0.130 ±0.002	44559 ±615	1.248			
Scillascillin	0.112 ±0.004	47985 ±4786	1.344			
Sclareol	0.088 ±0.004 **					
Sclareolide	0.120 ±0.002	35114 ±2204	0.983			
Sclareolide, (3aR)-(+)-	0.101 ±0.003	26537 ±180	0.743			
Sclerotiorin	0.099 ±0.001 *					
Scoparinol	0.116 ±0.015	32693 ±2798	0.915			
Scoparone	0.112 ±0.005	28759 ±1769	0.805			
Scopolamine hydrobromide	0.114 ±0.002	34017 ±1631	0.952			
Scopolamine hydrochloride	0.104 ±0.011	43149 ±4554	1.208			
Scopolamine n-butylbromide	0.114 ±0.002	26380 ±2225	0.739			
Scopolamin-N-oxide hydrobromide	0.120 ±0.002	38729 ±2672	1.084			
Scopoletin	0.117 ±0.004	62146 ±2204	1.846			
Scopoletin acetate	0.123 ±0.001	31497 ±1687	0.882			
Scopolin	0.112 ±0.011	34790 ±1319	0.974			
Scopolomine N-butylbromide	0.107 ±0.013	21145 ±932	0.628			
Scoulerine	0.090 ±0.004 **					
Scriptaid	0.102 ±0.005	19081 ±714	0.592			
SCS	0.114 ±0.003	32949 ±3620	0.923			
Sculponeatic acid	0.120 ±0.003	40423 ±3626	1.132			
Sculponeatin A	0.121 ±0.002	26947 ±1911	0.755			
Sculponeatin K	0.124 ±0.003	37017 ±443	1.036			
Sculponeatin N	0.125 ±0.011	41280 ±1093	1.156			
Sculponeatin O	0.127 ±0.002	52623 ±2826	1.473			
Scutebarbatine A	0.131 ±0.002	21588 ±315	0.603			
Scutebarbatine B	0.133 ±0.008	34006 ±3035	0.952			
Scutebata A	0.127 ±0.012	34413 ±5633	0.964			
Scutebata B	0.119 ±0.001	37825 ±1413	1.059			
Scutebata C	0.136 ±0.006	37398 ±3044	1.047			
Scutebata E	0.126 ±0.003	29810 ±2306	0.835			

Scutebata F	0.126 ± 0.002	33891 ± 4275	0.949			
Scutebata G	0.124 ± 0.008	34824 ± 1247	0.975			
Scutellaric acid	0.115 ± 0.005	31595 ± 1804	0.885			
Scytonemin, Lyngbya sp.	0.137 ± 0.018	24433 ± 4255	0.684			
SD 169	0.105 ± 0.005	48532 ± 1745	1.359			
SD 208	0.109 ± 0.009	22227 ± 3665	0.622			
SD 06	0.122 ± 0.001	38380 ± 2943	1.075			
SDM 25N	0.117 ± 0.003	25091 ± 4244	0.703			
SDZ 205557 hydrochloride	0.106 ± 0.005	39183 ± 3623	1.097			
SDZ 21009	0.115 ± 0.003	36469 ± 4570	1.021			
SDZ 220040	0.106 ± 0.004	39963 ± 1964	1.119			
SDZ 220581	0.114 ± 0.005	31687 ± 6094	0.887			
SDZ NKT 343	0.106 ± 0.009	34096 ± 757	0.955			
SDZ SER 082 fumarate	0.107 ± 0.006	35109 ± 1231	0.983			
SDZ WAG 994	0.105 ± 0.010	25995 ± 1337	0.728			
SDZ-201106	0.105 ± 0.002	55420 ± 4212	1.684			
SDZ-205,557 hydrochloride	0.106 ± 0.008	30198 ± 1590	0.846			
Se-(methyl)-selenocysteine hydrochloride	0.093 ± 0.008	60331 ± 1486	1.668			
Secaubryenol	0.118 ± 0.002	29635 ± 1894	0.830			
Secaubrytriol	0.120 ± 0.002	35228 ± 3689	0.986			
SecinH3	0.098 ± 0.004	37689 ± 1390	1.055			
Secnidazole	0.121 ± 0.004	26498 ± 1282	0.742			
Secoisolariciresinol	0.121 ± 0.005	55495 ± 7063	1.554			
Secologanin dimethyl acetal	0.130 ± 0.001	36508 ± 811	1.022			
Secoxyloganin	0.127 ± 0.002	31372 ± 2687	0.878			
Secoxyloganin methyl ester	0.117 ± 0.003	34040 ± 2761	0.953			
Securinine	0.123 ± 0.006	33700 ± 951	0.944			
Sedanolide	0.122 ± 0.003	38625 ± 845	1.081			
Selamectin	0.105 ± 0.008	31914 ± 1877	0.894			
Selegiline hydrochloride	0.123 ± 0.004	45163 ± 1949	1.265			
Selenomethionine	0.088 ± 0.005 **					
Sematilide monohydrochloride monohydrate	0.102 ± 0.007	36721 ± 356	1.028			

Semialactone	0.123 ±0.006	48169 ±4593	1.349			
Semicarbazide hydrochloride	0.094 ±0.005	29823 ±4298	0.835			
Sempervirine	0.134 ±0.009	39923 ±8031	1.118			
Semustine	0.111 ±0.004	26000 ±2237	0.728			
Sendanolactone	0.119 ±0.010	36937 ±3977	1.034			
Senecionine	0.112 ±0.006	21642 ±852	0.643			
Seneciphylline	0.095 ±0.009	20959 ±326	0.622			
Seneciphylline N-oxide	0.119 ±0.008	32416 ±2704	0.908			
Seneciphyllinine	0.118 ±0.008	30991 ±5718	0.868			
Seneganolide	0.125 ±0.003	30796 ±2220	0.862			
Sennoside a	0.113 ±0.007	30748 ±3283	0.861			
Sennoside b	0.113 ±0.006	31832 ±3498	0.891			
Sepiapterin	0.106 ±0.007	30926 ±1090	0.866			
Sepinol	0.119 ±0.006	51062 ±4834	1.430			
Seratrodast	0.106 ±0.006	37023 ±7519	1.037			
Serotonin	0.107 ±0.003	30378 ±2582	0.851			
Serotonin hydrochloride	0.102 ±0.011	35825 ±4904	1.003			
Serpentine	0.128 ±0.009	31882 ±1644	0.893			
Serpentine hydrochloride	0.123 ±0.004	33753 ±1668	0.945			
Serpentinic acid	0.132 ±0.000	43576 ±2254	1.220			
Serpentinine	0.119 ±0.011	27293 ±1480	0.764			
Serratenediol	0.127 ±0.005	40161 ±579	1.124			
Serratenediol diacetate	0.131 ±0.005	40603 ±440	1.137			
Serratriol	0.111 ±0.020	42794 ±2690	1.198			
Sertaconazole	0.066 ±0.002 **					
Sertaconazole nitrate	0.109 ±0.013	36776 ±891	1.030			
Sertindole	0.115 ±0.005	24262 ±1531	0.679			
Sertraline	0.123 ±0.003	13571 ±1421	0.378	0.113 ±0.008	27632 ±3161	0.846
Sertraline hydrochloride	0.109 ±0.016	23292 ±1208	0.644			
Sesamin	0.137 ±0.008	38570 ±3632	1.080			
Sesamoside	0.125 ±0.006	42463 ±4316	1.189			
Sessilifoline A	0.115 ±0.007	36534 ±1002	1.023			

S-Ethylisothiourea hydrobromide	0.107 ± 0.004	37221 ± 2358	1.042			
Sevedindione	0.102 ± 0.005	24206 ± 83	0.678			
Sevoflurane	0.109 ± 0.006	35803 ± 1790	1.002			
S-Farnesyl-L-cysteine ME	0.105 ± 0.003	33012 ± 2546	0.924			
S-Farnesyl-L-cysteine methyl ester	0.123 ± 0.002	53234 ± 6158	1.491			
sFRP-1 Inhibitor	0.098 ± 0.006	31599 ± 2458	0.885			
SG-209	0.114 ± 0.004	49114 ± 1648	1.375			
Shanzhiside	0.115 ± 0.001	38301 ± 780	1.072			
Shanzhiside methyl ester	0.123 ± 0.011	38891 ± 2928	1.089			
Shikimic Acid	0.102 ± 0.003	25368 ± 549	0.710			
Shikonin	0.107 ± 0.014	24621 ± 1328	0.689			
Shizukanolide H	0.121 ± 0.002	29281 ± 1221	0.820			
Shizukaol C	0.111 ± 0.006	46973 ± 1012	1.315			
Shizukaol D	0.127 ± 0.003	37348 ± 1506	1.046			
Shizukolidol	0.118 ± 0.004	38097 ± 2276	1.067			
Shoreic acid	0.118 ± 0.006	29716 ± 898	0.832			
SHP1/2 PTPase Inhibitor, NSC-87877	0.100 ± 0.003	44790 ± 3257	1.254			
Shyobunone	0.117 ± 0.001	40903 ± 2260	1.145			
SIB 1757	0.112 ± 0.006	64772 ± 5890	1.790			
SIB 1893	0.113 ± 0.005	35575 ± 574	0.996			
Sibiricin	0.116 ± 0.009	47601 ± 1033	1.333			
Sibutramine	0.132 ± 0.004 *					
Sibutramine hydrochloride	0.108 ± 0.007	32613 ± 2017	0.913			
SID 7969543	0.109 ± 0.012	36022 ± 1827	1.009			
Sideroxylonal A	0.109 ± 0.008	33768 ± 361	0.945			
Siguazodan	0.106 ± 0.001	26778 ± 3033	0.750			
Sildenafil	0.120 ± 0.001	48333 ± 5623	1.353			
Sildenafil Citrate	0.107 ± 0.006	48684 ± 3806	1.363			
Silibinin	0.096 ± 0.011	37738 ± 2058	1.057			
Silybine	0.123 ± 0.003	42043 ± 1667	1.177			
Silymarin	0.123 ± 0.001	31528 ± 2493	0.883			
Simiarenol	0.129 ± 0.004	33731 ± 1046	0.944			

Simiarenol acetate	0.116 ± 0.003	45690 ± 2109	1.279			
Simiarenol methylthiomethyl ether	0.124 ± 0.002	29447 ± 2073	0.824			
Simiarenone	0.119 ± 0.003	38598 ± 1222	1.081			
Simonsinol	0.116 ± 0.003	38839 ± 4045	1.087			
Simplidin butyl ether	0.112 ± 0.003	41966 ± 422	1.175			
Simvastatin	0.098 ± 0.001	35072 ± 2814	0.982			
Simvastatin, Sodium Salt	0.092 ± 0.004	35763 ± 1451	1.001			
Sinapaldehyde	0.129 ± 0.005	42073 ± 390	1.178			
Sinapaldehyde glucoside	0.127 ± 0.020	36449 ± 6118	1.021			
Sinapic acid	0.096 ± 0.004	85612 ± 3837	2.466	0.097 ± 0.006	60697 ± 4928	1.861
Sinapic acid methyl ether	0.120 ± 0.010	36695 ± 2891	1.027			
Sinensetin	0.107 ± 0.003	42563 ± 1608	1.192			
Sinensetine	0.113 ± 0.005	25582 ± 430	0.716			
Sinoacutine	0.119 ± 0.003	45330 ± 4826	1.269			
Sinomenine	0.123 ± 0.003	35146 ± 2089	0.984			
Sirolimus	0.104 ± 0.004	19208 ± 1692	0.553			
SIRT1 Inhibitor III	0.115 ± 0.008	153196 ± 5522	4.482	0.116 ± 0.001	41488 ± 865	1.270
SIRT1 Inhibitor IV, (S)-35	0.116 ± 0.015	40153 ± 1847	1.124			
SIRT1/2 Inhibitor IV, Cambinol	0.109 ± 0.004	39044 ± 5510	1.093			
SIRT1/2 Inhibitor VIII, Salermide	0.103 ± 0.006	37020 ± 337	1.037			
SIRT2 Inhibitor, AGK2	0.105 ± 0.009	31748 ± 2163	0.889			
Sirtinol	0.117 ± 0.009	34629 ± 2716	0.970			
S-isocorydine (+)	0.126 ± 0.009	31171 ± 2933	0.873			
Sisomicin sulfate	0.117 ± 0.001	69319 ± 1664	1.892			
S-Isopropylisothiourea hydrobromide	0.116 ± 0.004	34971 ± 5591	0.979			
Sissotrin	0.118 ± 0.004	32748 ± 2296	0.917			
Sitagliptin monophosphate monohydrate	0.101 ± 0.012	28201 ± 4138	0.790			
Sitoindoside I	0.117 ± 0.002	55880 ± 7638	1.565			
Sitostenone	0.112 ± 0.007	43241 ± 4198	1.211			
Sitosterol, b -	0.109 ± 0.004	35477 ± 1047	0.993			
Sitosteryl acetate	0.107 ± 0.002	41121 ± 6445	1.151			
Sitosteryl palmitate	0.105 ± 0.006	37136 ± 2971	1.040			

SK&F 97541	0.104 ± 0.003	29047 ± 1913	0.813			
SKA 31	0.118 ± 0.003	29850 ± 2588	0.836			
Skatole	0.109 ± 0.002	39197 ± 1132	1.097			
SKF 38393 hydrobromide	0.118 ± 0.003	36686 ± 6744	1.027			
SKF 525A hydrochloride	0.091 ± 0.012	45247 ± 2027	1.267			
SKF 60528	0.101 ± 0.005	27569 ± 1481	0.772			
SKF 75670 hydrobromide	0.099 ± 0.007	52873 ± 8459	1.480			
SKF 77434 hydrobromide	0.106 ± 0.006	22988 ± 2655	0.644			
SKF 81297 hydrobromide	0.101 ± 0.006	42142 ± 3221	1.180			
SKF 83566	0.123 ± 0.006	36578 ± 2242	1.024			
SKF 83566 hydrobromide	0.108 ± 0.001	24315 ± 809	0.681			
SKF 83959 hydrobromide	0.103 ± 0.008	40759 ± 6379	1.141			
SKF 86002	0.104 ± 0.009	37715 ± 1735	1.056			
SKF 86002 dihydrochloride	0.103 ± 0.005	29763 ± 7767	0.833			
SKF 86466	0.099 ± 0.009	32544 ± 2189	0.911			
SKF 89145 hydrobromide	0.101 ± 0.004	37819 ± 963	1.059			
SKF 89626	0.103 ± 0.006	27509 ± 5498	0.770			
SKF 89976A hydrochloride	0.108 ± 0.003	27207 ± 1514	0.762			
SKF 91488 dihydrochloride	0.114 ± 0.004	29111 ± 2333	0.815			
SKF 95282 dimaleate	0.099 ± 0.007	54990 ± 2912	1.520			
SKF 96365	0.108 ± 0.009	36280 ± 8178	1.016			
SKI II	0.110 ± 0.003	44123 ± 6414	1.235			
Skimmianine	0.096 ± 0.010	23482 ± 1038	0.657			
Skimmin	0.115 ± 0.003	37704 ± 2332	1.056			
Skullcapflavone I	0.127 ± 0.004	42905 ± 4072	1.201			
SL 327	0.111 ± 0.007	44240 ± 3406	1.239			
Sligrl	0.112 ± 0.002	29202 ± 929	0.818			
SLV 320	0.114 ± 0.002	37187 ± 1022	1.041			
SM-21 maleate	0.123 ± 0.002	36041 ± 4317	1.009			
SMC Proliferation Inhibitor-2w	0.108 ± 0.008	43603 ± 1810	1.221			
Smer28	0.107 ± 0.012	40733 ± 2747	1.141			
S-Methylisothiourea hemisulfate	0.105 ± 0.000	31786 ± 4527	0.890			

S-Methyl-L-thiocitrulline acetate	0.105 ± 0.009	40719 ± 2375	1.140			
Smilagenin	0.114 ± 0.005	31224 ± 2571	0.874			
Smilagenin acetate	0.109 ± 0.003	53123 ± 4663	1.487			
Smoothened Agonist, SAG	0.096 ± 0.002	37085 ± 1906	1.038			
Smyrindioloside	0.125 ± 0.015	28878 ± 3426	0.809			
SN 38	0.123 ± 0.006	30371 ± 1918	0.850			
SN 6	0.112 ± 0.002	40036 ± 6017	1.121			
SNAP	0.110 ± 0.001	32182 ± 630	0.901			
SNAP 5089	0.107 ± 0.008	35511 ± 1314	0.994			
SNAP 94847 hydrochloride	0.110 ± 0.007	18989 ± 2133	0.525			
SNC 121	0.107 ± 0.006	35672 ± 1052	0.999			
SNC 162	0.099 ± 0.008	42677 ± 1479	1.195			
SNC 80	0.100 ± 0.002	36233 ± 3341	1.015			
S-Nitrosoglutathione	0.105 ± 0.007	39306 ± 2553	1.101			
S-Nitroso-N-acetylpenicillamine	0.105 ± 0.010	37155 ± 2642	1.040			
SNS 032(BMS387032)	0.125 ± 0.005	40604 ± 2489	1.137			
SNS 314	0.123 ± 0.014	40801 ± 2886	1.142			
Sobuzoxane	0.099 ± 0.010	32266 ± 1970	0.903			
Sodium 4-Phenylbutyrate	0.108 ± 0.010	87449 ± 4981	2.558	0.113 ± 0.009	43756 ± 4316	1.339
Sodium cyclamate	0.095 ± 0.002 *					
Sodium fluoroacetate	0.072 ± 0.004 **					
Sodium gluconate	0.114 ± 0.002	22802 ± 1693	0.638			
Sodium monofluorophosphate	0.101 ± 0.007	41351 ± 9164	1.158			
Sodium nitroprusside	0.111 ± 0.006	29501 ± 4353	0.826			
Sodium nitroprusside dihydrate	0.107 ± 0.012	50404 ± 3707	1.411			
Sodium oxamate	0.096 ± 0.010	33938 ± 3533	0.950			
Sodium oxybate	0.104 ± 0.004	33476 ± 4321	0.937			
Sodium phenylacetate	0.117 ± 0.013	22498 ± 2019	0.630			
Sodium phenylbutyrate	0.102 ± 0.003	46055 ± 2934	1.290			
Sodium salicylate	0.113 ± 0.002 *					
Sodium taurocholate hydrate	0.091 ± 0.001 **					
Sodium tetradecyl sulfate	0.088 ± 0.003 **					

Sodium thioglycolate	0.115 ± 0.006	29862 ± 2053	0.836			
Solanesol	0.120 ± 0.006	36097 ± 1221	1.011			
Solanesyl acetate	0.115 ± 0.008	34896 ± 2889	0.977			
Solanidine	0.115 ± 0.007	31383 ± 1381	0.879			
Solanine, a -	0.119 ± 0.002	36088 ± 1010	1.010			
Solasodine	0.114 ± 0.009	32866 ± 606	0.920			
Solidagenone	0.104 ± 0.006	32057 ± 2855	0.898			
Solifenacin succinate	0.099 ± 0.007	33508 ± 7454	0.938			
Songorine	0.101 ± 0.010	21807 ± 578	0.648			
Sootepin D	0.117 ± 0.002	37819 ± 2649	1.059			
Sophocarpidine	0.121 ± 0.004	38394 ± 1647	1.075			
Sophocarpine	0.129 ± 0.005	30600 ± 2742	0.857			
Sophoraflavone G	0.072 ± 0.002 **					
Sophoridine	0.122 ± 0.002	42085 ± 2457	1.178			
Sorafenib	0.124 ± 0.005	28130 ± 3648	0.788			
Sorbitol	0.122 ± 0.003	31973 ± 3691	0.895			
Sorghumol	0.123 ± 0.008	46733 ± 4440	1.308			
Sorghumol acetate	0.117 ± 0.011	46084 ± 3866	1.290			
Sotalol hydrochloride	0.126 ± 0.006	23065 ± 3426	0.646			
Soyacerebroside I	0.124 ± 0.007	31937 ± 2942	0.894			
Soyacerebroside II	0.122 ± 0.004	39040 ± 2102	1.093			
Soyasapogenol B	0.120 ± 0.024	28924 ± 2426	0.810			
SP600125	0.112 ± 0.003	55391 ± 1321	1.551			
Spaglumic acid	0.124 ± 0.006	34926 ± 858	0.978			
Sparfloxacin	0.065 ± 0.002 **					
Spartein sulfate (-)-	0.122 ± 0.004	30318 ± 1444	0.849			
Sparteine sulfate	0.106 ± 0.002	20437 ± 2373	0.572			
Spathulenol	0.119 ± 0.016	33834 ± 2056	0.947			
Specioside	0.119 ± 0.002	31243 ± 1742	0.875			
Spectinomycin	0.114 ± 0.004	32683 ± 2335	0.915			
Spectinomycin dihydrochloride	0.110 ± 0.003	57272 ± 6354	1.563			
Spectinomycin hydrochloride	0.104 ± 0.010	16807 ± 1761	0.542			

Spectinomycin sulfate	0.100 ± 0.000 *					
Spermidine trihydrochloride	0.109 ± 0.012	48803 ± 2740	1.366			
Spermine quadrahydrochloride	0.110 ± 0.008	30318 ± 2721	0.849			
Sphingomyelin *	0.107 ± 0.002	27363 ± 865	0.766			
Sphingosine	0.103 ± 0.002	27628 ± 1031	0.774			
Sphingosine Kinase Inhibitor	0.109 ± 0.009	31479 ± 2015	0.881			
Sphingosine-1-phosphate *	0.107 ± 0.003	30531 ± 771	0.855			
Sphingosylphosphoryl choline *	0.110 ± 0.003	39669 ± 334	1.111			
Sphondin	0.112 ± 0.008	38376 ± 1722	1.075			
Spinizarine	0.065 ± 0.001 **					
Spiperone	0.113 ± 0.007	20107 ± 3702	0.563			
Spiperone hydrochloride	0.101 ± 0.002	29131 ± 5817	0.816			
Spiradine F	0.123 ± 0.002	43230 ± 2356	1.210			
Spiramilactone B	0.124 ± 0.001	42935 ± 827	1.202			
Spiramine A	0.125 ± 0.004	42713 ± 3633	1.196			
Spiramycin	0.122 ± 0.003	29360 ± 1276	0.822			
Spironolactone	0.103 ± 0.013	33798 ± 6330	0.946			
Spiroxatrine	0.100 ± 0.006	29638 ± 2506	0.830			
Splitomicin	0.117 ± 0.003	157633 ± 9514	4.790	0.116 ± 0.011	118249 ± 8616	3.619
SQ 22536	0.105 ± 0.008	28491 ± 2628	0.798			
SQ 29548	0.108 ± 0.003	33723 ± 572	0.944			
Squalene	0.110 ± 0.005	41416 ± 3332	1.160			
Squalene-2,3-diol	0.121 ± 0.003	34949 ± 2540	0.979			
Squamolone	0.113 ± 0.006	59600 ± 9055	1.669			
SR 11302	0.102 ± 0.004	30824 ± 784	0.863			
SR 202	0.107 ± 0.008	30066 ± 4170	0.842			
SR 2640	0.100 ± 0.006	37250 ± 9816	1.043			
SR 27897	0.112 ± 0.004	37755 ± 5163	1.057			
SR 33805 Oxalate	0.054 ± 0.003 **					
SR 3677	0.122 ± 0.007	35999 ± 5429	1.008			
SR 49059	0.100 ± 0.001	34207 ± 2785	0.958			
SR 57227A	0.101 ± 0.006	41584 ± 3971	1.164			

SR 59230A Oxalate	0.114 ± 0.005	25861 ± 2888	0.724			
SR 95531	0.110 ± 0.004	37714 ± 2966	1.056			
SR 95531 Hydrobromide	0.138 ± 0.004 **					
SR 95639A dihydrochloride	0.110 ± 0.007	52218 ± 4101	1.462			
Src I1	0.107 ± 0.007	43112 ± 818	1.207			
Src Kinase Inhibitor I	0.137 ± 0.012	32059 ± 1872	0.898			
Ssioriside	0.124 ± 0.006	34663 ± 1839	0.971			
S-Sulfo-L-cysteine sodium salt	0.100 ± 0.004	65461 ± 8603	1.750			
ST 148	0.103 ± 0.003	27661 ± 2034	0.774			
ST 91	0.114 ± 0.001	31097 ± 1371	0.871			
Stachydrine hydrochloride	0.098 ± 0.003	24273 ± 124	0.680			
Stanozolol	0.124 ± 0.004	86091 ± 3033	2.397	0.125 ± 0.004	29618 ± 1013	0.906
STAT1 Enhancer, 2-NP	0.098 ± 0.001	49381 ± 993	1.383			
STAT3 Inhibitor III, WP1066	0.096 ± 0.003	46379 ± 3536	1.299			
STAT3 Inhibitor VII	0.099 ± 0.021	34708 ± 6251	0.972			
STAT5 Inhibitor	0.108 ± 0.007	36381 ± 2839	1.019			
Statil	0.105 ± 0.006	30663 ± 3811	0.859			
Stattic	0.072 ± 0.012 **					
Staurosporine	0.107 ± 0.002	39945 ± 1925	1.118			
Staurosporine, N-benzoyl-	0.116 ± 0.007	29871 ± 3045	0.836			
Staurosporine, Streptomyces sp.	0.119 ± 0.026	34345 ± 1918	0.962			
Stavudine	0.126 ± 0.007	58302 ± 6572	1.623			
Ste11 Mapkkk Activation Inhibitor	0.130 ± 0.002	31095 ± 774	0.871			
Stellasterol	0.114 ± 0.005	33104 ± 835	0.927			
Stem-Cell Factor/c-Kit Inhibitor, ISCK03	0.102 ± 0.019	62287 ± 2036	1.845			
Stephanine	0.122 ± 0.002	47122 ± 5186	1.319			
Stephavanine	0.129 ± 0.008	53299 ± 1670	1.492			
Steppogenin	0.116 ± 0.003	37796 ± 2009	1.058			
Sterigmatocystin	0.110 ± 0.002	36895 ± 1684	1.033			
Steviol	0.103 ± 0.007	47976 ± 2000	1.343			
Stevioside	0.111 ± 0.003	24154 ± 3572	0.676			
Stictic acid	0.122 ± 0.004	43175 ± 3737	1.209			

Stigmast-4-ene-3,6-diol	0.120 ± 0.003	36179 ± 2959	1.013				
Stigmast-4-ene-3,6-dione	0.117 ± 0.004	40069 ± 1317	1.122				
Stigmasta-4,22,25-trien-3-one	0.123 ± 0.004	43368 ± 5383	1.214				
Stigmasta-4,22-dien-3-one	0.113 ± 0.008	39220 ± 4330	1.098				
Stigmasta-4,25-dien-3-one	0.123 ± 0.003	46591 ± 3422	1.305				
Stigmasta-5,8-dien-3-ol	0.125 ± 0.009	39148 ± 3202	1.096				
Stigmastane-3,5,6-triol	0.119 ± 0.001	31798 ± 1388	0.890				
Stigmastane-3,6-diol	0.136 ± 0.005	31188 ± 3032	0.873				
Stigmastane-3,6-dione	0.119 ± 0.004	31216 ± 1719	0.874				
Stigmasterol	0.120 ± 0.005	39959 ± 4165	1.119				
Stigmasterol glucoside	0.113 ± 0.003	31754 ± 1320	0.889				
Stiripentol	0.133 ± 0.007	65295 ± 5268	1.818				
STO-609	0.107 ± 0.008	30569 ± 1076	0.856				
Streptomycin	0.113 ± 0.007	36360 ± 1125	1.018				
Streptomycin sulfate	0.115 ± 0.005	25064 ± 1555	0.702				
Streptonigrin	0.062 ± 0.001 **						
Streptozocin	0.107 ± 0.009	7989 ± 1027	0.221	0.116 ± 0.009	36142 ± 2025	1.106	
Streptozotocin	0.112 ± 0.003	11221 ± 456	0.306	0.109 ± 0.006	30454 ± 1106	0.932	
Strictosamide	0.128 ± 0.002	33518 ± 724	0.938				
S-Trityl-L-cysteine	0.112 ± 0.002	29674 ± 3699	0.831				
Strophanthidin	0.098 ± 0.008	27959 ± 1067	0.783				
Strophanthidin acetate	0.102 ± 0.002	19393 ± 584	0.576				
Strophanthidinic acid lactone acetate	0.107 ± 0.021	28961 ± 1420	0.811				
Strychnine	0.094 ± 0.002 *						
Strychnine hydrochloride	0.102 ± 0.008	25855 ± 1369	0.724				
Strychnine methiodide	0.115 ± 0.003	31619 ± 2366	0.885				
SU 11652	0.061 ± 0.005 **						
SU 1498	0.122 ± 0.005	33605 ± 3982	0.941				
SU 3327	0.082 ± 0.002 **						
SU 4312	0.126 ± 0.005	36021 ± 2448	1.009				
SU 5402	0.129 ± 0.007	37333 ± 4884	1.045				
SU 5416	0.104 ± 0.009	28641 ± 7354	0.802				

SU 6656	0.106 ±0.005	29846 ±1421	0.836			
SU 6668	0.119 ±0.006	40141 ±1135	1.124			
SU 9516	0.076 ±0.010 **					
Subelliptenone G	0.117 ±0.005	40136 ±2787	1.124			
Suberosin	0.108 ±0.009	31198 ±2641	0.874			
Suberoyl bis Hydroxamic acid	0.108 ±0.005	32038 ±3113	0.897			
Substance P	0.113 ±0.002	31059 ±1262	0.870			
Succinic acid	0.121 ±0.003	37046 ±396	1.037			
Succinylcholine	0.109 ±0.004	28646 ±4112	0.802			
Succinylcholine chloride	0.102 ±0.001	32274 ±9237	0.904			
Succinylsulfathiazole	0.108 ±0.003	32112 ±2081	0.899			
Sucralfate	0.107 ±0.003	25864 ±1715	0.724			
Sucralose	0.104 ±0.018	30962 ±3756	0.867			
Sucrose	0.125 ±0.004	30719 ±2318	0.860			
Sulbactam	0.115 ±0.003	33382 ±1818	0.935			
Sulbentine	0.105 ±0.008	18314 ±359	0.528			
Sulconazole nitrate	0.057 ±0.003 **					
Sulfabenzamide	0.107 ±0.001	22904 ±1496	0.641			
Sulfacarbamide	0.102 ±0.009	25897 ±927	0.725			
Sulfacetamide	0.108 ±0.004	55021 ±8405	1.541			
Sulfacetamide sodic hydrate	0.112 ±0.003	19450 ±703	0.531			
Sulfachloropyridazine	0.111 ±0.004	13250 ±355	0.362	0.113 ±0.003	40853 ±4278	1.250
Sulfachlorpyridazine	0.106 ±0.002	20560 ±1123	0.576			
Sulfadiazine	0.111 ±0.003	44354 ±1411	1.242			
Sulfadimethoxine	0.104 ±0.003	138849 ±3034	3.949	0.105 ±0.010	33195 ±879	1.016
Sulfadimidine	0.099 ±0.004	37933 ±3306	1.062			
Sulfadoxine	0.108 ±0.002	44144 ±1410	1.236			
Sulfaethidole	0.103 ±0.002	27772 ±1679	0.778			
Sulfafurazole	0.101 ±0.002	51787 ±1800	1.450			
Sulfaguanidine	0.120 ±0.006	25703 ±2318	0.720			
Sulfalene	0.104 ±0.001	34143 ±2941	0.956			
Sulfamerazine	0.105 ±0.001	32613 ±2766	0.913			

Sulfameter	0.111 ± 0.005	29440 ± 1665	0.824			
Sulfamethazine	0.122 ± 0.001	29901 ± 3226	0.837			
Sulfamethazine sodium salt	0.107 ± 0.001	24958 ± 732	0.699			
Sulfamethizole	0.103 ± 0.003	36620 ± 882	1.025			
Sulfamethoxazole	0.097 ± 0.002	21527 ± 1725	0.603			
Sulfamethoxypyridazine	0.115 ± 0.003	36289 ± 4239	1.016			
Sulfamonomethoxine	0.106 ± 0.008	33733 ± 2625	0.945			
Sulfanilamide	0.115 ± 0.002	51519 ± 3875	1.443			
Sulfanilate zinc	0.103 ± 0.006	39720 ± 5811	1.112			
Sulfanitran	0.108 ± 0.006	19396 ± 1944	0.559			
Sulfaphenazole	0.106 ± 0.010	36087 ± 4810	1.010			
Sulfapyridine	0.112 ± 0.002	28495 ± 2464	0.798			
Sulfaquinoxaline sodium	0.102 ± 0.001	38581 ± 1575	1.080			
Sulfasalazine	0.109 ± 0.004	51491 ± 2880	1.442			
Sulfasuccinamide	0.121 ± 0.004	37118 ± 951	1.039			
Sulfathiazole	0.111 ± 0.008	41501 ± 2147	1.162			
Sulfinpyrazone	0.113 ± 0.004	38674 ± 1192	1.083			
Sulfisoxazole	0.100 ± 0.002 *					
Sulfisoxazole acetyl	0.108 ± 0.008	24969 ± 3003	0.699			
Sulfocostunolide A	0.110 ± 0.010	43290 ± 3308	1.212			
Sulfocostunolide B	0.127 ± 0.002	41851 ± 1123	1.172			
Sulfuretine	0.098 ± 0.005 *					
Sulindac	0.103 ± 0.015	34493 ± 8257	0.966			
Sulindac sulfide	0.101 ± 0.004	45671 ± 1311	1.279			
Sulindac sulfone	0.104 ± 0.005	29703 ± 6717	0.832			
sulisobenzone	0.099 ± 0.005	41947 ± 5656	1.174			
Sulmazole	0.118 ± 0.004	28774 ± 2141	0.806			
Suloctidil	0.112 ± 0.005	33717 ± 2265	0.944			
Sulpiride	0.113 ± 0.008	41500 ± 2189	1.162			
Sulpiride s (-)	0.110 ± 0.002	38159 ± 2479	1.068			
Sumatriptan succinate	0.101 ± 0.005	42684 ± 3972	1.195			
Sunitinib	0.117 ± 0.007	23343 ± 6796	0.654			

Supercinnamaldehyde	0.062 ± 0.003 **					
Suplatast tosylate	0.113 ± 0.010	40121 ± 764	1.123			
Suprofen	0.109 ± 0.006	36998 ± 4246	1.036			
Suramin	0.101 ± 0.014	34454 ± 2403	0.965			
Suramin hexasodium	0.102 ± 0.003	30093 ± 2620	0.843			
Suramin sodium	0.118 ± 0.002	35103 ± 1537	0.983			
Sutherlandin trans-p-coumarate	0.117 ± 0.005	35693 ± 4118	0.999			
Suxibuzone	0.112 ± 0.005	37533 ± 2785	1.051			
Swainsonine	0.105 ± 0.004	41003 ± 1164	1.148			
Sweroside	0.116 ± 0.006	40133 ± 4124	1.124			
Swertiamarin	0.100 ± 0.010	33070 ± 1254	0.926			
SX 011	0.103 ± 0.009	29806 ± 1572	0.835			
Syk Inhibitor	0.119 ± 0.017	26410 ± 1646	0.739			
Syk Inhibitor II	0.114 ± 0.010	21573 ± 261	0.604			
Syk Inhibitor III	0.087 ± 0.001 **					
SYM 2081	0.108 ± 0.005	19215 ± 2932	0.538			
SYM 2206	0.106 ± 0.006	35061 ± 5715	0.982			
Symclosene	0.100 ± 0.003	27142 ± 1872	0.760			
Syndyphalin SD-25	0.104 ± 0.006	52900 ± 5675	1.481			
Synephrine	0.127 ± 0.005	55897 ± 2188	1.556			
Synthalin sulfate	0.100 ± 0.012	29257 ± 5396	0.819			
Syringaresinol diacetate	0.130 ± 0.004	33553 ± 1436	0.939			
Syringetine-3-O-glucoside	0.102 ± 0.002	51879 ± 2012	1.541			
Syringic acid	0.110 ± 0.005	38470 ± 1710	1.077			
Syringin	0.112 ± 0.005	33234 ± 1154	0.931			
Syringin pentaacetate	0.131 ± 0.007	27911 ± 1666	0.781			
Syrosingopine	0.104 ± 0.002	25907 ± 724	0.725			
T 0156 hydrochloride	0.105 ± 0.009	35782 ± 622	1.002			
T0070907	0.108 ± 0.005	28884 ± 1199	0.809			
T0901317	0.110 ± 0.003	44644 ± 622	1.250			
T113242	0.101 ± 0.007	41158 ± 4472	1.152			
T-638	0.063 ± 0.005 **					

Tabersonine	0.136 ± 0.008	30954 ± 1909	0.867			
Tabimorelin	0.108 ± 0.001	32321 ± 805	0.905			
Tachioside	0.115 ± 0.005	50854 ± 2975	1.424			
Tacrine hydrochloride	0.088 ± 0.003 **					
Tacrolimus	0.113 ± 0.008	32220 ± 1939	0.902			
Tadalafil	0.113 ± 0.001	42882 ± 2486	1.201			
Tagitinin A	0.122 ± 0.004	39904 ± 2003	1.117			
Tagitinin F	0.127 ± 0.009	28919 ± 662	0.810			
Taiwanhomoflavone B	0.117 ± 0.004	39588 ± 3997	1.108			
TAK 165	0.106 ± 0.004	31349 ± 2248	0.878			
TAK 715	0.123 ± 0.005	29368 ± 2704	0.822			
Talampicillin hydrochloride	0.117 ± 0.019	12065 ± 2018	0.329	0.115 ± 0.009	39618 ± 1525	1.212
Talniflumate	0.111 ± 0.003	31149 ± 891	0.872			
Tamarixetin	0.132 ± 0.006	43618 ± 4614	1.221			
Tamarixetine	0.103 ± 0.006	21366 ± 406	0.635			
Tamoxifen	0.095 ± 0.007	60010 ± 5568	1.680			
Tamoxifen citrate	0.066 ± 0.002 **					
Tamsulosin hydrochloride	0.115 ± 0.002	22218 ± 1859	0.622			
Tanachin	0.111 ± 0.014	1475 ± 102	0.043	0.113 ± 0.004	17493 ± 1573	0.535
Tandutinib	0.127 ± 0.004	32917 ± 1177	0.922			
Tangeritin	0.110 ± 0.008	39273 ± 3322	1.100			
Tannic acid	0.077 ± 0.005 **					
Tanshinlactone	0.134 ± 0.010	32207 ± 382	0.902			
Tanshinone I	0.114 ± 0.001	38124 ± 3594	1.067			
Tanshinone II	0.128 ± 0.005	36738 ± 2848	1.029			
Tanshinone IIA	0.096 ± 0.004 *					
Tanshinone iia sulfonate sodium	0.103 ± 0.025	36897 ± 1448	1.033			
Tanshinone IIB	0.104 ± 0.025	48266 ± 2161	1.351			
Tapentadol hydrochloride	0.102 ± 0.004	39809 ± 4748	1.115			
Taraxasterol	0.117 ± 0.009	39107 ± 1757	1.095			
Taraxasterol acetate	0.122 ± 0.004	36501 ± 4920	1.022			
Taraxerol	0.124 ± 0.004	44157 ± 5079	1.236			

Taraxerone	0.113 ± 0.008	32880 ± 3074	0.921			
Taraxeryl acetate	0.115 ± 0.003	39935 ± 486	1.118			
TAS-301	0.105 ± 0.004	56165 ± 8516	1.573			
Tatarinoid A	0.115 ± 0.001	35304 ± 3249	0.988			
Taurine	0.109 ± 0.012	29064 ± 3161	0.814			
Taurocholic acid sodium salt	0.123 ± 0.002	28109 ± 2573	0.787			
Taurodeoxycholic acid sodium salt	0.121 ± 0.003	48652 ± 329	1.362			
Taxayunnansin A	0.117 ± 0.005	40804 ± 2559	1.142			
Taxifolin	0.122 ± 0.001	50154 ± 2991	1.404			
Taxifolin 3-O-β-D-xylopyranoside	0.114 ± 0.002	51912 ± 1691	1.454			
Taxifolin-(+)	0.127 ± 0.006	31776 ± 2483	0.890			
Taxifolin-(+/-)	0.127 ± 0.009	106283 ± 12277	2.959	0.121 ± 0.004	47159 ± 5294	1.443
Taxinine B	0.121 ± 0.005	47692 ± 2801	1.335			
Taxiphyllin	0.115 ± 0.002	25235 ± 983	0.707			
Taxol	0.101 ± 0.000	43710 ± 1790	1.224			
Tazobactam	0.110 ± 0.009	42971 ± 3982	1.203			
TBB	0.092 ± 0.002 *					
TBBz	0.089 ± 0.002	47032 ± 4033	1.317			
TC 1	0.114 ± 0.008	26770 ± 2616	0.750			
TC 2559 difumarate	0.104 ± 0.003	36401 ± 3536	1.019			
Tcpobop	0.126 ± 0.003	42989 ± 1176	1.204			
TCS 2002	0.107 ± 0.002	29843 ± 4759	0.836			
TCS 359	0.114 ± 0.003	35397 ± 2968	0.991			
Tebrofen	0.118 ± 0.004	62149 ± 9829	1.740			
Tectochrysin	0.119 ± 0.007	53562 ± 1628	1.500			
Tegafur	0.114 ± 0.012	35863 ± 2170	1.004			
Tegaserod maleate	0.115 ± 0.003	39697 ± 5098	1.111			
Teicoplanin	0.051 ± 0.001 **					
Telenzepine dihydrochloride	0.108 ± 0.009	25615 ± 2699	0.717			
Telithromycin	0.125 ± 0.002	43475 ± 4776	1.217			
Telmisartan	0.105 ± 0.007	30948 ± 1987	0.867			
Telomerase Inhibitor IX	0.063 ± 0.007 **					

Temozolamide	0.107 ± 0.017	48813 ± 7288	1.367			
Temozolomide	0.124 ± 0.008	36296 ± 5129	1.016			
Tempo	0.132 ± 0.006 *					
Tempol	0.118 ± 0.004	35121 ± 1615	0.983			
Tenacigenin B	0.118 ± 0.004	40629 ± 1883	1.138			
Tenacigenoside A	0.123 ± 0.005	33048 ± 2024	0.925			
Tenacissoside G	0.126 ± 0.001	30094 ± 1250	0.843			
Tenacissoside I	0.123 ± 0.001	29494 ± 555	0.826			
Tenatoprazole	0.106 ± 0.003	71384 ± 11085	1.987			
Tenidap	0.108 ± 0.003	31285 ± 755	0.876			
Teniposide	0.072 ± 0.010 **					
Tenofovir	0.102 ± 0.007	30414 ± 2767	0.852			
Tenonitrozole	0.072 ± 0.008 **					
Tenovin-1	0.099 ± 0.006	31807 ± 1981	0.891			
Tenoxicam	0.122 ± 0.007	22924 ± 1129	0.642			
Tenylidone	0.105 ± 0.010	31736 ± 2976	0.889			
Tepoxalin	0.068 ± 0.007 **					
Terazosin	0.103 ± 0.006	38629 ± 4743	1.082			
Terazosin hydrochloride	0.112 ± 0.004	29232 ± 3351	0.818			
Terbacil	0.111 ± 0.009	50451 ± 4933	1.413			
Terbinafine	0.130 ± 0.001 *					
Terbinafine hydrochloride	0.063 ± 0.006 **					
Terbutaline hemisulfate	0.108 ± 0.008	34523 ± 3060	0.967			
Terconazole	0.111 ± 0.002	49744 ± 4786	1.393			
Tereticornate A	0.115 ± 0.005	40835 ± 1625	1.143			
Terfenadine	0.108 ± 0.008	40730 ± 768	1.140			
Terpene hydrate	0.102 ± 0.006	35661 ± 1563	0.998			
Terreic acid	0.083 ± 0.006 **					
Tert-butylhydroquinone	0.097 ± 0.008	44918 ± 3411	1.258			
Testosterone	0.130 ± 0.002 *					
Testosterone acetate	0.113 ± 0.006	27064 ± 2682	0.758			
Testosterone decanoate	0.120 ± 0.002	43319 ± 1150	1.213			

Testosterone propionate	0.111 ± 0.011	42089 ± 368	1.178			
Tetrabenazine	0.104 ± 0.005	38406 ± 5034	1.075			
Tetracaine hydrochloride	0.103 ± 0.005	37236 ± 1849	1.043			
Tetrachloroisophthalonitrile	0.101 ± 0.002	38194 ± 3241	1.069			
Tetracosanoic acid	0.107 ± 0.013	29796 ± 2908	0.834			
Tetracycline	0.110 ± 0.005	41132 ± 3599	1.152			
Tetracycline hydrochloride	0.111 ± 0.003	30149 ± 1114	0.844			
Tetradecanoic acid	0.106 ± 0.012	34682 ± 2886	0.971			
Tetradecylthioacetic acid	0.118 ± 0.005	50724 ± 1073	1.420			
Tetraethylammonium chloride	0.107 ± 0.013	29431 ± 1946	0.824			
Tetraethylenepentamine pentahydrochloride	0.120 ± 0.001	55523 ± 4407	1.555			
Tetraethylthiuram disulfide	0.112 ± 0.007	49539 ± 4243	1.387			
Tetrahydroalstonine	0.101 ± 0.016	17519 ± 1423	0.520			
Tetrahydroamentoflavone	0.111 ± 0.003	25748 ± 801	0.721			
Tetrahydrocannabinol-7-oic acid	0.110 ± 0.007	38234 ± 696	1.071			
Tetrahydrogambogic acid	0.111 ± 0.016	32323 ± 2919	0.905			
Tetrahydroharmine	0.104 ± 0.011	28670 ± 4366	0.803			
Tetrahydrolipstatin	0.104 ± 0.002	23707 ± 207	0.664			
Tetrahydropalmatine	0.138 ± 0.023	41795 ± 3670	1.170			
Tetrahydropapaverine	0.093 ± 0.006 *					
Tetrahydrosappanone a trimethyl ether	0.106 ± 0.009	48252 ± 2883	1.351			
Tetrahydrouridine	0.099 ± 0.006	45474 ± 1624	1.273			
Tetrahydroxy-1,4-quinone monohydrate	0.128 ± 0.013	42977 ± 1489	1.203			
Tetrahydroxysqualene	0.108 ± 0.010	39681 ± 2241	1.111			
Tetrahydrozoline hydrochloride	0.110 ± 0.003	30558 ± 1302	0.856			
Tetraisopropyl pyrophosphoramide	0.103 ± 0.003	28960 ± 2898	0.811			
Tetramethylpyrazine	0.113 ± 0.004	33114 ± 1901	0.927			
Tetramethylscutellarein	0.105 ± 0.007	53039 ± 4036	1.485			
Tetramisole hydrochloride	0.124 ± 0.005	29805 ± 2547	0.835			
Tetrandrine	0.102 ± 0.007	27941 ± 1799	0.782			
Tetranor-12(R)-HETE	0.104 ± 0.003	35763 ± 2304	1.001			
Tetrindole mesylate	0.088 ± 0.001 **					

Teuclatriol	0.128 ± 0.008	35205 ± 142	0.986			
Teuvincenone H	0.116 ± 0.009	28519 ± 4070	0.799			
Tfllr-NH_2	0.109 ± 0.001	21662 ± 1423	0.607			
Tfmpp	0.127 ± 0.008	62912 ± 8971	1.762			
TG003	0.092 ± 0.010	48299 ± 5624	1.352			
TGF-b RI Inhibitor III	0.100 ± 0.014	29785 ± 3413	0.834			
TGF-b RI Kinase Inhibitor	0.121 ± 0.011	22545 ± 2175	0.631			
TGX221	0.119 ± 0.013	29897 ± 907	0.837			
Thalidomide	0.106 ± 0.006	39150 ± 2652	1.096			
Thapsigargin	0.107 ± 0.013	31674 ± 786	0.887			
Theaflavin	0.105 ± 0.005	31572 ± 2675	0.884			
Theaflavin monogallates	0.103 ± 0.004	88571 ± 5801	2.551	0.101 ± 0.008	185877 ± 10394	5.688
Theanine potassium	0.106 ± 0.007	27383 ± 2263	0.767			
Theobromine	0.111 ± 0.002	25243 ± 1796	0.707			
Theophylline	0.114 ± 0.006	33564 ± 3107	0.940			
Thermopsine perchlorate	0.115 ± 0.005	38687 ± 2806	1.083			
Thevebioside	0.123 ± 0.011	39492 ± 1595	1.106			
Theviridoside	0.130 ± 0.007	39197 ± 524	1.097			
Thiabendazole	0.104 ± 0.006	57439 ± 5222	1.608			
Thiamine	0.105 ± 0.017	33358 ± 2441	0.934			
Thiamine hydrochloride	0.114 ± 0.001	51723 ± 2190	1.448			
Thiamphenicol	0.122 ± 0.010	4271 ± 270	0.117	0.119 ± 0.007	18149 ± 1539	0.555
Thiamphenicol glycinate	0.119 ± 0.002	7421 ± 447	0.211	0.114 ± 0.015	21680 ± 3489	0.663
Thiamylal sodium	0.102 ± 0.005	21090 ± 2103	0.591			
Thiethylperazine dimalate	0.103 ± 0.001					
Thimerosal	0.059 ± 0.003 **					
Thiocitrulline [L-Thiocitrulline hydrochloride]	0.117 ± 0.002	28485 ± 915	0.798			
Thiocolchicoside	0.113 ± 0.002	52552 ± 7038	1.471			
Thioctic acid	0.102 ± 0.001	4712 ± 160	0.136	0.109 ± 0.007	8110 ± 399	0.248
Thiodiglycol	0.116 ± 0.002	27950 ± 843	0.783			
Thioguanine	0.112 ± 0.003	27873 ± 2153	0.780			
Thioguanosine	0.112 ± 0.003	55453 ± 2572	1.513			

Thiolactomycin	0.092 ± 0.004	56056 ± 7213	1.549			
Thio-L-citrulline	0.106 ± 0.006	30137 ± 2003	0.844			
Thio-NADP sodium	0.104 ± 0.010	13089 ± 477	0.362	0.105 ± 0.008	25159 ± 4462	0.770
Thiopental sodium	0.118 ± 0.016	37545 ± 2373	1.051			
Thioperamide	0.100 ± 0.011	26719 ± 1010	0.748			
Thioperamide maleate	0.108 ± 0.002	51643 ± 2906	1.446			
Thiophene, 5-bromo-2-(4-fluorophenyl)-3-[4-(methylsulfonyl)-phenyl]	0.117 ± 0.003	50193 ± 4127	1.405			
Thioproperazine dimesylate	0.114 ± 0.001	29056 ± 644	0.814			
Thioridazine hydrochloride	0.104 ± 0.007	50552 ± 1502	1.415			
Thiorphan	0.108 ± 0.002	30148 ± 675	0.844			
Thiorphan (DL)	0.117 ± 0.004	38054 ± 2252	1.065			
Thiostrepton	0.075 ± 0.006 **					
Thiotepa	0.103 ± 0.003	28015 ± 2503	0.784			
Thiothixene	0.117 ± 0.017	18460 ± 1634	0.532			
Thiothixene hydrochloride	0.109 ± 0.002	26849 ± 2133	0.752			
Thiourea	0.113 ± 0.003	33593 ± 4354	0.941			
THIP hydrochloride	0.110 ± 0.011	33858 ± 972	0.948			
Thiram	0.072 ± 0.003 **					
Thonzonium bromide	0.059 ± 0.006 **					
Thonzylamine hydrochloride	0.103 ± 0.006	31862 ± 2265	0.892			
threo-1-C-Syringylglycerol	0.123 ± 0.004	30539 ± 3436	0.855			
threo-Guaiacylglycerol	0.114 ± 0.006	41287 ± 3341	1.156			
threo-Guaiacylglycerol β-coniferyl ether	0.127 ± 0.009	35232 ± 5539	0.986			
Threonine (L)	0.099 ± 0.012	31376 ± 3175	0.879			
Thromboxane B2	0.106 ± 0.004	32854 ± 1201	0.920			
Thunberginol C	0.119 ± 0.006	46589 ± 1273	1.304			
Thymidine	0.128 ± 0.001	27465 ± 2398	0.769			
Thymopentin	0.118 ± 0.021	29909 ± 1821	0.837			
Thymoquinone	0.128 ± 0.002	33136 ± 984	0.928			
Thyroid Hormone Receptor Antagonist, 1-850	0.098 ± 0.002	50554 ± 3721	1.415			
Thyroxine (L)	0.138 ± 0.009	31099 ± 4693	0.871			
Tiabendazole	0.106 ± 0.014	51407 ± 5546	1.439			

Tiagabine hydrochloride	0.124 ± 0.012	19594 ± 1789	0.546			
Tianeptine	0.115 ± 0.008	9856 ± 467	0.273	0.115 ± 0.008	21864 ± 2409	0.669
Tianeptine sodium	0.094 ± 0.007	11406 ± 865	0.336	0.103 ± 0.014	38771 ± 3189	1.186
Tiapride hydrochloride	0.110 ± 0.007	30727 ± 758	0.860			
Tiaprofenic acid	0.109 ± 0.003	36815 ± 910	1.031			
Tibolone	0.130 ± 0.003	29314 ± 1497	0.821			
Ticarcillin sodium	0.122 ± 0.008	19958 ± 1263	0.545			
Ticlopidine	0.106 ± 0.010	32734 ± 6020	0.917			
Ticlopidine hydrochloride	0.120 ± 0.009	42241 ± 7697	1.183			
Tifenamil	0.113 ± 0.005	34665 ± 3515	0.971			
Tigecycline	0.109 ± 0.013	40233 ± 5476	1.127			
Tigogenin	0.109 ± 0.007	35114 ± 2837	0.983			
Tiletamine hydrochloride	0.119 ± 0.009	36936 ± 711	1.034			
Tilifodiolide	0.113 ± 0.006	32197 ± 502	0.901			
Tiliroside	0.130 ± 0.004	26730 ± 1816	0.748			
Tilmicosin	0.113 ± 0.006	17423 ± 612	0.502			
Tilorone	0.111 ± 0.006	31103 ± 4643	0.871			
Timolol maleate	0.115 ± 0.004	20037 ± 3053	0.561			
Timolol maleate (S)	0.107 ± 0.006	40584 ± 1430	1.136			
Timonacic	0.102 ± 0.001	40257 ± 1612	1.127			
Tinidazole	0.109 ± 0.006	56212 ± 7973	1.574			
Tinoridine	0.110 ± 0.006	46400 ± 1083	1.299			
Tioconazole	0.055 ± 0.001 **					
Tiopronin	0.116 ± 0.003	33437 ± 1698	0.936			
Tiotidine	0.103 ± 0.012	26269 ± 1126	0.736			
Tiotropium bromide	0.117 ± 0.006	43704 ± 4189	1.224			
Tioxidazole	0.112 ± 0.012	43932 ± 2911	1.230			
Tioxolone	0.108 ± 0.003	41069 ± 4172	1.150			
Tiratricol	0.095 ± 0.006	24159 ± 2510	0.676			
Tiratricol, 3,3',5-triiodothyroacetic acid	0.114 ± 0.004	30762 ± 2562	0.861			
Tirotundin	0.130 ± 0.010	39840 ± 4284	1.115			
Tizanidine hydrochloride	0.108 ± 0.011	34393 ± 365	0.963			

TJU103	0.102 ± 0.002	32171 ± 2166	0.901			
TK-44	0.108 ± 0.012	39139 ± 2883	1.096			
TLCK	0.118 ± 0.007	27648 ± 1105	0.774			
TMB-8	0.125 ± 0.007	54421 ± 5862	1.654			
TMB-8 hydrochloride	0.103 ± 0.003	61780 ± 6251	1.708			
TMPH hydrochloride	0.106 ± 0.006	31000 ± 6092	0.868			
TMS	0.104 ± 0.005	39799 ± 3783	1.114			
TNF-α Antagonist III, R-7050	0.098 ± 0.009	38551 ± 6298	1.079			
TNF-α Inhibitor	0.059 ± 0.004 **					
Tobramycin	0.076 ± 0.006 **					
Tobramycin (free base)	0.106 ± 0.003	37230 ± 2175	1.042			
Tocainide	0.132 ± 0.001 **					
Tocainide hydrochloride	0.096 ± 0.011	31236 ± 3908	0.875			
Tocopherol	0.115 ± 0.008	49030 ± 1625	1.369			
Tocopherol succinate	0.124 ± 0.005	30596 ± 1250	0.857			
Tocopherylquinone	0.101 ± 0.002	37653 ± 2365	1.054			
Toddalosin	0.122 ± 0.006	41548 ± 2957	1.163			
Todralazine hydrochloride	0.131 ± 0.007	58611 ± 3743	1.600			
Tolazamide	0.109 ± 0.002	30129 ± 982	0.844			
Tolazoline hydrochloride	0.111 ± 0.006	44033 ± 3402	1.233			
Tolbutamide	0.107 ± 0.003	36275 ± 1058	1.016			
Tolcapone	0.099 ± 0.001	52626 ± 1851	1.473			
Tolfenamic acid	0.102 ± 0.002	55024 ± 2171	1.541			
Toll-Like Receptor 7 Ligand II	0.095 ± 0.003	43400 ± 5116	1.215			
Tolmetin Na	0.115 ± 0.006	46877 ± 3087	1.313			
Tolnaftate	0.111 ± 0.005	36630 ± 1011	1.026			
Tolonium chloride	0.102 ± 0.003	18845 ± 436	0.543			
Tolperisone hydrochloride	0.113 ± 0.012	37824 ± 1514	1.059			
Tolterodine tartrate	0.117 ± 0.006	25477 ± 423	0.713			
Toltrazuril	0.113 ± 0.012	49228 ± 6306	1.378			
Tomatidine	0.072 ± 0.017 *	43388 ± 1185	1.215			
Tomatine	0.112 ± 0.010	42496 ± 5821	1.190			

Tombozine	0.128 ± 0.006	36105 ± 4923	1.011			
Tomentin	0.130 ± 0.033	32221 ± 5680	0.902			
Tomoxetine	0.102 ± 0.010	26847 ± 759	0.752			
Tomoxetine hydrochloride	0.118 ± 0.011	48059 ± 2319	1.346			
Topiramate	0.111 ± 0.002	39865 ± 2153	1.116			
Topotecan	0.113 ± 0.001	26550 ± 2766	0.743			
Topotecan hydrochloride	0.106 ± 0.001	24308 ± 4690	0.681			
Torachrysone	0.126 ± 0.003	27759 ± 870	0.777			
Torachrysone 8-O-glucoside	0.117 ± 0.012	33661 ± 4191	0.942			
Toradol	0.055 ± 0.006 **					
Toremifene	0.109 ± 0.003	51897 ± 2703	1.453			
Toremifene citrate	0.099 ± 0.001					
Toremiphene citrate	0.102 ± 0.005	32864 ± 3838	0.920			
Tormentic acid	0.118 ± 0.002	30461 ± 997	0.853			
Torsemide	0.117 ± 0.002	34411 ± 2365	0.963			
Tosufloxacin	0.062 ± 0.003 **					
Tosufloxacin hydrochloride	0.068 ± 0.001 **					
Tosufloxacin tosylate	0.074 ± 0.005 **					
Tosyl-Phe-CMK (TPCK)	0.112 ± 0.005	17690 ± 4503	0.525			
Totaradiol	0.119 ± 0.004	35932 ± 1314	1.006			
Totarol	0.132 ± 0.005	32765 ± 1973	0.917			
Totarol-19-carboxylic acid, methyl ester	0.102 ± 0.006	35123 ± 635	0.983			
TPCA-1	0.104 ± 0.002	18091 ± 706	0.501			
TPCK	0.092 ± 0.009 *					
TPEN	0.099 ± 0.005	142034 ± 10978	4.316	0.109 ± 0.005	47882 ± 8431	1.465
Tpl2 Kinase Inhibitor	0.105 ± 0.004	31179 ± 1633	0.873			
Tpl2 Kinase Inhibitor II	0.113 ± 0.003	17742 ± 1090	0.525			
TPMPA	0.111 ± 0.007	27508 ± 368	0.770			
Tracazolate	0.109 ± 0.004	34952 ± 1894	0.979			
Tracazolate hydrochloride	0.111 ± 0.005	33912 ± 2599	0.950			
Tramadol hydrochloride	0.108 ± 0.007	37345 ± 2732	1.046			
Tramiprosate	0.103 ± 0.006	35994 ± 3838	1.008			

Trandolapril	0.103 ± 0.008	40855 ± 1437	1.144			
Tranexamic acid	0.107 ± 0.004	26035 ± 1077	0.729			
Tranilast	0.107 ± 0.007	30897 ± 2560	0.865			
trans-(±)-ACPD	0.101 ± 0.004	29351 ± 3214	0.822			
trans-4-Aminocrotonic acid	0.103 ± 0.011	31081 ± 1101	0.870			
trans-4-Hydroxycinnamic acid	0.121 ± 0.004	45601 ± 3791	1.277			
trans-4-Hydroxycrotonic acid	0.105 ± 0.005	42676 ± 3284	1.195			
trans-7-Hydroxy-PIPAT maleate	0.113 ± 0.004	38042 ± 2366	1.065			
trans-Azetidine-2,4-dicarboxylic acid	0.107 ± 0.004	32440 ± 2686	0.908			
trans-Hinokiresinol	0.122 ± 0.003	41883 ± 3888	1.173			
trans-Retinoic Acid	0.120 ± 0.005 *					
trans-Triprolidine hydrochloride	0.104 ± 0.005	32023 ± 4862	0.897			
Tranylcypromine	0.112 ± 0.007	38102 ± 471	1.067			
Tranylcypromine hydrochloride	0.109 ± 0.010	31263 ± 2530	0.875			
Tranylcypromine sulfate	0.111 ± 0.006	24329 ± 2328	0.681			
Trap 101	0.116 ± 0.002	23822 ± 1562	0.667			
Trapidil	0.116 ± 0.010	36628 ± 4580	1.026			
Travoprost	0.100 ± 0.002	31130 ± 4784	0.872			
Trazodone hydrochloride	0.116 ± 0.005	36098 ± 4967	1.011			
Tremorine dihydrochloride	0.117 ± 0.001	34940 ± 1615	0.978			
Tremulacin	0.118 ± 0.002	62335 ± 3170	1.736			
Trequinsin	0.114 ± 0.003	40333 ± 1919	1.129			
Trequinsin hydrochloride	0.115 ± 0.004	57233 ± 2741	1.602			
Tretinoin	0.105 ± 0.004	33525 ± 4904	0.939			
Triacanthine	0.113 ± 0.009	26719 ± 3567	0.748			
Triacetin	0.118 ± 0.006	24248 ± 1347	0.679			
Triacetonamine	0.125 ± 0.002	30861 ± 954	0.864			
Triacetonamine hydrochloride	0.123 ± 0.005	24921 ± 2020	0.698			
Triacetylresveratrol	0.115 ± 0.003	33149 ± 2643	0.928			
Triadimefon	0.123 ± 0.004	48368 ± 5297	1.354			
Triamcinolone	0.112 ± 0.002	38464 ± 6855	1.077			
Triamcinolone acetonide	0.120 ± 0.004	48246 ± 2586	1.351			

Triamcinolone diacetate	0.115 ± 0.003	25854 ± 2864	0.724			
Triamterene	0.112 ± 0.004	30376 ± 148	0.851			
Tribenoside	0.113 ± 0.012	47499 ± 2759	1.330			
Trichlorfon	0.117 ± 0.004	52084 ± 3259	1.458			
Trichlormethiazide	0.107 ± 0.013	44264 ± 3407	1.239			
Trichlormethine hydrochloride	0.111 ± 0.005	58098 ± 4713	1.674			
Trichloromethiazide	0.114 ± 0.002	43014 ± 2957	1.204			
Trichodesmine	0.100 ± 0.005	21536 ± 320	0.640			
Trichorabdal A	0.112 ± 0.008	8213 ± 340	0.230	0.102 ± 0.007	10534 ± 1358	0.322
Trichostatin A	0.109 ± 0.012	36017 ± 1474	1.008			
Tricin	0.113 ± 0.013	46313 ± 3872	1.297			
Triciribine	0.125 ± 0.005	34357 ± 3137	0.962			
Triclabendazole	0.121 ± 0.009	206375 ± 32703	5.746	0.117 ± 0.007	39075 ± 3911	1.196
Triclazan	0.110 ± 0.020	34746 ± 3729	0.973			
Triclosan	0.111 ± 0.001	70750 ± 5785	1.931			
Tricosanoic acid	0.104 ± 0.015	25602 ± 3763	0.717			
Tridecanoic acid	0.123 ± 0.007	29174 ± 2290	0.817			
Tridihexethyl chloride	0.113 ± 0.008	21898 ± 2585	0.613			
Trientine hydrochloride	0.106 ± 0.009	22247 ± 1488	0.623			
Trifluoperazine	0.113 ± 0.006	46894 ± 6163	1.313			
Trifluoperazine dihydrochloride	0.097 ± 0.010	30229 ± 2045	0.846			
Trifluoperazine hydrochloride	0.089 ± 0.003 **					
Trifluperidol dihydrochloride	0.112 ± 0.004	22336 ± 2451	0.625			
Triflupromazine hydrochloride	0.113 ± 0.009	20710 ± 1625	0.572			
Trifluralin	0.113 ± 0.008	41402 ± 119	1.159			
Trifluridine	0.067 ± 0.017 **					
Triflusal	0.107 ± 0.001	47029 ± 3468	1.317			
Trifolirhizin	0.130 ± 0.003	47786 ± 2332	1.338			
Trigonelline	0.127 ± 0.005	36846 ± 4198	1.032			
Trigonelline hydrochloride	0.100 ± 0.005	23965 ± 582	0.671			
Trihexyphenidyl hydrochloride	0.111 ± 0.004	32440 ± 1265	0.908			
Trihexyphenidyl-D,L Hydrochloride	0.117 ± 0.002	42634 ± 2772	1.194			

Trileptal	0.119 ± 0.004	38504 ± 4069	1.078			
Trilostane	0.105 ± 0.006	29726 ± 2051	0.832			
Trim	0.105 ± 0.003	26549 ± 2550	0.743			
Trimebutine maleate	0.115 ± 0.010	36640 ± 3718	1.026			
Trimedlure	0.108 ± 0.006	37593 ± 2870	1.053			
Trimeprazine tartrate	0.117 ± 0.005	52537 ± 1354	1.434			
Trimetazidine Dihydrochloride	0.108 ± 0.010	37314 ± 1393	1.045			
Trimethadione	0.108 ± 0.002	40052 ± 1678	1.121			
Trimethobenzamide hydrochloride	0.120 ± 0.012	31364 ± 428	0.878			
Trimethoprim	0.118 ± 0.004	44515 ± 3148	1.246			
Trimethylpsoralen, 4,5',8-	0.104 ± 0.004	36796 ± 1586	1.030			
Trimetozine	0.107 ± 0.006	24384 ± 2222	0.683			
Trimipramine maleate	0.116 ± 0.013	26327 ± 2075	0.737			
Trioxsalen	0.111 ± 0.003	35839 ± 1735	1.003			
Tripelennamine citrate	0.112 ± 0.006	38586 ± 5278	1.080			
Tripelennamine hydrochloride	0.110 ± 0.001	46004 ± 1456	1.288			
Triprolidine hydrochloride	0.115 ± 0.016	46273 ± 407	1.296			
Tripterifordin	0.118 ± 0.001	34132 ± 852	0.956			
Triptocallic acid A	0.118 ± 0.008	32238 ± 3418	0.903			
Triptocallic acid D	0.137 ± 0.009	32141 ± 5793	0.900			
Triptohypol F	0.123 ± 0.006	37395 ± 2736	1.047			
Triptolide	0.138 ± 0.002 **					
Triptophenolide	0.121 ± 0.005	39343 ± 4902	1.102			
Triptoquinone B	0.120 ± 0.005	27335 ± 2176	0.765			
Triptoquinonide	0.129 ± 0.007	27807 ± 646	0.779			
Triptorelin	0.122 ± 0.004	22206 ± 1244	0.622			
Triptotriterpenic acid A	0.110 ± 0.003	42252 ± 2504	1.183			
Trisodium ethylenediamine tetracetate	0.094 ± 0.003 *					
Tritiozine	0.108 ± 0.006	34314 ± 4129	0.961			
Troclosene potassium	0.106 ± 0.006	26816 ± 1913	0.751			
Troglitazone	0.124 ± 0.002	39712 ± 2293	1.112			
Troleandomycin	0.103 ± 0.002	4398 ± 99	0.131	0.109 ± 0.006	7713 ± 827	0.236

Trolox	0.135 ± 0.003 **					
Tropanyl 2-phenylthiobutanoate oxalate	0.102 ± 0.010	42383 ± 5433	1.187			
Tropanyl-2-(4-bromophenyl)-propionate	0.101 ± 0.008	37826 ± 3630	1.059			
Tropanyl-3,5-dimethylbenzoate	0.097 ± 0.003 *					
Tropicamide	0.109 ± 0.001	41339 ± 1739	1.157			
Tropine	0.122 ± 0.001	28828 ± 823	0.807			
Tropisetron	0.107 ± 0.003	39042 ± 6634	1.093			
Tropisetron Hydrochloride	0.116 ± 0.006	25054 ± 1693	0.701			
Trospium chloride	0.119 ± 0.004	28981 ± 2185	0.811			
Troxerutin	0.119 ± 0.016	41076 ± 2239	1.150			
Troxipide	0.121 ± 0.009	27185 ± 3922	0.761			
Tryptamine	0.101 ± 0.008	32524 ± 1010	0.911			
Tryptamine hydrochloride	0.110 ± 0.009	38039 ± 4665	1.065			
Tryptanthrin	0.101 ± 0.001	32484 ± 608	0.910			
Tryptoline	0.124 ± 0.005	34227 ± 1532	0.958			
Tryptophan	0.100 ± 0.001	19727 ± 887	0.568			
Tryptophol	0.109 ± 0.002	39605 ± 4303	1.109			
Tschimganidin	0.102 ± 0.005	24738 ± 772	0.693			
Tschimganine	0.101 ± 0.002	25014 ± 413	0.700			
Ttetroquinone	0.102 ± 0.002	41946 ± 2448	1.174			
Ttnpb	0.106 ± 0.014	37687 ± 2056	1.055			
TTorasemide	0.125 ± 0.004	18286 ± 508	0.509			
Tuaminoheptane sulfate	0.098 ± 0.001	31401 ± 4624	0.879			
Tubeimoside A	0.108 ± 0.001	24398 ± 1250	0.683			
Tubercidin	0.107 ± 0.003	25281 ± 354	0.708			
Tubocurarine chloride	0.105 ± 0.002	40557 ± 2653	1.136			
Tulobuterol	0.110 ± 0.004	35763 ± 4297	1.001			
Tulobuterol hydrochloride	0.111 ± 0.006	34058 ± 3300	0.954			
Tunicamycin	0.114 ± 0.004	21614 ± 3083	0.605			
Tunicamycin B	0.110 ± 0.003	25571 ± 298	0.716			
Tupichilignan A	0.118 ± 0.003	38692 ± 2433	1.083			
Turraeanthin	0.112 ± 0.004	28672 ± 2491	0.803			

Tutin	0.126 ± 0.005	29696 ± 596	0.831			
TX-1918	0.056 ± 0.002 **					
Tylosin	0.087 ± 0.005 **					
Tylosin tartrate	0.100 ± 0.003	2989 ± 192	0.085	0.109 ± 0.003	10079 ± 1618	0.308
Tyloxapol	0.136 ± 0.014	27930 ± 4576	0.782			
Tyramine	0.107 ± 0.012	34892 ± 1184	0.977			
Tyromycic acid	0.120 ± 0.003	30410 ± 1799	0.851			
Tyrosine	0.113 ± 0.009	31157 ± 1275	0.872			
Tyrothricin	0.051 ± 0.002 **					
Tyrphostin 1	0.121 ± 0.001	34579 ± 4815	0.968			
Tyrphostin 23	0.117 ± 0.002	23718 ± 2196	0.664			
Tyrphostin 25	0.118 ± 0.001	25257 ± 670	0.707			
Tyrphostin 46	0.112 ± 0.002	30084 ± 5716	0.842			
Tyrphostin 47	0.115 ± 0.002	22426 ± 3084	0.628			
Tyrphostin 51	0.122 ± 0.002	34119 ± 1277	0.955			
Tyrphostin 8	0.110 ± 0.003	26506 ± 1899	0.742			
Tyrphostin 9	0.124 ± 0.004	29873 ± 3112	0.836			
Tyrphostin A9	0.109 ± 0.008	32928 ± 364	0.922			
Tyrphostin AG 112	0.112 ± 0.010	40288 ± 2538	1.128			
Tyrphostin AG 126	0.110 ± 0.011	53808 ± 4259	1.487			
Tyrphostin AG 1288	0.110 ± 0.001	43186 ± 3170	1.209			
Tyrphostin AG 1295	0.120 ± 0.007	35836 ± 2044	1.003			
Tyrphostin AG 1478	0.082 ± 0.001 **					
Tyrphostin AG 34	0.101 ± 0.001	50212 ± 3216	1.388			
Tyrphostin AG 490	0.102 ± 0.007	46678 ± 2598	1.307			
Tyrphostin AG 494	0.095 ± 0.005	10255 ± 1730	0.283	0.096 ± 0.009	27355 ± 4246	0.837
Tyrphostin AG 527	0.104 ± 0.002	43306 ± 787	1.213			
Tyrphostin AG 537	0.116 ± 0.007	36024 ± 3050	1.009			
Tyrphostin AG 538	0.109 ± 0.003	39120 ± 4577	1.095			
Tyrphostin AG 555	0.113 ± 0.013	63451 ± 3760	1.754			
Tyrphostin AG 698	0.106 ± 0.015	42815 ± 715	1.199			
Tyrphostin AG 808	0.123 ± 0.010	35912 ± 5162	1.006			

Tyrphostin AG 835	0.105 ± 0.009	58947 ± 4777	1.629			
Tyrphostin AG 879	0.104 ± 0.007	51453 ± 5552	1.422			
Tyrphostin AG-126	0.112 ± 0.003	34293 ± 2378	0.960			
Tyrphostin AG-825	0.115 ± 0.005	13373 ± 1568	0.406	0.114 ± 0.007	5928 ± 1231	0.181
Tyrphostin B44, (+)-enantiomer	0.104 ± 0.005	26314 ± 1887	0.737			
Tyrphostin-8	0.117 ± 0.004	60198 ± 4373	1.829			
U-0124	0.110 ± 0.002	33841 ± 3895	0.948			
U-0126	0.097 ± 0.015	30377 ± 1771	0.851			
U-101958 Maleate	0.112 ± 0.014	34357 ± 1569	0.962			
U-10293	0.114 ± 0.014	36984 ± 1315	1.036			
U-18666A	0.118 ± 0.011	43583 ± 5664	1.220			
U-37883A	0.110 ± 0.002	35558 ± 1119	0.996			
U-46619	0.104 ± 0.008	27791 ± 2799	0.778			
U-50488	0.109 ± 0.002	38073 ± 1738	1.066			
U-50-488H Mesylate	0.098 ± 0.012	32248 ± 2868	0.903			
U-54494A	0.106 ± 0.008	28216 ± 5726	0.790			
U-54494A Hydrochloride	0.105 ± 0.010	45745 ± 1661	1.281			
U-62066	0.112 ± 0.011	31829 ± 1486	0.891			
U-69593	0.103 ± 0.005	28821 ± 2231	0.807			
U-73122	0.110 ± 0.015	29367 ± 2144	0.822			
U-73343	0.100 ± 0.002	40642 ± 1342	1.138			
U-74389G	0.105 ± 0.008	125243 ± 3458	3.806	0.112 ± 0.005	61946 ± 4680	1.896
U-74389G Maleate	0.111 ± 0.006	27062 ± 1699	0.758			
U-75302	0.113 ± 0.008	36617 ± 1029	1.025			
U-83836E	0.067 ± 0.003 **					
U-99194 Maleate	0.115 ± 0.001	43631 ± 1306	1.222			
U-99194A Maleate	0.102 ± 0.006	32669 ± 1106	0.915			
UB 165 Fumarate	0.115 ± 0.003	46455 ± 2600	1.301			
Ubidecareneone	0.120 ± 0.004	37222 ± 3151	1.042			
UBP 141	0.111 ± 0.005	31288 ± 2151	0.876			
UBP 296	0.108 ± 0.009	34997 ± 5510	0.980			
UBP 302	0.112 ± 0.005	38539 ± 5193	1.079			

UBP 304	0.114 ± 0.004	31428 ± 1337	0.880			
UBP 308	0.116 ± 0.002	39135 ± 6173	1.096			
UCH-L1 Inhibitor	0.102 ± 0.004	58968 ± 3341	1.725			
UCH-L1 Inhibitor II	0.095 ± 0.002	47874 ± 5570	1.340			
UCH-L3 Inhibitor	0.102 ± 0.004	54265 ± 8364	1.519			
UCL 2077	0.102 ± 0.005	37295 ± 1154	1.044			
UCN-01	0.106 ± 0.005	36493 ± 4688	1.022			
UK 14304	0.104 ± 0.005	27610 ± 1393	0.773			
UK 14304 Tartrate	0.105 ± 0.007	28928 ± 2512	0.810			
Umbelliferone	0.126 ± 0.002	35420 ± 1705	0.992			
Uncargenin C	0.127 ± 0.008	36068 ± 2129	1.010			
Uncaric acid	0.133 ± 0.012	20066 ± 1165	0.562			
Undecanoic acid	0.104 ± 0.004	39301 ± 6043	1.100			
Undecylenic acid	0.112 ± 0.008	31868 ± 3552	0.892			
Ungerine nitrate	0.094 ± 0.003 *					
UPF 1069	0.116 ± 0.007	34164 ± 2734	0.957			
UPF 523	0.107 ± 0.007	28154 ± 3718	0.788			
Uracil	0.126 ± 0.007	41285 ± 4949	1.156			
Urapidil hydrochloride	0.104 ± 0.007	63371 ± 4398	1.695			
Urapidil, 5-Methyl-	0.113 ± 0.009	36764 ± 391	1.029			
URB-597	0.115 ± 0.003	43498 ± 1079	1.343			
Urea	0.104 ± 0.001	38339 ± 4805	1.073			
Urethane	0.098 ± 0.002	25645 ± 1764	0.718			
Uridine	0.126 ± 0.004	41353 ± 3053	1.158			
Uridine 5'-diphosphate sodium	0.108 ± 0.005	35475 ± 2224	0.993			
Uridine triphosphate trisodium	0.099 ± 0.010	30374 ± 3252	0.850			
Uridine-5'-monophosphate Na	0.104 ± 0.012	43021 ± 656	1.205			
Uridine-5'-triphosphate Na	0.105 ± 0.006	43431 ± 2639	1.216			
Urosiol	0.111 ± 0.003	36394 ± 4306	1.019			
Ursocholanic acid	0.110 ± 0.007	39205 ± 4941	1.098			
Ursodiol	0.099 ± 0.004	28056 ± 8176	0.786			
Ursolic acid	0.106 ± 0.004	41758 ± 629	1.169			

Ursonic acid	0.122 ± 0.002	35412 ± 2294	0.992			
Usnic acid, (+)-	0.103 ± 0.003	20108 ± 997	0.597			
Utilin	0.107 ± 0.008	27569 ± 1544	0.772			
Uvaol	0.105 ± 0.007	25748 ± 125	0.721			
Uvinul-D-49	0.108 ± 0.013	39369 ± 2127	1.102			
Uzarigenin	0.118 ± 0.005	38599 ± 3832	1.081			
Uzarigenin digitaloside	0.121 ± 0.006	42524 ± 3187	1.191			
Vacuolin-1	0.095 ± 0.003	48077 ± 991	1.346			
Valaciclovir	0.115 ± 0.002	31314 ± 1591	0.877			
Valacyclovir hydrochloride	0.114 ± 0.002	46036 ± 2389	1.289			
Valdecoxib	0.127 ± 0.009	33120 ± 2856	0.927			
Valeryl salycilate	0.104 ± 0.009	29161 ± 1673	0.816			
Valganciclovir hydrochloride	0.107 ± 0.009	33987 ± 6412	0.952			
Valinomycin	0.117 ± 0.004	39099 ± 920	1.095			
Vallesamine N-oxide	0.128 ± 0.003	45177 ± 3462	1.265			
Valproate sodium	0.104 ± 0.013	31986 ± 1059	0.896			
Valproic acid	0.109 ± 0.010	42467 ± 3452	1.189			
Valproxam	0.105 ± 0.015	29099 ± 2054	0.815			
Valsartan	0.117 ± 0.004	41826 ± 7031	1.171			
Vancomycin hydrochloride	0.056 ± 0.001 **					
Vandetanib	0.119 ± 0.001	35718 ± 1083	1.000			
Vandrikidine	0.121 ± 0.002	36648 ± 2318	1.026			
Vanillic acid	0.109 ± 0.004	29363 ± 1371	0.822			
Vanillic acid diethylamide	0.103 ± 0.004	35405 ± 1867	0.991			
Vanillylacetone	0.101 ± 0.003	46364 ± 2201	1.377			
Vardenafil	0.114 ± 0.006	41921 ± 2991	1.174			
Vardenafil citrate	0.115 ± 0.002	69269 ± 4440	1.929			
Vardenafil hydrochloride	0.110 ± 0.009	40036 ± 5168	1.121			
Vargatef	0.123 ± 0.002	35453 ± 2301	0.993			
Vasicine	0.118 ± 0.002	45447 ± 2732	1.272			
Vasicinol	0.111 ± 0.007	29776 ± 1716	0.834			
Vasicinolone	0.113 ± 0.006	43383 ± 1542	1.215			

Vasicinone	0.123 ± 0.002	36652 ± 1027	1.026			
Vatalanib	0.086 ± 0.003 **					
Vecuronium bromide	0.102 ± 0.016	37253 ± 1703	1.043			
VEGF Inducer, GS4012	0.101 ± 0.004	49573 ± 1129	1.388			
VEGF Receptor 2 Kinase Inhibitor I	0.128 ± 0.011	22694 ± 2444	0.635			
VEGF Receptor 2 Kinase Inhibitor II	0.127 ± 0.006	21302 ± 1667	0.596			
VEGF Receptor 2 Kinase Inhibitor III	0.115 ± 0.007	29362 ± 1944	0.822			
VEGF Receptor 2 Kinase Inhibitor IV	0.123 ± 0.006	24223 ± 537	0.678			
VEGFR Tyrosine Kinase Inhibitor IV	0.117 ± 0.017	27726 ± 2508	0.776			
VEGFR2/Flt3/c-Kit Inhibitor	0.091 ± 0.003 *					
Velutin	0.129 ± 0.002	32235 ± 2572	0.903			
Venlafaxine	0.111 ± 0.003	36091 ± 606	1.011			
Venlafaxine hydrochloride	0.104 ± 0.010	28719 ± 726	0.804			
VER-3323 hemifumarate salt	0.103 ± 0.004	25682 ± 1024	0.719			
Verapamil	0.110 ± 0.004	45115 ± 4503	1.263			
Verapamil Hydrochloride	0.111 ± 0.003	35308 ± 2799	0.989			
Veratraldehyde	0.118 ± 0.002	38153 ± 864	1.068			
Veratramine	0.112 ± 0.006	33445 ± 681	0.936			
Veratric acid	0.104 ± 0.009	30159 ± 2018	0.844			
Veratridine	0.107 ± 0.003	34437 ± 1735	0.964			
Veratrine sulfate	0.106 ± 0.007	36062 ± 1642	1.010			
Verbenalin	0.102 ± 0.014	33088 ± 2310	0.926			
Verminoside	0.122 ± 0.003	36425 ± 2122	1.020			
Verteporfin	0.112 ± 0.008	23880 ± 2481	0.669			
Vestitol	0.108 ± 0.004	53087 ± 5949	1.486			
VGX-1027	0.096 ± 0.002	31606 ± 3086	0.885			
Vibo-Quercitol	0.124 ± 0.005	34960 ± 3816	0.979			
Vidarabine	0.089 ± 0.007 **					
Vigabatrin	0.102 ± 0.013	35963 ± 720	1.007			
Vilazodone hydrochloride	0.096 ± 0.008	45954 ± 7399	1.287			
Villosin	0.128 ± 0.003	32856 ± 1177	0.920			
Villosin C	0.085 ± 0.003 **					

Viloxazine hydrochloride	0.118 ± 0.008	45615 ± 2373	1.277			
Vinblastine	0.107 ± 0.002	25462 ± 3506	0.713			
Vinblastine sulfate	0.116 ± 0.008	35857 ± 1500	1.004			
Vincamine	0.120 ± 0.004	27837 ± 561	0.779			
Vincosamide	0.110 ± 0.006	44190 ± 4204	1.237			
Vincristine sulfate	0.107 ± 0.005	28124 ± 1091	0.787			
Vindesine	0.117 ± 0.003	32640 ± 2635	0.914			
Vindesine sulfate	0.108 ± 0.002	39693 ± 2608	1.111			
Vindoline	0.122 ± 0.006	32427 ± 1596	0.908			
Vindorosine	0.117 ± 0.008	27142 ± 890	0.760			
Vineomycin A1	0.099 ± 0.002 *					
Vineridine	0.119 ± 0.002	42431 ± 1444	1.188			
Viniferin	0.117 ± 0.007	42449 ± 954	1.189			
Viniferol D	0.117 ± 0.017	45089 ± 2899	1.262			
Vinorelbine	0.105 ± 0.002	67267 ± 5589	1.913			
Vinorelbine base	0.119 ± 0.006	25758 ± 2666	0.721			
Vinorelbine bitatrate	0.138 ± 0.002 **					
Vinpocetine	0.114 ± 0.009	27808 ± 2522	0.779			
Violastyrene	0.105 ± 0.004	43288 ± 1015	1.212			
Viomycin sulfate	0.128 ± 0.014	40031 ± 5274	1.121			
VIP(6-28)	0.119 ± 0.003	21831 ± 2053	0.611			
Viramune	0.127 ± 0.004	38948 ± 3464	1.091			
Viscidulin I	0.135 ± 0.007	33230 ± 1362	0.930			
Viscidulin III	0.109 ± 0.006	32138 ± 1193	0.900			
Viscidulin III tetraacetate	0.125 ± 0.003	19784 ± 1449	0.553			
Visnagin	0.105 ± 0.002	47823 ± 1014	1.420			
Vitamin a (acetate)	0.117 ± 0.006	24839 ± 338	0.695			
Viteralone	0.106 ± 0.012	37168 ± 1621	1.041			
Vitexilactone	0.117 ± 0.009	37460 ± 2553	1.049			
Vitexin	0.104 ± 0.001	20844 ± 179	0.619			
Vitexin-2"-O-rhamnoside	0.102 ± 0.021	17024 ± 1325	0.506			
Voglibose	0.100 ± 0.012	32990 ± 2754	0.924			

Voleneol	0.123 ± 0.005	38394 ± 2620	1.075			
Vomifoliol	0.123 ± 0.002	27434 ± 915	0.768			
Voriconazole	0.126 ± 0.001	36498 ± 2959	1.022			
Vorinostat	0.104 ± 0.021	33793 ± 7632	0.946			
VU 0155069	0.097 ± 0.001 *					
VU 0238429	0.115 ± 0.016	31095 ± 242	0.871			
VU 0361737	0.115 ± 0.004	31843 ± 1391	0.892			
Vulpinic acid	0.099 ± 0.002	43747 ± 1080	1.225			
VX-680	0.123 ± 0.006	36057 ± 2028	1.010			
VX-702	0.127 ± 0.008	32284 ± 2373	0.904			
W7	0.121 ± 0.004	22208 ± 2799	0.622			
Walsuronoid B	0.126 ± 0.002	38097 ± 2298	1.067			
Warfarin	0.099 ± 0.012	18829 ± 3509	0.527			
WAY 100635 Maleate salt	0.106 ± 0.007	26616 ± 5188	0.745			
WAY 161503 Hydrochloride	0.111 ± 0.005	33275 ± 409	0.932			
WAY 170523	0.111 ± 0.002	45582 ± 2057	1.276			
WAY 200070	0.112 ± 0.004	37642 ± 1530	1.054			
WAY 213613	0.115 ± 0.019	30574 ± 2170	0.856			
WAY 629 Hydrochloride	0.109 ± 0.012	32582 ± 3463	0.912			
WB 4101 Hydrochloride	0.109 ± 0.012	30884 ± 5582	0.865			
WB 64	0.106 ± 0.005	8183 ± 204	0.226	0.121 ± 0.005	6691 ± 617	0.205
WEB 2086	0.104 ± 0.008	27563 ± 5305	0.772			
Wedelolactone	0.068 ± 0.006 **					
Wee1 Inhibitor	0.114 ± 0.011	50666 ± 6416	1.419			
Wee1 Inhibitor II	0.120 ± 0.015	30242 ± 1971	0.847			
Wee1/Chk1 Inhibitor	0.136 ± 0.007	27031 ± 2725	0.757			
WHI-P180, Hydrochloride	0.103 ± 0.012	20565 ± 1796	0.576			
Wighteone	0.132 ± 0.008	44505 ± 4264	1.246			
Wilforgine	0.121 ± 0.001	42554 ± 3521	1.191			
Wilforine	0.129 ± 0.005	37623 ± 2200	1.053			
Wilforlide A	0.128 ± 0.005	31372 ± 1239	0.878			
Wilforlide A acetate	0.132 ± 0.020	39745 ± 3007	1.113			

Wilforol C	0.116 ± 0.001	36785 ± 2230	1.030			
Wilsonine	0.123 ± 0.005	36013 ± 525	1.008			
WIN 55212-2	0.104 ± 0.007	35487 ± 1016	0.994			
WIN 62577	0.112 ± 0.010	43547 ± 2600	1.219			
WIN 64338 Hydrochloride	0.060 ± 0.004 **					
Wiskostatin	0.110 ± 0.003	17169 ± 361	0.522			
Withaferin A, Withania somnifera	0.099 ± 0.004	17213 ± 1395	0.504			
Wnt Agonist	0.112 ± 0.008	47884 ± 2558	1.341			
Wnt Antagonist I, IWR-1-endo	0.105 ± 0.006	52609 ± 3548	1.473			
Wogonin	0.105 ± 0.003	53599 ± 3752	1.592			
Wortmannin	0.108 ± 0.002	22208 ± 1183	0.622			
Wortmannin from Penicillium funiculosum	0.111 ± 0.008	20822 ± 1103	0.576			
WY 14643	0.113 ± 0.004	29896 ± 917	0.837			
Xaliproden hydrochloride	0.100 ± 0.002	34458 ± 1336	0.965			
Xamoterol hemifumarate	0.109 ± 0.007	41745 ± 5094	1.169			
Xanthatin	0.128 ± 0.015	32960 ± 3491	0.923			
Xanthine amine congener	0.109 ± 0.010	29478 ± 1537	0.825			
Xanthinin	0.130 ± 0.009	25227 ± 2042	0.706			
Xanthinol nicotinate	0.114 ± 0.003	42680 ± 7351	1.195			
Xanthobine	0.110 ± 0.008	42227 ± 3693	1.182			
Xanthohumol	0.123 ± 0.004	34909 ± 2712	0.977			
Xanthohumol D	0.119 ± 0.002	50565 ± 7077	1.416			
Xanthone	0.115 ± 0.005	36806 ± 4903	1.031			
Xanthoplanine	0.114 ± 0.002	45356 ± 1325	1.270			
Xanthopterin	0.114 ± 0.005	40112 ± 3925	1.123			
Xanthorin	0.113 ± 0.004	40369 ± 5331	1.130			
Xanthotoxin	0.119 ± 0.006	65414 ± 3456	1.943			
Xanthotoxol	0.117 ± 0.004	8463 ± 1025	0.237	0.113 ± 0.011	16556 ± 1820	0.507
Xanthoxylin	0.134 ± 0.002	33859 ± 2682	0.948			
Xanthurenic acid	0.116 ± 0.006	30552 ± 1684	0.855			
Xanthyletin	0.102 ± 0.006	20503 ± 4520	0.574			
XAV 939	0.134 ± 0.008 *					

XC-386	0.109 ± 0.006	48349 ± 1217	1.354			
XCT790	0.110 ± 0.011	33134 ± 2122	0.928			
XE 991 dihydrochloride	0.106 ± 0.005	22701 ± 2393	0.636			
Xerophilusin G	0.088 ± 0.002 **					
Xestospongin C	0.109 ± 0.002	20760 ± 196	0.581			
XK469	0.110 ± 0.012	34772 ± 2158	0.974			
Xylazine	0.106 ± 0.005	37169 ± 1096	1.041			
Xylazine hydrochloride	0.110 ± 0.006	32351 ± 4114	0.906			
Xylocarpus a	0.111 ± 0.008	28346 ± 1865	0.794			
Xylometazoline hydrochloride	0.113 ± 0.013	19481 ± 634	0.538			
Y 134	0.111 ± 0.006	34197 ± 236	0.957			
Y 29794 oxalate	0.065 ± 0.005 **					
Y-25130	0.112 ± 0.001	24466 ± 2701	0.685			
Y-26763	0.119 ± 0.005	29878 ± 3021	0.837			
Y-27152	0.133 ± 0.001 **					
Y-27632	0.122 ± 0.006	31400 ± 2291	0.879			
Yangonin	0.122 ± 0.002	54401 ± 4419	1.616			
Yatein	0.122 ± 0.003	33399 ± 3684	0.935			
YC-1	0.117 ± 0.012	13741 ± 1012	0.380	0.119 ± 0.004	22752 ± 3708	0.696
YM 138552	0.110 ± 0.018	24023 ± 4728	0.673			
YM 201636	0.123 ± 0.006	39600 ± 903	1.109			
YM 298198 Hydrochloride	0.113 ± 0.006	32167 ± 3015	0.901			
YM 298198, Desmethyl-	0.103 ± 0.006	28712 ± 2959	0.804			
YM 90709	0.110 ± 0.004	39322 ± 2195	1.101			
YM 976	0.099 ± 0.002	36154 ± 1039	1.012			
Yohimbine	0.110 ± 0.003	28968 ± 1529	0.811			
Yohimbine hydrochloride	0.121 ± 0.009	50611 ± 5375	1.417			
YS035	0.109 ± 0.002	36602 ± 1547	1.025			
Yuheinoside	0.105 ± 0.002	43192 ± 4637	1.209			
Yunnancoronarin A	0.118 ± 0.005	34937 ± 2137	0.978			
Yunnandaphninine G	0.114 ± 0.001	37511 ± 1015	1.050			
Zacopride	0.123 ± 0.007	50453 ± 2962	1.413			

Zacopride hydrochloride	0.100 ± 0.008	38889 ± 1790	1.089			
Zafirlukast	0.131 ± 0.012	54094 ± 9604	1.515			
Zalcitabine	0.124 ± 0.014	35696 ± 2281	0.999			
Zaleplon	0.121 ± 0.002	22834 ± 1244	0.639			
Zamanic acid	0.125 ± 0.005	39375 ± 5845	1.102			
Zamifenacin fumarate	0.113 ± 0.005	42297 ± 3020	1.184			
ZAPA H_2SO_4	0.105 ± 0.004	27218 ± 2247	0.762			
Zapoterin	0.120 ± 0.005	41033 ± 2370	1.149			
Zaprinast	0.111 ± 0.003	32675 ± 4715	0.915			
Zardaverine	0.108 ± 0.002	23065 ± 1466	0.646			
Zatebradine hydrochloride	0.114 ± 0.002	32124 ± 4794	0.899			
ZD 2079 hydrochloride	0.112 ± 0.001	51299 ± 8905	1.436			
ZD 7114 hydrochloride	0.116 ± 0.009	28106 ± 3900	0.787			
ZD 7155 hydrochloride	0.112 ± 0.006	31388 ± 2690	0.879			
ZD7288	0.107 ± 0.004	41199 ± 3870	1.154			
Zearalanol	0.107 ± 0.001	43534 ± 383	1.219			
Zearalenone	0.104 ± 0.005	35318 ± 778	0.989			
Zebularine	0.097 ± 0.009	37711 ± 1382	1.056			
Zeorin	0.131 ± 0.005	37554 ± 2867	1.051			
Zeranol	0.112 ± 0.005	59774 ± 6499	1.674			
Z-FA-FMK	0.095 ± 0.004 *					
Z-Guggulsterone	0.101 ± 0.004	44150 ± 2275	1.236			
Zidovudine	0.101 ± 0.004	30700 ± 2737	0.860			
Zileuton	0.127 ± 0.007	25549 ± 404	0.715			
Zilpaterol	0.115 ± 0.006	52180 ± 1441	1.395			
Zimelidine dihydrochloride monohydrate	0.117 ± 0.004	45622 ± 1213	1.277			
Zinc undecylenate	0.103 ± 0.003	29613 ± 5421	0.829			
Ziprasidone hydrochloride monohydrate	0.107 ± 0.002	41367 ± 1850	1.158			
Ziprasidone mesylate	0.112 ± 0.011	21106 ± 1432	0.591			
Ziyuglycoside I	0.122 ± 0.007	34383 ± 1729	0.963			
Ziyuglycoside II	0.121 ± 0.002	36597 ± 3129	1.025			
ZK 164015	0.110 ± 0.002	43464 ± 6479	1.217			

ZK 200775	0.105 ± 0.003	34059 ± 3798	0.954			
ZK 93423	0.112 ± 0.002	24411 ± 973	0.683			
ZK 93426 hydrochloride	0.101 ± 0.009	19089 ± 1886	0.528			
Z-Leu3-VS	0.127 ± 0.007	27460 ± 880	0.769			
Z-Leu-Leu-CHO	0.111 ± 0.002	32979 ± 1137	0.923			
Z-L-Phe chloromethyl ketone	0.100 ± 0.009	18807 ± 813	0.520			
ZM 226600	0.111 ± 0.005	35989 ± 2287	1.008			
ZM 241385	0.100 ± 0.006	44532 ± 3296	1.247			
ZM 306416 hydrochloride	0.113 ± 0.004	39717 ± 2391	1.112			
ZM 323881 hydrochloride	0.108 ± 0.001	40603 ± 1895	1.137			
ZM 336372	0.122 ± 0.006	37016 ± 4102	1.036			
ZM 39923 hydrochloride	0.109 ± 0.009	65699 ± 5759	1.816			
ZM 447439	0.108 ± 0.006	29728 ± 2517	0.832			
ZM 449829	0.073 ± 0.004 **					
Zolantidine dimaleate	0.089 ± 0.007 *					
Zoledronic acid	0.125 ± 0.012	27544 ± 1586	0.771			
Zolmitriptan	0.125 ± 0.004	44722 ± 1590	1.252			
Zolpidem	0.109 ± 0.012	24101 ± 2286	0.675			
Zolpidem tartrate	0.118 ± 0.005	39050 ± 2450	1.093			
Zomepirac sodium	0.108 ± 0.007	21704 ± 2887	0.608			
Zonisamide	0.107 ± 0.008	28769 ± 2683	0.806			
Zonisamide sodium	0.109 ± 0.008	30450 ± 1038	0.853			
Zopiclone	0.109 ± 0.007	32598 ± 1968	0.913			
Zotepine	0.111 ± 0.005	44538 ± 3041	1.247			
Zoxazolamine	0.110 ± 0.006	49802 ± 4771	1.394			
Z-PLG-NHOH	0.119 ± 0.008	29385 ± 1017	0.823			
Z-Prolyl-Prolinal	0.098 ± 0.012	13001 ± 356	0.402	0.105 ± 0.004	24846 ± 3579	0.760
Zuclopenthixol hydrochloride	0.124 ± 0.005	31315 ± 3198	0.877			
Z-VAD-FMK	0.108 ± 0.002	51045 ± 1367	1.576			
α-Acetyldigoxin	0.105 ± 0.002	34699 ± 1757	0.972			
α-Amyrin acetate	0.119 ± 0.007	48707 ± 5016	1.364			
α-Amyrin palmitate	0.114 ± 0.004	41419 ± 4358	1.160			

α-Asarone	0.103 ± 0.007	41421 ± 2744	1.160			
α-Boswellic acid	0.117 ± 0.005	41713 ± 3349	1.168			
α-Cannabispiranol	0.136 ± 0.004	32362 ± 3627	0.906			
α-Conidendrin	0.125 ± 0.003	43793 ± 2364	1.226			
α-Cyano-3-hydroxycinnamic acid	0.103 ± 0.004	32491 ± 595	0.910			
α-Cyano-4-hydroxycinnamic acid	0.102 ± 0.002	35117 ± 546	0.983			
α-Epoxydihydroartemisinic acid	0.124 ± 0.007	30414 ± 2320	0.852			
α-Guanidinoglutaric acid	0.105 ± 0.009	49476 ± 3652	1.385			
α-Hederin	0.122 ± 0.006	34129 ± 3261	0.956			
α-Hydroxydeoxycholic acid	0.105 ± 0.005	29656 ± 1775	0.830			
α-Isowighteone	0.118 ± 0.004	56287 ± 2996	1.573			
α-Lapachone	0.119 ± 0.003	34243 ± 2134	0.959			
α-Lipoic acid (DL)	0.098 ± 0.006	18292 ± 1228	0.511			
α-Lobeline hydrochloride	0.101 ± 0.004	36394 ± 2975	1.019			
α-Mangostin	0.113 ± 0.005	41779 ± 4206	1.170			
α-Methyl-5-hydroxytryptamine maleate	0.107 ± 0.013	33891 ± 2982	0.949			
α-Methyl-DL-tyrosine methyl ester hydrochloride	0.104 ± 0.007	28679 ± 2711	0.803			
α-Methyltryptamine	0.111 ± 0.003	31038 ± 3293	0.869			
α-MSH(free acid)	0.109 ± 0.002	20913 ± 1166	0.586			
α-Neoendorphin	0.107 ± 0.008	42584 ± 3399	1.192			
α-Onocerol	0.130 ± 0.010	52273 ± 4100	1.464			
α-Spinasterol	0.124 ± 0.003	33103 ± 1354	0.927			
α-Spinasterol acetate	0.129 ± 0.009	34042 ± 628	0.953			
α-Spinasterol glucoside	0.136 ± 0.004	40347 ± 3595	1.130			
α-Spinasterol methylthiomethyl ether	0.117 ± 0.003	39123 ± 3068	1.095			
α-Spinasterone	0.124 ± 0.002	35665 ± 2571	0.999			
α-Terthienylmethanol	0.117 ± 0.005	38948 ± 1624	1.091			
α-Tochopherol	0.099 ± 0.008	27663 ± 4549	0.775			
α-Tochopheryl acetate	0.097 ± 0.005	29084 ± 8957	0.814			
α-Tocopherol acetate	0.114 ± 0.006	38924 ± 3888	1.090			
α-Tocopherolquinone	0.133 ± 0.005	34018 ± 2801	0.952			
α-Toxicarol	0.107 ± 0.001	32850 ± 743	0.920			

α-Viniferin	0.078 ± 0.008 **					
α-Xalone	0.114 ± 0.001	40140 ± 6118	1.124			
α-Yohimbine	0.129 ± 0.002	37264 ± 3301	1.043			
β-Amyrenonol	0.123 ± 0.012	43051 ± 3476	1.205			
β-Amyrenonol acetate	0.133 ± 0.001	38029 ± 1850	1.065			
β-Amyrenonol methylthiomethyl ether	0.124 ± 0.001	49007 ± 5116	1.372			
β-Amyrin	0.106 ± 0.014	37898 ± 2475	1.061			
β-Amyrin acetate	0.106 ± 0.009	37223 ± 2000	1.042			
β-Amyrin palmitate	0.124 ± 0.012	45896 ± 4271	1.285			
β-Amyrone	0.115 ± 0.006	32296 ± 1233	0.904			
β-Anhydrouzarigenin	0.121 ± 0.001	25670 ± 2226	0.719			
β-ARK1 inhibitor	0.107 ± 0.007	28514 ± 790	0.798			
β-Asarone	0.122 ± 0.004	43277 ± 2490	1.212			
β-Belladonnine dichloroethylate	0.110 ± 0.008	28016 ± 1231	0.784			
β-Carboline-1-propanoic acid	0.135 ± 0.008	39729 ± 1166	1.112			
β-Carotene	0.113 ± 0.005	33639 ± 1719	0.942			
β-Caryophyllene alcohol	0.108 ± 0.008	34962 ± 3339	0.979			
β-Catenin/Tcf Inhibitor, FH535	0.091 ± 0.004 *					
β-CCB	0.118 ± 0.007	32628 ± 1314	0.914			
β-Chloro-L-alanine hydrochloride	0.095 ± 0.016	54033 ± 4905	1.513			
β-Costic acid	0.114 ± 0.003	36200 ± 1932	1.014			
β-Dihydroplumericinic acid	0.120 ± 0.003	35255 ± 1700	0.987			
β-Escin	0.112 ± 0.003	55862 ± 4955	1.525			
β-Estradiol	0.098 ± 0.002	63396 ± 6891	1.752			
β-Funaltrexamine hydrochloride	0.103 ± 0.008	25874 ± 1016	0.724			
β-Hydroxypropiovanillone	0.104 ± 0.008	38093 ± 1452	1.067			
β-Lapachone	0.109 ± 0.002	38716 ± 1520	1.084			
β-Naphthol	0.089 ± 0.002 **					
β-Nicotyrine	0.113 ± 0.010	33026 ± 4944	0.925			
β-Peltatin	0.102 ± 0.008	39771 ± 451	1.114			
β-Phenylethylamine hydrochloride	0.108 ± 0.003	22410 ± 2127	0.627			
β-Secretase inhibitor IV	0.098 ± 0.011	43091 ± 2497	1.207			

β-Sitosterol	0.104 ± 0.008	33608 ± 2247	0.941			
β-Yohimbine	0.127 ± 0.011	32560 ± 2739	0.912			
γ-Acetylinic GABA	0.110 ± 0.003	39652 ± 2069	1.110			
γ-Aminobutyric acid	0.115 ± 0.004	38244 ± 1413	1.071			
γ-Diasarone	0.123 ± 0.006	53260 ± 4519	1.491			
γ-Fagarine	0.126 ± 0.009	41363 ± 990	1.158			
γ-Linolenic acid (18:3 n-6)	0.103 ± 0.004	30004 ± 1278	0.840			
γ-Mangostin	0.070 ± 0.005 **					
γ-Secretase inhibitor IX	0.098 ± 0.005	17838 ± 212	0.522			
γ-Secretase inhibitor VI	0.101 ± 0.004	22317 ± 616	0.625			
γ-Secretase inhibitor XXI, compound E	0.100 ± 0.002	41150 ± 680	1.152			
δ-12-Prostaglandin J2	0.117 ± 0.004	32832 ± 2096	0.919			
δ-1-Hydrocortisone 21-hemisuccinate sodium salt	0.101 ± 0.006	32030 ± 503	0.897			
δ-Amyrin acetate	0.133 ± 0.001	24959 ± 636	0.699			

[#]Lux activity represents the relative Lux activity (units/OD600). Data are the mean ± standard deviation of triplicate measurements. Asterisks indicate statistically significant differences, compared with the control (DMSO) (Student's *t* -test). **P* <0.05; ***P* <0.01.

Table S2 The effect of three known T3SS inhibitors on the lux activity of the reporter strain[b]

Name	Concentrations of the compounds used											
	5 µM		10 µM		20 µM		30 µM		60 µM		100 µM	
	OD_{600}	Lux activity	OD_{600}	Lux activity	OD_{600}	Lux activity	OD_{600}	Lux activity	OD_{600}	Lux activity	OD_{600}	Lux activity
DMSO	0.115 ± 0.002	35482 ± 3926	0.117 ± 0.004	38419 ± 4762	0.115 ± 0.008	39085 ± 3104	0.113 ± 0.003	34162 ± 3509	0.116 ± 0.005	39035 ± 3710	0.109 ± 0.004	38043 ± 5064
Benzoic acid	0.113 ± 0.005	34762 ± 4027	0.114 ± 0.002	31832 ± 3862	0.110 ± 0.002	33795 ± 4926	0.113 ± 0.006	39163 ± 2176	0.112 ± 0.002	37286 ± 2716	0.098 ± 0.001*	35419 ± 3157
4,4'-Thiobis [2-methyl-phenol]	0.117 ± 0.004	29006 ± 4326	0.119 ± 0.007	30974 ± 3926	0.115 ± 0.007	42083 ± 3316	0.106 ± 0.005	38470 ± 3986	0.100 ± 0.004*	34641 ± 5276	0.084 ± 0.005**	35270 ± 1879
Imidocarb	0.112 ± 0.005	5948 ± 715**	0.109 ± 0.005	5769 ± 742**	0.105 ± 0.004	5337 ± 682**	0.098 ± 0.004*	4905 ± 649**	0.071 ± 0.006**	3241 ± 437**	0.058 ± 0.003**	2170 ± 186**

[b]Lux activity represents the relative Lux activity (units/OD_{600}). Data are the mean ± standard deviation of triplicate measurements. Asterisks indicate statistically significant differences, compared with the control (DMSO) (Student's *t*-test). *$P<0.05$; **$P<0.01$. The experiment was repeated three times. The results presented are from a representative experiment, and similar results were obtained in all other independent experiments.

Table S3 The result of the third screen#

medium	Inducer candidates	OD_{600}	Lux activity	Lux activity ratio (compound/DMSO)
XVM2	DMSO	0.178 ± 0.011	2987 ± 419	
	S0693	0.183 ± 0.012	9766 ± 1059**	3.270
	Resveratrol	0.174 ± 0.012	8293 ± 583**	2.776
	Brassinin	0.166 ± 0.010	9285 ± 816**	3.108
	Splitomicin	0.173 ± 0.018	10842 ± 944**	3.630
	NP-014322	0.178 ± 0.007	5647 ± 935**	1.891
	NP-006485	0.098 ± 0.015**		
	HBED	0.167 ± 0.009	3149 ± 485	1.054
	BML-281	0.173 ± 0.012	8237 ± 836**	2.758
	Pentetic acid	0.177 ± 0.008	9576 ± 644**	3.206
	Azelaic acid	0.182 ± 0.009	4232 ± 682	1.417
	Theaflavin monogallates	0.175 ± 0.010	5089 ± 391**	1.704
XCM1	**Inhibitor candidates**			
	DMSO	0.154 ± 0.009	222938 ± 26569	
	Motesanib	0.151 ± 0.007	31756 ± 3618**	0.142
	15-Deoxy-D12,14-prostaglandin J2	0.149 ± 0.004	194833 ± 15087	0.874
	WB 64	0.142 ± 0.004	24582 ± 3672**	0.110
	Aristolochic acid A	0.141 ± 0.006	100716 ± 8429**	0.465
	Troleandomycin	0.119 ± 0.005**		
	Carmofur	0.147 ± 0.013	48125 ± 1960**	0.216
	5-Fluorocytosine	0.151 ± 0.005	72607 ± 6421**	0.326
	A-3	0.139 ± 0.008	30849 ± 4174**	0.138
	Tyrphostin AG-825	0.158 ± 0.017	171566 ± 16705	0.770
	NP-009807	0.148 ± 0.012	28493 ± 2719**	0.128
	NP-014585	0.118 ± 0.006**		
	Kongensin A	0.098 ± 0.003**		
	Liangshanin A	0.071 ± 0.016**		
	Kamebanin	0.154 ± 0.013	176449 ± 21840	0.791
	Trichorabdal A	0.078 ± 0.006**		
	Mitoxantrone 2HCl	0.148 ± 0.011	203584 ± 32273	0.913
	Tylosin tartrate	0.105 ± 0.010**		
	HMS3229O07	0.138 ± 0.013	68276 ± 8549**	0.306
	Erastin	0.142 ± 0.005	150742 ± 25864	0.676
	DHBP dibromide	0.084 ± 0.007**		
	Erythromycin ethylsuccinate	0.120 ± 0.012*		
	Thioctic acid	0.148 ± 0.010	54867 ± 7004**	0.246
	Erythromycin stearate	0.095 ± 0.023*		
	7-Deacetoxy-7-oxokhivorin	0.114 ± 0.008*		
	Imidocarb	0.137 ± 0.012	83705 ± 8134**	0.375

#Lux activity represents the relative Lux activity (units/OD600). Data are the mean ± standard deviation of triplicate measurements. Asterisks indicate statistically significant differences, compared with the control (DMSO) (Student's *t*-test). *P<0.05; **P<0.01. The experiment was repeated three times. The results presented are from a representative experiment, and similar results were obtained in all other independent experiments.

Table S4 The optimal dosage of the inhibitors and inducers#

Name	Concentration													
	5 μM		10 μM		20 μM		30 μM		40 μM		80 μM		120 μM	
	OD_{600}	Lux activity	OD_{600}	Lux activity	OD_{600}	Lux activity	OD_{600}	Lux activity	OD_{600}	Lux activity	OD_{600}	Lux activity	OD_{600}	Lux activity
DMSO	0.116 ± 0.006	35482 ± 2926	0.113 ± 0.005	38419 ± 2762	0.115 ± 0.010	39085 ± 3004	0.112 ± 0.003	34162 ± 3509	0.112 ± 0.006	40199 ± 4028	0.118 ± 0.007	42035 ± 3913	0.106 ± 0.007	45043 ± 5064
Splitomicin	0.114 ± 0.005	50426 ± 6459*	0.109 ± 0.007	78341 ± 6822**	0.108 ± 0.009	101355 ± 8436**	0.105 ± 0.010	112441 ± 11389**	0.107 ± 0.002	105661 ± 7963**	0.107 ± 0.008	76238 ± 6370**	0.097 ± 0.005*	38649 ± 3621
Pentetic acid	0.108 ± 0.004	78431 ± 6425**	0.109 ± 0.004	125488 ± 10776**	0.110 ± 0.008	135317 ± 8934**	0.109 ± 0.006	115486 ± 9625**	0.106 ± 0.005	98341 ± 10220**	0.101 ± 0.005*	89647 ± 8317**	0.084 ± 0.007**	33486 ± 5405
S0693	0.113 ± 0.005	40276 ± 6024	0.116 ± 0.003	52716 ±7465*	0.109 ± 0.009	84135 ± 4389**	0.110 ± 0.004	92640 ± 8963**	0.107 ± 0.004	94783 ± 7529**	0.105 ± 0.011	84317 ± 5384**	0.104 ± 0.013	90538 ± 8463**
Brassinin	0.118 ± 0.002	28993 ± 4725	0.119 ± 0.005	44389 ± 6126	0.114 ± 0.002	72684 ± 4382**	0.112 ± 0.005	86029 ± 7204**	0.114 ± 0.004	67813 ± 2733**	0.107 ± 0.006	80402 ± 2020**	0.097 ± 0.006*	34602 ± 5025
BML-281	0.115 ± 0.004	38348 ± 5302	0.120 ± 0.005	49235 ± 4059*	0.117 ± 0.004	74155 ± 9420**	0.110 ± 0.013	76270 ± 6429**	0.116 ± 0.003	75041 ± 5062*	0.104 ± 0.007	70831 ± 5707**	0.096 ± 0.009*	32106 ± 1099
Resveratrol	0.109 ± 0.010	32079 ± 1056	0.108 ± 0.005	50742 ± 7029*	0.113 ± 0.003	80341 ± 5927**	0.107 ± 0.004	86431 ± 5869**	0.104 ± 0.008	70234 ± 4385**	0.109 ± 0.002	78421 ± 5853**	0.113 ± 0.001	69544 ± 3836**
A-3	0.114 ± 0.001	10549 ± 794**	0.110 ± 0.006	6405 ± 831**	0.112 ± 0.009	6315 ± 870**	0.110 ± 0.005	6710 ± 1135**	0.108 ± 0.006	6421 ± 650**	0.105 ± 0.009	5842 ± 395**	0.101 ± 0.003*	5934 ± 726**
Thioctic acid	0.107 ± 0.007	33471 ± 1610	0.109 ± 0.006	23764 ± 3088*	0.113 ± 0.003	17514 ± 1033**	0.107 ± 0.012	11986 ± 2047**	0.104 ± 0.009	8049 ± 710**	0.101 ± 0.003*	5780 ± 926**	0.094 ± 0.002**	4001 ± 352**
Motesanib	0.119 ± 0.007	13857 ± 2142**	0.116 ± 0.006	8973 ± 850**	0.109 ± 0.006	3829 ± 406**	0.114 ± 0.005	3895 ± 207**	0.112 ± 0.004	4084 ± 751**	0.110 ± 0.001	3604 ± 249**	0.108 ± 0.009	3062 ± 541**
5-Fluorocytosine	0.116 ± 0.003	12716 ± 743**	0.113 ± 0.002	8699 ± 1025**	0.116 ± 0.004	5871 ± 305**	0.109 ± 0.005	6047 ± 814**	0.104 ± 0.012	4786 ± 734**	0.098 ± 0.006*	4082 ± 386**	0.075 ± 0.006**	3592 ± 502**
Carmofur	0.122 ± 0.008	4039 ± 517**	0.112 ± 0.005	3716 ± 580**	0.117 ± 0.006	3906 ± 827**	0.111 ± 0.002	4067 ± 203**	0.106 ± 0.008	4215 ± 592**	0.094 ± 0.008*	3579 ± 302**	0.069 ± 0.005**	2694 ± 317**
WB 64	0.114 ± 0.004	8920 ± 1129**	0.110 ± 0.006	6583 ± 712**	0.111 ± 0.003	4158 ± 392**	0.115 ± 0.006	4058 ± 659**	0.115 ± 0.001	4693 ± 580**	0.109 ± 0.005	3724 ± 695**	0.111 ± 0.003	3836 ± 341**
HMS3229O07	0.115 ± 0.011	10116 ± 1135**	0.113 ± 0.005	6420 ± 894**	0.113 ± 0.006	5097 ± 442**	0.115 ± 0.002	4928 ± 617**	0.107 ± 0.006	4628 ± 935**	0.109 ± 0.004	4816 ± 756**	0.097 ± 0.004*	4627 ± 512**
Aristolochic acid	0.114 ± 0.002	26340 ± 1209*	0.113 ± 0.005	12376 ± 1845**	0.110 ± 0.003	10296 ± 1940**	0.106 ± 0.006	8471 ± 854**	0.103 ± 0.009	8536 ± 931**	0.095 ± 0.009*	8076 ± 728**	0.090 ± 0.002**	7740 ± 450**
NP-009807	0.113 ± 0.005	6186 ± 805**	0.113 ± 0.002	6019 ± 495**	0.115 ± 0.005	5823 ± 464**	0.114 ± 0.008	5890 ± 614**	0.110 ± 0.015	5691 ± 324**	0.108 ± 0.006	4871 ± 204**	0.106 ± 0.007	4809 ± 316**

#Lux activity represents the relative Lux activity (units/OD_{600}). Data are the mean ± standard deviation of triplicate measurements. Asterisks indicate statistically significant differences, compared with the control (DMSO) (Student's *t*-test). *P<0.05; **P<0.01. The experiment was repeated three times. The results presented are from a representative experiment, and similar results were obtained in all other independent experiments.

Table S5 The source of the compounds used in this work

Name	Library screened from	Company purchased from	Application for
Splitomicin	Epigenetics library	Tokyo Chemical Industry	Antitumor agents
Pentetic acid	Prestwick chemical library	J&K Scientific	Chelating titrant
S0693	Cell signaling & Neuroscience library	Sigma	Unknown
Brassinin	Natural products library	Toronto Research Chemicals	Unknown
BML-281	Epigenetics library	Targetmol	Antitumor agents
Resveratrol	Natural products library	J&K Scientific	Antidiabetic agents, antitumor agents
A-3	ICCB known bioactives library	Toronto Research Chemicals	Antitumor agents
Thioctic acid	The Spectrum collection library	J&K Scientific	Antioxidant, coenzyme
Motesanib	Syn kinase inhibitors library	J&K Scientific	Antitumor agents, angiokinase inhibitor
5-Fluorocytosine	NIH clinical collection library	J&K Scientific	Antifungal drugs
Carmofur	NIH clinical collection library	Sigma	Antitumor agents
WB 64	Cell signaling & Neuroscience library	National compound resource center	Antimycobacterial agents
HMS3229O07	Protein kinase inhibitors library	Merck Millipore	Tyrosine kinase inhibitor
Aristolochic acid	Stem cell regulators library	Sigma	Nephrotoxicity
NP-009807	Analyticon discovery library	Analyticon discovery	Unknown
Imidocarb	?	J&K Scientific	Antioxidant

Table S6 Sequences of the 2139-bp DNA fragment containing the luciferase genes *luxAB*

5’AAAGGATCCACGCCAGAAATGGCTTAGGTCTTATCGTAATACCAACAAATAAGGAAATGTTATGAAA
TTTGGAAACTTCCTTCTCACTTATCAGCCACCTGAGCTATCTCAGACCGAAGTGATGAAGCGATTGG
TTAATCTGGGCAAAGCGTCTGAAGGTTGTGGCTTCGACACCGTTTGGTTGCTAGAGCACCACTTCA
CTGAATTTGGGTTGTTAGGGAATCCTTATGTTGCTGCCGCACACCTATTAGGTGCGACAGAAACGCT
CAACGTTGGCACTGCAGCTATCGTATTGCCGACTGCCCATCCGGTTCGACAAGCAGAAGACGTAAA
CCTACTGGATCAAATGTCAAAAGGACGATTCCGTTTTGGTATTTGTCGCGGTTTGTACGATAAAGAT
TTTCGTGTCTTTGGTACAGACATGGATAACAGCCGAGCCTTAATGGACTGTTGGTATGACTTGATGA
AAGAAGGCTTCAATGAAGGCTATATCGCGGCGGATAACGAACATATTAAGTTCCCGAAAATCCAACT
GAATCCATCGGCTTACACACAAGGTGGTGCTCCTGTTTATGTCGTCGCGGAGTCAGCATCAACGAC
AGAATGGGCTGCAGAGCGTGGCCTACCAATGATTCTAAGCTGGATCATCAACACTCACGAGAAGAA
AGCGCAGCTTGATCTTTACAACGAAGTCGCGACTGAACATGGCTACGATGTGACTAAGATTGACCA
CTGTTTGTCTTACATCACCTCCGTCGATCATGACTCAAATAGAGCCAAAGATATTTGCCGCAACTTCT
TGGGCCATTGGTACGACTCATACGTGAATGCCACCAAGATTTTTGACGACTCTGACCAAACAAAAG
GTTACGACTTCAATAAAGGTCAATGGCGTGATTTTGTGTTGAAAGGCCACAAAGACACCAATCGCC
GAATTGATTACAGCTACGAAATCAACCCAGTAGGGACGCCTGAAGAGTGTATCGCGATTATCCAGCA
AGATATTGATGCGACGGGTATTGACAATATTTGTTGTGGTTTTGAAGCAAACGGTTCTGAAGAAGAA
ATTATCGCATCTATGAAGCTATTCCAGTCTGATGTGATGCCATATCTCAAAGAAAAACAGTAATTAAT
ATTTTCTAAAAGGAAAGAGACATGAAATTTGGATTATTCTTCCTCAATTTTATGAACTCAAAGCGTTC
TTCTGATCAAGTCATCGAAGAAATGTTAGATACCGCACATTACGTAGATCAGTTGAAGTTTGACACG
TTGGCTGTTTACGAAAACCATTTCTCGAACAATGGTGTGGTTGGTGCCCCACTAACAGTGGCTGGTT
TTTTACTTGGTATGACAAAGAACGCCAAAGTGGCTTCGTTGAATCACGTCATTACCACGCATCATCC
AGTACGTGTGGCGGAAGAAGCGTGTCTACTTGACCAAATGAGTGAAGGCCGTTTTGCCTTTGGCTT
TAGTGATTGTGAAAAGAGTGCAGATATGCGCTTCTTTAATCGACCAACGGATTCTCAGTTTCAGTTG
TTCAGTGAGTGTCACAAGATCATCAATGATGCATTCACTACTGGGTACTGCCATCCAAACAATGATT
TTTATAGTTTTCCTAAAATCTCCGTTAACCCACACGCGTTCACTGAAGGCGGTCCTGCGCAATTTGT
GAATGCGACGAGCAAAGAAGTGGTTGAATGGGCGGCTAAGTTAGGGCTTCCACTCGTGTTTAGATG
GGACGACTCAAACGCTCAAAGAAAAGAATACGCCGGTTTGTACCACGAAGTTGCTCAGGCACATG
GTGTCGATGTTAGTCAGGTTCGACACAAGCTGACGCTGCTGGTCAACCAAAATGTAGATGGTGAAG
CAGCAAGGGCAGAAGCTCGCGTGTATTTGGAAGAGTTTGTCCGTGAATCTTACTCAAATACCGACT
TTGAGCAAAAAATGGGAGAGCTGTTGTCAGAAAATGCCATCGGTACTTATGAAGAAAGTACTCAGG
CAGCGCGAGTTGCGATTGAGTGTTGTGGTGCCGCGGACCTATTGATGTCTTTTGAGTCGATGGAAG
ATAAAGCGCAGCAAAGAGCGGTTATCGATGTGGTAAACGCCAACATCGTCAAATACCACTCGTAAA
AGCTTCCC3’

Table S7 Sequences of the 647-bp DNA fragment containing the promoter and signal sequence of *xopN* gene

5’CGCTGGTCACGCCGTGCATGGGCGAGCGGGTGGATCTGCAGGCGCCGGCCAATCG<br>CGTGCGTTGGTGGCGGCAGGATGCGGCGGCAGCGGCGTCGGATGGAATGGTGGTGA<br>CGCGGTAACTGGCCTGAGGCATGCCGACGCGTTGCCATGACGTTTTGATGGCACACC<br>TCGCACGCGGATGCGCGCGGCACCCGTCGCCGCGTGACGCTTCACCAACCCTGCCA<br>TGGGAAGCAGCGCACCCGACCTTGGCGCCCACACCTACAGGGGGATTCGGGTGCAG<br>CGACATCGGTTCTGCTATCCGGCAAAGATGCACACTGTAGAGTGGTTCAATCTCTGA<br>GCAATGCGATCCGCAGTTTGATCTTTCATCGGCAACGGTTTCGTCGCGTGCTTGAAC<br>TCAGTGCTCTTTTCGAGCCATGGCGACCACGCCGCCCCTCGACAATGCGCCCAGCTC<br>AGGGCTGCGAGGCGATCGCATTCCTCGGCACCGGCCATTGATCGATGCCGCCACTGC<br>ATCAAGCGCCAGGAGAAATCTGATGAAACCTGCTGCATCTGCCAATCCGCCCAGTCG<br>CACTGTGTCAGCGTCGAGTGCTCACATCCATGAGATCGAAGAAGAAGCGTCCCAGG<br>ATGCCTCGCCCAGCCATTCGGATGCC3’
